# The circular RNA CDR1as regulate cell proliferation via TMED2 and TMED10

**DOI:** 10.1186/s12885-020-06794-5

**Published:** 2020-04-15

**Authors:** Xue Yang, Siting Li, Ying Wu, Feng Ge, Ying Chen, Qian Xiong

**Affiliations:** 1 State Key Laboratory of Freshwater Ecology and Biotechnology, Institute of Hydrobiology, Chinese Academy of Sciences, Wuhan, 430072 China; 2 Key Laboratory of Algal Biology, Institute of Hydrobiology, Chinese Academy of Sciences, Wuhan, 430072 China; 3 Graduate University of Chinese Academy of Sciences, Beijing, 100049 China; 4 College of Life Science, Yangtze University, Jingzhou, 434025 China

**Keywords:** Circular RNAs, CDR1as, Quantitative proteomics, TMED2, TMED10, miR-7

## Abstract

**Background:**

Circular RNAs (CircRNAs) are biologically active RNAs. CDR1as is one such circRNA previously reported to be a microRNA-7 (miR-7) sponge, thereby regulating associated gene expression. The specific underlying molecular mechanisms of CDR1as biology, however, remain largely unknown.

**Methods:**

We performed CDR1as knockdown in order to explore its function in cell proliferation, migration, the cell cycle, and tumorigenesis. We further employed quantitative proteomic analyses and associated bioinformatics strategies to globally assess CDR1as-regulated proteins (CRPs). Western blotting and immunofluorescence staining were used to validate the proteomic results. We additionally investigated a specific link between TMED2, TMED10, and miR-7 via a dual-luciferase reporter system, and generated CDR1as knockout cell lines via CRISPR/Cas9 editing.

**Results:**

We identified 353 proteins dysregulated upon CDR1as knockdown in 293 T cells. These CRPs were found to interact with one another and to play key roles in certain cellular pathways. Two such proteins, TMED2 and TMED10, were found to specifically contribute to the influence of CDR1as on cell proliferation. CDR1as may regulate these two TMED proteins through miR-7 sponging. We were able to further confirm these results using both CRISPRi cell lines and nude mouse models.

**Conclusion:**

This study suggested that CDR1as may regulate cell proliferation via serving as a miR-7 sponge, thereby regulating TMED2 and TMED10 expression. These results are an invaluable template for future streamlined studies of circRNAs.

## Background

Circular RNAs (circRNAs) are RNAs generated via pre-mRNA back-splicing which take the form of closed loops [1, 2]. While originally believed to be erroneously generated splicing variants, recent high-throughput sequencing studies have found many circRNAs to be highly expressed in mammalian cells, wherein they are stable and seem to have cell type-specific expression patterns [1, 3]. Indeed, many studies have found evidence that these circRNAs in fact play essential regulatory roles in both disease-associated and normal physiological processes [4–10].

CDR1as (CDR1 antisense RNA) is a 1500 nucleotide long circRNA that is produced as a closed loop from the antisense transcript of CDR1 (cerebellar degeneration-related protein 1) and is highly expressed in the brains of humans and mice [11, 12]. CDR1as contains over 70 miR-7 binding sites, thereby serving as a miR-7 sponge in cells [11, 12]. Ectopic CDR1as expression in zebrafish can drive defects in the midbrain region in a fashion phenotypically similar to miR-7 knockdown [13]. CDR1as-mediated regulation of insulin transcription in islet cells stems from its ability to alter the regulation of miR-7 and downstream target genes in a comparable fashion [14]. When the CDR1as locus was knocked out in mice, animals presented with defects in sensorimotor gating, suggesting that this binding of CDR1as to miR-7 is essential to normal brain functionality [15]. There is also evidence that CDR1as can serve as a sponge for other miRNAs including miR-876-5p and miR-135a [16, 17]. CDR1as additionally plays an oncogenic role in a range of tumor types including hepatocellular carcinoma [18–20], colorectal carcinoma [21], cholangiocarcinoma [22], esophageal squamous cell carcinoma [16], melanoma [23], non-small-cell lung cancer [24], laryngeal squamous cell carcinoma [25], and osteosarcoma [26]. There is some evidence, however, that CDR1as may be anti-oncogenic in bladder cancer [17]. This range of regulatory and phenotypic roles suggests that CDR1as plays cell type-specific roles in the context of cancer.

In order to identify putative CDR1as-regulated proteins (CRPs) in cells expressing high levels of this circRNA, we employed a quantitative proteomics-based approach. We ultimately identified 353 proteins that exhibited differential expression upon CDR1as knockdown, indicating that these were potential CRPs. Subsequent bioinformatics analyses revealed these CRPs to play key roles in essential cell regulatory pathways, indicating that CDR1as plays broad regulatory roles. Using additional in vitro and in vivo approaches, we were able to ultimately confirm that CDR1as-mediated sequestration of miR-7 may inhibit the expression of TMED2 and TMED10, thereby modulating cell proliferation.

## Methods

### Cell culture

The human 293 T (Cat. GNHu17), THLE-3 (Cat. GNHu40), Hep3B (SCSP-5045), MCF-7 (SCSP-531), A549 (SCSP-503), Huh7 (SCSP-526), MS751 (SCSP-537), and LoVo (SCSP-514) cell lines were all obtained from Shanghai Institute of Cell Biology. 293 T, Hep3B and Huh7 were purchased in 2016, THLE-3, MCF-7, A549, MS751 and LoVo cell lines were purchased in 2017. All cells have been identified by STR before purchase. During the experiment, we have performed a Mycoplasma test every month and confirm that the cells are not contaminated. DMEM (Gibco, Gaithersburg, MD) supplemented using 10% FBS (Gibco) and penicillin-streptomycin (100 μg/mL) was used for all cell culture in a 37 °C, 5% CO_2_ incubator. All siRNAs and has-miR-7-5p mimics came from Genepharm (Shanghai, China) and Lipofectamine 2000 (Invitrogen, Gaithersburg, MD) was utilized for transfection of these into cells. SiRNA transfection were performed according to the manufacturer’ manual. The siRNA sequences used for CDR1as knockdown were validated in a previous study [13]. siRNA sequences are shown in Table S1.

### Plasmid construction

Plasmids for TMED2/TMED10 overexpression were generated via the PCR amplification of the human TMED2/TMED10 coding regions, which were cloned into pcDNA3.0 digested using XbaI and HindIII. For the Dual-luciferase reporter gene assay, TMED2/TMED10 3′ UTR seed sequences from the miRTarBase database were identified [27]. The psiCheck2-TMED2 and psiCheck2-TMED10 plasmids were generated via PCR amplification of these sequences and cloning them into the psiCheck2 vector (Promega, Madison, WI). The mutated psiCheck2-TMED2-mut (GTCTTTC to CTGTTTG) and psiCheck2-TMED10-mut (GTCTTC to CTGTTG) plasmids were generated via PCR amplification of psiCheck2-TMED2/psiCheck2-TMED10. Sequencing was used to confirm that all constructs were accurately generated. The primers used for plasmid construction are listed in Table S1.

### Real-time quantitative reverse transcription-PCR (qRT-PCR)

Quantitative real-time RT-PCR (qRT-PCR) was performed according to a standard protocol. RNA was isolated via TRIzol (Invitrogen) extraction, after which cDNA was generated with the M-MLV reverse transcriptase (Promega) using random primers (Promega). SYBR Green PCR Master Mix (Roche, Germany) was then used for all qRT-PCR reactions, with GAPDH employed for normalization. PCR amplification was performed using conditions of 95 °C for 10 s, 40 cycles of 94 °C for 30 s, 60 °C for 30 s, and 72 °C for 30 s on an LightCycler 480 real-time PCR system (Roche) with SYBR Green Real-time PCR Master Mix (Takara). Divergent primers were used for CDR1as assessment. The relative levels of gene expression were calculated by the 2^−ΔΔCt^ method. Each experiment was performed in triplicate. qRT-PCR primers used are listed in Table S1.

### Cell proliferation

Transfected cells were plated in a 96-well plate at 5 × 10^3^/well in 100 μL DMEM, with triplicate samples being plated. Every other day, plates were collected and 10 μL Cell Counting Kit-8 (CCK-8) reagent (Bossed, Wuhan, China) was added into each well for 2 h at 37 °C. Absorbance at 450 nm was then measured, using readings at 650 nm for reference controls.

### Cell cycle analysis

Cell cycle progression was monitored via plating cells in 12-well plates (1 × 10^5^/well) Following transfection and a 48 h incubation, 70% ethanol was used to fix cells, which were then washed using PBS, stained using propidium iodide (Beyotime, Haimen, China), and analyzed via flow cytometry (FACSAria III, BD). The ModFit LT program (BD) was utilized for analyzing DNA within cells.

### Wound healing assay

Approximately 5 × 10^5^ cells were plated in a 35 mm cell culture dish, and after achieving confluency a straight wound was generated using a sterile 10 μL pipette tip. Cellular debris was washed away, and DMEM medium containing 1% FBS was added. Migration into the wounded area was measured and imaged every 12 h using an inverted microscope, and wound healing was determined by measuring the wounded area with the ImageJ software (NIH, Bethesda, MD).

### Protein extraction and iTRAQ labeling

Following a 48 h post-transfection incubation, 293 T cells that had been transfected using 20 nm siCDR1as or NC were collected and RIPA lysis buffer was used to extract total protein on ice for 20 min. Samples were then centrifuged at 12,000×g for 20 min at 4 °C, and protein was precipitated via incubation of samples with six volumes of ice-cold acetone at − 20 °C overnight. Samples were then again spun at 12000×g for 30 min at 4 °C, and a Bradford protein assay kit (CWBIO, China) was used to determine total protein content. Next, 10 mM D,L-dithiothreitol (DTT) was added to 100 μg of protein from each sample in order to facilitate a reduction reaction at 37 °C for 1 h. Protein alkylation was then achieved using 50 mM iodoacetamide (IAA) for 1 h, after which Trypsin Gold (Promega) was used at a ratio of 50:1 (w/w, sample: trypsin) at 37 °C for 16 h in order to digest all proteins. Desalting of the resultant peptides was performed using a Strata X-C18 SPE column (Phenomenex, Carlsbad, CA), and all samples underwent vacuum drying followed by resuspension in 0.5 M triethylammonium bicarbonate (TEAB). Digested peptides were then labeled with an iTRAQ Reagent-8Plex Multiplex Kit (AB SCIEX, CA) based on provided directions. Those peptides labeled using the 4-plex iTRAQ Tag 117 were generated from triplicate samples in the NC group mixed at an equal molar ratio. Triplicate samples of cells from the CDR1as knockdown group were labeled with iTRAQ tags 118,119, and 121. After labeling, peptides were pooled and dried via vacuum centrifugation.

### Peptide separation via high pH RP HPLC

Dried and pooled peptides were resuspended using 20 mM ammonium formate (pH 10; 200 μL) and separated with an Agilent 1200 HPLC System (Agilent, CA). Samples were then loaded into a narrow bore C18 column (2.1 × 150 mm, 5 μm, Agilent) and eluted via linear buffer B gradient, beginning at 2% buffer B and increasing by 1% per minute to 38% (buffer B: 20 mM ammonium formate in 90% acetonitrile (ACN), pH 10). Every minute, peptide eluates were isolated, with 20 fractions being collected, desalted, and vacuum-dried.

### LC-MS/MS analysis

Buffer A (0.1% formic acid, 2% ACN) was added to dried peptides. Mass spectrometry was performed with an Eksigent nanoLC Ultra 2D plus system (AB SCIEX, CA) coupled with a Triple TOF 5600 System (AB SCIEX, MA). The peptide samples underwent injection into a C18 nanoLC trap column (C18, 100 μm × 3 cm, 3 μm, 150 Å) and were washed using 2% ACN (0.1% formic acid) for 10 min at 2 μL/min. Peptides were eluted using a gradient of 5–35% ACN (0.1% FA) over 70 min with an analytical ChromXP C18 column (C18, 75 μm × 15 cm, 3 μm 120 Å) with a spray tip, and were then introduced into the mass spectrometer which was fitted with a NanoSpray III source (AB SCIEX, Canada). This mass spectrometer operated in a data-dependent manner. A cyclic series of a full mass spectrometry scans was then acquired in 250 ms periods, with a dynamic exclusion of 18 s. The total cycle time was fixed at 2.5 s. Collision-induced dissociation was performed using a rolling collision energy setting.

### Protein identification

The ProteinPilot software v5.0 (AB Sciex, MA) was used to compare MS/MS data to the Uniprot human protein database (UP000005640; 70,615 total sequences). Trypsin was chosen as the enzyme, and the maximum missed cleavage sites is set to 2, MS/MS Fragment mass tolerance was set at 0.1 Da. Precursor mass tolerance was set at 0.05 Da. Proteins were identified based on an unused score ≥ 1.3 and ≥ 2 unique peptides. CRPs were those proteins where, upon comparing the CDR1as knockdown group and the control group, a |Z score| ≥ 1.96 was identifiable. All raw data were deposited in the PeptideAtlas database (PASS01007).

### Western blotting

RIPA was used to lyse cells as above, after which a BCA (Beyotime, China) assay was used to quantify protein content. Cell lysates were separated using 12% SDS-PAGE gels, followed by transfer onto PVDF membranes (Millipore, Bedford, MA). Blots were blocked using 5% skim milk in TBST for 1 h, and were then probed with primary antibodies (1:1000; Proteintech Group, Wuhan, China) including rabbit polyclonal anti-CTGF, anti-TMED2, anti-TMED10, anti-TGFB, anti-JUN, anti-FER, anti-STMN1, and polyclonal mouse anti-GAPDH at 4 °C overnight. After washing, appropriate secondary antibodies against rabbit/mouse IgG were used to probe blots (1:3000; Proteintech Group). An Image Scanner (GE Healthcare) was utilized in order to visualize protein levels, with ImageJ being used for determination of densitometry.

### Bioinformatics

A KEGG pathway [28] analysis was conducted as a means of exploring which pathways were enriched for CRPs. In addition, a Gene ontology (GO) enrichment analysis was performed with the Cytoscape BiNGO plugin [29]. Protein-protein interaction (PPI) networks were further generated with the STRING database v10.0 [30], and Cytoscape v3.2.1 (http://www.cytoscape.org) was employed for network visualization. In addition, miR-7 targets were identified with the miRTarBase database [27]. Default parameters were used for bioinformatics analysis.

### Luciferase reporter assays

Cells were plated in 12-well plates (2 × 10^5^/well) overnight, after which they were co-transfected with 0.5 μg psiCheck2-TMED2/ psiCheck2-TMED2-mut and 10 nM miR-7 mimics/NC, or with 0.5 μg psiCheck2-TMED10/psiCheck2-TMED10-mut and miR-7 mimics/NC by using Lipofectamine 2000. After 48 h, luciferase activity was determined via a Dual-Luciferase Reporter Assay System (Promega, Madison, WI).

### RNA FISH assay

Cells were plated in 20 mm dishes until 50% confluent, after which they were washed twice in PBS, fixed for 30 min with 4% formaldehyde, washed twice, permeabilized using 0.1% Triton X-100 for 5 min, and resuspended in 2X SSC and 50% formamide for 5 min. Next, prehybridization was performed with hybridization buffer (2 mM vanadyl ribonucleotide complex, 25% deionized formamide, 250 μg/mL N-50 DNA, 10% dextran sulfate, 2 × SSC, 1 mg/mL yeast tRNA, 0.002 mg/mL BSA) at 37 °C for 1 h. An AF647-labeld CDR1as probe (250 ng) was then added to each dish for 5 min at 65 °C followed by overnight hybridization at 37 °C. Next, 2 × SSC and 50% formamide was used to wash all samples for 1 h, and 4′,6-diamidino-2-phenylindolein (DAPI) counterstaining was performed. After an additional wash, an LSM 710 laser scanning confocal microscope (Carl Zeiss, NY) was used for cell imaging. Probe sequences are given in Table S1.

### Immunofluorescence staining

After fixation for 15 min in 4% paraformaldehyde and washing with PBS, cells underwent permeabilization using 0.5% Triton X-100 in PBS for 15 min, and were blocked in 10% BSA. Samples were then probed overnight with primary antibodies (1:200) at 4 °C. Samples were next washed and probed using secondary Dylight488 Goat anti-Rabbit IgG (H + L) (1:1000, Abbkine, CA), followed by DAPI counterstaining. Cells were then washed and imaged via confocal microscopy as above.

### CRISPR/Cas9-mediated CDR1as knockout

In order to knockout CDR1as via CRISPR, two sgRNAs were designed via the CRISPR design website (http://crispr.mit.edu/), and were added into the lentiCRISPR v2 plasmid. Cells were then plated in 6-well plates (4 × 10^5^/well) and transfected with these plasmids. After 24 h of culture and puromycin selection, single cells were sorted into 96-well plates. After a 3 week culture period, genomic DNA was extracted from cells for PCR and sequencing reactions, and those with target gene locus knockouts were maintained for further study.

### Xenograft animal model

Female BALB/c nude mice (5 weeks old) were obtained from the Animal Experiment Center/Animal Biosafety Level-III Laboratory of Wuhan University. A barrier system (SPF level) was used for the feeding and housing of all animals under controlled conditions provided by the Animal Experiment Center of Wuhan University. A total of 293 T cells (1 × 10^7^/animal) that had been transfected with siCDR1as or NC were then injected into right armpit of mice. A total of ten mice were used in this study, with five in each group. After 19 days, animals were euthanized by cervical dislocation and tumor samples were isolated for PCR and Western blotting analyses. All animal studies were performed consistent with the approval and requirements of the Institutional Animal Care and Use Committee of the Animal Experiment Center/Animal Biosafety Level- III Laboratory of Wuhan University (SPF level). The IACUC permit number is 2017103.

### Statistical analysis

All experiments were conducted using 3 biological replicates, and Student’s t-tests (two-tailed) were used for all statistical analyses with a significance threshold of *P* < 0.05. All data are presented as the means ± SD.

## Results

### The role of CDR1as in 293 T cells

**Fig. 1:**
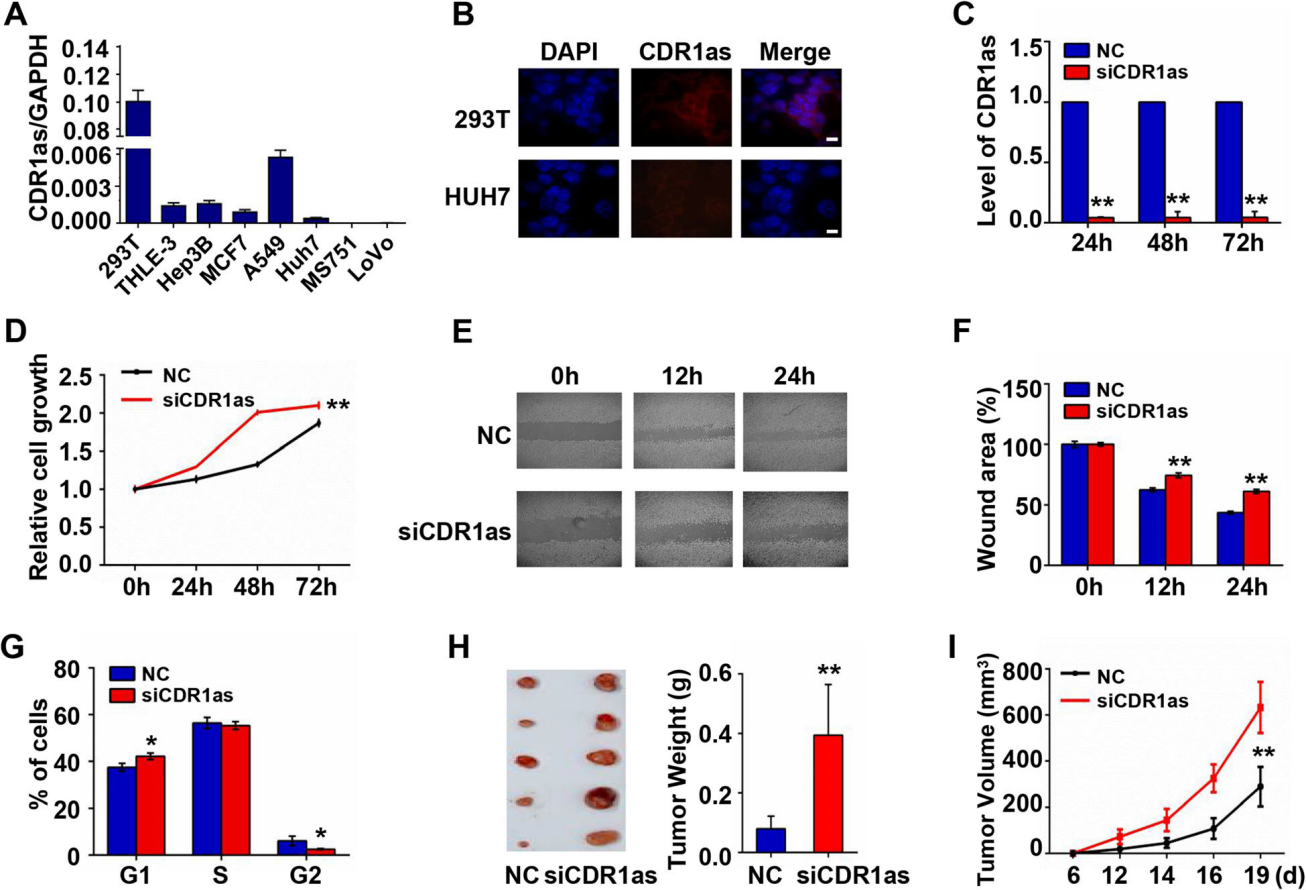
Functional effects of CDR1as inhibition in 293 T cells. **a** Relative CDR1as expression in different cell lines. **b** RNA FISH assessment of CDR1as in 293 T and Huh7 cells. Scale bar, 10 μm. **c** CDR1as knockdown efficiency following siCDR1as transfection. **d** CDR1as knockdown effects on cell proliferation. **e** & **f** CDR1as knockdown effects on cell migration. **g** CDR1as knockdown effects on cell cycle progression. **h** Xenograft assessment of tumor size and weight in nude mice using 293 T cells in which CDR1as was knocked down (*n* = 5). **i** Tumor volumes from mice in (**h**)

We began by measuring the expression of CDR1as in a variety of cell lines, revealing it to be expressed at high levels in the 293 T, THLE-3, Hep3B, MCF-7, and A549 cell lines (Fig. 1a). Expression was markedly elevated in 293 T cells, with levels 100 times higher than those in the Huh7, MS751, and LoVo cell lines. We further confirmed this high CDR1as expression in 293 T cells via RNA FISH assessment, determining that circRNA is localized to the cytoplasm (Fig. 1b). Next, a loss-of function approach was used to explore the biology of CDR1as in 293 T cells, with siRNA transfection reducing CDR1as expression to less than 5% of baseline levels for at least 72 h (Fig. 1c). After CDR1as knockdown, we observed marked increases in cell proliferation (Fig. 1d), but the migratory capacity of these cells decreased substantially (Fig. 1e-f). Knockdown of CDR1as lead to marked increases in the G1 phase cell and decrease in the G2 phase cell (Fig. 1g). In a nude mice xenograft tumor model, the tumors in which CDR1as had been knocked down were significantly larger than those in which it had not (Fig. 1h-i). Together these findings suggest CDR1as plays a suppressive role in constraining the ability of 293 T cells to proliferate.

### Identification of CDR1as-related proteins in 293 T cells

**Fig. 2:**
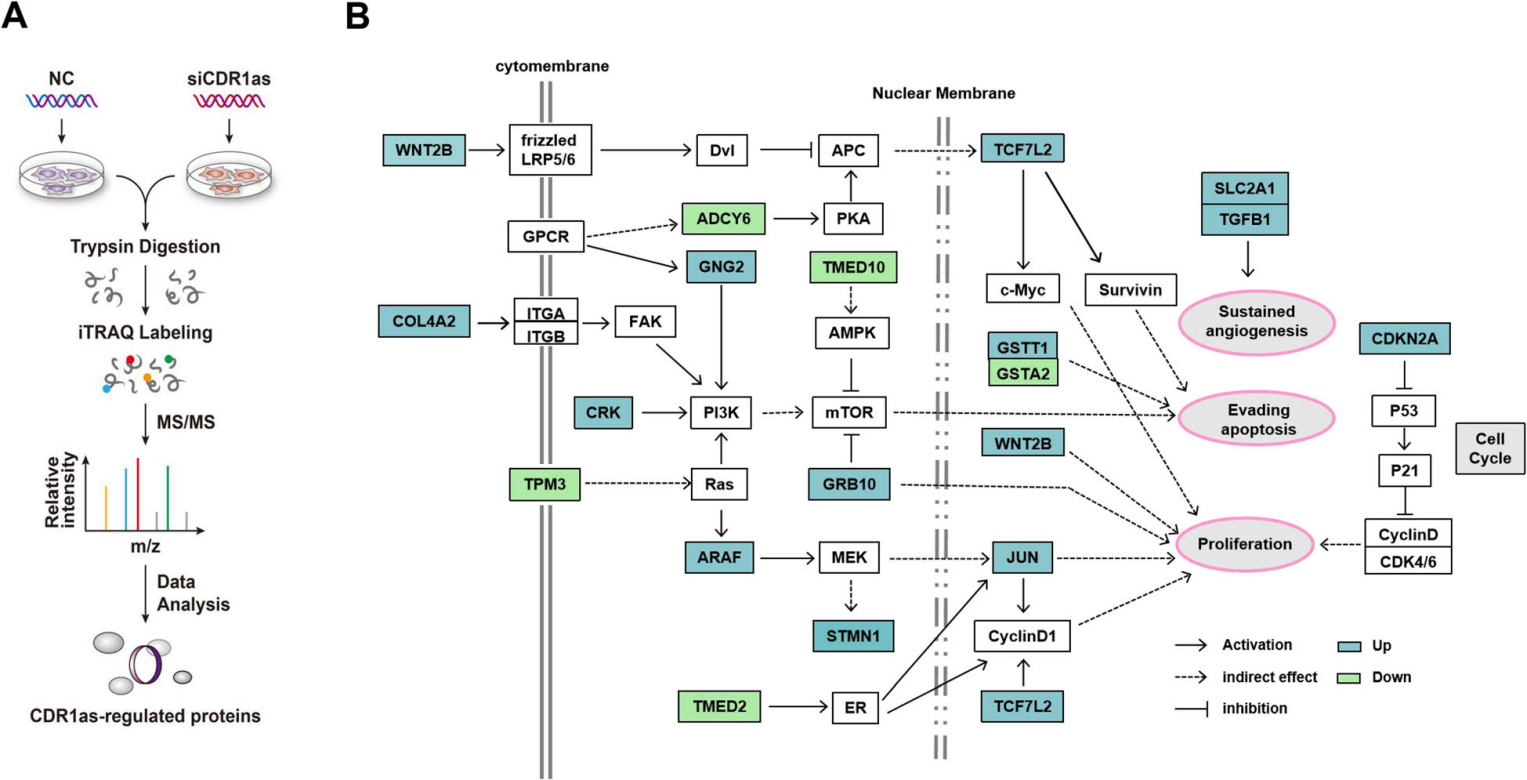
CRP identification in 293 T cells. **a** An overview of CRP identification via quantitative proteomics. **b** KEGG analysis of CRPs, with those mapping to “pathways in cancer” shown. Upregulated CRPs are marked in blue, while downregulated CRPs are marked in green

We next sought to identify those proteins affected by CDR1as knockdown in order to systematically explore the molecular mechanisms underlying the observed effects of CDR1as knockdown on cell proliferation. We therefore used an iTRAQ-based quantitative proteomics approach, identifying global CRPs in 293 T cells after CDR1as knockdown (Fig. 2a). We detected 6143 proteins in this experiment (Table S2), of which 353 were dysregulated upon CDR1as knockdown (Table S3), including 235 upregulated and 118 downregulated proteins.

### Bioinformatics analysis and validation of CRPs

**Fig. 3:**
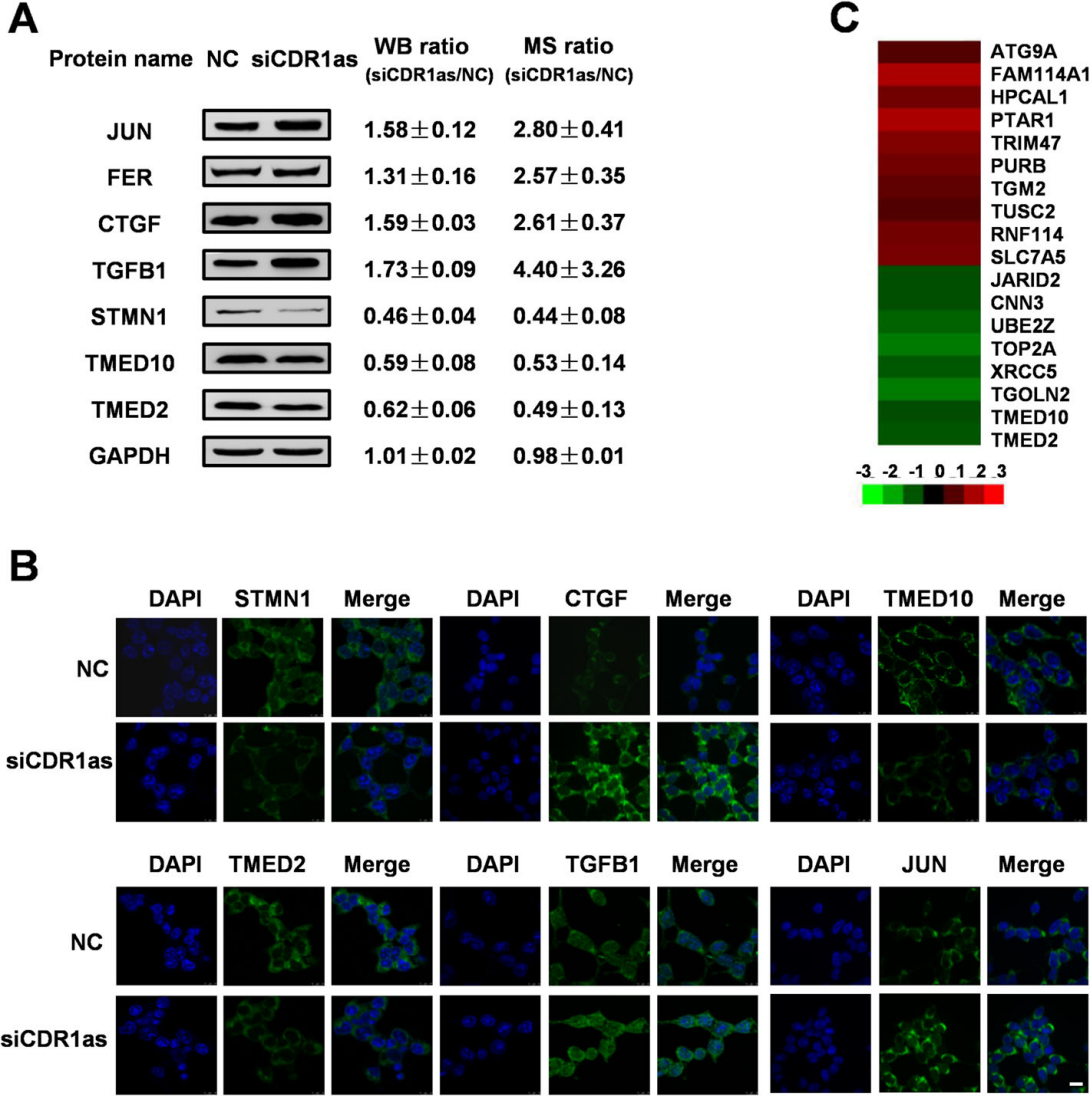
Proteomic data validation. **a**&**b** CRPs involved in “pathways in cancer” were validated via western blotting (cropping of blots, full-length blots are presented in Fig. S4) (**a**) and immunofluorescent microscopy (**b**) following CDR1as knockdown in 293 T cells. Scale bar, 10 μm. (**c**) miR-7 targets among these CRPs

Having identified proteins affected by CDR1as in 293 T cells, we next conducted bioinformatics analyses to explore the functionality of these CRPs. A KEGG analysis indicated that these proteins play roles in metabolic pathways, cancer related pathways, the Rap1 signaling pathway, mTOR signaling pathway and the MAPK signaling pathway (Fig. S1A), with 18 proteins mapping to pathways in cancer (Fig. 2b) which were primarily linked to cell proliferation and the cell cycle. Next, a GO enrichment analysis was conducted to explore the biological processes (BP), molecular functions (MF) and cellular components (CC) represented among CRPs (Fig. S1B & Table S4). Enriched GO terms for this CRP dataset included “DNA conformational change”, “nucleosome organization”, and “protein binding”. A PPI network was additionally generated in order to explore interactions between CRPs (Fig. S2 & Table S5). Roughly 70% of these 353 CRPs were represented in this network, suggesting there are close ties in their regulation and biological functionality in cells. A total of 7 CRPs (JUN, FER, CTGF, TGFB1, STMN1, TMED2, and TMED10), which participate in “pathways in cancer” and are known to play potential regulatory roles were next selected for Western blotting (Fig. 3a) and immunofluorescence (Fig. 3b) validation, which confirmed the accuracy and robustness of our proteomics data.

### CDR1as modulates cell growth via regulating TMED2 and TMED10

**Fig. 4:**
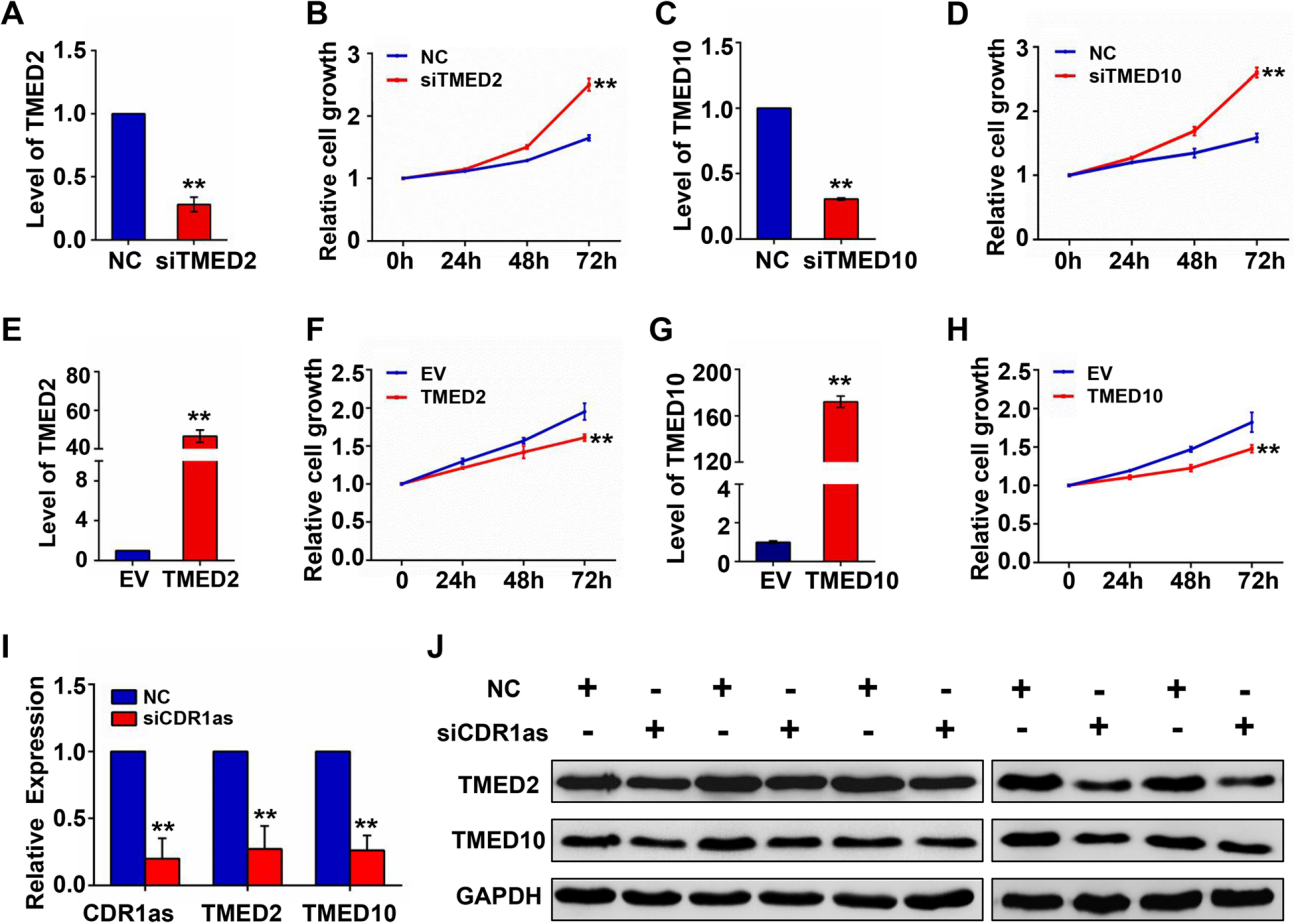
Functional effects of TMED2/TMED10 knockdown or overexpression in 293 T cells. **a** & **c** TMED2 and TMED10 knockdown efficiency. **b** & **d** TMED2 or TMED10 knockdown effects on cell proliferation. **e** & **g** TMED2 and TMED10 overexpression results. **f** & **h** TMED2 or TMED10 overexpression effects on cell proliferation. **i** Results of a xenograft assay in nude mice using 293 T cells following transient CDR1as knockdown. CDR1as, *TMED2*, and *TMED10* expression levels were measured via qRT-PCR. **j** Levels of TMED2 and TMED10 expression in xenografts in nude mice were measured via western blotting (cropping of blots, full-length blots are presented in Fig. S5)

CDR1as is a well-characterized miR-7 sponge [11, 14], and as such the fact that 18 of the identified CRPs were putative miR-7 targets (as determined with the miRTarBase database [27]) was perhaps unsurprising. Of these, 8 were significantly downregulated following CDR1as knockdown (Fig. 3c). Among these 8 proteins, TMED2 and TMED10 were also found above to be involved in “pathways in cancer” (Fig. 2b), and were therefore chosen as subjects of further research. We initially confirmed that the expression of TMED2 and TMED10 was significantly suppressed in additional high CDR1as-expressing cell lines upon CDR1as knockdown (Fig. 3d). We then conducted loss-of-function and gain-of function analyses to gauge the importance of TMED2 and TMED10 as regulators of CDR1as functionality. We found that when either of these proteins was knocked down, cell proliferation increased markedly (Fig. 4a-d), whereas overexpression of these proteins suppressed cell proliferation (Fig. 4e-h). These results were further confirmed in a xenograft nude mouse model, which exhibited TMED2 and TMED10 downregulation in CDR1as knockdown xenograft animals relative to controls (Fig. 4i-j). Together these findings indicate that CDR1as acts to promote cell proliferation at least in part via the regulation of TMED2 and TMED10 expression.

### TMED2 and TMED10 are miR-7 targets

**Fig. 5:**
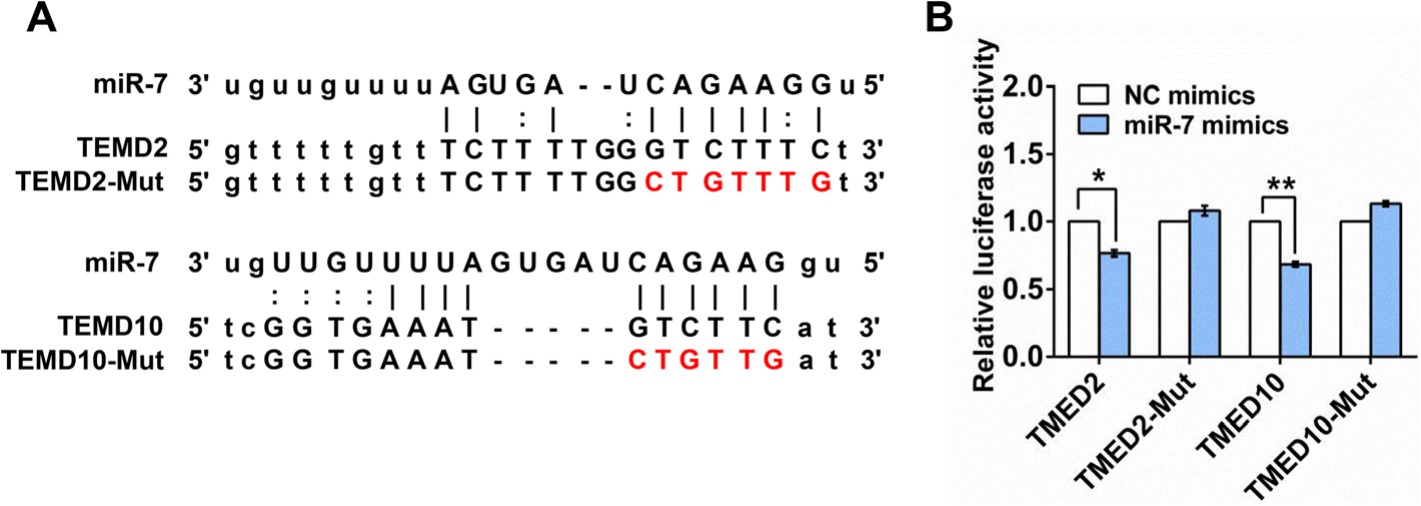
CDR1as knockdown downregulates expression of the miR-7 targets TMED2 and TMED10. **a** The miR-7 binding sites in *TMED2/TMED10* mRNA are shown. **b** A luciferase assay revealed that TMED2 and TMED10 are miR-7 targets

Next, luciferase assays were conducted in order to confirm that TMED2 and TMED10 are miR-7 targets, as predicted above. These assays were performed via cloning the 3′ UTR fragments of TMED2 or TMED10 containing the putative miR-7 binding sites or mutated forms thereof into the psiCheck2 Vector (Fig. 5a). We found that luciferase activity was significantly reduced in 293 T cells following the co-transfection of miR-7 and psiCheck2-TMED2 or psiCheck2-TMED10 into 293 T cells, whereas luciferase activity was unaffected when plasmids containing mutated miR-7 binding sites were instead co-transfected into these cells (Fig. 5b). These results confirmed that TMED2/TMED10 are miR-7 targets, suggesting that CDR1as may regulate the expression of these genes via serving as a miR-7 sponge and thereby mediating the effective upregulation of these miR-7 targets.

### CRISPR-mediated generation of CDR1as CRISPRi cell lines

**Fig. 6:**
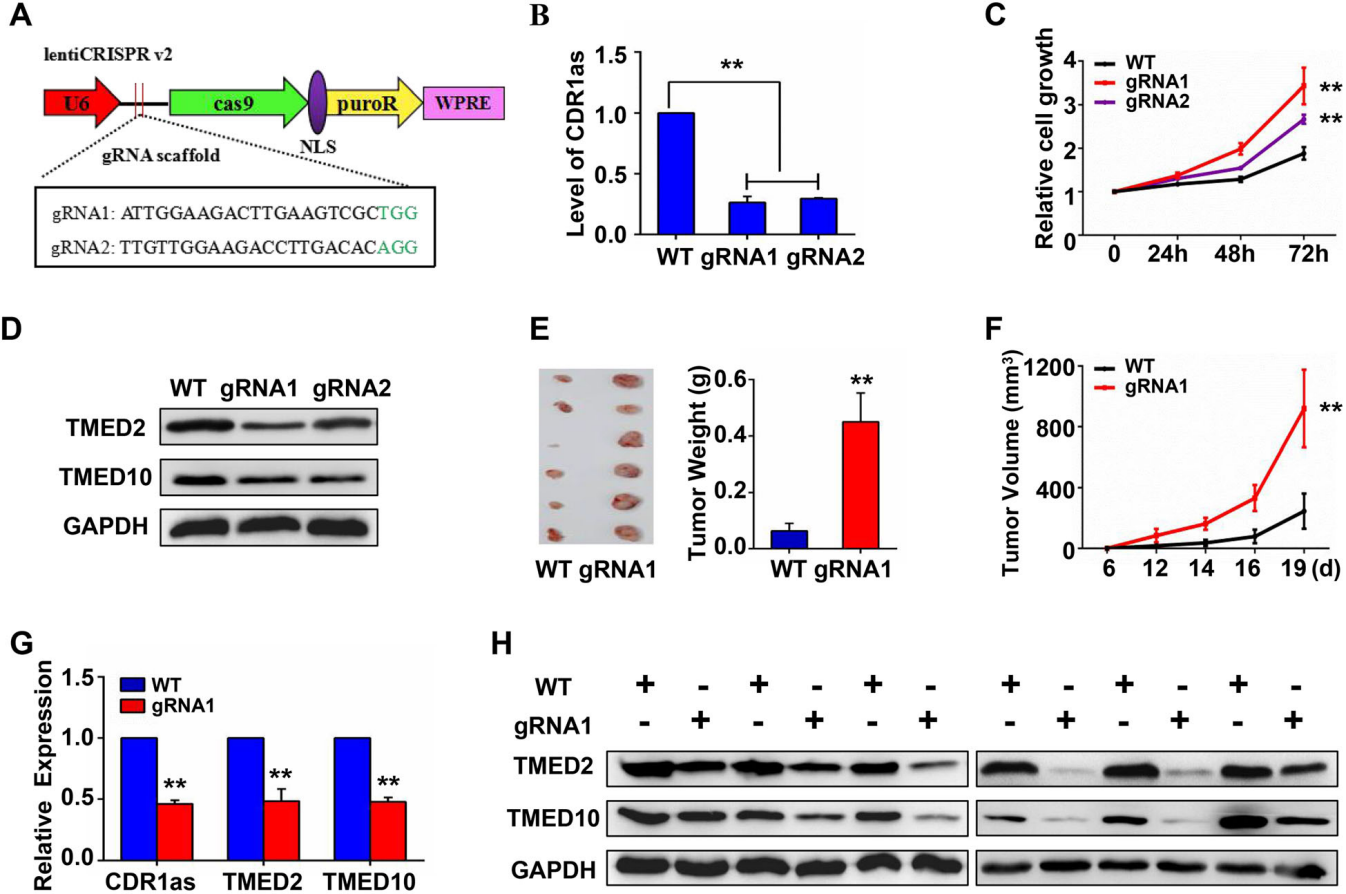
Validation of TMED2 and TMED10 expression in CDR1as CRISPRi cell lines. **a** Generation of CDR1as knockout cell lines using CRISPR/Cas9. A map of lentiCRISPR V2 vector and CDR1as deletion sites is shown. **b** CDR1as expression in CRISPRi cell lines. **c** Cell proliferation of CDR1as CRISPRi 293 T cells relative to WT cells. **d** TMED2 and TMED10 expression in CDR1as CRISPRi 293 T or WT cells as determined by western blotting (cropping of blots, full-length blots are presented in Fig. S6). **e** A xenograft assay conducted in nude mice using CDR1as CRISPRi 293 T cells. Tumor size and tumor weight are shown. **f** Tumor volumes from mice in (**e**). **g** Tumor CDR1as, TMED2, and TMED10 expression as determined via qRT-PCR. **h** Measurements of TMED2 and TMED10 via western blotting (cropping of blots, full-length blots are presented in Fig. S7)

CRISPR-mediated gene editing is a powerful technology that has been successfully used to knock out specific lncRNAs and circRNAs [31–34]. We therefore sought to use this approach to knock out CDR1as in 293 T cells, using two sgRNAs targeting the CDR1 locus containing the CDR1as sequence (Fig. S3A). These guides were clones into lentiCRISPR v2 plasmids (Fig. 6a), which were transfected into 293 T cells that then underwent puromycin selection. CDR1as CRISPRi cell lines were then generated via flow cytometry-assisted single cell sorting and culture, with sequencing used to confirm target gene knockout (Fig. S3B). We further confirmed that negligible CDR1as expression was detectable in these knockout cell lines (Fig. 6b). Importantly, cell proliferation was enhanced in these CDR1as CRISPRi lines compared to parental 293 T cells (Fig. 6c), and TMED2/TMED10 were significantly downregulated in these cells (Fig. 6d), consistent with our siRNA results.

### CDR1as knockout promotes tumor growth and downregulates TMED2 and TMED10 in nude mice

We next established a nude xenograft mouse model in order to confirm our above findings in vivo. When mice were injected with the CDR1as CRISPRi cell line, there was a marked increase in the weight and volume of tumors (Fig. 6e-f), and CDR1as downregulation was maintained in these cells (Fig. 6g). Further qPCR (Fig. 6g) and western blotting (Fig. 6h) confirmed TMED2 and TMED10 downregulation in this CDR1as knockdown model relative to control xenografts, confirming that CDR1as can regulate the expression of TMED2 and TMED10 in vivo.

## Discussion

There is increasing evidence that circRNAs regulate the expression of specific genes and regulate a variety of biological processes [5, 35, 36]. CDR1as in particular is known to play key roles in a wide range of cancer types [14, 16]. CDR1as expression varies across tissues and cell lines [17, 18, 21, 26], but there is currently a lack of information regarding the functional importance of CDR1as in cell lines expressing high levels of this circRNA.

In this study we used a quantitative proteomics approach to identify a total of 353 proteins that were differentially regulated following CDR1as knockdown in 293 T cells, offering systematic insights into the biology of CDR1as. KEGG pathway analyses revealed these CRPs to be enriched for key cellular pathways including “pathway in cancer”, “Rap1 signaling”, “mTOR signaling” and “MAPK signaling”, which are closely linked to cell proliferation, cell migration, apoptosis, and the cell cycle [37–39]. Nearly 70% (241/353) of these CRPs were incorporated into a single protein-protein interaction network, indicating a close functional relationship among these CRPs.

Many studies have found that circRNAs can serve as sponges for miRNAs, and as such they have also been referred to as competing endogenous RNAs (ceRNAs), competitively sequestering miRNAs and preventing them from influencing the expression of their targets [40–43]. CDR1as is well known to serve as a sponge for miR-7, regulating the expression of miR-7 target genes accordingly [14, 17, 44]. miR-7 has well documented roles in controlling cell proliferation, migration, survival and invasion [45–48]. Among our identified CRPs, 18 were predicted miR-7 targets, including both TMED2 and TMED10, which are both linked to oncogenic pathways. TMED2/TMED10 are EMP24/GP25L family members that co-localize together and play roles in regulating vesicular protein trafficking, serving as a cargo receptor [49, 50]. These proteins have also been shown to play key roles in cancer cells, influencing the AMPK/mTOR pathway and the TGF-β signaling pathway, and regulating apoptosis and cell proliferation [51–54]. More broadly, TMED2 and TMED10 are also linked to disorders and diseases including Alzheimer’s disease (AD) [55], kidney disorders, and diabetes [56–58]. In this study, we found both TMED2 and TMED10 to be downregulated upon CDR1as knockdown, and luciferase assays confirmed them to be miR-7 targets (Fig. 5). We further found that CDR1as mediates its effects on cell proliferation at least in part via TMED2/TMED10. These results were further confirmed in both animal models and CRISPRi cell lines (Fig. 6). Together these results reveal that CDR1as may regulate cell proliferation by serving as a miR-7 sponge, thereby modulating the expression of TMED2/TMED10 in 293 T cells. There was also evidence of a similar mechanism in other high CDR1as-expressing cell lines, suggesting this is a broadly relevant regulatory mechanism (Fig. 3d).

We have found in this study that CDR1as regulates cell proliferation, and in a previous study we found that overexpressing CDR1as in HCC cells that normally express low levels of this circRNA can promote proliferation via regulating miR-7 targeting of EGFR [18]. Overexpression and knockdown of CDR1as can thus influence cell proliferation in a similar manner, as has been observed for other regulatory RNAs [10, 23, 59]. For example, the circRNA circHIPK3 has been shown to suppress cell proliferation in Huh7, HCT-116, HeLa, and 293 T cells [10], whereas its overexpression enhances proliferation in osteosarcoma (OS) cells [23]. Similarly, the lncRNA HOTAIR exhibits significant cell-type-specific effects within the same cancer tissue [59]. Urothelial carcinoma (UC) VM-CUB1 cells grew faster upon HOTAIR overexpression, whereas the UC 5637 cells grew slower upon similar overexpression [59]. These results indicate that CDR1as and other regulatory RNAs mediate their effects based not only on their expression, but also in a cell/developmental stage-specific manner, with functional effects varying based on the tissue in question. CDR1as may also form a component of a complex which negatively regulates cell proliferation, and upon its overexpression or knockdown such a complex may not form correctly, thereby enhancing proliferation as in the case of the splicing kinase SRPK1 [60]. There may also be a range of feedback mechanisms further regulating CDR1as expression and regulatory activity.

## Conclusions

**Fig. 7:**
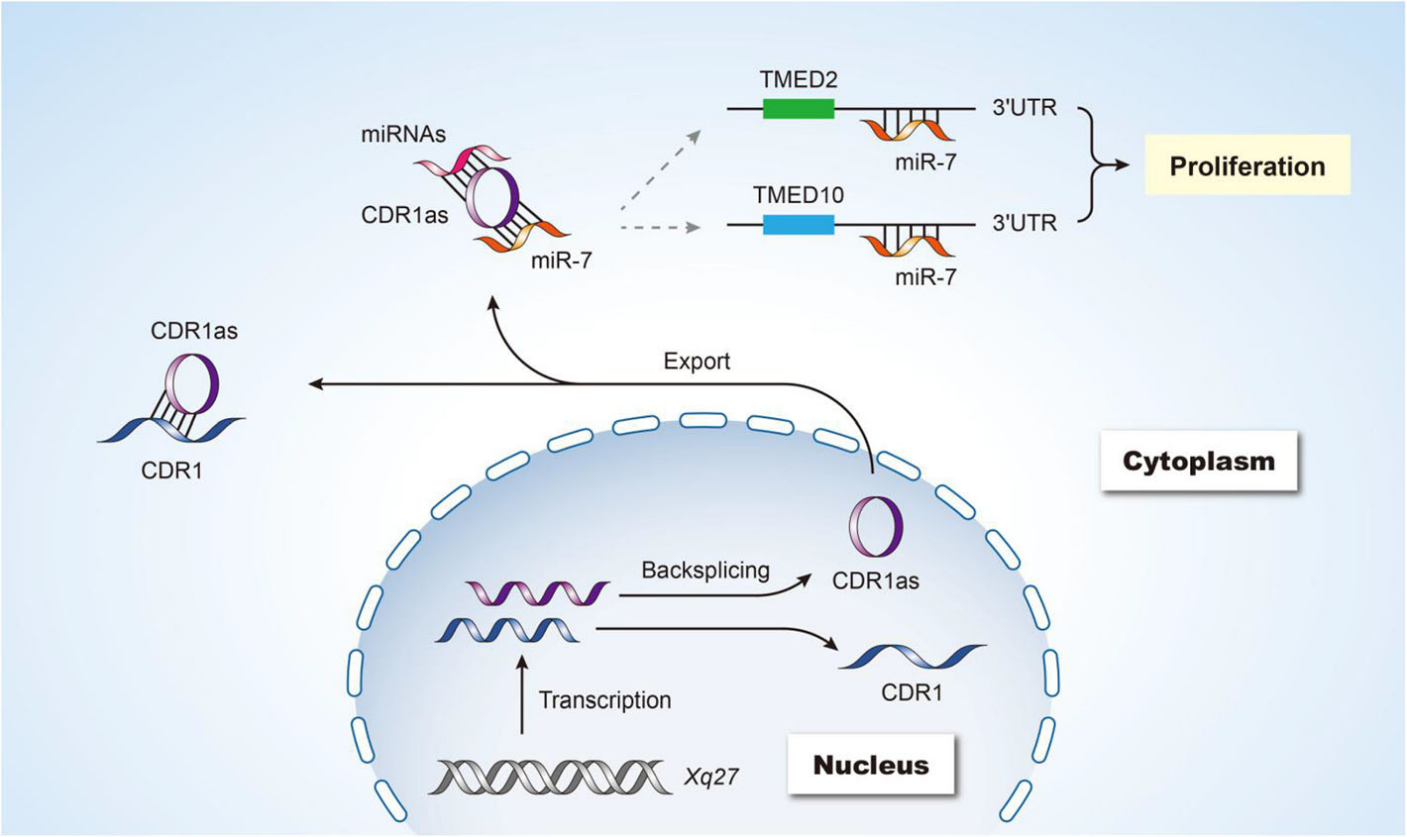
Proposed CDR1as regulatory model. As a CDR1 antisense transcript, this circRNA can form a duplex with CDR1, stabilizing it. In addition, it can act as a sponge for miR-7, regulating the expression of the targets of this miRNA. We found that CDR1as can also regulate cell proliferation, at least in part via serving as a miR-7 sponge and thereby regulating the expression of TMED2 and TMED10

Together these results suggest that the biology of CDR1as is very complex, with multiple distinct elements interacting to mediate the observed cell type-specific phenotype (Fig. 7). As a natural antisense transcript of CDR1, it can interact with and stabilize CDR1 via duplex formation. It can additionally serve as a miR-7 sponge to regulate associated target gene expression [16, 17]. In addition, we found that CDR1as regulates cell proliferation by inhibiting TMED2 and TMED10, the targets of miR-7.

## Supplementary information

**Additional file 1.**

**Additional file 2.**

**Additional file 3.**

**Additional file 4.**

**Additional file 5.**

**Additional file 6.**

## Data Availability

All the data obtained and materials analyzed in this research are available with the corresponding author upon reasonable request.

## Abbreviations

circRNA: circular RNA
miR-7: microRNA-7
ceRNA: competing eRNA
qRT-PCR: quantitative real-time PCR
CRP: CDR1as-regulated protein
GO: Gene ontology
TMED2/10: TMED2 and TMED10
