# Supplementary material for: The circular RNA CDR1as regulate cell proliferation via TMED2 and TMED10
Source: BMC Cancer. 2020 Apr 15;20:312. doi: 10.1186/s12885-020-06794-5 (PMC7160961; doi:10.1186/s12885-020-06794-5)
Supplement: Supplementary file 1 — Additional file 1. [file 12885_2020_6794_MOESM1_ESM.doc]

**The circular RNA CDR1as serves as a miR-7 sponge to regulate cell proliferation via TMED2 and TMED10**

Xue Yang^1,2,3^, Siting Li^1,2,3^, Ying Wu^1,2,3^, Feng Ge^1,2,3^, Chen Ying^4^, Qian Xiong^1,2,3^*

^1^ State Key Laboratory of Freshwater Ecology and Biotechnology, Institute of Hydrobiology, Chinese Academy of Sciences, Wuhan 430072, China

^2^ Key Laboratory of Algal Biology, Institute of Hydrobiology, Chinese Academy of Sciences, Wuhan 430072, China

^3^ Graduate University of Chinese Academy of Sciences, Beijing 100049, China

^4^ College of Life Science, Yangtze University, Jingzhou 434025, China

*To whom correspondence should be addressed: Qian Xiong, Institute of Hydrobiology, Chinese Academy of Sciences, E-mail: xiongqian@ihb.ac.cn. Phone/Fax: +86-27-68780730

**Figure S1** KEGG pathway enrichment (A) and GO enrichment (B) analysis of the CRPs


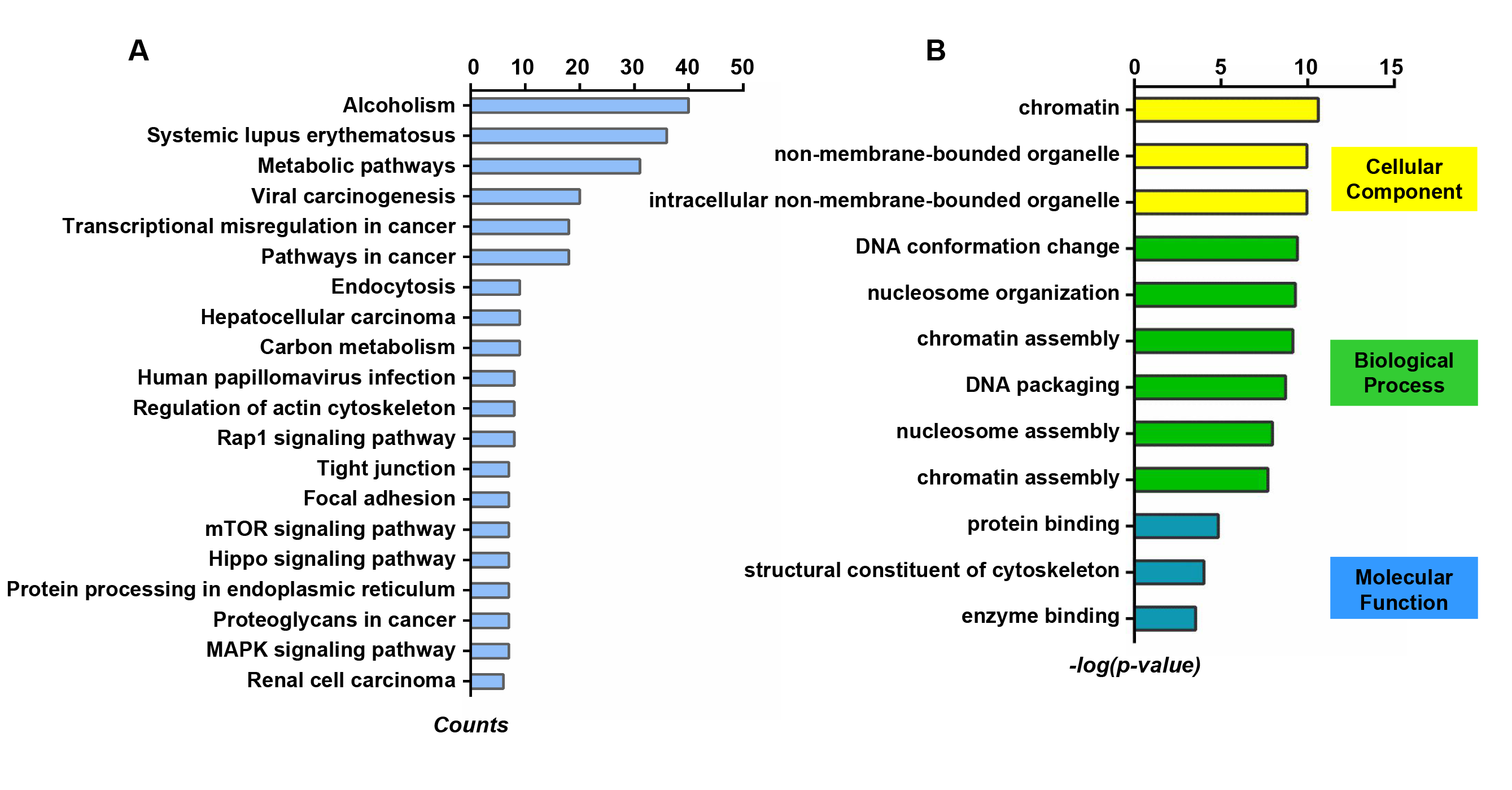


**Figure S2** Protein-protein interaction (PPI) network of CRPs. Yellow nodes represent CRPs participated in “pathways in cancer”, blue nodes are CRPs involved in the PPI network.

**
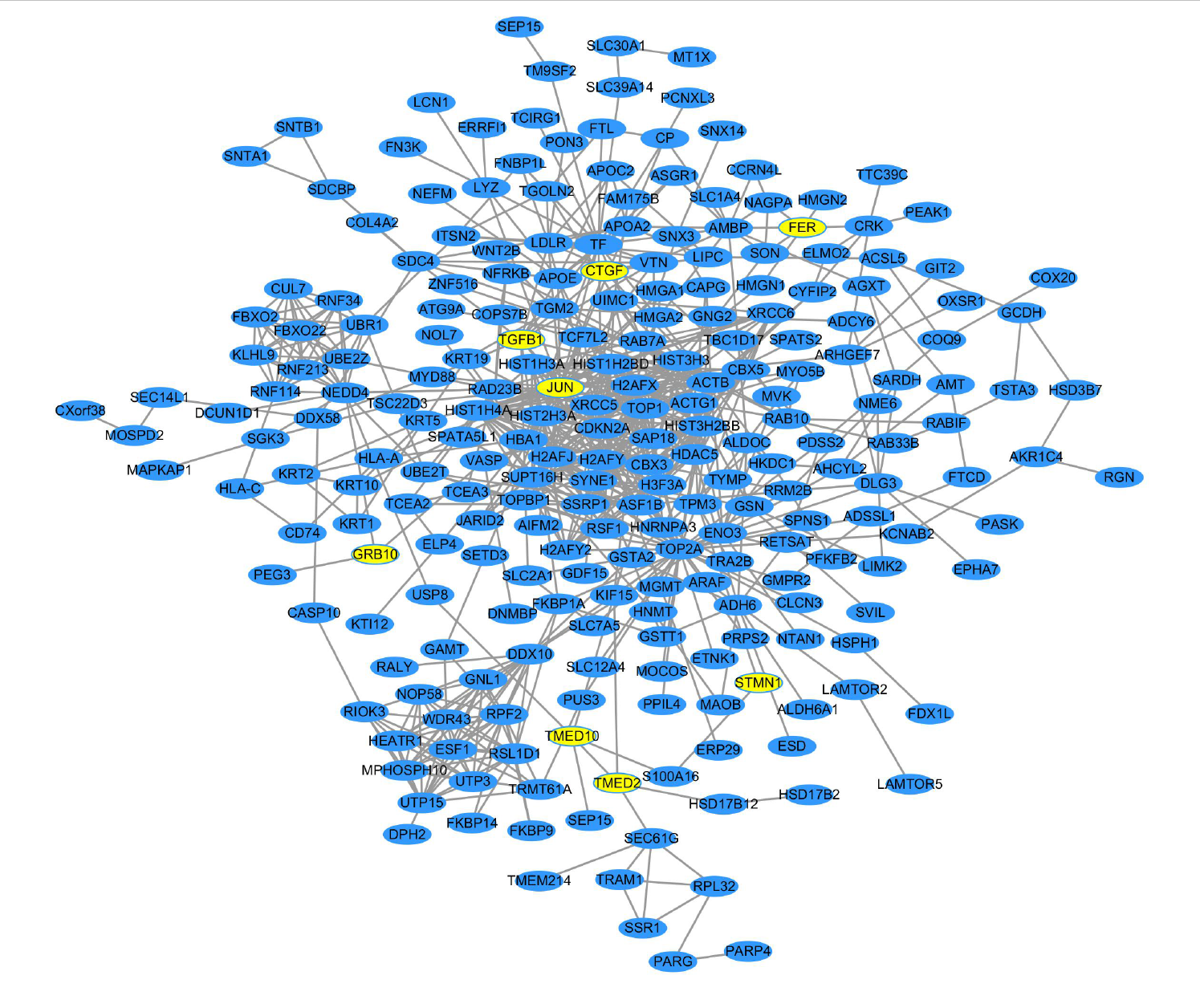
**

**Figure S3** Knockdown of CDR1as by using CRISPR/Cas9 technology. (A) Cdr1as locus was deleted using CRISPR/Cas9 technology. (B) Sequencing of target locus of CDR1as in CRISPRi cell lines.

**
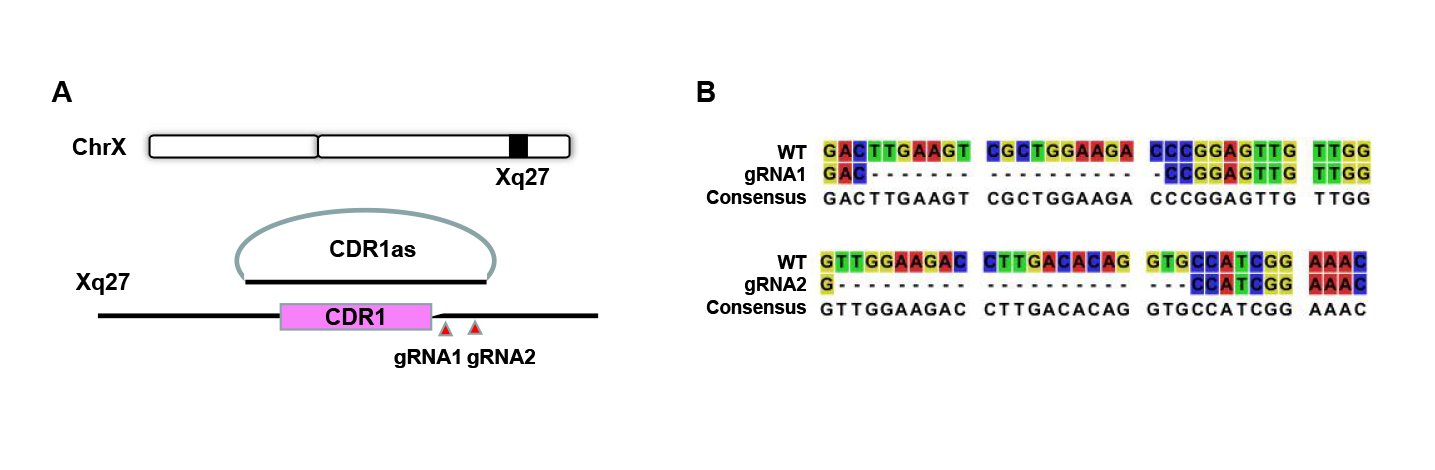
**

**Figure S4** Western blots for Fig. 3A.


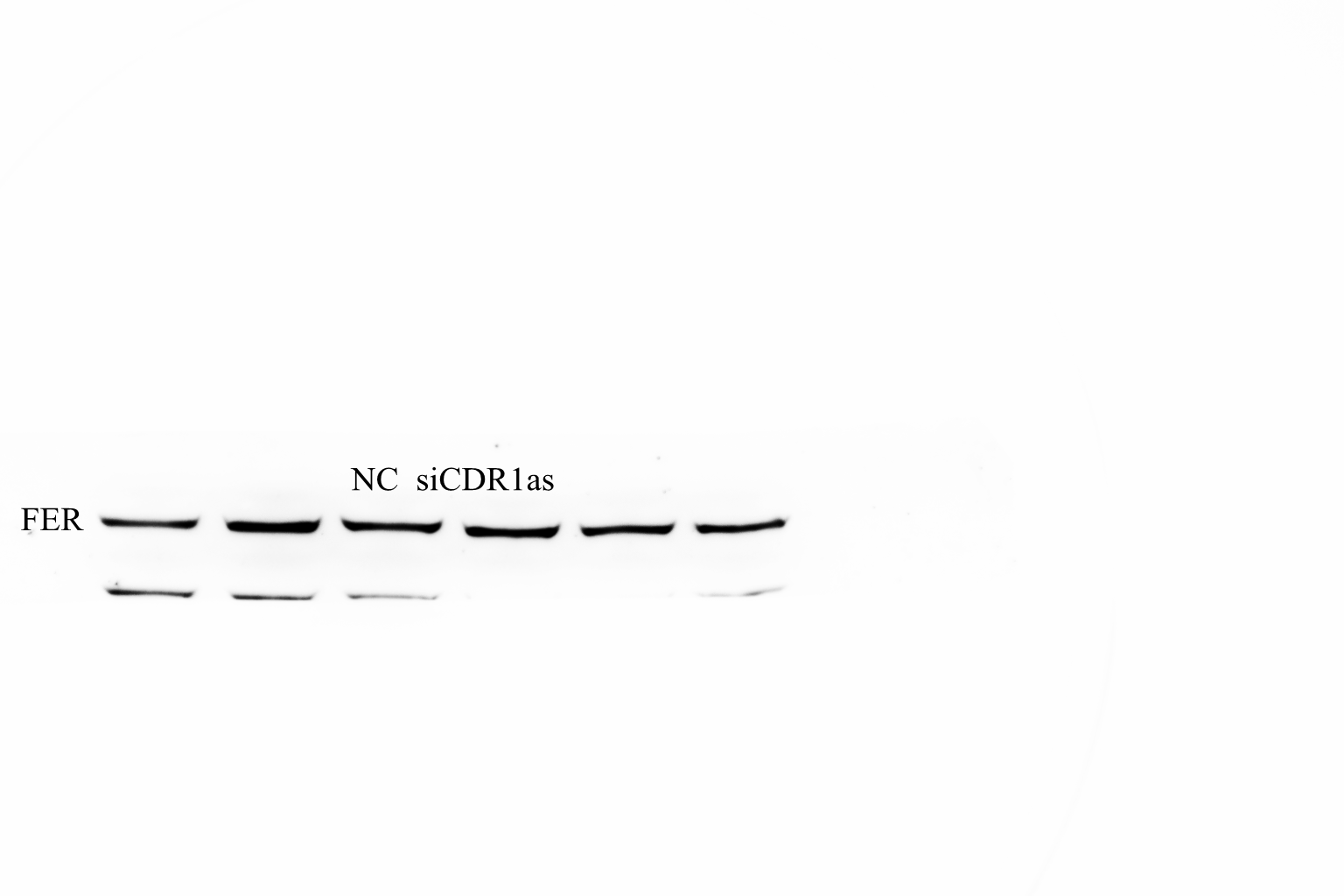


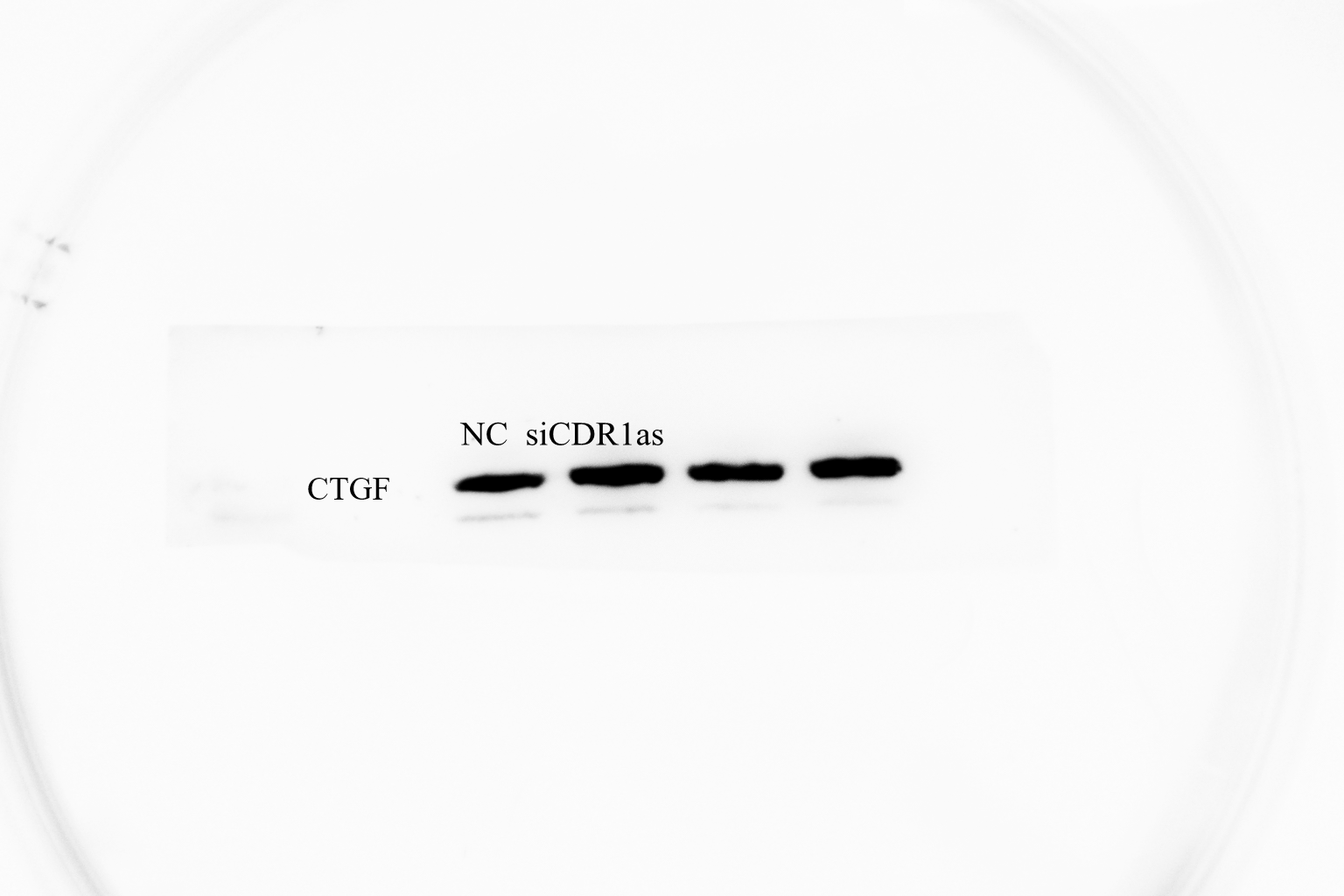


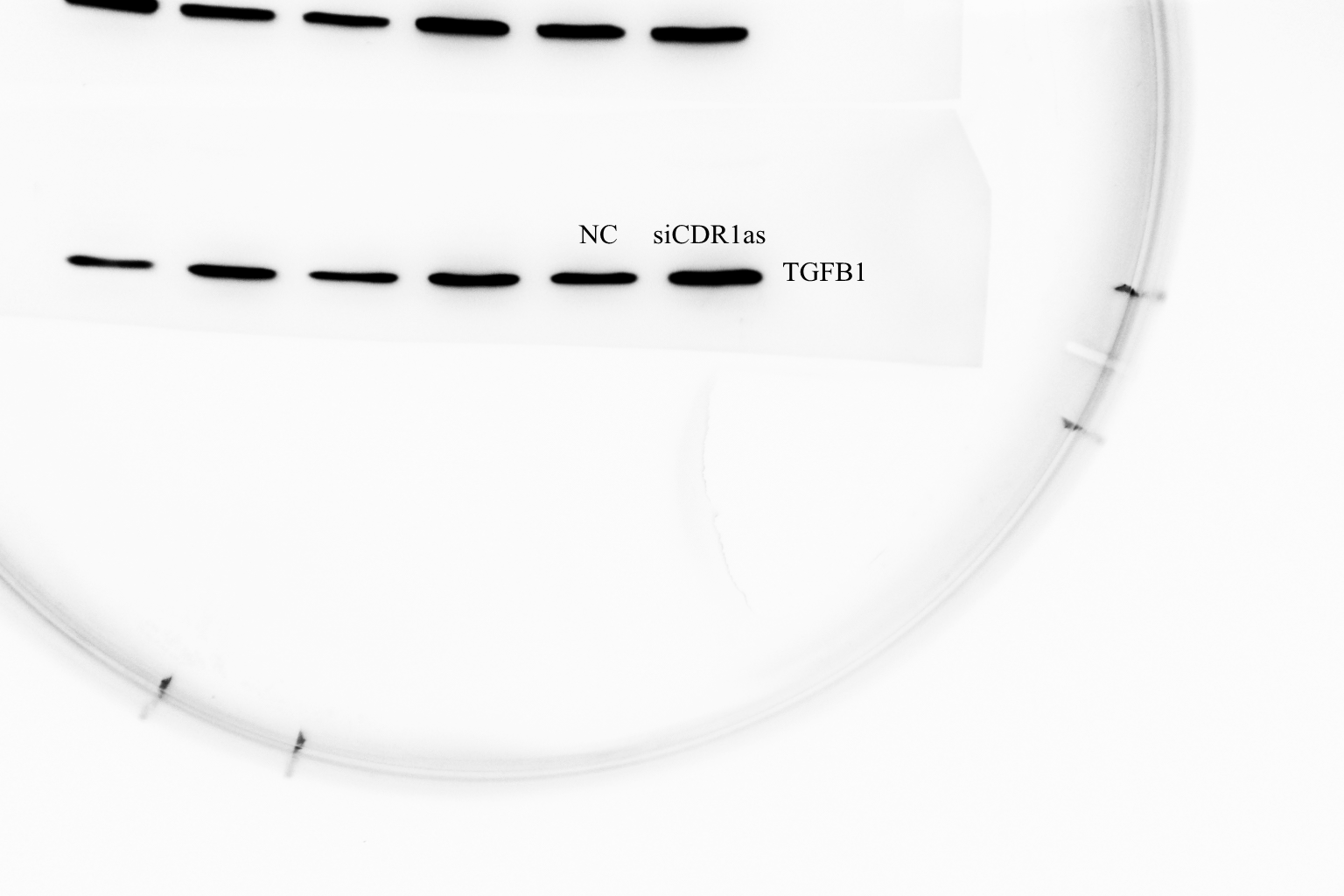


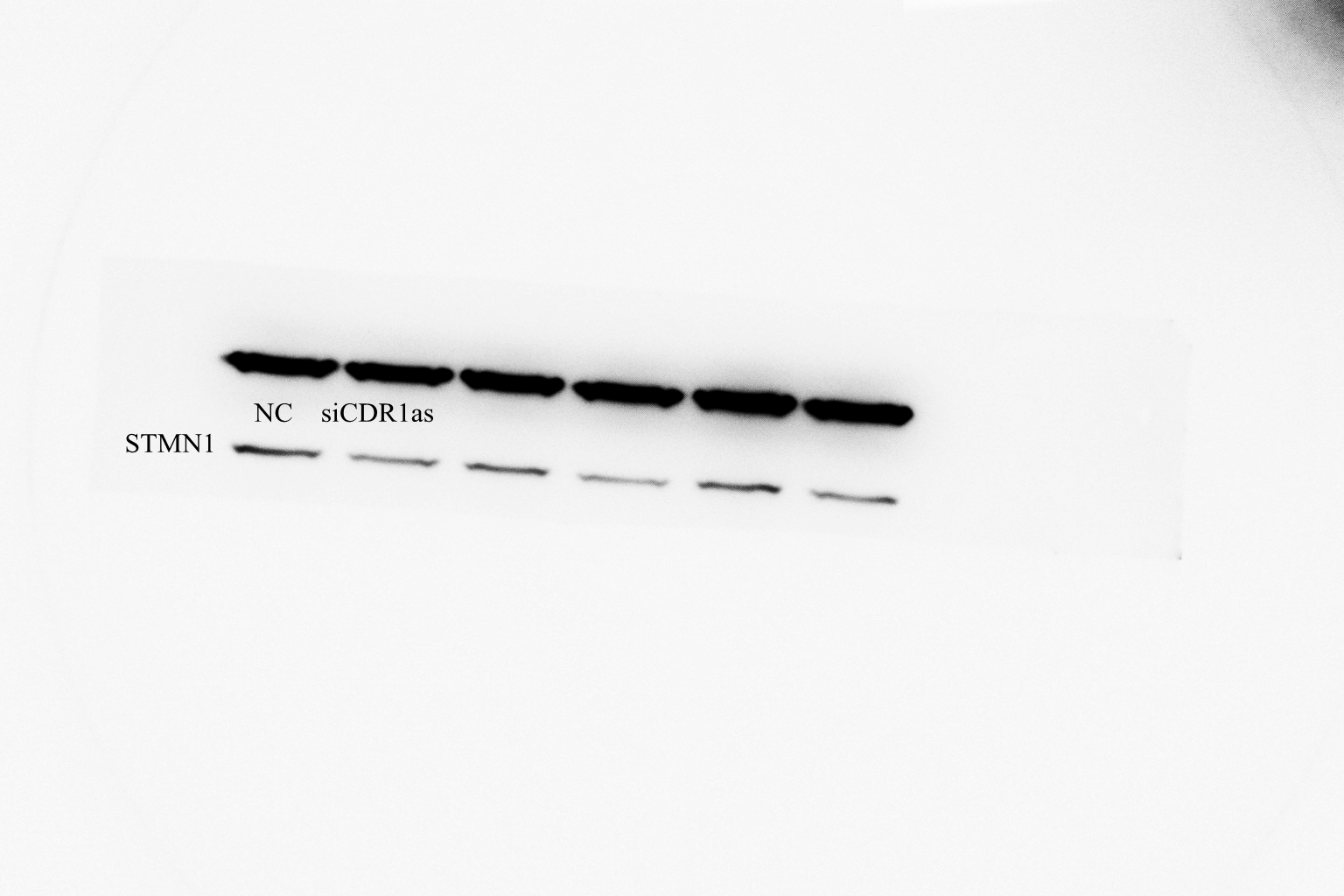


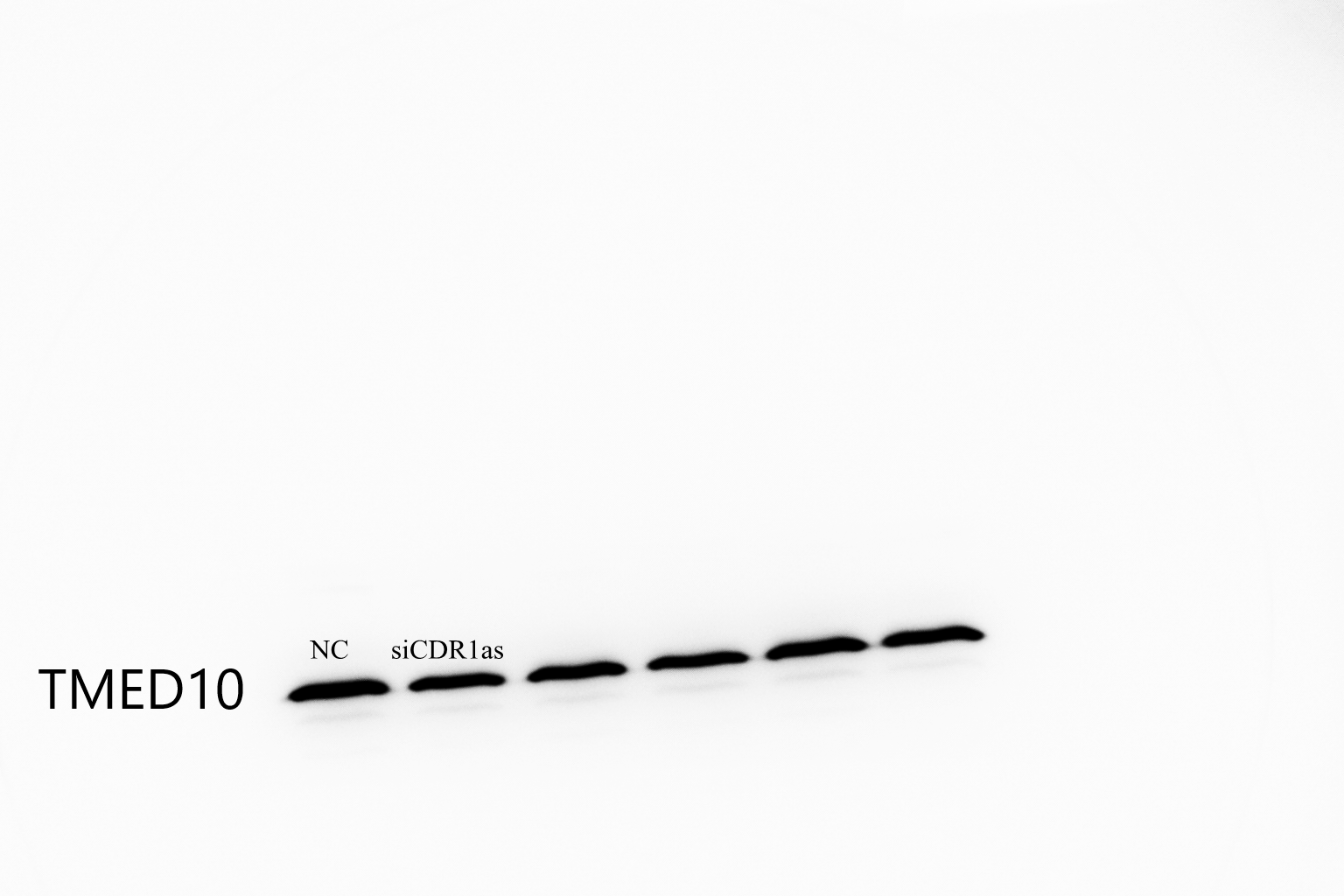


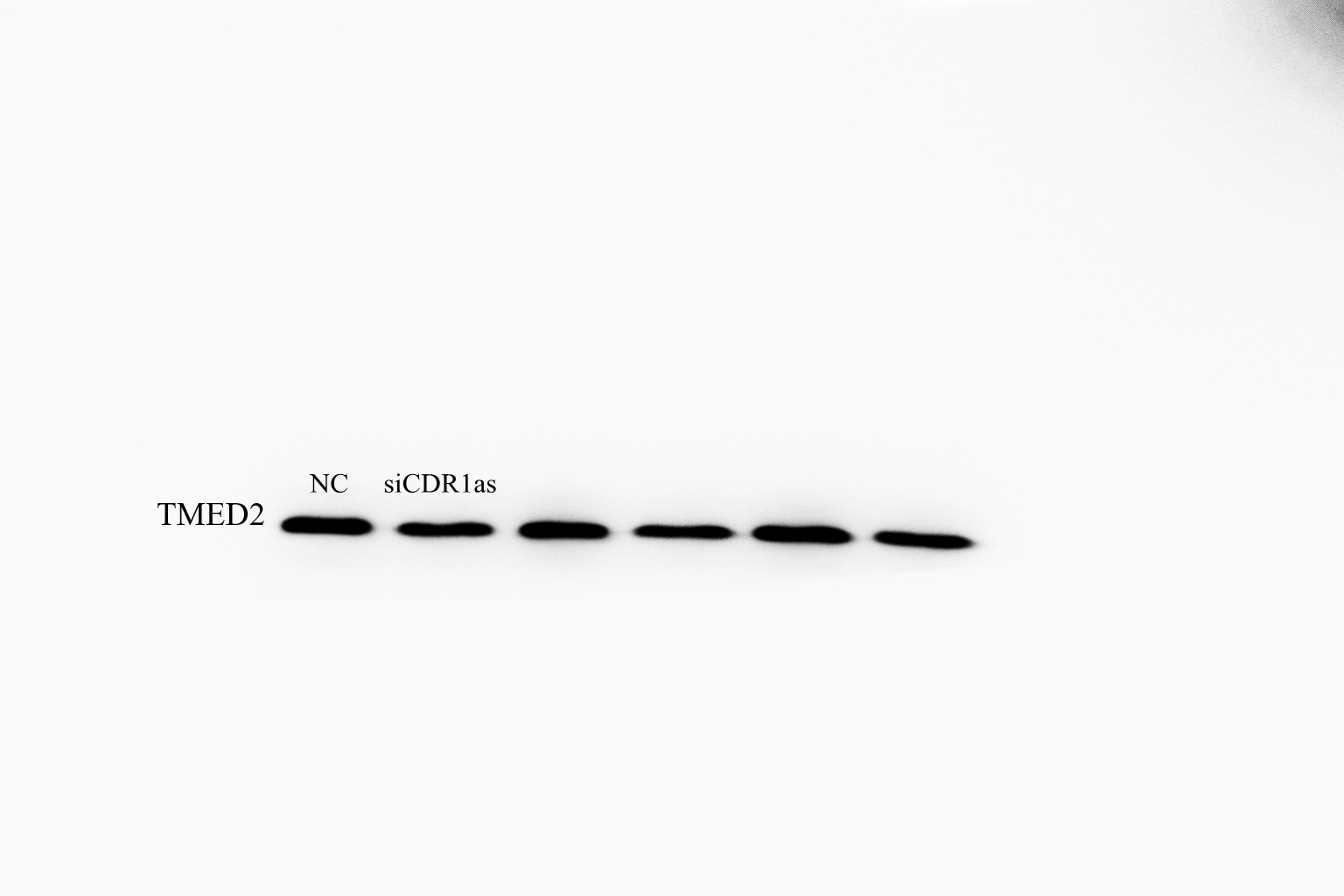


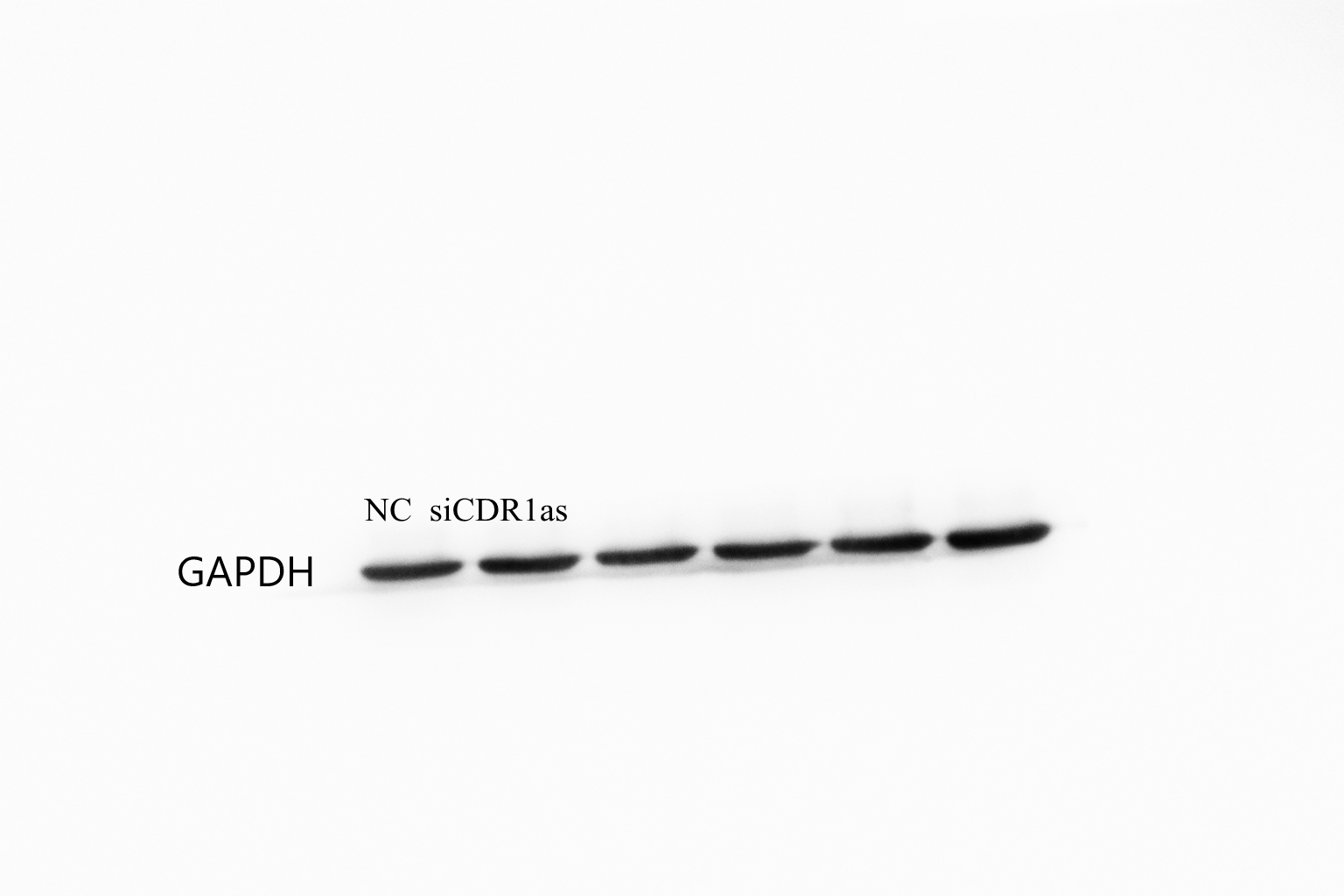


**Figure S5** Western blots for Fig. 4J.


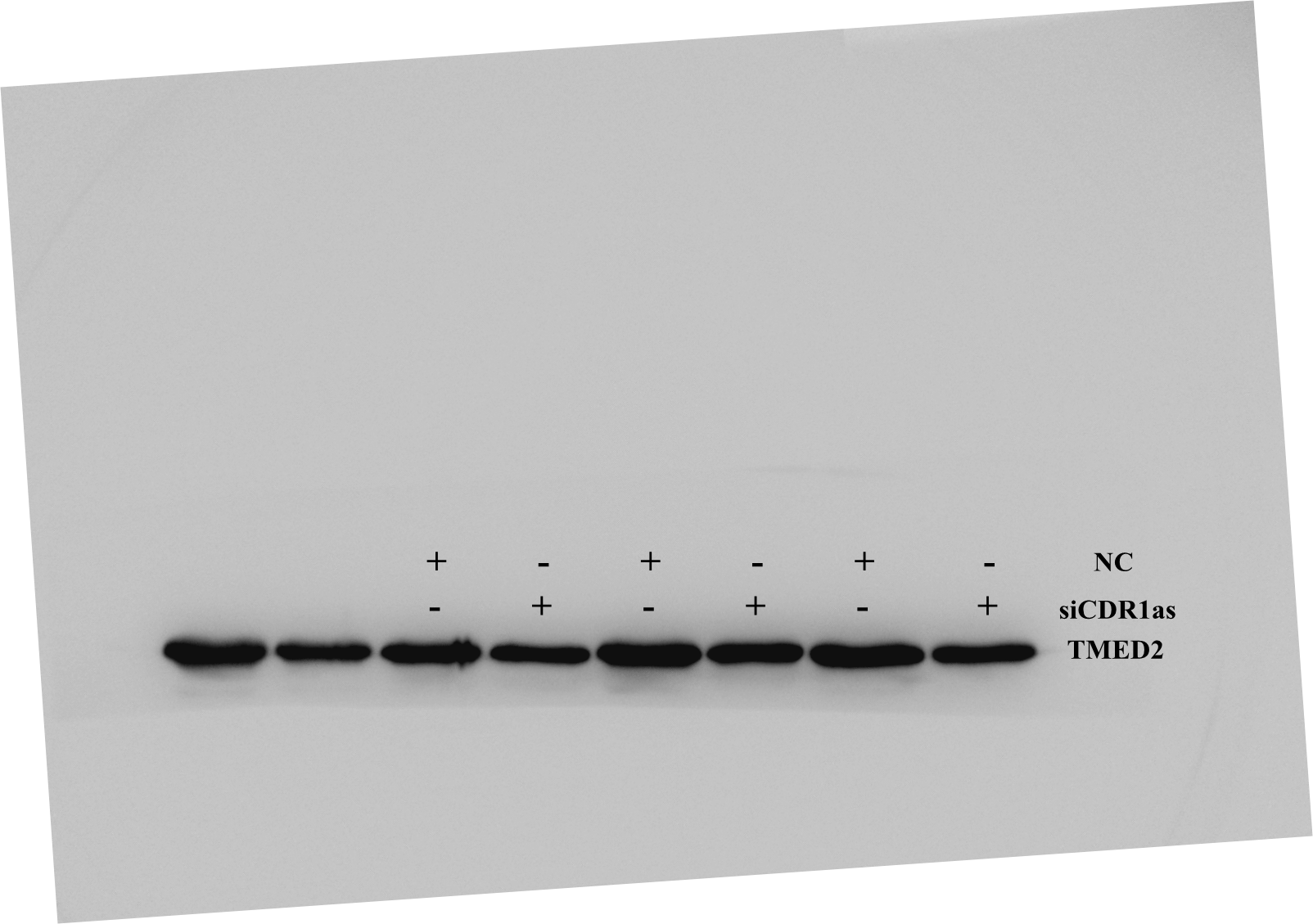


TMED2 sample 1, sample 2, sample 3


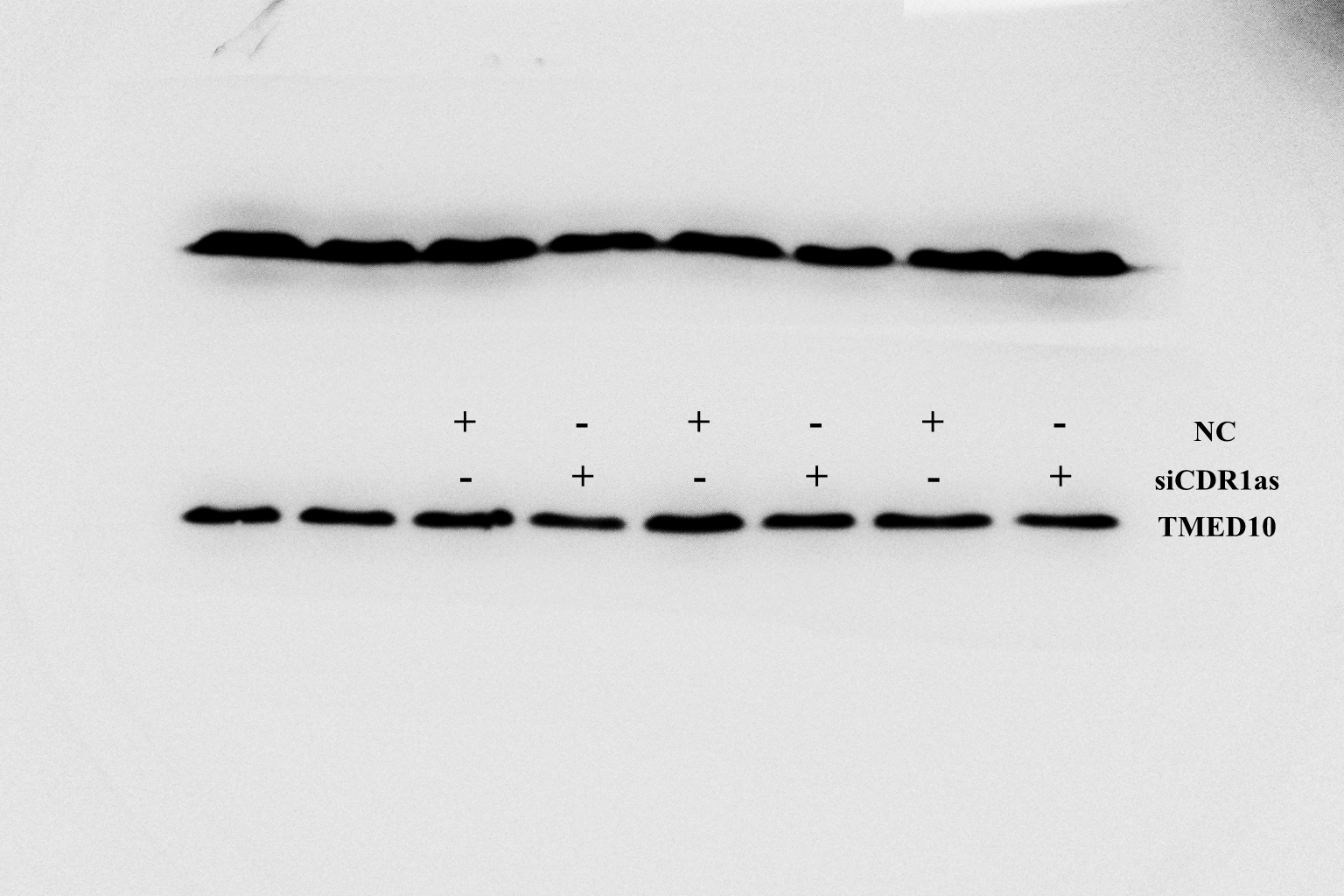


TMED10 sample 1, sample 2, sample 3


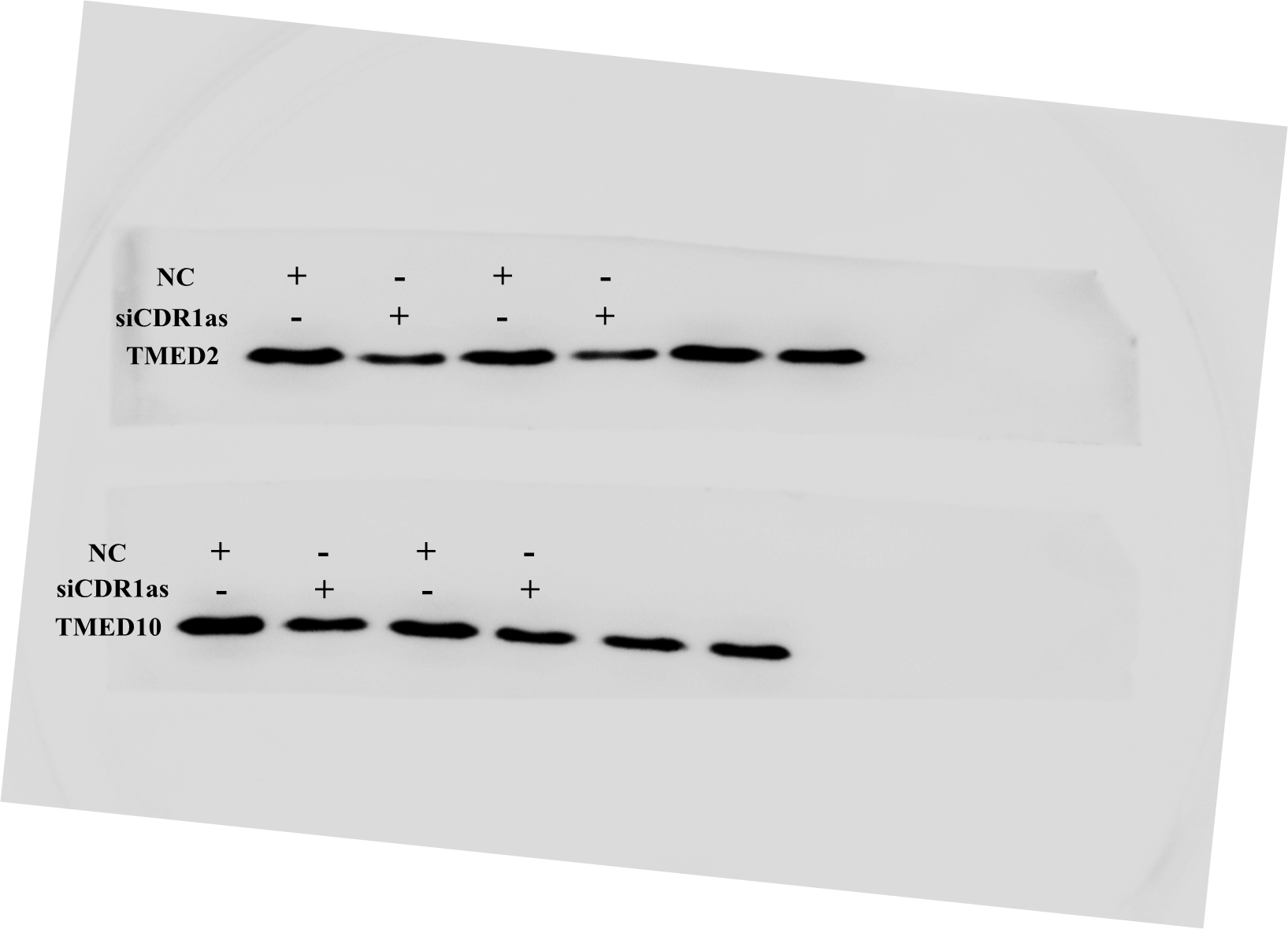


TMED2 TMED10 sample 4, sample 5

**
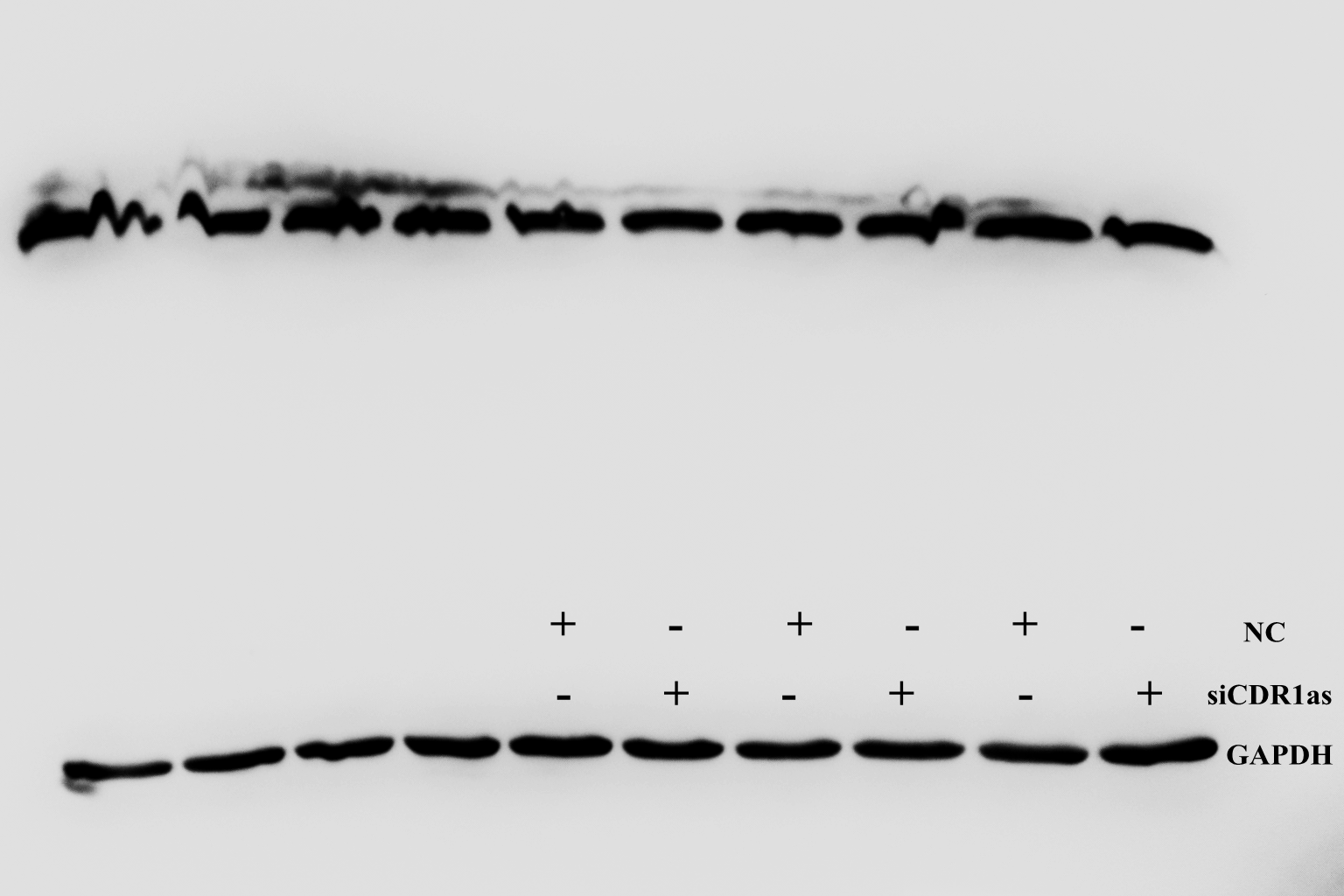
**

GAPDH sample 1, sample 2, sample 3

**
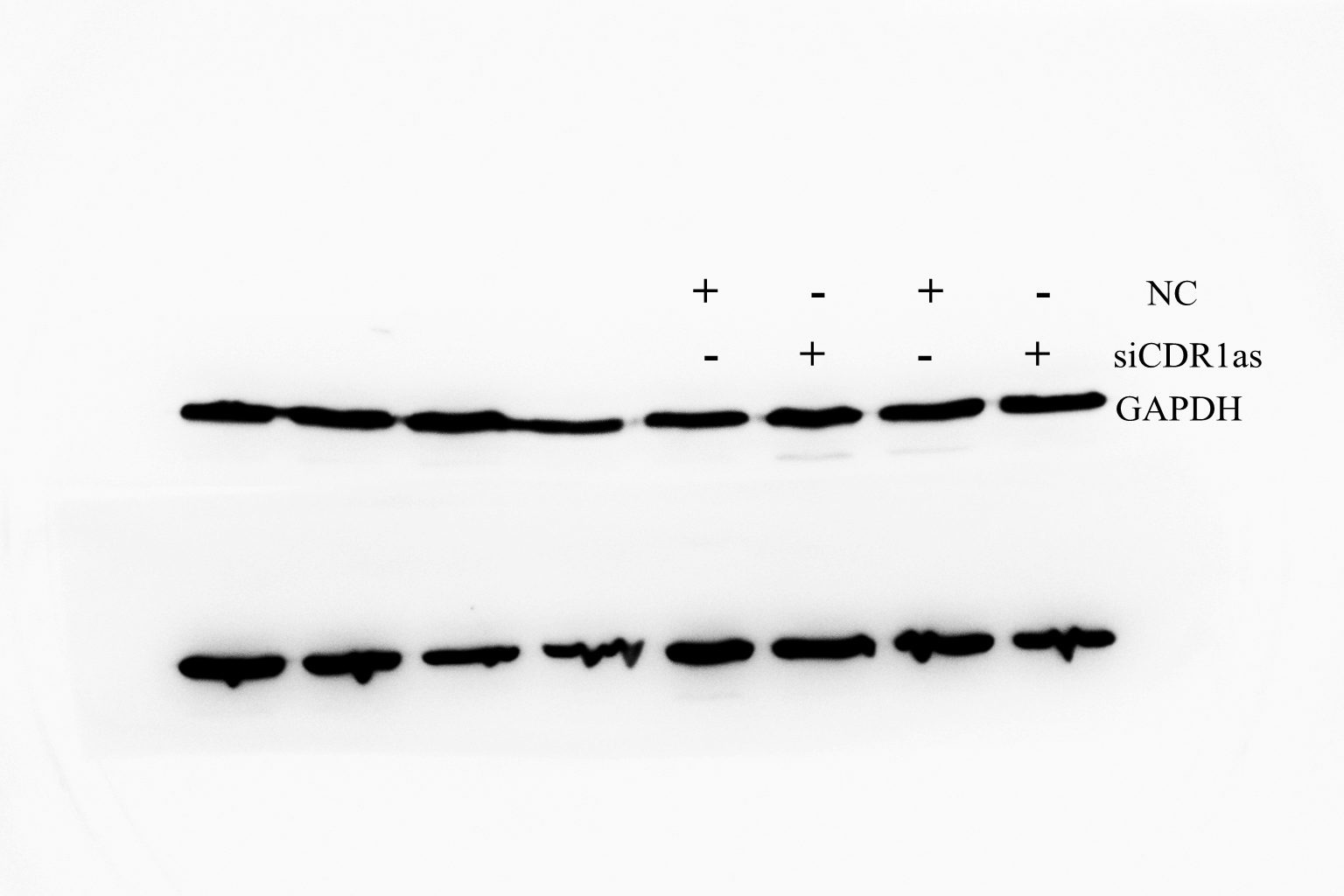
**

GAPDH sample 4, sample 5

**Figure S6** Western blots for Fig. 6D.

**
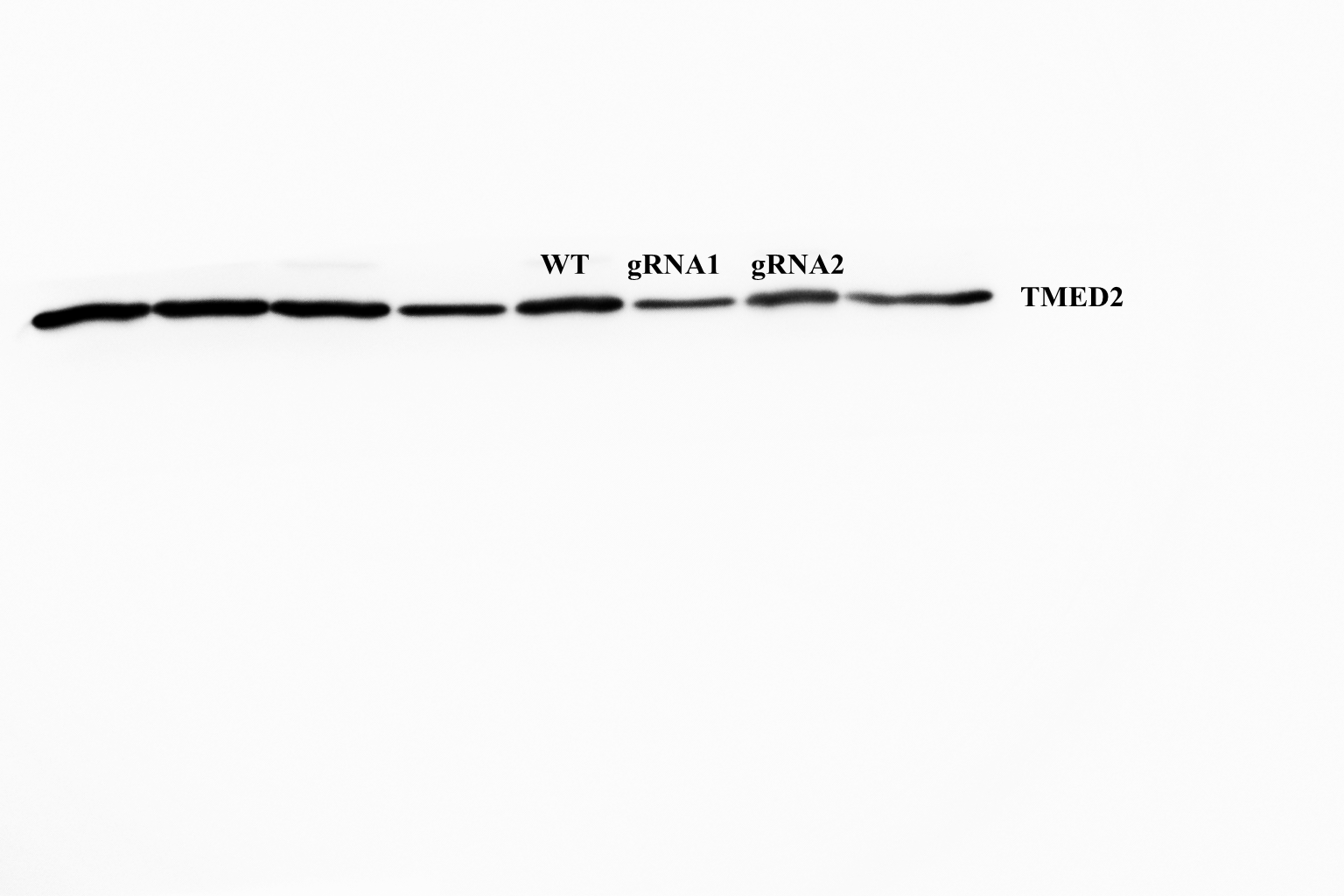
**

**
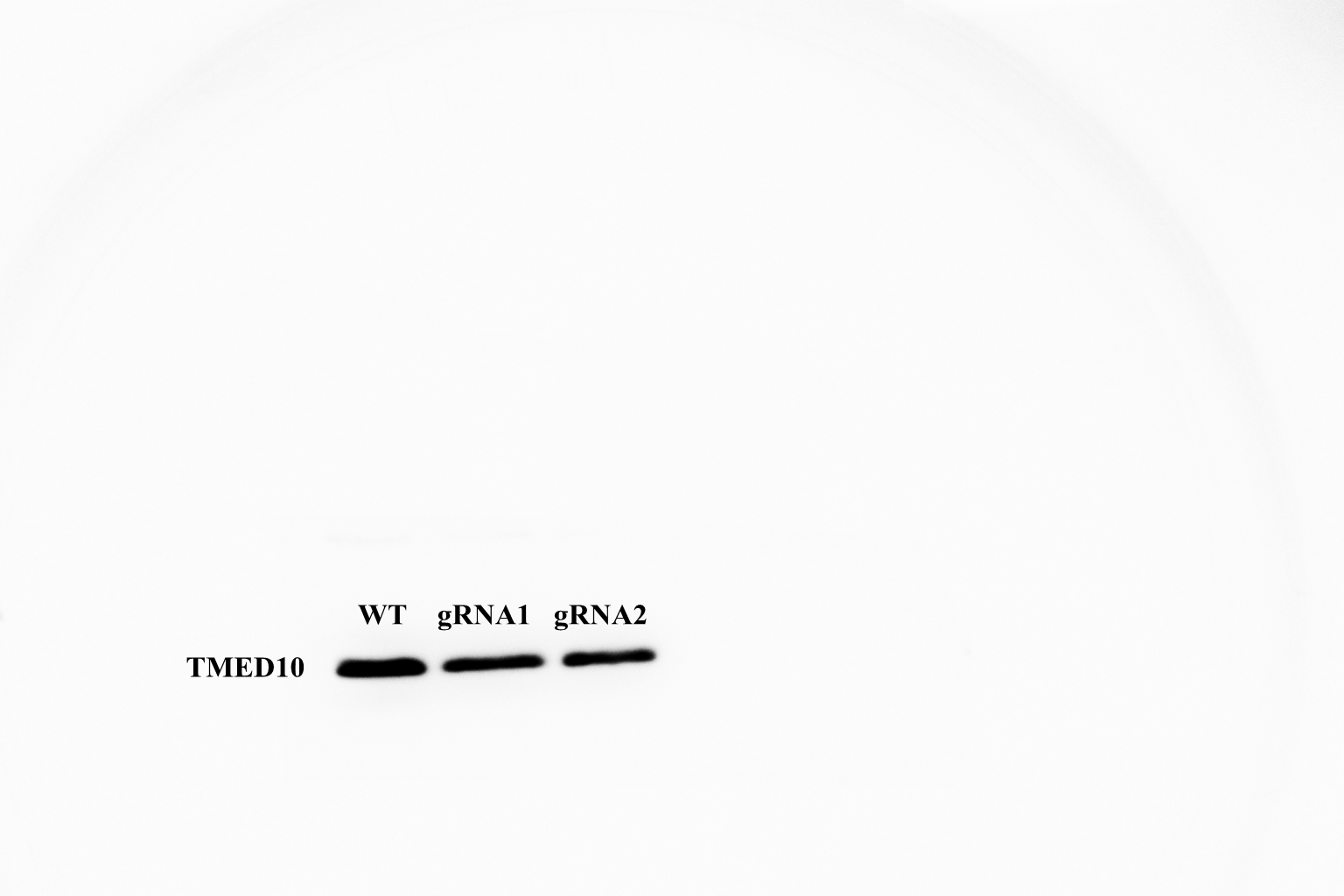
**

**
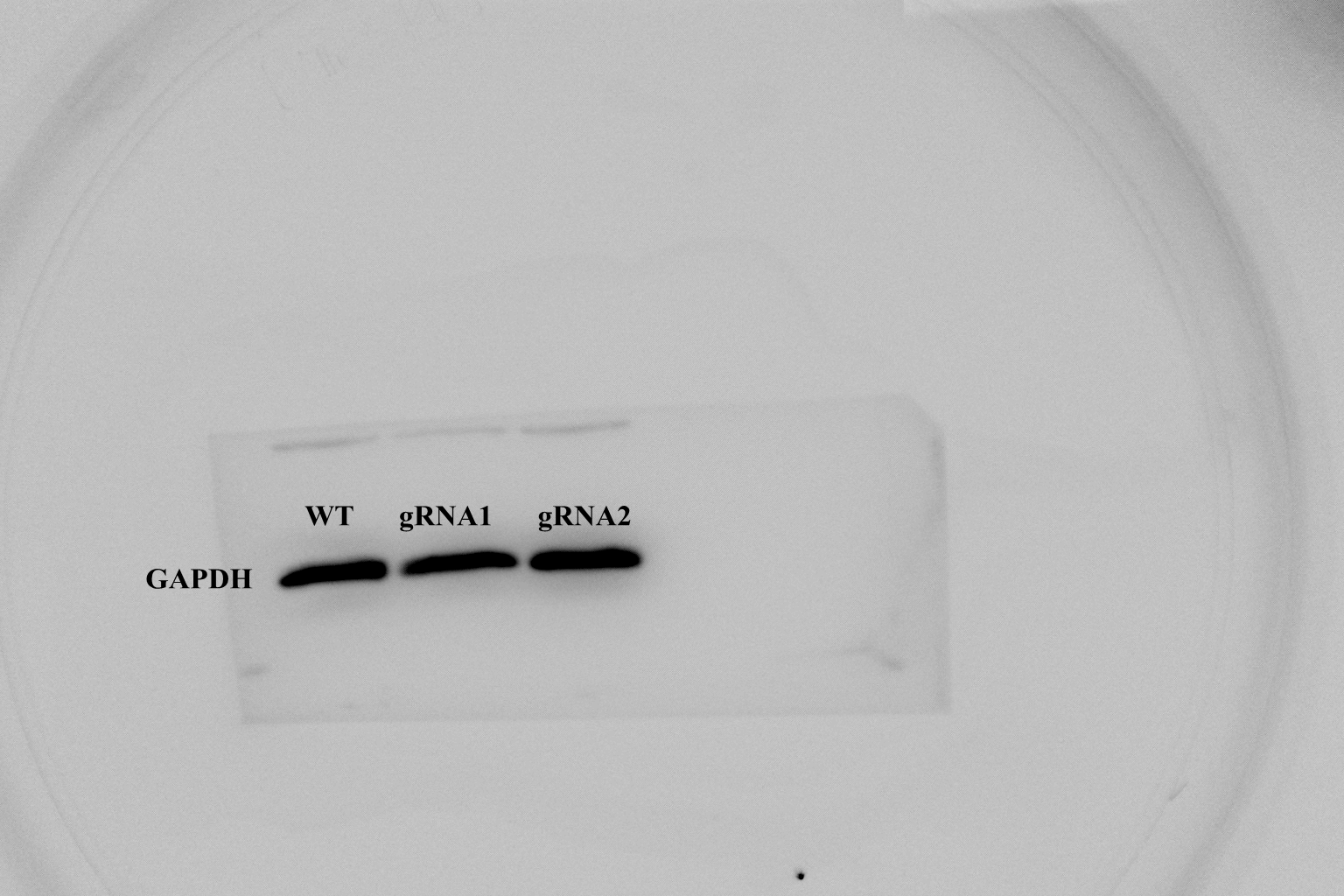
**

**Figure S7** Western blots for Fig. 6H

**
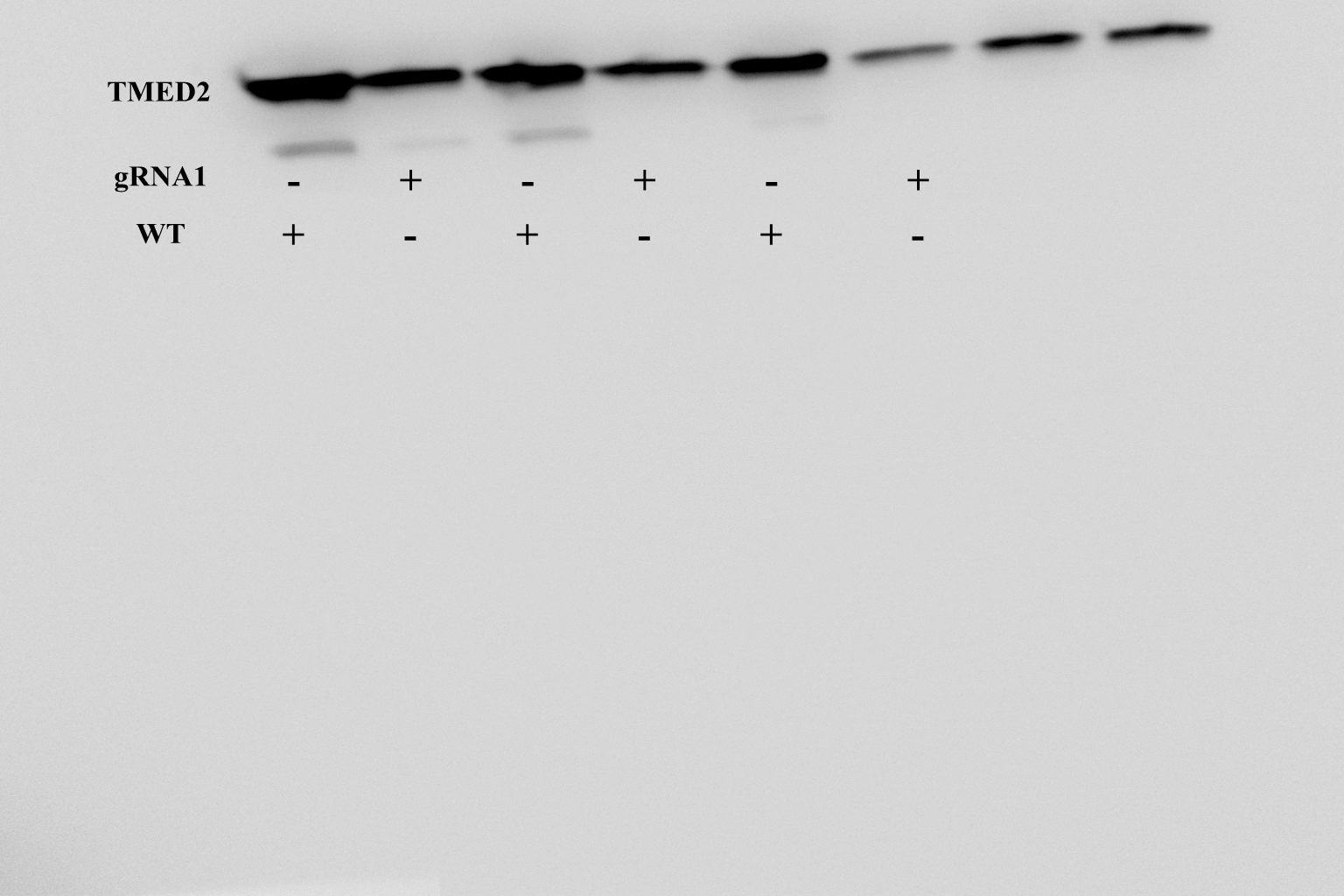
**

TMED2 sample 1, sample 2, sample 3

**
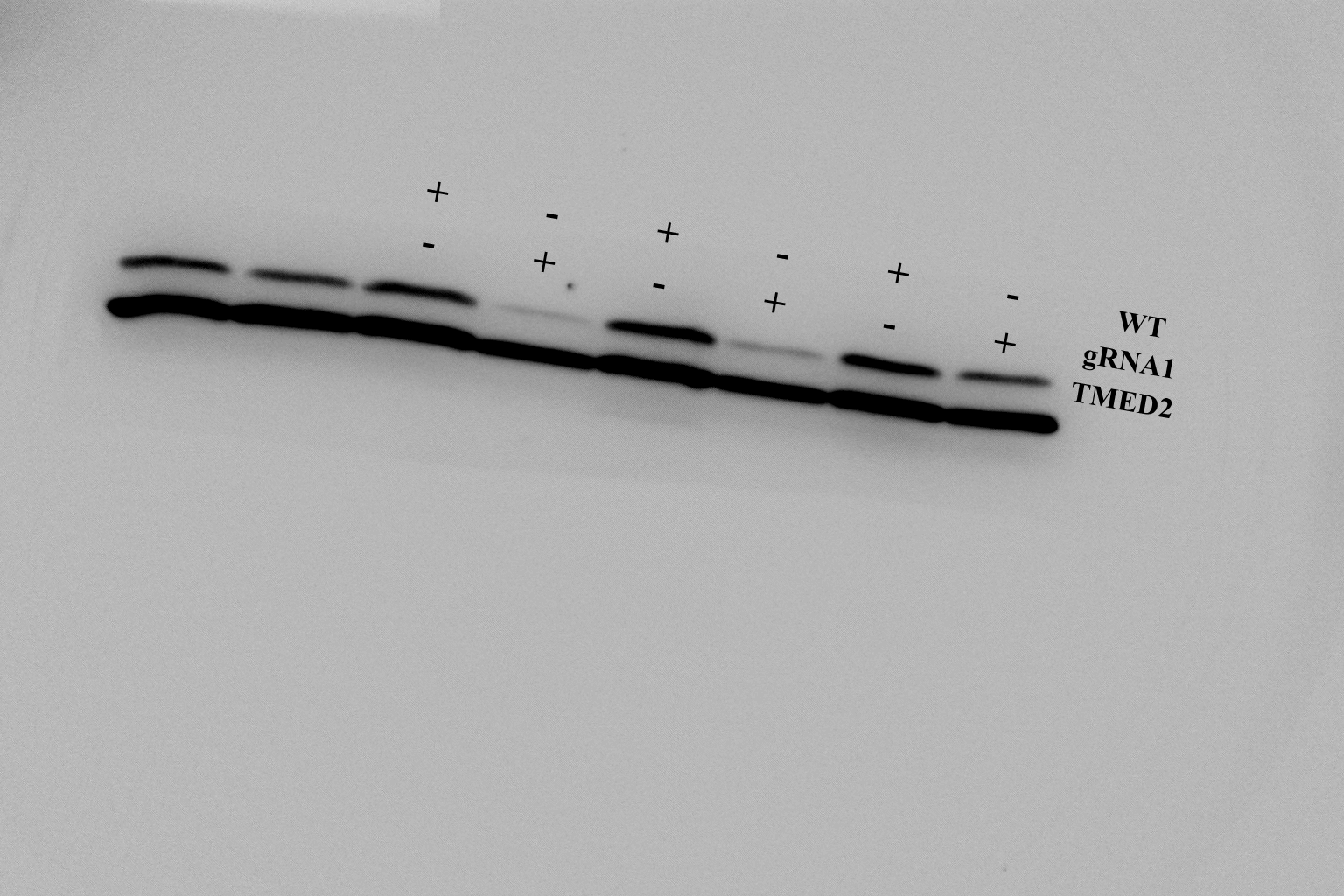
**TMED2 sample 4, sample 5, sample 6


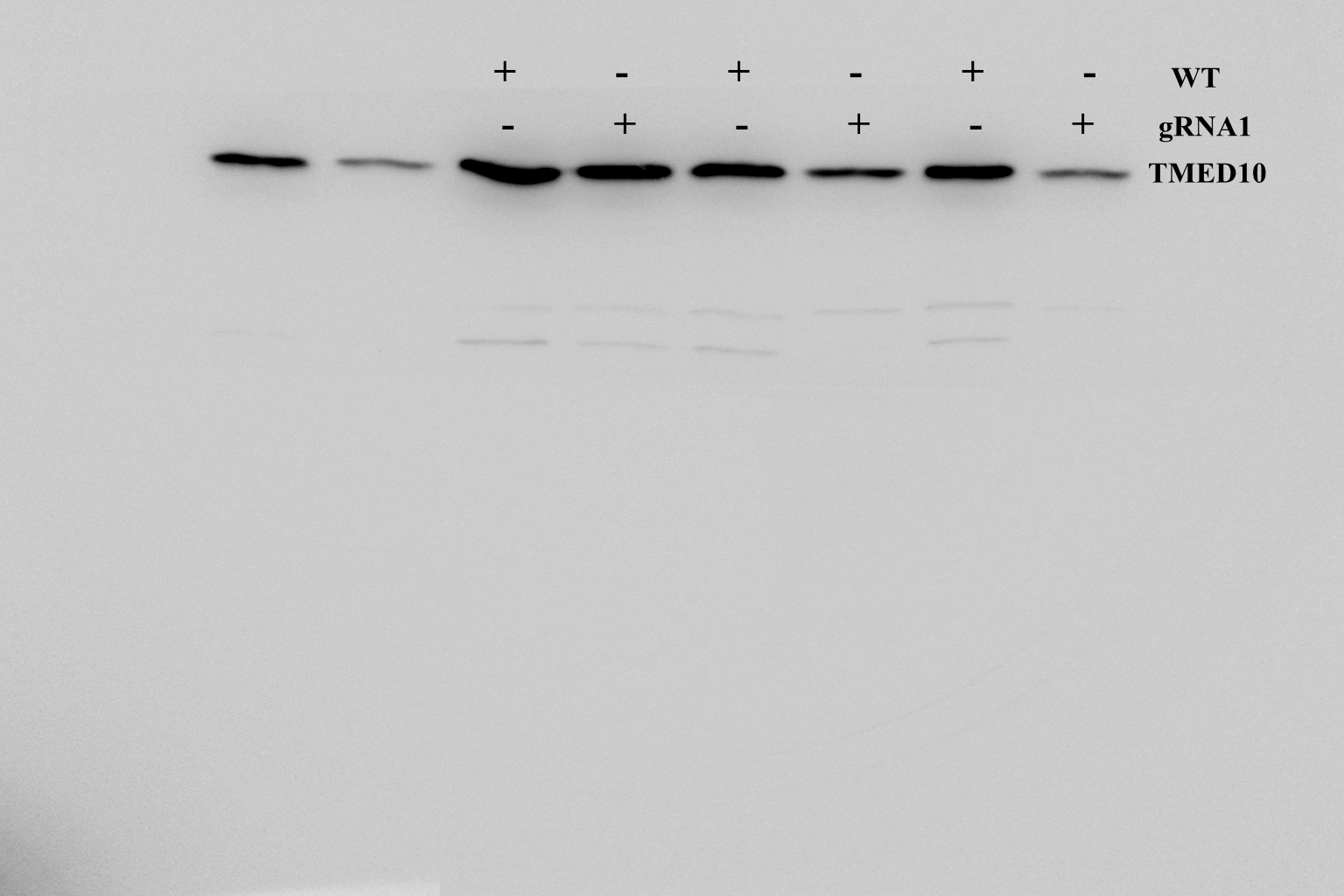


TMED10 sample 1, sample 2, sample 3


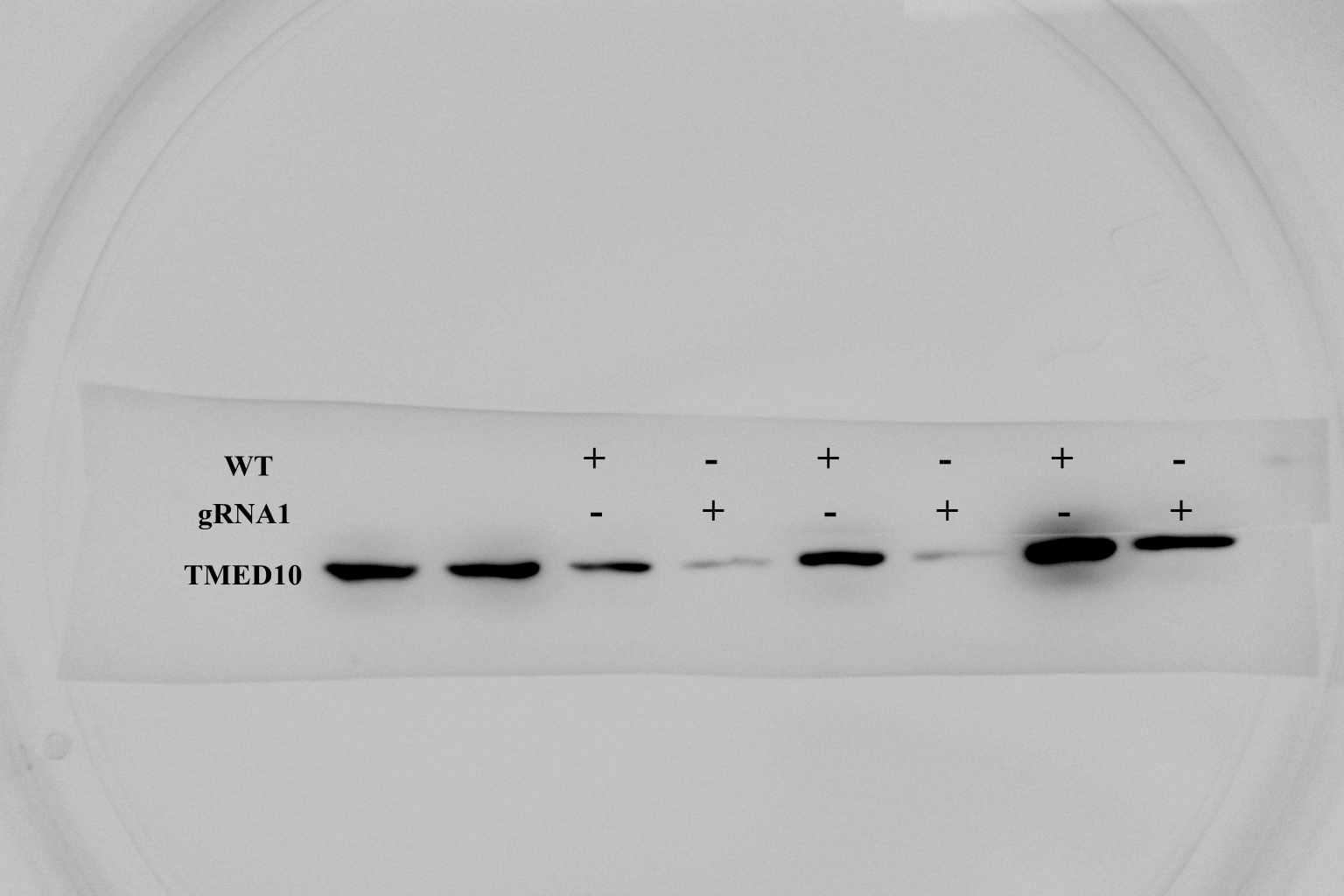


TMED10 sample 4, sample 5, sample 6


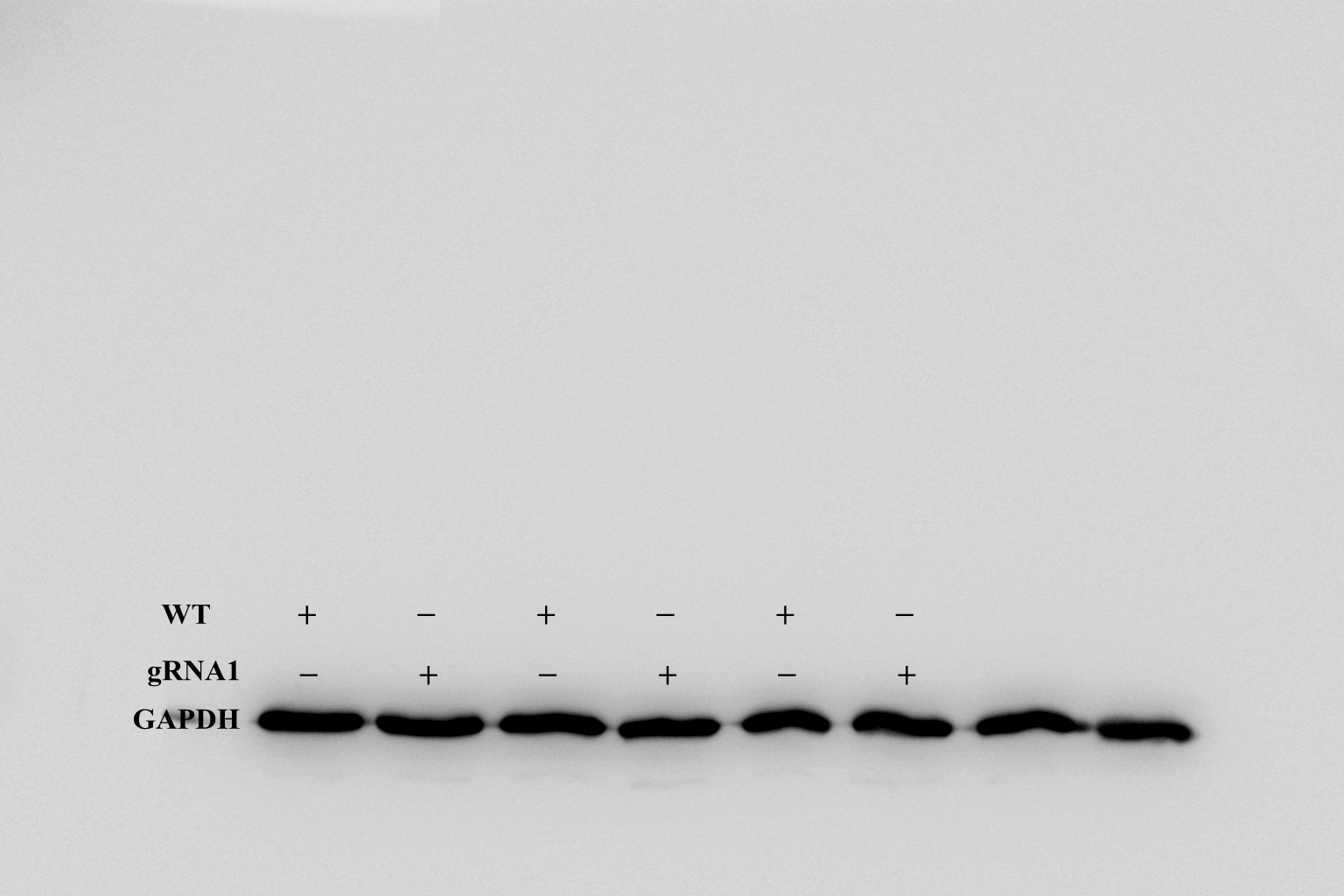


GAPDH sample 1, sample 2, sample 3


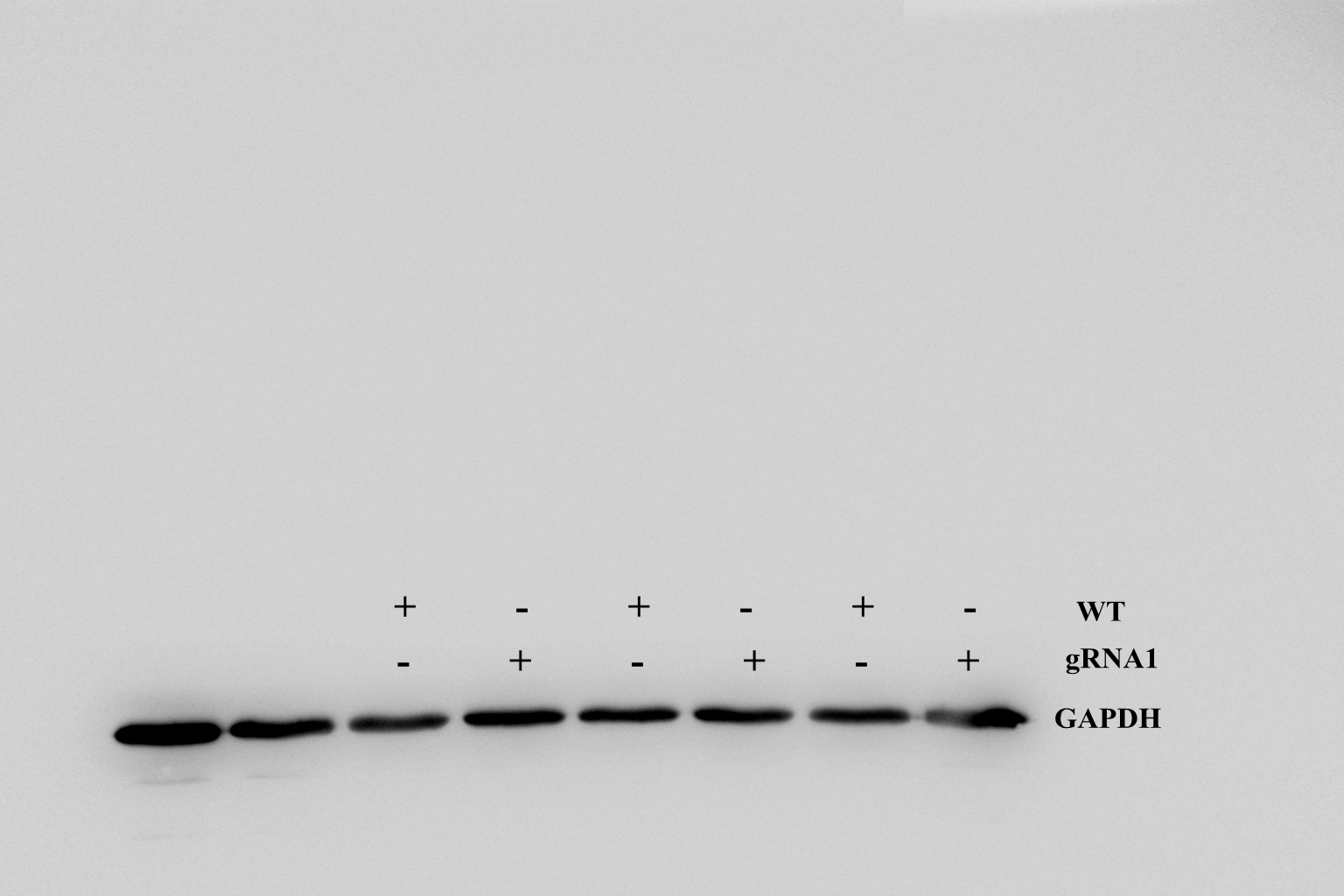


GAPDH sample 4, sample 5, sample 6
