# Supplementary material for: The circular RNA CDR1as regulate cell proliferation via TMED2 and TMED10
Source: BMC Cancer. 2020 Apr 15;20:312. doi: 10.1186/s12885-020-06794-5 (PMC7160961; doi:10.1186/s12885-020-06794-5)
Supplement: Supplementary file 2 — Additional file 2. [file 12885_2020_6794_MOESM2_ESM.pdf]

Table S1 List of oligos

**List of siRNA sequences**		
siNC		5'-UUCUCCGAACGUGUCACGUTT-3'
siCDR1as		5'-GGTCTTCCAGCGACTTCAATT-3’
siTMED2		5'-GGACCAGAUAACAAAGGAATT-3’
siTMED10		5'-UCUUCUACCUGCGACGCUUTT-3’
**Primer sequences for qRT-PCR**		
CDR1as	Forward	5'-ACGTCTCCAGTGTGCTGA-3’
	Reverse	5'-CTTGACACAGGTGCCATC-3’
TMED2	Forward	5'-ATGTATTCCTGTTCGTGCTT-3’
	Reverse	5'-CACATGGATGGAACATACAA-3’
TMED10	Forward	5'-GAGATGCGTGATACCAACGA-3’
	Reverse	5'-TTCTTGGCCTTGAAGAAGCG-3’
GAPDH	Forward	5'-GTCAGCCGCATCTTCTTTTG-3’
	Reverse	5'-GCGCCCAATACGACCAAATC-3’
**Primer sequences for plasmids construction**		
TMED2	Forward	5'-CCCAAGCTTGCCACCATGGTGACGCTTGCTG-3’
	Reverse	5'-TGCTCTAGATTAAACAACTCTCCG-3’
TMED10	Forward	5'-CCCAAGCTTGCCACCATGTCTGGTTTGTC-3’
	Reverse	5'-TGCTCTAGATTACTCAATCAATTTCTTGGC-3’
psiCheck2-TMED2	Forward	5'-CCTCGAGGGCATGTAAGAAATAAGCA-3’
	Reverse	5'-TGCGGCCGCACCCAAGGTGGAAGTATG-3’

psiCheck2-TMED2-mut	Forward	5'-TCTTTTGGCTGTTTGTTTTTGGCACATGTG-3’
	Reverse	5'-CCAAAAAACAAACAGCCAAAAGAAACAAAAAC-3’
psiCheck2-TMED10	Forward	5'-CCTCGAGTCCAATGGCTAATGATGT-3’
	Reverse	5'-TGCGGCCGCATCTGTAGCTCCCGGTAA-3’
psiCheck2-TMED10-mut	Forward	5'-CGGTGAAATCTGTTGATGTCTTTTGTCTATTTTC-3’
	Reverse	5'-AAGACATCAACAGATTTCACCGAAGAGGATACA-3’
CDR1as (has) FISH probe		
5’Alex Fluor 647-TACATGGATTTGTTGGAAGACATGGATTTTCTGGAAGACATGGATTTTCT-3’		
gRNA used in the CRISPR experiment		
gRNA1-F		CACCGATTGGAAGACTTGAAGTCGC
gRNA1-R		AAACGCGACTTCAAGTCTTCCAATC
gRNA2-F		CACCGTTGTTGGAAGACCTTGACAC
gRNA2-R		AAACGTGTCAAGGTCTTCCAACAAC
