# Supplementary material for: The circular RNA CDR1as regulate cell proliferation via TMED2 and TMED10
Source: BMC Cancer. 2020 Apr 15;20:312. doi: 10.1186/s12885-020-06794-5 (PMC7160961; doi:10.1186/s12885-020-06794-5)
Supplement: Supplementary file 3 — Additional file 3. [file 12885_2020_6794_MOESM3_ESM.pdf]

Table S2 Complete list of the quantitated proteins in iTRAQ experments

Accession #	Gene name	Protein	Unused	Total	% Cov	Peptides(95%)	Ratio (siCDR1as/NC)
sp|Q6ZS30|NBEL1_HUMAN	NBEAL1	Neurobeachin-like protein 1	2.05	4.97	11.3	5	0.163
sp|Q96BR1|SGK3_HUMAN	SGK3	Serine/threonine-protein kinase Sgk3	4.03	4.32	21.2	3	0.218
sp|P52926|HMGA2_HUMAN	HMGA2	High mobility group protein HMGI-C	10.02	10.31	60.6	6	0.253
sp|P60059|SC61G_HUMAN	SEC61G	Protein transport protein Sec61 subunit gamma	4.01	4.01	48.5	4	0.264
sp|P68431|H31_HUMAN	HIST1H3A	Histone H3.1	35.62	37.49	80.9	139	0.266
sp|Q08945|SSRP1_HUMAN	SSRP1	FACT complex subunit SSRP1	53.38	53.59	50.6	35	0.270
sp|P69905|HBA_HUMAN	HBA1	Hemoglobin subunit alpha	4.96	5.1	43.7	6	0.297
sp|Q53R41|FAKD1_HUMAN	FASTKD1	FAST kinase domain-containing protein 1	10.91	13.75	24.1	10	0.300
sp|Q9Y5B9|SP16H_HUMAN	SUPT16H	FACT complex subunit SPT16	114.46	114.77	68.4	86	0.317
sp|O75367|H2AY_HUMAN	H2AFY	Core histone macro-H2A.1	37.36	37.74	58.6	34	0.320
sp|O43903|GAS2_HUMAN	GAS2	Growth arrest-specific protein 2	8.08	9.71	37.7	6	0.330
sp|Q71DI3|H32_HUMAN	HIST2H3A	Histone H3.2	2.22	32.94	80.9	146	0.342
sp|P51991|ROA3_HUMAN	HNRNPA3	Heterogeneous nuclear ribonucleoprotein A3	49.27	60.17	63	78	0.345
sp|P54727|RD23B_HUMAN	RAD23B	UV excision repair protein RAD23 homolog B	36.01	36.11	68.2	34	0.346
sp|Q15043|S39AE_HUMAN	SLC39A14	Zinc transporter ZIP14	6.14	6.23	21.3	6	0.352
sp|O95994|AGR2_HUMAN	AGR2	Anterior gradient protein 2 homolog	3.8	3.89	24.6	2	0.355
sp|O95747|OXSR1_HUMAN	OXSR1	Serine/threonine-protein kinase R1	26.34	28.12	52	16	0.359
sp|P62805|H4_HUMAN	HIST1H4A	Histone H4	33.01	33.08	87.4	140	0.360
sp|Q96M27|PRRC1_HUMAN	PRRC1	Protein PRRC1	15.17	15.33	33.5	10	0.360
sp|P51149|RAB7A_HUMAN	RAB7A	Ras-related protein Rab-7a	33.21	33.47	92.3	34	0.362
sp|O43493|TGON2_HUMAN	TGOLN2	Trans-Golgi network integral membrane protein 2	7.46	7.54	22.9	5	0.365
sp|P11388|TOP2A_HUMAN	TOP2A	DNA topoisomerase 2-alpha	133.68	136.24	60.3	102	0.367
sp|Q16695|H31T_HUMAN	HIST3H3	Histone H3.1t	1.89	23.26	78.7	26	0.368
sp|O43306|ADCY6_HUMAN	ADCY6	Adenylate cyclase type 6	2.04	2.09	12.7	3	0.374
sp|Q9UNW9|NOVA2_HUMAN	NOVA2	RNA-binding protein Nova-2	3.51	3.76	13.8	4	0.384
sp|P17096|HMGA1_HUMAN	HMGA1	High mobility group protein HMG-I/HMG-Y	10.55	11.1	49.5	6	0.398
sp|P84243|H33_HUMAN	H3F3A	Histone H3.3	7.6	31.53	78.7	78	0.398
sp|Q8TE76|MORC4_HUMAN	MORC4	MORC family CW-type zinc finger protein 4	2.02	4.71	16.1	3	0.402
sp|Q9BXS9|S26A6_HUMAN	SLC26A6	Solute carrier family 26 member 6	4.03	4.09	16.3	3	0.402
sp|P43307|SSRA_HUMAN	SSR1	Translocon-associated protein subunit alpha	8.01	8.01	29.4	14	0.406
sp|Q8IVP5|FUND1_HUMAN	FUNDC1	FUN14 domain-containing protein 1	2	2	18.7	2	0.415
sp|Q6NXT1|ANR54_HUMAN	ANKRD54	Ankyrin repeat domain-containing protein 54	1.9	2.09	13	4	0.416
sp|P62995|TRA2B_HUMAN	TRA2B	Transformer-2 protein homolog beta	12.07	14.62	41.3	11	0.422
sp|P09210|GSTA2_HUMAN	GSTA2	Glutathione S-transferase A2	6.81	7.95	44.1	7	0.424
sp|Q15166|PON3_HUMAN	PON3	Serum paraoxonase/lactonase 3	5.53	5.7	47.5	5	0.424
sp|P00450|CERU_HUMAN	CP	Ceruloplasmin	4.01	4.12	11	2	0.430
sp|Q53GQ0|DHB12_HUMAN	HSD17B12	Very-long-chain 3-oxoacyl-CoA reductase	24.86	24.98	69.6	19	0.434
sp|P16949|STMN1_HUMAN	STMN1	Stathmin	15.35	15.53	76.5	16	0.437
sp|P19971|TYPH_HUMAN	TYMP	Thymidine phosphorylase	6.55	6.6	20.1	3	0.439
sp|Q9H832|UBE2Z_HUMAN	UBE2Z	Ubiquitin-conjugating enzyme E2 Z	10.12	10.3	31.1	7	0.443
sp|O76021|RL1D1_HUMAN	RSL1D1	Ribosomal L1 domain-containing protein 1	64.21	65.26	72.6	52	0.443
sp|Q9BZE2|PUS3_HUMAN	PUS3	tRNA pseudouridine(38/39) synthase	7.61	7.74	23.3	5	0.446
sp|O00422|SAP18_HUMAN	SAP18	Histone deacetylase complex subunit SAP18	21.18	21.41	73.9	12	0.451
sp|Q96EB1|ELP4_HUMAN	ELP4	Elongator complex protein 4	4	4.01	17	2	0.455
sp|Q15375|EPHA7_HUMAN	EPHA7	Ephrin type-A receptor 7	1.83	6.09	13.4	3	0.456
sp|Q92982|NINJ1_HUMAN	NINJ1	Ninjurin-1	2.02	2.02	17.1	2	0.461

sp|Q2PPJ7|RGPA2_HUMAN	RALGAPA2	Ral GTPase-activating protein subunit alpha-2	2.2	2.37	14.6	2	0.462
sp|Q9H2V7|SPNS1_HUMAN	SPNS1	Protein spinster homolog 1	10	10	19.3	5	0.463
sp|Q13185|CBX3_HUMAN	CBX3	Chromobox protein homolog 3	22.95	23.08	68.9	27	0.464
sp|Q9H501|ESF1_HUMAN	ESF1	ESF1 homolog	41.33	42.55	42.9	25	0.466
sp|P61026|RAB10_HUMAN	RAB10	Ras-related protein Rab-10	17.24	28.34	77	20	0.467
sp|Q99805|TM9S2_HUMAN	TM9SF2	Transmembrane 9 superfamily member 2	28.43	29.19	36.5	27	0.469
sp|P05114|HMGN1_HUMAN	HMGN1	Non-histone chromosomal protein HMG-14	12.87	13	69	10	0.473
sp|P30040|ERP29_HUMAN	ERP29	Endoplasmic reticulum resident protein 29	27.12	29.79	82	33	0.474
sp|P12956|XRCC6_HUMAN	XRCC6	X-ray repair cross-complementing protein 6	107.37	108.98	75	137	0.476
sp|P53999|TCP4_HUMAN	SUB1	Activated RNA polymerase II transcriptional coactivator p15	17.98	18.53	55.1	15	0.477
sp|Q9UKM9|RALY_HUMAN	RALY	RNA-binding protein Raly	34.52	36.45	77.8	30	0.478
sp|Q9H019|MFR1L_HUMAN	MTFR1L	Mitochondrial fission regulator 1-like	6.04	6.44	27.1	5	0.481
sp|P13010|XRCC5_HUMAN	XRCC5	X-ray repair cross-complementing protein 5	102.39	102.61	85.8	127	0.482
sp|P05204|HMGN2_HUMAN	HMGN2	Non-histone chromosomal protein HMG-17	11.18	11.44	64.4	9	0.484
sp|Q9UGJ1|GCP4_HUMAN	TUBGCP4	Gamma-tubulin complex component 4	7.66	7.78	24.4	5	0.485
sp|Q9P2E3|ZNFX1_HUMAN	ZNFX1	NFX1-type zinc finger-containing protein 1	2.95	3.49	12	4	0.486
sp|Q8IZ21|PHAR4_HUMAN	PHACTR4	Phosphatase and actin regulator 4	7.3	8.07	22.7	6	0.487
sp|Q6P4F2|ADXL_HUMAN	FDX1L	Adrenodoxin-like protein, mitochondrial	4.08	4.1	40.4	3	0.487
sp|P11387|TOP1_HUMAN	TOP1	DNA topoisomerase 1	78.81	79	63.5	51	0.488
sp|Q9BPZ7|SIN1_HUMAN	MAPKAP1	Target of rapamycin complex 2 subunit MAPKAP1	2.01	2.45	19.5	3	0.490
sp|Q9BVQ7|SPA5L_HUMAN	SPATA5L1	Spermatogenesis-associated protein 5-like protein 1	13.69	13.96	33.6	10	0.490
sp|Q8WUA2|PPIL4_HUMAN	PPIL4	Peptidyl-prolyl cis-trans isomerase-like 4	26.85	26.99	44.5	15	0.491
sp|P45973|CBX5_HUMAN	CBX5	Chromobox protein homolog 5	13.84	15.96	51.8	11	0.491
sp|Q15629|TRAM1_HUMAN	TRAM1	Translocating chain-associated membrane protein 1	7.8	8.01	22.5	6	0.494
sp|P06753|TPM3_HUMAN	TPM3	Tropomyosin alpha-3 chain	13.12	32.62	67	23	0.494
sp|P04004|VTNC_HUMAN	VTN	Vitronectin	2.08	2.09	21.8	5	0.495
sp|Q15363|TMED2_HUMAN	TMED2	Transmembrane emp24 domain-containing protein 2	12.11	12.32	50.3	13	0.496
sp|Q9BRJ2|RM45_HUMAN	MRPL45	39S ribosomal protein L45, mitochondrial	23.63	23.77	58.2	14	0.496
sp|Q9H9H4|VP37B_HUMAN	VPS37B	Vacuolar protein sorting-associated protein 37B	8.01	8.03	38.3	4	0.497
sp|P58876|H2B1D_HUMAN	HIST1H2BD	Histone H2B type 1-D	31.58	32.94	88.1	154	0.505
sp|Q96GG9|DCNL1_HUMAN	DCUN1D1	DCN1-like protein 1	8.29	8.39	41.3	5	0.506
sp|P50552|VASP_HUMAN	VASP	Vasodilator-stimulated phosphoprotein	28.49	28.57	55	20	0.506
sp|Q5JU69|TOR2A_HUMAN	TOR2A	Torsin-2A	4	4.88	25.6	3	0.507
sp|P16104|H2AX_HUMAN	H2AFX	Histone H2AX	28.86	28.98	53.9	91	0.508
sp|O60493|SNX3_HUMAN	SNX3	Sorting nexin-3	7.84	11.7	67.3	7	0.508
sp|P02655|APOC2_HUMAN	APOC2	Apolipoprotein C-II	5.17	6.57	47.5	4	0.508
sp|O00566|MPP10_HUMAN	MPHOSPH10	U3 small nucleolar ribonucleoprotein protein MPP10	28.88	30.39	40.8	20	0.508
sp|Q9UMY1|NOL7_HUMAN	NOL7	Nucleolar protein 7	11.47	12.91	40.5	10	0.510
sp|Q9H7B2|RPF2_HUMAN	RPF2	Ribosome production factor 2 homolog	15.27	19.95	62.4	13	0.512
sp|Q9BQE5|APOL2_HUMAN	APOL2	Apolipoprotein L2	10.85	14.02	44.2	12	0.514
sp|Q9UN36|NDRG2_HUMAN	NDRG2	Protein NDRG2	7.4	7.55	23.5	5	0.515
sp|Q9H583|HEAT1_HUMAN	HEATR1	HEAT repeat-containing protein 1	100.56	104.79	44.5	65	0.515
sp|Q96T23|RSF1_HUMAN	RSF1	Remodeling and spacing factor 1	26.05	28.94	27	16	0.516
sp|Q9Y2X3|NOP58_HUMAN	NOP58	Nucleolar protein 58	45.5	47.7	62.8	40	0.516
sp|Q15061|WDR43_HUMAN	WDR43	WD repeat-containing protein 43	35.03	35.21	54.8	33	0.517
sp|P62910|RL32_HUMAN	RPL32	60S ribosomal protein L32	13.21	13.97	63	16	0.518
sp|Q9P0M6|H2AW_HUMAN	H2AFY2	Core histone macro-H2A.2	23.66	30.65	62.6	25	0.519
sp|Q9BTM1|H2AJ_HUMAN	H2AFJ	Histone H2A.J	4.15	22.97	52.7	83	0.520
sp|Q92833|JARD2_HUMAN	JARID2	Protein Jumonji	2.02	2.26	13.2	3	0.520

sp|Q15417|CNN3_HUMAN	CNN3	Calponin-3	19.61	24.07	61.4	22	0.521
sp|Q8N257|H2B3B_HUMAN	HIST3H2BB	Histone H2B type 3-B	5.61	29.83	84.9	144	0.521
sp|P81605|DCD_HUMAN	DCD	Dermcidin	7.58	8.99	38.2	6	0.522
sp|O95425|SVIL_HUMAN	SVIL	Supervillin	7.03	8.29	13.6	7	0.523
sp|O00560|SDCB1_HUMAN	SDCBP	Syntenin-1	21.61	21.74	78.5	19	0.523
sp|Q9NQZ2|SAS10_HUMAN	UTP3	Something about silencing protein 10	14.18	14.52	34.5	9	0.524
sp|Q9Y241|HIG1A_HUMAN	HIGD1A	HIG1 domain family member 1A, mitochondrial	3.76	4	53.8	4	0.524
sp|Q8TED0|UTP15_HUMAN	UTP15	U3 small nucleolar RNA-associated protein 15 homolog	35	35.16	54.8	22	0.527
sp|O75414|NDK6_HUMAN	NME6	Nucleoside diphosphate kinase 6	3.5	3.65	30.7	5	0.527
sp|Q92598|HS105_HUMAN	HSPH1	Heat shock protein 105 kDa	91.53	100.67	72.7	78	0.527
sp|O43504|LTOR5_HUMAN	LAMTOR5	Ragulator complex protein LAMTOR5	10.01	10.01	96.7	11	0.528
sp|Q9Y2Q5|LTOR2_HUMAN	LAMTOR2	Ragulator complex protein LAMTOR2	12.67	12.79	68	8	0.528
sp|P18583|SON_HUMAN	SON	Protein SON	67.9	68.89	32.1	44	0.529
sp|Q8N584|TT39C_HUMAN	TTC39C	Tetratricopeptide repeat protein 39C	1.63	2.04	17.3	2	0.529
sp|P11908|PRPS2_HUMAN	PRPS2	Ribose-phosphate pyrophosphokinase 2	7.26	20.79	49.1	16	0.530
sp|P49755|TMEDA_HUMAN	TMED10	Transmembrane emp24 domain-containing protein 10	19.04	19.18	54.8	23	0.530
sp|Q13671|RIN1_HUMAN	RIN1	Ras and Rab interactor 1	2.01	4.03	9.2	2	0.532
sp|Q9NXJ5|PGPI_HUMAN	PGPEP1	Pyroglutamyl-peptidase 1	4	4	28.2	2	0.533
sp|Q13206|DDX10_HUMAN	DDX10	Probable ATP-dependent RNA helicase DDX10	47.16	48.86	49.1	28	0.533
sp|Q9BQ13|KCD14_HUMAN	KCTD14	BTB/POZ domain-containing protein KCTD14	2.48	2.52	20.8	2	0.536
sp|Q9NYV6|RRN3_HUMAN	RRN3	RNA polymerase I-specific transcription initiation factor RRN3	6	6.07	26.1	3	0.538
sp|Q969Z3|MARC2_HUMAN	MARC2	Mitochondrial amidoxime reducing component 2	6.44	9.03	34.9	7	0.538
sp|P10768|ESTD_HUMAN	ESD	S-formylglutathione hydrolase	33.54	33.61	85.5	27	0.539
sp|Q9BVA1|TBB2B_HUMAN	TUBB2B	Tubulin beta-2B chain	2	86.77	84.5	314	0.541
sp|Q92609|TBCD5_HUMAN	TBC1D5	TBC1 domain family member 5	11.64	12.19	29.8	9	0.541
sp|Q9NZB8|MOCS1_HUMAN	MOCS1	Molybdenum cofactor biosynthesis protein 1	6.31	6.4	31.8	3	0.545
sp|Q99640|PMYT1_HUMAN	PKMYT1	Membrane-associated tyrosine- and threonine-specific cdc2-inhibitory kinase	7.41	7.49	14.4	4	0.548
sp|Q8WY22|BRI3B_HUMAN	BRI3BP	BRI3-binding protein	4.31	4.57	22.3	4	0.550
sp|Q9H6W3|NO66_HUMAN	NO66	Bifunctional lysine-specific demethylase and histidyl-hydroxylase NO66	4.65	4.79	14	3	0.550
sp|P41223|BUD31_HUMAN	BUD31	Protein BUD31 homolog	9.43	9.55	37.5	6	0.551
sp|P15311|EZRI_HUMAN	EZR	Ezrin	84.13	84.59	68.6	68	0.552
sp|Q9Y5J1|UTP18_HUMAN	UTP18	U3 small nucleolar RNA-associated protein 18 homolog	29.96	30.01	49.6	23	0.553
sp|Q9BRU9|UTP23_HUMAN	UTP23	rRNA-processing protein UTP23 homolog	6.12	6.15	34.5	3	0.553
sp|O60930|RNH1_HUMAN	RNASEH1	Ribonuclease H1	2.66	2.74	31.5	3	0.553
sp|Q02880|TOP2B_HUMAN	TOP2B	DNA topoisomerase 2-beta	82.38	113.4	53	72	0.553
sp|O15116|LSM1_HUMAN	LSM1	U6 snRNA-associated Sm-like protein LSm1	6.4	6.73	72.2	8	0.553
sp|P48509|CD151_HUMAN	CD151	CD151 antigen	3.23	3.49	20.6	2	0.554
sp|Q6IQ21|ZN770_HUMAN	ZNF770	Zinc finger protein 770	2.44	2.96	13.6	4	0.556
sp|Q9NYI0|PSD3_HUMAN	PSD3	PH and SEC7 domain-containing protein 3	2.24	3.07	13.7	3	0.556
sp|Q9BXS5|AP1M1_HUMAN	AP1M1	AP-1 complex subunit mu-1	38.7	40.1	72.3	27	0.559
sp|O75386|TULP3_HUMAN	TULP3	Tubby-related protein 3	2	2.8	7.5	2	0.559
sp|O75531|BAF_HUMAN	BANF1	Barrier-to-autointegration factor	11.44	11.65	84.3	7	0.563
sp|P62424|RL7A_HUMAN	RPL7A	60S ribosomal protein L7a	49.75	51.95	70.7	53	0.564
sp|P00338|LDHA_HUMAN	LDHA	L-lactate dehydrogenase A chain	76.86	76.93	94	109	0.565
sp|O14646|CHD1_HUMAN	CHD1	Chromodomain-helicase-DNA-binding protein 1	24.2	24.95	27.3	17	0.565
sp|P38571|LICH_HUMAN	LIPA	Lysosomal acid lipase/cholesteryl ester hydrolase	4.01	4.01	13	2	0.567
sp|P13051|UNG_HUMAN	UNG	Uracil-DNA glycosylase	8.18	9.63	29.7	5	0.568
sp|P56537|IF6_HUMAN	EIF6	Eukaryotic translation initiation factor 6	18.9	19.2	64.5	40	0.568
sp|Q02218|ODO1_HUMAN	OGDH	2-oxoglutarate dehydrogenase, mitochondrial	56.18	56.69	54.5	42	0.568

sp|P49815|TSC2_HUMAN	TSC2	Tuberin	8.24	12.7	17.2	10	0.569
sp|P62081|RS7_HUMAN	RPS7	40S ribosomal protein S7	26.38	26.86	78.4	42	0.571
sp|P61201|CSN2_HUMAN	COPS2	COP9 signalosome complex subunit 2	35.99	36.2	62.3	18	0.571
sp|P49356|FNTB_HUMAN	FNTB	Protein farnesyltransferase subunit beta	5.6	5.75	24.9	3	0.571
sp|Q8IZQ1|WDFY3_HUMAN	WDFY3	WD repeat and FYVE domain-containing protein 3	1.3	1.75	9.7	7	0.571
sp|A0AVT1|UBA6_HUMAN	UBA6	Ubiquitin-like modifier-activating enzyme 6	53.53	54.34	51.6	32	0.572
sp|Q9Y3A6|TMED5_HUMAN	TMED5	Transmembrane emp24 domain-containing protein 5	17.41	17.51	52	11	0.572
sp|Q9NW68|BSDC1_HUMAN	BSDC1	BSD domain-containing protein 1	4.27	4.44	28.1	4	0.573
sp|P20340|RAB6A_HUMAN	RAB6A	Ras-related protein Rab-6A	15.97	18.61	72.6	17	0.573
sp|P28066|PSA5_HUMAN	PSMA5	Proteasome subunit alpha type-5	19.76	19.89	72.6	24	0.574
sp|Q9H4B0|OSGP2_HUMAN	OSGEPL1	Probable tRNA N6-adenosine threonylcarbamoyltransferase, mitochondrial	4.05	4.19	24.9	3	0.574
sp|Q6NUS6|TECT3_HUMAN	TCTN3	Tectonic-3	2	2.01	7.1	2	0.577
sp|Q9HA92|RSAD1_HUMAN	RSAD1	Radical S-adenosyl methionine domain-containing protein 1, mitochondrial	4.86	4.92	14.7	3	0.578
sp|Q6P1L8|RM14_HUMAN	MRPL14	39S ribosomal protein L14, mitochondrial	8.99	9.09	55.2	9	0.578
sp|O00488|ZN593_HUMAN	ZNF593	Zinc finger protein 593	7.88	7.95	64.2	4	0.578
sp|Q9UGN5|PARP2_HUMAN	PARP2	Poly [ADP-ribose] polymerase 2	11.64	12.78	42.2	8	0.579
sp|P45984|MK09_HUMAN	MAPK9	Mitogen-activated protein kinase 9	11.39	14.58	39.2	9	0.579
sp|Q8NCF5|NF2IP_HUMAN	NFATC2IP	NFATC2-interacting protein	13.93	14.01	37.2	8	0.579
sp|Q9NQ84|GPC5C_HUMAN	GPRC5C	G-protein coupled receptor family C group 5 member C	8.47	8.61	21.3	6	0.580
sp|Q86SX6|GLRX5_HUMAN	GLRX5	Glutaredoxin-related protein 5, mitochondrial	8.82	8.95	57.3	14	0.580
sp|Q9NTK5|OLA1_HUMAN	OLA1	Obg-like ATPase 1	40.91	40.98	83.3	36	0.581
sp|P83916|CBX1_HUMAN	CBX1	Chromobox protein homolog 1	15.46	17.68	60.5	15	0.581
sp|Q9Y4I1|MYO5A_HUMAN	MYO5A	Unconventional myosin-Va	12.51	14.12	20.3	10	0.581
sp|P06681|CO2_HUMAN	C2	Complement C2	2.03	3.12	16.4	6	0.582
sp|P30046|DOPD_HUMAN	DDT	D-dopachrome decarboxylase	15.51	15.8	67	14	0.582
sp|P51654|GPC3_HUMAN	GPC3	Glypican-3	17.49	17.71	35.7	20	0.582
sp|Q93096|TP4A1_HUMAN	PTP4A1	Protein tyrosine phosphatase type IVA 1	17.44	18.52	67.1	11	0.583
sp|P18077|RL35A_HUMAN	RPL35A	60S ribosomal protein L35a	17.98	19.31	68.2	15	0.583
sp|Q9C0C4|SEM4C_HUMAN	SEMA4C	Semaphorin-4C	2.78	2.87	14.2	3	0.584
sp|Q9H1E3|NUCKS_HUMAN	NUCKS1	Nuclear ubiquitous casein and cyclin-dependent kinase substrate 1	6.28	6.31	30.9	3	0.584
sp|P22087|FBRL_HUMAN	FBL	rRNA 2'-O-methyltransferase fibrillarin	37.53	37.76	85.4	53	0.584
sp|O00567|NOP56_HUMAN	NOP56	Nucleolar protein 56	49.1	49.38	66.5	45	0.584
sp|Q8IWA0|WDR75_HUMAN	WDR75	WD repeat-containing protein 75	43.83	44.14	50	26	0.584
sp|O75506|HSBP1_HUMAN	HSBP1	Heat shock factor-binding protein 1	2	4	50	2	0.585
sp|P11172|UMPS_HUMAN	UMPS	Uridine 5'-monophosphate synthase	49.23	49.29	65	37	0.585
sp|Q9H8P0|PORED_HUMAN	SRD5A3	Polyprenol reductase	2.02	4.13	12	3	0.586
sp|Q9NWU2|GID8_HUMAN	GID8	Glucose-induced degradation protein 8 homolog	12.99	15.36	71.1	10	0.586
sp|Q9UHW5|GPN3_HUMAN	GPN3	GPN-loop GTPase 3	8	8.15	32.8	4	0.586
sp|Q92536|YLAT2_HUMAN	SLC7A6	Y+L amino acid transporter 2	1.85	4.07	7.8	2	0.587
sp|Q9HAV0|GBB4_HUMAN	GNB4	Guanine nucleotide-binding protein subunit beta-4	7.83	17.54	58.2	14	0.587
sp|O75820|ZN189_HUMAN	ZNF189	Zinc finger protein 189	2	3.77	5.9	2	0.587
sp|Q9H8H0|NOL11_HUMAN	NOL11	Nucleolar protein 11	34.89	37.29	59	30	0.588
sp|P67870|CSK2B_HUMAN	CSNK2B	Casein kinase II subunit beta	17.38	17.44	54	20	0.588
sp|Q71UI9|H2AV_HUMAN	H2AFV	Histone H2A.V	6.21	9.2	65.6	15	0.588
sp|Q9C0H2|TTYH3_HUMAN	TTYH3	Protein tweety homolog 3	5.3	5.41	16.3	3	0.589
sp|O43237|DC1L2_HUMAN	DYNC1LI2	Cytoplasmic dynein 1 light intermediate chain 2	22.58	26.92	53.7	18	0.589
sp|P63172|DYLT1_HUMAN	DYNLT1	Dynein light chain Tctex-type 1	10.01	10.02	81.4	10	0.592
sp|Q16836|HCDH_HUMAN	HADH	Hydroxyacyl-coenzyme A dehydrogenase, mitochondrial	31.16	33.01	85.4	30	0.593
sp|Q8N612|F16A2_HUMAN	FAM160A2	FTS and Hook-interacting protein	4	4.02	15.7	5	0.594

sp|Q9H173|SIL1_HUMAN	SIL1	Nucleotide exchange factor SIL1	5.19	5.27	25	4	0.594
sp|Q06033|ITIH3_HUMAN	ITIH3	Inter-alpha-trypsin inhibitor heavy chain H3	3.71	4.17	13.6	3	0.594
sp|O95260|ATE1_HUMAN	ATE1	Arginyl-tRNA--protein transferase 1	10.67	10.86	31.9	7	0.594
sp|Q9BXR0|TGT_HUMAN	QTRT1	Queuine tRNA-ribosyltransferase	15.9	16.04	51.1	12	0.594
sp|P42574|CASP3_HUMAN	CASP3	Caspase-3	12.59	12.84	49.5	7	0.594
sp|Q9H0H5|RGAP1_HUMAN	RACGAP1	Rac GTPase-activating protein 1	11.75	12.32	48.7	6	0.595
sp|Q9UJX5|APC4_HUMAN	ANAPC4	Anaphase-promoting complex subunit 4	18.04	18.52	31.8	11	0.595
sp|Q07817|B2CL1_HUMAN	BCL2L1	Bcl-2-like protein 1	6.13	6.14	13.7	3	0.596
sp|P49840|GSK3A_HUMAN	GSK3A	Glycogen synthase kinase-3 alpha	15.76	18.01	36.9	11	0.597
sp|Q14692|BMS1_HUMAN	BMS1	Ribosome biogenesis protein BMS1 homolog	57.54	59.7	41.3	36	0.597
sp|Q9BV20|MTNA_HUMAN	MRI1	Methylthioribose-1-phosphate isomerase	24.01	24.11	58.8	13	0.597
sp|Q8TEA1|NSUN6_HUMAN	NSUN6	Putative methyltransferase NSUN6	7.37	7.59	29.9	4	0.598
sp|Q9NZC9|SMAL1_HUMAN	SMARCAL1	SWI/SNF-related matrix-associated actin-dependent regulator of chromatin subfamily A-like protein 1	15.23	15.35	23.3	7	0.599
sp|O15212|PFD6_HUMAN	PFDN6	Prefoldin subunit 6	12.17	12.46	69	8	0.599
sp|O95429|BAG4_HUMAN	BAG4	BAG family molecular chaperone regulator 4	13.33	13.55	29.1	7	0.600
sp|P63279|UBC9_HUMAN	UBE2I	SUMO-conjugating enzyme UBC9	13.14	13.9	54.4	10	0.601
sp|Q96G21|IMP4_HUMAN	IMP4	U3 small nucleolar ribonucleoprotein protein IMP4	14.37	15.12	43.6	11	0.601
sp|O60658|PDE8A_HUMAN	PDE8A	High affinity cAMP-specific and IBMX-insensitive 3',5'-cyclic phosphodiesterase 8A	2.2	2.36	16.8	4	0.601
sp|Q13177|PAK2_HUMAN	PAK2	Serine/threonine-protein kinase PAK 2	44.03	44.39	71.8	32	0.601
sp|Q8TBM8|DJB14_HUMAN	DNAJB14	DnaJ homolog subfamily B member 14	4.16	4.35	16.1	4	0.602
sp|O95232|LC7L3_HUMAN	LUC7L3	Luc7-like protein 3	24.16	26.86	43.1	19	0.603
sp|O14656|TOR1A_HUMAN	TOR1A	Torsin-1A	10.02	10.08	31	5	0.605
sp|P07305|H10_HUMAN	H1F0	Histone H1.0	8.72	10.18	49.5	14	0.605
sp|Q9Y3Q3|TMED3_HUMAN	TMED3	Transmembrane emp24 domain-containing protein 3	7.19	7.39	56.2	6	0.605
sp|P62906|RL10A_HUMAN	RPL10A	60S ribosomal protein L10a	25.48	27.83	63.1	24	0.605
sp|Q9UGC7|RF1ML_HUMAN	MTRF1L	Peptide chain release factor 1-like, mitochondrial	1.82	2.55	15.5	2	0.605
sp|Q8ND82|Z280C_HUMAN	ZNF280C	Zinc finger protein 280C	13.57	13.77	30.5	8	0.605
sp|Q13509|TBB3_HUMAN	TUBB3	Tubulin beta-3 chain	13.65	61.81	82	221	0.605
sp|Q9NWQ9|CN119_HUMAN	C14orf119	Uncharacterized protein C14orf119	6	6	24.3	4	0.606
sp|O15260|SURF4_HUMAN	SURF4	Surfeit locus protein 4	10.12	10.19	34.9	18	0.606
sp|Q13243|SRSF5_HUMAN	SRSF5	Serine/arginine-rich splicing factor 5	14.39	19.62	37.5	11	0.606
sp|O15213|WDR46_HUMAN	WDR46	WD repeat-containing protein 46	34.77	36.18	46.6	21	0.607
sp|P51668|UB2D1_HUMAN	UBE2D1	Ubiquitin-conjugating enzyme E2 D1	4.1	8.38	45.6	18	0.607
sp|P83436|COG7_HUMAN	COG7	Conserved oligomeric Golgi complex subunit 7	27.88	28.62	42	16	0.608
sp|Q9P2Y5|UVRAG_HUMAN	UVRAG	UV radiation resistance-associated gene protein	2.1	2.89	18	2	0.608
sp|P55769|NH2L1_HUMAN	NHP2L1	NHP2-like protein 1	19.44	20.73	68	25	0.608
sp|O00154|BACH_HUMAN	ACOT7	Cytosolic acyl coenzyme A thioester hydrolase	18.53	18.56	46.8	14	0.609
sp|Q12849|GRSF1_HUMAN	GRSF1	G-rich sequence factor 1	29.35	29.52	62.9	21	0.609
sp|Q9UET6|TRM7_HUMAN	FTSJ1	Putative tRNA (cytidine(32)/guanosine(34)-2'-O)-methyltransferase	5.15	5.26	25.8	3	0.609
sp|Q9H3S7|PTN23_HUMAN	PTPN23	Tyrosine-protein phosphatase non-receptor type 23	41.31	44.27	27.1	25	0.610
sp|Q01995|TAGL_HUMAN	TAGLN	Transgelin	14.99	15.09	63.2	12	0.610
sp|Q86TS9|RM52_HUMAN	MRPL52	39S ribosomal protein L52, mitochondrial	6.84	7.52	59.4	8	0.611
sp|Q92688|AN32B_HUMAN	ANP32B	Acidic leucine-rich nuclear phosphoprotein 32 family member B	25.03	25.98	35.9	21	0.611
sp|Q06520|ST2A1_HUMAN	SULT2A1	Bile salt sulfotransferase	28.67	30.39	76.8	20	0.611
sp|Q68D85|NR3L1_HUMAN	NCR3LG1	Natural cytotoxicity triggering receptor 3 ligand 1	7.12	7.26	26.9	4	0.611
sp|Q9BV19|CA050_HUMAN	C1orf50	Uncharacterized protein C1orf50	4.02	4.03	36.7	2	0.611
sp|P20839|IMDH1_HUMAN	IMPDH1	Inosine-5'-monophosphate dehydrogenase 1	21.29	31.32	57	22	0.612
sp|Q96P70|IPO9_HUMAN	IPO9	Importin-9	53.32	53.54	40.4	43	0.612
sp|Q15648|MED1_HUMAN	MED1	Mediator of RNA polymerase II transcription subunit 1	21.54	22.61	20.8	16	0.612

sp|O75962|TRIO_HUMAN	TRIO	Triple functional domain protein	4.14	5.35	13.7	6	0.613
sp|Q96IU4|ABHEB_HUMAN	ABHD14B	Alpha/beta hydrolase domain-containing protein 14B	10	10.06	52.4	8	0.613
sp|Q9ULD4|BRPF3_HUMAN	BRPF3	Bromodomain and PHD finger-containing protein 3	2.01	6.5	19.1	5	0.613
sp|Q13277|STX3_HUMAN	STX3	Syntaxin-3	4.63	4.93	31.8	4	0.614
sp|Q9BSC4|NOL10_HUMAN	NOL10	Nucleolar protein 10	39.63	40.24	58.9	23	0.614
sp|Q9UKD2|MRT4_HUMAN	MRTO4	mRNA turnover protein 4 homolog	26.1	27.66	65.3	19	0.615
sp|Q9GZT8|NIF3L_HUMAN	NIF3L1	NIF3-like protein 1	15.66	16.41	40.3	10	0.616
sp|P16152|CBR1_HUMAN	CBR1	Carbonyl reductase [NADPH] 1	28.33	28.49	75.8	22	0.616
sp|Q4VC31|CCD58_HUMAN	CCDC58	Coiled-coil domain-containing protein 58	15.77	15.84	67.4	11	0.617
sp|Q9UHD1|CHRD1_HUMAN	CHORDC1	Cysteine and histidine-rich domain-containing protein 1	33.01	33.92	76.5	19	0.617
sp|P68871|HBB_HUMAN	HBB	Hemoglobin subunit beta	6.2	6.22	53.7	7	0.618
sp|O60573|IF4E2_HUMAN	EIF4E2	Eukaryotic translation initiation factor 4E type 2	6.06	6.12	30.2	6	0.618
sp|P43686|PRS6B_HUMAN	PSMC4	26S protease regulatory subunit 6B	36.95	37.02	76.6	31	0.619
sp|Q9H8M5|CNNM2_HUMAN	CNNM2	Metal transporter CNNM2	2.42	6.36	17.6	8	0.619
sp|Q13636|RAB31_HUMAN	RAB31	Ras-related protein Rab-31	2	6.04	47.9	4	0.620
sp|P25054|APC_HUMAN	APC	Adenomatous polyposis coli protein	2.6	3.36	9.3	5	0.620
sp|P39023|RL3_HUMAN	RPL3	60S ribosomal protein L3	51.99	53.41	68.5	81	0.620
sp|P53611|PGTB2_HUMAN	RABGGTB	Geranylgeranyl transferase type-2 subunit beta	8.04	8.61	30.5	8	0.620
sp|Q12767|K0195_HUMAN	KIAA0195	Uncharacterized protein KIAA0195	1.51	2.61	9	3	0.621
sp|Q12840|KIF5A_HUMAN	KIF5A	Kinesin heavy chain isoform 5A	2.01	14.2	25	9	0.621
sp|Q969M3|YIPF5_HUMAN	YIPF5	Protein YIPF5	4	4.01	24.1	4	0.621
sp|P37108|SRP14_HUMAN	SRP14	Signal recognition particle 14 kDa protein	19.29	19.39	79.4	24	0.621
sp|Q53ET0|CRTC2_HUMAN	CRTC2	CREB-regulated transcription coactivator 2	4	4.05	8.9	3	0.621
sp|Q9NV06|DCA13_HUMAN	DCAF13	DDB1- and CUL4-associated factor 13	25.11	26.01	57.5	17	0.623
sp|Q9BYG3|MK67I_HUMAN	NIFK	MKI67 FHA domain-interacting nucleolar phosphoprotein	35.86	36.28	73.4	21	0.623
sp|O60291|MGRN1_HUMAN	MGRN1	E3 ubiquitin-protein ligase MGRN1	6.84	7.13	21	5	0.623
sp|P46087|NOP2_HUMAN	NOP2	Probable 28S rRNA (cytosine(4447)-C(5))-methyltransferase	54.64	55.46	60.2	41	0.623
sp|Q13427|PPIG_HUMAN	PPIG	Peptidyl-prolyl cis-trans isomerase G	16.51	16.59	26.8	16	0.623
sp|Q9UEE9|CFDP1_HUMAN	CFDP1	Craniofacial development protein 1	9.14	9.45	48.5	7	0.624
sp|Q96QE5|TEFM_HUMAN	TEFM	Transcription elongation factor, mitochondrial	8.88	9.07	33.6	6	0.624
sp|P23381|SYWC_HUMAN	WARS	Tryptophan--tRNA ligase, cytoplasmic	40.68	41.09	73.5	31	0.625
sp|Q9Y6W3|CAN7_HUMAN	CAPN7	Calpain-7	9.89	10.17	25	9	0.625
sp|O15541|R113A_HUMAN	RNF113A	RING finger protein 113A	4.7	4.81	24.5	3	0.625
sp|P26045|PTN3_HUMAN	PTPN3	Tyrosine-protein phosphatase non-receptor type 3	6	6.02	11.1	7	0.625
sp|Q6PJG2|EMSA1_HUMAN	ELMSAN1	ELM2 and SANT domain-containing protein 1	5.36	5.56	18.8	4	0.626
sp|Q9BSL1|UBAC1_HUMAN	UBAC1	Ubiquitin-associated domain-containing protein 1	4.54	4.62	30.1	2	0.626
sp|Q9NRG7|D39U1_HUMAN	SDR39U1	Epimerase family protein SDR39U1	13.77	13.8	30.7	7	0.626
sp|P23528|COF1_HUMAN	CFL1	Cofilin-1	50.96	51.56	96.4	69	0.626
sp|P40429|RL13A_HUMAN	RPL13A	60S ribosomal protein L13a	24.32	31.8	59.6	22	0.626
sp|O14653|GOSR2_HUMAN	GOSR2	Golgi SNAP receptor complex member 2	11.75	11.91	53.3	8	0.627
sp|Q9BRF8|CPPED_HUMAN	CPPED1	Serine/threonine-protein phosphatase CPPED1	27.17	27.3	77.7	27	0.627
sp|Q9UBT7|CTNL1_HUMAN	CTNNAL1	Alpha-catulin	14.56	14.77	39.2	9	0.627
sp|O75494|SRS10_HUMAN	SRSF10	Serine/arginine-rich splicing factor 10	14.49	14.58	31.3	11	0.627
sp|Q9H307|PININ_HUMAN	PNN	Pinin	35.01	35.7	53.7	23	0.627
sp|Q53EZ4|CEP55_HUMAN	CEP55	Centrosomal protein of 55 kDa	6.8	7.12	44.8	5	0.627
sp|P17987|TCPA_HUMAN	TCP1	T-complex protein 1 subunit alpha	70.29	73.43	84.4	114	0.627
sp|O00461|GOLI4_HUMAN	GOLIM4	Golgi integral membrane protein 4	17.04	18.79	23.7	11	0.628
sp|P57723|PCBP4_HUMAN	PCBP4	Poly(rC)-binding protein 4	3.38	5.87	27.5	6	0.628
sp|Q8IWZ8|SUGP1_HUMAN	SUGP1	SURP and G-patch domain-containing protein 1	19.41	20.07	37.1	12	0.628

sp|Q14689|DIP2A_HUMAN	DIP2A	Disco-interacting protein 2 homolog A	2.99	9.32	17.2	6	0.629
sp|P16403|H12_HUMAN	HIST1H1C	Histone H1.2	12.42	44.15	85.9	81	0.629
sp|O00487|PSDE_HUMAN	PSMD14	26S proteasome non-ATPase regulatory subunit 14	24.33	25.1	59.4	20	0.630
sp|O75794|CD123_HUMAN	CDC123	Cell division cycle protein 123 homolog	14.73	16.87	46.4	12	0.631
sp|Q9UBH6|XPR1_HUMAN	XPR1	Xenotropic and polytropic retrovirus receptor 1	3.21	3.4	23.3	3	0.632
sp|Q6DHV7|ADAL_HUMAN	ADAL	Adenosine deaminase-like protein	4.01	4.03	22	2	0.632
sp|P51571|SSRD_HUMAN	SSR4	Translocon-associated protein subunit delta	7.59	7.66	32.4	12	0.633
sp|Q12974|TP4A2_HUMAN	PTP4A2	Protein tyrosine phosphatase type IVA 2	4.28	13.35	67.1	8	0.633
sp|P46779|RL28_HUMAN	RPL28	60S ribosomal protein L28	16.05	16.34	56.9	18	0.633
sp|P22626|ROA2_HUMAN	HNRNPA2B1	Heterogeneous nuclear ribonucleoproteins A2/B1	75.94	76.33	89.2	159	0.634
sp|Q969X6|CIR1A_HUMAN	CIRH1A	Cirhin	41.34	41.43	52.6	28	0.635
sp|O14979|HNRDL_HUMAN	HNRNPDL	Heterogeneous nuclear ribonucleoprotein D-like	47.13	50.28	56.2	46	0.635
sp|P78504|JAG1_HUMAN	JAG1	Protein jagged-1	11.95	12.39	15.9	7	0.636
sp|O43818|U3IP2_HUMAN	RRP9	U3 small nucleolar RNA-interacting protein 2	22.73	22.84	47.2	15	0.636
sp|O75533|SF3B1_HUMAN	SF3B1	Splicing factor 3B subunit 1	105.36	105.78	63.7	73	0.636
sp|P11233|RALA_HUMAN	RALA	Ras-related protein Ral-A	19.7	20.09	61.2	13	0.636
sp|P02647|APOA1_HUMAN	APOA1	Apolipoprotein A-I	10.58	11.72	59.6	8	0.637
sp|Q9BPX3|CND3_HUMAN	NCAPG	Condensin complex subunit 3	35.83	37.42	43.7	22	0.637
sp|Q9H0F7|ARL6_HUMAN	ARL6	ADP-ribosylation factor-like protein 6	10.01	10.06	60.2	6	0.637
sp|P60983|GMFB_HUMAN	GMFB	Glia maturation factor beta	15.18	15.3	76.1	11	0.638
sp|Q9BVV7|TIM21_HUMAN	TIMM21	Mitochondrial import inner membrane translocase subunit Tim21	8.79	8.92	33.5	5	0.638
sp|P05455|LA_HUMAN	SSB	Lupus La protein	81.12	82.01	81.9	51	0.639
sp|P36578|RL4_HUMAN	RPL4	60S ribosomal protein L4	68.46	70.21	67.7	76	0.639
sp|Q96IZ7|RSRC1_HUMAN	RSRC1	Serine/Arginine-related protein 53	6.77	6.95	33.8	5	0.640
sp|P21964|COMT_HUMAN	COMT	Catechol O-methyltransferase	39.52	39.97	78.2	32	0.640
sp|O95391|SLU7_HUMAN	SLU7	Pre-mRNA-splicing factor SLU7	4.79	4.95	33.5	3	0.640
sp|P54136|SYRC_HUMAN	RARS	Arginine--tRNA ligase, cytoplasmic	86.7	89.11	70.6	65	0.640
sp|P63241|IF5A1_HUMAN	EIF5A	Eukaryotic translation initiation factor 5A-1	33.78	33.94	90.3	68	0.640
sp|Q9GZL7|WDR12_HUMAN	WDR12	Ribosome biogenesis protein WDR12	37.37	38.95	75.4	25	0.641
sp|Q6DKI1|RL7L_HUMAN	RPL7L1	60S ribosomal protein L7-like 1	15.28	19.05	58.9	11	0.641
sp|Q9Y2C4|EXOG_HUMAN	EXOG	Nuclease EXOG, mitochondrial	8.66	9.06	46.7	5	0.641
sp|P24390|ERD21_HUMAN	KDELR1	ER lumen protein-retaining receptor 1	4.26	4.34	27.4	5	0.641
sp|Q16254|E2F4_HUMAN	E2F4	Transcription factor E2F4	2	2.42	10.7	3	0.642
sp|P52895|AK1C2_HUMAN	AKR1C2	Aldo-keto reductase family 1 member C2	7.84	66.6	93.2	150	0.642
sp|Q9Y657|SPIN1_HUMAN	SPIN1	Spindlin-1	7.88	7.98	41.2	5	0.642
sp|O43242|PSMD3_HUMAN	PSMD3	26S proteasome non-ATPase regulatory subunit 3	48.45	48.5	64.4	31	0.643
sp|Q14137|BOP1_HUMAN	BOP1	Ribosome biogenesis protein BOP1	47.11	47.15	63.4	30	0.643
sp|Q9UBK8|MTRR_HUMAN	MTRR	Methionine synthase reductase	5.7	6.01	26.1	6	0.643
sp|Q9UM21|MGT4A_HUMAN	MGAT4A	Alpha-1,3-mannosyl-glycoprotein 4-beta-N-acetylglucosaminyltransferase A	2.26	2.29	17.8	2	0.644
sp|Q9NVS9|PNPO_HUMAN	PNPO	Pyridoxine-5'-phosphate oxidase	19.01	19.29	60.5	10	0.644
sp|P51993|FUT6_HUMAN	FUT6	Alpha-(1,3)-fucosyltransferase 6	4	4	7.8	2	0.644
sp|P05388|RLA0_HUMAN	RPLP0	60S acidic ribosomal protein P0	51.42	53.31	72.2	75	0.645
sp|P83731|RL24_HUMAN	RPL24	60S ribosomal protein L24	21.5	21.73	70.7	24	0.645
sp|P14859|PO2F1_HUMAN	POU2F1	POU domain, class 2, transcription factor 1	9.69	9.91	17.1	6	0.645
sp|P19338|NUCL_HUMAN	NCL	Nucleolin	132.17	132.74	64.1	150	0.645
sp|Q0VGL1|LTOR4_HUMAN	LAMTOR4	Ragulator complex protein LAMTOR4	3.05	3.2	40.4	3	0.645
sp|Q10567|AP1B1_HUMAN	AP1B1	AP-1 complex subunit beta-1	31.46	68.39	51	53	0.645
sp|Q9UMY4|SNX12_HUMAN	SNX12	Sorting nexin-12	15.67	15.74	71.5	8	0.645
sp|P43694|GATA4_HUMAN	GATA4	Transcription factor GATA-4	5.75	5.87	19.2	5	0.646

sp|P22392|NDKB_HUMAN	NME2	Nucleoside diphosphate kinase B	24.93	25.08	94.1	36	0.646
sp|P61960|UFM1_HUMAN	UFM1	Ubiquitin-fold modifier 1	7.35	7.49	72.9	6	0.647
sp|Q15397|K0020_HUMAN	KIAA0020	Pumilio domain-containing protein KIAA0020	46.06	47.58	60.3	30	0.648
sp|Q9NQS1|AVEN_HUMAN	AVEN	Cell death regulator Aven	7.27	7.46	34.8	4	0.648
sp|P10599|THIO_HUMAN	TXN	Thioredoxin	21.31	21.39	95.2	37	0.648
sp|Q6NW34|CC017_HUMAN	C3orf17	Uncharacterized protein C3orf17	3.5	3.83	23.1	2	0.649
sp|P61966|AP1S1_HUMAN	AP1S1	AP-1 complex subunit sigma-1A	10.13	10.15	58.9	6	0.649
sp|Q9NZJ7|MTCH1_HUMAN	MTCH1	Mitochondrial carrier homolog 1	24.01	26.02	51.7	14	0.649
sp|Q8NCN5|PDPR_HUMAN	PDPR	Pyruvate dehydrogenase phosphatase regulatory subunit, mitochondrial	32.47	33.01	44.9	22	0.649
sp|Q15691|MARE1_HUMAN	MAPRE1	Microtubule-associated protein RP/EB family member 1	31.55	31.71	89.6	32	0.650
sp|Q9Y262|EIF3L_HUMAN	EIF3L	Eukaryotic translation initiation factor 3 subunit L	51.51	51.74	58.3	38	0.651
sp|Q15012|LAP4A_HUMAN	LAPTM4A	Lysosomal-associated transmembrane protein 4A	4	4.01	17.2	3	0.651
sp|Q8NFH5|NUP53_HUMAN	NUP35	Nucleoporin NUP53	15.05	15.98	62.6	9	0.651
sp|O00571|DDX3X_HUMAN	DDX3X	ATP-dependent RNA helicase DDX3X	66.54	73.16	72.1	64	0.651
sp|Q8NEZ2|VP37A_HUMAN	VPS37A	Vacuolar protein sorting-associated protein 37A	6.41	7	29.5	5	0.652
sp|Q96FJ0|STALP_HUMAN	STAMBPL1	AMSH-like protease	1.77	1.97	21.1	3	0.652
sp|Q96G23|CERS2_HUMAN	CERS2	Ceramide synthase 2	7.54	7.65	32.1	9	0.653
sp|P09429|HMGB1_HUMAN	HMGB1	High mobility group protein B1	37.99	38.74	65.6	38	0.653
sp|Q5BKT4|AG10A_HUMAN	ALG10	Dol-P-Glc:Glc(2)Man(9)GlcNAc(2)-PP-Dol alpha-1,2-glucosyltransferase	4	4.03	16.3	4	0.653
sp|P78344|IF4G2_HUMAN	EIF4G2	Eukaryotic translation initiation factor 4 gamma 2	64.81	67.67	55.5	41	0.653
sp|Q9Y2S7|PDIP2_HUMAN	POLDIP2	Polymerase delta-interacting protein 2	18.65	18.72	48.6	12	0.653
sp|Q99832|TCPH_HUMAN	CCT7	T-complex protein 1 subunit eta	89.19	90.86	87.5	110	0.654
sp|P62241|RS8_HUMAN	RPS8	40S ribosomal protein S8	23.66	23.72	63.9	49	0.654
sp|P62847|RS24_HUMAN	RPS24	40S ribosomal protein S24	6.13	6.84	58.7	16	0.654
sp|Q96HE7|ERO1A_HUMAN	ERO1L	ERO1-like protein alpha	36.7	37.26	65.4	27	0.655
sp|Q6ZWJ1|STXB4_HUMAN	STXBP4	Syntaxin-binding protein 4	9.64	12.42	31.1	8	0.655
sp|Q8TE02|ELP5_HUMAN	ELP5	Elongator complex protein 5	4	4.01	19.6	4	0.655
sp|Q8IZH2|XRN1_HUMAN	XRN1	5'-3' exoribonuclease 1	16.21	18.99	25.4	13	0.655
sp|Q99598|TSNAX_HUMAN	TSNAX	Translin-associated protein X	27.71	27.8	71	23	0.655
sp|P61769|B2MG_HUMAN	B2M	Beta-2-microglobulin	5.46	5.53	51.3	3	0.656
sp|Q16658|FSCN1_HUMAN	FSCN1	Fascin	54.91	55.15	71.8	39	0.656
sp|Q9H0W8|SMG9_HUMAN	SMG9	Protein SMG9	5.71	5.8	19.6	3	0.656
sp|P27635|RL10_HUMAN	RPL10	60S ribosomal protein L10	25.41	25.7	65	57	0.656
sp|P20337|RAB3B_HUMAN	RAB3B	Ras-related protein Rab-3B	2.1	11.01	41.6	10	0.656
sp|P48729|KC1A_HUMAN	CSNK1A1	Casein kinase I isoform alpha	22.53	22.77	51.9	16	0.657
sp|O43390|HNRPR_HUMAN	HNRNPR	Heterogeneous nuclear ribonucleoprotein R	42.64	63.11	72.7	73	0.657
sp|Q9P0J7|KCMF1_HUMAN	KCMF1	E3 ubiquitin-protein ligase KCMF1	7.53	7.74	32.8	6	0.657
sp|P62888|RL30_HUMAN	RPL30	60S ribosomal protein L30	16.94	19.14	89.6	39	0.658
sp|Q15287|RNPS1_HUMAN	RNPS1	RNA-binding protein with serine-rich domain 1	9.29	9.69	22	7	0.658
sp|P06744|G6PI_HUMAN	GPI	Glucose-6-phosphate isomerase	61.78	62.41	65.8	70	0.659
sp|Q5JWF2|GNAS1_HUMAN	GNAS	Guanine nucleotide-binding protein G(s) subunit alpha isoforms XLas	23.97	30.46	24.6	24	0.659
sp|Q9BZJ0|CRNL1_HUMAN	CRNKL1	Crooked neck-like protein 1	43.05	44.75	44.9	25	0.660
sp|O14757|CHK1_HUMAN	CHEK1	Serine/threonine-protein kinase Chk1	8.39	8.53	32.4	5	0.660
sp|Q96JJ7|TMX3_HUMAN	TMX3	Protein disulfide-isomerase TMX3	20.75	26.35	46	16	0.660
sp|Q96JC1|VPS39_HUMAN	VPS39	Vam6/Vps39-like protein	2.06	2.24	17.2	2	0.661
sp|P68104|EF1A1_HUMAN	EEF1A1	Elongation factor 1-alpha 1	115.73	116.58	92	374	0.661
sp|Q13885|TBB2A_HUMAN	TUBB2A	Tubulin beta-2A chain	13.92	83.42	84.5	299	0.661
sp|O75844|FACE1_HUMAN	ZMPSTE24	CAAX prenyl protease 1 homolog	19.29	19.53	37.5	11	0.661
sp|O00264|PGRC1_HUMAN	PGRMC1	Membrane-associated progesterone receptor component 1	23.97	24.01	62.6	17	0.661

sp|Q8N5I9|CL045_HUMAN	C12orf45	Uncharacterized protein C12orf45	2.77	2.84	27.6	2	0.661
sp|Q9Y6K5|OAS3_HUMAN	OAS3	2'-5'-oligoadenylate synthase 3	6.96	7.25	15.7	4	0.661
sp|O75083|WDR1_HUMAN	WDR1	WD repeat-containing protein 1	60.5	60.97	67.3	55	0.662
sp|P35270|SPRE_HUMAN	SPR	Sepiapterin reductase	22.36	22.55	60.2	15	0.662
sp|Q9H497|TOR3A_HUMAN	TOR3A	Torsin-3A	6.45	6.51	33.5	4	0.662
sp|Q9NX18|SDHF2_HUMAN	SDHAF2	Succinate dehydrogenase assembly factor 2, mitochondrial	7.7	7.81	53.6	5	0.662
sp|Q70J99|UN13D_HUMAN	UNC13D	Protein unc-13 homolog D	5.18	5.62	15.3	4	0.662
sp|P31944|CASPE_HUMAN	CASP14	Caspase-14	4.03	4.38	39.7	3	0.662
sp|P84103|SRSF3_HUMAN	SRSF3	Serine/arginine-rich splicing factor 3	17.49	17.72	60.4	24	0.663
sp|Q9Y2Y1|RPC10_HUMAN	POLR3K	DNA-directed RNA polymerase III subunit RPC10	2.31	2.34	50	4	0.663
sp|P41250|SYG_HUMAN	GARS	Glycine--tRNA ligase	60.84	62	67	49	0.664
sp|Q16186|ADRM1_HUMAN	ADRM1	Proteasomal ubiquitin receptor ADRM1	18.36	20.57	39.8	23	0.664
sp|P18074|ERCC2_HUMAN	ERCC2	TFIIH basal transcription factor complex helicase XPD subunit	18.15	18.3	33.4	10	0.665
sp|Q9NZR1|TMOD2_HUMAN	TMOD2	Tropomodulin-2	2.01	2.28	32.5	2	0.665
sp|P35579|MYH9_HUMAN	MYH9	Myosin-9	242.64	242.23	68.6	211	0.666
sp|Q00534|CDK6_HUMAN	CDK6	Cyclin-dependent kinase 6	14.39	17.67	38.7	11	0.666
sp|Q9Y2B2|PIGL_HUMAN	PIGL	N-acetylglucosaminyl-phosphatidylinositol de-N-acetylase	4.68	4.77	34.5	4	0.667
sp|Q9H4L7|SMRCD_HUMAN	SMARCAD1	SWI/SNF-related matrix-associated actin-dependent regulator of chromatin subfamily A	27.55	27.94	38.7	17	0.668
sp|Q969Q0|RL36L_HUMAN	RPL36AL	60S ribosomal protein L36a-like	2	11.27	66	11	0.668
sp|Q8TED1|GPX8_HUMAN	GPX8	Probable glutathione peroxidase 8	8.2	9.04	49.8	7	0.669
sp|Q9BXY0|MAK16_HUMAN	MAK16	Protein MAK16 homolog	19.49	21.29	50.7	13	0.669
sp|P31146|COR1A_HUMAN	CORO1A	Coronin-1A	17	19.32	45.3	9	0.670
sp|Q9C0E2|XPO4_HUMAN	XPO4	Exportin-4	18.38	18.7	24.9	14	0.670
sp|O14681|EI24_HUMAN	EI24	Etoposide-induced protein 2.4 homolog	4.09	4.15	22.7	2	0.670
sp|Q96AT9|RPE_HUMAN	RPE	Ribulose-phosphate 3-epimerase	9.99	11.65	48.3	9	0.670
sp|P10114|RAP2A_HUMAN	RAP2A	Ras-related protein Rap-2a	4.01	17.08	70.5	13	0.670
sp|P21266|GSTM3_HUMAN	GSTM3	Glutathione S-transferase Mu 3	29.75	29.96	78.7	18	0.670
sp|Q9UHA3|RLP24_HUMAN	RSL24D1	Probable ribosome biogenesis protein RLP24	6	6.13	47.9	4	0.670
sp|P31321|KAP1_HUMAN	PRKAR1B	cAMP-dependent protein kinase type I-beta regulatory subunit	4.04	9.79	47.2	5	0.671
sp|O60568|PLOD3_HUMAN	PLOD3	Procollagen-lysine,2-oxoglutarate 5-dioxygenase 3	40.63	40.91	49.7	26	0.671
sp|Q16854|DGUOK_HUMAN	DGUOK	Deoxyguanosine kinase, mitochondrial	6.17	6.29	40.4	3	0.671
sp|Q12788|TBL3_HUMAN	TBL3	Transducin beta-like protein 3	56.46	56.76	68.6	38	0.672
sp|Q15208|STK38_HUMAN	STK38	Serine/threonine-protein kinase 38	8.08	8.7	28.6	6	0.672
sp|Q9H5Q4|TFB2M_HUMAN	TFB2M	Dimethyladenosine transferase 2, mitochondrial	10.67	10.87	35.1	6	0.672
sp|O43301|HS12A_HUMAN	HSPA12A	Heat shock 70 kDa protein 12A	16.36	16.77	27.4	10	0.672
sp|P09564|CD7_HUMAN	CD7	T-cell antigen CD7	5.07	5.12	16.7	5	0.673
sp|O43747|AP1G1_HUMAN	AP1G1	AP-1 complex subunit gamma-1	42.83	44.23	41.2	31	0.674
sp|Q9H9L3|I20L2_HUMAN	ISG20L2	Interferon-stimulated 20 kDa exonuclease-like 2	5.04	5.13	27.8	3	0.675
sp|Q99460|PSMD1_HUMAN	PSMD1	26S proteasome non-ATPase regulatory subunit 1	87.82	88.9	70.6	66	0.675
sp|P62861|RS30_HUMAN	FAU	40S ribosomal protein S30	4.07	4.08	20.3	4	0.675
sp|Q8TDY4|ASAP3_HUMAN	ASAP3	Arf-GAP with SH3 domain, ANK repeat and PH domain-containing protein 3	2.65	3.17	14.3	5	0.675
sp|Q8WWQ0|PHIP_HUMAN	PHIP	PH-interacting protein	33.82	34.57	24.7	16	0.675
sp|P08243|ASNS_HUMAN	ASNS	Asparagine synthetase [glutamine-hydrolyzing]	53.64	53.7	71.5	45	0.675
sp|Q96KC2|ARL5B_HUMAN	ARL5B	ADP-ribosylation factor-like protein 5B	5.93	6.1	35.2	5	0.676
sp|P28347|TEAD1_HUMAN	TEAD1	Transcriptional enhancer factor TEF-1	4	4.01	14.8	2	0.676
sp|Q12904|AIMP1_HUMAN	AIMP1	Aminoacyl tRNA synthase complex-interacting multifunctional protein 1	37.6	39.98	82.7	34	0.676
sp|Q14142|TRI14_HUMAN	TRIM14	Tripartite motif-containing protein 14	2.05	2.46	19.5	3	0.676
sp|Q9UHN6|TMEM2_HUMAN	TMEM2	Transmembrane protein 2	48.38	48.99	38.6	26	0.677
sp|Q5TH69|BIG3_HUMAN	ARFGEF3	Brefeldin A-inhibited guanine nucleotide-exchange protein 3	2.04	2.27	12.1	2	0.677

sp|P20226|TBP_HUMAN	TBP	TATA-box-binding protein	2.52	2.57	17.1	2	0.677
sp|Q8TCT9|HM13_HUMAN	HM13	Minor histocompatibility antigen H13	17.13	17.28	31.6	12	0.677
sp|Q9UJW0|DCTN4_HUMAN	DCTN4	Dynactin subunit 4	19.23	19.35	47.8	13	0.677
sp|P12235|ADT1_HUMAN	SLC25A4	ADP/ATP translocase 1	8.33	46.63	76.2	53	0.677
sp|Q9Y4E8|UBP15_HUMAN	USP15	Ubiquitin carboxyl-terminal hydrolase 15	46.37	48.08	45.1	25	0.677
sp|O94842|TOX4_HUMAN	TOX4	TOX high mobility group box family member 4	15.11	15.47	23	9	0.677
sp|P51798|CLCN7_HUMAN	CLCN7	H(+)/Cl(-) exchange transporter 7	14.99	15.34	22.4	10	0.677
sp|Q8TD55|PKHO2_HUMAN	PLEKHO2	Pleckstrin homology domain-containing family O member 2	2.61	2.72	20	2	0.678
sp|P05026|AT1B1_HUMAN	ATP1B1	Sodium/potassium-transporting ATPase subunit beta-1	18.9	19.07	51.8	13	0.678
sp|O00754|MA2B1_HUMAN	MAN2B1	Lysosomal alpha-mannosidase	38.14	38.32	40.2	28	0.679
sp|Q9ULJ6|ZMIZ1_HUMAN	ZMIZ1	Zinc finger MIZ domain-containing protein 1	6.95	10.67	12.3	7	0.679
sp|P63173|RL38_HUMAN	RPL38	60S ribosomal protein L38	14	14.25	57.1	13	0.679
sp|P08962|CD63_HUMAN	CD63	CD63 antigen	8.07	9.2	26.9	8	0.679
sp|Q14103|HNRPD_HUMAN	HNRNPD	Heterogeneous nuclear ribonucleoprotein D0	40.35	48.36	71	74	0.679
sp|P53814|SMTN_HUMAN	SMTN	Smoothelin	2.99	3.19	15.1	3	0.679
sp|Q92626|PXDN_HUMAN	PXDN	Peroxidasin homolog	1.71	2.05	10	3	0.679
sp|Q9UL15|BAG5_HUMAN	BAG5	BAG family molecular chaperone regulator 5	19.05	20.76	49.9	11	0.679
sp|Q96BM9|ARL8A_HUMAN	ARL8A	ADP-ribosylation factor-like protein 8A	4.03	16.48	68.3	11	0.680
sp|P68363|TBA1B_HUMAN	TUBA1B	Tubulin alpha-1B chain	93.99	94.05	92.7	345	0.680
sp|Q99707|METH_HUMAN	MTR	Methionine synthase	18.39	18.76	29.8	14	0.680
sp|O43324|MCA3_HUMAN	EEF1E1	Eukaryotic translation elongation factor 1 epsilon-1	12.08	12.39	69	17	0.680
sp|Q13595|TRA2A_HUMAN	TRA2A	Transformer-2 protein homolog alpha	16.12	16.2	45.4	15	0.680
sp|Q16629|SRSF7_HUMAN	SRSF7	Serine/arginine-rich splicing factor 7	12.31	14.72	29.4	15	0.681
sp|Q9P1F3|ABRAL_HUMAN	ABRACL	Costars family protein ABRACL	10.06	10.08	86.4	7	0.681
sp|Q92979|NEP1_HUMAN	EMG1	Ribosomal RNA small subunit methyltransferase NEP1	20.21	21.19	86.5	18	0.682
sp|Q9UBQ6|EXTL2_HUMAN	EXTL2	Exostosin-like 2	2	2.05	13.3	2	0.682
sp|Q13432|U119A_HUMAN	UNC119	Protein unc-119 homolog A	4	6.01	20.8	3	0.683
sp|P62841|RS15_HUMAN	RPS15	40S ribosomal protein S15	6.36	6.49	62.1	23	0.683
sp|Q9BQ61|CS043_HUMAN	C19orf43	Uncharacterized protein C19orf43	11.52	12.11	42.1	8	0.683
sp|P32119|PRDX2_HUMAN	PRDX2	Peroxiredoxin-2	26.95	30.07	79.8	43	0.684
sp|Q9NU23|LYRM2_HUMAN	LYRM2	LYR motif-containing protein 2	4.68	4.86	63.6	3	0.684
sp|Q00610|CLH1_HUMAN	CLTC	Clathrin heavy chain 1	198.38	200.63	73.6	248	0.684
sp|Q14118|DAG1_HUMAN	DAG1	Dystroglycan	18.8	19.72	22.9	13	0.684
sp|Q9NVP1|DDX18_HUMAN	DDX18	ATP-dependent RNA helicase DDX18	57.7	59.51	63.9	55	0.684
sp|Q9H9Y2|RPF1_HUMAN	RPF1	Ribosome production factor 1	13.28	13.63	46.4	10	0.684
sp|P30405|PPIF_HUMAN	PPIF	Peptidyl-prolyl cis-trans isomerase F, mitochondrial	21.96	24.5	69.1	40	0.685
sp|P14923|PLAK_HUMAN	JUP	Junction plakoglobin	35.3	50.9	57.5	34	0.685
sp|Q96DE0|NUD16_HUMAN	NUDT16	U8 snoRNA-decapping enzyme	13.31	13.41	61	7	0.685
sp|Q6DD88|ATLA3_HUMAN	ATL3	Atlastin-3	12.62	15.6	37.2	11	0.685
sp|P30085|KCY_HUMAN	CMPK1	UMP-CMP kinase	16.62	18.57	66.8	11	0.686
sp|Q9UJX4|APC5_HUMAN	ANAPC5	Anaphase-promoting complex subunit 5	8.27	9.04	30.3	5	0.686
sp|O00308|WWP2_HUMAN	WWP2	NEDD4-like E3 ubiquitin-protein ligase WWP2	5.54	5.71	17.9	4	0.686
sp|Q9Y3B4|SF3B6_HUMAN	SF3B6	Splicing factor 3B subunit 6	10.63	11.75	71.2	7	0.686
sp|Q969J3|L12R1_HUMAN	LOH12CR1	Loss of heterozygosity 12 chromosomal region 1 protein	4.26	4.28	25	2	0.686
sp|Q99523|SORT_HUMAN	SORT1	Sortilin	14.56	19.15	24.1	10	0.686
sp|Q9NR33|DPOE4_HUMAN	POLE4	DNA polymerase epsilon subunit 4	10	10	85.5	10	0.686
sp|Q92905|CSN5_HUMAN	COPS5	COP9 signalosome complex subunit 5	25.48	25.77	58.7	19	0.686
sp|O95900|TRUB2_HUMAN	TRUB2	Probable tRNA pseudouridine synthase 2	5.9	6	27.5	3	0.686
sp|P60842|IF4A1_HUMAN	EIF4A1	Eukaryotic initiation factor 4A-I	65.31	65.8	83.3	100	0.686

sp|Q9H3N1|TMX1_HUMAN	TMX1	Thioredoxin-related transmembrane protein 1	17.67	17.78	30.4	11	0.687
sp|Q9NRN9|METL5_HUMAN	METTL5	Methyltransferase-like protein 5	7.38	8.72	42.6	5	0.687
sp|Q86Y79|PTH_HUMAN	PTRH1	Probable peptidyl-tRNA hydrolase	7.57	7.65	49.1	6	0.687
sp|P49643|PRI2_HUMAN	PRIM2	DNA primase large subunit	19.56	19.77	35.2	11	0.687
sp|Q9BZM4|N2DL3_HUMAN	ULBP3	NKG2D ligand 3	2.75	2.82	29.1	2	0.688
sp|P53618|COPB_HUMAN	COPB1	Coatomer subunit beta	75.39	77.45	61.9	70	0.688
sp|O75352|MPU1_HUMAN	MPDU1	Mannose-P-dolichol utilization defect 1 protein	6.02	6.32	27.5	4	0.688
sp|Q9BUJ2|HNRL1_HUMAN	HNRNPUL1	Heterogeneous nuclear ribonucleoprotein U-like protein 1	47.86	50.17	49	34	0.688
sp|Q01518|CAP1_HUMAN	CAP1	Adenylyl cyclase-associated protein 1	49.51	49.63	71.4	48	0.688
sp|Q02818|NUCB1_HUMAN	NUCB1	Nucleobindin-1	38.09	39.43	64.9	23	0.689
sp|Q9UK99|FBX3_HUMAN	FBXO3	F-box only protein 3	4.97	5.7	20.2	4	0.689
sp|Q96KG9|NTKL_HUMAN	SCYL1	N-terminal kinase-like protein	28.16	28.34	38.2	14	0.689
sp|P48643|TCPE_HUMAN	CCT5	T-complex protein 1 subunit epsilon	89.99	90.23	83	116	0.689
sp|P47755|CAZA2_HUMAN	CAPZA2	F-actin-capping protein subunit alpha-2	27.01	28.28	76.9	22	0.689
sp|P15531|NDKA_HUMAN	NME1	Nucleoside diphosphate kinase A	13.35	24.68	96.1	26	0.689
sp|P61020|RAB5B_HUMAN	RAB5B	Ras-related protein Rab-5B	8.33	15.54	69.8	16	0.690
sp|O43314|VIP2_HUMAN	PPIP5K2	Inositol hexakisphosphate and diphosphoinositol-pentakisphosphate kinase 2	12.23	13.7	27.4	9	0.690
sp|P51659|DHB4_HUMAN	HSD17B4	Peroxisomal multifunctional enzyme type 2	72.46	72.56	74.1	60	0.690
sp|Q9H993|ARMT1_HUMAN	ARMT1	Protein-glutamate O-methyltransferase	27.67	27.82	56.9	17	0.690
sp|P18440|ARY1_HUMAN	NAT1	Arylamine N-acetyltransferase 1	4.49	4.65	29.7	5	0.691
sp|Q13347|EIF3I_HUMAN	EIF3I	Eukaryotic translation initiation factor 3 subunit I	34.35	36.96	84.9	34	0.691
sp|Q14376|GALE_HUMAN	GALE	UDP-glucose 4-epimerase	21.66	21.9	50	13	0.692
sp|O14672|ADA10_HUMAN	ADAM10	Disintegrin and metalloproteinase domain-containing protein 10	15.13	15.28	21	12	0.692
sp|O94925|GLSK_HUMAN	GLS	Glutaminase kidney isoform, mitochondrial	50.65	51.61	55.8	42	0.693
sp|Q92968|PEX13_HUMAN	PEX13	Peroxisomal membrane protein PEX13	4.06	4.08	23.8	5	0.694
sp|Q9BZE4|NOG1_HUMAN	GTPBP4	Nucleolar GTP-binding protein 1	53.19	54.75	58.8	30	0.694
sp|Q01968|OCRL_HUMAN	OCRL	Inositol polyphosphate 5-phosphatase OCRL-1	21.42	23.28	30.3	13	0.694
sp|Q9BVJ6|UT14A_HUMAN	UTP14A	U3 small nucleolar RNA-associated protein 14 homolog A	64.64	64.8	57.5	34	0.694
sp|Q05D32|CTSL2_HUMAN	CTDSPL2	CTD small phosphatase-like protein 2	5.85	6.18	30	7	0.694
sp|Q02878|RL6_HUMAN	RPL6	60S ribosomal protein L6	35.55	37.24	59.4	28	0.695
sp|Q9BUH6|PAXX_HUMAN	C9orf142	Protein PAXX	13.24	13.3	56.4	7	0.695
sp|Q3KQV9|UAP1L_HUMAN	UAP1L1	UDP-N-acetylhexosamine pyrophosphorylase-like protein 1	24.79	24.89	47.3	12	0.695
sp|Q12906|ILF3_HUMAN	ILF3	Interleukin enhancer-binding factor 3	89.33	89.81	64.7	91	0.695
sp|Q05682|CALD1_HUMAN	CALD1	Caldesmon	35.75	37.46	46.5	23	0.695
sp|Q9NRW3|ABC3C_HUMAN	APOBEC3C	DNA dC->dU-editing enzyme APOBEC-3C	6.31	6.43	53.2	4	0.695
sp|Q07866|KLC1_HUMAN	KLC1	Kinesin light chain 1	21.08	42.97	58.1	29	0.695
sp|Q02750|MP2K1_HUMAN	MAP2K1	Dual specificity mitogen-activated protein kinase kinase 1	10.12	19.92	47.3	19	0.696
sp|Q2TAL8|QRIC1_HUMAN	QRICH1	Glutamine-rich protein 1	14	15.34	21.9	8	0.696
sp|P61024|CKS1_HUMAN	CKS1B	Cyclin-dependent kinases regulatory subunit 1	4	4.17	60.8	4	0.696
sp|Q9BVP2|GNL3_HUMAN	GNL3	Guanine nucleotide-binding protein-like 3	46.68	47.96	57.9	41	0.696
sp|P26368|U2AF2_HUMAN	U2AF2	Splicing factor U2AF 65 kDa subunit	36.11	37.26	69.7	63	0.696
sp|Q92783|STAM1_HUMAN	STAM	Signal transducing adapter molecule 1	17.89	18.07	31.9	10	0.696
sp|P18085|ARF4_HUMAN	ARF4	ADP-ribosylation factor 4	10.85	23.93	95	28	0.696
sp|P51784|UBP11_HUMAN	USP11	Ubiquitin carboxyl-terminal hydrolase 11	27.81	30.59	35.4	20	0.696
sp|Q9NW13|RBM28_HUMAN	RBM28	RNA-binding protein 28	49.89	50.75	48.8	30	0.697
sp|P18859|ATP5J_HUMAN	ATP5J	ATP synthase-coupling factor 6, mitochondrial	12.45	13.23	71.3	17	0.697
sp|Q9BX66|SRBS1_HUMAN	SORBS1	Sorbin and SH3 domain-containing protein 1	10.8	13.79	16.2	8	0.698
sp|O14925|TIM23_HUMAN	TIMM23	Mitochondrial import inner membrane translocase subunit Tim23	12.75	12.86	70.3	10	0.698
sp|Q03154|ACY1_HUMAN	ACY1	Aminoacylase-1	38.03	38.08	75.3	23	0.698

sp|Q9Y696|CLIC4_HUMAN	CLIC4	Chloride intracellular channel protein 4	22.79	26.9	79.1	18	0.698
sp|Q14320|FA50A_HUMAN	FAM50A	Protein FAM50A	14.82	15.16	48.1	13	0.698
sp|Q9Y3C8|UFC1_HUMAN	UFC1	Ubiquitin-fold modifier-conjugating enzyme 1	7.09	7.18	47.9	5	0.698
sp|P11940|PABP1_HUMAN	PABPC1	Polyadenylate-binding protein 1	72.79	73.58	75	77	0.698
sp|Q9BW72|HIG2A_HUMAN	HIGD2A	HIG1 domain family member 2A, mitochondrial	3.35	3.43	50.9	2	0.699
sp|Q99757|THIOM_HUMAN	TXN2	Thioredoxin, mitochondrial	9.41	9.73	60.2	11	0.699
sp|Q01484|ANK2_HUMAN	ANK2	Ankyrin-2	13.61	18.54	12	11	0.699
sp|Q9NXE4|NSMA3_HUMAN	SMPD4	Sphingomyelin phosphodiesterase 4	38.53	38.78	42.7	21	0.699
sp|Q7L523|RRAGA_HUMAN	RRAGA	Ras-related GTP-binding protein A	14.79	17.23	44.7	9	0.699
sp|P23458|JAK1_HUMAN	JAK1	Tyrosine-protein kinase JAK1	14.2	15.06	24.5	10	0.700
sp|P17612|KAPCA_HUMAN	PRKACA	cAMP-dependent protein kinase catalytic subunit alpha	20.19	23.94	64.1	16	0.700
sp|Q6IAA8|LTOR1_HUMAN	LAMTOR1	Ragulator complex protein LAMTOR1	12.59	12.69	83.9	11	0.701
sp|P38159|RBMX_HUMAN	RBMX	RNA-binding motif protein, X chromosome	29.81	30.72	47.8	33	0.701
sp|Q9BWU0|NADAP_HUMAN	SLC4A1AP	Kanadaptin	36.29	36.48	45.5	25	0.701
sp|P04637|P53_HUMAN	TP53	Cellular tumor antigen p53	18.19	19.29	45.6	13	0.701
sp|P54802|ANAG_HUMAN	NAGLU	Alpha-N-acetylglucosaminidase	12.15	12.19	21	6	0.702
sp|Q9NRR4|RNC_HUMAN	DROSHA	Ribonuclease 3	6.02	6.13	13.3	7	0.702
sp|P41567|EIF1_HUMAN	EIF1	Eukaryotic translation initiation factor 1	22.22	22.27	83.2	15	0.702
sp|Q92844|TANK_HUMAN	TANK	TRAF family member-associated NF-kappa-B activator	2.44	2.48	13.9	2	0.702
sp|P50454|SERPH_HUMAN	SERPINH1	Serpin H1	47.07	47.17	76.8	64	0.702
sp|Q9UNF1|MAGD2_HUMAN	MAGED2	Melanoma-associated antigen D2	41.31	42.17	60.7	25	0.702
sp|P61927|RL37_HUMAN	RPL37	60S ribosomal protein L37	4.79	5.5	34	5	0.703
sp|Q86YB8|ERO1B_HUMAN	ERO1LB	ERO1-like protein beta	2.4	5.63	34.3	5	0.703
sp|Q4J6C6|PPCEL_HUMAN	PREPL	Prolyl endopeptidase-like	21.84	22.1	35.5	14	0.703
sp|Q9ULM3|YETS2_HUMAN	YEATS2	YEATS domain-containing protein 2	9.35	11.72	20.2	8	0.703
sp|P36543|VATE1_HUMAN	ATP6V1E1	V-type proton ATPase subunit E 1	23.88	23.97	65.9	13	0.703
sp|Q8IXJ6|SIR2_HUMAN	SIRT2	NAD-dependent protein deacetylase sirtuin-2	2.05	2.63	16.5	2	0.704
sp|Q08AE8|SPIR1_HUMAN	SPIRE1	Protein spire homolog 1	1.39	3.28	12	2	0.704
sp|Q9BX67|JAM3_HUMAN	JAM3	Junctional adhesion molecule C	10.3	10.52	38.1	7	0.704
sp|P68371|TBB4B_HUMAN	TUBB4B	Tubulin beta-4B chain	2	91.94	84.5	349	0.705
sp|P07910|HNRPC_HUMAN	HNRNPC	Heterogeneous nuclear ribonucleoproteins C1/C2	43.92	45.47	72.9	113	0.705
sp|P60981|DEST_HUMAN	DSTN	Destrin	31.95	33.89	86.7	22	0.705
sp|Q9UDW1|QCR9_HUMAN	UQCR10	Cytochrome b-c1 complex subunit 9	4.01	4.01	57.1	3	0.706
sp|Q14181|DPOA2_HUMAN	POLA2	DNA polymerase alpha subunit B	17.44	17.61	29.1	9	0.706
sp|Q86WC4|OSTM1_HUMAN	OSTM1	Osteopetrosis-associated transmembrane protein 1	3.8	3.89	11.4	2	0.706
sp|Q05655|KPCD_HUMAN	PRKCD	Protein kinase C delta type	30.12	30.61	47.8	20	0.707
sp|Q9H840|GEMI7_HUMAN	GEMIN7	Gem-associated protein 7	6.16	6.18	70.2	5	0.707
sp|Q8TDN6|BRX1_HUMAN	BRIX1	Ribosome biogenesis protein BRX1 homolog	20.59	20.72	57.8	16	0.707
sp|Q9Y2Z9|COQ6_HUMAN	COQ6	Ubiquinone biosynthesis monooxygenase COQ6, mitochondrial	4.02	4.13	9.2	4	0.707
sp|Q7Z7K6|CENPV_HUMAN	CENPV	Centromere protein V	11.73	11.89	61.5	14	0.707
sp|P07339|CATD_HUMAN	CTSD	Cathepsin D	35.33	35.36	53.6	40	0.707
sp|Q13643|FHL3_HUMAN	FHL3	Four and a half LIM domains protein 3	3.09	3.22	19.3	3	0.707
sp|P45877|PPIC_HUMAN	PPIC	Peptidyl-prolyl cis-trans isomerase C	7.57	8.53	41.5	11	0.707
sp|O00625|PIR_HUMAN	PIR	Pirin	10.24	10.31	51	8	0.708
sp|Q9Y3B3|TMED7_HUMAN	TMED7	Transmembrane emp24 domain-containing protein 7	12.6	12.76	65.6	12	0.708
sp|Q07955|SRSF1_HUMAN	SRSF1	Serine/arginine-rich splicing factor 1	28.66	29.65	66.1	22	0.708
sp|P62979|RS27A_HUMAN	RPS27A	Ubiquitin-40S ribosomal protein S27a	33.24	34.88	84.6	56	0.708
sp|Q9NY12|GAR1_HUMAN	GAR1	H/ACA ribonucleoprotein complex subunit 1	8.93	9.16	55.8	6	0.708
sp|Q49AM1|MTEF2_HUMAN	MTERF2	Transcription termination factor 2, mitochondrial	2.01	2.02	23.6	2	0.709

sp|Q2Q1W2|LIN41_HUMAN	TRIM71	E3 ubiquitin-protein ligase TRIM71	34.42	34.75	44.4	19	0.709
sp|P23284|PPIB_HUMAN	PPIB	Peptidyl-prolyl cis-trans isomerase B	27.99	29.94	73.2	33	0.709
sp|Q9BUA3|CK084_HUMAN	C11orf84	Uncharacterized protein C11orf84	8.19	8.33	28.1	5	0.709
sp|Q9H3S5|PIGM_HUMAN	PIGM	GPI mannosyltransferase 1	2.86	2.93	18.7	2	0.709
sp|Q13601|KRR1_HUMAN	KRR1	KRR1 small subunit processome component homolog	31.49	35.19	68.5	20	0.709
sp|A6NNH0|GATL1_HUMAN	GATSL1	GATS-like protein 1	3.13	3.2	20.4	3	0.709
sp|P06280|AGAL_HUMAN	GLA	Alpha-galactosidase A	20.18	20.24	52.7	14	0.710
sp|P35580|MYH10_HUMAN	MYH10	Myosin-10	186	219.21	67.4	148	0.710
sp|Q9NSD9|SYFB_HUMAN	FARSB	Phenylalanine--tRNA ligase beta subunit	48.14	48.71	56.5	31	0.710
sp|P62750|RL23A_HUMAN	RPL23A	60S ribosomal protein L23a	24.04	24.27	61.5	24	0.710
sp|Q6UWP2|DHR11_HUMAN	DHRS11	Dehydrogenase/reductase SDR family member 11	6.4	6.43	29.6	3	0.710
sp|Q96S90|LYSM1_HUMAN	LYSMD1	LysM and putative peptidoglycan-binding domain-containing protein 1	2.1	2.78	18.9	2	0.711
sp|Q13426|XRCC4_HUMAN	XRCC4	DNA repair protein XRCC4	9.18	9.38	34.8	6	0.711
sp|Q5VUB5|F1711_HUMAN	FAM171A1	Protein FAM171A1	2	2.03	8.5	2	0.711
sp|Q9NUB1|ACS2L_HUMAN	ACSS1	Acetyl-coenzyme A synthetase 2-like, mitochondrial	28.8	29.24	47.9	16	0.712
sp|Q8NEF9|SRFB1_HUMAN	SRFBP1	Serum response factor-binding protein 1	9.26	11.21	35.2	8	0.712
sp|Q9BVS4|RIOK2_HUMAN	RIOK2	Serine/threonine-protein kinase RIO2	13.46	14.6	33.2	9	0.712
sp|Q96FW1|OTUB1_HUMAN	OTUB1	Ubiquitin thioesterase OTUB1	30.2	30.41	77.1	22	0.712
sp|Q9NWT1|PK1IP_HUMAN	PAK1IP1	p21-activated protein kinase-interacting protein 1	30.85	30.89	54.9	18	0.712
sp|Q8NI36|WDR36_HUMAN	WDR36	WD repeat-containing protein 36	42.49	42.98	40.5	28	0.712
sp|O00115|DNS2A_HUMAN	DNASE2	Deoxyribonuclease-2-alpha	7.19	7.47	22.2	6	0.712
sp|Q9P2W9|STX18_HUMAN	STX18	Syntaxin-18	23.32	23.36	52.5	13	0.712
sp|P54289|CA2D1_HUMAN	CACNA2D1	Voltage-dependent calcium channel subunit alpha-2/delta-1	4.86	5.22	22.7	3	0.713
sp|Q969R5|LMBL2_HUMAN	L3MBTL2	Lethal(3)malignant brain tumor-like protein 2	4.38	4.42	10.8	3	0.713
sp|P47897|SYQ_HUMAN	QARS	Glutamine--tRNA ligase	75.92	78.68	69.9	51	0.713
sp|P08758|ANXA5_HUMAN	ANXA5	Annexin A5	48.3	52.67	78.8	61	0.713
sp|Q13813|SPTN1_HUMAN	SPTAN1	Spectrin alpha chain, non-erythrocytic 1	302.41	302.45	78.6	222	0.713
sp|E9PAV3|NACAM_HUMAN	NACA	Nascent polypeptide-associated complex subunit alpha, muscle-specific form	13.25	14.75	13.6	31	0.713
sp|Q9H078|CLPB_HUMAN	CLPB	Caseinolytic peptidase B protein homolog	33.69	34.32	52.6	24	0.714
sp|P61956|SUMO2_HUMAN	SUMO2	Small ubiquitin-related modifier 2	5.02	5.12	67.4	12	0.714
sp|Q9NYH9|UTP6_HUMAN	UTP6	U3 small nucleolar RNA-associated protein 6 homolog	45.94	46.12	53.8	26	0.714
sp|Q5QJE6|TDIF2_HUMAN	DNTTIP2	Deoxynucleotidyltransferase terminal-interacting protein 2	40.64	40.86	45.9	25	0.714
sp|Q04637|IF4G1_HUMAN	EIF4G1	Eukaryotic translation initiation factor 4 gamma 1	110.01	111.45	58	83	0.714
sp|Q9UEW8|STK39_HUMAN	STK39	STE20/SPS1-related proline-alanine-rich protein kinase	16.06	17.53	42	10	0.714
sp|Q8WX92|NELFB_HUMAN	NELFB	Negative elongation factor B	26.66	28.86	53.5	16	0.714
sp|P62937|PPIA_HUMAN	PPIA	Peptidyl-prolyl cis-trans isomerase A	33.29	35.45	83.6	154	0.714
sp|P68400|CSK21_HUMAN	CSNK2A1	Casein kinase II subunit alpha	25.33	30.1	55.2	20	0.715
sp|O00541|PESC_HUMAN	PES1	Pescadillo homolog	42.01	44.67	55.1	28	0.715
sp|Q14690|RRP5_HUMAN	PDCD11	Protein RRP5 homolog	133.85	135.13	59	82	0.716
sp|Q9Y2R4|DDX52_HUMAN	DDX52	Probable ATP-dependent RNA helicase DDX52	36.29	40.27	64.1	28	0.716
sp|Q9UKY7|CDV3_HUMAN	CDV3	Protein CDV3 homolog	28.41	28.62	77.1	24	0.716
sp|Q96EU7|C1GLC_HUMAN	C1GALT1C1	C1GALT1-specific chaperone 1	2.22	2.51	31.1	3	0.716
sp|Q86U42|PABP2_HUMAN	PABPN1	Polyadenylate-binding protein 2	20.68	20.78	74.2	23	0.717
sp|Q9NUT2|ABCB8_HUMAN	ABCB8	ATP-binding cassette sub-family B member 8, mitochondrial	9.49	9.7	27.8	10	0.717
sp|O43760|SNG2_HUMAN	SYNGR2	Synaptogyrin-2	6.04	6.14	24.6	6	0.717
sp|P32322|P5CR1_HUMAN	PYCR1	Pyrroline-5-carboxylate reductase 1, mitochondrial	18.04	23.71	73	30	0.717
sp|O60287|NPA1P_HUMAN	URB1	Nucleolar pre-ribosomal-associated protein 1	40.84	43.91	30.7	26	0.717
sp|Q15599|NHRF2_HUMAN	SLC9A3R2	Na(+)/H(+) exchange regulatory cofactor NHE-RF2	10.73	10.96	27.6	7	0.717
sp|Q8NBN3|TM87A_HUMAN	TMEM87A	Transmembrane protein 87A	6.25	6.28	28.3	5	0.717

sp|Q96HS1|PGAM5_HUMAN	PGAM5	Serine/threonine-protein phosphatase PGAM5, mitochondrial	29.52	30.14	74.1	26	0.718
sp|Q96S59|RANB9_HUMAN	RANBP9	Ran-binding protein 9	22.39	22.54	29.4	13	0.718
sp|Q7Z6I8|CE024_HUMAN	C5orf24	UPF0461 protein C5orf24	7.38	7.56	38.3	4	0.718
sp|Q9BQT8|ODC_HUMAN	SLC25A21	Mitochondrial 2-oxodicarboxylate carrier	1.42	1.56	21.4	2	0.718
sp|Q9ULV4|COR1C_HUMAN	CORO1C	Coronin-1C	44.78	46.74	62.9	40	0.718
sp|P00491|PNPH_HUMAN	PNP	Purine nucleoside phosphorylase	39.43	39.48	92	29	0.718
sp|P40855|PEX19_HUMAN	PEX19	Peroxisomal biogenesis factor 19	16.29	16.35	53.9	12	0.718
sp|Q07812|BAX_HUMAN	BAX	Apoptosis regulator BAX	13.67	14.27	49.5	10	0.719
sp|Q9H1A7|RPB1C_HUMAN	POLR2J3	DNA-directed RNA polymerase II subunit RPB11-b2	3.03	3.1	50.4	5	0.719
sp|Q9Y244|POMP_HUMAN	POMP	Proteasome maturation protein	4.32	4.38	46.1	3	0.719
sp|P49406|RM19_HUMAN	MRPL19	39S ribosomal protein L19, mitochondrial	18.02	20.33	55.5	13	0.719
sp|P07900|HS90A_HUMAN	HSP90AA1	Heat shock protein HSP 90-alpha	97.99	167.29	82.1	325	0.720
sp|Q86X10|RLGPB_HUMAN	RALGAPB	Ral GTPase-activating protein subunit beta	18.04	18.28	24.7	10	0.720
sp|P11142|HSP7C_HUMAN	HSPA8	Heat shock cognate 71 kDa protein	104.05	117.74	93.2	266	0.720
sp|Q9GZU8|F192A_HUMAN	FAM192A	Protein FAM192A	10.14	12.76	55.1	9	0.720
sp|Q14678|KANK1_HUMAN	KANK1	KN motif and ankyrin repeat domain-containing protein 1	15.25	16.15	20.3	12	0.720
sp|O75381|PEX14_HUMAN	PEX14	Peroxisomal membrane protein PEX14	9.82	9.89	27.1	6	0.720
sp|Q9H0C8|ILKAP_HUMAN	ILKAP	Integrin-linked kinase-associated serine/threonine phosphatase 2C	27.47	27.66	52.6	15	0.720
sp|P17028|ZNF24_HUMAN	ZNF24	Zinc finger protein 24	1.75	2.13	29.9	2	0.722
sp|P27361|MK03_HUMAN	MAPK3	Mitogen-activated protein kinase 3	14.45	23.9	69.9	16	0.723
sp|Q08431|MFGM_HUMAN	MFGE8	Lactadherin	4.57	4.7	20.4	3	0.723
sp|P05386|RLA1_HUMAN	RPLP1	60S acidic ribosomal protein P1	12.21	15.7	94.7	130	0.723
sp|P48556|PSMD8_HUMAN	PSMD8	26S proteasome non-ATPase regulatory subunit 8	29.54	29.74	67.7	22	0.723
sp|Q8TCA0|LRC20_HUMAN	LRRC20	Leucine-rich repeat-containing protein 20	2.36	2.38	26.1	2	0.724
sp|O14732|IMPA2_HUMAN	IMPA2	Inositol monophosphatase 2	4.26	4.44	19.8	3	0.724
sp|P36957|ODO2_HUMAN	DLST	Dihydrolipoyllysine-residue succinyltransferase component of 2-oxoglutarate dehydrogenase complex	35.88	36.04	54.5	31	0.724
sp|P51808|DYLT3_HUMAN	DYNLT3	Dynein light chain Tctex-type 3	2.67	2.8	23.3	2	0.724
sp|Q9C0D5|TANC1_HUMAN	TANC1	Protein TANC1	2.47	4.4	9.8	5	0.725
sp|Q13242|SRSF9_HUMAN	SRSF9	Serine/arginine-rich splicing factor 9	18.48	21.24	56.1	13	0.725
sp|Q5JTH9|RRP12_HUMAN	RRP12	RRP12-like protein	56.9	60.28	45.3	41	0.725
sp|Q5VZK9|LR16A_HUMAN	LRRC16A	Leucine-rich repeat-containing protein 16A	9.88	10.61	15.9	8	0.725
sp|O95619|YETS4_HUMAN	YEATS4	YEATS domain-containing protein 4	4.07	4.65	41.9	5	0.725
sp|Q9H0U3|MAGT1_HUMAN	MAGT1	Magnesium transporter protein 1	10.25	10.37	27.8	8	0.725
sp|Q9NPH2|INO1_HUMAN	ISYNA1	Inositol-3-phosphate synthase 1	20.95	21.13	40.5	13	0.725
sp|Q12797|ASPH_HUMAN	ASPH	Aspartyl/asparaginyl beta-hydroxylase	48.88	49.35	48.6	35	0.726
sp|Q9BVA0|KTNB1_HUMAN	KATNB1	Katanin p80 WD40 repeat-containing subunit B1	11.08	11.94	30.1	8	0.726
sp|Q6ZVM7|TM1L2_HUMAN	TOM1L2	TOM1-like protein 2	5.25	6.56	30.6	5	0.726
sp|Q86X55|CARM1_HUMAN	CARM1	Histone-arginine methyltransferase CARM1	28.79	29.13	52.1	16	0.726
sp|P10620|MGST1_HUMAN	MGST1	Microsomal glutathione S-transferase 1	11.38	11.5	58.7	11	0.726
sp|P16444|DPEP1_HUMAN	DPEP1	Dipeptidase 1	7.32	7.49	20.9	4	0.726
sp|P22234|PUR6_HUMAN	PAICS	Multifunctional protein ADE2	60.85	62.45	82.1	62	0.726
sp|P55884|EIF3B_HUMAN	EIF3B	Eukaryotic translation initiation factor 3 subunit B	78.28	80.56	67.3	67	0.727
sp|P23258|TBG1_HUMAN	TUBG1	Tubulin gamma-1 chain	36.5	36.55	66.3	22	0.727
sp|Q92544|TM9S4_HUMAN	TM9SF4	Transmembrane 9 superfamily member 4	17.37	17.66	26	12	0.727
sp|P14174|MIF_HUMAN	MIF	Macrophage migration inhibitory factor	12.35	12.55	87.8	61	0.728
sp|Q8N999|CL029_HUMAN	C12orf29	Uncharacterized protein C12orf29	4.75	5.05	26.2	4	0.728
sp|Q9BRK0|REEP2_HUMAN	REEP2	Receptor expression-enhancing protein 2	2	2.01	21.8	2	0.728
sp|Q07157|ZO1_HUMAN	TJP1	Tight junction protein ZO-1	49.86	51.07	36.7	30	0.728
sp|P36969|GPX4_HUMAN	GPX4	Phospholipid hydroperoxide glutathione peroxidase, mitochondrial	3.18	3.67	52.3	3	0.728

sp|Q96GD4|AURKB_HUMAN	AURKB	Aurora kinase B	14.97	15.18	56.1	10	0.729
sp|Q86Y37|CACL1_HUMAN	CACUL1	CDK2-associated and cullin domain-containing protein 1	5.1	5.17	26	3	0.729
sp|Q6NSJ5|LRC8E_HUMAN	LRRC8E	Volume-regulated anion channel subunit LRRC8E	10.08	10.12	22	5	0.729
sp|Q9BRA2|TXD17_HUMAN	TXNDC17	Thioredoxin domain-containing protein 17	14.08	14.09	87	10	0.729
sp|O94880|PHF14_HUMAN	PHF14	PHD finger protein 14	12.91	13.08	18.4	9	0.729
sp|Q9H4Z3|PCIF1_HUMAN	PCIF1	Phosphorylated CTD-interacting factor 1	7.71	8.01	20.9	7	0.729
sp|Q12769|NU160_HUMAN	NUP160	Nuclear pore complex protein Nup160	63.21	65.44	47.6	44	0.729
sp|Q9BS16|CENPK_HUMAN	CENPK	Centromere protein K	1.68	1.79	24.5	2	0.730
sp|P56589|PEX3_HUMAN	PEX3	Peroxisomal biogenesis factor 3	8.55	8.96	25.5	5	0.730
sp|O95490|AGRL2_HUMAN	ADGRL2	Adhesion G protein-coupled receptor L2	12.83	13.22	18.2	8	0.730
sp|Q15427|SF3B4_HUMAN	SF3B4	Splicing factor 3B subunit 4	11.73	11.83	28.5	12	0.730
sp|Q14240|IF4A2_HUMAN	EIF4A2	Eukaryotic initiation factor 4A-II	9.78	39.88	68.6	52	0.730
sp|Q6UWW8|EST3_HUMAN	CES3	Carboxylesterase 3	2.71	2.78	14.5	3	0.730
sp|P61221|ABCE1_HUMAN	ABCE1	ATP-binding cassette sub-family E member 1	55.97	57.78	67.5	47	0.730
sp|Q9H3Z4|DNJC5_HUMAN	DNAJC5	DnaJ homolog subfamily C member 5	4.12	4.3	33.8	3	0.731
sp|O14639|ABLM1_HUMAN	ABLIM1	Actin-binding LIM protein 1	7.59	7.95	24.3	7	0.731
sp|P53004|BIEA_HUMAN	BLVRA	Biliverdin reductase A	26.22	26.9	64.9	19	0.731
sp|Q92845|KIFA3_HUMAN	KIFAP3	Kinesin-associated protein 3	4.1	6.55	22.4	5	0.731
sp|P46736|BRCC3_HUMAN	BRCC3	Lys-63-specific deubiquitinase BRCC36	6.99	7.09	32.6	8	0.731
sp|P35610|SOAT1_HUMAN	SOAT1	Sterol O-acyltransferase 1	12.09	12.59	26.6	8	0.731
sp|Q96EY4|TMA16_HUMAN	TMA16	Translation machinery-associated protein 16	8.49	10.41	40.9	11	0.731
sp|O60941|DTNB_HUMAN	DTNB	Dystrobrevin beta	4.45	11.08	28.1	9	0.731
sp|Q15022|SUZ12_HUMAN	SUZ12	Polycomb protein SUZ12	25.38	26.81	39.5	16	0.731
sp|Q8N1I0|DOCK4_HUMAN	DOCK4	Dedicator of cytokinesis protein 4	11.79	14.4	19.3	13	0.732
sp|Q9NZ01|TECR_HUMAN	TECR	Very-long-chain enoyl-CoA reductase	14.86	16.45	33.4	14	0.732
sp|O00303|EIF3F_HUMAN	EIF3F	Eukaryotic translation initiation factor 3 subunit F	22.25	22.27	47.6	20	0.732
sp|P60468|SC61B_HUMAN	SEC61B	Protein transport protein Sec61 subunit beta	6	6	50	5	0.732
sp|Q8TCE6|FA45A_HUMAN	FAM45A	Protein FAM45A	13.82	13.91	33.3	7	0.732
sp|Q9NZI8|IF2B1_HUMAN	IGF2BP1	Insulin-like growth factor 2 mRNA-binding protein 1	59.95	61.48	64	66	0.732
sp|Q9GZR7|DDX24_HUMAN	DDX24	ATP-dependent RNA helicase DDX24	46.78	48.99	55.2	32	0.733
sp|P01111|RASN_HUMAN	NRAS	GTPase NRas	18.26	20.33	63	20	0.733
sp|P31943|HNRH1_HUMAN	HNRNPH1	Heterogeneous nuclear ribonucleoprotein H	48.01	49.11	67.5	112	0.733
sp|Q12999|TSN31_HUMAN	TSPAN31	Tetraspanin-31	1.83	2.01	13.3	3	0.734
sp|Q9UNY4|TTF2_HUMAN	TTF2	Transcription termination factor 2	20.81	21.36	28.1	11	0.734
sp|Q8NCA5|FA98A_HUMAN	FAM98A	Protein FAM98A	20.4	27.44	48.4	18	0.734
sp|P17480|UBF1_HUMAN	UBTF	Nucleolar transcription factor 1	47.94	48.49	48.3	32	0.734
sp|P18124|RL7_HUMAN	RPL7	60S ribosomal protein L7	43.49	45.08	73.4	42	0.734
sp|Q96FN4|CPNE2_HUMAN	CPNE2	Copine-2	10.09	12.81	26.6	10	0.734
sp|O75534|CSDE1_HUMAN	CSDE1	Cold shock domain-containing protein E1	72.2	72.86	61.5	42	0.735
sp|Q9GZY4|COA1_HUMAN	COA1	Cytochrome c oxidase assembly factor 1 homolog	7.41	7.48	52.1	4	0.735
sp|Q13459|MYO9B_HUMAN	MYO9B	Unconventional myosin-IXb	8.25	10.91	18.5	7	0.735
sp|Q9NVR2|INT10_HUMAN	INTS10	Integrator complex subunit 10	19.83	20.16	32.8	11	0.735
sp|P28330|ACADL_HUMAN	ACADL	Long-chain specific acyl-CoA dehydrogenase, mitochondrial	2	2.36	14	2	0.735
sp|Q96N64|PWP2A_HUMAN	PWWP2A	PWWP domain-containing protein 2A	2.76	2.84	8.9	2	0.735
sp|Q92871|PMM1_HUMAN	PMM1	Phosphomannomutase 1	2.61	6.35	38.9	5	0.736
sp|Q8TDZ2|MICA1_HUMAN	MICAL1	Protein-methionine sulfoxide oxidase MICAL1	6.58	6.67	17.8	4	0.736
sp|Q96S82|UBL7_HUMAN	UBL7	Ubiquitin-like protein 7	2.78	2.85	14	2	0.736
sp|O43617|TPPC3_HUMAN	TRAPPC3	Trafficking protein particle complex subunit 3	12.47	12.63	46.7	7	0.737
sp|P19525|E2AK2_HUMAN	EIF2AK2	Interferon-induced, double-stranded RNA-activated protein kinase	31.32	32.4	43.4	19	0.737

sp|O95470|SGPL1_HUMAN	SGPL1	Sphingosine-1-phosphate lyase 1	27.72	27.9	39.4	16	0.737
sp|Q9NRX1|PNO1_HUMAN	PNO1	RNA-binding protein PNO1	22.32	22.42	63.9	12	0.737
sp|Q86V88|MGDP1_HUMAN	MDP1	Magnesium-dependent phosphatase 1	11.69	11.81	61.4	6	0.737
sp|O00748|EST2_HUMAN	CES2	Cocaine esterase	11.23	11.48	29.9	10	0.738
sp|P30048|PRDX3_HUMAN	PRDX3	Thioredoxin-dependent peroxide reductase, mitochondrial	26.29	26.37	66	40	0.738
sp|P40227|TCPZ_HUMAN	CCT6A	T-complex protein 1 subunit zeta	79.38	83.04	76.1	95	0.738
sp|Q9UJY4|GGA2_HUMAN	GGA2	ADP-ribosylation factor-binding protein GGA2	21.85	23.6	50.6	17	0.738
sp|Q9BVK6|TMED9_HUMAN	TMED9	Transmembrane emp24 domain-containing protein 9	21.33	22.55	51.5	17	0.738
sp|Q8N4H5|TOM5_HUMAN	TOMM5	Mitochondrial import receptor subunit TOM5 homolog	5.05	5.19	43.1	3	0.738
sp|P17931|LEG3_HUMAN	LGALS3	Galectin-3	8.15	9.16	46.8	8	0.739
sp|Q562R1|ACTBL_HUMAN	ACTBL2	Beta-actin-like protein 2	4.32	28.95	76.1	105	0.739
sp|P15927|RFA2_HUMAN	RPA2	Replication protein A 32 kDa subunit	19.79	20.21	71.1	30	0.739
sp|Q9NPQ8|RIC8A_HUMAN	RIC8A	Synembryn-A	26.09	26.37	49	16	0.739
sp|O43896|KIF1C_HUMAN	KIF1C	Kinesin-like protein KIF1C	12.85	21.51	22.9	11	0.739
sp|Q8IU81|I2BP1_HUMAN	IRF2BP1	Interferon regulatory factor 2-binding protein 1	15.95	16.31	39	11	0.739
sp|P22307|NLTP_HUMAN	SCP2	Non-specific lipid-transfer protein	42.68	45.34	60.3	39	0.740
sp|Q9BUT9|F195A_HUMAN	FAM195A	Protein FAM195A	4.03	4.23	42.5	3	0.740
sp|P35658|NU214_HUMAN	NUP214	Nuclear pore complex protein Nup214	67.72	70.59	39.3	44	0.740
sp|P62633|CNBP_HUMAN	CNBP	Cellular nucleic acid-binding protein	11.43	11.65	63.3	16	0.740
sp|O75600|KBL_HUMAN	GCAT	2-amino-3-ketobutyrate coenzyme A ligase, mitochondrial	12.36	12.84	52.5	11	0.740
sp|P30876|RPB2_HUMAN	POLR2B	DNA-directed RNA polymerase II subunit RPB2	71.54	71.79	55.7	43	0.741
sp|P62158|CALM_HUMAN	CALM1	Calmodulin	30.65	30.78	99.3	77	0.741
sp|Q5T2R2|DPS1_HUMAN	PDSS1	Decaprenyl-diphosphate synthase subunit 1	3.66	3.76	16.6	2	0.741
sp|P46778|RL21_HUMAN	RPL21	60S ribosomal protein L21	14.14	15.82	60	26	0.742
sp|Q99622|C10_HUMAN	C12orf57	Protein C10	8.13	8.27	63.5	8	0.742
sp|O15360|FANCA_HUMAN	FANCA	Fanconi anemia group A protein	6.01	6.11	12.4	4	0.743
sp|P07814|SYEP_HUMAN	EPRS	Bifunctional glutamate/proline--tRNA ligase	188.44	189.68	76.9	146	0.743
sp|Q9HC38|GLOD4_HUMAN	GLOD4	Glyoxalase domain-containing protein 4	28.73	28.84	66.1	16	0.743
sp|Q6NXT4|ZNT6_HUMAN	SLC30A6	Zinc transporter 6	2.35	2.38	17.8	2	0.743
sp|O75525|KHDR3_HUMAN	KHDRBS3	KH domain-containing, RNA-binding, signal transduction-associated protein 3	5.23	9.92	29.2	10	0.744
sp|P78316|NOP14_HUMAN	NOP14	Nucleolar protein 14	46.38	48.55	41.1	28	0.744
sp|Q96BW5|PTER_HUMAN	PTER	Phosphotriesterase-related protein	4.5	4.57	31.2	3	0.744
sp|Q96BK5|PINX1_HUMAN	PINX1	PIN2/TERF1-interacting telomerase inhibitor 1	5.9	5.98	31.1	3	0.744
sp|Q14185|DOCK1_HUMAN	DOCK1	Dedicator of cytokinesis protein 1	8.25	11.1	20.2	11	0.744
sp|Q9UGK3|STAP2_HUMAN	STAP2	Signal-transducing adaptor protein 2	1.35	1.68	24.8	2	0.744
sp|P18827|SDC1_HUMAN	SDC1	Syndecan-1	5.17	5.24	20.7	3	0.744
sp|Q9H3Q1|BORG4_HUMAN	CDC42EP4	Cdc42 effector protein 4	6.45	6.5	38.5	3	0.745
sp|Q6GYQ0|RGPA1_HUMAN	RALGAPA1	Ral GTPase-activating protein subunit alpha-1	17.13	18.36	20.8	15	0.745
sp|Q9H0D6|XRN2_HUMAN	XRN2	5'-3' exoribonuclease 2	85.72	85.91	67.6	53	0.745
sp|P14866|HNRPL_HUMAN	HNRNPL	Heterogeneous nuclear ribonucleoprotein L	67.14	67.76	72.7	113	0.745
sp|O60942|MCE1_HUMAN	RNGTT	mRNA-capping enzyme	15.47	15.81	35.9	9	0.745
sp|Q9HD33|RM47_HUMAN	MRPL47	39S ribosomal protein L47, mitochondrial	18.06	18.15	48.4	9	0.745
sp|P25440|BRD2_HUMAN	BRD2	Bromodomain-containing protein 2	15.48	20.86	34.1	13	0.745
sp|Q96FX2|DPH3_HUMAN	DPH3	DPH3 homolog	2	2.01	36.6	2	0.745
sp|O95684|FR1OP_HUMAN	FGFR1OP	FGFR1 oncogene partner	16.17	17.29	45.9	11	0.745
sp|Q8WUM9|S20A1_HUMAN	SLC20A1	Sodium-dependent phosphate transporter 1	7.43	8.33	19.3	5	0.745
sp|Q969E8|TSR2_HUMAN	TSR2	Pre-rRNA-processing protein TSR2 homolog	3.77	3.86	19.4	2	0.746
sp|Q96FV9|THOC1_HUMAN	THOC1	THO complex subunit 1	32.03	33.05	49.3	22	0.746
sp|P56378|68MP_HUMAN	MP68	6.8 kDa mitochondrial proteolipid	2.62	3.16	39.7	2	0.746

sp|Q6P2Q9|PRP8_HUMAN	PRPF8	Pre-mRNA-processing-splicing factor 8	188.83	188.73	61.6	140	0.746
sp|Q8NBK3|SUMF1_HUMAN	SUMF1	Sulfatase-modifying factor 1	4	4	15	2	0.747
sp|Q9H553|ALG2_HUMAN	ALG2	Alpha-1,3/1,6-mannosyltransferase ALG2	15.91	16.84	36.3	10	0.747
sp|O94813|SLIT2_HUMAN	SLIT2	Slit homolog 2 protein	12.12	12.25	16	6	0.747
sp|P51809|VAMP7_HUMAN	VAMP7	Vesicle-associated membrane protein 7	11.36	12.79	41.4	7	0.747
sp|Q13620|CUL4B_HUMAN	CUL4B	Cullin-4B	51.23	54.66	53.6	30	0.747
sp|P52565|GDIR1_HUMAN	ARHGDIA	Rho GDP-dissociation inhibitor 1	26.44	27.04	80.9	22	0.747
sp|O95218|ZRAB2_HUMAN	ZRANB2	Zinc finger Ran-binding domain-containing protein 2	10.61	10.85	36.7	7	0.748
sp|O15498|YKT6_HUMAN	YKT6	Synaptobrevin homolog YKT6	21.3	22.14	79.8	15	0.748
sp|Q9BX93|PG12B_HUMAN	PLA2G12B	Group XIIB secretory phospholipase A2-like protein	5.74	5.87	30.3	3	0.748
sp|Q9H074|PAIP1_HUMAN	PAIP1	Polyadenylate-binding protein-interacting protein 1	6.53	6.65	29.2	5	0.748
sp|P00167|CYB5_HUMAN	CYB5A	Cytochrome b5	9.68	10.17	63.4	7	0.748
sp|Q676U5|A16L1_HUMAN	ATG16L1	Autophagy-related protein 16-1	14.35	15.52	38.1	12	0.748
sp|Q5THK1|PR14L_HUMAN	PRR14L	Protein PRR14L	3.68	4.06	9.9	5	0.748
sp|O60879|DIAP2_HUMAN	DIAPH2	Protein diaphanous homolog 2	18.42	24.78	30.4	17	0.749
sp|Q9BUB7|TMM70_HUMAN	TMEM70	Transmembrane protein 70, mitochondrial	9.15	9.32	32.3	5	0.749
sp|P50150|GBG4_HUMAN	GNG4	Guanine nucleotide-binding protein G(I)/G(S)/G(O) subunit gamma-4	4	4.01	53.3	2	0.749
sp|Q15475|SIX1_HUMAN	SIX1	Homeobox protein SIX1	4	4.01	13	2	0.749
sp|Q5NDL2|EOGT_HUMAN	EOGT	EGF domain-specific O-linked N-acetylglucosamine transferase	6.02	6.21	26.4	5	0.749
sp|Q96F10|SAT2_HUMAN	SAT2	Diamine acetyltransferase 2	4.03	4.79	45.9	3	0.749
sp|O60306|AQR_HUMAN	AQR	Intron-binding protein aquarius	62.63	64.13	40	35	0.749
sp|Q7Z4V5|HDGR2_HUMAN	HDGFRP2	Hepatoma-derived growth factor-related protein 2	10.36	12.98	23.1	10	0.749
sp|Q9UMS0|NFU1_HUMAN	NFU1	NFU1 iron-sulfur cluster scaffold homolog, mitochondrial	9.66	10.02	39.4	6	0.750
sp|Q9BTX1|NDC1_HUMAN	NDC1	Nucleoporin NDC1	24.95	26.08	35.6	16	0.750
sp|Q9UKB1|FBW1B_HUMAN	FBXW11	F-box/WD repeat-containing protein 11	20.58	22.15	46.9	11	0.750
sp|Q96KB5|TOPK_HUMAN	PBK	Lymphokine-activated killer T-cell-originated protein kinase	16.26	17.37	55.6	9	0.750
sp|Q15370|ELOB_HUMAN	TCEB2	Transcription elongation factor B polypeptide 2	18.4	18.52	94.1	18	0.750
sp|O94760|DDAH1_HUMAN	DDAH1	N(G),N(G)-dimethylarginine dimethylaminohydrolase 1	20.84	22.16	59.3	13	0.751
sp|P30307|MPIP3_HUMAN	CDC25C	M-phase inducer phosphatase 3	2	2.31	16.7	2	0.751
sp|Q9BTC8|MTA3_HUMAN	MTA3	Metastasis-associated protein MTA3	9.97	20.86	36.4	15	0.751
sp|Q6PKC3|TXD11_HUMAN	TXNDC11	Thioredoxin domain-containing protein 11	3.81	4.11	12.8	5	0.751
sp|Q8N6C5|IGSF1_HUMAN	IGSF1	Immunoglobulin superfamily member 1	12.52	12.87	14.1	9	0.751
sp|Q9NP92|RT30_HUMAN	MRPS30	28S ribosomal protein S30, mitochondrial	20.78	20.88	57.6	14	0.751
sp|Q9H0P0|5NT3A_HUMAN	NT5C3A	Cytosolic 5'-nucleotidase 3A	16.24	16.33	53	8	0.751
sp|Q16531|DDB1_HUMAN	DDB1	DNA damage-binding protein 1	111.23	111.73	58.6	74	0.751
sp|P62269|RS18_HUMAN	RPS18	40S ribosomal protein S18	23.79	25.36	63.2	19	0.752
sp|P61006|RAB8A_HUMAN	RAB8A	Ras-related protein Rab-8A	24.3	32.48	80.2	22	0.752
sp|Q7L5D6|GET4_HUMAN	GET4	Golgi to ER traffic protein 4 homolog	12.25	12.57	39.5	7	0.752
sp|O95785|WIZ_HUMAN	WIZ	Protein Wiz	20.92	22.44	26	18	0.752
sp|P62314|SMD1_HUMAN	SNRPD1	Small nuclear ribonucleoprotein Sm D1	12.61	12.65	54.6	14	0.752
sp|P49368|TCPG_HUMAN	CCT3	T-complex protein 1 subunit gamma	95.64	96.18	79.5	111	0.752
sp|Q13033|STRN3_HUMAN	STRN3	Striatin-3	22.96	25.48	42.7	21	0.752
sp|Q15019|SEPT2_HUMAN	SEPT2	Septin-2	26.2	28.34	75.9	37	0.752
sp|Q7L9B9|EEPD1_HUMAN	EEPD1	Endonuclease/exonuclease/phosphatase family domain-containing protein 1	6.59	6.76	23.2	5	0.752
sp|Q14781|CBX2_HUMAN	CBX2	Chromobox protein homolog 2	8	8.14	29.9	5	0.752
sp|Q9HD34|LYRM4_HUMAN	LYRM4	LYR motif-containing protein 4	4	4.02	44	3	0.752
sp|P40425|PBX2_HUMAN	PBX2	Pre-B-cell leukemia transcription factor 2	8.76	9.07	27.4	7	0.753
sp|P55786|PSA_HUMAN	NPEPPS	Puromycin-sensitive aminopeptidase	72.23	73.37	63.6	59	0.753
sp|Q9BTT6|LRRC1_HUMAN	LRRC1	Leucine-rich repeat-containing protein 1	16.79	23.25	49.6	13	0.753

sp|Q15050|RRS1_HUMAN	RRS1	Ribosome biogenesis regulatory protein homolog	28.27	28.97	52.3	20	0.753
sp|P49748|ACADV_HUMAN	ACADVL	Very long-chain specific acyl-CoA dehydrogenase, mitochondrial	60.83	61.02	66.9	42	0.754
sp|P62244|RS15A_HUMAN	RPS15A	40S ribosomal protein S15a	24.44	25.52	81.5	31	0.754
sp|Q13017|RHG05_HUMAN	ARHGAP5	Rho GTPase-activating protein 5	22.6	24.09	31.9	16	0.754
sp|Q7L3T8|SYPM_HUMAN	PARS2	Probable proline--tRNA ligase, mitochondrial	14.28	14.46	40.6	7	0.754
sp|O00399|DCTN6_HUMAN	DCTN6	Dynactin subunit 6	4.3	4.33	35.3	2	0.754
sp|Q14192|FHL2_HUMAN	FHL2	Four and a half LIM domains protein 2	4.43	4.54	35.5	3	0.755
sp|Q6P587|FAHD1_HUMAN	FAHD1	Acylpyruvase FAHD1, mitochondrial	14.96	15.02	75.5	12	0.755
sp|Q6ZS17|FA65A_HUMAN	FAM65A	Protein FAM65A	2.49	2.64	13	4	0.755
sp|Q15631|TSN_HUMAN	TSN	Translin	15.13	15.34	51.8	11	0.755
sp|Q5HYK3|COQ5_HUMAN	COQ5	2-methoxy-6-polyprenyl-1,4-benzoquinol methylase, mitochondrial	13.5	13.55	37.6	8	0.756
sp|P03886|NU1M_HUMAN	MT-ND1	NADH-ubiquinone oxidoreductase chain 1	4	4	11	2	0.756
sp|P60602|ROMO1_HUMAN	ROMO1	Reactive oxygen species modulator 1	4.93	4.99	50.6	7	0.756
sp|P52292|IMA1_HUMAN	KPNA2	Importin subunit alpha-1	56.06	58.67	73.9	60	0.756
sp|Q10471|GALT2_HUMAN	GALNT2	Polypeptide N-acetylgalactosaminyltransferase 2	40.9	40.94	63.8	24	0.756
sp|Q15773|MLF2_HUMAN	MLF2	Myeloid leukemia factor 2	4.33	4.43	41.9	3	0.756
sp|Q8N1F8|S11IP_HUMAN	STK11IP	Serine/threonine-protein kinase 11-interacting protein	17.58	18.45	24.7	11	0.756
sp|Q6DN90|IQEC1_HUMAN	IQSEC1	IQ motif and SEC7 domain-containing protein 1	15.19	15.55	23.7	9	0.756
sp|O00584|RNT2_HUMAN	RNASET2	Ribonuclease T2	8.3	8.6	49.2	9	0.756
sp|P62266|RS23_HUMAN	RPS23	40S ribosomal protein S23	15.81	18.33	72	22	0.757
sp|Q15418|KS6A1_HUMAN	RPS6KA1	Ribosomal protein S6 kinase alpha-1	25.99	45.46	55.9	30	0.757
sp|Q96P48|ARAP1_HUMAN	ARAP1	Arf-GAP with Rho-GAP domain, ANK repeat and PH domain-containing protein 1	37.85	39.22	32.1	19	0.757
sp|P45985|MP2K4_HUMAN	MAP2K4	Dual specificity mitogen-activated protein kinase kinase 4	12.7	12.76	39.9	11	0.757
sp|P68036|UB2L3_HUMAN	UBE2L3	Ubiquitin-conjugating enzyme E2 L3	18.04	19.16	77.3	24	0.757
sp|Q9HCU5|PREB_HUMAN	PREB	Prolactin regulatory element-binding protein	21.51	21.76	58.8	14	0.757
sp|Q6N069|NAA16_HUMAN	NAA16	N-alpha-acetyltransferase 16, NatA auxiliary subunit	4.09	19.19	39.9	15	0.757
sp|O43633|CHM2A_HUMAN	CHMP2A	Charged multivesicular body protein 2a	9.71	10.27	48.7	5	0.757
sp|Q9Y5P6|GMPPB_HUMAN	GMPPB	Mannose-1-phosphate guanyltransferase beta	18.26	18.94	58.1	14	0.757
sp|P48739|PIPNB_HUMAN	PITPNB	Phosphatidylinositol transfer protein beta isoform	36.06	36.29	86.4	25	0.757
sp|Q13555|KCC2G_HUMAN	CAMK2G	Calcium/calmodulin-dependent protein kinase type II subunit gamma	4.21	13	33.9	11	0.758
sp|P53634|CATC_HUMAN	CTSC	Dipeptidyl peptidase 1	18.16	18.3	40.4	15	0.758
sp|Q12830|BPTF_HUMAN	BPTF	Nucleosome-remodeling factor subunit BPTF	40.88	45.68	22.7	24	0.758
sp|O95298|NDUC2_HUMAN	NDUFC2	NADH dehydrogenase [ubiquinone] 1 subunit C2	3.07	3.52	43.7	3	0.758
sp|Q96QD9|UIF_HUMAN	FYTTD1	UAP56-interacting factor	2.65	2.73	31.1	2	0.758
sp|Q8N5D0|WDTC1_HUMAN	WDTC1	WD and tetratricopeptide repeats protein 1	2.63	2.94	19.4	2	0.759
sp|Q9NPA5|ZF64A_HUMAN	ZFP64	Zinc finger protein 64 homolog, isoforms 1 and 2	2.03	2.09	8.5	2	0.759
sp|Q96ME7|ZN512_HUMAN	ZNF512	Zinc finger protein 512	20.68	21.94	41.8	15	0.759
sp|P14618|KPYM_HUMAN	PKM	Pyruvate kinase PKM	118.75	120.1	85.5	228	0.759
sp|O43464|HTRA2_HUMAN	HTRA2	Serine protease HTRA2, mitochondrial	19.78	19.84	46.9	14	0.759
sp|Q9Y5A6|ZSC21_HUMAN	ZSCAN21	Zinc finger and SCAN domain-containing protein 21	2	3.77	22.6	2	0.759
sp|Q6P4Q7|CNNM4_HUMAN	CNNM4	Metal transporter CNNM4	16.57	18.22	25.8	14	0.760
sp|Q5EBL8|PDZ11_HUMAN	PDZD11	PDZ domain-containing protein 11	4.93	5.91	70.7	5	0.760
sp|Q9UPQ3|AGAP1_HUMAN	AGAP1	Arf-GAP with GTPase, ANK repeat and PH domain-containing protein 1	10.07	12.53	17.9	8	0.760
sp|O43615|TIM44_HUMAN	TIMM44	Mitochondrial import inner membrane translocase subunit TIM44	44.09	44.21	59.1	28	0.760
sp|Q9UNW1|MINP1_HUMAN	MINPP1	Multiple inositol polyphosphate phosphatase 1	15.61	15.69	36.8	9	0.760
sp|P08865|RSSA_HUMAN	RPSA	40S ribosomal protein SA	36.33	38.32	65.4	69	0.760
sp|Q9H6F5|CCD86_HUMAN	CCDC86	Coiled-coil domain-containing protein 86	21.79	21.92	56.1	17	0.761
sp|Q8NAT1|PMGT2_HUMAN	POMGNT2	Protein O-linked-mannose beta-1,4-N-acetylglucosaminyltransferase 2	9.52	9.69	31	8	0.761
sp|O75155|CAND2_HUMAN	CAND2	Cullin-associated NEDD8-dissociated protein 2	12.91	17.78	22.9	16	0.761

sp|Q9HC21|TPC_HUMAN	SLC25A19	Mitochondrial thiamine pyrophosphate carrier	6.18	6.21	26.3	4	0.761
sp|Q15185|TEBP_HUMAN	PTGES3	Prostaglandin E synthase 3	14.03	14.09	71.3	13	0.761
sp|P49419|AL7A1_HUMAN	ALDH7A1	Alpha-aminoadipic semialdehyde dehydrogenase	56.07	56.1	77.6	52	0.761
sp|O43709|WBS22_HUMAN	WBSCR22	Probable 18S rRNA (guanine-N(7))-methyltransferase	5.9	6.18	27.1	4	0.761
sp|Q13283|G3BP1_HUMAN	G3BP1	Ras GTPase-activating protein-binding protein 1	37.41	38.91	62.5	41	0.762
sp|Q8WZA0|LZIC_HUMAN	LZIC	Protein LZIC	10.85	11.76	66.3	8	0.762
sp|O43681|ASNA_HUMAN	ASNA1	ATPase ASNA1	26.37	26.45	66.4	20	0.762
sp|Q5TBB1|RNH2B_HUMAN	RNASEH2B	Ribonuclease H2 subunit B	8.6	8.77	34	6	0.762
sp|Q9UNX4|WDR3_HUMAN	WDR3	WD repeat-containing protein 3	86.9	87.13	69.5	54	0.762
sp|Q9BVC6|TM109_HUMAN	TMEM109	Transmembrane protein 109	9.5	9.58	25.1	6	0.762
sp|Q9Y584|TIM22_HUMAN	TIMM22	Mitochondrial import inner membrane translocase subunit Tim22	2.34	2.37	21.1	2	0.762
sp|O15228|GNPAT_HUMAN	GNPAT	Dihydroxyacetone phosphate acyltransferase	37.75	38.82	48.1	22	0.763
sp|P61088|UBE2N_HUMAN	UBE2N	Ubiquitin-conjugating enzyme E2 N	22.4	22.63	82.2	20	0.763
sp|O60264|SMCA5_HUMAN	SMARCA5	SWI/SNF-related matrix-associated actin-dependent regulator of chromatin subfamily A member 5	96.68	98.8	58.1	64	0.763
sp|P10412|H14_HUMAN	HIST1H1E	Histone H1.4	43.24	44.73	84	79	0.764
sp|Q9NV31|IMP3_HUMAN	IMP3	U3 small nucleolar ribonucleoprotein protein IMP3	14.89	15.05	73.9	15	0.764
sp|Q9UH17|ABC3B_HUMAN	APOBEC3B	DNA dC->dU-editing enzyme APOBEC-3B	6.39	6.42	23.8	3	0.764
sp|P16402|H13_HUMAN	HIST1H1D	Histone H1.3	10.26	37.94	76	59	0.764
sp|P28070|PSB4_HUMAN	PSMB4	Proteasome subunit beta type-4	20.82	21.86	52.3	35	0.764
sp|Q12905|ILF2_HUMAN	ILF2	Interleukin enhancer-binding factor 2	44.7	44.92	78.7	73	0.764
sp|P63167|DYL1_HUMAN	DYNLL1	Dynein light chain 1, cytoplasmic	9.86	11.19	68.5	12	0.764
sp|Q7L4I2|RSRC2_HUMAN	RSRC2	Arginine/serine-rich coiled-coil protein 2	9.39	9.54	26.3	5	0.764
sp|Q16740|CLPP_HUMAN	CLPP	ATP-dependent Clp protease proteolytic subunit, mitochondrial	16.93	16.98	43.7	14	0.765
sp|O15446|RPA34_HUMAN	CD3EAP	DNA-directed RNA polymerase I subunit RPA34	22.47	22.58	59.2	13	0.765
sp|P53396|ACLY_HUMAN	ACLY	ATP-citrate synthase	113.98	114.99	65.2	91	0.765
sp|Q8IUI8|CRLF3_HUMAN	CRLF3	Cytokine receptor-like factor 3	9.52	9.59	26.2	6	0.765
sp|Q9Y6A5|TACC3_HUMAN	TACC3	Transforming acidic coiled-coil-containing protein 3	27.27	29.23	41.8	14	0.766
sp|Q8IXB1|DJC10_HUMAN	DNAJC10	DnaJ homolog subfamily C member 10	38.12	38.21	42.2	20	0.766
sp|P08397|HEM3_HUMAN	HMBS	Porphobilinogen deaminase	18.33	18.39	49.3	10	0.766
sp|Q14CB8|RHG19_HUMAN	ARHGAP19	Rho GTPase-activating protein 19	3.1	3.18	17.4	2	0.766
sp|O60841|IF2P_HUMAN	EIF5B	Eukaryotic translation initiation factor 5B	84.74	85.89	54.9	52	0.766
sp|P49006|MRP_HUMAN	MARCKSL1	MARCKS-related protein	15.71	15.75	52.3	12	0.766
sp|P54725|RD23A_HUMAN	RAD23A	UV excision repair protein RAD23 homolog A	13.51	21.27	49.9	16	0.766
sp|Q9UHB4|NDOR1_HUMAN	NDOR1	NADPH-dependent diflavin oxidoreductase 1	6	6	10.6	3	0.767
sp|Q9HBM1|SPC25_HUMAN	SPC25	Kinetochore protein Spc25	6.11	7.65	57.1	5	0.767
sp|Q9NWT6|HIF1N_HUMAN	HIF1AN	Hypoxia-inducible factor 1-alpha inhibitor	11.38	11.47	36.7	8	0.767
sp|P35637|FUS_HUMAN	FUS	RNA-binding protein FUS	25.81	26.76	32.9	39	0.767
sp|O75143|ATG13_HUMAN	ATG13	Autophagy-related protein 13	2.16	2.23	18.6	3	0.767
sp|P60953|CDC42_HUMAN	CDC42	Cell division control protein 42 homolog	20.11	20.14	72.3	18	0.768
sp|Q9NVZ3|NECP2_HUMAN	NECAP2	Adaptin ear-binding coat-associated protein 2	8.17	9.95	64.3	7	0.768
sp|Q9NTI5|PDS5B_HUMAN	PDS5B	Sister chromatid cohesion protein PDS5 homolog B	56.63	70.63	45.3	40	0.768
sp|P55082|MFAP3_HUMAN	MFAP3	Microfibril-associated glycoprotein 3	4.34	4.54	17.4	4	0.769
sp|Q68D91|MBLC2_HUMAN	MBLAC2	Metallo-beta-lactamase domain-containing protein 2	9.77	11.79	35.8	6	0.769
sp|P35080|PROF2_HUMAN	PFN2	Profilin-2	15.91	15.97	85.7	13	0.769
sp|P61457|PHS_HUMAN	PCBD1	Pterin-4-alpha-carbinolamine dehydratase	11.73	12.38	74	9	0.769
sp|Q96AQ6|PBIP1_HUMAN	PBXIP1	Pre-B-cell leukemia transcription factor-interacting protein 1	11.11	11.26	19.4	7	0.769
sp|P51795|CLCN5_HUMAN	CLCN5	H(+)/Cl(-) exchange transporter 5	2.46	3.9	20.4	4	0.769
sp|Q15393|SF3B3_HUMAN	SF3B3	Splicing factor 3B subunit 3	101.45	106.41	68.1	94	0.770
sp|Q9BY32|ITPA_HUMAN	ITPA	Inosine triphosphate pyrophosphatase	12.53	12.63	61.9	11	0.770

sp|O75170|PP6R2_HUMAN	PPP6R2	Serine/threonine-protein phosphatase 6 regulatory subunit 2	17.19	20.17	39.1	13	0.770
sp|Q9Y3F4|STRAP_HUMAN	STRAP	Serine-threonine kinase receptor-associated protein	32.15	32.2	74	26	0.770
sp|Q7L592|NDUF7_HUMAN	NDUFAF7	NADH dehydrogenase [ubiquinone] complex I, assembly factor 7	20.1	20.7	47.9	12	0.770
sp|Q9NQT8|KI13B_HUMAN	KIF13B	Kinesin-like protein KIF13B	24.2	28.05	30.7	15	0.771
sp|P00734|THRB_HUMAN	F2	Prothrombin	17.38	17.49	37.1	10	0.771
sp|Q96J84|KIRR1_HUMAN	KIRREL	Kin of IRRE-like protein 1	2.76	3.01	17.3	3	0.771
sp|Q96D09|GASP2_HUMAN	GPRASP2	G-protein coupled receptor-associated sorting protein 2	7.4	7.5	16.2	5	0.771
sp|P30519|HMOX2_HUMAN	HMOX2	Heme oxygenase 2	31.76	33.21	73.1	23	0.771
sp|Q8WUD4|CCD12_HUMAN	CCDC12	Coiled-coil domain-containing protein 12	11.13	11.51	71.1	6	0.771
sp|P60660|MYL6_HUMAN	MYL6	Myosin light polypeptide 6	19.28	19.79	88.1	24	0.771
sp|Q587I9|SFT2C_HUMAN	SFT2D3	Vesicle transport protein SFT2C	5.8	5.85	32.1	3	0.771
sp|Q00613|HSF1_HUMAN	HSF1	Heat shock factor protein 1	6.38	7.41	25	5	0.772
sp|Q7Z6V5|ADAT2_HUMAN	ADAT2	tRNA-specific adenosine deaminase 2	3.56	3.64	39.8	2	0.772
sp|Q9BVC5|ASHWN_HUMAN	C2orf49	Ashwin	6.38	6.53	48.3	4	0.772
sp|Q16539|MK14_HUMAN	MAPK14	Mitogen-activated protein kinase 14	23.06	23.22	58.3	15	0.772
sp|Q9Y4D7|PLXD1_HUMAN	PLXND1	Plexin-D1	3.37	3.56	12.5	3	0.772
sp|Q9Y3C1|NOP16_HUMAN	NOP16	Nucleolar protein 16	7.25	7.62	50.6	7	0.772
sp|Q5M775|CYTSB_HUMAN	SPECC1	Cytospin-B	4.37	7.1	22.4	5	0.773
sp|P53794|SC5A3_HUMAN	SLC5A3	Sodium/myo-inositol cotransporter	4.67	6.88	15.5	4	0.773
sp|P63000|RAC1_HUMAN	RAC1	Ras-related C3 botulinum toxin substrate 1	17.76	20.23	91.1	16	0.774
sp|Q9Y224|CN166_HUMAN	C14orf166	UPF0568 protein C14orf166	25.36	25.51	77.1	22	0.774
sp|Q9NY93|DDX56_HUMAN	DDX56	Probable ATP-dependent RNA helicase DDX56	31.11	33.94	59.4	24	0.774
sp|Q969F9|HPS3_HUMAN	HPS3	Hermansky-Pudlak syndrome 3 protein	2.76	3.45	14.4	3	0.775
sp|Q15428|SF3A2_HUMAN	SF3A2	Splicing factor 3A subunit 2	18.89	21.18	35.3	20	0.775
sp|Q06481|APLP2_HUMAN	APLP2	Amyloid-like protein 2	23.01	23.15	39.6	15	0.775
sp|Q92930|RAB8B_HUMAN	RAB8B	Ras-related protein Rab-8B	2	18.91	58.5	12	0.775
sp|P55072|TERA_HUMAN	VCP	Transitional endoplasmic reticulum ATPase	126.7	130.24	86.1	169	0.775
sp|Q9UDX5|MTFP1_HUMAN	MTFP1	Mitochondrial fission process protein 1	6.07	6.67	34.3	4	0.776
sp|Q9BTW9|TBCD_HUMAN	TBCD	Tubulin-specific chaperone D	50.55	50.74	47.3	28	0.776
sp|Q96I59|SYNM_HUMAN	NARS2	Probable asparagine--tRNA ligase, mitochondrial	12.71	13.06	50.1	8	0.776
sp|Q5T1C6|THEM4_HUMAN	THEM4	Acyl-coenzyme A thioesterase THEM4	2.26	2.29	22.1	2	0.776
sp|P25789|PSA4_HUMAN	PSMA4	Proteasome subunit alpha type-4	34.06	34.32	77.4	45	0.776
sp|Q06830|PRDX1_HUMAN	PRDX1	Peroxiredoxin-1	43.99	45.13	80.9	72	0.776
sp|P52434|RPAB3_HUMAN	POLR2H	DNA-directed RNA polymerases I, II, and III subunit RPABC3	11.29	11.86	51.3	11	0.776
sp|Q07065|CKAP4_HUMAN	CKAP4	Cytoskeleton-associated protein 4	74.22	76.03	72.1	65	0.776
sp|Q9H4M9|EHD1_HUMAN	EHD1	EH domain-containing protein 1	48.69	50.66	80.7	30	0.776
sp|Q76FK4|NOL8_HUMAN	NOL8	Nucleolar protein 8	23.22	23.51	34.6	13	0.776
sp|Q13155|AIMP2_HUMAN	AIMP2	Aminoacyl tRNA synthase complex-interacting multifunctional protein 2	20.49	20.9	64.4	14	0.776
sp|O95985|TOP3B_HUMAN	TOP3B	DNA topoisomerase 3-beta-1	3	5.32	22.2	5	0.777
sp|Q8NBX0|SCPDL_HUMAN	SCCPDH	Saccharopine dehydrogenase-like oxidoreductase	24.41	24.47	61.3	19	0.777
sp|Q9H6U6|BCAS3_HUMAN	BCAS3	Breast carcinoma-amplified sequence 3	5.74	5.83	10.5	4	0.777
sp|P20042|IF2B_HUMAN	EIF2S2	Eukaryotic translation initiation factor 2 subunit 2	40.46	40.52	82.9	25	0.777
sp|Q12965|MYO1E_HUMAN	MYO1E	Unconventional myosin-Ie	18.42	19.25	39	12	0.778
sp|Q8TAM2|TTC8_HUMAN	TTC8	Tetratricopeptide repeat protein 8	2.01	2.07	12.9	2	0.778
sp|Q8NE71|ABCF1_HUMAN	ABCF1	ATP-binding cassette sub-family F member 1	64.59	68.33	65.8	50	0.778
sp|P50225|ST1A1_HUMAN	SULT1A1	Sulfotransferase 1A1	35.9	35.98	87.1	25	0.778
sp|Q9HD42|CHM1A_HUMAN	CHMP1A	Charged multivesicular body protein 1a	7.58	7.78	41.3	5	0.778
sp|P36873|PP1G_HUMAN	PPP1CC	Serine/threonine-protein phosphatase PP1-gamma catalytic subunit	5.53	38.66	79.9	39	0.778
sp|Q6PL18|ATAD2_HUMAN	ATAD2	ATPase family AAA domain-containing protein 2	31.28	32.08	33.8	18	0.778

sp|Q86V81|THOC4_HUMAN	ALYREF	THO complex subunit 4	20.89	21.21	65.8	33	0.778
sp|P62330|ARF6_HUMAN	ARF6	ADP-ribosylation factor 6	14.89	17.08	77.7	11	0.778
sp|P55263|ADK_HUMAN	ADK	Adenosine kinase	28.95	29.2	67.7	18	0.778
sp|Q01844|EWS_HUMAN	EWSR1	RNA-binding protein EWS	16.54	16.69	32.9	28	0.778
sp|Q9UKR5|ERG28_HUMAN	C14orf1	Probable ergosterol biosynthetic protein 28	4	4	24.3	2	0.778
sp|Q9NSY1|BMP2K_HUMAN	BMP2K	BMP-2-inducible protein kinase	6.27	6.35	14.6	4	0.778
sp|Q92504|S39A7_HUMAN	SLC39A7	Zinc transporter SLC39A7	4.02	4.02	19.8	3	0.779
sp|P23434|GCSH_HUMAN	GCSH	Glycine cleavage system H protein, mitochondrial	15.39	15.49	68.2	15	0.779
sp|Q6IQ49|SDE2_HUMAN	SDE2	Protein SDE2 homolog	8.21	9.17	36.8	7	0.779
sp|Q92552|RT27_HUMAN	MRPS27	28S ribosomal protein S27, mitochondrial	33.33	33.48	62.6	21	0.779
sp|Q8IVH4|MMAA_HUMAN	MMAA	Methylmalonic aciduria type A protein, mitochondrial	4.82	4.91	30.1	3	0.779
sp|P57081|WDR4_HUMAN	WDR4	tRNA (guanine-N(7)-)-methyltransferase non-catalytic subunit WDR4	10.01	12.13	46.1	9	0.779
sp|P50991|TCPD_HUMAN	CCT4	T-complex protein 1 subunit delta	77.25	81.65	83.3	118	0.779
sp|P30533|AMRP_HUMAN	LRPAP1	Alpha-2-macroglobulin receptor-associated protein	24.64	24.91	56.9	18	0.779
sp|P56270|MAZ_HUMAN	MAZ	Myc-associated zinc finger protein	6.43	6.47	30.6	4	0.779
sp|Q8TF74|WIPF2_HUMAN	WIPF2	WAS/WASL-interacting protein family member 2	7.8	8.09	30.7	6	0.779
sp|Q9H7Z7|PGES2_HUMAN	PTGES2	Prostaglandin E synthase 2	33.78	33.82	68.2	19	0.779
sp|Q96AC1|FERM2_HUMAN	FERMT2	Fermitin family homolog 2	34.79	36.87	50.4	21	0.780
sp|Q96JB5|CK5P3_HUMAN	CDK5RAP3	CDK5 regulatory subunit-associated protein 3	28.35	31.26	46.4	24	0.780
sp|Q9NQ55|SSF1_HUMAN	PPAN	Suppressor of SWI4 1 homolog	26.19	26.91	55.6	19	0.780
sp|A0JNW5|UH1BL_HUMAN	UHRF1BP1L	UHRF1-binding protein 1-like	4.11	4.24	18	2	0.780
sp|Q96FZ7|CHMP6_HUMAN	CHMP6	Charged multivesicular body protein 6	6	6.02	38.8	3	0.780
sp|P51153|RAB13_HUMAN	RAB13	Ras-related protein Rab-13	10.88	18.47	74.9	15	0.780
sp|Q9HCG8|CWC22_HUMAN	CWC22	Pre-mRNA-splicing factor CWC22 homolog	12.26	12.63	24.8	9	0.780
sp|O14524|NEMP1_HUMAN	NEMP1	Nuclear envelope integral membrane protein 1	2.9	2.98	21.2	2	0.780
sp|O60684|IMA7_HUMAN	KPNA6	Importin subunit alpha-7	41.58	41.85	64.2	35	0.780
sp|Q14126|DSG2_HUMAN	DSG2	Desmoglein-2	45.85	46.5	45.8	34	0.780
sp|Q15165|PON2_HUMAN	PON2	Serum paraoxonase/arylesterase 2	23.24	25.39	62.4	19	0.780
sp|Q92520|FAM3C_HUMAN	FAM3C	Protein FAM3C	16.1	17.3	51.5	10	0.780
sp|Q4VCS5|AMOT_HUMAN	AMOT	Angiomotin	53.8	56.88	40.3	48	0.781
sp|Q92973|TNPO1_HUMAN	TNPO1	Transportin-1	51.68	51.82	43.2	43	0.781
sp|O75190|DNJB6_HUMAN	DNAJB6	DnaJ homolog subfamily B member 6	13.38	13.45	38.7	9	0.781
sp|P40925|MDHC_HUMAN	MDH1	Malate dehydrogenase, cytoplasmic	45.1	49.25	64.7	42	0.781
sp|Q8NHS3|MFSD8_HUMAN	MFSD8	Major facilitator superfamily domain-containing protein 8	4.42	4.45	12.7	3	0.781
sp|O43395|PRPF3_HUMAN	PRPF3	U4/U6 small nuclear ribonucleoprotein Prp3	36.43	36.98	47.7	22	0.781
sp|A6NJ78|MET15_HUMAN	METTL15	Probable methyltransferase-like protein 15	13.18	13.37	51.4	7	0.782
sp|Q9Y2W6|TDRKH_HUMAN	TDRKH	Tudor and KH domain-containing protein	10.01	10.25	22.3	7	0.782
sp|Q9GZZ9|UBA5_HUMAN	UBA5	Ubiquitin-like modifier-activating enzyme 5	16.16	16.53	47.8	13	0.782
sp|P63244|GBLP_HUMAN	GNB2L1	Guanine nucleotide-binding protein subunit beta-2-like 1	55.32	55.38	87.7	88	0.782
sp|P25788|PSA3_HUMAN	PSMA3	Proteasome subunit alpha type-3	25.33	26.05	62	23	0.782
sp|Q9BZL6|KPCD2_HUMAN	PRKD2	Serine/threonine-protein kinase D2	4.29	4.83	12.4	3	0.782
sp|Q96GQ7|DDX27_HUMAN	DDX27	Probable ATP-dependent RNA helicase DDX27	62.53	64.85	53.9	32	0.782
sp|Q13435|SF3B2_HUMAN	SF3B2	Splicing factor 3B subunit 2	80.04	80.73	57.1	55	0.782
sp|P42677|RS27_HUMAN	RPS27	40S ribosomal protein S27	4.55	4.65	40.5	10	0.782
sp|Q9UHR4|BI2L1_HUMAN	BAIAP2L1	Brain-specific angiogenesis inhibitor 1-associated protein 2-like protein 1	21.04	21.62	51.7	15	0.782
sp|P82933|RT09_HUMAN	MRPS9	28S ribosomal protein S9, mitochondrial	30.22	31.34	57.6	18	0.782
sp|Q9HA82|CERS4_HUMAN	CERS4	Ceramide synthase 4	3.73	6.03	23.4	5	0.783
sp|Q96GW9|SYMM_HUMAN	MARS2	Methionine--tRNA ligase, mitochondrial	23.44	25.93	38.1	15	0.783
sp|Q14562|DHX8_HUMAN	DHX8	ATP-dependent RNA helicase DHX8	65.33	68.92	54	36	0.783

sp|P07108|ACBP_HUMAN	DBI	Acyl-CoA-binding protein	13.3	13.54	83.9	17	0.783
sp|O15504|NUPL2_HUMAN	NUPL2	Nucleoporin-like protein 2	3.27	3.39	25.5	2	0.783
sp|O43813|LANC1_HUMAN	LANCL1	LanC-like protein 1	22.29	22.35	44.1	14	0.783
sp|P02675|FIBB_HUMAN	FGB	Fibrinogen beta chain	3.05	3.82	13.2	4	0.783
sp|P35453|HXD13_HUMAN	HOXD13	Homeobox protein Hox-D13	2.37	2.4	23.9	2	0.783
sp|Q8WY36|BBX_HUMAN	BBX	HMG box transcription factor BBX	6.26	6.45	17.4	4	0.783
sp|P61981|1433G_HUMAN	YWHAG	14-3-3 protein gamma	26.14	37.44	90.3	52	0.783
sp|A8MTT3|CEBOS_HUMAN	CEBPZOS	Protein CEBPZ	2.64	2.67	32.5	2	0.783
sp|Q9C0B9|ZCHC2_HUMAN	ZCCHC2	Zinc finger CCHC domain-containing protein 2	5.42	5.58	12.6	5	0.783
sp|Q8NBS9|TXND5_HUMAN	TXNDC5	Thioredoxin domain-containing protein 5	49.05	57.87	75.7	87	0.783
sp|Q15059|BRD3_HUMAN	BRD3	Bromodomain-containing protein 3	18.23	25.7	31.8	13	0.784
sp|P15121|ALDR_HUMAN	AKR1B1	Aldose reductase	32.37	36	88.3	25	0.784
sp|Q9BRX8|F213A_HUMAN	FAM213A	Redox-regulatory protein FAM213A	16.78	16.88	50.7	10	0.784
sp|P06241|FYN_HUMAN	FYN	Tyrosine-protein kinase Fyn	3.81	13.79	32.6	9	0.784
sp|P55060|XPO2_HUMAN	CSE1L	Exportin-2	70.24	71.52	56.4	56	0.784
sp|Q00796|DHSO_HUMAN	SORD	Sorbitol dehydrogenase	35.17	35.83	83.8	29	0.784
sp|Q5W111|SPRY7_HUMAN	SPRYD7	SPRY domain-containing protein 7	7.57	7.68	41.3	4	0.785
sp|P98196|AT11A_HUMAN	ATP11A	Probable phospholipid-transporting ATPase IH	2.18	3.09	15.7	3	0.785
sp|P25787|PSA2_HUMAN	PSMA2	Proteasome subunit alpha type-2	29.06	31.85	72.6	33	0.785
sp|Q6N075|MFSD5_HUMAN	MFSD5	Molybdate-anion transporter	2.89	2.95	7.6	2	0.785
sp|Q86XN8|MEX3D_HUMAN	MEX3D	RNA-binding protein MEX3D	3.23	3.31	7.2	2	0.785
sp|Q13247|SRSF6_HUMAN	SRSF6	Serine/arginine-rich splicing factor 6	9.66	21.37	46.5	15	0.785
sp|Q8N1G2|CMTR1_HUMAN	CMTR1	Cap-specific mRNA (nucleoside-2'-O-)-methyltransferase 1	44.06	46.24	50.7	25	0.786
sp|Q9NZ63|CI078_HUMAN	C9orf78	Uncharacterized protein C9orf78	6.31	6.48	57.8	5	0.786
sp|Q99848|EBP2_HUMAN	EBNA1BP2	Probable rRNA-processing protein EBP2	27.19	27.28	58.8	17	0.786
sp|P62851|RS25_HUMAN	RPS25	40S ribosomal protein S25	14.23	14.37	59.2	14	0.786
sp|Q8N5W9|F101B_HUMAN	FAM101B	Filamin-interacting protein FAM101B	6.82	6.87	36.5	4	0.786
sp|Q9GZZ1|NAA50_HUMAN	NAA50	N-alpha-acetyltransferase 50	22.06	22.85	66.3	13	0.786
sp|O43318|M3K7_HUMAN	MAP3K7	Mitogen-activated protein kinase kinase kinase 7	14.08	14.26	36.5	9	0.786
sp|O00423|EMAL1_HUMAN	EML1	Echinoderm microtubule-associated protein-like 1	4	4.09	12.4	3	0.786
sp|P51580|TPMT_HUMAN	TPMT	Thiopurine S-methyltransferase	16.51	16.78	62	10	0.786
sp|P53621|COPA_HUMAN	COPA	Coatomer subunit alpha	141.74	142.14	74.8	105	0.787
sp|Q13572|ITPK1_HUMAN	ITPK1	Inositol-tetrakisphosphate 1-kinase	8.45	8.53	28.3	5	0.787
sp|P62280|RS11_HUMAN	RPS11	40S ribosomal protein S11	30.55	31.4	72.2	22	0.787
sp|P61326|MGN_HUMAN	MAGOH	Protein mago nashi homolog	22.69	22.75	82.2	26	0.788
sp|Q96IV0|NGLY1_HUMAN	NGLY1	Peptide-N(4)-(N-acetyl-beta-glucosaminyl)asparagine amidase	14.48	16.98	36.9	10	0.788
sp|Q9UPN6|SCAF8_HUMAN	SCAF8	Protein SCAF8	10.74	17.9	19.8	10	0.788
sp|Q99633|PRP18_HUMAN	PRPF18	Pre-mRNA-splicing factor 18	2	2.03	31.6	2	0.788
sp|Q9P2J5|SYLC_HUMAN	LARS	Leucine--tRNA ligase, cytoplasmic	94.98	96.83	58.9	72	0.788
sp|Q96QD8|S38A2_HUMAN	SLC38A2	Sodium-coupled neutral amino acid transporter 2	6	6.04	19.2	10	0.788
sp|O43819|SCO2_HUMAN	SCO2	Protein SCO2 homolog, mitochondrial	12.04	12.54	43.6	9	0.788
sp|Q96LR5|UB2E2_HUMAN	UBE2E2	Ubiquitin-conjugating enzyme E2 E2	2	2.58	27.4	4	0.788
sp|Q6ZN17|LN28B_HUMAN	LIN28B	Protein lin-28 homolog B	15.33	15.65	64	11	0.788
sp|O43847|NRDC_HUMAN	NRD1	Nardilysin	29.57	29.97	28.3	19	0.788
sp|Q86V97|KBTB6_HUMAN	KBTBD6	Kelch repeat and BTB domain-containing protein 6	4.51	4.67	15.3	3	0.788
sp|P17010|ZFX_HUMAN	ZFX	Zinc finger X-chromosomal protein	3.35	3.44	11.7	2	0.789
sp|Q99943|PLCA_HUMAN	AGPAT1	1-acyl-sn-glycerol-3-phosphate acyltransferase alpha	6	6.02	22.6	4	0.789
sp|Q969X1|LFG3_HUMAN	TMBIM1	Protein lifeguard 3	3.85	3.92	11.9	2	0.789
sp|P49916|DNLI3_HUMAN	LIG3	DNA ligase 3	58.99	59.08	54.8	33	0.790

sp|Q96SI1|KCD15_HUMAN	KCTD15	BTB/POZ domain-containing protein KCTD15	3.46	3.56	21.2	3	0.790
sp|P31948|STIP1_HUMAN	STIP1	Stress-induced-phosphoprotein 1	106.73	108.02	73.7	87	0.790
sp|O15235|RT12_HUMAN	MRPS12	28S ribosomal protein S12, mitochondrial	4.06	4.07	38.4	2	0.790
sp|O75643|U520_HUMAN	SNRNP200	U5 small nuclear ribonucleoprotein 200 kDa helicase	234.75	234.5	69.8	171	0.790
sp|Q5T2E6|CJ076_HUMAN	C10orf76	UPF0668 protein C10orf76	4.01	4.05	12.8	2	0.790
sp|P0C221|CC175_HUMAN	CCDC175	Coiled-coil domain-containing protein 175	2.01	2.26	23.6	3	0.791
sp|O60216|RAD21_HUMAN	RAD21	Double-strand-break repair protein rad21 homolog	44.42	44.51	61.2	23	0.791
sp|Q6PIU2|NCEH1_HUMAN	NCEH1	Neutral cholesterol ester hydrolase 1	13.02	13.23	34.8	7	0.791
sp|P31689|DNJA1_HUMAN	DNAJA1	DnaJ homolog subfamily A member 1	39.54	45.32	74.6	45	0.792
sp|Q96SB4|SRPK1_HUMAN	SRPK1	SRSF protein kinase 1	30.55	32.72	37.6	22	0.792
sp|Q9NYL2|MLTK_HUMAN	ZAK	Mitogen-activated protein kinase kinase kinase MLT	12.6	12.88	24.3	7	0.792
sp|Q15637|SF01_HUMAN	SF1	Splicing factor 1	30.45	30.57	37.1	24	0.792
sp|Q8WVX9|FACR1_HUMAN	FAR1	Fatty acyl-CoA reductase 1	12.4	13.29	27.4	9	0.792
sp|P27797|CALR_HUMAN	CALR	Calreticulin	64.44	65.77	81.8	120	0.792
sp|P20290|BTF3_HUMAN	BTF3	Transcription factor BTF3	23.8	23.86	76.2	42	0.792
sp|Q9NYZ3|GTSE1_HUMAN	GTSE1	G2 and S phase-expressed protein 1	1.47	2.42	20	2	0.793
sp|P50395|GDIB_HUMAN	GDI2	Rab GDP dissociation inhibitor beta	79.45	79.44	87.2	92	0.793
sp|Q14254|FLOT2_HUMAN	FLOT2	Flotillin-2	44.16	45.16	65.7	30	0.793
sp|Q9NX20|RM16_HUMAN	MRPL16	39S ribosomal protein L16, mitochondrial	14.58	15.02	60.6	8	0.793
sp|Q15269|PWP2_HUMAN	PWP2	Periodic tryptophan protein 2 homolog	48.12	50.57	46.1	29	0.793
sp|Q8IXQ3|CI040_HUMAN	C9orf40	Uncharacterized protein C9orf40	3.12	3.19	28.9	2	0.794
sp|Q92879|CELF1_HUMAN	CELF1	CUGBP Elav-like family member 1	26.37	26.6	41.6	20	0.794
sp|Q8IV38|ANKY2_HUMAN	ANKMY2	Ankyrin repeat and MYND domain-containing protein 2	4	4.02	21.1	2	0.794
sp|P05387|RLA2_HUMAN	RPLP2	60S acidic ribosomal protein P2	28.13	28.19	96.5	65	0.795
sp|Q6NSJ0|K1161_HUMAN	KIAA1161	Uncharacterized family 31 glucosidase KIAA1161	8.47	8.6	17	6	0.795
sp|O75832|PSD10_HUMAN	PSMD10	26S proteasome non-ATPase regulatory subunit 10	24.29	26.06	65	21	0.795
sp|Q9NY61|AATF_HUMAN	AATF	Protein AATF	23.12	23.22	50.9	17	0.795
sp|P28290|SSFA2_HUMAN	SSFA2	Sperm-specific antigen 2	15.84	16.6	21.8	12	0.796
sp|O00425|IF2B3_HUMAN	IGF2BP3	Insulin-like growth factor 2 mRNA-binding protein 3	46.55	55	59.6	38	0.796
sp|O43707|ACTN4_HUMAN	ACTN4	Alpha-actinin-4	77.02	117.87	78.5	127	0.796
sp|Q96H20|SNF8_HUMAN	SNF8	Vacuolar-sorting protein SNF8	6.83	7.03	51.2	7	0.796
sp|Q6UWP7|LCLT1_HUMAN	LCLAT1	Lysocardiolipin acyltransferase 1	10.55	10.7	32.1	6	0.796
sp|Q6Q0C0|TRAF7_HUMAN	TRAF7	E3 ubiquitin-protein ligase TRAF7	2.64	4.9	16.6	3	0.796
sp|O14617|AP3D1_HUMAN	AP3D1	AP-3 complex subunit delta-1	60.73	61.07	47.1	38	0.797
sp|O00299|CLIC1_HUMAN	CLIC1	Chloride intracellular channel protein 1	39.75	39.81	95.4	60	0.797
sp|O15042|SR140_HUMAN	U2SURP	U2 snRNP-associated SURP motif-containing protein	43.35	44.36	44.4	32	0.797
sp|Q99873|ANM1_HUMAN	PRMT1	Protein arginine N-methyltransferase 1	51.61	51.66	71.2	43	0.797
sp|O95453|PARN_HUMAN	PARN	Poly(A)-specific ribonuclease PARN	33.56	35.43	42.9	23	0.797
sp|O95716|RAB3D_HUMAN	RAB3D	Ras-related protein Rab-3D	2	14.06	45.7	11	0.797
sp|P30084|ECHM_HUMAN	ECHS1	Enoyl-CoA hydratase, mitochondrial	42.95	43.09	87.6	48	0.797
sp|Q96PE3|INP4A_HUMAN	INPP4A	Type I inositol 3,4-bisphosphate 4-phosphatase	13.4	13.73	26	8	0.797
sp|Q8NB78|KDM1B_HUMAN	KDM1B	Lysine-specific histone demethylase 1B	6.74	6.83	14.8	4	0.798
sp|Q01415|GALK2_HUMAN	GALK2	N-acetylgalactosamine kinase	2.72	2.85	16.4	3	0.798
sp|Q9P0J1|PDP1_HUMAN	PDP1	[Pyruvate dehydrogenase [acetyl-transferring]]-phosphatase 1, mitochondrial	9	9.08	25	5	0.798
sp|O14980|XPO1_HUMAN	XPO1	Exportin-1	61.22	63.15	51.1	58	0.798
sp|Q8N2W9|PIAS4_HUMAN	PIAS4	E3 SUMO-protein ligase PIAS4	4.56	4.67	29.6	4	0.798
sp|P33240|CSTF2_HUMAN	CSTF2	Cleavage stimulation factor subunit 2	27.81	28.03	53.6	18	0.798
sp|Q12980|NPRL3_HUMAN	NPRL3	Nitrogen permease regulator 3-like protein	4.02	4.03	11.1	2	0.798
sp|Q99986|VRK1_HUMAN	VRK1	Serine/threonine-protein kinase VRK1	38.43	38.65	74	25	0.798

sp|Q86YN1|DOPP1_HUMAN	DOLPP1	Dolichyldiphosphatase 1	4.01	4.03	33.2	2	0.798
sp|Q07960|RHG01_HUMAN	ARHGAP1	Rho GTPase-activating protein 1	26.77	26.88	58.8	16	0.799
sp|P18283|GPX2_HUMAN	GPX2	Glutathione peroxidase 2	3.35	3.53	37.4	3	0.799
sp|P28676|GRAN_HUMAN	GCA	Grancalcin	5.26	5.37	21.7	5	0.799
sp|Q3KRA9|ALKB6_HUMAN	ALKBH6	Alpha-ketoglutarate-dependent dioxygenase alkB homolog 6	1.4	1.56	18.5	3	0.800
sp|Q8NHV4|NEDD1_HUMAN	NEDD1	Protein NEDD1	6.9	7.23	21.4	5	0.800
sp|Q9H329|E41LB_HUMAN	EPB41L4B	Band 4.1-like protein 4B	2.2	4.28	12.1	2	0.800
sp|P31949|S10AB_HUMAN	S100A11	Protein S100-A11	7.06	7.49	62.9	5	0.800
sp|Q9Y2A7|NCKP1_HUMAN	NCKAP1	Nck-associated protein 1	58.94	59.74	42.3	37	0.800
sp|Q8WUY1|THEM6_HUMAN	THEM6	Protein THEM6	13.53	13.7	47.1	10	0.800
sp|Q969G9|NKD1_HUMAN	NKD1	Protein naked cuticle homolog 1	4.14	4.21	19.8	2	0.800
sp|Q9Y6G3|RM42_HUMAN	MRPL42	39S ribosomal protein L42, mitochondrial	2.21	2.24	32.4	2	0.800
sp|P27144|KAD4_HUMAN	AK4	Adenylate kinase 4, mitochondrial	19.75	20.01	67.7	11	0.800
sp|Q8ND04|SMG8_HUMAN	SMG8	Protein SMG8	8.26	10.61	22.1	8	0.800
sp|P82664|RT10_HUMAN	MRPS10	28S ribosomal protein S10, mitochondrial	5.8	6.38	51.7	8	0.801
sp|Q13228|SBP1_HUMAN	SELENBP1	Selenium-binding protein 1	34.14	34.31	62.3	19	0.801
sp|Q9BYC9|RM20_HUMAN	MRPL20	39S ribosomal protein L20, mitochondrial	10.59	11.55	57.1	7	0.801
sp|Q5VYK3|ECM29_HUMAN	ECM29	Proteasome-associated protein ECM29 homolog	115.23	118	56	73	0.801
sp|P78417|GSTO1_HUMAN	GSTO1	Glutathione S-transferase omega-1	33.71	33.97	64.7	19	0.801
sp|Q5VUJ6|LRCH2_HUMAN	LRCH2	Leucine-rich repeat and calponin homology domain-containing protein 2	4.34	4.42	18.7	2	0.801
sp|Q6NS38|ALKB2_HUMAN	ALKBH2	Alpha-ketoglutarate-dependent dioxygenase alkB homolog 2	4	4.16	25.3	2	0.801
sp|Q6P1Q0|LTMD1_HUMAN	LETMD1	LETM1 domain-containing protein 1	2.05	2.16	14.4	2	0.801
sp|P54762|EPHB1_HUMAN	EPHB1	Ephrin type-B receptor 1	2	8.14	13.4	5	0.801
sp|Q66GS9|CP135_HUMAN	CEP135	Centrosomal protein of 135 kDa	2.36	2.79	23.3	4	0.801
sp|Q9Y324|FCF1_HUMAN	FCF1	rRNA-processing protein FCF1 homolog	9.99	10.1	40.9	6	0.802
sp|Q14165|MLEC_HUMAN	MLEC	Malectin	21.56	22.32	53.1	12	0.802
sp|P04632|CPNS1_HUMAN	CAPNS1	Calpain small subunit 1	19.17	19.28	60.1	16	0.802
sp|P62312|LSM6_HUMAN	LSM6	U6 snRNA-associated Sm-like protein LSm6	15.37	15.44	90	16	0.802
sp|Q14249|NUCG_HUMAN	ENDOG	Endonuclease G, mitochondrial	7.34	7.46	31.3	4	0.802
sp|Q9Y2R9|RT07_HUMAN	MRPS7	28S ribosomal protein S7, mitochondrial	24.8	25.2	62	16	0.802
sp|Q92974|ARHG2_HUMAN	ARHGEF2	Rho guanine nucleotide exchange factor 2	40.7	44.82	40.3	30	0.802
sp|Q92522|H1X_HUMAN	H1FX	Histone H1x	16.2	18.27	58.2	19	0.802
sp|O75530|EED_HUMAN	EED	Polycomb protein EED	25.66	25.87	45.8	14	0.803
sp|Q7KZ85|SPT6H_HUMAN	SUPT6H	Transcription elongation factor SPT6	101.35	103.11	47.6	56	0.803
sp|Q9BU76|MMTA2_HUMAN	MMTAG2	Multiple myeloma tumor-associated protein 2	10.32	10.41	36.5	6	0.803
sp|P61081|UBC12_HUMAN	UBE2M	NEDD8-conjugating enzyme Ubc12	16.9	17.02	59.6	12	0.803
sp|Q13535|ATR_HUMAN	ATR	Serine/threonine-protein kinase ATR	13.07	14.11	18.8	9	0.803
sp|O00165|HAX1_HUMAN	HAX1	HCLS1-associated protein X-1	10.9	11.2	51.6	7	0.803
sp|Q12800|TFCP2_HUMAN	TFCP2	Alpha-globin transcription factor CP2	15.99	17	34.7	10	0.803
sp|P30154|2AAB_HUMAN	PPP2R1B	Serine/threonine-protein phosphatase 2A 65 kDa regulatory subunit A beta isoform	21.88	37.85	45.6	31	0.803
sp|Q76L83|ASXL2_HUMAN	ASXL2	Putative Polycomb group protein ASXL2	1.34	1.71	15.5	2	0.803
sp|Q92890|UFD1_HUMAN	UFD1L	Ubiquitin fusion degradation protein 1 homolog	23.69	23.88	56.7	15	0.803
sp|Q9HD45|TM9S3_HUMAN	TM9SF3	Transmembrane 9 superfamily member 3	15.82	16.11	29.9	13	0.803
sp|Q6EMK4|VASN_HUMAN	VASN	Vasorin	6.27	6.92	13.1	4	0.803
sp|O76071|CIAO1_HUMAN	CIAO1	Probable cytosolic iron-sulfur protein assembly protein CIAO1	15.19	15.23	39.5	9	0.803
sp|Q12968|NFAC3_HUMAN	NFATC3	Nuclear factor of activated T-cells, cytoplasmic 3	2.44	2.55	8.1	2	0.803
sp|Q9BY44|EIF2A_HUMAN	EIF2A	Eukaryotic translation initiation factor 2A	51.29	51.58	70.6	34	0.804
sp|O43303|CP110_HUMAN	CCP110	Centriolar coiled-coil protein of 110 kDa	3.28	3.53	15.1	3	0.804
sp|Q27J81|INF2_HUMAN	INF2	Inverted formin-2	44.41	45.61	40.9	30	0.804

sp|Q6UXV4|MIC27_HUMAN	APOOL	MIC complex subunit MIC27	9.94	11.2	52.6	6	0.804
sp|Q6JQN1|ACD10_HUMAN	ACAD10	Acyl-CoA dehydrogenase family member 10	13.5	14.51	22.3	10	0.805
sp|Q16352|AINX_HUMAN	INA	Alpha-internexin	2.93	5.21	31.3	8	0.805
sp|Q7L1Q6|BZW1_HUMAN	BZW1	Basic leucine zipper and W2 domain-containing protein 1	45.7	45.87	70.4	30	0.805
sp|Q9Y483|MTF2_HUMAN	MTF2	Metal-response element-binding transcription factor 2	11.91	12.5	34.6	9	0.805
sp|Q16763|UBE2S_HUMAN	UBE2S	Ubiquitin-conjugating enzyme E2 S	11.89	12.5	76.6	8	0.805
sp|Q9UDY4|DNJB4_HUMAN	DNAJB4	DnaJ homolog subfamily B member 4	10.81	17.41	51.9	9	0.805
sp|Q9Y221|NIP7_HUMAN	NIP7	60S ribosome subunit biogenesis protein NIP7 homolog	21.59	22.13	82.2	15	0.805
sp|Q9Y383|LC7L2_HUMAN	LUC7L2	Putative RNA-binding protein Luc7-like 2	28.61	29.21	49.5	18	0.805
sp|Q86UU1|PHLB1_HUMAN	PHLDB1	Pleckstrin homology-like domain family B member 1	8.45	12.42	20.8	8	0.805
sp|Q9H425|CA198_HUMAN	C1orf198	Uncharacterized protein C1orf198	3.04	3.14	19.9	3	0.805
sp|P24534|EF1B_HUMAN	EEF1B2	Elongation factor 1-beta	23.18	30.88	96.4	58	0.805
sp|Q9UBB9|TFP11_HUMAN	TFIP11	Tuftelin-interacting protein 11	26.2	26.37	37.4	14	0.805
sp|P40937|RFC5_HUMAN	RFC5	Replication factor C subunit 5	16.62	19.7	57.4	15	0.805
sp|Q9UHV9|PFD2_HUMAN	PFDN2	Prefoldin subunit 2	14.16	14.54	77.9	14	0.805
sp|Q8N573|OXR1_HUMAN	OXR1	Oxidation resistance protein 1	5.52	6.29	16.4	5	0.806
sp|Q04760|LGUL_HUMAN	GLO1	Lactoylglutathione lyase	27.52	27.62	88	24	0.806
sp|Q12874|SF3A3_HUMAN	SF3A3	Splicing factor 3A subunit 3	40.1	40.99	64.3	31	0.806
sp|Q8WVP5|TP8L1_HUMAN	TNFAIP8L1	Tumor necrosis factor alpha-induced protein 8-like protein 1	2	3.6	24.7	3	0.806
sp|Q9NR30|DDX21_HUMAN	DDX21	Nucleolar RNA helicase 2	81.43	84.16	68.5	68	0.806
sp|O94973|AP2A2_HUMAN	AP2A2	AP-2 complex subunit alpha-2	36.15	56.64	50.6	45	0.806
sp|P20336|RAB3A_HUMAN	RAB3A	Ras-related protein Rab-3A	10.37	16.21	54.1	12	0.806
sp|P09601|HMOX1_HUMAN	HMOX1	Heme oxygenase 1	22.41	23.41	57.6	19	0.806
sp|Q9NQS7|INCE_HUMAN	INCENP	Inner centromere protein	13.75	14.43	26.9	8	0.807
sp|P27105|STOM_HUMAN	STOM	Erythrocyte band 7 integral membrane protein	19.33	19.47	62.2	18	0.807
sp|Q5VZE5|NAA35_HUMAN	NAA35	N-alpha-acetyltransferase 35, NatC auxiliary subunit	21.76	24.4	38.8	12	0.807
sp|Q8N6H7|ARFG2_HUMAN	ARFGAP2	ADP-ribosylation factor GTPase-activating protein 2	12.3	12.43	31.3	6	0.807
sp|P26885|FKBP2_HUMAN	FKBP2	Peptidyl-prolyl cis-trans isomerase FKBP2	13.04	13.31	63.4	10	0.807
sp|P80723|BASP1_HUMAN	BASP1	Brain acid soluble protein 1	1.36	1.53	31.3	2	0.807
sp|Q93074|MED12_HUMAN	MED12	Mediator of RNA polymerase II transcription subunit 12	9.47	9.74	14.3	10	0.807
sp|Q9NTX5|ECHD1_HUMAN	ECHDC1	Ethylmalonyl-CoA decarboxylase	22.77	23.01	59.9	16	0.808
sp|O15066|KIF3B_HUMAN	KIF3B	Kinesin-like protein KIF3B	4.24	4.75	27.7	3	0.808
sp|Q9P0K7|RAI14_HUMAN	RAI14	Ankycorbin	32.37	35.15	48.8	21	0.808
sp|Q9NP97|DLRB1_HUMAN	DYNLRB1	Dynein light chain roadblock-type 1	10.43	10.48	83.3	11	0.808
sp|Q13310|PABP4_HUMAN	PABPC4	Polyadenylate-binding protein 4	37.71	65.95	66.8	55	0.808
sp|Q86SF2|GALT7_HUMAN	GALNT7	N-acetylgalactosaminyltransferase 7	5.16	5.35	29.2	6	0.808
sp|Q9Y5T5|UBP16_HUMAN	USP16	Ubiquitin carboxyl-terminal hydrolase 16	10.72	11.19	26.5	7	0.808
sp|P61421|VA0D1_HUMAN	ATP6V0D1	V-type proton ATPase subunit d 1	26.24	26.34	57	22	0.808
sp|Q7Z4G4|TRM11_HUMAN	TRMT11	tRNA (guanine(10)-N2)-methyltransferase homolog	6.78	6.96	19.9	5	0.808
sp|Q9Y3A2|UTP11_HUMAN	UTP11L	Probable U3 small nucleolar RNA-associated protein 11	14.62	15.95	55.3	11	0.808
sp|O43414|ERI3_HUMAN	ERI3	ERI1 exoribonuclease 3	6.54	6.64	24.9	4	0.808
sp|O00170|AIP_HUMAN	AIP	AH receptor-interacting protein	18.04	18.08	57.9	11	0.809
sp|Q8WWV3|RT4I1_HUMAN	RTN4IP1	Reticulon-4-interacting protein 1, mitochondrial	4.27	4.51	38.9	4	0.809
sp|O14770|MEIS2_HUMAN	MEIS2	Homeobox protein Meis2	4	4.03	12	3	0.809
sp|P61513|RL37A_HUMAN	RPL37A	60S ribosomal protein L37a	13.52	14.25	66.3	16	0.809
sp|Q00765|REEP5_HUMAN	REEP5	Receptor expression-enhancing protein 5	12.01	14.24	39.2	10	0.809
sp|Q9Y6N1|COX11_HUMAN	COX11	Cytochrome c oxidase assembly protein COX11, mitochondrial	9.86	10.05	35.1	7	0.809
sp|P28702|RXRB_HUMAN	RXRB	Retinoic acid receptor RXR-beta	7.48	7.8	19.9	6	0.809
sp|Q53GS7|GLE1_HUMAN	GLE1	Nucleoporin GLE1	10.27	10.92	31.4	7	0.809

sp|P48444|COPD_HUMAN	ARCN1	Coatomer subunit delta	62.01	62.03	74	39	0.809
sp|Q9UPY3|DICER_HUMAN	DICER1	Endoribonuclease Dicer	43.14	45.88	33.8	25	0.809
sp|Q8IY81|SPB1_HUMAN	FTSJ3	pre-rRNA processing protein FTSJ3	59.74	59.78	55.6	43	0.809
sp|P12532|KCRU_HUMAN	CKMT1A	Creatine kinase U-type, mitochondrial	32.07	35.64	66.2	42	0.810
sp|P11766|ADHX_HUMAN	ADH5	Alcohol dehydrogenase class-3	39.77	39.97	83.2	32	0.810
sp|Q9NUQ3|TXLNG_HUMAN	TXLNG	Gamma-taxilin	14.1	16.19	47.4	9	0.810
sp|Q8IX90|SKA3_HUMAN	SKA3	Spindle and kinetochore-associated protein 3	2	2.6	17	2	0.810
sp|Q9Y5Q9|TF3C3_HUMAN	GTF3C3	General transcription factor 3C polypeptide 3	31.34	31.51	42.6	18	0.810
sp|Q9H1Y0|ATG5_HUMAN	ATG5	Autophagy protein 5	10	10.28	40	6	0.810
sp|P23588|IF4B_HUMAN	EIF4B	Eukaryotic translation initiation factor 4B	35.74	35.86	51.1	19	0.810
sp|Q9UQR0|SCML2_HUMAN	SCML2	Sex comb on midleg-like protein 2	22.1	23.39	44.1	15	0.810
sp|Q8NDT2|RB15B_HUMAN	RBM15B	Putative RNA-binding protein 15B	6.28	6.47	30.3	4	0.810
sp|Q9NVJ2|ARL8B_HUMAN	ARL8B	ADP-ribosylation factor-like protein 8B	18.12	18.28	68.3	14	0.810
sp|Q13200|PSMD2_HUMAN	PSMD2	26S proteasome non-ATPase regulatory subunit 2	74.82	75.93	64.7	60	0.810
sp|Q9BZZ5|API5_HUMAN	API5	Apoptosis inhibitor 5	42.55	43.79	58	28	0.811
sp|P22413|ENPP1_HUMAN	ENPP1	Ectonucleotide pyrophosphatase/phosphodiesterase family member 1	21.78	22.33	26.9	17	0.811
sp|Q9NQT4|EXOS5_HUMAN	EXOSC5	Exosome complex component RRP46	13.62	14.06	59.6	10	0.811
sp|Q9H977|WDR54_HUMAN	WDR54	WD repeat-containing protein 54	5.62	5.68	13.5	3	0.812
sp|Q9H3U1|UN45A_HUMAN	UNC45A	Protein unc-45 homolog A	63.79	66.42	57.4	38	0.812
sp|Q969X5|ERGI1_HUMAN	ERGIC1	Endoplasmic reticulum-Golgi intermediate compartment protein 1	12.44	12.58	37.2	8	0.812
sp|Q8WYA6|CTBL1_HUMAN	CTNNBL1	Beta-catenin-like protein 1	38.48	38.78	54.9	22	0.812
sp|Q9BX10|GTPB2_HUMAN	GTPBP2	GTP-binding protein 2	5.34	8.03	33.1	7	0.812
sp|P61619|S61A1_HUMAN	SEC61A1	Protein transport protein Sec61 subunit alpha isoform 1	20.27	21.27	35.1	16	0.812
sp|Q9Y3B7|RM11_HUMAN	MRPL11	39S ribosomal protein L11, mitochondrial	18.77	19.49	66.7	13	0.812
sp|Q9P2B4|CT2NL_HUMAN	CTTNBP2NL	CTTNBP2 N-terminal-like protein	7.3	7.48	25.2	4	0.812
sp|Q8WVM8|SCFD1_HUMAN	SCFD1	Sec1 family domain-containing protein 1	48.73	50.28	65.1	33	0.812
sp|Q9NRY2|SOSSC_HUMAN	INIP	SS complex subunit C	2.32	2.34	22.1	2	0.812
sp|Q14669|TRIPC_HUMAN	TRIP12	E3 ubiquitin-protein ligase TRIP12	72.47	74.69	38.4	44	0.813
sp|Q13309|SKP2_HUMAN	SKP2	S-phase kinase-associated protein 2	6.83	6.97	24.5	4	0.813
sp|P33527|MRP1_HUMAN	ABCC1	Multidrug resistance-associated protein 1	54.12	55.26	40.6	40	0.813
sp|P34932|HSP74_HUMAN	HSPA4	Heat shock 70 kDa protein 4	133.91	135.14	86.7	122	0.813
sp|Q9UKA9|PTBP2_HUMAN	PTBP2	Polypyrimidine tract-binding protein 2	11.72	16.64	45.2	16	0.813
sp|P07737|PROF1_HUMAN	PFN1	Profilin-1	29.96	30.12	94.3	71	0.813
sp|Q02241|KIF23_HUMAN	KIF23	Kinesin-like protein KIF23	20.59	22.97	32	14	0.813
sp|Q9Y5U9|IR3IP_HUMAN	IER3IP1	Immediate early response 3-interacting protein 1	4	4	40.2	3	0.814
sp|P78549|NTH_HUMAN	NTHL1	Endonuclease III-like protein 1	8.01	8.01	35.3	4	0.814
sp|Q14847|LASP1_HUMAN	LASP1	LIM and SH3 domain protein 1	24.6	25.72	67.4	24	0.814
sp|Q15527|SURF2_HUMAN	SURF2	Surfeit locus protein 2	3.16	3.24	20.7	2	0.814
sp|Q13867|BLMH_HUMAN	BLMH	Bleomycin hydrolase	30.25	30.93	63.3	28	0.814
sp|Q6P996|PDXD1_HUMAN	PDXDC1	Pyridoxal-dependent decarboxylase domain-containing protein 1	53.69	54	63.6	35	0.814
sp|Q15054|DPOD3_HUMAN	POLD3	DNA polymerase delta subunit 3	15.01	15.27	41.6	10	0.814
sp|Q15645|PCH2_HUMAN	TRIP13	Pachytene checkpoint protein 2 homolog	28.62	29.25	58.8	21	0.814
sp|Q6UW02|CP20A_HUMAN	CYP20A1	Cytochrome P450 20A1	17.33	17.43	43.5	12	0.814
sp|P36551|HEM6_HUMAN	CPOX	Oxygen-dependent coproporphyrinogen-III oxidase, mitochondrial	33.48	33.57	58.8	22	0.815
sp|Q9NX62|IMPA3_HUMAN	IMPAD1	Inositol monophosphatase 3	19.57	19.7	53.8	12	0.815
sp|Q8N9N2|ASCC1_HUMAN	ASCC1	Activating signal cointegrator 1 complex subunit 1	3.92	4	20.3	2	0.815
sp|Q14839|CHD4_HUMAN	CHD4	Chromodomain-helicase-DNA-binding protein 4	146.34	147.52	57.9	90	0.815
sp|Q8N4F0|BPIB2_HUMAN	BPIFB2	BPI fold-containing family B member 2	3.13	3.34	14.9	3	0.815
sp|Q15833|STXB2_HUMAN	STXBP2	Syntaxin-binding protein 2	34.65	36.27	61.2	21	0.815

sp|Q96EU6|RRP36_HUMAN	RRP36	Ribosomal RNA processing protein 36 homolog	7.07	7.55	63.7	5	0.815
sp|Q96EY7|PTCD3_HUMAN	PTCD3	Pentatricopeptide repeat domain-containing protein 3, mitochondrial	46.21	51.53	60.1	42	0.815
sp|Q6AI08|HEAT6_HUMAN	HEATR6	HEAT repeat-containing protein 6	2.74	4.09	16.6	4	0.815
sp|O43427|FIBP_HUMAN	FIBP	Acidic fibroblast growth factor intracellular-binding protein	10.51	11.32	42.9	9	0.815
sp|Q96A49|SYAP1_HUMAN	SYAP1	Synapse-associated protein 1	33.89	34.27	61.9	18	0.815
sp|Q9HAV7|GRPE1_HUMAN	GRPEL1	GrpE protein homolog 1, mitochondrial	31.55	32.77	77	22	0.816
sp|Q6GMV3|PTRD1_HUMAN	PTRHD1	Putative peptidyl-tRNA hydrolase PTRHD1	7.8	8.28	64.3	9	0.816
sp|P42224|STAT1_HUMAN	STAT1	Signal transducer and activator of transcription 1-alpha/beta	51.69	52.32	53.3	31	0.816
sp|P55081|MFAP1_HUMAN	MFAP1	Microfibrillar-associated protein 1	9.7	9.97	33	8	0.816
sp|Q8N4A0|GALT4_HUMAN	GALNT4	Polypeptide N-acetylgalactosaminyltransferase 4	2.88	6.55	18.3	5	0.816
sp|P61247|RS3A_HUMAN	RPS3A	40S ribosomal protein S3a	53.68	53.7	79.6	61	0.816
sp|O43805|SSNA1_HUMAN	SSNA1	Sjoegren syndrome nuclear autoantigen 1	2.05	2.06	34.5	2	0.816
sp|Q6NXR4|TTI2_HUMAN	TTI2	TELO2-interacting protein 2	6.97	7.14	25.4	6	0.817
sp|P50542|PEX5_HUMAN	PEX5	Peroxisomal targeting signal 1 receptor	8.2	8.22	19.6	6	0.817
sp|P30153|2AAA_HUMAN	PPP2R1A	Serine/threonine-protein phosphatase 2A 65 kDa regulatory subunit A alpha isoform	60.85	63.85	66.9	59	0.817
sp|P12236|ADT3_HUMAN	SLC25A6	ADP/ATP translocase 3	16.99	57.84	81.9	65	0.817
sp|O60749|SNX2_HUMAN	SNX2	Sorting nexin-2	31.65	36.96	48.8	21	0.817
sp|Q92625|ANS1A_HUMAN	ANKS1A	Ankyrin repeat and SAM domain-containing protein 1A	13.25	13.44	22.2	8	0.817
sp|P36871|PGM1_HUMAN	PGM1	Phosphoglucomutase-1	66.31	66.93	77.2	42	0.817
sp|O15372|EIF3H_HUMAN	EIF3H	Eukaryotic translation initiation factor 3 subunit H	37.85	37.92	79.3	34	0.818
sp|Q9GZN1|ARP6_HUMAN	ACTR6	Actin-related protein 6	10.02	10.15	31.8	7	0.818
sp|P22695|QCR2_HUMAN	UQCRC2	Cytochrome b-c1 complex subunit 2, mitochondrial	43.13	43.19	88.1	59	0.818
sp|Q02086|SP2_HUMAN	SP2	Transcription factor Sp2	2.94	3.09	8.2	3	0.818
sp|Q8TBE9|NANP_HUMAN	NANP	N-acylneuraminate-9-phosphatase	7.32	7.55	55.7	6	0.818
sp|Q9Y5V0|ZN706_HUMAN	ZNF706	Zinc finger protein 706	3.8	3.89	46.1	2	0.818
sp|Q5VZ89|DEN4C_HUMAN	DENND4C	DENN domain-containing protein 4C	4.12	5.2	11.9	3	0.818
sp|Q9Y243|AKT3_HUMAN	AKT3	RAC-gamma serine/threonine-protein kinase	1.62	8.48	23	4	0.818
sp|Q6P161|RM54_HUMAN	MRPL54	39S ribosomal protein L54, mitochondrial	6.01	6.03	48.6	3	0.818
sp|Q03701|CEBPZ_HUMAN	CEBPZ	CCAAT/enhancer-binding protein zeta	60.23	62.44	52.2	42	0.819
sp|P51648|AL3A2_HUMAN	ALDH3A2	Fatty aldehyde dehydrogenase	19.47	24.85	42.5	19	0.819
sp|P17544|ATF7_HUMAN	ATF7	Cyclic AMP-dependent transcription factor ATF-7	4.01	4.01	12.2	2	0.819
sp|Q92769|HDAC2_HUMAN	HDAC2	Histone deacetylase 2	24.53	26.82	60.3	19	0.819
sp|Q8WV74|NUDT8_HUMAN	NUDT8	Nucleoside diphosphate-linked moiety X motif 8, mitochondrial	4	4	12.3	2	0.819
sp|Q86T24|KAISO_HUMAN	ZBTB33	Transcriptional regulator Kaiso	20.43	20.62	26.8	13	0.819
sp|Q9H3H5|GPT_HUMAN	DPAGT1	UDP-N-acetylglucosamine--dolichyl-phosphate N-acetylglucosaminephosphotransferase	2.08	2.57	7.8	2	0.819
sp|Q8IVS8|GLCTK_HUMAN	GLYCTK	Glycerate kinase	2	2.08	12.4	3	0.820
sp|Q969Z0|TBRG4_HUMAN	TBRG4	Protein TBRG4	37.75	42.54	61.8	30	0.820
sp|P30101|PDIA3_HUMAN	PDIA3	Protein disulfide-isomerase A3	112.01	112.88	84.4	144	0.820
sp|P57721|PCBP3_HUMAN	PCBP3	Poly(rC)-binding protein 3	2	15.94	36.1	24	0.820
sp|Q7L099|RUFY3_HUMAN	RUFY3	Protein RUFY3	2	2.11	22.8	2	0.820
sp|O95208|EPN2_HUMAN	EPN2	Epsin-2	2.29	2.34	13.4	2	0.820
sp|Q9NV66|TYW1_HUMAN	TYW1	S-adenosyl-L-methionine-dependent tRNA 4-demethylwyosine synthase	4.29	4.48	19.1	3	0.821
sp|Q99496|RING2_HUMAN	RNF2	E3 ubiquitin-protein ligase RING2	17.61	19.4	49.1	11	0.821
sp|Q9UBC2|EP15R_HUMAN	EPS15L1	Epidermal growth factor receptor substrate 15-like 1	39.94	40.08	53.2	24	0.821
sp|O00159|MYO1C_HUMAN	MYO1C	Unconventional myosin-Ic	67.52	72.89	62.1	44	0.821
sp|P49589|SYCC_HUMAN	CARS	Cysteine--tRNA ligase, cytoplasmic	39.57	39.95	58.8	20	0.821
sp|Q5JTJ3|COA6_HUMAN	COA6	Cytochrome c oxidase assembly factor 6 homolog	7.24	7.61	56.8	4	0.821
sp|O43657|TSN6_HUMAN	TSPAN6	Tetraspanin-6	11.6	11.65	32.2	6	0.821
sp|P60903|S10AA_HUMAN	S100A10	Protein S100-A10	2.51	3.1	36.1	4	0.821

sp|Q6IA86|ELP2_HUMAN	ELP2	Elongator complex protein 2	24.14	24.34	33.9	13	0.822
sp|Q8ND30|LIPB2_HUMAN	PPFIBP2	Liprin-beta-2	2.01	6.32	22.4	3	0.823
sp|P05091|ALDH2_HUMAN	ALDH2	Aldehyde dehydrogenase, mitochondrial	49.43	56.7	80.5	44	0.823
sp|Q96DI7|SNR40_HUMAN	SNRNP40	U5 small nuclear ribonucleoprotein 40 kDa protein	31.77	32.25	79	28	0.823
sp|Q15021|CND1_HUMAN	NCAPD2	Condensin complex subunit 1	68.01	68.36	47.4	43	0.823
sp|Q8NCM8|DYHC2_HUMAN	DYNC2H1	Cytoplasmic dynein 2 heavy chain 1	15.81	28.83	23	20	0.823
sp|P12955|PEPD_HUMAN	PEPD	Xaa-Pro dipeptidase	31.71	31.81	65.5	29	0.823
sp|Q9NRV9|HEBP1_HUMAN	HEBP1	Heme-binding protein 1	15.95	16.03	72	10	0.824
sp|Q9UPQ9|TNR6B_HUMAN	TNRC6B	Trinucleotide repeat-containing gene 6B protein	7.57	7.69	9.9	6	0.824
sp|Q9UHY7|ENOPH_HUMAN	ENOPH1	Enolase-phosphatase E1	10.65	10.69	45.2	13	0.824
sp|Q9BRT6|LLPH_HUMAN	LLPH	Protein LLP homolog	4.56	4.65	47.3	4	0.824
sp|Q01081|U2AF1_HUMAN	U2AF1	Splicing factor U2AF 35 kDa subunit	20.92	20.97	67.5	19	0.824
sp|Q9BSF4|CS052_HUMAN	C19orf52	Uncharacterized protein C19orf52	4.46	4.51	32.3	5	0.825
sp|P62834|RAP1A_HUMAN	RAP1A	Ras-related protein Rap-1A	6.7	26.51	82.1	31	0.825
sp|O94915|FRYL_HUMAN	FRYL	Protein furry homolog-like	4.36	4.98	15.6	8	0.825
sp|Q9BZF3|OSBL6_HUMAN	OSBPL6	Oxysterol-binding protein-related protein 6	1.76	4.26	17.1	2	0.825
sp|Q9HC52|CBX8_HUMAN	CBX8	Chromobox protein homolog 8	15.33	15.49	53	7	0.825
sp|Q07020|RL18_HUMAN	RPL18	60S ribosomal protein L18	12.56	13.34	64.9	21	0.825
sp|Q9NTJ3|SMC4_HUMAN	SMC4	Structural maintenance of chromosomes protein 4	83.38	85.14	61.2	54	0.825
sp|Q9NUP9|LIN7C_HUMAN	LIN7C	Protein lin-7 homolog C	16.28	19.93	74.6	17	0.825
sp|Q5XKP0|MIC13_HUMAN	MIC13	MIC complex subunit MIC13	6.07	6.08	72.9	3	0.826
sp|Q15084|PDIA6_HUMAN	PDIA6	Protein disulfide-isomerase A6	70.44	72.57	80.9	127	0.826
sp|P27540|ARNT_HUMAN	ARNT	Aryl hydrocarbon receptor nuclear translocator	6.07	6.92	14.6	4	0.826
sp|Q9P2I0|CPSF2_HUMAN	CPSF2	Cleavage and polyadenylation specificity factor subunit 2	53.2	55.48	63.9	31	0.826
sp|P21281|VATB2_HUMAN	ATP6V1B2	V-type proton ATPase subunit B, brain isoform	51.93	51.96	79.8	39	0.826
sp|P03915|NU5M_HUMAN	MT-ND5	NADH-ubiquinone oxidoreductase chain 5	6.96	7.07	13.8	5	0.826
sp|P49753|ACOT2_HUMAN	ACOT2	Acyl-coenzyme A thioesterase 2, mitochondrial	2	46.37	74.7	41	0.826
sp|Q9NYP3|DONS_HUMAN	DONSON	Protein downstream neighbor of Son	3.51	3.62	16.8	2	0.826
sp|Q16822|PCKGM_HUMAN	PCK2	Phosphoenolpyruvate carboxykinase [GTP], mitochondrial	58.63	58.7	69.1	55	0.826
sp|Q5SY16|NOL9_HUMAN	NOL9	Polynucleotide 5'-hydroxyl-kinase NOL9	22.6	25.33	36	18	0.827
sp|P09661|RU2A_HUMAN	SNRPA1	U2 small nuclear ribonucleoprotein A'	24.99	25.19	68.6	32	0.827
sp|P06737|PYGL_HUMAN	PYGL	Glycogen phosphorylase, liver form	97.27	97.74	67.5	73	0.827
sp|Q15293|RCN1_HUMAN	RCN1	Reticulocalbin-1	31.36	31.46	63.4	20	0.827
sp|O95219|SNX4_HUMAN	SNX4	Sorting nexin-4	14.42	15.03	42.2	10	0.827
sp|P55084|ECHB_HUMAN	HADHB	Trifunctional enzyme subunit beta, mitochondrial	66.35	66.45	73	49	0.827
sp|Q86WA6|BPHL_HUMAN	BPHL	Valacyclovir hydrolase	14.35	14.44	62.9	11	0.827
sp|Q12873|CHD3_HUMAN	CHD3	Chromodomain-helicase-DNA-binding protein 3	2.91	31.61	21.2	27	0.827
sp|Q9H488|OFUT1_HUMAN	POFUT1	GDP-fucose protein O-fucosyltransferase 1	28.11	28.19	58	18	0.827
sp|P08174|DAF_HUMAN	CD55	Complement decay-accelerating factor	10.77	11.08	28.6	7	0.827
sp|P0DMN0|ST1A4_HUMAN	SULT1A4	Sulfotransferase 1A4	14.14	35.88	80.3	35	0.827
sp|P43487|RANG_HUMAN	RANBP1	Ran-specific GTPase-activating protein	19.54	19.65	78.6	27	0.827
sp|Q9BXK5|B2L13_HUMAN	BCL2L13	Bcl-2-like protein 13	15.93	16.24	45.4	13	0.828
sp|Q9Y4R8|TELO2_HUMAN	TELO2	Telomere length regulation protein TEL2 homolog	24.25	24.38	33.3	14	0.828
sp|Q9Y520|PRC2C_HUMAN	PRRC2C	Protein PRRC2C	77.73	78.49	31.4	45	0.828
sp|P06865|HEXA_HUMAN	HEXA	Beta-hexosaminidase subunit alpha	21.71	27.86	48.4	20	0.828
sp|Q9NVN8|GNL3L_HUMAN	GNL3L	Guanine nucleotide-binding protein-like 3-like protein	16.41	18.53	36.6	9	0.828
sp|Q14676|MDC1_HUMAN	MDC1	Mediator of DNA damage checkpoint protein 1	85.42	85.79	49.5	53	0.829
sp|Q66K14|TBC9B_HUMAN	TBC1D9B	TBC1 domain family member 9B	16.24	16.76	25.7	12	0.829
sp|Q92820|GGH_HUMAN	GGH	Gamma-glutamyl hydrolase	12.68	12.9	34.3	10	0.829

sp|Q9UGI8|TES_HUMAN	TES	Testin	37.08	39.01	61.8	22	0.829
sp|P18621|RL17_HUMAN	RPL17	60S ribosomal protein L17	20.2	20.3	58.2	28	0.829
sp|Q13363|CTBP1_HUMAN	CTBP1	C-terminal-binding protein 1	24.36	25.05	56.4	23	0.829
sp|O14654|IRS4_HUMAN	IRS4	Insulin receptor substrate 4	62.26	64.28	51.2	40	0.829
sp|Q13107|UBP4_HUMAN	USP4	Ubiquitin carboxyl-terminal hydrolase 4	12.88	19.45	23.1	14	0.830
sp|Q8TBP6|S2540_HUMAN	SLC25A40	Solute carrier family 25 member 40	6.05	6.12	33.4	5	0.830
sp|Q8IWF6|DEN6A_HUMAN	DENND6A	Protein DENND6A	2.02	2.03	17.4	2	0.830
sp|Q8IY31|IFT20_HUMAN	IFT20	Intraflagellar transport protein 20 homolog	2.52	2.55	41.7	2	0.830
sp|O60507|TPST1_HUMAN	TPST1	Protein-tyrosine sulfotransferase 1	4.01	4.39	20.8	3	0.830
sp|P30050|RL12_HUMAN	RPL12	60S ribosomal protein L12	19.72	20.03	73.3	29	0.830
sp|Q9H8M7|F188A_HUMAN	FAM188A	Protein FAM188A	13.52	13.64	39.3	8	0.830
sp|Q9Y5S9|RBM8A_HUMAN	RBM8A	RNA-binding protein 8A	13.74	14.4	75.9	22	0.831
sp|P52732|KIF11_HUMAN	KIF11	Kinesin-like protein KIF11	58.19	59.29	49.2	35	0.831
sp|P61019|RAB2A_HUMAN	RAB2A	Ras-related protein Rab-2A	22.78	24.29	69.8	29	0.831
sp|Q8NDD1|CA131_HUMAN	C1orf131	Uncharacterized protein C1orf131	15.2	17.07	42.9	9	0.831
sp|O75380|NDUS6_HUMAN	NDUFS6	NADH dehydrogenase [ubiquinone] iron-sulfur protein 6, mitochondrial	12.66	12.76	71	7	0.831
sp|O75880|SCO1_HUMAN	SCO1	Protein SCO1 homolog, mitochondrial	7.47	7.55	45.2	6	0.831
sp|Q14151|SAFB2_HUMAN	SAFB2	Scaffold attachment factor B2	23.42	44.14	44.3	29	0.831
sp|Q9BZF1|OSBL8_HUMAN	OSBPL8	Oxysterol-binding protein-related protein 8	27.09	27.32	34.4	17	0.831
sp|O00469|PLOD2_HUMAN	PLOD2	Procollagen-lysine,2-oxoglutarate 5-dioxygenase 2	45.77	46.76	51.7	27	0.831
sp|O75153|CLU_HUMAN	CLUH	Clustered mitochondria protein homolog	68.7	73.24	49.7	38	0.831
sp|Q9UKX7|NUP50_HUMAN	NUP50	Nuclear pore complex protein Nup50	30.56	32.61	75.2	24	0.832
sp|P62495|ERF1_HUMAN	ETF1	Eukaryotic peptide chain release factor subunit 1	41.95	44.15	71.6	31	0.832
sp|P30837|AL1B1_HUMAN	ALDH1B1	Aldehyde dehydrogenase X, mitochondrial	55.59	59.83	87	55	0.832
sp|Q9UFG5|CS025_HUMAN	C19orf25	UPF0449 protein C19orf25	6.58	6.63	70.3	4	0.832
sp|Q8NEJ9|NGDN_HUMAN	NGDN	Neuroguidin	12.29	12.62	43.8	7	0.832
sp|P61160|ARP2_HUMAN	ACTR2	Actin-related protein 2	32.53	35.75	64.5	28	0.832
sp|Q8WUH6|TM263_HUMAN	TMEM263	Transmembrane protein 263	10	10	62.1	5	0.832
sp|Q9HAV4|XPO5_HUMAN	XPO5	Exportin-5	60.21	61.56	46.8	48	0.832
sp|Q13492|PICAL_HUMAN	PICALM	Phosphatidylinositol-binding clathrin assembly protein	29.61	29.9	42.8	24	0.832
sp|O00287|RFXAP_HUMAN	RFXAP	Regulatory factor X-associated protein	4.14	4.48	16.5	2	0.833
sp|Q9NRP0|OSTC_HUMAN	OSTC	Oligosaccharyltransferase complex subunit TC	4.01	4.01	23.5	3	0.833
sp|Q9BVI4|NOC4L_HUMAN	NOC4L	Nucleolar complex protein 4 homolog	24.13	24.21	51.4	14	0.833
sp|Q96T37|RBM15_HUMAN	RBM15	Putative RNA-binding protein 15	51.35	53.65	46.2	30	0.833
sp|Q08J23|NSUN2_HUMAN	NSUN2	tRNA (cytosine(34)-C(5))-methyltransferase	68.58	68.63	73.8	46	0.833
sp|P19105|ML12A_HUMAN	MYL12A	Myosin regulatory light chain 12A	17.43	17.49	66.7	13	0.834
sp|O60243|H6ST1_HUMAN	HS6ST1	Heparan-sulfate 6-O-sulfotransferase 1	2	2.08	15.6	2	0.834
sp|P08183|MDR1_HUMAN	ABCB1	Multidrug resistance protein 1	44.45	44.88	39.8	24	0.834
sp|Q15369|ELOC_HUMAN	TCEB1	Transcription elongation factor B polypeptide 1	12	12	66.1	13	0.834
sp|Q6P1X6|CH082_HUMAN	C8orf82	UPF0598 protein C8orf82	9.85	11.38	48.2	7	0.834
sp|P42892|ECE1_HUMAN	ECE1	Endothelin-converting enzyme 1	7.82	8.21	19.9	8	0.834
sp|Q99807|COQ7_HUMAN	COQ7	5-demethoxyubiquinone hydroxylase, mitochondrial	8.08	8.11	38.7	5	0.835
sp|Q13112|CAF1B_HUMAN	CHAF1B	Chromatin assembly factor 1 subunit B	19.64	19.76	39.2	15	0.835
sp|Q9C0C9|UBE2O_HUMAN	UBE2O	E2/E3 hybrid ubiquitin-protein ligase UBE2O	52.61	54.5	50	32	0.835
sp|Q96AG4|LRC59_HUMAN	LRRC59	Leucine-rich repeat-containing protein 59	34.99	36.38	64.8	29	0.835
sp|Q9HCS7|SYF1_HUMAN	XAB2	Pre-mRNA-splicing factor SYF1	43.97	46.84	47.8	24	0.836
sp|Q9H7Z3|NRDE2_HUMAN	NRDE2	Protein NRDE2 homolog	3.75	4.91	11.9	4	0.836
sp|Q8IWA4|MFN1_HUMAN	MFN1	Mitofusin-1	20.81	25.88	41.8	14	0.836
sp|Q6W2J9|BCOR_HUMAN	BCOR	BCL-6 corepressor	13.41	13.69	16	9	0.836

sp|P49005|DPOD2_HUMAN	POLD2	DNA polymerase delta subunit 2	8.93	8.98	22.2	5	0.836
sp|P52888|THOP1_HUMAN	THOP1	Thimet oligopeptidase	36.16	41.65	53.4	25	0.836
sp|P21333|FLNA_HUMAN	FLNA	Filamin-A	282.08	281.99	75	251	0.836
sp|Q9H160|ING2_HUMAN	ING2	Inhibitor of growth protein 2	2.69	3.07	24.6	2	0.836
sp|O75694|NU155_HUMAN	NUP155	Nuclear pore complex protein Nup155	68.19	70.42	50.5	54	0.836
sp|P49848|TAF6_HUMAN	TAF6	Transcription initiation factor TFIID subunit 6	13.62	13.81	37.1	9	0.836
sp|Q70E73|RAPH1_HUMAN	RAPH1	Ras-associated and pleckstrin homology domains-containing protein 1	8.23	10.78	11.4	6	0.836
sp|Q9NWV8|BABA1_HUMAN	BABAM1	BRISC and BRCA1-A complex member 1	14.77	14.85	40.1	9	0.836
sp|Q7L0Y3|MRRP1_HUMAN	TRMT10C	Mitochondrial ribonuclease P protein 1	43.42	43.67	73.2	30	0.836
sp|P46783|RS10_HUMAN	RPS10	40S ribosomal protein S10	20.69	20.94	61.8	20	0.836
sp|P13612|ITA4_HUMAN	ITGA4	Integrin alpha-4	2.09	2.74	10.9	2	0.837
sp|Q9H8W4|PKHF2_HUMAN	PLEKHF2	Pleckstrin homology domain-containing family F member 2	8	8.01	35.7	4	0.837
sp|Q9C0I1|MTMRC_HUMAN	MTMR12	Myotubularin-related protein 12	14.74	17.06	33.2	9	0.837
sp|Q9BQ67|GRWD1_HUMAN	GRWD1	Glutamate-rich WD repeat-containing protein 1	31.89	32.22	59.2	24	0.837
sp|Q6PCB8|EMB_HUMAN	EMB	Embigin	2	2.01	12.8	2	0.837
sp|Q8TC07|TBC15_HUMAN	TBC1D15	TBC1 domain family member 15	17.06	19.79	39.7	15	0.837
sp|P61313|RL15_HUMAN	RPL15	60S ribosomal protein L15	18.06	18.13	53.4	14	0.837
sp|P09497|CLCB_HUMAN	CLTB	Clathrin light chain B	16.27	16.45	39.3	11	0.837
sp|Q9H936|GHC1_HUMAN	SLC25A22	Mitochondrial glutamate carrier 1	21.43	22.42	79	15	0.838
sp|P61353|RL27_HUMAN	RPL27	60S ribosomal protein L27	28.83	30.81	76.5	26	0.838
sp|Q9BYT8|NEUL_HUMAN	NLN	Neurolysin, mitochondrial	48.93	49.45	54.6	28	0.838
sp|P31939|PUR9_HUMAN	ATIC	Bifunctional purine biosynthesis protein PURH	90.4	90.9	91.1	97	0.838
sp|O75122|CLAP2_HUMAN	CLASP2	CLIP-associating protein 2	33.03	44.69	40.6	26	0.838
sp|Q9BTU6|P4K2A_HUMAN	PI4K2A	Phosphatidylinositol 4-kinase type 2-alpha	14.19	15.11	47.6	8	0.838
sp|P24386|RAE1_HUMAN	CHM	Rab proteins geranylgeranyltransferase component A 1	11.11	11.38	21.9	7	0.838
sp|O75663|TIPRL_HUMAN	TIPRL	TIP41-like protein	12.92	13.62	52.9	8	0.838
sp|O15392|BIRC5_HUMAN	BIRC5	Baculoviral IAP repeat-containing protein 5	8.21	8.24	79.6	5	0.839
sp|Q9NVC3|S38A7_HUMAN	SLC38A7	Putative sodium-coupled neutral amino acid transporter 7	1.4	1.49	9.7	2	0.839
sp|Q86SZ2|TPC6B_HUMAN	TRAPPC6B	Trafficking protein particle complex subunit 6B	4.68	4.9	67.7	4	0.839
sp|Q8N163|CCAR2_HUMAN	CCAR2	Cell cycle and apoptosis regulator protein 2	56.21	59.4	64.1	44	0.839
sp|Q13148|TADBP_HUMAN	TARDBP	TAR DNA-binding protein 43	26.59	27.99	54.8	26	0.839
sp|Q15181|IPYR_HUMAN	PPA1	Inorganic pyrophosphatase	46.03	47.05	83.7	42	0.839
sp|P15880|RS2_HUMAN	RPS2	40S ribosomal protein S2	39.38	39.63	68.3	39	0.839
sp|Q08211|DHX9_HUMAN	DHX9	ATP-dependent RNA helicase A	127.12	129.15	66.5	152	0.839
sp|P54646|AAPK2_HUMAN	PRKAA2	5'-AMP-activated protein kinase catalytic subunit alpha-2	6.93	15.04	29.4	9	0.839
sp|O43752|STX6_HUMAN	STX6	Syntaxin-6	7.95	10.2	37.7	7	0.840
sp|P22415|USF1_HUMAN	USF1	Upstream stimulatory factor 1	2.45	4.73	19.7	2	0.840
sp|O14562|UBFD1_HUMAN	UBFD1	Ubiquitin domain-containing protein UBFD1	17.07	18.24	70.6	12	0.840
sp|Q07864|DPOE1_HUMAN	POLE	DNA polymerase epsilon catalytic subunit A	42.51	46.17	27	24	0.840
sp|Q15424|SAFB1_HUMAN	SAFB	Scaffold attachment factor B1	50.54	53.28	50.7	37	0.840
sp|P54132|BLM_HUMAN	BLM	Bloom syndrome protein	14.38	17.2	22.6	9	0.840
sp|Q86U70|LDB1_HUMAN	LDB1	LIM domain-binding protein 1	5.86	5.94	18.7	4	0.840
sp|Q9UN81|LORF1_HUMAN	L1RE1	LINE-1 retrotransposable element ORF1 protein	9.14	9.43	43.8	6	0.840
sp|Q86YT6|MIB1_HUMAN	MIB1	E3 ubiquitin-protein ligase MIB1	5.27	5.53	17.3	5	0.840
sp|Q8N9N8|EIF1A_HUMAN	EIF1AD	Probable RNA-binding protein EIF1AD	10.27	10.36	50.3	8	0.841
sp|P49591|SYSC_HUMAN	SARS	Serine--tRNA ligase, cytoplasmic	41.51	41.69	65.4	30	0.841
sp|Q86UW7|CAPS2_HUMAN	CADPS2	Calcium-dependent secretion activator 2	1.35	1.69	19.6	3	0.841
sp|Q9BUR5|MIC26_HUMAN	APOO	MIC complex subunit MIC26	6.11	6.12	48	3	0.841
sp|O43353|RIPK2_HUMAN	RIPK2	Receptor-interacting serine/threonine-protein kinase 2	5.05	5.15	26.1	3	0.842

sp|P23511|NFYA_HUMAN	NFYA	Nuclear transcription factor Y subunit alpha	3.74	3.83	17.6	3	0.842
sp|Q9H7S9|ZN703_HUMAN	ZNF703	Zinc finger protein 703	9.05	9.44	19.8	7	0.842
sp|Q9UNH7|SNX6_HUMAN	SNX6	Sorting nexin-6	38.95	39.41	72.7	28	0.842
sp|Q5VTB9|RN220_HUMAN	RNF220	E3 ubiquitin-protein ligase RNF220	3.7	3.81	10.4	3	0.842
sp|Q8WZA9|IRGQ_HUMAN	IRGQ	Immunity-related GTPase family Q protein	28.5	28.51	47.7	15	0.842
sp|O14818|PSA7_HUMAN	PSMA7	Proteasome subunit alpha type-7	31.09	32.14	80.7	29	0.842
sp|P26639|SYTC_HUMAN	TARS	Threonine--tRNA ligase, cytoplasmic	70.89	74.31	68.7	61	0.842
sp|Q6ZYL4|TF2H5_HUMAN	GTF2H5	General transcription factor IIH subunit 5	4	4.02	49.3	4	0.842
sp|P42704|LPPRC_HUMAN	LRPPRC	Leucine-rich PPR motif-containing protein, mitochondrial	215.9	217	84.9	199	0.842
sp|Q9HB71|CYBP_HUMAN	CACYBP	Calcyclin-binding protein	28.47	30.79	81.6	25	0.843
sp|Q9UBM7|DHCR7_HUMAN	DHCR7	7-dehydrocholesterol reductase	16.9	17.57	22.3	10	0.843
sp|Q15041|AR6P1_HUMAN	ARL6IP1	ADP-ribosylation factor-like protein 6-interacting protein 1	4.04	4.33	38.9	9	0.843
sp|O43143|DHX15_HUMAN	DHX15	Pre-mRNA-splicing factor ATP-dependent RNA helicase DHX15	79.62	80.01	67.4	64	0.843
sp|P78406|RAE1L_HUMAN	RAE1	mRNA export factor	34.99	35.11	82.3	36	0.843
sp|Q9BQ70|TCF25_HUMAN	TCF25	Transcription factor 25	11.53	11.78	28.6	7	0.843
sp|P28370|SMCA1_HUMAN	SMARCA1	Probable global transcription activator SNF2L1	44.41	74.3	47.3	40	0.843
sp|O75044|SRGP2_HUMAN	SRGAP2	SLIT-ROBO Rho GTPase-activating protein 2	23.55	24.2	34.7	14	0.844
sp|P53985|MOT1_HUMAN	SLC16A1	Monocarboxylate transporter 1	12.73	12.98	20.4	15	0.844
sp|Q01105|SET_HUMAN	SET	Protein SET	28.37	28.84	68.3	33	0.844
sp|Q5T9A4|ATD3B_HUMAN	ATAD3B	ATPase family AAA domain-containing protein 3B	57.82	57.95	65.7	40	0.844
sp|O14519|CDKA1_HUMAN	CDK2AP1	Cyclin-dependent kinase 2-associated protein 1	1.63	1.74	33.9	2	0.844
sp|O95864|FADS2_HUMAN	FADS2	Fatty acid desaturase 2	6.08	8.7	22.5	5	0.844
sp|P33176|KINH_HUMAN	KIF5B	Kinesin-1 heavy chain	97.5	98.45	72.5	59	0.844
sp|O15400|STX7_HUMAN	STX7	Syntaxin-7	21.23	21.31	79.7	15	0.845
sp|O75907|DGAT1_HUMAN	DGAT1	Diacylglycerol O-acyltransferase 1	4	4.01	17.6	2	0.845
sp|Q8TDY2|RBCC1_HUMAN	RB1CC1	RB1-inducible coiled-coil protein 1	10.68	12.66	25	10	0.845
sp|P04083|ANXA1_HUMAN	ANXA1	Annexin A1	11.76	12.41	51.7	8	0.845
sp|Q9Y3A4|RRP7A_HUMAN	RRP7A	Ribosomal RNA-processing protein 7 homolog A	16.13	17.48	51.1	14	0.845
sp|P52597|HNRPF_HUMAN	HNRNPF	Heterogeneous nuclear ribonucleoprotein F	33.33	43.32	74	70	0.845
sp|O75915|PRAF3_HUMAN	ARL6IP5	PRA1 family protein 3	14	14	31.4	11	0.845
sp|P57737|CORO7_HUMAN	CORO7	Coronin-7	25.18	26.69	35.4	16	0.845
sp|Q5JSZ5|PRC2B_HUMAN	PRRC2B	Protein PRRC2B	10.85	12.34	15.4	8	0.845
sp|O00267|SPT5H_HUMAN	SUPT5H	Transcription elongation factor SPT5	75.97	76.82	50.1	44	0.845
sp|Q92804|RBP56_HUMAN	TAF15	TATA-binding protein-associated factor 2N	12.39	18.7	49.7	26	0.845
sp|Q16850|CP51A_HUMAN	CYP51A1	Lanosterol 14-alpha demethylase	30.78	31.51	56.9	18	0.846
sp|Q96RE7|NACC1_HUMAN	NACC1	Nucleus accumbens-associated protein 1	15.46	15.53	31.9	10	0.846
sp|Q9NX00|TM160_HUMAN	TMEM160	Transmembrane protein 160	4.72	4.84	28.2	3	0.846
sp|P62820|RAB1A_HUMAN	RAB1A	Ras-related protein Rab-1A	43.3	43.43	92.7	49	0.846
sp|O75683|SURF6_HUMAN	SURF6	Surfeit locus protein 6	15.92	16.73	46.8	11	0.846
sp|P61758|PFD3_HUMAN	VBP1	Prefoldin subunit 3	23.8	23.94	72.1	16	0.846
sp|Q8NBZ7|UXS1_HUMAN	UXS1	UDP-glucuronic acid decarboxylase 1	6.16	6.99	24.3	5	0.846
sp|O60506|HNRPQ_HUMAN	SYNCRIP	Heterogeneous nuclear ribonucleoprotein Q	69.5	72.57	74.6	71	0.846
sp|P09960|LKHA4_HUMAN	LTA4H	Leukotriene A-4 hydrolase	52.98	55.15	62.2	42	0.846
sp|P62263|RS14_HUMAN	RPS14	40S ribosomal protein S14	23.93	23.96	76.2	31	0.847
sp|Q96RL7|VP13A_HUMAN	VPS13A	Vacuolar protein sorting-associated protein 13A	18.81	21.62	20	14	0.847
sp|O60870|KIN17_HUMAN	KIN	DNA/RNA-binding protein KIN17	10.56	13.04	47.8	8	0.847
sp|Q5T0B9|ZN362_HUMAN	ZNF362	Zinc finger protein 362	6.34	6.39	26.7	4	0.847
sp|Q04726|TLE3_HUMAN	TLE3	Transducin-like enhancer protein 3	6.28	10.38	17.4	6	0.847
sp|Q9BQ04|RBM4B_HUMAN	RBM4B	RNA-binding protein 4B	4	26	43.5	14	0.847

sp|P10643|CO7_HUMAN	C7	Complement component C7	3.69	3.82	9.8	3	0.847
sp|O43933|PEX1_HUMAN	PEX1	Peroxisome biogenesis factor 1	6.32	8.71	16.8	5	0.847
sp|Q9BRJ7|SDOS_HUMAN	NUDT16L1	Protein syndesmos	10.52	12.9	55	8	0.847
sp|Q9HC07|TM165_HUMAN	TMEM165	Transmembrane protein 165	9.77	9.87	38.3	7	0.847
sp|P80365|DHI2_HUMAN	HSD11B2	Corticosteroid 11-beta-dehydrogenase isozyme 2	2	4	11.4	2	0.848
sp|Q9NR31|SAR1A_HUMAN	SAR1A	GTP-binding protein SAR1a	27.05	27.11	78.3	30	0.848
sp|Q96A26|F162A_HUMAN	FAM162A	Protein FAM162A	10.18	10.32	52	7	0.848
sp|P37837|TALDO_HUMAN	TALDO1	Transaldolase	43.37	45.44	75.4	31	0.848
sp|Q8TF46|DI3L1_HUMAN	DIS3L	DIS3-like exonuclease 1	4.03	4.05	15.1	2	0.848
sp|Q96I25|SPF45_HUMAN	RBM17	Splicing factor 45	18.98	22.15	63.3	14	0.848
sp|P49441|INPP_HUMAN	INPP1	Inositol polyphosphate 1-phosphatase	9.27	9.55	28.3	6	0.848
sp|Q8NAV1|PR38A_HUMAN	PRPF38A	Pre-mRNA-splicing factor 38A	17.89	18.02	48.1	12	0.848
sp|O43852|CALU_HUMAN	CALU	Calumenin	42.29	43.28	81.3	31	0.848
sp|Q14643|ITPR1_HUMAN	ITPR1	Inositol 1,4,5-trisphosphate receptor type 1	3.24	21.14	21.2	16	0.848
sp|P06730|IF4E_HUMAN	EIF4E	Eukaryotic translation initiation factor 4E	10.17	11	51.2	9	0.848
sp|Q6ZRP7|QSOX2_HUMAN	QSOX2	Sulfhydryl oxidase 2	24.77	24.86	49.3	17	0.848
sp|O75691|UTP20_HUMAN	UTP20	Small subunit processome component 20 homolog	116.06	119	42	68	0.848
sp|P49915|GUAA_HUMAN	GMPS	GMP synthase [glutamine-hydrolyzing]	76.27	78.79	75.2	51	0.848
sp|A6NNF4|ZN726_HUMAN	ZNF726	Zinc finger protein 726	1.61	2.1	15.9	2	0.848
sp|Q6ZRV2|FA83H_HUMAN	FAM83H	Protein FAM83H	13.81	14.27	19.1	8	0.849
sp|P53582|MAP11_HUMAN	METAP1	Methionine aminopeptidase 1	24.74	24.93	71.2	19	0.849
sp|P58546|MTPN_HUMAN	MTPN	Myotrophin	9.4	9.84	72.9	12	0.849
sp|Q9HBG6|IF122_HUMAN	IFT122	Intraflagellar transport protein 122 homolog	2.15	2.74	16.3	3	0.849
sp|Q9Y277|VDAC3_HUMAN	VDAC3	Voltage-dependent anion-selective channel protein 3	29.29	32.66	77.7	39	0.849
sp|Q86WJ1|CHD1L_HUMAN	CHD1L	Chromodomain-helicase-DNA-binding protein 1-like	37	39.3	42	20	0.849
sp|Q9NRX2|RM17_HUMAN	MRPL17	39S ribosomal protein L17, mitochondrial	13.83	15.73	49.7	11	0.849
sp|P31749|AKT1_HUMAN	AKT1	RAC-alpha serine/threonine-protein kinase	10.97	13.77	32.3	7	0.849
sp|Q9UEG4|ZN629_HUMAN	ZNF629	Zinc finger protein 629	3.12	5.24	15.3	4	0.849
sp|Q9Y2P8|RCL1_HUMAN	RCL1	RNA 3'-terminal phosphate cyclase-like protein	20.57	21.2	48.3	11	0.849
sp|P82912|RT11_HUMAN	MRPS11	28S ribosomal protein S11, mitochondrial	11.65	11.71	63.9	6	0.849
sp|P53609|PGTB1_HUMAN	PGGT1B	Geranylgeranyl transferase type-1 subunit beta	7.4	8.02	27.1	5	0.849
sp|Q9BRP1|PDD2L_HUMAN	PDCD2L	Programmed cell death protein 2-like	9.29	9.49	34.1	5	0.849
sp|O00231|PSD11_HUMAN	PSMD11	26S proteasome non-ATPase regulatory subunit 11	50.38	50.88	72.8	39	0.849
sp|P48507|GSH0_HUMAN	GCLM	Glutamate--cysteine ligase regulatory subunit	13.17	13.25	51.8	11	0.849
sp|P14927|QCR7_HUMAN	UQCRB	Cytochrome b-c1 complex subunit 7	12.81	14.52	82.9	11	0.850
sp|Q8IUW5|RELL1_HUMAN	RELL1	RELT-like protein 1	6.79	6.91	25.5	4	0.850
sp|Q14147|DHX34_HUMAN	DHX34	Probable ATP-dependent RNA helicase DHX34	6.71	7.35	21.8	7	0.850
sp|Q9H9Y6|RPA2_HUMAN	POLR1B	DNA-directed RNA polymerase I subunit RPA2	34.7	36.35	28.8	22	0.850
sp|P30419|NMT1_HUMAN	NMT1	Glycylpeptide N-tetradecanoyltransferase 1	36.3	38.29	52.8	31	0.850
sp|Q9NWS8|RMND1_HUMAN	RMND1	Required for meiotic nuclear division protein 1 homolog	10.75	10.83	31.2	9	0.850
sp|P0DMV9|HS71B_HUMAN	HSPA1B	Heat shock 70 kDa protein 1B	77.92	112.23	90.6	215	0.850
sp|P05023|AT1A1_HUMAN	ATP1A1	Sodium/potassium-transporting ATPase subunit alpha-1	106.11	106.2	57.4	141	0.851
sp|Q9Y4F1|FARP1_HUMAN	FARP1	FERM, RhoGEF and pleckstrin domain-containing protein 1	38.22	39.65	45.7	25	0.851
sp|Q9H0L4|CSTFT_HUMAN	CSTF2T	Cleavage stimulation factor subunit 2 tau variant	8.64	19.8	40.3	11	0.851
sp|Q9Y6Y8|S23IP_HUMAN	SEC23IP	SEC23-interacting protein	48.63	50	40	28	0.851
sp|O00161|SNP23_HUMAN	SNAP23	Synaptosomal-associated protein 23	21.83	21.89	78.2	17	0.851
sp|O60869|EDF1_HUMAN	EDF1	Endothelial differentiation-related factor 1	14.77	15.19	64.2	10	0.851
sp|P14735|IDE_HUMAN	IDE	Insulin-degrading enzyme	66.78	69.97	58.2	41	0.851
sp|Q5VWN6|F208B_HUMAN	FAM208B	Protein FAM208B	12.83	16.17	20.9	13	0.851

sp|P48066|S6A11_HUMAN	SLC6A11	Sodium- and chloride-dependent GABA transporter 3	1.73	2.08	13	4	0.851
sp|Q6P3X3|TTC27_HUMAN	TTC27	Tetratricopeptide repeat protein 27	19.37	20.89	32	14	0.851
sp|Q9P0U4|CXXC1_HUMAN	CXXC1	CXXC-type zinc finger protein 1	8.03	8.21	18.9	5	0.852
sp|O14763|TR10B_HUMAN	TNFRSF10B	Tumor necrosis factor receptor superfamily member 10B	7.24	7.32	29.1	5	0.852
sp|P00387|NB5R3_HUMAN	CYB5R3	NADH-cytochrome b5 reductase 3	40.12	40.7	77.4	39	0.852
sp|Q13613|MTMR1_HUMAN	MTMR1	Myotubularin-related protein 1	8.13	8.27	28.3	4	0.852
sp|P07199|CENPB_HUMAN	CENPB	Major centromere autoantigen B	19.23	19.72	36.2	10	0.852
sp|P54578|UBP14_HUMAN	USP14	Ubiquitin carboxyl-terminal hydrolase 14	43.56	44.16	69.6	32	0.852
sp|Q9HCJ0|TNR6C_HUMAN	TNRC6C	Trinucleotide repeat-containing gene 6C protein	2.01	3.32	8.5	2	0.852
sp|Q9UPW5|CBPC1_HUMAN	AGTPBP1	Cytosolic carboxypeptidase 1	10.07	12.34	18.8	9	0.853
sp|P78371|TCPB_HUMAN	CCT2	T-complex protein 1 subunit beta	98.12	98.16	85.6	118	0.853
sp|P28062|PSB8_HUMAN	PSMB8	Proteasome subunit beta type-8	7.1	7.22	33.3	6	0.853
sp|Q9UMS4|PRP19_HUMAN	PRPF19	Pre-mRNA-processing factor 19	36.39	36.86	83.1	64	0.853
sp|Q15365|PCBP1_HUMAN	PCBP1	Poly(rC)-binding protein 1	42.87	43.59	77.8	98	0.853
sp|Q9BRK5|CAB45_HUMAN	SDF4	45 kDa calcium-binding protein	20.39	21.69	62.4	23	0.853
sp|Q9UGV2|NDRG3_HUMAN	NDRG3	Protein NDRG3	8.71	8.9	32.3	8	0.853
sp|Q96EE3|SEH1_HUMAN	SEH1L	Nucleoporin SEH1	30.22	30.3	65.6	19	0.853
sp|Q93009|UBP7_HUMAN	USP7	Ubiquitin carboxyl-terminal hydrolase 7	86.32	86.94	63.6	49	0.853
sp|Q9BQ75|CMS1_HUMAN	CMSS1	Protein CMSS1	10.76	11.81	49.8	9	0.853
sp|O43505|B4GA1_HUMAN	B4GAT1	Beta-1,4-glucuronyltransferase 1	10.31	10.41	41.9	7	0.853
sp|Q9H1K1|ISCU_HUMAN	ISCU	Iron-sulfur cluster assembly enzyme ISCU, mitochondrial	6.96	7.15	56.3	5	0.853
sp|Q969F1|TF3C6_HUMAN	GTF3C6	General transcription factor 3C polypeptide 6	2.06	2.41	12.7	3	0.853
sp|P62913|RL11_HUMAN	RPL11	60S ribosomal protein L11	18.41	20.3	74.2	18	0.853
sp|Q9H4A5|GLP3L_HUMAN	GOLPH3L	Golgi phosphoprotein 3-like	2.95	7.3	37.5	7	0.853
sp|Q6P2P2|ANM9_HUMAN	PRMT9	Putative protein arginine N-methyltransferase 9	3.38	3.5	14.9	3	0.853
sp|P32969|RL9_HUMAN	RPL9	60S ribosomal protein L9	25.26	26.27	77.1	38	0.854
sp|O43347|MSI1H_HUMAN	MSI1	RNA-binding protein Musashi homolog 1	17.32	19.11	39.5	12	0.854
sp|O15397|IPO8_HUMAN	IPO8	Importin-8	16.6	18.99	30.5	17	0.854
sp|P68402|PA1B2_HUMAN	PAFAH1B2	Platelet-activating factor acetylhydrolase IB subunit beta	11.31	11.78	59.8	16	0.854
sp|P98179|RBM3_HUMAN	RBM3	RNA-binding protein 3	8.3	8.51	64.3	11	0.854
sp|Q9BWJ5|SF3B5_HUMAN	SF3B5	Splicing factor 3B subunit 5	8.24	9.17	65.1	5	0.854
sp|P41240|CSK_HUMAN	CSK	Tyrosine-protein kinase CSK	36.76	36.83	67.6	21	0.854
sp|A6NED2|RCCD1_HUMAN	RCCD1	RCC1 domain-containing protein 1	6.61	6.65	27.9	4	0.854
sp|O43808|PM34_HUMAN	SLC25A17	Peroxisomal membrane protein PMP34	8	8.06	27.4	6	0.854
sp|P09651|ROA1_HUMAN	HNRNPA1	Heterogeneous nuclear ribonucleoprotein A1	56.96	69.19	90.9	170	0.854
sp|O94854|K0754_HUMAN	KIAA0754	Uncharacterized protein KIAA0754	2.04	2.06	6	2	0.855
sp|P00558|PGK1_HUMAN	PGK1	Phosphoglycerate kinase 1	77.33	80.39	87.3	98	0.855
sp|Q9BYE7|PCGF6_HUMAN	PCGF6	Polycomb group RING finger protein 6	7.39	7.49	33.4	4	0.855
sp|Q8IU60|DCP2_HUMAN	DCP2	m7GpppN-mRNA hydrolase	8.88	8.96	28.1	5	0.855
sp|P78368|KC1G2_HUMAN	CSNK1G2	Casein kinase I isoform gamma-2	2.92	11.16	34.2	7	0.855
sp|P52272|HNRPM_HUMAN	HNRNPM	Heterogeneous nuclear ribonucleoprotein M	100.98	101.02	82.1	134	0.855
sp|P06493|CDK1_HUMAN	CDK1	Cyclin-dependent kinase 1	29.72	30.18	78.1	24	0.855
sp|Q15003|CND2_HUMAN	NCAPH	Condensin complex subunit 2	47.38	47.74	61.3	32	0.855
sp|Q8IYB7|DI3L2_HUMAN	DIS3L2	DIS3-like exonuclease 2	28.64	30.97	38.9	16	0.855
sp|O14579|COPE_HUMAN	COPE	Coatomer subunit epsilon	22.23	22.41	69.5	17	0.855
sp|Q15758|AAAT_HUMAN	SLC1A5	Neutral amino acid transporter B(0)	23.14	23.26	40.3	16	0.856
sp|Q9BW71|HIRP3_HUMAN	HIRIP3	HIRA-interacting protein 3	4.3	6.05	18.4	4	0.856
sp|Q8IZ81|ELMD2_HUMAN	ELMOD2	ELMO domain-containing protein 2	11.21	11.41	39.3	6	0.856
sp|Q92621|NU205_HUMAN	NUP205	Nuclear pore complex protein Nup205	86.79	88.89	40.1	54	0.856

sp|Q9UHA4|LTOR3_HUMAN	LAMTOR3	Ragulator complex protein LAMTOR3	2.22	2.27	51.6	2	0.856
sp|Q58A45|PAN3_HUMAN	PAN3	PAB-dependent poly(A)-specific ribonuclease subunit PAN3	3.8	3.9	10.6	4	0.856
sp|O15118|NPC1_HUMAN	NPC1	Niemann-Pick C1 protein	28.19	28.28	18.8	14	0.856
sp|P16278|BGAL_HUMAN	GLB1	Beta-galactosidase	26.24	27.72	36	16	0.856
sp|Q9ULX3|NOB1_HUMAN	NOB1	RNA-binding protein NOB1	21.61	21.79	44.4	13	0.856
sp|P41247|PLPL4_HUMAN	PNPLA4	Patatin-like phospholipase domain-containing protein 4	4.62	4.67	30.8	3	0.856
sp|Q70UQ0|IKIP_HUMAN	IKBIP	Inhibitor of nuclear factor kappa-B kinase-interacting protein	27.76	29.83	52.6	19	0.856
sp|Q8NHU6|TDRD7_HUMAN	TDRD7	Tudor domain-containing protein 7	8.48	10.57	20.7	6	0.856
sp|Q96AY3|FKB10_HUMAN	FKBP10	Peptidyl-prolyl cis-trans isomerase FKBP10	43.16	45.28	44.7	32	0.857
sp|P61764|STXB1_HUMAN	STXBP1	Syntaxin-binding protein 1	19.85	21.08	38.6	13	0.857
sp|Q7Z5L9|I2BP2_HUMAN	IRF2BP2	Interferon regulatory factor 2-binding protein 2	22.15	22.23	53.7	17	0.857
sp|Q3SXM5|HSDL1_HUMAN	HSDL1	Inactive hydroxysteroid dehydrogenase-like protein 1	21.42	21.82	49.4	11	0.857
sp|Q9BQ39|DDX50_HUMAN	DDX50	ATP-dependent RNA helicase DDX50	36.54	49.02	65.1	36	0.857
sp|Q9UMF0|ICAM5_HUMAN	ICAM5	Intercellular adhesion molecule 5	2.01	2.02	6.4	2	0.857
sp|Q12872|SFSWA_HUMAN	SFSWAP	Splicing factor, suppressor of white-apricot homolog	18.99	19.76	22	14	0.857
sp|Q9UK45|LSM7_HUMAN	LSM7	U6 snRNA-associated Sm-like protein LSm7	11.41	11.47	71.8	7	0.857
sp|O15355|PPM1G_HUMAN	PPM1G	Protein phosphatase 1G	44.67	44.68	59.3	35	0.857
sp|Q9BQS8|FYCO1_HUMAN	FYCO1	FYVE and coiled-coil domain-containing protein 1	20.39	23.16	29.5	13	0.857
sp|Q96D05|CJ035_HUMAN	C10orf35	Uncharacterized protein C10orf35	2.92	2.99	38	2	0.858
sp|Q9BQG2|NUD12_HUMAN	NUDT12	Peroxisomal NADH pyrophosphatase NUDT12	3.6	3.75	24.2	3	0.858
sp|Q9NPA8|ENY2_HUMAN	ENY2	Transcription and mRNA export factor ENY2	3.09	3.55	63.4	3	0.858
sp|Q9P015|RM15_HUMAN	MRPL15	39S ribosomal protein L15, mitochondrial	34.76	38.03	78	23	0.858
sp|O96000|NDUBA_HUMAN	NDUFB10	NADH dehydrogenase [ubiquinone] 1 beta subcomplex subunit 10	20.41	20.44	69.8	13	0.858
sp|P11117|PPAL_HUMAN	ACP2	Lysosomal acid phosphatase	14.04	16.05	28.8	10	0.858
sp|Q14974|IMB1_HUMAN	KPNB1	Importin subunit beta-1	66.5	66.6	59.5	88	0.858
sp|O60832|DKC1_HUMAN	DKC1	H/ACA ribonucleoprotein complex subunit 4	42.95	43.29	69.8	31	0.858
sp|Q9UDR5|AASS_HUMAN	AASS	Alpha-aminoadipic semialdehyde synthase, mitochondrial	49.06	50.05	51.3	34	0.858
sp|P63208|SKP1_HUMAN	SKP1	S-phase kinase-associated protein 1	18.98	19.63	92	21	0.858
sp|P51572|BAP31_HUMAN	BCAP31	B-cell receptor-associated protein 31	22.83	23.1	48.4	22	0.858
sp|O75448|MED24_HUMAN	MED24	Mediator of RNA polymerase II transcription subunit 24	16.01	16.26	23.4	11	0.859
sp|Q6NXT6|TAPT1_HUMAN	TAPT1	Transmembrane anterior posterior transformation protein 1 homolog	3	3.16	22.1	3	0.859
sp|Q9BVL2|NUPL1_HUMAN	NUPL1	Nucleoporin p58/p45	11.84	13.36	19.5	13	0.859
sp|Q8TDJ6|DMXL2_HUMAN	DMXL2	DmX-like protein 2	2.01	2.17	8.2	3	0.859
sp|O75369|FLNB_HUMAN	FLNB	Filamin-B	228.15	249.31	75.5	186	0.859
sp|Q9UQ53|MGT4B_HUMAN	MGAT4B	Alpha-1,3-mannosyl-glycoprotein 4-beta-N-acetylglucosaminyltransferase B	2.01	2.03	24.1	2	0.859
sp|P62306|RUXF_HUMAN	SNRPF	Small nuclear ribonucleoprotein F	6.2	6.29	70.9	7	0.860
sp|P30520|PURA2_HUMAN	ADSS	Adenylosuccinate synthetase isozyme 2	31.33	31.53	50.7	27	0.860
sp|Q9H3G5|CPVL_HUMAN	CPVL	Probable serine carboxypeptidase CPVL	16.87	17.1	36.6	10	0.860
sp|Q5SSJ5|HP1B3_HUMAN	HP1BP3	Heterochromatin protein 1-binding protein 3	34.73	35.06	49.6	21	0.860
sp|Q9NYF8|BCLF1_HUMAN	BCLAF1	Bcl-2-associated transcription factor 1	48.54	48.75	42.6	33	0.860
sp|Q9NX24|NHP2_HUMAN	NHP2	H/ACA ribonucleoprotein complex subunit 2	12.61	12.67	85.6	11	0.860
sp|Q15404|RSU1_HUMAN	RSU1	Ras suppressor protein 1	11.98	12.26	44.8	9	0.861
sp|Q9BTE3|MCMBP_HUMAN	MCMBP	Mini-chromosome maintenance complex-binding protein	36.47	36.76	46.1	23	0.861
sp|Q86SQ9|DHDDS_HUMAN	DHDDS	Dehydrodolichyl diphosphate syntase complex subunit DHDDS	6.95	7.06	35.1	4	0.861
sp|Q99747|SNAG_HUMAN	NAPG	Gamma-soluble NSF attachment protein	16.95	17.13	55.8	10	0.861
sp|Q96P11|NSUN5_HUMAN	NSUN5	Probable 28S rRNA (cytosine-C(5))-methyltransferase	29.14	29.51	61.5	18	0.861
sp|P47756|CAPZB_HUMAN	CAPZB	F-actin-capping protein subunit beta	31.93	32.42	75.8	28	0.861
sp|Q86UP2|KTN1_HUMAN	KTN1	Kinectin	151.54	153.22	69.3	92	0.861
sp|Q9GZU7|CTDS1_HUMAN	CTDSP1	Carboxy-terminal domain RNA polymerase II polypeptide A small phosphatase 1	2.4	2.45	14.2	3	0.861

sp|P36639|8ODP_HUMAN	NUDT1	7,8-dihydro-8-oxoguanine triphosphatase	4.01	4.03	29.4	2	0.861
sp|P63010|AP2B1_HUMAN	AP2B1	AP-2 complex subunit beta	72.44	72.6	53.6	61	0.861
sp|Q9UJX2|CDC23_HUMAN	CDC23	Cell division cycle protein 23 homolog	22.46	24.14	44.6	15	0.861
sp|Q9H0U9|TSYL1_HUMAN	TSPYL1	Testis-specific Y-encoded-like protein 1	8.01	8.03	25.6	6	0.861
sp|Q15029|U5S1_HUMAN	EFTUD2	116 kDa U5 small nuclear ribonucleoprotein component	90.16	93.27	68.4	76	0.861
sp|Q4G176|ACSF3_HUMAN	ACSF3	Acyl-CoA synthetase family member 3, mitochondrial	17.17	18.21	49	10	0.861
sp|Q9NWQ8|PHAG1_HUMAN	PAG1	Phosphoprotein associated with glycosphingolipid-enriched microdomains 1	3.76	3.84	16.9	2	0.861
sp|O95793|STAU1_HUMAN	STAU1	Double-stranded RNA-binding protein Staufen homolog 1	43.4	44.29	61.9	32	0.861
sp|Q8N1B4|VPS52_HUMAN	VPS52	Vacuolar protein sorting-associated protein 52 homolog	13.15	13.59	35.6	9	0.862
sp|P49588|SYAC_HUMAN	AARS	Alanine--tRNA ligase, cytoplasmic	101.53	102.04	76.8	94	0.862
sp|Q13395|TARB1_HUMAN	TARBP1	Probable methyltransferase TARBP1	14.14	14.26	18.9	7	0.862
sp|Q96CW6|S7A6O_HUMAN	SLC7A6OS	Probable RNA polymerase II nuclear localization protein SLC7A6	4	4.01	13.9	3	0.862
sp|Q9UKD1|GMEB2_HUMAN	GMEB2	Glucocorticoid modulatory element-binding protein 2	2.05	2.07	14.7	2	0.862
sp|P35869|AHR_HUMAN	AHR	Aryl hydrocarbon receptor	2.49	4.01	5.7	2	0.862
sp|Q9P003|CNIH4_HUMAN	CNIH4	Protein cornichon homolog 4	2	2	18	4	0.862
sp|Q13144|EI2BE_HUMAN	EIF2B5	Translation initiation factor eIF-2B subunit epsilon	25.7	25.89	38.6	21	0.862
sp|P16870|CBPE_HUMAN	CPE	Carboxypeptidase E	3.77	3.94	18.9	3	0.862
sp|Q9Y692|GMEB1_HUMAN	GMEB1	Glucocorticoid modulatory element-binding protein 1	2.12	2.7	14.7	2	0.862
sp|O00468|AGRIN_HUMAN	AGRN	Agrin	31.25	31.98	18.3	15	0.862
sp|P27694|RFA1_HUMAN	RPA1	Replication protein A 70 kDa DNA-binding subunit	58.54	58.57	71.1	38	0.862
sp|Q5VV42|CDKAL_HUMAN	CDKAL1	Threonylcarbamoyladenosine tRNA methylthiotransferase	21.63	22.65	44.2	15	0.863
sp|P61916|NPC2_HUMAN	NPC2	Epididymal secretory protein E1	11.3	11.42	58.3	10	0.863
sp|Q9UNA4|POLI_HUMAN	POLI	DNA polymerase iota	2.01	2.02	10.4	2	0.863
sp|Q92599|SEPT8_HUMAN	SEPT8	Septin-8	8.09	14.2	29.2	9	0.863
sp|Q13526|PIN1_HUMAN	PIN1	Peptidyl-prolyl cis-trans isomerase NIMA-interacting 1	10.74	11.29	60.1	8	0.863
sp|P14550|AK1A1_HUMAN	AKR1A1	Alcohol dehydrogenase [NADP(+)]	36.59	40.23	76	29	0.863
sp|P62140|PP1B_HUMAN	PPP1CB	Serine/threonine-protein phosphatase PP1-beta catalytic subunit	15.69	42.38	81.4	33	0.863
sp|Q9C005|DPY30_HUMAN	DPY30	Protein dpy-30 homolog	9.3	9.4	89.9	6	0.863
sp|O14662|STX16_HUMAN	STX16	Syntaxin-16	8.02	8.02	40.6	4	0.863
sp|Q9Y644|RFNG_HUMAN	RFNG	Beta-1,3-N-acetylglucosaminyltransferase radical fringe	4	4.01	23.6	2	0.863
sp|P53803|RPAB4_HUMAN	POLR2K	DNA-directed RNA polymerases I, II, and III subunit RPABC4	2.27	2.3	60.3	2	0.863
sp|P29992|GNA11_HUMAN	GNA11	Guanine nucleotide-binding protein subunit alpha-11	12.81	15.98	53.2	10	0.864
sp|Q15459|SF3A1_HUMAN	SF3A1	Splicing factor 3A subunit 1	70.15	70.22	52.7	54	0.864
sp|Q96ER9|CCD51_HUMAN	CCDC51	Coiled-coil domain-containing protein 51	18.05	18.19	36	11	0.864
sp|Q1KMD3|HNRL2_HUMAN	HNRNPUL2	Heterogeneous nuclear ribonucleoprotein U-like protein 2	57.81	59.03	51.9	40	0.864
sp|A0PK00|T120B_HUMAN	TMEM120B	Transmembrane protein 120B	3.78	3.88	24.2	2	0.865
sp|Q9NXK8|FXL12_HUMAN	FBXL12	F-box/LRR-repeat protein 12	11.21	11.35	43.6	7	0.865
sp|P48200|IREB2_HUMAN	IREB2	Iron-responsive element-binding protein 2	5.84	6.2	22.9	5	0.865
sp|Q659C4|LAR1B_HUMAN	LARP1B	La-related protein 1B	4.95	10.95	21.2	7	0.865
sp|P57772|SELB_HUMAN	EEFSEC	Selenocysteine-specific elongation factor	20.81	20.94	35.9	13	0.865
sp|Q53T59|H1BP3_HUMAN	HS1BP3	HCLS1-binding protein 3	15.57	15.63	40.1	9	0.865
sp|O43447|PPIH_HUMAN	PPIH	Peptidyl-prolyl cis-trans isomerase H	19.99	21.44	90.4	20	0.865
sp|Q9NRH2|SNRK_HUMAN	SNRK	SNF-related serine/threonine-protein kinase	2	2.02	8.8	2	0.866
sp|P35813|PPM1A_HUMAN	PPM1A	Protein phosphatase 1A	12.76	18.89	47.1	13	0.866
sp|O75145|LIPA3_HUMAN	PPFIA3	Liprin-alpha-3	1.36	5.92	21.3	5	0.866
sp|Q01130|SRSF2_HUMAN	SRSF2	Serine/arginine-rich splicing factor 2	11.95	12.06	32.6	21	0.866
sp|Q92574|TSC1_HUMAN	TSC1	Hamartin	3.03	3.27	13.5	4	0.866
sp|Q9UKV3|ACINU_HUMAN	ACIN1	Apoptotic chromatin condensation inducer in the nucleus	66.62	66.95	47.2	40	0.866
sp|P48029|SC6A8_HUMAN	SLC6A8	Sodium- and chloride-dependent creatine transporter 1	3.28	3.47	9.9	3	0.866

sp|P03905|NU4M_HUMAN	MT-ND4	NADH-ubiquinone oxidoreductase chain 4	6	6	13.3	3	0.866
sp|Q99595|TI17A_HUMAN	TIMM17A	Mitochondrial import inner membrane translocase subunit Tim17-A	2.05	4.06	33.9	2	0.866
sp|Q9NUL3|STAU2_HUMAN	STAU2	Double-stranded RNA-binding protein Staufen homolog 2	8.75	13.44	40	8	0.866
sp|P19388|RPAB1_HUMAN	POLR2E	DNA-directed RNA polymerases I, II, and III subunit RPABC1	12.95	13.36	67.6	12	0.866
sp|Q92896|GSLG1_HUMAN	GLG1	Golgi apparatus protein 1	94.02	95.66	53.9	67	0.866
sp|Q15643|TRIPB_HUMAN	TRIP11	Thyroid receptor-interacting protein 11	25.58	34.41	38.8	27	0.866
sp|Q8WUM4|PDC6I_HUMAN	PDCD6IP	Programmed cell death 6-interacting protein	87.49	87.52	69	53	0.867
sp|Q86V87|F16B2_HUMAN	FAM160B2	Protein FAM160B2	1.63	4.18	11.8	4	0.867
sp|Q96C01|F136A_HUMAN	FAM136A	Protein FAM136A	13.3	14.52	69.6	10	0.867
sp|O75475|PSIP1_HUMAN	PSIP1	PC4 and SFRS1-interacting protein	42.15	42.35	60.6	26	0.867
sp|P49585|PCY1A_HUMAN	PCYT1A	Choline-phosphate cytidylyltransferase A	22.54	22.68	62.4	11	0.867
sp|P38646|GRP75_HUMAN	HSPA9	Stress-70 protein, mitochondrial	124.84	126.7	80.6	181	0.867
sp|Q86VI3|IQGA3_HUMAN	IQGAP3	Ras GTPase-activating-like protein IQGAP3	24.55	33.28	33.8	21	0.867
sp|Q96I99|SUCB2_HUMAN	SUCLG2	Succinyl-CoA ligase [GDP-forming] subunit beta, mitochondrial	46.7	49.42	75.9	38	0.867
sp|Q6PJG6|BRAT1_HUMAN	BRAT1	BRCA1-associated ATM activator 1	19.53	22.06	32.9	12	0.868
sp|Q7Z4H3|HDDC2_HUMAN	HDDC2	HD domain-containing protein 2	14.58	14.73	79.9	12	0.868
sp|P02771|FETA_HUMAN	AFP	Alpha-fetoprotein	49.12	50.05	72.1	33	0.868
sp|P24941|CDK2_HUMAN	CDK2	Cyclin-dependent kinase 2	24.98	29.52	62.1	16	0.868
sp|P62701|RS4X_HUMAN	RPS4X	40S ribosomal protein S4, X isoform	49.74	51.04	74.5	49	0.868
sp|O95163|ELP1_HUMAN	IKBKAP	Elongator complex protein 1	70.67	71.22	49	42	0.868
sp|P14649|MYL6B_HUMAN	MYL6B	Myosin light chain 6B	4.52	9.49	63	7	0.868
sp|Q8WTS1|ABHD5_HUMAN	ABHD5	1-acylglycerol-3-phosphate O-acyltransferase ABHD5	3.3	3.4	17.8	2	0.868
sp|Q9Y5X1|SNX9_HUMAN	SNX9	Sorting nexin-9	24.15	24.25	54.3	17	0.868
sp|O14787|TNPO2_HUMAN	TNPO2	Transportin-2	12.05	27.22	28.9	23	0.868
sp|P14678|RSMB_HUMAN	SNRPB	Small nuclear ribonucleoprotein-associated proteins B and B'	15.75	15.82	50.4	21	0.868
sp|Q8WVC6|DCAKD_HUMAN	DCAKD	Dephospho-CoA kinase domain-containing protein	13.42	13.49	58.9	8	0.868
sp|Q96KC8|DNJC1_HUMAN	DNAJC1	DnaJ homolog subfamily C member 1	4.99	7.08	24.6	4	0.868
sp|Q99570|PI3R4_HUMAN	PIK3R4	Phosphoinositide 3-kinase regulatory subunit 4	22.9	26.62	26.4	17	0.868
sp|Q9P206|K1522_HUMAN	KIAA1522	Uncharacterized protein KIAA1522	5.57	5.8	13.2	5	0.869
sp|P23470|PTPRG_HUMAN	PTPRG	Receptor-type tyrosine-protein phosphatase gamma	3.45	3.61	12.4	3	0.869
sp|Q8N3J9|ZN664_HUMAN	ZNF664	Zinc finger protein 664	2	2.02	17.6	2	0.869
sp|O75165|DJC13_HUMAN	DNAJC13	DnaJ homolog subfamily C member 13	58.17	61.25	31.1	33	0.869
sp|Q96K58|ZN668_HUMAN	ZNF668	Zinc finger protein 668	2.01	2.17	10	2	0.869
sp|O15182|CETN3_HUMAN	CETN3	Centrin-3	5.68	7.19	32.9	6	0.869
sp|P49458|SRP09_HUMAN	SRP9	Signal recognition particle 9 kDa protein	9.21	9.65	76.7	13	0.869
sp|Q13151|ROA0_HUMAN	HNRNPA0	Heterogeneous nuclear ribonucleoprotein A0	22.27	27.98	63.3	38	0.869
sp|Q13838|DX39B_HUMAN	DDX39B	Spliceosome RNA helicase DDX39B	57.12	58.97	76.4	69	0.869
sp|Q9H845|ACAD9_HUMAN	ACAD9	Acyl-CoA dehydrogenase family member 9, mitochondrial	41.92	42.3	63.5	26	0.869
sp|Q9NWS6|F118A_HUMAN	FAM118A	Protein FAM118A	3.64	3.82	26.9	3	0.870
sp|Q6PGN9|PSRC1_HUMAN	PSRC1	Proline/serine-rich coiled-coil protein 1	1.92	2	12.7	2	0.870
sp|Q3T906|GNPTA_HUMAN	GNPTAB	N-acetylglucosamine-1-phosphotransferase subunits alpha/beta	4.06	4.13	15.1	5	0.870
sp|Q9UBC3|DNM3B_HUMAN	DNMT3B	DNA (cytosine-5)-methyltransferase 3B	4.3	5.24	13.7	3	0.870
sp|P26440|IVD_HUMAN	IVD	Isovaleryl-CoA dehydrogenase, mitochondrial	27.19	28.05	48.7	17	0.870
sp|Q9Y281|COF2_HUMAN	CFL2	Cofilin-2	16.56	29.84	97	29	0.870
sp|Q8WUB8|PHF10_HUMAN	PHF10	PHD finger protein 10	8.09	8.26	24.1	6	0.870
sp|Q5H9R7|PP6R3_HUMAN	PPP6R3	Serine/threonine-protein phosphatase 6 regulatory subunit 3	31.25	36.42	38.6	24	0.870
sp|Q6PL24|TMED8_HUMAN	TMED8	Protein TMED8	4.81	5.1	25.2	4	0.870
sp|P11171|41_HUMAN	EPB41	Protein 4.1	46.75	47.65	55.3	27	0.871
sp|Q9Y6X8|ZHX2_HUMAN	ZHX2	Zinc fingers and homeoboxes protein 2	10.96	11.77	24.9	9	0.871

sp|P02753|RET4_HUMAN	RBP4	Retinol-binding protein 4	17.96	18	57.2	11	0.871
sp|Q15121|PEA15_HUMAN	PEA15	Astrocytic phosphoprotein PEA-15	2.83	3.67	56.9	6	0.871
sp|Q15555|MARE2_HUMAN	MAPRE2	Microtubule-associated protein RP/EB family member 2	16.64	18.82	62.7	13	0.871
sp|Q16563|SYPL1_HUMAN	SYPL1	Synaptophysin-like protein 1	3.72	3.96	16.6	2	0.871
sp|P84098|RL19_HUMAN	RPL19	60S ribosomal protein L19	19.34	21.96	56.1	26	0.872
sp|Q15814|TBCC_HUMAN	TBCC	Tubulin-specific chaperone C	17.41	17.77	73.1	10	0.872
sp|Q8IWR0|Z3H7A_HUMAN	ZC3H7A	Zinc finger CCCH domain-containing protein 7A	16.43	17.18	33.4	9	0.872
sp|Q147X3|NAA30_HUMAN	NAA30	N-alpha-acetyltransferase 30	13.66	13.73	31.5	8	0.872
sp|Q01813|PFKAP_HUMAN	PFKP	ATP-dependent 6-phosphofructokinase, platelet type	34.4	41.13	46.1	26	0.872
sp|P23786|CPT2_HUMAN	CPT2	Carnitine O-palmitoyltransferase 2, mitochondrial	41.55	41.93	51.8	24	0.872
sp|Q96DG6|CMBL_HUMAN	CMBL	Carboxymethylenebutenolidase homolog	13.88	15.26	60.4	11	0.872
sp|Q86UK7|ZN598_HUMAN	ZNF598	Zinc finger protein 598	23.26	25.59	26.8	14	0.872
sp|Q96N21|AP4AT_HUMAN	ENTHD2	AP-4 complex accessory subunit tepsin	2.1	2.21	24.2	3	0.872
sp|Q9HB07|MYG1_HUMAN	C12orf10	UPF0160 protein MYG1, mitochondrial	22.66	23.28	56.7	17	0.872
sp|Q8TER5|ARH40_HUMAN	ARHGEF40	Rho guanine nucleotide exchange factor 40	7.79	8.05	19	5	0.873
sp|P24539|AT5F1_HUMAN	ATP5F1	ATP synthase F(0) complex subunit B1, mitochondrial	23.48	25.51	64.8	25	0.873
sp|Q6ZN55|ZN574_HUMAN	ZNF574	Zinc finger protein 574	2.71	2.77	9.9	2	0.873
sp|Q9NXG2|THUM1_HUMAN	THUMPD1	THUMP domain-containing protein 1	24.72	25.41	55.2	18	0.873
sp|Q8IWU2|LMTK2_HUMAN	LMTK2	Serine/threonine-protein kinase LMTK2	2.02	2.03	8.1	3	0.873
sp|Q8IUH3|RBM45_HUMAN	RBM45	RNA-binding protein 45	15.98	16.77	39.9	9	0.874
sp|P35269|T2FA_HUMAN	GTF2F1	General transcription factor IIF subunit 1	24.07	24.44	46.4	18	0.874
sp|P21796|VDAC1_HUMAN	VDAC1	Voltage-dependent anion-selective channel protein 1	58.9	58.94	93.6	99	0.874
sp|P42166|LAP2A_HUMAN	TMPO	Lamina-associated polypeptide 2, isoform alpha	62.22	64.64	71.9	59	0.874
sp|Q9NRX5|SERC1_HUMAN	SERINC1	Serine incorporator 1	5.86	5.95	13.9	3	0.874
sp|P15907|SIAT1_HUMAN	ST6GAL1	Beta-galactoside alpha-2,6-sialyltransferase 1	8.42	8.68	27.3	8	0.874
sp|Q96A54|ADR1_HUMAN	ADIPOR1	Adiponectin receptor protein 1	4.27	4.32	14.9	4	0.874
sp|P08651|NFIC_HUMAN	NFIC	Nuclear factor 1 C-type	12.64	12.9	31.1	7	0.875
sp|Q9C075|K1C23_HUMAN	KRT23	Keratin, type I cytoskeletal 23	7.89	8.08	45	8	0.875
sp|Q9BPX5|ARP5L_HUMAN	ARPC5L	Actin-related protein 2/3 complex subunit 5-like protein	6.23	8.56	63.4	6	0.875
sp|P78383|S35B1_HUMAN	SLC35B1	Solute carrier family 35 member B1	4.01	4.01	16.5	2	0.875
sp|Q99729|ROAA_HUMAN	HNRNPAB	Heterogeneous nuclear ribonucleoprotein A/B	26.02	30.13	56.3	38	0.875
sp|P33897|ABCD1_HUMAN	ABCD1	ATP-binding cassette sub-family D member 1	14.32	15.73	30.3	9	0.875
sp|Q9H0R4|HDHD2_HUMAN	HDHD2	Haloacid dehalogenase-like hydrolase domain-containing protein 2	8.22	8.31	63.3	4	0.875
sp|Q9H1A4|APC1_HUMAN	ANAPC1	Anaphase-promoting complex subunit 1	32.77	33.71	22.1	17	0.875
sp|P61289|PSME3_HUMAN	PSME3	Proteasome activator complex subunit 3	30.34	32.28	74.8	23	0.875
sp|P04626|ERBB2_HUMAN	ERBB2	Receptor tyrosine-protein kinase erbB-2	17.11	18.06	22.4	12	0.875
sp|Q8WZA1|PMGT1_HUMAN	POMGNT1	Protein O-linked-mannose beta-1,2-N-acetylglucosaminyltransferase 1	7.42	7.52	17.4	5	0.875
sp|P61163|ACTZ_HUMAN	ACTR1A	Alpha-centractin	26.58	30.34	67	29	0.876
sp|Q86T03|TM55B_HUMAN	TMEM55B	Type 1 phosphatidylinositol 4,5-bisphosphate 4-phosphatase	6.01	6.02	30.3	4	0.876
sp|Q6PGP7|TTC37_HUMAN	TTC37	Tetratricopeptide repeat protein 37	49.46	52.55	35	35	0.876
sp|O14618|CCS_HUMAN	CCS	Copper chaperone for superoxide dismutase	8.48	8.86	30.3	5	0.876
sp|Q96DH6|MSI2H_HUMAN	MSI2	RNA-binding protein Musashi homolog 2	10.48	16.22	41.5	11	0.876
sp|Q96HP4|OXND1_HUMAN	OXNAD1	Oxidoreductase NAD-binding domain-containing protein 1	7.73	7.94	42	4	0.876
sp|P09001|RM03_HUMAN	MRPL3	39S ribosomal protein L3, mitochondrial	23.85	24.13	55.2	17	0.876
sp|Q02952|AKA12_HUMAN	AKAP12	A-kinase anchor protein 12	89.75	89.86	53.9	54	0.876
sp|Q9Y597|KCTD3_HUMAN	KCTD3	BTB/POZ domain-containing protein KCTD3	6.58	6.97	23.7	6	0.876
sp|Q96CP2|FWCH2_HUMAN	FLYWCH2	FLYWCH family member 2	7.67	7.76	57.9	4	0.877
sp|O60830|TI17B_HUMAN	TIMM17B	Mitochondrial import inner membrane translocase subunit Tim17-B	9.57	9.64	47.1	6	0.877
sp|Q712K3|UB2R2_HUMAN	UBE2R2	Ubiquitin-conjugating enzyme E2 R2	7.15	7.45	31.1	6	0.877

sp|Q8N0X7|SPG20_HUMAN	SPG20	Spartin	17.98	19.39	36.8	14	0.877
sp|Q9NUJ1|ABHDA_HUMAN	ABHD10	Mycophenolic acid acyl-glucuronide esterase, mitochondrial	31.18	31.28	60.8	23	0.877
sp|Q9NVR5|KTU_HUMAN	DNAAF2	Protein kintoun	2.01	2.03	11	2	0.877
sp|P34896|GLYC_HUMAN	SHMT1	Serine hydroxymethyltransferase, cytosolic	24.85	27.37	57.6	24	0.877
sp|P63218|GBG5_HUMAN	GNG5	Guanine nucleotide-binding protein G(I)/G(S)/G(O) subunit gamma-5	3.97	4.14	38.2	6	0.877
sp|P04350|TBB4A_HUMAN	TUBB4A	Tubulin beta-4A chain	8	80	84.7	285	0.877
sp|Q6VN20|RBP10_HUMAN	RANBP10	Ran-binding protein 10	9.1	14	30.8	12	0.878
sp|Q13405|RM49_HUMAN	MRPL49	39S ribosomal protein L49, mitochondrial	7.91	9.37	65.1	10	0.878
sp|Q9BRR8|GPTC1_HUMAN	GPATCH1	G patch domain-containing protein 1	5.04	5.11	11.2	3	0.878
sp|P85037|FOXK1_HUMAN	FOXK1	Forkhead box protein K1	17.59	18.73	25.4	10	0.878
sp|Q8N5F7|NKAP_HUMAN	NKAP	NF-kappa-B-activating protein	3.57	4.09	25.3	4	0.878
sp|P83111|LACTB_HUMAN	LACTB	Serine beta-lactamase-like protein LACTB, mitochondrial	15.71	15.87	36.9	9	0.878
sp|Q9H0A0|NAT10_HUMAN	NAT10	N-acetyltransferase 10	88.14	88.62	66.1	66	0.878
sp|P20936|RASA1_HUMAN	RASA1	Ras GTPase-activating protein 1	15.59	15.91	27.5	11	0.878
sp|Q9NPI1|BRD7_HUMAN	BRD7	Bromodomain-containing protein 7	8.01	8.54	21.8	5	0.878
sp|Q96S55|WRIP1_HUMAN	WRNIP1	ATPase WRNIP1	32	32.2	36.1	16	0.878
sp|O00330|ODPX_HUMAN	PDHX	Pyruvate dehydrogenase protein X component, mitochondrial	29.83	30.11	66.3	22	0.878
sp|P11802|CDK4_HUMAN	CDK4	Cyclin-dependent kinase 4	15.66	18.07	69	10	0.879
sp|P39060|COIA1_HUMAN	COL18A1	Collagen alpha-1(XVIII) chain	6.12	8.45	9.5	5	0.879
sp|Q13795|ARFRP_HUMAN	ARFRP1	ADP-ribosylation factor-related protein 1	9.49	10.17	59.2	6	0.879
sp|Q99426|TBCB_HUMAN	TBCB	Tubulin-folding cofactor B	14.45	14.52	66.4	8	0.879
sp|Q9UGM6|SYWM_HUMAN	WARS2	Tryptophan--tRNA ligase, mitochondrial	19.39	19.63	52.8	11	0.879
sp|Q8NE86|MCU_HUMAN	MCU	Calcium uniporter protein, mitochondrial	18.62	18.79	45.6	10	0.879
sp|Q9Y282|ERGI3_HUMAN	ERGIC3	Endoplasmic reticulum-Golgi intermediate compartment protein 3	13.7	14.17	36.6	7	0.879
sp|Q6UW68|TM205_HUMAN	TMEM205	Transmembrane protein 205	6.02	6.03	29.6	5	0.879
sp|P13473|LAMP2_HUMAN	LAMP2	Lysosome-associated membrane glycoprotein 2	6.35	6.39	20	6	0.880
sp|Q9Y4C2|TCAF1_HUMAN	TCAF1	TRPM8 channel-associated factor 1	21	21.56	27.9	13	0.880
sp|Q9ULW3|ABT1_HUMAN	ABT1	Activator of basal transcription 1	11.39	13.35	39.7	8	0.880
sp|P54252|ATX3_HUMAN	ATXN3	Ataxin-3	3.96	4.01	13.7	2	0.880
sp|Q9BYD6|RM01_HUMAN	MRPL1	39S ribosomal protein L1, mitochondrial	25.15	25.28	57.5	24	0.880
sp|Q9NUI1|DECR2_HUMAN	DECR2	Peroxisomal 2,4-dienoyl-CoA reductase	6.58	7.67	44.9	6	0.880
sp|Q9Y3I0|RTCB_HUMAN	RTCB	tRNA-splicing ligase RtcB homolog	52.77	52.82	73.9	48	0.880
sp|P09936|UCHL1_HUMAN	UCHL1	Ubiquitin carboxyl-terminal hydrolase isozyme L1	20.36	21.61	81.2	21	0.880
sp|Q9BRG1|VPS25_HUMAN	VPS25	Vacuolar protein-sorting-associated protein 25	13.77	13.82	67.6	8	0.880
sp|Q9H3H3|CK068_HUMAN	C11orf68	UPF0696 protein C11orf68	8	8	23.5	4	0.881
sp|Q13257|MD2L1_HUMAN	MAD2L1	Mitotic spindle assembly checkpoint protein MAD2A	9.38	10.78	56.6	9	0.881
sp|Q14790|CASP8_HUMAN	CASP8	Caspase-8	17.09	17.43	44.9	10	0.881
sp|Q99575|POP1_HUMAN	POP1	Ribonucleases P/MRP protein subunit POP1	40.43	41.63	49	29	0.881
sp|Q96QK1|VPS35_HUMAN	VPS35	Vacuolar protein sorting-associated protein 35	58.3	60.41	52.8	39	0.882
sp|P49189|AL9A1_HUMAN	ALDH9A1	4-trimethylaminobutyraldehyde dehydrogenase	52.23	55.09	68.6	42	0.882
sp|Q9UNE7|CHIP_HUMAN	STUB1	E3 ubiquitin-protein ligase CHIP	26.25	27.09	70.3	17	0.882
sp|Q9Y2X9|ZN281_HUMAN	ZNF281	Zinc finger protein 281	7.03	7.17	19.6	4	0.882
sp|Q9Y2T2|AP3M1_HUMAN	AP3M1	AP-3 complex subunit mu-1	25.1	25.2	58.9	16	0.882
sp|Q14554|PDIA5_HUMAN	PDIA5	Protein disulfide-isomerase A5	31.84	34.08	56.8	21	0.882
sp|P31323|KAP3_HUMAN	PRKAR2B	cAMP-dependent protein kinase type II-beta regulatory subunit	8.72	14.7	40.4	9	0.882
sp|Q9NRG4|SMYD2_HUMAN	SMYD2	N-lysine methyltransferase SMYD2	4.17	4.29	32.1	2	0.882
sp|P49207|RL34_HUMAN	RPL34	60S ribosomal protein L34	10.59	10.68	59.8	8	0.882
sp|P00505|AATM_HUMAN	GOT2	Aspartate aminotransferase, mitochondrial	59.02	59.15	83	64	0.882
sp|Q70Z53|F10C1_HUMAN	FRA10AC1	Protein FRA10AC1	3.45	3.59	27.9	3	0.883

sp|Q8WUW1|BRK1_HUMAN	BRK1	Protein BRICK1	6.02	6.52	72	4	0.883
sp|Q9Y3A3|PHOCN_HUMAN	MOB4	MOB-like protein phocein	18.47	18.5	77.3	13	0.883
sp|Q8TDX7|NEK7_HUMAN	NEK7	Serine/threonine-protein kinase Nek7	5.75	6.01	30.8	4	0.883
sp|Q96TA2|YMEL1_HUMAN	YME1L1	ATP-dependent zinc metalloprotease YME1L1	36.73	37.11	47.4	23	0.883
sp|P49459|UBE2A_HUMAN	UBE2A	Ubiquitin-conjugating enzyme E2 A	6	6	50	3	0.883
sp|Q9Y2L9|LRCH1_HUMAN	LRCH1	Leucine-rich repeat and calponin homology domain-containing protein 1	7.94	9.2	18.1	5	0.883
sp|Q16543|CDC37_HUMAN	CDC37	Hsp90 co-chaperone Cdc37	34.01	34.42	60.9	26	0.883
sp|Q08379|GOGA2_HUMAN	GOLGA2	Golgin subfamily A member 2	50.05	50.53	47.6	24	0.883
sp|Q99627|CSN8_HUMAN	COPS8	COP9 signalosome complex subunit 8	15	15.07	82.8	13	0.883
sp|P43034|LIS1_HUMAN	PAFAH1B1	Platelet-activating factor acetylhydrolase IB subunit alpha	31.82	33.49	61	22	0.883
sp|P62993|GRB2_HUMAN	GRB2	Growth factor receptor-bound protein 2	21.98	22.43	80.2	18	0.883
sp|Q96J01|THOC3_HUMAN	THOC3	THO complex subunit 3	31.4	32.33	66.1	19	0.883
sp|O60739|EIF1B_HUMAN	EIF1B	Eukaryotic translation initiation factor 1b	6	17.98	83.2	10	0.883
sp|Q14247|SRC8_HUMAN	CTTN	Src substrate cortactin	40.24	40.55	53.1	28	0.883
sp|Q96MW1|CCD43_HUMAN	CCDC43	Coiled-coil domain-containing protein 43	8.21	8.24	51.3	5	0.884
sp|P19634|SL9A1_HUMAN	SLC9A1	Sodium/hydrogen exchanger 1	2.05	2.16	15.5	2	0.884
sp|P35251|RFC1_HUMAN	RFC1	Replication factor C subunit 1	44.01	45.14	41.4	25	0.884
sp|P62273|RS29_HUMAN	RPS29	40S ribosomal protein S29	4.7	6.73	28.6	4	0.884
sp|O75496|GEMI_HUMAN	GMNN	Geminin	10.23	10.36	44	6	0.884
sp|P56192|SYMC_HUMAN	MARS	Methionine--tRNA ligase, cytoplasmic	77.5	79.73	59.6	58	0.884
sp|P86791|CCZ1_HUMAN	CCZ1	Vacuolar fusion protein CCZ1 homolog	4.59	4.98	34.9	4	0.884
sp|Q92499|DDX1_HUMAN	DDX1	ATP-dependent RNA helicase DDX1	82.78	82.96	72	54	0.884
sp|O00221|IKBE_HUMAN	NFKBIE	NF-kappa-B inhibitor epsilon	6.51	6.64	24	4	0.884
sp|Q8NFZ8|CADM4_HUMAN	CADM4	Cell adhesion molecule 4	4.16	4.26	11.6	3	0.884
sp|Q9NQ88|TIGAR_HUMAN	TIGAR	Fructose-2,6-bisphosphatase TIGAR	21.16	21.23	75.6	12	0.885
sp|Q15025|TNIP1_HUMAN	TNIP1	TNFAIP3-interacting protein 1	1.42	1.75	12.3	2	0.885
sp|P09525|ANXA4_HUMAN	ANXA4	Annexin A4	53.14	55.32	82.5	47	0.885
sp|O15523|DDX3Y_HUMAN	DDX3Y	ATP-dependent RNA helicase DDX3Y	5.31	64.26	65.2	46	0.885
sp|Q9HB58|SP110_HUMAN	SP110	Sp110 nuclear body protein	2.02	2.05	10	2	0.885
sp|Q9NZJ9|NUDT4_HUMAN	NUDT4	Diphosphoinositol polyphosphate phosphohydrolase 2	8.55	10	55.6	6	0.885
sp|Q96KQ4|ASPP1_HUMAN	PPP1R13B	Apoptosis-stimulating of p53 protein 1	2.54	2.61	11.7	3	0.885
sp|Q9NQZ5|STAR7_HUMAN	STARD7	StAR-related lipid transfer protein 7, mitochondrial	6.63	7.15	31.4	5	0.885
sp|O75347|TBCA_HUMAN	TBCA	Tubulin-specific chaperone A	18.92	19.05	74.1	15	0.885
sp|P37840|SYUA_HUMAN	SNCA	Alpha-synuclein	6.69	6.95	65.7	5	0.885
sp|Q9NRN7|ADPPT_HUMAN	AASDHPPT	L-aminoadipate-semialdehyde dehydrogenase-phosphopantetheinyl transferase	21.79	21.83	49.8	14	0.885
sp|Q12959|DLG1_HUMAN	DLG1	Disks large homolog 1	31.36	31.58	36.7	19	0.886
sp|O00203|AP3B1_HUMAN	AP3B1	AP-3 complex subunit beta-1	52.62	56.15	48.8	33	0.886
sp|O00411|RPOM_HUMAN	POLRMT	DNA-directed RNA polymerase, mitochondrial	56.26	56.69	45.2	38	0.886
sp|Q9Y3B6|EMC9_HUMAN	EMC9	ER membrane protein complex subunit 9	1.31	1.41	26.9	2	0.886
sp|O60502|OGA_HUMAN	MGEA5	Protein O-GlcNAcase	32.62	32.81	33.1	18	0.886
sp|Q9Y4K1|AIM1_HUMAN	AIM1	Absent in melanoma 1 protein	11.37	12.37	16.7	8	0.886
sp|P23919|KTHY_HUMAN	DTYMK	Thymidylate kinase	25.8	25.99	82.6	14	0.886
sp|P36954|RPB9_HUMAN	POLR2I	DNA-directed RNA polymerase II subunit RPB9	7.88	8	59.2	6	0.886
sp|Q99735|MGST2_HUMAN	MGST2	Microsomal glutathione S-transferase 2	4	4	19.1	2	0.886
sp|P61106|RAB14_HUMAN	RAB14	Ras-related protein Rab-14	25.05	28.4	77.2	24	0.886
sp|P24666|PPAC_HUMAN	ACP1	Low molecular weight phosphotyrosine protein phosphatase	16.83	16.89	73.4	20	0.887
sp|P57105|SYJ2B_HUMAN	SYNJ2BP	Synaptojanin-2-binding protein	12.13	12.24	72.4	8	0.887
sp|O60256|KPRB_HUMAN	PRPSAP2	Phosphoribosyl pyrophosphate synthase-associated protein 2	27.84	28	61.5	15	0.887
sp|Q5VST6|AB17B_HUMAN	ABHD17B	Alpha/beta hydrolase domain-containing protein 17B	4.66	4.72	36.1	3	0.887

sp|Q9Y266|NUDC_HUMAN	NUDC	Nuclear migration protein nudC	39.42	42.54	73.4	31	0.887
sp|P49841|GSK3B_HUMAN	GSK3B	Glycogen synthase kinase-3 beta	18.25	18.58	52.9	11	0.887
sp|Q5VSL9|STRP1_HUMAN	STRIP1	Striatin-interacting protein 1	23.51	24.32	38.1	15	0.887
sp|Q9BQP7|MGME1_HUMAN	MGME1	Mitochondrial genome maintenance exonuclease 1	28.73	28.94	56.7	18	0.887
sp|P15924|DESP_HUMAN	DSP	Desmoplakin	208.2	211.36	61.7	124	0.887
sp|Q9BY89|K1671_HUMAN	KIAA1671	Uncharacterized protein KIAA1671	12.38	13.19	20.2	10	0.887
sp|Q8IXM3|RM41_HUMAN	MRPL41	39S ribosomal protein L41, mitochondrial	11.41	11.64	51.1	7	0.887
sp|Q93052|LPP_HUMAN	LPP	Lipoma-preferred partner	14.5	14.76	33.5	8	0.888
sp|Q00341|VIGLN_HUMAN	HDLBP	Vigilin	143	145.85	73.8	107	0.888
sp|Q149N8|SHPRH_HUMAN	SHPRH	E3 ubiquitin-protein ligase SHPRH	6.8	6.97	22.9	5	0.888
sp|Q9Y5J9|TIM8B_HUMAN	TIMM8B	Mitochondrial import inner membrane translocase subunit Tim8 B	6	6	34.9	4	0.888
sp|Q9NRZ7|PLCC_HUMAN	AGPAT3	1-acyl-sn-glycerol-3-phosphate acyltransferase gamma	5.7	5.98	26.9	3	0.888
sp|Q9Y237|PIN4_HUMAN	PIN4	Peptidyl-prolyl cis-trans isomerase NIMA-interacting 4	12.07	12.09	70.2	11	0.888
sp|P06733|ENOA_HUMAN	ENO1	Alpha-enolase	93.51	93.9	92.2	267	0.888
sp|Q8TDP1|RNH2C_HUMAN	RNASEH2C	Ribonuclease H2 subunit C	6.68	6.73	53.1	4	0.888
sp|Q9BT17|MTG1_HUMAN	MTG1	Mitochondrial ribosome-associated GTPase 1	13.73	13.83	54.8	9	0.888
sp|O14745|NHRF1_HUMAN	SLC9A3R1	Na(+)/H(+) exchange regulatory cofactor NHE-RF1	36.65	37.16	69.3	31	0.888
sp|P42025|ACTY_HUMAN	ACTR1B	Beta-centractin	8.57	26.18	64.4	21	0.888
sp|Q9NPH0|PPA6_HUMAN	ACP6	Lysophosphatidic acid phosphatase type 6	4.49	4.55	26.2	3	0.888
sp|Q9Y4P8|WIPI2_HUMAN	WIPI2	WD repeat domain phosphoinositide-interacting protein 2	4.69	4.79	19.6	3	0.888
sp|Q9H9P8|L2HDH_HUMAN	L2HGDH	L-2-hydroxyglutarate dehydrogenase, mitochondrial	22.07	22.15	43.8	12	0.888
sp|Q58EX2|SDK2_HUMAN	SDK2	Protein sidekick-2	3.21	3.41	8.7	4	0.888
sp|Q9H0N0|RAB6C_HUMAN	RAB6C	Ras-related protein Rab-6C	2	2.36	27.2	6	0.889
sp|P53667|LIMK1_HUMAN	LIMK1	LIM domain kinase 1	2	2.27	13.3	2	0.889
sp|Q8N2F6|ARM10_HUMAN	ARMC10	Armadillo repeat-containing protein 10	4.47	4.53	25.1	4	0.889
sp|Q9Y388|RBMX2_HUMAN	RBMX2	RNA-binding motif protein, X-linked 2	8.38	8.41	25.8	5	0.889
sp|P43121|MUC18_HUMAN	MCAM	Cell surface glycoprotein MUC18	7.74	7.93	21.7	6	0.889
sp|P07437|TBB5_HUMAN	TUBB	Tubulin beta chain	97.11	97.21	85.4	393	0.889
sp|Q9H6R4|NOL6_HUMAN	NOL6	Nucleolar protein 6	46.05	46.93	41.1	30	0.889
sp|P46013|KI67_HUMAN	MKI67	Antigen KI-67	193	193.22	56.8	108	0.889
sp|Q9UBF8|PI4KB_HUMAN	PI4KB	Phosphatidylinositol 4-kinase beta	4.55	4.71	13.2	5	0.889
sp|P39656|OST48_HUMAN	DDOST	Dolichyl-diphosphooligosaccharide--protein glycosyltransferase 48 kDa subunit	35.98	37.24	58.3	43	0.890
sp|P51452|DUS3_HUMAN	DUSP3	Dual specificity protein phosphatase 3	12.01	12.01	53	11	0.890
sp|Q9Y4E1|FA21C_HUMAN	FAM21C	WASH complex subunit FAM21C	2.03	32.52	34.2	17	0.890
sp|O14939|PLD2_HUMAN	PLD2	Phospholipase D2	2.37	2.73	12.1	3	0.890
sp|Q96IZ0|PAWR_HUMAN	PAWR	PRKC apoptosis WT1 regulator protein	12.65	12.82	57.1	7	0.890
sp|Q8IY95|TM192_HUMAN	TMEM192	Transmembrane protein 192	6.67	6.84	25.1	5	0.890
sp|O95721|SNP29_HUMAN	SNAP29	Synaptosomal-associated protein 29	13.86	13.95	49.6	7	0.890
sp|Q00535|CDK5_HUMAN	CDK5	Cyclin-dependent-like kinase 5	12.16	14.96	53.4	10	0.890
sp|Q969T9|WBP2_HUMAN	WBP2	WW domain-binding protein 2	4.91	5.12	21.5	5	0.890
sp|Q9P289|STK26_HUMAN	STK26	Serine/threonine-protein kinase 26	30.97	35.78	65.1	30	0.891
sp|Q8N2M8|CLASR_HUMAN	CLASRP	CLK4-associating serine/arginine rich protein	4.1	4.16	23.9	3	0.891
sp|Q9NSE4|SYIM_HUMAN	IARS2	Isoleucine--tRNA ligase, mitochondrial	80.27	82.98	64.6	63	0.891
sp|Q9BTT0|AN32E_HUMAN	ANP32E	Acidic leucine-rich nuclear phosphoprotein 32 family member E	14.66	15.01	41.8	11	0.891
sp|Q16774|KGUA_HUMAN	GUK1	Guanylate kinase	10.08	10.32	75.1	7	0.891
sp|O94979|SC31A_HUMAN	SEC31A	Protein transport protein Sec31A	71.16	72.4	46.8	56	0.891
sp|Q9NVV4|PAPD1_HUMAN	MTPAP	Poly(A) RNA polymerase, mitochondrial	26.75	27.77	52.4	20	0.891
sp|Q96IJ6|GMPPA_HUMAN	GMPPA	Mannose-1-phosphate guanyltransferase alpha	14.77	14.81	35.5	10	0.891
sp|P49770|EI2BB_HUMAN	EIF2B2	Translation initiation factor eIF-2B subunit beta	9.45	10.63	30.8	7	0.891

sp|P49903|SPS1_HUMAN	SEPHS1	Selenide, water dikinase 1	23.34	24.09	52.8	21	0.892
sp|P56945|BCAR1_HUMAN	BCAR1	Breast cancer anti-estrogen resistance protein 1	18.07	18.59	30.7	9	0.892
sp|Q6UVY6|MOXD1_HUMAN	MOXD1	DBH-like monooxygenase protein 1	2.28	3.01	13.7	3	0.892
sp|P52907|CAZA1_HUMAN	CAPZA1	F-actin-capping protein subunit alpha-1	16.14	23.27	77.6	23	0.892
sp|Q14739|LBR_HUMAN	LBR	Lamin-B receptor	29.22	30.53	34.6	28	0.892
sp|P78357|CNTP1_HUMAN	CNTNAP1	Contactin-associated protein 1	3.07	3.18	11.1	3	0.892
sp|Q9HBH1|DEFM_HUMAN	PDF	Peptide deformylase, mitochondrial	16.68	16.79	55.1	13	0.893
sp|O00410|IPO5_HUMAN	IPO5	Importin-5	86	86.89	71.6	86	0.893
sp|P07954|FUMH_HUMAN	FH	Fumarate hydratase, mitochondrial	54.96	55.49	74.9	53	0.893
sp|Q13889|TF2H3_HUMAN	GTF2H3	General transcription factor IIH subunit 3	6.17	7.45	33.8	4	0.893
sp|Q9NRL2|BAZ1A_HUMAN	BAZ1A	Bromodomain adjacent to zinc finger domain protein 1A	53.12	57.76	40.2	31	0.893
sp|Q14498|RBM39_HUMAN	RBM39	RNA-binding protein 39	44.25	44.41	54.2	49	0.893
sp|Q9NTJ5|SAC1_HUMAN	SACM1L	Phosphatidylinositide phosphatase SAC1	46.05	47.8	53	31	0.893
sp|P42226|STAT6_HUMAN	STAT6	Signal transducer and activator of transcription 6	4.02	4.19	18.7	2	0.893
sp|Q86XP3|DDX42_HUMAN	DDX42	ATP-dependent RNA helicase DDX42	61.9	62.15	55.7	34	0.894
sp|Q6NUQ1|RINT1_HUMAN	RINT1	RAD50-interacting protein 1	10.45	11.71	28.8	7	0.894
sp|P82650|RT22_HUMAN	MRPS22	28S ribosomal protein S22, mitochondrial	33.73	35.32	65.6	19	0.894
sp|P31937|3HIDH_HUMAN	HIBADH	3-hydroxyisobutyrate dehydrogenase, mitochondrial	32.77	32.84	75.9	29	0.894
sp|P0CW22|RS17L_HUMAN	RPS17L	40S ribosomal protein S17-like	22.14	22.89	69.6	44	0.894
sp|Q9P0U3|SENP1_HUMAN	SENP1	Sentrin-specific protease 1	6.2	6.36	19.7	5	0.895
sp|O15145|ARPC3_HUMAN	ARPC3	Actin-related protein 2/3 complex subunit 3	13.78	13.97	67.4	10	0.895
sp|O95159|ZFPL1_HUMAN	ZFPL1	Zinc finger protein-like 1	11.68	11.72	30	6	0.895
sp|Q9BWD1|THIC_HUMAN	ACAT2	Acetyl-CoA acetyltransferase, cytosolic	46.31	46.39	81.9	60	0.895
sp|Q8IZP0|ABI1_HUMAN	ABI1	Abl interactor 1	6.68	11.75	27.2	8	0.895
sp|Q9Y3B2|EXOS1_HUMAN	EXOSC1	Exosome complex component CSL4	11.51	11.68	76.4	6	0.895
sp|P09417|DHPR_HUMAN	QDPR	Dihydropteridine reductase	18.79	19.72	74.2	16	0.895
sp|P53350|PLK1_HUMAN	PLK1	Serine/threonine-protein kinase PLK1	26.89	27.94	49.4	19	0.895
sp|P40306|PSB10_HUMAN	PSMB10	Proteasome subunit beta type-10	4.03	4.05	26	2	0.895
sp|Q9Y5A9|YTHD2_HUMAN	YTHDF2	YTH domain-containing family protein 2	22.32	23.52	34.4	15	0.895
sp|P37198|NUP62_HUMAN	NUP62	Nuclear pore glycoprotein p62	27.78	28.1	35.1	22	0.895
sp|Q96KR1|ZFR_HUMAN	ZFR	Zinc finger RNA-binding protein	53.31	54.91	52.9	38	0.895
sp|P22033|MUTA_HUMAN	MUT	Methylmalonyl-CoA mutase, mitochondrial	38.88	39.22	49.9	25	0.895
sp|P50990|TCPQ_HUMAN	CCT8	T-complex protein 1 subunit theta	85.18	87.07	88.3	89	0.895
sp|O95433|AHSA1_HUMAN	AHSA1	Activator of 90 kDa heat shock protein ATPase homolog 1	36.94	37.42	78.7	31	0.896
sp|Q8N9F7|GDPD1_HUMAN	GDPD1	Glycerophosphodiester phosphodiesterase domain-containing protein 1	11.49	11.62	45.9	8	0.896
sp|Q15435|PP1R7_HUMAN	PPP1R7	Protein phosphatase 1 regulatory subunit 7	29.84	30.5	60.3	21	0.896
sp|Q9Y6V7|DDX49_HUMAN	DDX49	Probable ATP-dependent RNA helicase DDX49	23.86	26.57	61.7	14	0.896
sp|Q7Z7A4|PXK_HUMAN	PXK	PX domain-containing protein kinase-like protein	2	4.09	17.1	2	0.896
sp|P28288|ABCD3_HUMAN	ABCD3	ATP-binding cassette sub-family D member 3	46.79	47.97	55.8	34	0.896
sp|Q6UX53|MET7B_HUMAN	METTL7B	Methyltransferase-like protein 7B	13	15.22	44.3	8	0.896
sp|Q96T17|MA7D2_HUMAN	MAP7D2	MAP7 domain-containing protein 2	3.38	3.69	27.1	4	0.896
sp|Q96EM0|T3HPD_HUMAN	L3HYPDH	Trans-3-hydroxy-L-proline dehydratase	11.12	13.39	40.7	9	0.896
sp|Q6NVY1|HIBCH_HUMAN	HIBCH	3-hydroxyisobutyryl-CoA hydrolase, mitochondrial	30.74	32.68	64.8	19	0.896
sp|Q96DB5|RMD1_HUMAN	RMDN1	Regulator of microtubule dynamics protein 1	21.91	22.05	55.7	16	0.896
sp|P00403|COX2_HUMAN	MT-CO2	Cytochrome c oxidase subunit 2	9.71	9.83	44.9	19	0.896
sp|O75146|HIP1R_HUMAN	HIP1R	Huntingtin-interacting protein 1-related protein	29.78	30.81	43.8	16	0.896
sp|P11234|RALB_HUMAN	RALB	Ras-related protein Ral-B	4.64	17.1	51.5	10	0.896
sp|P46459|NSF_HUMAN	NSF	Vesicle-fusing ATPase	62.83	63.12	63	42	0.896
sp|O43776|SYNC_HUMAN	NARS	Asparagine--tRNA ligase, cytoplasmic	42.23	45.47	45.1	34	0.896

sp|Q9Y2I1|NISCH_HUMAN	NISCH	Nischarin	11.71	14.69	15.2	10	0.896
sp|O43795|MYO1B_HUMAN	MYO1B	Unconventional myosin-Ib	84.3	84.7	60	58	0.896
sp|O15347|HMGB3_HUMAN	HMGB3	High mobility group protein B3	20.14	24.2	53	17	0.897
sp|Q8NBM8|PCYXL_HUMAN	PCYOX1L	Prenylcysteine oxidase-like	4.02	4.5	15.2	4	0.897
sp|Q96JN8|NEUL4_HUMAN	NEURL4	Neuralized-like protein 4	6.01	6.08	11.8	3	0.897
sp|P32121|ARRB2_HUMAN	ARRB2	Beta-arrestin-2	14.05	16.22	38.4	8	0.897
sp|Q8WUA8|TSK_HUMAN	TSKU	Tsukushin	2	2	4.8	2	0.897
sp|O75955|FLOT1_HUMAN	FLOT1	Flotillin-1	33.82	34.81	68.2	20	0.897
sp|Q5T4S7|UBR4_HUMAN	UBR4	E3 ubiquitin-protein ligase UBR4	155	158.2	35.9	90	0.897
sp|Q09472|EP300_HUMAN	EP300	Histone acetyltransferase p300	14.3	14.86	16	10	0.897
sp|Q9Y375|CIA30_HUMAN	NDUFAF1	Complex I intermediate-associated protein 30, mitochondrial	12.38	12.75	43.4	9	0.897
sp|O60762|DPM1_HUMAN	DPM1	Dolichol-phosphate mannosyltransferase subunit 1	23.73	24.69	70.4	18	0.897
sp|Q15907|RB11B_HUMAN	RAB11B	Ras-related protein Rab-11B	19.27	20.06	58.7	14	0.897
sp|P60866|RS20_HUMAN	RPS20	40S ribosomal protein S20	6.66	7.06	42	6	0.897
sp|Q8N3Z6|ZCHC7_HUMAN	ZCCHC7	Zinc finger CCHC domain-containing protein 7	2.23	2.29	18.8	2	0.897
sp|P30260|CDC27_HUMAN	CDC27	Cell division cycle protein 27 homolog	32.94	35.2	44.2	22	0.898
sp|Q99497|PARK7_HUMAN	PARK7	Protein deglycase DJ-1	34.33	34.39	95.8	37	0.898
sp|P20339|RAB5A_HUMAN	RAB5A	Ras-related protein Rab-5A	10.09	18.3	72.6	24	0.898
sp|P45880|VDAC2_HUMAN	VDAC2	Voltage-dependent anion-selective channel protein 2	39.4	45.11	79.6	68	0.898
sp|Q6P158|DHX57_HUMAN	DHX57	Putative ATP-dependent RNA helicase DHX57	33.39	34.08	35.2	22	0.898
sp|O60563|CCNT1_HUMAN	CCNT1	Cyclin-T1	3.46	3.61	15.2	3	0.898
sp|B7ZAP0|RBG10_HUMAN	RABGAP1L	Rab GTPase-activating protein 1-like, isoform 10	1.52	6	41.9	4	0.898
sp|P52294|IMA5_HUMAN	KPNA1	Importin subunit alpha-5	6.18	19.32	33.5	12	0.898
sp|Q9NV70|EXOC1_HUMAN	EXOC1	Exocyst complex component 1	32.74	33.16	44	19	0.898
sp|Q8NI35|INADL_HUMAN	INADL	InaD-like protein	2.04	3.77	14.1	5	0.898
sp|Q96RQ3|MCCA_HUMAN	MCCC1	Methylcrotonoyl-CoA carboxylase subunit alpha, mitochondrial	30.21	30.84	46.5	16	0.899
sp|P06213|INSR_HUMAN	INSR	Insulin receptor	11.25	17.21	18	12	0.899
sp|Q32NB8|PGPS1_HUMAN	PGS1	CDP-diacylglycerol--glycerol-3-phosphate 3-phosphatidyltransferase, mitochondrial	2.26	2.36	20	2	0.899
sp|Q6FI81|CPIN1_HUMAN	CIAPIN1	Anamorsin	22.62	24.11	67.6	14	0.899
sp|Q16881|TRXR1_HUMAN	TXNRD1	Thioredoxin reductase 1, cytoplasmic	56.09	56.25	63.6	38	0.899
sp|O75976|CBPD_HUMAN	CPD	Carboxypeptidase D	43.48	44.02	33.4	25	0.899
sp|Q96Q15|SMG1_HUMAN	SMG1	Serine/threonine-protein kinase SMG1	36.56	43.88	22.2	23	0.899
sp|Q9BSB4|ATGA1_HUMAN	ATG101	Autophagy-related protein 101	6	6	29.8	5	0.899
sp|Q2NL82|TSR1_HUMAN	TSR1	Pre-rRNA-processing protein TSR1 homolog	47.88	48.35	56.5	26	0.900
sp|Q9Y2V2|CHSP1_HUMAN	CARHSP1	Calcium-regulated heat stable protein 1	9.07	9.24	64	13	0.900
sp|Q99661|KIF2C_HUMAN	KIF2C	Kinesin-like protein KIF2C	27.27	35.34	53.5	22	0.900
sp|Q9HDC9|APMAP_HUMAN	APMAP	Adipocyte plasma membrane-associated protein	36.89	39.1	61.5	40	0.900
sp|Q9NXH8|TOR4A_HUMAN	TOR4A	Torsin-4A	2.35	2.48	20.3	3	0.900
sp|P40424|PBX1_HUMAN	PBX1	Pre-B-cell leukemia transcription factor 1	3.05	8.42	30	6	0.900
sp|Q8N684|CPSF7_HUMAN	CPSF7	Cleavage and polyadenylation specificity factor subunit 7	22.92	23.1	39.7	16	0.900
sp|Q9NW64|RBM22_HUMAN	RBM22	Pre-mRNA-splicing factor RBM22	25.01	25.18	48.1	19	0.900
sp|Q86W42|THOC6_HUMAN	THOC6	THO complex subunit 6 homolog	17.14	17.48	62.2	14	0.901
sp|Q13642|FHL1_HUMAN	FHL1	Four and a half LIM domains protein 1	15.49	16.2	50.5	10	0.901
sp|P04049|RAF1_HUMAN	RAF1	RAF proto-oncogene serine/threonine-protein kinase	8.79	15.63	34.6	10	0.901
sp|Q8IVT5|KSR1_HUMAN	KSR1	Kinase suppressor of Ras 1	2.68	2.77	14.1	3	0.901
sp|Q9H2K0|IF3M_HUMAN	MTIF3	Translation initiation factor IF-3, mitochondrial	6.04	6.15	24.8	4	0.901
sp|Q86TX2|ACOT1_HUMAN	ACOT1	Acyl-coenzyme A thioesterase 1	48.33	48.42	86.7	42	0.901
sp|Q9Y6Y0|NS1BP_HUMAN	IVNS1ABP	Influenza virus NS1A-binding protein	4.84	5.09	15.7	5	0.901
sp|Q86U86|PB1_HUMAN	PBRM1	Protein polybromo-1	66.62	68.21	42	32	0.902

sp|Q5JPI3|CC038_HUMAN	C3orf38	Uncharacterized protein C3orf38	6.19	6.26	29.8	3	0.902
sp|Q7L8L6|FAKD5_HUMAN	FASTKD5	FAST kinase domain-containing protein 5	42.51	44.31	51.3	29	0.902
sp|Q9HAS0|NJMU_HUMAN	C17orf75	Protein Njmu-R1	10.48	10.73	38.4	7	0.902
sp|Q9H9C1|SPE39_HUMAN	VIPAS39	Spermatogenesis-defective protein 39 homolog	8.72	8.93	33.5	7	0.902
sp|Q9Y5X3|SNX5_HUMAN	SNX5	Sorting nexin-5	32.74	35.85	60.9	24	0.902
sp|Q9H7L9|SDS3_HUMAN	SUDS3	Sin3 histone deacetylase corepressor complex component SDS3	14.06	14.48	39.3	7	0.902
sp|P10606|COX5B_HUMAN	COX5B	Cytochrome c oxidase subunit 5B, mitochondrial	17.94	18.12	66.7	11	0.902
sp|P49366|DHYS_HUMAN	DHPS	Deoxyhypusine synthase	18.69	18.79	46.6	9	0.902
sp|Q9BWH2|FUND2_HUMAN	FUNDC2	FUN14 domain-containing protein 2	4.06	4.07	36.5	3	0.902
sp|P38919|IF4A3_HUMAN	EIF4A3	Eukaryotic initiation factor 4A-III	52.29	61.74	79.1	63	0.902
sp|Q9P258|RCC2_HUMAN	RCC2	Protein RCC2	42.88	43.12	67.6	38	0.902
sp|Q00839|HNRPU_HUMAN	HNRNPU	Heterogeneous nuclear ribonucleoprotein U	126.13	126.22	69.5	215	0.902
sp|P55209|NP1L1_HUMAN	NAP1L1	Nucleosome assembly protein 1-like 1	34.61	35.63	56.5	39	0.903
sp|Q9NP61|ARFG3_HUMAN	ARFGAP3	ADP-ribosylation factor GTPase-activating protein 3	20.37	20.52	35.9	13	0.903
sp|P28838|AMPL_HUMAN	LAP3	Cytosol aminopeptidase	60.69	61.82	78.4	60	0.903
sp|Q13501|SQSTM_HUMAN	SQSTM1	Sequestosome-1	13.58	13.85	44.8	11	0.903
sp|Q13057|COASY_HUMAN	COASY	Bifunctional coenzyme A synthase	21.29	21.48	44.5	15	0.903
sp|Q00059|TFAM_HUMAN	TFAM	Transcription factor A, mitochondrial	24.71	25.44	58.9	14	0.903
sp|Q8N2G8|GHDC_HUMAN	GHDC	GH3 domain-containing protein	9.2	9.34	18.7	6	0.903
sp|Q96BW9|TAM41_HUMAN	TAMM41	Phosphatidate cytidylyltransferase, mitochondrial	18.37	18.54	35.2	11	0.903
sp|Q5EBL4|RIPL1_HUMAN	RILPL1	RILP-like protein 1	3.48	3.58	37.2	3	0.903
sp|Q9UEY8|ADDG_HUMAN	ADD3	Gamma-adducin	24.64	25.52	40.7	17	0.903
sp|Q9NUQ7|UFSP2_HUMAN	UFSP2	Ufm1-specific protease 2	20.15	20.23	43.9	10	0.903
sp|Q12894|IFRD2_HUMAN	IFRD2	Interferon-related developmental regulator 2	4.02	4.02	16.8	2	0.903
sp|A3KN83|SBNO1_HUMAN	SBNO1	Protein strawberry notch homolog 1	24.78	25.84	27.6	15	0.903
sp|P22102|PUR2_HUMAN	GART	Trifunctional purine biosynthetic protein adenosine-3	102.28	103.49	76.9	104	0.903
sp|Q5VTL8|PR38B_HUMAN	PRPF38B	Pre-mRNA-splicing factor 38B	7.63	8.92	25.6	6	0.904
sp|Q9BRX5|PSF3_HUMAN	GINS3	DNA replication complex GINS protein PSF3	21.11	21.17	71.8	12	0.904
sp|P16930|FAAA_HUMAN	FAH	Fumarylacetoacetase	34.12	34.16	65.4	22	0.904
sp|Q53F19|CQ085_HUMAN	C17orf85	Uncharacterized protein C17orf85	9.94	10.65	34.4	7	0.904
sp|P40616|ARL1_HUMAN	ARL1	ADP-ribosylation factor-like protein 1	14.02	14.17	68.5	8	0.904
sp|Q8IY26|PPAC2_HUMAN	PPAPDC2	Presqualene diphosphate phosphatase	6.01	6.04	24.1	3	0.904
sp|Q8N5L8|RP25L_HUMAN	RPP25L	Ribonuclease P protein subunit p25-like protein	5.28	5.36	39.9	3	0.904
sp|Q6ZMI0|PPR21_HUMAN	PPP1R21	Protein phosphatase 1 regulatory subunit 21	12.04	12.3	33.2	6	0.904
sp|Q9BTY7|HGH1_HUMAN	HGH1	Protein HGH1 homolog	21.05	21.15	52.1	11	0.904
sp|A4D1S0|KLRG2_HUMAN	KLRG2	Killer cell lectin-like receptor subfamily G member 2	3.19	3.31	14.9	3	0.904
sp|O14949|QCR8_HUMAN	UQCRQ	Cytochrome b-c1 complex subunit 8	8.44	8.73	62.2	8	0.904
sp|Q9BWS9|CHID1_HUMAN	CHID1	Chitinase domain-containing protein 1	24.54	25.51	45.3	13	0.904
sp|Q6GQQ9|OTU7B_HUMAN	OTUD7B	OTU domain-containing protein 7B	11.79	12.1	18.2	6	0.904
sp|Q99470|SDF2_HUMAN	SDF2	Stromal cell-derived factor 2	4.47	4.51	34.1	7	0.904
sp|P78381|S35A2_HUMAN	SLC35A2	UDP-galactose translocator	1.35	1.43	9.1	2	0.905
sp|O60508|PRP17_HUMAN	CDC40	Pre-mRNA-processing factor 17	28.21	28.31	47.7	15	0.905
sp|Q9HB09|B2L12_HUMAN	BCL2L12	Bcl-2-like protein 12	3.35	3.46	20.7	2	0.905
sp|P46734|MP2K3_HUMAN	MAP2K3	Dual specificity mitogen-activated protein kinase kinase 3	24.87	26.17	54.2	20	0.905
sp|Q9NQC7|CYLD_HUMAN	CYLD	Ubiquitin carboxyl-terminal hydrolase CYLD	2.01	2.61	12.2	3	0.905
sp|Q9NQA3|WASH6_HUMAN	WASH6P	WAS protein family homolog 6	2	8.13	28.9	6	0.905
sp|P52306|GDS1_HUMAN	RAP1GDS1	Rap1 GTPase-GDP dissociation stimulator 1	41.19	42.7	54.4	25	0.905
sp|O96013|PAK4_HUMAN	PAK4	Serine/threonine-protein kinase PAK 4	19.9	20.45	40.4	11	0.905
sp|P62070|RRAS2_HUMAN	RRAS2	Ras-related protein R-Ras2	12.63	12.67	48	9	0.905

sp|Q86VP1|TAXB1_HUMAN	TAX1BP1	Tax1-binding protein 1	3.01	3.77	19.1	6	0.905
sp|Q9NWH9|SLTM_HUMAN	SLTM	SAFB-like transcription modulator	24.67	28.22	31.3	15	0.905
sp|Q9UII2|ATIF1_HUMAN	ATPIF1	ATPase inhibitor, mitochondrial	3.89	4.02	32.1	3	0.905
sp|Q9BZE9|ASPC1_HUMAN	ASPSCR1	Tether containing UBX domain for GLUT4	27.23	27.59	66.6	19	0.906
sp|Q9BUE0|MED18_HUMAN	MED18	Mediator of RNA polymerase II transcription subunit 18	6.88	7.04	38	5	0.906
sp|Q93063|EXT2_HUMAN	EXT2	Exostosin-2	8.2	8.22	17.6	5	0.906
sp|Q99424|ACOX2_HUMAN	ACOX2	Peroxisomal acyl-coenzyme A oxidase 2	4.28	6.38	12.8	3	0.906
sp|Q8IXI1|MIRO2_HUMAN	RHOT2	Mitochondrial Rho GTPase 2	23.3	26.3	49.2	18	0.906
sp|Q14108|SCRB2_HUMAN	SCARB2	Lysosome membrane protein 2	13.58	17.72	38.5	10	0.906
sp|O14786|NRP1_HUMAN	NRP1	Neuropilin-1	7.05	7.24	15.6	5	0.906
sp|P32754|HPPD_HUMAN	HPD	4-hydroxyphenylpyruvate dioxygenase	33.42	33.5	77.6	21	0.906
sp|Q13868|EXOS2_HUMAN	EXOSC2	Exosome complex component RRP4	19.01	19.1	53.2	18	0.906
sp|P13196|HEM1_HUMAN	ALAS1	5-aminolevulinate synthase, nonspecific, mitochondrial	6.34	6.58	27.7	5	0.906
sp|Q14232|EI2BA_HUMAN	EIF2B1	Translation initiation factor eIF-2B subunit alpha	22.48	22.71	65.3	15	0.906
sp|Q16891|MIC60_HUMAN	IMMT	MIC complex subunit MIC60	98.24	98.5	75.6	81	0.906
sp|Q6UUV9|CRTC1_HUMAN	CRTC1	CREB-regulated transcription coactivator 1	6.97	7.05	15.8	4	0.906
sp|Q8WWB7|GLMP_HUMAN	GLMP	Glycosylated lysosomal membrane protein	3.26	3.34	14.3	4	0.906
sp|Q07021|C1QBP_HUMAN	C1QBP	Complement component 1 Q subcomponent-binding protein, mitochondrial	21.53	21.77	73.1	66	0.906
sp|Q5MIZ7|P4R3B_HUMAN	SMEK2	Serine/threonine-protein phosphatase 4 regulatory subunit 3B	9.56	19.93	30	12	0.906
sp|Q9BTV4|TMM43_HUMAN	TMEM43	Transmembrane protein 43	27.97	28.96	53	18	0.906
sp|Q6ZT21|TMPPE_HUMAN	TMPPE	Transmembrane protein with metallophosphoesterase domain	4.01	4.02	18.1	2	0.907
sp|Q9BY42|RTF2_HUMAN	RTFDC1	Protein RTF2 homolog	13.41	13.66	56.2	8	0.907
sp|Q9UDY2|ZO2_HUMAN	TJP2	Tight junction protein ZO-2	82.24	83.9	58.8	52	0.907
sp|Q9Y570|PPME1_HUMAN	PPME1	Protein phosphatase methylesterase 1	32.32	33.3	52.1	22	0.907
sp|Q9Y2W2|WBP11_HUMAN	WBP11	WW domain-binding protein 11	21.86	22.5	33.7	12	0.907
sp|P47813|IF1AX_HUMAN	EIF1AX	Eukaryotic translation initiation factor 1A, X-chromosomal	12.03	14.89	51.4	10	0.907
sp|Q92791|SC65_HUMAN	P3H4	Synaptonemal complex protein SC65	12.46	12.56	31.4	8	0.907
sp|Q8WW01|SEN15_HUMAN	TSEN15	tRNA-splicing endonuclease subunit Sen15	6	6	36.8	3	0.907
sp|P13674|P4HA1_HUMAN	P4HA1	Prolyl 4-hydroxylase subunit alpha-1	35.82	38.16	57.5	24	0.907
sp|Q12846|STX4_HUMAN	STX4	Syntaxin-4	11.88	12.19	47.1	10	0.907
sp|Q9NZJ6|COQ3_HUMAN	COQ3	Ubiquinone biosynthesis O-methyltransferase, mitochondrial	3.35	3.45	22.2	2	0.907
sp|O15320|CTGE5_HUMAN	CTAGE5	cTAGE family member 5	34.18	35.09	39.6	21	0.907
sp|Q07889|SOS1_HUMAN	SOS1	Son of sevenless homolog 1	3.59	6.97	19.2	9	0.907
sp|Q96IY1|NSL1_HUMAN	NSL1	Kinetochore-associated protein NSL1 homolog	7.51	7.7	45.2	6	0.907
sp|P52701|MSH6_HUMAN	MSH6	DNA mismatch repair protein Msh6	94.88	95.05	55.5	57	0.907
sp|Q96RN5|MED15_HUMAN	MED15	Mediator of RNA polymerase II transcription subunit 15	13.74	13.85	24.2	8	0.908
sp|Q5U5X0|LYRM7_HUMAN	LYRM7	Complex III assembly factor LYRM7	4.51	6.64	54.8	4	0.908
sp|Q6P087|RUSD3_HUMAN	RPUSD3	RNA pseudouridylate synthase domain-containing protein 3	12.99	13.23	44.4	7	0.908
sp|Q05397|FAK1_HUMAN	PTK2	Focal adhesion kinase 1	22.15	22.83	30.7	14	0.908
sp|Q9H1A3|METL9_HUMAN	METTL9	Methyltransferase-like protein 9	4.24	4.28	25.2	4	0.908
sp|Q9UQR1|ZN148_HUMAN	ZNF148	Zinc finger protein 148	10.4	10.55	24.1	8	0.908
sp|O15144|ARPC2_HUMAN	ARPC2	Actin-related protein 2/3 complex subunit 2	32.32	33.29	77	23	0.909
sp|Q6IBS0|TWF2_HUMAN	TWF2	Twinfilin-2	24.43	26.62	73.9	19	0.909
sp|Q7Z569|BRAP_HUMAN	BRAP	BRCA1-associated protein	14.84	14.93	29.1	9	0.909
sp|Q13451|FKBP5_HUMAN	FKBP5	Peptidyl-prolyl cis-trans isomerase FKBP5	37.54	38.41	61.9	24	0.909
sp|P49750|YLPM1_HUMAN	YLPM1	YLP motif-containing protein 1	64.66	64.85	32.2	34	0.909
sp|Q8IUF8|MINA_HUMAN	MINA	Bifunctional lysine-specific demethylase and histidyl-hydroxylase MINA	14.95	15.5	39.4	10	0.909
sp|Q8WUF8|F172A_HUMAN	FAM172A	Protein FAM172A	2.29	2.32	13.7	2	0.909
sp|Q9H410|DSN1_HUMAN	DSN1	Kinetochore-associated protein DSN1 homolog	8.88	9.13	38.8	6	0.909

sp|P00390|GSHR_HUMAN	GSR	Glutathione reductase, mitochondrial	33.25	35.83	69.5	36	0.909
sp|P63104|1433Z_HUMAN	YWHAZ	14-3-3 protein zeta/delta	39.75	48.66	88.6	59	0.909
sp|Q10469|MGAT2_HUMAN	MGAT2	Alpha-1,6-mannosyl-glycoprotein 2-beta-N-acetylglucosaminyltransferase	13.21	13.42	26	7	0.909
sp|Q9H446|RWDD1_HUMAN	RWDD1	RWD domain-containing protein 1	5.44	5.57	33.3	6	0.909
sp|Q9NV88|INT9_HUMAN	INTS9	Integrator complex subunit 9	11.44	11.75	31	6	0.909
sp|Q7KZF4|SND1_HUMAN	SND1	Staphylococcal nuclease domain-containing protein 1	111.59	111.94	80.7	92	0.909
sp|Q8N567|ZCHC9_HUMAN	ZCCHC9	Zinc finger CCHC domain-containing protein 9	7.39	7.76	45.4	6	0.910
sp|Q14257|RCN2_HUMAN	RCN2	Reticulocalbin-2	26.45	26.53	59.9	20	0.910
sp|Q5VZL5|ZMYM4_HUMAN	ZMYM4	Zinc finger MYM-type protein 4	32.59	39.39	34	23	0.910
sp|Q9UGP8|SEC63_HUMAN	SEC63	Translocation protein SEC63 homolog	48.75	49.29	49.6	35	0.910
sp|Q9Y394|DHRS7_HUMAN	DHRS7	Dehydrogenase/reductase SDR family member 7	20.41	20.91	56.1	14	0.910
sp|Q8IXM2|BAP18_HUMAN	BAP18	Chromatin complexes subunit BAP18	8.23	8.26	69.2	4	0.910
sp|O75911|DHRS3_HUMAN	DHRS3	Short-chain dehydrogenase/reductase 3	2.94	3.02	32.1	2	0.910
sp|Q9NQW6|ANLN_HUMAN	ANLN	Actin-binding protein anillin	20.95	21.61	32.2	12	0.910
sp|P51531|SMCA2_HUMAN	SMARCA2	Probable global transcription activator SNF2L2	9.85	39.02	28.2	28	0.910
sp|Q8TDB4|HUMMR_HUMAN	MGARP	Protein MGARP	2.29	2.31	24.6	2	0.910
sp|Q9UHB7|AFF4_HUMAN	AFF4	AF4/FMR2 family member 4	4.79	4.96	12.9	3	0.910
sp|Q13485|SMAD4_HUMAN	SMAD4	Mothers against decapentaplegic homolog 4	6	6.07	12.7	3	0.910
sp|O75054|IGSF3_HUMAN	IGSF3	Immunoglobulin superfamily member 3	3.64	5.56	14.9	7	0.910
sp|Q15120|PDK3_HUMAN	PDK3	[Pyruvate dehydrogenase (acetyl-transferring)] kinase isozyme 3, mitochondrial	24.74	24.97	55.4	14	0.911
sp|Q01831|XPC_HUMAN	XPC	DNA repair protein complementing XP-C cells	14.45	15.43	27.5	11	0.911
sp|Q9Y3Y2|CHTOP_HUMAN	CHTOP	Chromatin target of PRMT1 protein	10.94	11.22	66.5	11	0.911
sp|Q4G0F5|VP26B_HUMAN	VPS26B	Vacuolar protein sorting-associated protein 26B	9.86	10.05	37.8	6	0.911
sp|Q9UBV8|PEF1_HUMAN	PEF1	Peflin	9.72	9.79	29.9	5	0.911
sp|Q13573|SNW1_HUMAN	SNW1	SNW domain-containing protein 1	39.31	39.51	63.3	22	0.912
sp|O43768|ENSA_HUMAN	ENSA	Alpha-endosulfine	13.29	13.57	63.6	9	0.912
sp|O75446|SAP30_HUMAN	SAP30	Histone deacetylase complex subunit SAP30	4.96	5.18	50	4	0.912
sp|P82914|RT15_HUMAN	MRPS15	28S ribosomal protein S15, mitochondrial	12.17	13.23	43.2	7	0.912
sp|P17980|PRS6A_HUMAN	PSMC3	26S protease regulatory subunit 6A	57.11	57.79	82.7	44	0.912
sp|Q7L9L4|MOB1B_HUMAN	MOB1B	MOB kinase activator 1B	4.98	5.08	31	5	0.912
sp|P82663|RT25_HUMAN	MRPS25	28S ribosomal protein S25, mitochondrial	17.63	17.71	78	11	0.912
sp|Q7LBC6|KDM3B_HUMAN	KDM3B	Lysine-specific demethylase 3B	45.95	46.49	35.8	25	0.912
sp|Q49B96|COX19_HUMAN	COX19	Cytochrome c oxidase assembly protein COX19	5.55	6.39	45.6	5	0.912
sp|Q9H5V9|CX056_HUMAN	CXorf56	UPF0428 protein CXorf56	10.22	10.35	41.4	6	0.912
sp|Q2M3G4|SHRM1_HUMAN	SHROOM1	Protein Shroom1	4.88	5.31	19.7	5	0.913
sp|Q7Z7H5|TMED4_HUMAN	TMED4	Transmembrane emp24 domain-containing protein 4	13.58	17.46	56.4	15	0.913
sp|P84085|ARF5_HUMAN	ARF5	ADP-ribosylation factor 5	10.01	23.92	84.4	27	0.913
sp|Q9HB40|RISC_HUMAN	SCPEP1	Retinoid-inducible serine carboxypeptidase	11.5	11.56	23.5	7	0.913
sp|O60313|OPA1_HUMAN	OPA1	Dynamin-like 120 kDa protein, mitochondrial	53	53.78	54.9	29	0.913
sp|Q96S66|CLCC1_HUMAN	CLCC1	Chloride channel CLIC-like protein 1	18.69	18.94	39.8	12	0.913
sp|Q15075|EEA1_HUMAN	EEA1	Early endosome antigen 1	107.29	113.97	65.6	62	0.913
sp|O75477|ERLN1_HUMAN	ERLIN1	Erlin-1	15.56	23.75	51.7	19	0.913
sp|P51858|HDGF_HUMAN	HDGF	Hepatoma-derived growth factor	27.37	29.56	67.9	20	0.913
sp|Q16851|UGPA_HUMAN	UGP2	UTP--glucose-1-phosphate uridylyltransferase	44.8	48.17	68.5	35	0.913
sp|P13693|TCTP_HUMAN	TPT1	Translationally-controlled tumor protein	15.76	16.78	74.4	13	0.914
sp|Q9GZR2|REXO4_HUMAN	REXO4	RNA exonuclease 4	21.3	21.57	56.4	13	0.914
sp|Q9NZZ3|CHMP5_HUMAN	CHMP5	Charged multivesicular body protein 5	8.69	8.92	41.6	6	0.914
sp|Q9NVU7|SDA1_HUMAN	SDAD1	Protein SDA1 homolog	15.53	18.22	29.6	13	0.914
sp|Q86V48|LUZP1_HUMAN	LUZP1	Leucine zipper protein 1	15.41	16.01	30.6	11	0.914

sp|O14975|S27A2_HUMAN	SLC27A2	Very long-chain acyl-CoA synthetase	21.38	21.68	41	12	0.914
sp|Q8NCW5|NNRE_HUMAN	APOA1BP	NAD(P)H-hydrate epimerase	17.15	17.21	45.1	13	0.914
sp|Q9Y5S1|TRPV2_HUMAN	TRPV2	Transient receptor potential cation channel subfamily V member 2	4.95	5.05	14.8	4	0.914
sp|Q96DP5|FMT_HUMAN	MTFMT	Methionyl-tRNA formyltransferase, mitochondrial	5.68	5.79	23.4	3	0.914
sp|O43299|AP5Z1_HUMAN	AP5Z1	AP-5 complex subunit zeta-1	3.04	3.17	15.1	3	0.914
sp|P07099|HYEP_HUMAN	EPHX1	Epoxide hydrolase 1	33.83	34.05	69.9	25	0.914
sp|Q99536|VAT1_HUMAN	VAT1	Synaptic vesicle membrane protein VAT-1 homolog	32.66	33.51	52.7	48	0.914
sp|O60499|STX10_HUMAN	STX10	Syntaxin-10	10.57	10.72	61.5	7	0.914
sp|Q6PCT2|FXL19_HUMAN	FBXL19	F-box/LRR-repeat protein 19	2.04	2.55	10.2	2	0.915
sp|Q5T5U3|RHG21_HUMAN	ARHGAP21	Rho GTPase-activating protein 21	3.84	4.59	13.1	3	0.915
sp|Q9BT73|PSMG3_HUMAN	PSMG3	Proteasome assembly chaperone 3	6.42	6.46	58.2	4	0.915
sp|Q8NHQ9|DDX55_HUMAN	DDX55	ATP-dependent RNA helicase DDX55	21.16	22.04	41.5	13	0.915
sp|O94822|LTN1_HUMAN	LTN1	E3 ubiquitin-protein ligase listerin	13.27	16.13	26.2	12	0.915
sp|Q9BRT8|CBWD1_HUMAN	CBWD1	COBW domain-containing protein 1	20.98	21.33	52.9	13	0.915
sp|Q96SI9|STRBP_HUMAN	STRBP	Spermatid perinuclear RNA-binding protein	28.05	38.66	52.5	22	0.916
sp|Q13445|TMED1_HUMAN	TMED1	Transmembrane emp24 domain-containing protein 1	11.46	11.61	42.7	10	0.916
sp|P49756|RBM25_HUMAN	RBM25	RNA-binding protein 25	26.69	29.92	43.2	22	0.916
sp|Q9Y305|ACOT9_HUMAN	ACOT9	Acyl-coenzyme A thioesterase 9, mitochondrial	27.96	28.04	49.7	17	0.916
sp|Q969N2|PIGT_HUMAN	PIGT	GPI transamidase component PIG-T	22.1	24.08	41	13	0.916
sp|Q9BZH6|WDR11_HUMAN	WDR11	WD repeat-containing protein 11	38.15	38.37	34.4	23	0.916
sp|O75934|SPF27_HUMAN	BCAS2	Pre-mRNA-splicing factor SPF27	22.76	22.9	84.9	18	0.916
sp|Q9UPY5|XCT_HUMAN	SLC7A11	Cystine/glutamate transporter	5.61	5.78	21	4	0.916
sp|P09493|TPM1_HUMAN	TPM1	Tropomyosin alpha-1 chain	7.68	24.96	62.3	17	0.916
sp|O14972|DSCR3_HUMAN	DSCR3	Down syndrome critical region protein 3	3.74	3.93	42.1	3	0.916
sp|O43765|SGTA_HUMAN	SGTA	Small glutamine-rich tetratricopeptide repeat-containing protein alpha	17.35	18.63	41.5	15	0.916
sp|Q05707|COEA1_HUMAN	COL14A1	Collagen alpha-1(XIV) chain	2.81	3.01	11.3	3	0.916
sp|Q6IA69|NADE_HUMAN	NADSYN1	Glutamine-dependent NAD(+) synthetase	2.82	5.38	15.7	7	0.917
sp|Q8IWJ2|GCC2_HUMAN	GCC2	GRIP and coiled-coil domain-containing protein 2	44.57	49.94	41.7	28	0.917
sp|P39687|AN32A_HUMAN	ANP32A	Acidic leucine-rich nuclear phosphoprotein 32 family member A	10.31	24.38	39	28	0.917
sp|Q15477|SKIV2_HUMAN	SKIV2L	Helicase SKI2W	53.56	55.49	47.3	30	0.917
sp|Q15007|FL2D_HUMAN	WTAP	Pre-mRNA-splicing regulator WTAP	18.58	18.69	50	13	0.917
sp|Q9HCM4|E41L5_HUMAN	EPB41L5	Band 4.1-like protein 5	21.33	21.49	34.1	11	0.917
sp|Q9P0M9|RM27_HUMAN	MRPL27	39S ribosomal protein L27, mitochondrial	10.3	12.46	54.1	6	0.918
sp|Q5JPE7|NOMO2_HUMAN	NOMO2	Nodal modulator 2	91.37	92.8	70.2	66	0.918
sp|Q9NNW7|TRXR2_HUMAN	TXNRD2	Thioredoxin reductase 2, mitochondrial	22.73	25.15	58.8	17	0.918
sp|P35268|RL22_HUMAN	RPL22	60S ribosomal protein L22	8.67	10.7	86.7	26	0.918
sp|Q92581|SL9A6_HUMAN	SLC9A6	Sodium/hydrogen exchanger 6	2.81	2.88	8.8	2	0.918
sp|P37268|FDFT_HUMAN	FDFT1	Squalene synthase	28.87	29.04	54	24	0.918
sp|P61158|ARP3_HUMAN	ACTR3	Actin-related protein 3	39.87	39.97	75.4	32	0.918
sp|Q9BZ23|PANK2_HUMAN	PANK2	Pantothenate kinase 2, mitochondrial	11.54	15.48	36.3	11	0.918
sp|O43164|PJA2_HUMAN	PJA2	E3 ubiquitin-protein ligase Praja-2	4.32	4.38	14.4	4	0.918
sp|P26583|HMGB2_HUMAN	HMGB2	High mobility group protein B2	24.94	37.8	64.1	32	0.918
sp|Q5BJF2|TMM97_HUMAN	TMEM97	Transmembrane protein 97	4.01	4.01	26.7	2	0.918
sp|Q7L273|KCTD9_HUMAN	KCTD9	BTB/POZ domain-containing protein KCTD9	5.23	5.35	28	3	0.919
sp|Q9NZW5|MPP6_HUMAN	MPP6	MAGUK p55 subfamily member 6	36.72	43.85	67.4	26	0.919
sp|Q8WWM7|ATX2L_HUMAN	ATXN2L	Ataxin-2-like protein	51.52	51.67	45.5	36	0.919
sp|O00148|DX39A_HUMAN	DDX39A	ATP-dependent RNA helicase DDX39A	18.04	49.36	72.6	54	0.919
sp|Q6UB35|C1TM_HUMAN	MTHFD1L	Monofunctional C1-tetrahydrofolate synthase, mitochondrial	57.83	63.08	58.8	47	0.919
sp|Q9Y6M4|KC1G3_HUMAN	CSNK1G3	Casein kinase I isoform gamma-3	12.59	15.32	35.4	9	0.919

sp|P53007|TXTP_HUMAN	SLC25A1	Tricarboxylate transport protein, mitochondrial	17.67	17.93	45.3	16	0.919
sp|P17844|DDX5_HUMAN	DDX5	Probable ATP-dependent RNA helicase DDX5	53.77	78.2	69.5	79	0.919
sp|P62166|NCS1_HUMAN	NCS1	Neuronal calcium sensor 1	6.02	6.28	46.3	6	0.919
sp|P07477|TRY1_HUMAN	PRSS1	Trypsin-1	9.83	9.9	25.1	11	0.919
sp|Q02790|FKBP4_HUMAN	FKBP4	Peptidyl-prolyl cis-trans isomerase FKBP4	72.59	72.61	91.7	71	0.919
sp|O43639|NCK2_HUMAN	NCK2	Cytoplasmic protein NCK2	1.61	3.79	21.1	4	0.920
sp|Q8TAT6|NPL4_HUMAN	NPLOC4	Nuclear protein localization protein 4 homolog	34.09	34.29	51.6	20	0.920
sp|Q96CS2|HAUS1_HUMAN	HAUS1	HAUS augmin-like complex subunit 1	9.63	9.85	43.5	6	0.920
sp|Q8NEY8|PPHLN_HUMAN	PPHLN1	Periphilin-1	7.11	9.48	24.7	8	0.920
sp|Q92882|OSTF1_HUMAN	OSTF1	Osteoclast-stimulating factor 1	9.9	10.01	50	8	0.920
sp|Q9Y2H5|PKHA6_HUMAN	PLEKHA6	Pleckstrin homology domain-containing family A member 6	1.32	3.65	11	3	0.920
sp|O00193|SMAP_HUMAN	SMAP	Small acidic protein	9.75	9.81	34.4	7	0.920
sp|Q15813|TBCE_HUMAN	TBCE	Tubulin-specific chaperone E	35.66	36.3	75.7	21	0.920
sp|Q96Q45|TM237_HUMAN	TMEM237	Transmembrane protein 237	7.95	8.15	27	6	0.920
sp|Q9Y3U8|RL36_HUMAN	RPL36	60S ribosomal protein L36	8.03	10.05	45.7	11	0.921
sp|Q69YN2|C19L1_HUMAN	CWF19L1	CWF19-like protein 1	18.78	19.52	42	11	0.921
sp|Q9UBF2|COPG2_HUMAN	COPG2	Coatomer subunit gamma-2	43.45	53.78	63.4	34	0.921
sp|Q96LL9|DJC30_HUMAN	DNAJC30	DnaJ homolog subfamily C member 30	3.8	3.89	15	3	0.921
sp|Q13042|CDC16_HUMAN	CDC16	Cell division cycle protein 16 homolog	10.43	10.97	22.3	8	0.921
sp|Q15382|RHEB_HUMAN	RHEB	GTP-binding protein Rheb	8.79	9	52.2	6	0.921
sp|Q8WWY3|PRP31_HUMAN	PRPF31	U4/U6 small nuclear ribonucleoprotein Prp31	30.68	31.86	49.9	21	0.921
sp|Q9NXV2|KCTD5_HUMAN	KCTD5	BTB/POZ domain-containing protein KCTD5	5.42	5.5	30.8	3	0.921
sp|Q9Y316|MEMO1_HUMAN	MEMO1	Protein MEMO1	11.81	13.39	46.5	8	0.921
sp|Q7Z7F7|RM55_HUMAN	MRPL55	39S ribosomal protein L55, mitochondrial	4.01	4.03	47.7	4	0.921
sp|Q92667|AKAP1_HUMAN	AKAP1	A-kinase anchor protein 1, mitochondrial	37.63	38.12	43.7	21	0.921
sp|Q96JG6|SYNDE_HUMAN	CCDC132	Syndetin	12.68	13.08	24.9	8	0.921
sp|Q9BVG9|PTSS2_HUMAN	PTDSS2	Phosphatidylserine synthase 2	4.06	4.07	13.4	4	0.921
sp|Q8NB16|MLKL_HUMAN	MLKL	Mixed lineage kinase domain-like protein	2.93	3.76	23.1	5	0.922
sp|Q53T94|TAF1B_HUMAN	TAF1B	TATA box-binding protein-associated factor RNA polymerase I subunit B	2.56	3.25	20.9	5	0.922
sp|P62249|RS16_HUMAN	RPS16	40S ribosomal protein S16	31.36	31.5	83.6	26	0.922
sp|O75323|NIPS2_HUMAN	GBAS	Protein NipSnap homolog 2	19.95	22.23	63.6	11	0.922
sp|Q9H3K6|BOLA2_HUMAN	BOLA2	BolA-like protein 2	11.57	11.71	82.6	11	0.922
sp|O75925|PIAS1_HUMAN	PIAS1	E3 SUMO-protein ligase PIAS1	10.39	10.74	26.7	6	0.922
sp|P23634|AT2B4_HUMAN	ATP2B4	Plasma membrane calcium-transporting ATPase 4	20.48	42.73	39.9	34	0.922
sp|Q8N8S7|ENAH_HUMAN	ENAH	Protein enabled homolog	37.54	37.7	51.8	30	0.922
sp|P06748|NPM_HUMAN	NPM1	Nucleophosmin	56.08	57.34	80.6	221	0.922
sp|Q9H9Q4|NHEJ1_HUMAN	NHEJ1	Non-homologous end-joining factor 1	6	6.11	39.1	5	0.922
sp|P52789|HXK2_HUMAN	HK2	Hexokinase-2	82.7	83.33	61.2	59	0.923
sp|P10746|HEM4_HUMAN	UROS	Uroporphyrinogen-III synthase	1.72	1.95	20	2	0.923
sp|Q8TAV3|CP2W1_HUMAN	CYP2W1	Cytochrome P450 2W1	2.98	4.77	15.5	5	0.923
sp|Q8NC51|PAIRB_HUMAN	SERBP1	Plasminogen activator inhibitor 1 RNA-binding protein	55.48	55.9	73.8	58	0.923
sp|A0AVI2|FR1L5_HUMAN	FER1L5	Fer-1-like protein 5	2.03	2.27	9.9	3	0.923
sp|O00214|LEG8_HUMAN	LGALS8	Galectin-8	8.62	8.8	25.2	7	0.923
sp|Q6P4E1|CASC4_HUMAN	CASC4	Protein CASC4	5.1	6.4	29.3	5	0.923
sp|Q15742|NAB2_HUMAN	NAB2	NGFI-A-binding protein 2	6.72	6.91	29.5	4	0.923
sp|Q8N8R7|AL14E_HUMAN	ARL14EP	ARL14 effector protein	2.47	2.52	20.4	2	0.923
sp|Q9P1Q0|VPS54_HUMAN	VPS54	Vacuolar protein sorting-associated protein 54	2.02	2.12	18.8	2	0.923
sp|Q6P1S2|CC033_HUMAN	C3orf33	Protein C3orf33	4.01	4.63	32.3	5	0.923
sp|Q9NPJ3|ACO13_HUMAN	ACOT13	Acyl-coenzyme A thioesterase 13	11.93	11.97	75	9	0.923

sp|P11413|G6PD_HUMAN	G6PD	Glucose-6-phosphate 1-dehydrogenase	52	52.06	74.2	36	0.923
sp|Q3ZCQ8|TIM50_HUMAN	TIMM50	Mitochondrial import inner membrane translocase subunit TIM50	26.18	26.46	46.7	17	0.923
sp|P27986|P85A_HUMAN	PIK3R1	Phosphatidylinositol 3-kinase regulatory subunit alpha	2.06	4.13	22.7	3	0.924
sp|P48730|KC1D_HUMAN	CSNK1D	Casein kinase I isoform delta	5.04	17.72	36.1	12	0.924
sp|Q9BVK8|TM147_HUMAN	TMEM147	Transmembrane protein 147	3.83	3.97	16.5	3	0.924
sp|Q9UBQ7|GRHPR_HUMAN	GRHPR	Glyoxylate reductase/hydroxypyruvate reductase	29.41	29.95	69.8	22	0.924
sp|Q9BVC3|DCC1_HUMAN	DSCC1	Sister chromatid cohesion protein DCC1	7.71	7.96	28.5	4	0.924
sp|Q9UIJ7|KAD3_HUMAN	AK3	GTP:AMP phosphotransferase AK3, mitochondrial	21.7	21.75	70	14	0.924
sp|Q15051|IQCB1_HUMAN	IQCB1	IQ calmodulin-binding motif-containing protein 1	3.36	3.98	26.6	6	0.924
sp|P08134|RHOC_HUMAN	RHOC	Rho-related GTP-binding protein RhoC	4	30.54	68.9	34	0.924
sp|Q9C0E8|LNP_HUMAN	LNP	Protein lunapark	16.04	18.46	38.1	15	0.924
sp|Q9UGU5|HMGX4_HUMAN	HMGXB4	HMG domain-containing protein 4	5.97	6.1	23.6	4	0.924
sp|P13798|ACPH_HUMAN	APEH	Acylamino-acid-releasing enzyme	44.74	44.78	58.1	42	0.924
sp|Q92546|RGP1_HUMAN	RGP1	RAB6A-GEF complex partner protein 2	1.86	1.97	15.9	2	0.924
sp|Q9P032|NDUF4_HUMAN	NDUFAF4	NADH dehydrogenase [ubiquinone] 1 alpha subcomplex assembly factor 4	12.69	12.86	57.7	7	0.924
sp|Q8IX04|UEVLD_HUMAN	UEVLD	Ubiquitin-conjugating enzyme E2 variant 3	5.38	5.69	21.4	3	0.924
sp|Q9NXX6|NSE4A_HUMAN	NSMCE4A	Non-structural maintenance of chromosomes element 4 homolog A	6.7	6.78	20.3	5	0.924
sp|Q8NBM4|UBAC2_HUMAN	UBAC2	Ubiquitin-associated domain-containing protein 2	12.94	13.04	38.4	9	0.925
sp|P48426|PI42A_HUMAN	PIP4K2A	Phosphatidylinositol 5-phosphate 4-kinase type-2 alpha	7.36	11.83	37.9	8	0.925
sp|P39019|RS19_HUMAN	RPS19	40S ribosomal protein S19	22.23	22.31	77.2	26	0.925
sp|P48047|ATPO_HUMAN	ATP5O	ATP synthase subunit O, mitochondrial	22.58	23.23	68.1	28	0.925
sp|Q9P016|THYN1_HUMAN	THYN1	Thymocyte nuclear protein 1	14.27	14.48	55.6	10	0.925
sp|Q13686|ALKB1_HUMAN	ALKBH1	Alkylated DNA repair protein alkB homolog 1	6.31	6.36	27.5	3	0.925
sp|Q8IXT5|RB12B_HUMAN	RBM12B	RNA-binding protein 12B	29.82	30.09	28	16	0.925
sp|Q03013|GSTM4_HUMAN	GSTM4	Glutathione S-transferase Mu 4	4	4.92	45.9	4	0.926
sp|P33992|MCM5_HUMAN	MCM5	DNA replication licensing factor MCM5	75.26	75.58	73.6	51	0.926
sp|P41227|NAA10_HUMAN	NAA10	N-alpha-acetyltransferase 10	18.82	18.94	57.5	10	0.926
sp|O95409|ZIC2_HUMAN	ZIC2	Zinc finger protein ZIC 2	5.04	5.19	16	3	0.926
sp|Q96MH6|TMM68_HUMAN	TMEM68	Transmembrane protein 68	2	2.06	28.1	2	0.926
sp|Q8TCX1|DC2L1_HUMAN	DYNC2LI1	Cytoplasmic dynein 2 light intermediate chain 1	4.14	4.34	33.3	2	0.926
sp|P17655|CAN2_HUMAN	CAPN2	Calpain-2 catalytic subunit	33.67	33.75	48.7	18	0.926
sp|P35659|DEK_HUMAN	DEK	Protein DEK	29.46	31.92	51.5	22	0.926
sp|Q96KQ7|EHMT2_HUMAN	EHMT2	Histone-lysine N-methyltransferase EHMT2	38.92	40.28	37	24	0.926
sp|Q96B23|CR025_HUMAN	C18orf25	Uncharacterized protein C18orf25	6	6	16.6	3	0.926
sp|Q9Y6I4|UBP3_HUMAN	USP3	Ubiquitin carboxyl-terminal hydrolase 3	5.21	5.31	13.7	3	0.926
sp|O75348|VATG1_HUMAN	ATP6V1G1	V-type proton ATPase subunit G 1	4.09	4.84	68.6	6	0.926
sp|Q8N4T8|CBR4_HUMAN	CBR4	Carbonyl reductase family member 4	9.8	9.95	54.4	6	0.926
sp|O75354|ENTP6_HUMAN	ENTPD6	Ectonucleoside triphosphate diphosphohydrolase 6	3.97	4.12	20	3	0.927
sp|P13639|EF2_HUMAN	EEF2	Elongation factor 2	138.79	139.19	88.3	254	0.927
sp|Q6PI48|SYDM_HUMAN	DARS2	Aspartate--tRNA ligase, mitochondrial	48.52	51.71	55.4	39	0.927
sp|O95155|UBE4B_HUMAN	UBE4B	Ubiquitin conjugation factor E4 B	21.74	22.11	26.7	13	0.927
sp|P52429|DGKE_HUMAN	DGKE	Diacylglycerol kinase epsilon	9.6	11.77	18.9	6	0.927
sp|Q6PCB5|RSBNL_HUMAN	RSBN1L	Round spermatid basic protein 1-like protein	6.77	6.85	18.9	4	0.927
sp|O15234|CASC3_HUMAN	CASC3	Protein CASC3	2.71	2.83	14.7	3	0.927
sp|Q8WW12|PCNP_HUMAN	PCNP	PEST proteolytic signal-containing nuclear protein	10.25	10.66	47.8	6	0.927
sp|Q96ES6|MFSD3_HUMAN	MFSD3	Major facilitator superfamily domain-containing protein 3	4	5.3	12.1	3	0.927
sp|O95373|IPO7_HUMAN	IPO7	Importin-7	51.84	52.14	43.9	42	0.927
sp|P23246|SFPQ_HUMAN	SFPQ	Splicing factor, proline- and glutamine-rich	66.99	67.14	52.8	76	0.927
sp|Q9NUU7|DD19A_HUMAN	DDX19A	ATP-dependent RNA helicase DDX19A	48.66	49.49	72.6	39	0.928

sp|P25490|TYY1_HUMAN	YY1	Transcriptional repressor protein YY1	7.77	8.33	32.9	7	0.928
sp|Q8WXE9|STON2_HUMAN	STON2	Stonin-2	2.28	2.32	8.3	2	0.928
sp|P31942|HNRH3_HUMAN	HNRNPH3	Heterogeneous nuclear ribonucleoprotein H3	26.85	29.09	68.8	36	0.928
sp|Q99543|DNJC2_HUMAN	DNAJC2	DnaJ homolog subfamily C member 2	42.37	42.6	47.3	28	0.928
sp|A6NDU8|CE051_HUMAN	C5orf51	UPF0600 protein C5orf51	10.13	10.22	52	6	0.928
sp|O43660|PLRG1_HUMAN	PLRG1	Pleiotropic regulator 1	28.61	29.42	55.6	15	0.929
sp|P61086|UBE2K_HUMAN	UBE2K	Ubiquitin-conjugating enzyme E2 K	20.48	21.49	75.5	20	0.929
sp|P55010|IF5_HUMAN	EIF5	Eukaryotic translation initiation factor 5	33.44	33.53	55.5	23	0.929
sp|Q8N1F7|NUP93_HUMAN	NUP93	Nuclear pore complex protein Nup93	68.28	68.35	66.2	52	0.929
sp|O95822|DCMC_HUMAN	MLYCD	Malonyl-CoA decarboxylase, mitochondrial	13.21	14.34	43.4	7	0.929
sp|Q9BRJ6|CG050_HUMAN	C7orf50	Uncharacterized protein C7orf50	12.02	12.04	58.3	6	0.929
sp|Q969V5|MUL1_HUMAN	MUL1	Mitochondrial ubiquitin ligase activator of NFKB 1	4.52	5.04	29.6	5	0.929
sp|Q9NVV0|TM38B_HUMAN	TMEM38B	Trimeric intracellular cation channel type B	4.6	4.64	22.7	4	0.929
sp|P51965|UB2E1_HUMAN	UBE2E1	Ubiquitin-conjugating enzyme E2 E1	3.43	4.15	32.6	5	0.930
sp|P62318|SMD3_HUMAN	SNRPD3	Small nuclear ribonucleoprotein Sm D3	8.29	10.23	65.1	24	0.930
sp|Q96CN7|ISOC1_HUMAN	ISOC1	Isochorismatase domain-containing protein 1	22.65	23.01	55.4	17	0.930
sp|Q96HD1|CREL1_HUMAN	CRELD1	Cysteine-rich with EGF-like domain protein 1	10.26	10.36	28.1	7	0.930
sp|Q9ULC4|MCTS1_HUMAN	MCTS1	Malignant T-cell-amplified sequence 1	19.3	19.36	76.2	15	0.930
sp|Q6WCQ1|MPRIP_HUMAN	MPRIP	Myosin phosphatase Rho-interacting protein	21.05	21.76	33.4	13	0.930
sp|Q9NPB8|GPCP1_HUMAN	GPCPD1	Glycerophosphocholine phosphodiesterase GPCPD1	10.43	11.31	26	8	0.930
sp|Q9H330|TM245_HUMAN	TMEM245	Transmembrane protein 245	4.21	5.85	16	4	0.930
sp|Q96GA7|SDSL_HUMAN	SDSL	Serine dehydratase-like	4.01	4.1	32.8	2	0.931
sp|Q53EL6|PDCD4_HUMAN	PDCD4	Programmed cell death protein 4	26.39	29.08	53.3	19	0.931
sp|Q15118|PDK1_HUMAN	PDK1	[Pyruvate dehydrogenase (acetyl-transferring)] kinase isozyme 1, mitochondrial	21.86	23.24	47.3	15	0.931
sp|O15382|BCAT2_HUMAN	BCAT2	Branched-chain-amino-acid aminotransferase, mitochondrial	17.94	18.17	49.5	13	0.931
sp|Q9NQ50|RM40_HUMAN	MRPL40	39S ribosomal protein L40, mitochondrial	11.52	12.05	56.3	10	0.931
sp|P52739|ZN131_HUMAN	ZNF131	Zinc finger protein 131	2.55	2.58	10.9	2	0.931
sp|O95352|ATG7_HUMAN	ATG7	Ubiquitin-like modifier-activating enzyme ATG7	21.58	21.69	30.3	12	0.931
sp|Q658P3|STEA3_HUMAN	STEAP3	Metalloreductase STEAP3	7.85	8	21.5	4	0.931
sp|P50053|KHK_HUMAN	KHK	Ketohexokinase	6.18	6.2	30.9	3	0.931
sp|Q92615|LAR4B_HUMAN	LARP4B	La-related protein 4B	20.61	21.17	32.5	15	0.931
sp|Q9UPN9|TRI33_HUMAN	TRIM33	E3 ubiquitin-protein ligase TRIM33	32.85	33.87	27.1	25	0.931
sp|Q5JTZ9|SYAM_HUMAN	AARS2	Alanine--tRNA ligase, mitochondrial	61.28	61.33	65.2	38	0.931
sp|Q8TC12|RDH11_HUMAN	RDH11	Retinol dehydrogenase 11	18.38	18.75	59.8	15	0.931
sp|P26641|EF1G_HUMAN	EEF1G	Elongation factor 1-gamma	55.55	55.85	81.5	69	0.931
sp|Q9BWT6|MND1_HUMAN	MND1	Meiotic nuclear division protein 1 homolog	2.51	2.98	44.9	3	0.932
sp|P80404|GABT_HUMAN	ABAT	4-aminobutyrate aminotransferase, mitochondrial	38.4	39.95	64	27	0.932
sp|O14974|MYPT1_HUMAN	PPP1R12A	Protein phosphatase 1 regulatory subunit 12A	35.64	36.09	39.7	22	0.932
sp|Q00325|MPCP_HUMAN	SLC25A3	Phosphate carrier protein, mitochondrial	34.29	34.39	44.8	32	0.932
sp|P13797|PLST_HUMAN	PLS3	Plastin-3	74.11	76.37	72.9	83	0.932
sp|Q9BQA9|CQ062_HUMAN	C17orf62	Uncharacterized protein C17orf62	5.67	5.75	26.7	3	0.932
sp|P50914|RL14_HUMAN	RPL14	60S ribosomal protein L14	14.84	17.84	67.9	20	0.932
sp|Q8N6M0|OTU6B_HUMAN	OTUD6B	OTU domain-containing protein 6B	12.92	13.09	44.7	7	0.932
sp|P23526|SAHH_HUMAN	AHCY	Adenosylhomocysteinase	51.02	51.07	67.8	60	0.932
sp|P36507|MP2K2_HUMAN	MAP2K2	Dual specificity mitogen-activated protein kinase kinase 2	20.94	21.28	54	16	0.932
sp|Q15942|ZYX_HUMAN	ZYX	Zyxin	30.36	30.82	44.1	25	0.932
sp|P46782|RS5_HUMAN	RPS5	40S ribosomal protein S5	21.84	21.92	54.4	45	0.932
sp|P98175|RBM10_HUMAN	RBM10	RNA-binding protein 10	32.87	33.04	36.7	18	0.932
sp|Q9P260|K1468_HUMAN	KIAA1468	LisH domain and HEAT repeat-containing protein KIAA1468	16.19	16.38	24.6	10	0.932

sp|P46937|YAP1_HUMAN	YAP1	Transcriptional coactivator YAP1	21.65	21.72	41.5	14	0.932
sp|Q00688|FKBP3_HUMAN	FKBP3	Peptidyl-prolyl cis-trans isomerase FKBP3	26.66	29.87	82.6	21	0.932
sp|Q7Z6Z7|HUWE1_HUMAN	HUWE1	E3 ubiquitin-protein ligase HUWE1	148.32	149.3	35.8	88	0.932
sp|Q9UQ13|SHOC2_HUMAN	SHOC2	Leucine-rich repeat protein SHOC-2	7.9	8.02	30.1	8	0.932
sp|P01116|RASK_HUMAN	KRAS	GTPase KRas	4	16.62	55	14	0.933
sp|Q8TBX8|PI42C_HUMAN	PIP4K2C	Phosphatidylinositol 5-phosphate 4-kinase type-2 gamma	12.07	12.38	41.8	8	0.933
sp|Q6VEQ5|WASH2_HUMAN	WASH2P	WAS protein family homolog 2	8.13	8.35	29.5	6	0.933
sp|P54098|DPOG1_HUMAN	POLG	DNA polymerase subunit gamma-1	25.7	26.61	25.4	16	0.933
sp|P24385|CCND1_HUMAN	CCND1	G1/S-specific cyclin-D1	2.29	2.32	15.9	2	0.933
sp|P02765|FETUA_HUMAN	AHSG	Alpha-2-HS-glycoprotein	19.41	19.5	47.1	12	0.933
sp|Q7Z6M4|MTEF4_HUMAN	MTERF4	Transcription termination factor 4, mitochondrial	8.68	9.31	32	6	0.933
sp|A8MT69|CENPX_HUMAN	STRA13	Centromere protein X	4.03	4.04	60.5	2	0.933
sp|Q9NXF1|TEX10_HUMAN	TEX10	Testis-expressed sequence 10 protein	27.6	28.73	32	20	0.933
sp|Q12929|EPS8_HUMAN	EPS8	Epidermal growth factor receptor kinase substrate 8	37.16	37.63	42.9	21	0.933
sp|Q99956|DUS9_HUMAN	DUSP9	Dual specificity protein phosphatase 9	14.45	14.96	42.2	10	0.933
sp|Q86SE8|NPM2_HUMAN	NPM2	Nucleoplasmin-2	4.01	4.01	30.8	2	0.933
sp|Q9Y2R5|RT17_HUMAN	MRPS17	28S ribosomal protein S17, mitochondrial	13.82	13.99	68.5	11	0.933
sp|P57678|GEMI4_HUMAN	GEMIN4	Gem-associated protein 4	43.38	46.79	42.1	28	0.933
sp|Q9BV68|RN126_HUMAN	RNF126	E3 ubiquitin-protein ligase RNF126	6	6	14.1	3	0.934
sp|Q96EY1|DNJA3_HUMAN	DNAJA3	DnaJ homolog subfamily A member 3, mitochondrial	30.76	30.91	48.5	22	0.934
sp|Q9GZN8|CT027_HUMAN	C20orf27	UPF0687 protein C20orf27	6.77	6.89	48.9	4	0.934
sp|O75528|TADA3_HUMAN	TADA3	Transcriptional adapter 3	12.03	12.14	40.1	8	0.934
sp|Q06587|RING1_HUMAN	RING1	E3 ubiquitin-protein ligase RING1	10.27	14.69	45.8	8	0.934
sp|P62380|TBPL1_HUMAN	TBPL1	TATA box-binding protein-like protein 1	9.94	10.03	41.9	6	0.934
sp|Q16181|SEPT7_HUMAN	SEPT7	Septin-7	45.3	47.36	68.7	37	0.934
sp|Q8WXD5|GEMI6_HUMAN	GEMIN6	Gem-associated protein 6	4.14	4.16	31.1	3	0.934
sp|P51532|SMCA4_HUMAN	SMARCA4	Transcription activator BRG1	71.35	72.61	37.6	40	0.934
sp|P07602|SAP_HUMAN	PSAP	Prosaposin	36.28	36.37	51.7	21	0.935
sp|P78346|RPP30_HUMAN	RPP30	Ribonuclease P protein subunit p30	23.39	23.47	64.2	14	0.935
sp|Q8TEX9|IPO4_HUMAN	IPO4	Importin-4	57.54	60.13	46.8	43	0.935
sp|Q9BV73|CP250_HUMAN	CEP250	Centrosome-associated protein CEP250	8.12	8.73	22.1	8	0.935
sp|Q7Z6J8|UBE3D_HUMAN	UBE3D	E3 ubiquitin-protein ligase E3D	4.71	4.79	27	3	0.935
sp|Q9BT78|CSN4_HUMAN	COPS4	COP9 signalosome complex subunit 4	44.2	44.45	82.8	30	0.935
sp|Q9NQX4|MYO5C_HUMAN	MYO5C	Unconventional myosin-Vc	2.5	12.56	21	9	0.935
sp|Q6P1X5|TAF2_HUMAN	TAF2	Transcription initiation factor TFIID subunit 2	19.35	22.57	27.4	15	0.935
sp|Q9NP74|PALMD_HUMAN	PALMD	Palmdelphin	11.46	14.06	32.7	9	0.935
sp|O75503|CLN5_HUMAN	CLN5	Ceroid-lipofuscinosis neuronal protein 5	8.02	8.05	22.1	4	0.935
sp|P10644|KAP0_HUMAN	PRKAR1A	cAMP-dependent protein kinase type I-alpha regulatory subunit	30.94	31.76	58	22	0.935
sp|Q99624|S38A3_HUMAN	SLC38A3	Sodium-coupled neutral amino acid transporter 3	4.89	4.95	17.9	3	0.935
sp|Q9GZT9|EGLN1_HUMAN	EGLN1	Egl nine homolog 1	20.81	21.14	46	12	0.935
sp|Q86VP6|CAND1_HUMAN	CAND1	Cullin-associated NEDD8-dissociated protein 1	93.23	94.8	54.6	61	0.936
sp|Q9NUW8|TYDP1_HUMAN	TDP1	Tyrosyl-DNA phosphodiesterase 1	16.91	17.02	30.6	9	0.936
sp|O60244|MED14_HUMAN	MED14	Mediator of RNA polymerase II transcription subunit 14	17.43	19.37	25.3	11	0.936
sp|O00505|IMA4_HUMAN	KPNA3	Importin subunit alpha-4	16.08	25.13	52	27	0.936
sp|Q96EF6|FBX17_HUMAN	FBXO17	F-box only protein 17	4.09	4.11	29.1	3	0.936
sp|Q99766|ATP5S_HUMAN	ATP5S	ATP synthase subunit s, mitochondrial	5.68	5.84	44.2	3	0.936
sp|Q15633|TRBP2_HUMAN	TARBP2	RISC-loading complex subunit TARBP2	5.6	5.7	33.1	4	0.936
sp|P84077|ARF1_HUMAN	ARF1	ADP-ribosylation factor 1	26.09	26.26	93.4	42	0.936
sp|O43290|SNUT1_HUMAN	SART1	U4/U6.U5 tri-snRNP-associated protein 1	52.04	53.87	59.3	28	0.936

sp|P35606|COPB2_HUMAN	COPB2	Coatomer subunit beta'	95.71	96.01	77.5	60	0.936
sp|P14625|ENPL_HUMAN	HSP90B1	Endoplasmin	122.08	133.44	73.1	154	0.936
sp|Q7L1W4|LRC8D_HUMAN	LRRC8D	Volume-regulated anion channel subunit LRRC8D	2.03	2.09	16.7	2	0.936
sp|P00918|CAH2_HUMAN	CA2	Carbonic anhydrase 2	28.3	28.37	64.2	22	0.936
sp|O43708|MAAI_HUMAN	GSTZ1	Maleylacetoacetate isomerase	9.44	9.73	59.3	10	0.936
sp|Q8TCU4|ALMS1_HUMAN	ALMS1	Alstrom syndrome protein 1	1.62	2.06	9.4	3	0.936
sp|Q96PU4|UHRF2_HUMAN	UHRF2	E3 ubiquitin-protein ligase UHRF2	9.65	10.29	18.5	6	0.936
sp|Q8WUD6|CHPT1_HUMAN	CHPT1	Cholinephosphotransferase 1	3.7	3.78	20.4	4	0.936
sp|P33552|CKS2_HUMAN	CKS2	Cyclin-dependent kinases regulatory subunit 2	1.41	1.64	53.2	2	0.937
sp|Q96I23|PREY_HUMAN	PYURF	Protein preY, mitochondrial	4.18	4.21	71.1	2	0.937
sp|Q9NPF4|OSGEP_HUMAN	OSGEP	Probable tRNA N6-adenosine threonylcarbamoyltransferase	16.86	17.03	52.2	10	0.937
sp|P55957|BID_HUMAN	BID	BH3-interacting domain death agonist	6	6.03	38.5	6	0.937
sp|Q08752|PPID_HUMAN	PPID	Peptidyl-prolyl cis-trans isomerase D	40.38	40.72	80.3	28	0.937
sp|P18031|PTN1_HUMAN	PTPN1	Tyrosine-protein phosphatase non-receptor type 1	36.69	36.79	59.3	22	0.937
sp|P12814|ACTN1_HUMAN	ACTN1	Alpha-actinin-1	118.48	118.72	82	116	0.937
sp|P16298|PP2BB_HUMAN	PPP3CB	Serine/threonine-protein phosphatase 2B catalytic subunit beta isoform	6.02	16.99	31.9	12	0.937
sp|P36776|LONM_HUMAN	LONP1	Lon protease homolog, mitochondrial	88.04	90.71	70.6	55	0.937
sp|Q9BQ69|MACD1_HUMAN	MACROD1	O-acetyl-ADP-ribose deacetylase MACROD1	11.55	12.15	44.3	8	0.937
sp|Q6NZY4|ZCHC8_HUMAN	ZCCHC8	Zinc finger CCHC domain-containing protein 8	28.68	28.82	46.5	15	0.938
sp|P46109|CRKL_HUMAN	CRKL	Crk-like protein	30.21	30.3	67.3	19	0.938
sp|Q7L576|CYFP1_HUMAN	CYFIP1	Cytoplasmic FMR1-interacting protein 1	58.08	59.85	45.3	34	0.938
sp|Q96EK7|F120B_HUMAN	FAM120B	Constitutive coactivator of peroxisome proliferator-activated receptor gamma	7.82	8.25	18.9	5	0.938
sp|Q9NPI6|DCP1A_HUMAN	DCP1A	mRNA-decapping enzyme 1A	20.91	21.1	38.5	11	0.938
sp|Q9Y3E1|HDGR3_HUMAN	HDGFRP3	Hepatoma-derived growth factor-related protein 3	5.91	8.18	33.5	5	0.938
sp|Q9UJC3|HOOK1_HUMAN	HOOK1	Protein Hook homolog 1	20.64	21.81	47.9	13	0.938
sp|O14715|RGPD8_HUMAN	RGPD8	RANBP2-like and GRIP domain-containing protein 8	4.78	67.57	42	41	0.938
sp|P12074|CX6A1_HUMAN	COX6A1	Cytochrome c oxidase subunit 6A1, mitochondrial	2.01	2.01	46.8	2	0.938
sp|P18754|RCC1_HUMAN	RCC1	Regulator of chromosome condensation	40.98	41.69	64.4	51	0.938
sp|Q86TP1|PRUNE_HUMAN	PRUNE	Protein prune homolog	10.01	10.01	25.2	5	0.938
sp|Q9NVX2|NLE1_HUMAN	NLE1	Notchless protein homolog 1	24.86	25.27	58.1	15	0.938
sp|P49761|CLK3_HUMAN	CLK3	Dual specificity protein kinase CLK3	5.44	5.54	16.5	3	0.939
sp|Q8WV60|PTCD2_HUMAN	PTCD2	Pentatricopeptide repeat-containing protein 2, mitochondrial	4.02	4.03	21.1	2	0.939
sp|Q5SW96|ARH_HUMAN	LDLRAP1	Low density lipoprotein receptor adapter protein 1	4	4	20.5	2	0.939
sp|O60884|DNJA2_HUMAN	DNAJA2	DnaJ homolog subfamily A member 2	35.45	36.19	74	30	0.939
sp|P56199|ITA1_HUMAN	ITGA1	Integrin alpha-1	8.23	8.82	17.6	8	0.939
sp|Q8WUH1|CHUR_HUMAN	CHURC1	Protein Churchill	5.14	5.21	36	3	0.939
sp|Q9NXF8|ZDHC7_HUMAN	ZDHHC7	Palmitoyltransferase ZDHHC7	2.87	2.93	10.4	2	0.939
sp|P52788|SPSY_HUMAN	SMS	Spermine synthase	25.26	25.52	49.5	18	0.939
sp|P51003|PAPOA_HUMAN	PAPOLA	Poly(A) polymerase alpha	36.94	37.56	45.1	20	0.939
sp|Q8NE01|CNNM3_HUMAN	CNNM3	Metal transporter CNNM3	18.64	19.27	26.9	10	0.939
sp|Q13523|PRP4B_HUMAN	PRPF4B	Serine/threonine-protein kinase PRP4 homolog	45.1	46.48	40.6	28	0.940
sp|Q15717|ELAV1_HUMAN	ELAVL1	ELAV-like protein 1	28.58	28.62	64.1	37	0.940
sp|Q9BZE1|RM37_HUMAN	MRPL37	39S ribosomal protein L37, mitochondrial	39.9	39.99	71.6	23	0.940
sp|P19838|NFKB1_HUMAN	NFKB1	Nuclear factor NF-kappa-B p105 subunit	29.59	30.48	36	20	0.940
sp|P78527|PRKDC_HUMAN	PRKDC	DNA-dependent protein kinase catalytic subunit	366.85	364.92	59.6	258	0.940
sp|Q8IY63|AMOL1_HUMAN	AMOTL1	Angiomotin-like protein 1	6.04	11.8	19	11	0.940
sp|Q15545|TAF7_HUMAN	TAF7	Transcription initiation factor TFIID subunit 7	7.24	7.44	36.4	4	0.940
sp|Q9H9V9|JMJD4_HUMAN	JMJD4	JmjC domain-containing protein 4	2.02	2.32	13.8	2	0.940
sp|O75608|LYPA1_HUMAN	LYPLA1	Acyl-protein thioesterase 1	6.82	8.74	57	7	0.940

sp|P08697|A2AP_HUMAN	SERPINF2	Alpha-2-antiplasmin	6.13	6.25	25.5	4	0.940
sp|Q96NT5|PCFT_HUMAN	SLC46A1	Proton-coupled folate transporter	1.49	1.59	9.2	2	0.940
sp|Q13895|BYST_HUMAN	BYSL	Bystin	28.23	33.36	61.3	22	0.941
sp|Q93034|CUL5_HUMAN	CUL5	Cullin-5	39.59	40.63	51.3	22	0.941
sp|Q9UI09|NDUAC_HUMAN	NDUFA12	NADH dehydrogenase [ubiquinone] 1 alpha subcomplex subunit 12	11.56	11.74	80	8	0.941
sp|Q9H3K2|GHITM_HUMAN	GHITM	Growth hormone-inducible transmembrane protein	11.66	11.84	31.9	7	0.941
sp|P00439|PH4H_HUMAN	PAH	Phenylalanine-4-hydroxylase	23.77	23.86	54.9	15	0.941
sp|Q9BU23|LMF2_HUMAN	LMF2	Lipase maturation factor 2	13.34	13.64	19	7	0.941
sp|Q06136|KDSR_HUMAN	KDSR	3-ketodihydrosphingosine reductase	4.01	4.03	22.9	2	0.941
sp|Q96F45|ZN503_HUMAN	ZNF503	Zinc finger protein 503	3.84	6.38	16.7	4	0.941
sp|P51553|IDH3G_HUMAN	IDH3G	Isocitrate dehydrogenase [NAD] subunit gamma, mitochondrial	20.59	21.17	56.2	14	0.941
sp|Q14318|FKBP8_HUMAN	FKBP8	Peptidyl-prolyl cis-trans isomerase FKBP8	19.72	19.78	43	17	0.941
sp|Q7L2E3|DHX30_HUMAN	DHX30	Putative ATP-dependent RNA helicase DHX30	102.45	105.89	73.3	55	0.941
sp|Q9UHG0|DCDC2_HUMAN	DCDC2	Doublecortin domain-containing protein 2	4.15	4.24	19.1	2	0.941
sp|P08621|RU17_HUMAN	SNRNP70	U1 small nuclear ribonucleoprotein 70 kDa	24.94	25.12	43	17	0.941
sp|Q9UK73|FEM1B_HUMAN	FEM1B	Protein fem-1 homolog B	1.37	3.18	18.8	5	0.941
sp|Q9UH99|SUN2_HUMAN	SUN2	SUN domain-containing protein 2	34.64	35.24	47	23	0.941
sp|Q96HY7|DHTK1_HUMAN	DHTKD1	Probable 2-oxoglutarate dehydrogenase E1 component DHKTD1, mitochondrial	27.79	30.17	35	16	0.942
sp|Q9H9S5|FKRP_HUMAN	FKRP	Fukutin-related protein	2.44	2.5	17	2	0.942
sp|Q8NEM7|SP20H_HUMAN	SUPT20H	Transcription factor SPT20 homolog	3.44	3.93	15	3	0.942
sp|Q9UHW9|S12A6_HUMAN	SLC12A6	Solute carrier family 12 member 6	1.92	4.95	16.1	6	0.942
sp|Q9BUQ8|DDX23_HUMAN	DDX23	Probable ATP-dependent RNA helicase DDX23	43.29	44.9	52.1	23	0.942
sp|Q9UKL3|C8AP2_HUMAN	CASP8AP2	CASP8-associated protein 2	2.04	2.18	14.1	3	0.942
sp|O43310|CTIF_HUMAN	CTIF	CBP80/20-dependent translation initiation factor	4.64	4.73	25.9	2	0.942
sp|P43490|NAMPT_HUMAN	NAMPT	Nicotinamide phosphoribosyltransferase	38.87	39.26	77	25	0.942
sp|P19784|CSK22_HUMAN	CSNK2A2	Casein kinase II subunit alpha'	27.01	31.81	65.4	22	0.942
sp|P60484|PTEN_HUMAN	PTEN	Phosphatidylinositol 3,4,5-trisphosphate 3-phosphatase and dual-specificity protein phosphatase PTEN	2	2.12	14.6	2	0.942
sp|P62873|GBB1_HUMAN	GNB1	Guanine nucleotide-binding protein G(I)/G(S)/G(T) subunit beta-1	16.56	27.82	67.4	29	0.942
sp|Q9Y3P4|RHBD3_HUMAN	RHBDD3	Rhomboid domain-containing protein 3	2	2	9.3	3	0.943
sp|Q02224|CENPE_HUMAN	CENPE	Centromere-associated protein E	2.79	9.26	30.5	14	0.943
sp|O75152|ZC11A_HUMAN	ZC3H11A	Zinc finger CCCH domain-containing protein 11A	46	47.84	53.6	34	0.943
sp|P60228|EIF3E_HUMAN	EIF3E	Eukaryotic translation initiation factor 3 subunit E	38.42	38.5	72.4	31	0.943
sp|Q00537|CDK17_HUMAN	CDK17	Cyclin-dependent kinase 17	3.2	9.5	33.8	5	0.943
sp|Q13442|HAP28_HUMAN	PDAP1	28 kDa heat- and acid-stable phosphoprotein	15.25	15.32	45.9	9	0.943
sp|P00352|AL1A1_HUMAN	ALDH1A1	Retinal dehydrogenase 1	69.32	70.23	85.4	83	0.943
sp|O60645|EXOC3_HUMAN	EXOC3	Exocyst complex component 3	16.72	17.11	36	9	0.943
sp|O75396|SC22B_HUMAN	SEC22B	Vesicle-trafficking protein SEC22b	12.38	12.53	79.1	22	0.943
sp|P11279|LAMP1_HUMAN	LAMP1	Lysosome-associated membrane glycoprotein 1	11.01	12.17	21.1	11	0.943
sp|P15586|GNS_HUMAN	GNS	N-acetylglucosamine-6-sulfatase	25.17	26.08	41.1	15	0.943
sp|Q9C0C7|AMRA1_HUMAN	AMBRA1	Activating molecule in BECN1-regulated autophagy protein 1	2.5	2.57	6.2	2	0.943
sp|Q04721|NOTC2_HUMAN	NOTCH2	Neurogenic locus notch homolog protein 2	17.82	17.89	9	11	0.943
sp|Q9NQH7|XPP3_HUMAN	XPNPEP3	Probable Xaa-Pro aminopeptidase 3	21.27	23.35	49.5	14	0.943
sp|Q13423|NNTM_HUMAN	NNT	NAD(P) transhydrogenase, mitochondrial	67.81	69.61	50.8	43	0.944
sp|O14641|DVL2_HUMAN	DVL2	Segment polarity protein dishevelled homolog DVL-2	8.77	9.04	26.6	7	0.944
sp|Q9H7C9|AAMDC_HUMAN	AAMDC	Mth938 domain-containing protein	11.66	11.73	73.8	7	0.944
sp|Q9P0L0|VAPA_HUMAN	VAPA	Vesicle-associated membrane protein-associated protein A	21.7	21.85	71.9	32	0.944
sp|Q8IX01|SUGP2_HUMAN	SUGP2	SURP and G-patch domain-containing protein 2	53.68	53.96	44.4	28	0.944
sp|P20645|MPRD_HUMAN	M6PR	Cation-dependent mannose-6-phosphate receptor	8.48	11.89	41.2	10	0.944
sp|Q9UJC5|SH3L2_HUMAN	SH3BGRL2	SH3 domain-binding glutamic acid-rich-like protein 2	4.42	4.45	74.8	3	0.944

sp|O94888|UBXN7_HUMAN	UBXN7	UBX domain-containing protein 7	11.47	12.55	33.3	11	0.944
sp|Q16643|DREB_HUMAN	DBN1	Drebrin	39.99	40.05	60.3	29	0.944
sp|Q7L5N1|CSN6_HUMAN	COPS6	COP9 signalosome complex subunit 6	27.56	27.73	57.8	18	0.944
sp|Q9BTV5|FSD1_HUMAN	FSD1	Fibronectin type III and SPRY domain-containing protein 1	3.79	4.08	21	4	0.944
sp|Q7Z5K2|WAPL_HUMAN	WAPAL	Wings apart-like protein homolog	16.43	17.11	26.5	11	0.944
sp|Q96EL2|RT24_HUMAN	MRPS24	28S ribosomal protein S24, mitochondrial	9.32	9.45	52.1	7	0.945
sp|P38432|COIL_HUMAN	COIL	Coilin	17.61	18	38.9	11	0.945
sp|Q49AR2|CE022_HUMAN	C5orf22	UPF0489 protein C5orf22	8.01	8.07	22.9	7	0.945
sp|P54687|BCAT1_HUMAN	BCAT1	Branched-chain-amino-acid aminotransferase, cytosolic	13.1	13.67	39.4	8	0.945
sp|Q8WYQ5|DGCR8_HUMAN	DGCR8	Microprocessor complex subunit DGCR8	3.53	3.71	15.7	3	0.945
sp|Q96A57|TM230_HUMAN	TMEM230	Transmembrane protein 230	7.46	7.52	45.8	4	0.945
sp|Q15526|SURF1_HUMAN	SURF1	Surfeit locus protein 1	6.43	6.58	41.7	6	0.945
sp|Q9NWY4|CD027_HUMAN	C4orf27	UPF0609 protein C4orf27	12.86	13.07	52	7	0.945
sp|Q5JTV8|TOIP1_HUMAN	TOR1AIP1	Torsin-1A-interacting protein 1	34	34.41	51.1	27	0.945
sp|P61254|RL26_HUMAN	RPL26	60S ribosomal protein L26	19.94	20.2	64.8	14	0.945
sp|Q70CQ3|UBP30_HUMAN	USP30	Ubiquitin carboxyl-terminal hydrolase 30	2.99	3.62	28.6	4	0.945
sp|Q9Y5K6|CD2AP_HUMAN	CD2AP	CD2-associated protein	32.77	34.93	54.6	19	0.945
sp|Q08722|CD47_HUMAN	CD47	Leukocyte surface antigen CD47	5.57	5.72	17.7	5	0.945
sp|Q96HY6|DDRGK_HUMAN	DDRGK1	DDRGK domain-containing protein 1	14.29	14.37	56.1	13	0.945
sp|Q9P2M7|CING_HUMAN	CGN	Cingulin	6.37	6.75	26.9	4	0.946
sp|Q96N67|DOCK7_HUMAN	DOCK7	Dedicator of cytokinesis protein 7	62.71	65.13	35.1	38	0.946
sp|O00400|ACATN_HUMAN	SLC33A1	Acetyl-coenzyme A transporter 1	4.58	4.78	17.7	3	0.946
sp|Q6P582|MZT2A_HUMAN	MZT2A	Mitotic-spindle organizing protein 2A	6.69	6.8	50.6	4	0.946
sp|Q9Y5Z9|UBIA1_HUMAN	UBIAD1	UbiA prenyltransferase domain-containing protein 1	2.42	2.54	11.2	3	0.946
sp|P04843|RPN1_HUMAN	RPN1	Dolichyl-diphosphooligosaccharide--protein glycosyltransferase subunit 1	72.12	74.73	73.8	76	0.946
sp|Q8TD16|BICD2_HUMAN	BICD2	Protein bicaudal D homolog 2	22.86	24.33	32.3	15	0.946
sp|Q9Y5J6|T10B_HUMAN	TIMM10B	Mitochondrial import inner membrane translocase subunit Tim10 B	5.34	5.44	47.6	4	0.946
sp|P35244|RFA3_HUMAN	RPA3	Replication protein A 14 kDa subunit	15.8	15.87	87.6	14	0.946
sp|Q9BSJ5|CQ080_HUMAN	C17orf80	Uncharacterized protein C17orf80	1.93	2.12	16.4	2	0.946
sp|O95671|ASML_HUMAN	ASMTL	N-acetylserotonin O-methyltransferase-like protein	18.42	18.6	40.4	11	0.946
sp|P51948|MAT1_HUMAN	MNAT1	CDK-activating kinase assembly factor MAT1	7.33	7.43	42.1	6	0.946
sp|Q8WXX5|DNJC9_HUMAN	DNAJC9	DnaJ homolog subfamily C member 9	25.93	26.17	64.6	16	0.946
sp|Q5JVF3|PCID2_HUMAN	PCID2	PCI domain-containing protein 2	22.96	24.24	68.7	18	0.946
sp|Q9UMX0|UBQL1_HUMAN	UBQLN1	Ubiquilin-1	17.96	25.86	50.8	32	0.946
sp|O75688|PPM1B_HUMAN	PPM1B	Protein phosphatase 1B	24.1	25.59	50.1	17	0.946
sp|A9UHW6|MI4GD_HUMAN	MIF4GD	MIF4G domain-containing protein	5.26	6.63	43.2	6	0.947
sp|O43159|RRP8_HUMAN	RRP8	Ribosomal RNA-processing protein 8	15.84	16.01	35.5	12	0.947
sp|Q13951|PEBB_HUMAN	CBFB	Core-binding factor subunit beta	9.81	11.11	48.9	7	0.947
sp|Q8IY18|SMC5_HUMAN	SMC5	Structural maintenance of chromosomes protein 5	21.33	22.8	40.1	14	0.947
sp|Q9BQ24|ZFY21_HUMAN	ZFYVE21	Zinc finger FYVE domain-containing protein 21	5.92	6	28.6	3	0.947
sp|Q9BRP8|WIBG_HUMAN	WIBG	Partner of Y14 and mago	16.56	16.64	79.4	9	0.947
sp|O95487|SC24B_HUMAN	SEC24B	Protein transport protein Sec24B	17.35	22.21	21.4	14	0.947
sp|Q8N490|PNKD_HUMAN	PNKD	Probable hydrolase PNKD	2.4	2.86	10.9	3	0.947
sp|Q9BQ15|SOSB1_HUMAN	NABP2	SS complex subunit B1	2.63	2.66	32.7	2	0.947
sp|Q13541|4EBP1_HUMAN	EIF4EBP1	Eukaryotic translation initiation factor 4E-binding protein 1	8.94	9.39	69.5	10	0.947
sp|Q13564|ULA1_HUMAN	NAE1	NEDD8-activating enzyme E1 regulatory subunit	26.15	26.3	56.6	20	0.947
sp|Q9UNL2|SSRG_HUMAN	SSR3	Translocon-associated protein subunit gamma	2	2.14	21.1	2	0.947
sp|Q9H857|NT5D2_HUMAN	NT5DC2	5'-nucleotidase domain-containing protein 2	39.73	40.07	70.4	27	0.947
sp|Q96BI3|APH1A_HUMAN	APH1A	Gamma-secretase subunit APH-1A	2.51	2.61	6.8	2	0.947

sp|Q8NDF8|PAPD5_HUMAN	PAPD5	Non-canonical poly(A) RNA polymerase PAPD5	8.19	8.5	32.2	7	0.947
sp|O43837|IDH3B_HUMAN	IDH3B	Isocitrate dehydrogenase [NAD] subunit beta, mitochondrial	23.14	23.75	59.7	28	0.947
sp|Q9NX40|OCAD1_HUMAN	OCIAD1	OCIA domain-containing protein 1	12.84	13.02	32.7	9	0.948
sp|Q8N9N7|LRC57_HUMAN	LRRC57	Leucine-rich repeat-containing protein 57	12.75	12.9	53.1	8	0.948
sp|Q9BV44|THUM3_HUMAN	THUMPD3	THUMP domain-containing protein 3	13.99	14.06	31.8	10	0.948
sp|Q9Y512|SAM50_HUMAN	SAMM50	Sorting and assembly machinery component 50 homolog	39.09	41.43	69.7	25	0.948
sp|Q8NB49|AT11C_HUMAN	ATP11C	Phospholipid-transporting ATPase IG	35.3	36.13	36.1	24	0.948
sp|Q96LI5|CNO6L_HUMAN	CNOT6L	CCR4-NOT transcription complex subunit 6-like	2.18	6.88	20.4	4	0.948
sp|P15291|B4GT1_HUMAN	B4GALT1	Beta-1,4-galactosyltransferase 1	2	2.01	11.1	2	0.948
sp|Q9BV38|WDR18_HUMAN	WDR18	WD repeat-containing protein 18	17.62	17.68	35.4	15	0.948
sp|P55036|PSMD4_HUMAN	PSMD4	26S proteasome non-ATPase regulatory subunit 4	29.97	30.05	53.1	35	0.948
sp|P09110|THIK_HUMAN	ACAA1	3-ketoacyl-CoA thiolase, peroxisomal	38.21	38.24	83	27	0.948
sp|Q9HC36|MRM3_HUMAN	RNMTL1	rRNA methyltransferase 3, mitochondrial	14	16.88	40.5	11	0.948
sp|Q15653|IKBB_HUMAN	NFKBIB	NF-kappa-B inhibitor beta	1.79	1.95	18.3	2	0.949
sp|Q9GZS1|RPA49_HUMAN	POLR1E	DNA-directed RNA polymerase I subunit RPA49	20.13	20.31	42.8	13	0.949
sp|Q6UXH1|CREL2_HUMAN	CRELD2	Cysteine-rich with EGF-like domain protein 2	4.97	5.04	13.6	3	0.949
sp|Q969V6|MKL1_HUMAN	MKL1	MKL/myocardin-like protein 1	4.83	5.07	17.9	6	0.949
sp|Q9Y4E5|ZN451_HUMAN	ZNF451	Zinc finger protein 451	3.94	4.26	12.7	3	0.949
sp|Q86W34|AMZ2_HUMAN	AMZ2	Archaemetzincin-2	3.16	3.82	23.3	5	0.949
sp|Q9UK59|DBR1_HUMAN	DBR1	Lariat debranching enzyme	14.45	15.39	39.5	12	0.949
sp|Q06330|SUH_HUMAN	RBPJ	Recombining binding protein suppressor of hairless	16.71	16.81	31	12	0.949
sp|A6NHR9|SMHD1_HUMAN	SMCHD1	Structural maintenance of chromosomes flexible hinge domain-containing protein 1	86.35	87.87	47.6	46	0.950
sp|Q86VM9|ZCH18_HUMAN	ZC3H18	Zinc finger CCCH domain-containing protein 18	13.08	14.52	20.6	12	0.950
sp|Q96N46|TTC14_HUMAN	TTC14	Tetratricopeptide repeat protein 14	5.26	5.47	19.7	3	0.950
sp|Q13415|ORC1_HUMAN	ORC1	Origin recognition complex subunit 1	5.22	5.42	15.9	3	0.950
sp|O14548|COX7R_HUMAN	COX7A2L	Cytochrome c oxidase subunit 7A-related protein, mitochondrial	9.22	9.29	72.8	6	0.950
sp|Q9UL46|PSME2_HUMAN	PSME2	Proteasome activator complex subunit 2	26.38	28.89	82.4	30	0.950
sp|Q15155|NOMO1_HUMAN	NOMO1	Nodal modulator 1	2	88.29	69.2	64	0.950
sp|Q9UIU6|SIX4_HUMAN	SIX4	Homeobox protein SIX4	2.07	2.1	10.1	2	0.950
sp|Q9Y606|TRUA_HUMAN	PUS1	tRNA pseudouridine synthase A, mitochondrial	29.16	30.25	47.5	16	0.950
sp|O96019|ACL6A_HUMAN	ACTL6A	Actin-like protein 6A	31.22	31.53	51.1	21	0.950
sp|P50402|EMD_HUMAN	EMD	Emerin	19.87	20.01	70.9	15	0.950
sp|Q0JRZ9|FCHO2_HUMAN	FCHO2	F-BAR domain only protein 2	6.27	8.4	28.2	5	0.950
sp|O14556|G3PT_HUMAN	GAPDHS	Glyceraldehyde-3-phosphate dehydrogenase, testis-specific	2.12	3.99	26	12	0.950
sp|Q9NR45|SIAS_HUMAN	NANS	Sialic acid synthase	33	33.03	79.9	32	0.950
sp|P22061|PIMT_HUMAN	PCMT1	Protein-L-isoaspartate(D-aspartate) O-methyltransferase	28.81	29.84	84.6	34	0.951
sp|Q13740|CD166_HUMAN	ALCAM	CD166 antigen	33.63	33.71	52.5	24	0.951
sp|O60443|DFNA5_HUMAN	DFNA5	Non-syndromic hearing impairment protein 5	5.59	6.12	25.2	6	0.951
sp|Q9Y3D7|TIM16_HUMAN	PAM16	Mitochondrial import inner membrane translocase subunit TIM16	4	4.25	40.8	5	0.951
sp|Q14146|URB2_HUMAN	URB2	Unhealthy ribosome biogenesis protein 2 homolog	34.91	37.2	29.9	22	0.951
sp|Q9H0S4|DDX47_HUMAN	DDX47	Probable ATP-dependent RNA helicase DDX47	34.19	34.32	57.8	21	0.951
sp|Q96C90|PP14B_HUMAN	PPP1R14B	Protein phosphatase 1 regulatory subunit 14B	12.82	12.89	68	8	0.951
sp|Q03164|KMT2A_HUMAN	KMT2A	Histone-lysine N-methyltransferase 2A	49.08	49.8	19	27	0.951
sp|Q8N5S9|KKCC1_HUMAN	CAMKK1	Calcium/calmodulin-dependent protein kinase kinase 1	2.02	2.54	18.4	2	0.951
sp|Q06124|PTN11_HUMAN	PTPN11	Tyrosine-protein phosphatase non-receptor type 11	46.02	48.7	61.6	29	0.951
sp|Q96GS4|CQ059_HUMAN	C17orf59	Uncharacterized protein C17orf59	6	6.01	19.9	3	0.951
sp|P54577|SYYC_HUMAN	YARS	Tyrosine--tRNA ligase, cytoplasmic	96.71	99.54	84.9	73	0.951
sp|Q93008|USP9X_HUMAN	USP9X	Probable ubiquitin carboxyl-terminal hydrolase FAF-X	104.99	111.09	42	64	0.951
sp|Q7KYR7|BT2A1_HUMAN	BTN2A1	Butyrophilin subfamily 2 member A1	10.63	10.76	33	7	0.951

sp|P41091|IF2G_HUMAN	EIF2S3	Eukaryotic translation initiation factor 2 subunit 3	41.92	44.59	64.2	40	0.952
sp|O00116|ADAS_HUMAN	AGPS	Alkyldihydroxyacetonephosphate synthase, peroxisomal	52.23	52.53	61.1	34	0.952
sp|P35754|GLRX1_HUMAN	GLRX	Glutaredoxin-1	9.91	10.67	88.7	17	0.952
sp|Q9UQ35|SRRM2_HUMAN	SRRM2	Serine/arginine repetitive matrix protein 2	78.77	78.82	33	58	0.952
sp|Q6STE5|SMRD3_HUMAN	SMARCD3	SWI/SNF-related matrix-associated actin-dependent regulator of chromatin subfamily D member 3	2	8	29.4	6	0.952
sp|Q6ZNB6|NFXL1_HUMAN	NFXL1	NF-X1-type zinc finger protein NFXL1	27.17	27.47	36.9	13	0.952
sp|O15226|NKRF_HUMAN	NKRF	NF-kappa-B-repressing factor	54.12	54.22	56.2	31	0.952
sp|O94826|TOM70_HUMAN	TOMM70A	Mitochondrial import receptor subunit TOM70	75.87	78.15	74.8	67	0.952
sp|Q8IVM0|CCD50_HUMAN	CCDC50	Coiled-coil domain-containing protein 50	5.77	6.09	34	6	0.952
sp|Q9H944|MED20_HUMAN	MED20	Mediator of RNA polymerase II transcription subunit 20	10	10.19	42	6	0.952
sp|P55265|DSRAD_HUMAN	ADAR	Double-stranded RNA-specific adenosine deaminase	92.68	94.45	51.6	72	0.952
sp|Q8WZ82|OVCA2_HUMAN	OVCA2	Ovarian cancer-associated gene 2 protein	7.66	7.73	41.9	4	0.952
sp|Q9UHD9|UBQL2_HUMAN	UBQLN2	Ubiquilin-2	8.4	21.78	47.3	26	0.952
sp|P42695|CNDD3_HUMAN	NCAPD3	Condensin-2 complex subunit D3	23.04	23.46	26.6	11	0.952
sp|P62753|RS6_HUMAN	RPS6	40S ribosomal protein S6	31.24	33.52	57.4	28	0.952
sp|Q9NUK0|MBNL3_HUMAN	MBNL3	Muscleblind-like protein 3	2	8	16.4	4	0.953
sp|Q96CB8|INT12_HUMAN	INTS12	Integrator complex subunit 12	1.88	2.12	16.5	3	0.953
sp|Q9NZU5|LMCD1_HUMAN	LMCD1	LIM and cysteine-rich domains protein 1	2.61	2.67	30.4	3	0.953
sp|Q15853|USF2_HUMAN	USF2	Upstream stimulatory factor 2	8.54	8.79	21.1	6	0.953
sp|Q9UIG0|BAZ1B_HUMAN	BAZ1B	Tyrosine-protein kinase BAZ1B	75.02	76.36	46.2	50	0.953
sp|Q8WXF1|PSPC1_HUMAN	PSPC1	Paraspeckle component 1	43.06	46.49	67.5	44	0.953
sp|Q99623|PHB2_HUMAN	PHB2	Prohibitin-2	50.23	50.28	83.6	58	0.953
sp|Q9Y546|LRC42_HUMAN	LRRC42	Leucine-rich repeat-containing protein 42	2.68	2.93	22.9	3	0.953
sp|Q9NPE3|NOP10_HUMAN	NOP10	H/ACA ribonucleoprotein complex subunit 3	4.28	5.16	85.9	6	0.953
sp|P68366|TBA4A_HUMAN	TUBA4A	Tubulin alpha-4A chain	6.86	81.85	78.8	240	0.953
sp|Q9NXF7|DCA16_HUMAN	DCAF16	DDB1- and CUL4-associated factor 16	2.59	3.54	16.7	3	0.953
sp|Q96KR6|F210B_HUMAN	FAM210B	Protein FAM210B	3.56	3.68	33.3	2	0.953
sp|Q9H1D9|RPC6_HUMAN	POLR3F	DNA-directed RNA polymerase III subunit RPC6	12.54	14.81	39.9	8	0.953
sp|Q9NPE2|NGRN_HUMAN	NGRN	Neugrin	6.43	6.66	20.6	4	0.953
sp|Q0IIM8|TBC8B_HUMAN	TBC1D8B	TBC1 domain family member 8B	2.2	4.21	14.6	3	0.953
sp|Q9Y6E2|BZW2_HUMAN	BZW2	Basic leucine zipper and W2 domain-containing protein 2	32.39	41.62	57.8	23	0.953
sp|P52569|CTR2_HUMAN	SLC7A2	Cationic amino acid transporter 2	2.05	4.08	19.6	2	0.953
sp|Q6P1J9|CDC73_HUMAN	CDC73	Parafibromin	44.69	44.95	55.4	24	0.954
sp|Q14566|MCM6_HUMAN	MCM6	DNA replication licensing factor MCM6	69.99	70.3	57.9	44	0.954
sp|O75882|ATRN_HUMAN	ATRN	Attractin	7.45	7.55	11.6	4	0.954
sp|O60637|TSN3_HUMAN	TSPAN3	Tetraspanin-3	3.54	3.62	17.4	2	0.954
sp|O75937|DNJC8_HUMAN	DNAJC8	DnaJ homolog subfamily C member 8	16.55	16.68	58.5	16	0.954
sp|Q53H82|LACB2_HUMAN	LACTB2	Beta-lactamase-like protein 2	18.06	18.19	61.5	11	0.955
sp|Q96EV2|RBM33_HUMAN	RBM33	RNA-binding protein 33	8.18	8.81	19	5	0.955
sp|Q9H6Y2|WDR55_HUMAN	WDR55	WD repeat-containing protein 55	12.45	13.08	53.3	11	0.955
sp|Q86TB9|PATL1_HUMAN	PATL1	Protein PAT1 homolog 1	28.02	28.38	39.5	15	0.955
sp|Q9NVE7|PANK4_HUMAN	PANK4	Pantothenate kinase 4	17.79	18.22	38.8	16	0.955
sp|O14965|AURKA_HUMAN	AURKA	Aurora kinase A	16.27	16.67	51.4	10	0.955
sp|P14854|CX6B1_HUMAN	COX6B1	Cytochrome c oxidase subunit 6B1	11.4	11.92	79.1	15	0.955
sp|Q9H1B7|I2BPL_HUMAN	IRF2BPL	Interferon regulatory factor 2-binding protein-like	6.79	11.73	24.1	7	0.955
sp|O75400|PR40A_HUMAN	PRPF40A	Pre-mRNA-processing factor 40 homolog A	52.37	53.01	41.1	36	0.955
sp|Q9NZM5|GSCR2_HUMAN	GLTSCR2	Glioma tumor suppressor candidate region gene 2 protein	13.87	14.57	36.8	8	0.955
sp|P54920|SNAA_HUMAN	NAPA	Alpha-soluble NSF attachment protein	26.5	26.75	82.4	20	0.955
sp|A6NKF1|SAC31_HUMAN	SAC3D1	SAC3 domain-containing protein 1	7.4	8.69	22.3	5	0.955

sp|P29083|T2EA_HUMAN	GTF2E1	General transcription factor IIE subunit 1	4.4	4.49	27.3	4		0.955
sp|O43521|B2L11_HUMAN	BCL2L11	Bcl-2-like protein 11	3.7	3.8	15.2	2		0.955
sp|P11177|ODPB_HUMAN	PDHB	Pyruvate dehydrogenase E1 component subunit beta, mitochondrial	29.34	29.47	68.5	44		0.955
sp|Q9H9F9|ARP5_HUMAN	ACTR5	Actin-related protein 5	16.74	16.8	37.1	10		0.955
sp|O95239|KIF4A_HUMAN	KIF4A	Chromosome-associated kinesin KIF4A	42.91	44.94	42.9	25		0.955
sp|Q8N442|GUF1_HUMAN	GUF1	Translation factor GUF1, mitochondrial	22.12	22.29	42.8	14		0.956
sp|P04183|KITH_HUMAN	TK1	Thymidine kinase, cytosolic	17.32	17.47	55.6	12		0.956
sp|O75874|IDHC_HUMAN	IDH1	Isocitrate dehydrogenase [NADP] cytoplasmic	58.57	60.21	84.5	78		0.956
sp|P28289|TMOD1_HUMAN	TMOD1	Tropomodulin-1	4.01	6.17	20.3	4		0.956
sp|Q9Y314|NOSIP_HUMAN	NOSIP	Nitric oxide synthase-interacting protein	16.01	16.31	69.4	12		0.956
sp|Q8N806|UBR7_HUMAN	UBR7	Putative E3 ubiquitin-protein ligase UBR7	22.12	22.52	58.6	15		0.956
sp|Q8WV92|MITD1_HUMAN	MITD1	MIT domain-containing protein 1	2.53	2.65	37.8	2		0.956
sp|Q8WVY7|UBCP1_HUMAN	UBLCP1	Ubiquitin-like domain-containing CTD phosphatase 1	16.69	17.45	48.1	12		0.956
sp|O00139|KIF2A_HUMAN	KIF2A	Kinesin-like protein KIF2A	42.09	43.79	49.3	29		0.956
sp|P10155|RO60_HUMAN	TROVE2	60 kDa SS-A/Ro ribonucleoprotein	37.36	37.55	62.3	27		0.956
sp|Q5QJ74|TBCEL_HUMAN	TBCEL	Tubulin-specific chaperone cofactor E-like protein	6.05	6.06	23.4	3		0.956
sp|Q92747|ARC1A_HUMAN	ARPC1A	Actin-related protein 2/3 complex subunit 1A	15.12	21.22	50	18		0.956
sp|P30740|ILEU_HUMAN	SERPINB1	Leukocyte elastase inhibitor	34.23	34.36	76.5	21		0.957
sp|P49641|MA2A2_HUMAN	MAN2A2	Alpha-mannosidase 2x	6	7.96	8.3	4		0.957
sp|Q96BJ3|AIDA_HUMAN	AIDA	Axin interactor, dorsalization-associated protein	10.44	10.61	47.1	5		0.957
sp|Q9BRK4|LZTS2_HUMAN	LZTS2	Leucine zipper putative tumor suppressor 2	2.05	2.06	22.1	2		0.957
sp|Q9UNN5|FAF1_HUMAN	FAF1	FAS-associated factor 1	32.09	33.44	42.9	18		0.957
sp|Q9NX55|HYPK_HUMAN	HYPK	Huntingtin-interacting protein K	14.98	15.74	81.4	12		0.957
sp|P62877|RBX1_HUMAN	RBX1	E3 ubiquitin-protein ligase RBX1	5.94	6.06	53.7	6		0.957
sp|Q15392|DHC24_HUMAN	DHCR24	Delta(24)-sterol reductase	17.76	18.24	25.8	10		0.957
sp|Q6UX04|CWC27_HUMAN	CWC27	Peptidyl-prolyl cis-trans isomerase CWC27 homolog	14.23	14.49	41.1	8		0.957
sp|Q8NBL1|PGLT1_HUMAN	POGLUT1	Protein O-glucosyltransferase 1	5.89	6.04	28.6	4		0.957
sp|Q4V328|GRAP1_HUMAN	GRIPAP1	GRIP1-associated protein 1	31.8	33.45	50.1	17		0.958
sp|Q9NRG0|CHRC1_HUMAN	CHRAC1	Chromatin accessibility complex protein 1	7.52	7.64	65.7	8		0.958
sp|Q6RW13|ATRAP_HUMAN	AGTRAP	Type-1 angiotensin II receptor-associated protein	2	2.01	18.2	2		0.958
sp|Q9BPW8|NIPS1_HUMAN	NIPSNAP1	Protein NipSnap homolog 1	23.54	23.7	79.9	19		0.958
sp|Q8IVS2|FABD_HUMAN	MCAT	Malonyl-CoA-acyl carrier protein transacylase, mitochondrial	22.3	22.34	62.3	18		0.958
sp|Q9BUR4|WAP53_HUMAN	WRAP53	Telomerase Cajal body protein 1	22.06	22.07	38.3	12		0.958
sp|Q5F1R6|DJC21_HUMAN	DNAJC21	DnaJ homolog subfamily C member 21	7.62	10.16	29.4	6		0.958
sp|Q9BXB4|OSB11_HUMAN	OSBPL11	Oxysterol-binding protein-related protein 11	8.06	8.15	22.2	4		0.958
sp|Q00587|BORG5_HUMAN	CDC42EP1	Cdc42 effector protein 1	7.35	7.56	32.7	4		0.958
sp|P52209|6PGD_HUMAN	PGD	6-phosphogluconate dehydrogenase, decarboxylating	53.82	53.98	64.6	37		0.958
sp|Q6NT16|S18B1_HUMAN	SLC18B1	MFS-type transporter SLC18B1	3.2	3.29	10.8	3		0.958
sp|P27824|CALX_HUMAN	CANX	Calnexin	80.03	80.08	76.2	72		0.958
sp|Q16595|FRDA_HUMAN	FXN	Frataxin, mitochondrial	11.91	11.98	45.2	8		0.958
sp|Q15262|PTPRK_HUMAN	PTPRK	Receptor-type tyrosine-protein phosphatase kappa	19.06	19.29	20.2	12		0.958
sp|Q969M1|TM40L_HUMAN	TOMM40L	Mitochondrial import receptor subunit TOM40B	5.82	5.91	22.1	4		0.958
sp|Q9Y617|SERC_HUMAN	PSAT1	Phosphoserine aminotransferase	55.96	56.05	88.7	50		0.958
sp|Q16134|ETFD_HUMAN	ETFDH	Electron transfer flavoprotein-ubiquinone oxidoreductase, mitochondrial	20.51	20.73	42.3	11		0.959
sp|O94830|DDHD2_HUMAN	DDHD2	Phospholipase DDHD2	8.21	8.34	21.5	5		0.959
sp|Q96K17|BT3L4_HUMAN	BTF3L4	Transcription factor BTF3 homolog 4	12.2	12.6	55.1	10		0.959
sp|Q9Y3E0|GOT1B_HUMAN	GOLT1B	Vesicle transport protein GOT1B	4.48	4.54	23.2	3		0.959
sp|P60033|CD81_HUMAN	CD81	CD81 antigen	6.48	6.52	31.4	13		0.959
sp|A5PLN9|TPC13_HUMAN	TRAPPC13	Trafficking protein particle complex subunit 13	1.77	2.2	24.9	3		0.959

sp|Q7L2H7|EIF3M_HUMAN	EIF3M	Eukaryotic translation initiation factor 3 subunit M	27.42	27.57	58.8	29		0.959
sp|Q15008|PSMD6_HUMAN	PSMD6	26S proteasome non-ATPase regulatory subunit 6	47.42	47.52	72.2	34		0.959
sp|Q15006|EMC2_HUMAN	EMC2	ER membrane protein complex subunit 2	14.04	14.04	45.1	7		0.959
sp|Q9Y6G9|DC1L1_HUMAN	DYNC1LI1	Cytoplasmic dynein 1 light intermediate chain 1	41.16	41.25	69.4	27		0.959
sp|O76095|JTB_HUMAN	JTB	Protein JTB	2.09	2.11	22.6	2		0.960
sp|Q14202|ZMYM3_HUMAN	ZMYM3	Zinc finger MYM-type protein 3	38.51	38.79	32.4	23		0.960
sp|P54619|AAKG1_HUMAN	PRKAG1	5'-AMP-activated protein kinase subunit gamma-1	17.27	17.39	37.8	10		0.960
sp|P56182|RRP1_HUMAN	RRP1	Ribosomal RNA processing protein 1 homolog A	27.42	27.62	48.2	19		0.960
sp|P28799|GRN_HUMAN	GRN	Granulins	11.36	11.46	30	6		0.960
sp|P56556|NDUA6_HUMAN	NDUFA6	NADH dehydrogenase [ubiquinone] 1 alpha subcomplex subunit 6	6.03	7.35	46.1	8		0.960
sp|O96033|MOC2A_HUMAN	MOCS2	Molybdopterin synthase sulfur carrier subunit	2	2.02	31.8	2		0.960
sp|P03891|NU2M_HUMAN	MT-ND2	NADH-ubiquinone oxidoreductase chain 2	3.12	3.25	14.4	4		0.960
sp|O75431|MTX2_HUMAN	MTX2	Metaxin-2	18.11	18.7	56.7	14		0.960
sp|Q96GJ1|TRM2_HUMAN	TRMT2B	tRNA (uracil(54)-C(5))-methyltransferase homolog	4.07	4.2	18.7	3		0.960
sp|Q86TV6|TTC7B_HUMAN	TTC7B	Tetratricopeptide repeat protein 7B	6.16	6.35	22.4	4		0.961
sp|Q96IX5|USMG5_HUMAN	USMG5	Up-regulated during skeletal muscle growth protein 5	6.7	6.85	62.1	6		0.961
sp|Q86UY6|NAA40_HUMAN	NAA40	N-alpha-acetyltransferase 40	6	6	22.8	4		0.961
sp|Q6DKK2|TTC19_HUMAN	TTC19	Tetratricopeptide repeat protein 19, mitochondrial	13.11	14.79	51.6	9		0.961
sp|Q14241|ELOA1_HUMAN	TCEB3	Transcription elongation factor B polypeptide 3	15.6	15.83	33.6	11		0.961
sp|Q16560|U1SBP_HUMAN	SNRNP35	U11/U12 small nuclear ribonucleoprotein 35 kDa protein	2	2.05	25.6	2		0.961
sp|Q9NXN4|GDAP2_HUMAN	GDAP2	Ganglioside-induced differentiation-associated protein 2	2.6	3.53	16.1	3		0.961
sp|Q6NZ67|MZT2B_HUMAN	MZT2B	Mitotic-spindle organizing protein 2B	2	5.29	49.4	3		0.961
sp|O00483|NDUA4_HUMAN	NDUFA4	Cytochrome c oxidase subunit NDUFA4	7.68	7.87	61.7	8		0.961
sp|Q14149|MORC3_HUMAN	MORC3	MORC family CW-type zinc finger protein 3	8.84	10.98	26	6		0.961
sp|Q15361|TTF1_HUMAN	TTF1	Transcription termination factor 1	4.13	4.45	17	2		0.961
sp|Q9BZQ6|EDEM3_HUMAN	EDEM3	ER degradation-enhancing alpha-mannosidase-like protein 3	11.46	11.74	19.6	10		0.961
sp|Q9HAU0|PKHA5_HUMAN	PLEKHA5	Pleckstrin homology domain-containing family A member 5	16.7	16.96	24	9		0.961
sp|O15155|BET1_HUMAN	BET1	BET1 homolog	4	4.05	39	3		0.961
sp|Q8WVM7|STAG1_HUMAN	STAG1	Cohesin subunit SA-1	17.15	32.1	36.6	21		0.961
sp|Q96S38|KS6C1_HUMAN	RPS6KC1	Ribosomal protein S6 kinase delta-1	5.32	5.91	18.3	4		0.961
sp|Q9UNH6|SNX7_HUMAN	SNX7	Sorting nexin-7	5.63	5.73	26.1	3		0.961
sp|Q9Y580|RBM7_HUMAN	RBM7	RNA-binding protein 7	5.31	5.43	28.2	4		0.961
sp|P50583|AP4A_HUMAN	NUDT2	Bis(5'-nucleosyl)-tetraphosphatase [asymmetrical]	12.77	12.89	72.8	8		0.961
sp|Q16512|PKN1_HUMAN	PKN1	Serine/threonine-protein kinase N1	17.42	22.12	32.4	16		0.962
sp|Q9Y6A4|CFA20_HUMAN	CFAP20	Cilia- and flagella-associated protein 20	10.05	10.08	56.5	6		0.962
sp|P18615|NELFE_HUMAN	NELFE	Negative elongation factor E	21.92	22.14	51.1	14		0.962
sp|Q14683|SMC1A_HUMAN	SMC1A	Structural maintenance of chromosomes protein 1A	119.82	123.46	64.4	71		0.962
sp|Q6P1K8|T2H2L_HUMAN	GTF2H2C	General transcription factor IIH subunit 2-like protein	10.29	12.43	33.2	8		0.962
sp|P50851|LRBA_HUMAN	LRBA	Lipopolysaccharide-responsive and beige-like anchor protein	60.31	61.68	26.1	31		0.962
sp|Q9ULA0|DNPEP_HUMAN	DNPEP	Aspartyl aminopeptidase	21.44	22.4	52.2	16		0.962
sp|Q13705|AVR2B_HUMAN	ACVR2B	Activin receptor type-2B	2	2.02	13.1	2		0.962
sp|P82675|RT05_HUMAN	MRPS5	28S ribosomal protein S5, mitochondrial	27.07	29.81	54	16		0.962
sp|A6NK58|LIPT2_HUMAN	LIPT2	Putative lipoyltransferase 2, mitochondrial	2.03	2.05	25.1	2		0.962
sp|Q330K2|NDUF6_HUMAN	NDUFAF6	NADH dehydrogenase (ubiquinone) complex I, assembly factor 6	9.05	9.13	30.9	6		0.962
sp|Q96BY7|ATG2B_HUMAN	ATG2B	Autophagy-related protein 2 homolog B	4.04	4.47	12.3	3		0.962
sp|Q99538|LGMN_HUMAN	LGMN	Legumain	10.98	11.03	21.3	7		0.962
sp|P35625|TIMP3_HUMAN	TIMP3	Metalloproteinase inhibitor 3	2.4	2.44	17.1	3		0.962
sp|Q9BYB4|GNB1L_HUMAN	GNB1L	Guanine nucleotide-binding protein subunit beta-like protein 1	11.5	11.58	37.6	8		0.962
sp|Q9HA64|KT3K_HUMAN	FN3KRP	Ketosamine-3-kinase	5.77	5.92	29.5	6		0.962

sp|O14744|ANM5_HUMAN	PRMT5	Protein arginine N-methyltransferase 5	65.24	65.29	66.3	45	0.963
sp|O95478|NSA2_HUMAN	NSA2	Ribosome biogenesis protein NSA2 homolog	20.33	20.77	62.3	13	0.963
sp|O43924|PDE6D_HUMAN	PDE6D	Retinal rod rhodopsin-sensitive cGMP 3',5'-cyclic phosphodiesterase subunit delta	6	6.04	50.7	3	0.963
sp|Q9UER7|DAXX_HUMAN	DAXX	Death domain-associated protein 6	10.73	10.94	16.9	6	0.963
sp|P17174|AATC_HUMAN	GOT1	Aspartate aminotransferase, cytoplasmic	50.41	51.48	84.3	45	0.963
sp|Q15554|TERF2_HUMAN	TERF2	Telomeric repeat-binding factor 2	12.73	12.99	31.2	8	0.963
sp|Q9H9J2|RM44_HUMAN	MRPL44	39S ribosomal protein L44, mitochondrial	23.88	24	56.3	16	0.963
sp|Q96S19|CP013_HUMAN	C16orf13	UPF0585 protein C16orf13	4.74	5.17	32.8	4	0.963
sp|O76075|DFFB_HUMAN	DFFB	DNA fragmentation factor subunit beta	2.36	2.4	21.9	2	0.963
sp|P13667|PDIA4_HUMAN	PDIA4	Protein disulfide-isomerase A4	102.69	110.36	77.8	121	0.963
sp|Q16775|GLO2_HUMAN	HAGH	Hydroxyacylglutathione hydrolase, mitochondrial	11.84	12.13	54.9	9	0.963
sp|Q9UHD2|TBK1_HUMAN	TBK1	Serine/threonine-protein kinase TBK1	14.62	15.1	28.1	14	0.963
sp|Q92508|PIEZ1_HUMAN	PIEZO1	Piezo-type mechanosensitive ion channel component 1	2.45	2.52	10.5	4	0.963
sp|Q9BS26|ERP44_HUMAN	ERP44	Endoplasmic reticulum resident protein 44	27.09	30.64	74.9	29	0.964
sp|Q14667|K0100_HUMAN	KIAA0100	Protein KIAA0100	2.04	3.58	13.6	6	0.964
sp|O14874|BCKD_HUMAN	BCKDK	[3-methyl-2-oxobutanoate dehydrogenase [lipoamide]] kinase, mitochondrial	16.01	16.93	44.7	11	0.964
sp|P51692|STA5B_HUMAN	STAT5B	Signal transducer and activator of transcription 5B	8.36	9.45	24.7	7	0.964
sp|Q9H7Z6|KAT8_HUMAN	KAT8	Histone acetyltransferase KAT8	5.23	9.81	29.3	6	0.964
sp|Q9NVH6|TMLH_HUMAN	TMLHE	Trimethyllysine dioxygenase, mitochondrial	4.98	5.51	31.4	4	0.964
sp|P30414|NKTR_HUMAN	NKTR	NK-tumor recognition protein	7.47	7.68	14.2	6	0.964
sp|P49116|NR2C2_HUMAN	NR2C2	Nuclear receptor subfamily 2 group C member 2	6.9	7.14	23.8	6	0.964
sp|Q96NB2|SFXN2_HUMAN	SFXN2	Sideroflexin-2	6.27	6.45	37	5	0.964
sp|Q9BTE6|AASD1_HUMAN	AARSD1	Alanyl-tRNA editing protein Aarsd1	8.81	9.09	43.7	11	0.964
sp|O94766|B3GA3_HUMAN	B3GAT3	Galactosylgalactosylxylosylprotein 3-beta-glucuronosyltransferase 3	9.43	9.91	36.1	6	0.964
sp|P62487|RPB7_HUMAN	POLR2G	DNA-directed RNA polymerase II subunit RPB7	11.15	11.27	75	7	0.964
sp|Q9NW08|RPC2_HUMAN	POLR3B	DNA-directed RNA polymerase III subunit RPC2	34.88	35.81	40.7	19	0.964
sp|A0AVF1|IFT56_HUMAN	TTC26	Intraflagellar transport protein 56	2.18	2.57	23.7	3	0.964
sp|Q8WVJ2|NUDC2_HUMAN	NUDCD2	NudC domain-containing protein 2	6.23	6.35	59.2	3	0.964
sp|P51665|PSMD7_HUMAN	PSMD7	26S proteasome non-ATPase regulatory subunit 7	22.89	25.13	68.8	19	0.964
sp|Q9BSU1|CP070_HUMAN	C16orf70	UPF0183 protein C16orf70	6.52	6.58	20.9	5	0.964
sp|Q9BZL4|PP12C_HUMAN	PPP1R12C	Protein phosphatase 1 regulatory subunit 12C	2.38	2.74	17.9	3	0.964
sp|Q9UQN3|CHM2B_HUMAN	CHMP2B	Charged multivesicular body protein 2b	11.44	11.68	41.3	8	0.964
sp|Q96N11|CG026_HUMAN	C7orf26	Uncharacterized protein C7orf26	2.06	2.16	19.4	2	0.964
sp|O75312|ZPR1_HUMAN	ZPR1	Zinc finger protein ZPR1	12.03	14.93	33.8	11	0.964
sp|O75351|VPS4B_HUMAN	VPS4B	Vacuolar protein sorting-associated protein 4B	11.96	18.42	40.5	11	0.964
sp|P29084|T2EB_HUMAN	GTF2E2	Transcription initiation factor IIE subunit beta	14.76	16.46	63.9	10	0.964
sp|Q13561|DCTN2_HUMAN	DCTN2	Dynactin subunit 2	36.46	37.45	72.3	24	0.965
sp|Q8TAA9|VANG1_HUMAN	VANGL1	Vang-like protein 1	13.37	13.49	24.2	7	0.965
sp|Q9UH03|SEPT3_HUMAN	SEPT3	Neuronal-specific septin-3	2.65	3.1	30.5	3	0.965
sp|Q9NWV4|CA123_HUMAN	C1orf123	UPF0587 protein C1orf123	11.6	13.67	65	7	0.965
sp|P25205|MCM3_HUMAN	MCM3	DNA replication licensing factor MCM3	71.42	74.98	63.2	53	0.965
sp|P33316|DUT_HUMAN	DUT	Deoxyuridine 5'-triphosphate nucleotidohydrolase, mitochondrial	19.94	20.02	54	19	0.965
sp|P30566|PUR8_HUMAN	ADSL	Adenylosuccinate lyase	36.23	38.45	68.8	30	0.965
sp|Q9NUQ6|SPS2L_HUMAN	SPATS2L	SPATS2-like protein	32.59	32.79	55.2	21	0.965
sp|Q9BVG4|PBDC1_HUMAN	PBDC1	Protein PBDC1	18.6	18.72	78.5	11	0.965
sp|Q8WXW3|PIBF1_HUMAN	PIBF1	Progesterone-induced-blocking factor 1	2.1	4.18	31.4	4	0.965
sp|Q15257|PTPA_HUMAN	PPP2R4	Serine/threonine-protein phosphatase 2A activator	18.73	18.79	60.3	13	0.965
sp|O14907|TX1B3_HUMAN	TAX1BP3	Tax1-binding protein 3	4.09	4.11	57.3	6	0.965
sp|P48059|LIMS1_HUMAN	LIMS1	LIM and senescent cell antigen-like-containing domain protein 1	10.51	10.89	51.7	9	0.965

sp|Q9H2P0|ADNP_HUMAN	ADNP	Activity-dependent neuroprotector homeobox protein	41.34	41.46	39	25	0.965
sp|Q5PSV4|BRM1L_HUMAN	BRMS1L	Breast cancer metastasis-suppressor 1-like protein	9.52	9.59	35	6	0.965
sp|P04075|ALDOA_HUMAN	ALDOA	Fructose-bisphosphate aldolase A	78.33	78.36	97.3	159	0.965
sp|Q9P2E9|RRBP1_HUMAN	RRBP1	Ribosome-binding protein 1	135.34	138.36	78.8	115	0.965
sp|P84095|RHOG_HUMAN	RHOG	Rho-related GTP-binding protein RhoG	16.08	18.23	69.1	9	0.966
sp|Q8NBJ4|GOLM1_HUMAN	GOLM1	Golgi membrane protein 1	4.71	4.95	23.2	3	0.966
sp|Q9Y5V3|MAGD1_HUMAN	MAGED1	Melanoma-associated antigen D1	12.33	14.41	21.3	10	0.966
sp|Q01581|HMCS1_HUMAN	HMGCS1	Hydroxymethylglutaryl-CoA synthase, cytoplasmic	48.51	48.57	71.5	40	0.966
sp|Q16875|F263_HUMAN	PFKFB3	6-phosphofructo-2-kinase/fructose-2,6-bisphosphatase 3	1.83	8.65	30.8	5	0.966
sp|Q96RT1|LAP2_HUMAN	ERBB2IP	Protein LAP2	18.89	19.43	23.7	10	0.966
sp|Q96BR5|COA7_HUMAN	COA7	Cytochrome c oxidase assembly factor 7	21.67	23.95	86.2	14	0.966
sp|Q9NUD5|ZCHC3_HUMAN	ZCCHC3	Zinc finger CCHC domain-containing protein 3	23.04	23.17	55.7	15	0.966
sp|O95696|BRD1_HUMAN	BRD1	Bromodomain-containing protein 1	9.72	12.58	25.7	8	0.966
sp|P49773|HINT1_HUMAN	HINT1	Histidine triad nucleotide-binding protein 1	15.93	18.53	94.4	24	0.966
sp|Q9Y689|ARL5A_HUMAN	ARL5A	ADP-ribosylation factor-like protein 5A	2	5.99	31.3	4	0.966
sp|Q8NBJ5|GT251_HUMAN	COLGALT1	Procollagen galactosyltransferase 1	50.6	51.1	44.4	30	0.966
sp|P67809|YBOX1_HUMAN	YBX1	Nuclease-sensitive element-binding protein 1	28.09	28.43	68.2	47	0.966
sp|P24928|RPB1_HUMAN	POLR2A	DNA-directed RNA polymerase II subunit RPB1	104.2	105.08	47.1	57	0.966
sp|P42285|SK2L2_HUMAN	SKIV2L2	Superkiller viralicidic activity 2-like 2	82.97	85.17	58.2	48	0.966
sp|Q13608|PEX6_HUMAN	PEX6	Peroxisome assembly factor 2	2.01	4.27	14.5	2	0.967
sp|Q86VX2|COMD7_HUMAN	COMMD7	COMM domain-containing protein 7	4.04	4.1	25	3	0.967
sp|P12004|PCNA_HUMAN	PCNA	Proliferating cell nuclear antigen	32.64	32.77	77.4	79	0.967
sp|P16885|PLCG2_HUMAN	PLCG2	1-phosphatidylinositol 4,5-bisphosphate phosphodiesterase gamma-2	2.15	5.57	13	3	0.967
sp|Q9BRR6|ADPGK_HUMAN	ADPGK	ADP-dependent glucokinase	15.83	15.91	45.3	10	0.967
sp|O14920|IKKB_HUMAN	IKBKB	Inhibitor of nuclear factor kappa-B kinase subunit beta	8.61	10.52	23.8	6	0.967
sp|Q9H2J4|PDCL3_HUMAN	PDCL3	Phosducin-like protein 3	15.69	16.39	61.1	10	0.967
sp|A2RRP1|NBAS_HUMAN	NBAS	Neuroblastoma-amplified sequence	46.64	49.74	29.5	31	0.967
sp|P49247|RPIA_HUMAN	RPIA	Ribose-5-phosphate isomerase	23.02	23.14	63.7	21	0.967
sp|Q14C86|GAPD1_HUMAN	GAPVD1	GTPase-activating protein and VPS9 domain-containing protein 1	49.91	53.88	34	30	0.967
sp|Q8NC60|NOA1_HUMAN	NOA1	Nitric oxide-associated protein 1	34.88	34.95	52.9	19	0.967
sp|Q9NP79|VTA1_HUMAN	VTA1	Vacuolar protein sorting-associated protein VTA1 homolog	23.24	23.38	48.2	15	0.967
sp|P54886|P5CS_HUMAN	ALDH18A1	Delta-1-pyrroline-5-carboxylate synthase	83.93	84.05	71.8	94	0.967
sp|Q8NFQ8|TOIP2_HUMAN	TOR1AIP2	Torsin-1A-interacting protein 2	8.5	14.58	50	9	0.967
sp|P46019|KPB2_HUMAN	PHKA2	Phosphorylase b kinase regulatory subunit alpha, liver isoform	8.03	10.15	15.4	5	0.967
sp|Q02338|BDH_HUMAN	BDH1	D-beta-hydroxybutyrate dehydrogenase, mitochondrial	2.37	2.41	32.4	2	0.967
sp|Q9NVC6|MED17_HUMAN	MED17	Mediator of RNA polymerase II transcription subunit 17	12.92	13.19	33.6	8	0.967
sp|Q99417|MYCBP_HUMAN	MYCBP	C-Myc-binding protein	11.68	11.93	71.8	8	0.967
sp|P52594|AGFG1_HUMAN	AGFG1	Arf-GAP domain and FG repeat-containing protein 1	15.86	17.15	24.2	13	0.967
sp|Q9UPU7|TBD2B_HUMAN	TBC1D2B	TBC1 domain family member 2B	3.67	6.7	17.3	6	0.967
sp|Q9BXW9|FACD2_HUMAN	FANCD2	Fanconi anemia group D2 protein	26.96	28.79	29.2	16	0.967
sp|Q8IXQ5|KLHL7_HUMAN	KLHL7	Kelch-like protein 7	5.17	5.26	17.2	4	0.967
sp|Q12923|PTN13_HUMAN	PTPN13	Tyrosine-protein phosphatase non-receptor type 13	16.72	17.7	16.1	9	0.968
sp|P31151|S10A7_HUMAN	S100A7	Protein S100-A7	3.7	3.77	26.7	2	0.968
sp|Q9GZQ8|MLP3B_HUMAN	MAP1LC3B	Microtubule-associated proteins 1A/1B light chain 3B	3.38	3.6	22.4	2	0.968
sp|Q9UIL1|SCOC_HUMAN	SCOC	Short coiled-coil protein	4.01	4.06	40.9	4	0.968
sp|P25685|DNJB1_HUMAN	DNAJB1	DnaJ homolog subfamily B member 1	28.58	30.66	62.4	20	0.968
sp|Q9NZ32|ARP10_HUMAN	ACTR10	Actin-related protein 10	9.84	9.95	22.3	6	0.968
sp|O43524|FOXO3_HUMAN	FOXO3	Forkhead box protein O3	4.01	4.01	10	2	0.968
sp|Q9C0B1|FTO_HUMAN	FTO	Alpha-ketoglutarate-dependent dioxygenase FTO	36.28	36.29	68.9	22	0.968

sp|Q9H871|RMD5A_HUMAN	RMND5A	Protein RMD5 homolog A	5.89	6.03	26.3	3	0.968
sp|Q08378|GOGA3_HUMAN	GOLGA3	Golgin subfamily A member 3	53.91	56.29	43.5	31	0.968
sp|Q8N3Z3|GTPB8_HUMAN	GTPBP8	GTP-binding protein 8	8.85	8.99	38.7	5	0.968
sp|Q14157|UBP2L_HUMAN	UBAP2L	Ubiquitin-associated protein 2-like	39.48	39.76	44.4	33	0.968
sp|Q8TF76|HASP_HUMAN	GSG2	Serine/threonine-protein kinase haspin	6.82	6.91	16.3	4	0.968
sp|Q96GX5|GWL_HUMAN	MASTL	Serine/threonine-protein kinase greatwall	4.06	6.11	13	3	0.968
sp|O00244|ATOX1_HUMAN	ATOX1	Copper transport protein ATOX1	11.84	11.91	94.1	9	0.969
sp|Q92990|GLMN_HUMAN	GLMN	Glomulin	22.09	23.68	42.4	18	0.969
sp|Q8NFV4|ABHDB_HUMAN	ABHD11	Alpha/beta hydrolase domain-containing protein 11	12.78	12.92	40.3	9	0.969
sp|Q9Y5Z4|HEBP2_HUMAN	HEBP2	Heme-binding protein 2	17.16	17.2	61	13	0.969
sp|Q14493|SLBP_HUMAN	SLBP	Histone RNA hairpin-binding protein	2.63	2.67	21.9	2	0.969
sp|Q9BVT8|TMUB1_HUMAN	TMUB1	Transmembrane and ubiquitin-like domain-containing protein 1	6	6	21.1	3	0.969
sp|Q9HBI6|CP4FB_HUMAN	CYP4F11	Phylloquinone omega-hydroxylase CYP4F11	4.06	4.09	21.6	2	0.969
sp|Q8N0X4|CLYBL_HUMAN	CLYBL	Citrate lyase subunit beta-like protein, mitochondrial	16.76	19.77	51.2	13	0.969
sp|Q14684|RRP1B_HUMAN	RRP1B	Ribosomal RNA processing protein 1 homolog B	60.8	61.16	58.7	42	0.970
sp|Q8N5M9|JAGN1_HUMAN	JAGN1	Protein jagunal homolog 1	3.64	3.76	20.2	3	0.970
sp|P50570|DYN2_HUMAN	DNM2	Dynamin-2	67.64	69.9	59.8	46	0.970
sp|P51151|RAB9A_HUMAN	RAB9A	Ras-related protein Rab-9A	14.56	16.41	62.7	11	0.970
sp|Q68CQ4|DIEXF_HUMAN	DIEXF	Digestive organ expansion factor homolog	19.91	21.36	34.1	10	0.970
sp|P16383|GCFC2_HUMAN	GCFC2	GC-rich sequence DNA-binding factor 2	15.72	16.07	37.1	11	0.970
sp|P13498|CY24A_HUMAN	CYBA	Cytochrome b-245 light chain	1.71	1.88	12.8	3	0.970
sp|Q9ULR0|ISY1_HUMAN	ISY1	Pre-mRNA-splicing factor ISY1 homolog	11.69	13.55	48.4	10	0.970
sp|O14686|KMT2D_HUMAN	KMT2D	Histone-lysine N-methyltransferase 2D	12.94	13.62	10.5	9	0.970
sp|P13984|T2FB_HUMAN	GTF2F2	General transcription factor IIF subunit 2	13.99	14.43	68.3	11	0.970
sp|P30041|PRDX6_HUMAN	PRDX6	Peroxiredoxin-6	44.05	45.7	88	54	0.970
sp|Q14444|CAPR1_HUMAN	CAPRIN1	Caprin-1	27.33	27.49	41.6	34	0.970
sp|Q9NY33|DPP3_HUMAN	DPP3	Dipeptidyl peptidase 3	51.13	54.12	59.6	38	0.970
sp|O95071|UBR5_HUMAN	UBR5	E3 ubiquitin-protein ligase UBR5	59.9	60.25	29.4	34	0.970
sp|P83881|RL36A_HUMAN	RPL36A	60S ribosomal protein L36a	10.62	11.95	65.1	12	0.970
sp|Q5TAQ9|DCAF8_HUMAN	DCAF8	DDB1- and CUL4-associated factor 8	9.56	9.69	19.6	6	0.970
sp|O95166|GBRAP_HUMAN	GABARAP	Gamma-aminobutyric acid receptor-associated protein	4.01	6.58	51.3	5	0.970
sp|Q8NB90|SPAT5_HUMAN	SPATA5	Spermatogenesis-associated protein 5	18.45	21.43	32.6	12	0.970
sp|Q9Y333|LSM2_HUMAN	LSM2	U6 snRNA-associated Sm-like protein LSm2	12.33	13.19	99	10	0.970
sp|P15104|GLNA_HUMAN	GLUL	Glutamine synthetase	18.62	18.65	41.6	16	0.970
sp|O60518|RNBP6_HUMAN	RANBP6	Ran-binding protein 6	20.22	26.26	32.3	15	0.970
sp|Q9UKG1|DP13A_HUMAN	APPL1	DCC-interacting protein 13-alpha	26.34	27.66	43.6	15	0.970
sp|P08195|4F2_HUMAN	SLC3A2	4F2 cell-surface antigen heavy chain	57.07	58.46	54.1	44	0.970
sp|P30043|BLVRB_HUMAN	BLVRB	Flavin reductase (NADPH)	16.9	16.99	59.7	11	0.970
sp|Q86U90|YRDC_HUMAN	YRDC	YrdC domain-containing protein, mitochondrial	11.83	11.93	52.7	6	0.971
sp|Q9H5K3|SG196_HUMAN	POMK	Protein O-mannose kinase	3.95	5.07	23.4	3	0.971
sp|Q9BXI6|TB10A_HUMAN	TBC1D10A	TBC1 domain family member 10A	5.41	5.59	28.7	4	0.971
sp|Q8IYB8|SUV3_HUMAN	SUPV3L1	ATP-dependent RNA helicase SUPV3L1, mitochondrial	31.68	33.16	34.7	23	0.971
sp|O14966|RAB7L_HUMAN	RAB29	Ras-related protein Rab-7L1	8.03	10.22	46.3	5	0.971
sp|Q9UGH3|S23A2_HUMAN	SLC23A2	Solute carrier family 23 member 2	4.09	4.1	11.9	2	0.971
sp|P30613|KPYR_HUMAN	PKLR	Pyruvate kinase PKLR	25.58	32.46	52.8	23	0.971
sp|Q15388|TOM20_HUMAN	TOMM20	Mitochondrial import receptor subunit TOM20 homolog	8	10.12	44.1	7	0.971
sp|Q8IZT6|ASPM_HUMAN	ASPM	Abnormal spindle-like microcephaly-associated protein	4.05	7.19	19.2	7	0.971
sp|Q5TGZ0|MIC10_HUMAN	MINOS1	MIC complex subunit MIC10	4	4	51.3	2	0.972
sp|Q16576|RBBP7_HUMAN	RBBP7	Histone-binding protein RBBP7	29.42	29.56	64	27	0.972

sp|O14964|HGS_HUMAN	HGS	Hepatocyte growth factor-regulated tyrosine kinase substrate	34.92	35.68	39.8	30	0.972
sp|Q6KCM7|SCMC2_HUMAN	SLC25A25	Calcium-binding mitochondrial carrier protein SCaMC-2	3.9	4.09	27.5	3	0.972
sp|A8MXV4|NUD19_HUMAN	NUDT19	Nucleoside diphosphate-linked moiety X motif 19, mitochondrial	14.8	15.69	51.7	10	0.972
sp|Q6YHU6|THADA_HUMAN	THADA	Thyroid adenoma-associated protein	15.84	16.84	21.4	10	0.972
sp|P17050|NAGAB_HUMAN	NAGA	Alpha-N-acetylgalactosaminidase	2.57	2.62	18.7	3	0.972
sp|Q96PU5|NED4L_HUMAN	NEDD4L	E3 ubiquitin-protein ligase NEDD4-like	19.13	22.52	23.3	13	0.973
sp|Q9P2X0|DPM3_HUMAN	DPM3	Dolichol-phosphate mannosyltransferase subunit 3	3.13	3.28	31.5	5	0.973
sp|O94874|UFL1_HUMAN	UFL1	E3 UFM1-protein ligase 1	66.41	67.99	60	43	0.973
sp|P49757|NUMB_HUMAN	NUMB	Protein numb homolog	12.73	13.03	24	9	0.973
sp|Q14166|TTL12_HUMAN	TTLL12	Tubulin--tyrosine ligase-like protein 12	56.06	56.57	68	49	0.973
sp|Q14204|DYHC1_HUMAN	DYNC1H1	Cytoplasmic dynein 1 heavy chain 1	433.36	433.36	67.6	290	0.973
sp|Q99615|DNJC7_HUMAN	DNAJC7	DnaJ homolog subfamily C member 7	50.91	51.15	65	35	0.973
sp|Q86YQ8|CPNE8_HUMAN	CPNE8	Copine-8	8.67	11.48	28.6	8	0.973
sp|Q8WWH5|TRUB1_HUMAN	TRUB1	Probable tRNA pseudouridine synthase 1	17.3	17.65	56.2	12	0.973
sp|Q92900|RENT1_HUMAN	UPF1	Regulator of nonsense transcripts 1	92.08	94.16	62.5	61	0.973
sp|Q9H3P2|NELFA_HUMAN	NELFA	Negative elongation factor A	16.15	16.24	46.2	9	0.973
sp|P49959|MRE11_HUMAN	MRE11A	Double-strand break repair protein MRE11A	39.73	39.93	46.9	27	0.973
sp|P52298|NCBP2_HUMAN	NCBP2	Nuclear cap-binding protein subunit 2	8.13	8.38	62.8	7	0.973
sp|P33991|MCM4_HUMAN	MCM4	DNA replication licensing factor MCM4	69.7	71.43	65.4	58	0.973
sp|Q9NR56|MBNL1_HUMAN	MBNL1	Muscleblind-like protein 1	12.76	12.81	25.5	7	0.973
sp|Q9NWU5|RM22_HUMAN	MRPL22	39S ribosomal protein L22, mitochondrial	13.8	13.92	61.2	12	0.973
sp|Q8N335|GPD1L_HUMAN	GPD1L	Glycerol-3-phosphate dehydrogenase 1-like protein	31.49	31.63	63.8	18	0.973
sp|Q96F86|EDC3_HUMAN	EDC3	Enhancer of mRNA-decapping protein 3	25.7	25.9	50.6	13	0.973
sp|A1X283|SPD2B_HUMAN	SH3PXD2B	SH3 and PX domain-containing protein 2B	13.42	13.98	30.1	10	0.973
sp|P09874|PARP1_HUMAN	PARP1	Poly [ADP-ribose] polymerase 1	155.62	158.35	80.6	150	0.973
sp|Q02127|PYRD_HUMAN	DHODH	Dihydroorotate dehydrogenase (quinone), mitochondrial	24.49	24.68	62.8	12	0.973
sp|Q6PD74|AAGAB_HUMAN	AAGAB	Alpha- and gamma-adaptin-binding protein p34	10.01	10.14	46.4	7	0.973
sp|Q9NQ66|PLCB1_HUMAN	PLCB1	1-phosphatidylinositol 4,5-bisphosphate phosphodiesterase beta-1	13.7	14.26	24.3	9	0.973
sp|P50747|BPL1_HUMAN	HLCS	Biotin--protein ligase	9.59	9.78	18.2	5	0.973
sp|Q15031|SYLM_HUMAN	LARS2	Probable leucine--tRNA ligase, mitochondrial	55.12	56.67	55.3	31	0.973
sp|Q96GC9|VMP1_HUMAN	VMP1	Vacuole membrane protein 1	8	8.05	28.6	7	0.973
sp|P62917|RL8_HUMAN	RPL8	60S ribosomal protein L8	36.99	37.23	69.3	39	0.974
sp|P47914|RL29_HUMAN	RPL29	60S ribosomal protein L29	6.32	8.52	39.6	10	0.974
sp|Q96ES7|SGF29_HUMAN	CCDC101	SAGA-associated factor 29 homolog	3.75	4.48	19.5	5	0.974
sp|O76003|GLRX3_HUMAN	GLRX3	Glutaredoxin-3	38.02	38.15	71.9	42	0.974
sp|Q8TCG1|CIP2A_HUMAN	KIAA1524	Protein CIP2A	34.59	42.34	46.1	25	0.974
sp|Q15013|MD2BP_HUMAN	MAD2L1BP	MAD2L1-binding protein	4.05	5.75	36.1	4	0.974
sp|P35249|RFC4_HUMAN	RFC4	Replication factor C subunit 4	34.9	36.28	81	21	0.974
sp|Q8IUD2|RB6I2_HUMAN	ERC1	ELKS/Rab6-interacting/CAST family member 1	29.89	33.8	35.8	22	0.974
sp|P46940|IQGA1_HUMAN	IQGAP1	Ras GTPase-activating-like protein IQGAP1	144.02	144.8	70.6	112	0.974
sp|Q6R327|RICTR_HUMAN	RICTOR	Rapamycin-insensitive companion of mTOR	3.24	3.48	15.2	5	0.974
sp|Q9P253|VPS18_HUMAN	VPS18	Vacuolar protein sorting-associated protein 18 homolog	15.57	15.97	28.6	9	0.974
sp|Q9Y6X3|SCC4_HUMAN	MAU2	MAU2 chromatid cohesion factor homolog	7.92	8.94	19.7	6	0.974
sp|Q7Z494|NPHP3_HUMAN	NPHP3	Nephrocystin-3	5.12	5.78	18.3	5	0.974
sp|Q16864|VATF_HUMAN	ATP6V1F	V-type proton ATPase subunit F	8.05	8.07	60.5	6	0.974
sp|Q92604|LGAT1_HUMAN	LPGAT1	Acyl-CoA:lysophosphatidylglycerol acyltransferase 1	8.13	8.21	31.6	4	0.974
sp|P82932|RT06_HUMAN	MRPS6	28S ribosomal protein S6, mitochondrial	12.33	13.11	79.2	10	0.975
sp|Q12972|PP1R8_HUMAN	PPP1R8	Nuclear inhibitor of protein phosphatase 1	21.2	21.28	55.8	13	0.975
sp|Q8NE62|CHDH_HUMAN	CHDH	Choline dehydrogenase, mitochondrial	25.44	25.7	48.3	16	0.975

sp|Q9BUK6|MSTO1_HUMAN	MSTO1	Protein misato homolog 1	12.84	12.94	24.6	7	0.975
sp|O60337|MARH6_HUMAN	6-Mar	E3 ubiquitin-protein ligase MARCH6	2.62	2.91	8.8	3	0.975
sp|Q9H2J7|S6A15_HUMAN	SLC6A15	Sodium-dependent neutral amino acid transporter B(0)AT2	11.52	11.71	20.6	6	0.975
sp|Q9UNZ2|NSF1C_HUMAN	NSFL1C	NSFL1 cofactor p47	23.87	23.94	58.7	24	0.975
sp|Q96T51|RUFY1_HUMAN	RUFY1	RUN and FYVE domain-containing protein 1	21.98	22.27	40.4	13	0.975
sp|O95167|NDUA3_HUMAN	NDUFA3	NADH dehydrogenase [ubiquinone] 1 alpha subcomplex subunit 3	6.01	6.13	57.1	3	0.975
sp|Q9Y2H6|FND3A_HUMAN	FNDC3A	Fibronectin type-III domain-containing protein 3A	38.73	39.12	39.6	19	0.975
sp|P06400|RB_HUMAN	RB1	Retinoblastoma-associated protein	9.43	9.87	36.5	10	0.975
sp|P31260|HXA10_HUMAN	HOXA10	Homeobox protein Hox-A10	2.56	4.72	18.1	3	0.975
sp|P55011|S12A2_HUMAN	SLC12A2	Solute carrier family 12 member 2	27.13	29.53	31.4	20	0.975
sp|P46781|RS9_HUMAN	RPS9	40S ribosomal protein S9	22.26	24.55	57.7	19	0.975
sp|Q9HAB8|PPCS_HUMAN	PPCS	Phosphopantothenate--cysteine ligase	15.53	16.33	50.5	11	0.975
sp|Q9Y2Y0|AR2BP_HUMAN	ARL2BP	ADP-ribosylation factor-like protein 2-binding protein	3.81	3.9	23.3	3	0.975
sp|Q7Z333|SETX_HUMAN	SETX	Probable helicase senataxin	2.15	4.29	11.9	5	0.976
sp|Q9BWU1|CDK19_HUMAN	CDK19	Cyclin-dependent kinase 19	3.92	4.27	17.3	3	0.976
sp|P62829|RL23_HUMAN	RPL23	60S ribosomal protein L23	36.51	36.54	88.6	52	0.976
sp|Q9H1P3|OSBL2_HUMAN	OSBPL2	Oxysterol-binding protein-related protein 2	14.18	14.34	33.5	9	0.976
sp|Q96QC0|PP1RA_HUMAN	PPP1R10	Serine/threonine-protein phosphatase 1 regulatory subunit 10	25.29	25.75	30	16	0.976
sp|P35520|CBS_HUMAN	CBS	Cystathionine beta-synthase	35.26	38.14	64.1	24	0.976
sp|Q9Y487|VPP2_HUMAN	ATP6V0A2	V-type proton ATPase 116 kDa subunit a isoform 2	23.7	24.1	32.9	12	0.976
sp|Q9UKF6|CPSF3_HUMAN	CPSF3	Cleavage and polyadenylation specificity factor subunit 3	41.71	41.82	54.2	26	0.976
sp|Q05519|SRS11_HUMAN	SRSF11	Serine/arginine-rich splicing factor 11	14.79	15.08	32.6	9	0.976
sp|Q04206|TF65_HUMAN	RELA	Transcription factor p65	20.08	21.01	36.5	12	0.976
sp|Q9GZP9|DERL2_HUMAN	DERL2	Derlin-2	4.35	4.38	22.2	4	0.976
sp|P07195|LDHB_HUMAN	LDHB	L-lactate dehydrogenase B chain	44.08	48.42	73.4	77	0.976
sp|Q01469|FABP5_HUMAN	FABP5	Fatty acid-binding protein, epidermal	27.74	28.39	93.3	27	0.976
sp|Q9NP72|RAB18_HUMAN	RAB18	Ras-related protein Rab-18	19.74	19.97	78.6	15	0.976
sp|Q02543|RL18A_HUMAN	RPL18A	60S ribosomal protein L18a	17.92	18.05	58	20	0.976
sp|Q96MC6|HIAT1_HUMAN	HIAT1	Hippocampus abundant transcript 1 protein	2	2.06	11.6	2	0.976
sp|Q5T2T1|MPP7_HUMAN	MPP7	MAGUK p55 subfamily member 7	1.37	1.52	22.9	2	0.977
sp|Q9BUL5|PHF23_HUMAN	PHF23	PHD finger protein 23	6.43	6.52	14.1	3	0.977
sp|O15305|PMM2_HUMAN	PMM2	Phosphomannomutase 2	22.67	23.78	69.5	17	0.977
sp|A7E2V4|ZSWM8_HUMAN	ZSWIM8	Zinc finger SWIM domain-containing protein 8	2.59	2.66	7.4	3	0.977
sp|Q14849|STAR3_HUMAN	STARD3	StAR-related lipid transfer protein 3	3.18	3.41	26.1	4	0.977
sp|Q9Y2W1|TR150_HUMAN	THRAP3	Thyroid hormone receptor-associated protein 3	36.75	40.42	39.7	25	0.977
sp|P41236|IPP2_HUMAN	PPP1R2	Protein phosphatase inhibitor 2	7.23	7.54	42.4	4	0.977
sp|Q9NRS6|SNX15_HUMAN	SNX15	Sorting nexin-15	1.43	1.68	25.7	3	0.978
sp|Q9Y2S6|TMA7_HUMAN	TMA7	Translation machinery-associated protein 7	4.28	4.56	46.9	3	0.978
sp|Q8IWB7|WDFY1_HUMAN	WDFY1	WD repeat and FYVE domain-containing protein 1	28.13	28.29	53.7	15	0.978
sp|Q8NCG7|DGLB_HUMAN	DAGLB	Sn1-specific diacylglycerol lipase beta	4.02	4.24	15.6	2	0.978
sp|Q99549|MPP8_HUMAN	MPHOSPH8	M-phase phosphoprotein 8	16.54	17.27	35.4	8	0.978
sp|Q9NRP4|SDHF3_HUMAN	SDHAF3	Succinate dehydrogenase assembly factor 3, mitochondrial	2.54	2.58	37.6	2	0.978
sp|Q9UBC1|IKBL1_HUMAN	NFKBIL1	NF-kappa-B inhibitor-like protein 1	1.5	1.78	14.4	2	0.978
sp|Q9Y6W5|WASF2_HUMAN	WASF2	Wiskott-Aldrich syndrome protein family member 2	7.26	9.44	20.9	9	0.979
sp|P49327|FAS_HUMAN	FASN	Fatty acid synthase	286.79	287.02	79.1	418	0.979
sp|P09012|SNRPA_HUMAN	SNRPA	U1 small nuclear ribonucleoprotein A	22.08	22.67	50	15	0.979
sp|O60220|TIM8A_HUMAN	TIMM8A	Mitochondrial import inner membrane translocase subunit Tim8 A	9.72	9.8	76.3	14	0.979
sp|P08240|SRPR_HUMAN	SRPR	Signal recognition particle receptor subunit alpha	43.95	45.17	64.4	27	0.979
sp|Q9GZT6|CC90B_HUMAN	CCDC90B	Coiled-coil domain-containing protein 90B, mitochondrial	5.83	5.97	38.2	3	0.979

sp|Q6P9B9|INT5_HUMAN	INTS5	Integrator complex subunit 5	12.46	14.69	19.2	8	0.979
sp|Q9C0B5|ZDHC5_HUMAN	ZDHHC5	Palmitoyltransferase ZDHHC5	4.33	4.47	15.9	5	0.979
sp|P15289|ARSA_HUMAN	ARSA	Arylsulfatase A	7.99	8.43	18.5	6	0.979
sp|P46379|BAG6_HUMAN	BAG6	Large proline-rich protein BAG6	53.58	53.6	45.1	32	0.979
sp|Q9NRR5|UBQL4_HUMAN	UBQLN4	Ubiquilin-4	31.8	31.88	46.8	29	0.979
sp|P35240|MERL_HUMAN	NF2	Merlin	16.2	19.04	35.1	10	0.979
sp|Q9Y4K3|TRAF6_HUMAN	TRAF6	TNF receptor-associated factor 6	7.3	7.8	19.7	5	0.979
sp|Q96GQ5|RUS1_HUMAN	C16orf58	RUS1 family protein C16orf58	11.82	12.08	34.4	7	0.979
sp|Q14186|TFDP1_HUMAN	TFDP1	Transcription factor Dp-1	8.74	8.85	26.6	6	0.979
sp|Q9H0H0|INT2_HUMAN	INTS2	Integrator complex subunit 2	13.48	13.82	22.8	7	0.979
sp|Q9NRZ9|HELLS_HUMAN	HELLS	Lymphoid-specific helicase	42.14	43.13	50.4	23	0.979
sp|O94992|HEXI1_HUMAN	HEXIM1	Protein HEXIM1	13.71	13.8	36.5	9	0.979
sp|Q9Y2D5|AKAP2_HUMAN	AKAP2	A-kinase anchor protein 2	11.53	11.82	25.8	7	0.979
sp|Q13263|TIF1B_HUMAN	TRIM28	Transcription intermediary factor 1-beta	86.68	86.98	79.5	126	0.979
sp|P00367|DHE3_HUMAN	GLUD1	Glutamate dehydrogenase 1, mitochondrial	72.48	75.05	77.6	81	0.980
sp|Q9H2C0|GAN_HUMAN	GAN	Gigaxonin	10.25	12.42	22.1	7	0.980
sp|Q00653|NFKB2_HUMAN	NFKB2	Nuclear factor NF-kappa-B p100 subunit	7.16	7.31	25.2	4	0.980
sp|P20338|RAB4A_HUMAN	RAB4A	Ras-related protein Rab-4A	15.16	17.58	75.7	13	0.980
sp|Q9UBQ0|VPS29_HUMAN	VPS29	Vacuolar protein sorting-associated protein 29	22.27	22.38	67.6	13	0.980
sp|O75607|NPM3_HUMAN	NPM3	Nucleoplasmin-3	12.03	12.08	69.7	9	0.980
sp|Q96Q11|TRNT1_HUMAN	TRNT1	CCA tRNA nucleotidyltransferase 1, mitochondrial	17.85	20.02	53.7	11	0.980
sp|P49721|PSB2_HUMAN	PSMB2	Proteasome subunit beta type-2	19.2	19.88	73.1	21	0.980
sp|O95292|VAPB_HUMAN	VAPB	Vesicle-associated membrane protein-associated protein B/C	13.14	16.51	50.6	18	0.980
sp|Q14004|CDK13_HUMAN	CDK13	Cyclin-dependent kinase 13	2.61	10.74	19.8	7	0.980
sp|Q32P28|P3H1_HUMAN	P3H1	Prolyl 3-hydroxylase 1	41.38	41.63	57.9	25	0.980
sp|Q9UHX1|PUF60_HUMAN	PUF60	Poly(U)-binding-splicing factor PUF60	52.22	52.32	65.1	45	0.980
sp|P61586|RHOA_HUMAN	RHOA	Transforming protein RhoA	36.12	36.63	75.1	44	0.980
sp|Q00403|TF2B_HUMAN	GTF2B	Transcription initiation factor IIB	19.5	19.61	53.8	11	0.981
sp|Q9Y2J2|E41L3_HUMAN	EPB41L3	Band 4.1-like protein 3	22.52	36.7	32.1	19	0.981
sp|Q9BQB6|VKOR1_HUMAN	VKORC1	Vitamin K epoxide reductase complex subunit 1	4	4	20.3	2	0.981
sp|P12694|ODBA_HUMAN	BCKDHA	2-oxoisovalerate dehydrogenase subunit alpha, mitochondrial	25.2	25.68	53	17	0.981
sp|Q9BQ95|ECSIT_HUMAN	ECSIT	Evolutionarily conserved signaling intermediate in Toll pathway, mitochondrial	15.86	15.92	41.3	10	0.981
sp|Q5GLZ8|HERC4_HUMAN	HERC4	Probable E3 ubiquitin-protein ligase HERC4	14.33	14.79	26	11	0.981
sp|Q7Z392|TPC11_HUMAN	TRAPPC11	Trafficking protein particle complex subunit 11	5.34	5.58	17.4	5	0.981
sp|P14868|SYDC_HUMAN	DARS	Aspartate--tRNA ligase, cytoplasmic	56.52	57.93	72.3	48	0.981
sp|Q96FV2|SCRN2_HUMAN	SCRN2	Secernin-2	6.08	6.1	21.7	3	0.981
sp|Q96SB8|SMC6_HUMAN	SMC6	Structural maintenance of chromosomes protein 6	17.37	18.57	35.8	15	0.981
sp|Q15436|SC23A_HUMAN	SEC23A	Protein transport protein Sec23A	64.46	64.42	68.4	47	0.981
sp|O43251|RFOX2_HUMAN	RBFOX2	RNA binding protein fox-1 homolog 2	6.13	6.77	26.4	4	0.981
sp|Q12933|TRAF2_HUMAN	TRAF2	TNF receptor-associated factor 2	11.33	11.55	37.1	6	0.981
sp|Q9P2R7|SUCB1_HUMAN	SUCLA2	Succinyl-CoA ligase [ADP-forming] subunit beta, mitochondrial	39.16	39.62	59.4	27	0.982
sp|P49720|PSB3_HUMAN	PSMB3	Proteasome subunit beta type-3	15.51	15.78	62.4	21	0.982
sp|P48735|IDHP_HUMAN	IDH2	Isocitrate dehydrogenase [NADP], mitochondrial	46.26	49.84	63.3	39	0.982
sp|Q9NP80|PLPL8_HUMAN	PNPLA8	Calcium-independent phospholipase A2-gamma	4.01	4.81	22.6	4	0.982
sp|Q9ULU4|PKCB1_HUMAN	ZMYND8	Protein kinase C-binding protein 1	36.81	39.62	33.2	21	0.982
sp|Q9BV94|EDEM2_HUMAN	EDEM2	ER degradation-enhancing alpha-mannosidase-like protein 2	4.07	4.57	17.3	4	0.982
sp|P04406|G3P_HUMAN	GAPDH	Glyceraldehyde-3-phosphate dehydrogenase	106.7	106.77	95.8	355	0.982
sp|Q6P2E9|EDC4_HUMAN	EDC4	Enhancer of mRNA-decapping protein 4	76.54	77.22	56.3	46	0.982
sp|Q8NHZ8|CDC26_HUMAN	CDC26	Anaphase-promoting complex subunit CDC26	3.01	3.09	60	2	0.982

sp|Q9NP81|SYSM_HUMAN	SARS2	Serine--tRNA ligase, mitochondrial	38.26	41.51	63.7	26	0.982
sp|Q8N5N7|RM50_HUMAN	MRPL50	39S ribosomal protein L50, mitochondrial	16.2	16.21	63.3	11	0.982
sp|O95782|AP2A1_HUMAN	AP2A1	AP-2 complex subunit alpha-1	71.24	72.13	57.7	56	0.982
sp|P34897|GLYM_HUMAN	SHMT2	Serine hydroxymethyltransferase, mitochondrial	71.41	73.82	83.9	92	0.982
sp|P52564|MP2K6_HUMAN	MAP2K6	Dual specificity mitogen-activated protein kinase kinase 6	8.2	12.32	40.1	7	0.982
sp|Q86US8|EST1A_HUMAN	SMG6	Telomerase-binding protein EST1A	8.75	8.96	16.4	5	0.982
sp|Q96CW1|AP2M1_HUMAN	AP2M1	AP-2 complex subunit mu	26.6	28.29	65.1	19	0.982
sp|O75569|PRKRA_HUMAN	PRKRA	Interferon-inducible double-stranded RNA-dependent protein kinase activator A	13.1	13.16	47.9	7	0.982
sp|O14495|LPP3_HUMAN	PPAP2B	Lipid phosphate phosphohydrolase 3	5.96	6.02	20.6	3	0.982
sp|Q9Y6K9|NEMO_HUMAN	IKBKG	NF-kappa-B essential modulator	16.3	16.78	43.2	9	0.982
sp|O60232|SSA27_HUMAN	SSSCA1	Sjoegren syndrome/scleroderma autoantigen 1	13.16	13.28	71.9	12	0.982
sp|Q69YN4|VIR_HUMAN	KIAA1429	Protein virilizer homolog	26.9	27.66	23.8	18	0.982
sp|Q8WUK0|PTPM1_HUMAN	PTPMT1	Phosphatidylglycerophosphatase and protein-tyrosine phosphatase 1	8.15	10.53	44.8	8	0.982
sp|Q7L7X3|TAOK1_HUMAN	TAOK1	Serine/threonine-protein kinase TAO1	15.91	16.34	25.9	12	0.982
sp|Q9UHK6|AMACR_HUMAN	AMACR	Alpha-methylacyl-CoA racemase	5.09	5.51	25.9	7	0.982
sp|Q96F44|TRI11_HUMAN	TRIM11	E3 ubiquitin-protein ligase TRIM11	4.95	5.05	14.3	4	0.983
sp|Q9NZC3|GDE1_HUMAN	GDE1	Glycerophosphodiester phosphodiesterase 1	4.01	4.93	22.1	3	0.983
sp|Q96E29|MTEF3_HUMAN	MTERF3	Transcription termination factor 3, mitochondrial	6.08	6.28	30.5	4	0.983
sp|Q9UNK0|STX8_HUMAN	STX8	Syntaxin-8	12.85	15.62	58.5	9	0.983
sp|Q9HAZ1|CLK4_HUMAN	CLK4	Dual specificity protein kinase CLK4	4	4.01	10.4	3	0.983
sp|P16401|H15_HUMAN	HIST1H1B	Histone H1.5	16.51	28.17	75.2	24	0.983
sp|Q99797|MIPEP_HUMAN	MIPEP	Mitochondrial intermediate peptidase	35.22	36.83	53	19	0.983
sp|O43175|SERA_HUMAN	PHGDH	D-3-phosphoglycerate dehydrogenase	52.23	52.81	70.9	82	0.984
sp|Q9UKK9|NUDT5_HUMAN	NUDT5	ADP-sugar pyrophosphatase	19.18	19.43	72.2	15	0.984
sp|P43897|EFTS_HUMAN	TSFM	Elongation factor Ts, mitochondrial	27.17	28.11	70.5	24	0.984
sp|Q9Y399|RT02_HUMAN	MRPS2	28S ribosomal protein S2, mitochondrial	14.06	14.72	57.8	11	0.984
sp|Q99638|RAD9A_HUMAN	RAD9A	Cell cycle checkpoint control protein RAD9A	4	4.01	17.4	2	0.984
sp|Q96RP9|EFGM_HUMAN	GFM1	Elongation factor G, mitochondrial	55.71	56.77	56.2	35	0.984
sp|Q15628|TRADD_HUMAN	TRADD	Tumor necrosis factor receptor type 1-associated DEATH domain protein	6.01	6.02	19.2	3	0.984
sp|Q9Y5P4|C43BP_HUMAN	COL4A3BP	Collagen type IV alpha-3-binding protein	10.5	10.67	29.3	5	0.984
sp|Q13546|RIPK1_HUMAN	RIPK1	Receptor-interacting serine/threonine-protein kinase 1	5.54	7.54	24	6	0.984
sp|Q9P1Y5|CAMP3_HUMAN	CAMSAP3	Calmodulin-regulated spectrin-associated protein 3	25.32	25.97	31.8	16	0.984
sp|O15111|IKKA_HUMAN	CHUK	Inhibitor of nuclear factor kappa-B kinase subunit alpha	14.4	14.98	35.7	8	0.985
sp|Q08AG7|MZT1_HUMAN	MZT1	Mitotic-spindle organizing protein 1	4.65	4.69	56.1	3	0.985
sp|Q9Y530|OARD1_HUMAN	OARD1	O-acetyl-ADP-ribose deacetylase 1	11.27	11.35	61.2	6	0.985
sp|Q5VWQ0|RSBN1_HUMAN	RSBN1	Round spermatid basic protein 1	4.01	4.29	12.3	3	0.985
sp|P08238|HS90B_HUMAN	HSP90AB1	Heat shock protein HSP 90-beta	173.77	173.98	82.7	390	0.985
sp|Q96C86|DCPS_HUMAN	DCPS	m7GpppX diphosphatase	30.24	30.4	58.2	17	0.985
sp|Q9UK41|VPS28_HUMAN	VPS28	Vacuolar protein sorting-associated protein 28 homolog	19.13	19.16	69.7	12	0.985
sp|Q9Y5Y0|FLVC1_HUMAN	FLVCR1	Feline leukemia virus subgroup C receptor-related protein 1	4.12	4.28	20.5	4	0.985
sp|Q9H633|RPP21_HUMAN	RPP21	Ribonuclease P protein subunit p21	5.13	5.28	35.1	3	0.985
sp|O00459|P85B_HUMAN	PIK3R2	Phosphatidylinositol 3-kinase regulatory subunit beta	8.9	9.03	22.3	6	0.985
sp|Q5TEU4|NDUF5_HUMAN	NDUFAF5	NADH dehydrogenase [ubiquinone] 1 alpha subcomplex assembly factor 5	4.53	4.68	20	4	0.985
sp|Q9P2R3|ANFY1_HUMAN	ANKFY1	Rabankyrin-5	44.57	46.75	41.6	28	0.985
sp|Q96B54|ZN428_HUMAN	ZNF428	Zinc finger protein 428	6	6	23.9	4	0.985
sp|Q96ME1|FXL18_HUMAN	FBXL18	F-box/LRR-repeat protein 18	9.49	11.64	19.1	7	0.985
sp|Q92485|ASM3B_HUMAN	SMPDL3B	Acid sphingomyelinase-like phosphodiesterase 3b	3.38	3.46	19.8	2	0.985
sp|Q9NZ43|USE1_HUMAN	USE1	Vesicle transport protein USE1	4.73	5.76	48.7	7	0.985
sp|Q9Y3B8|ORN_HUMAN	REXO2	Oligoribonuclease, mitochondrial	16.69	16.77	59.9	10	0.985

sp|Q9Y6J9|TAF6L_HUMAN	TAF6L	TAF6-like RNA polymerase II p300/CBP-associated factor-associated factor 65 kDa subunit 6L	12.87	13.02	29.3	8	0.985
sp|O95347|SMC2_HUMAN	SMC2	Structural maintenance of chromosomes protein 2	87.49	92.25	61.6	51	0.985
sp|Q16630|CPSF6_HUMAN	CPSF6	Cleavage and polyadenylation specificity factor subunit 6	20.09	20.51	28	14	0.985
sp|Q96HR8|NAF1_HUMAN	NAF1	H/ACA ribonucleoprotein complex non-core subunit NAF1	3.05	3.15	17.6	2	0.985
sp|Q8N122|RPTOR_HUMAN	RPTOR	Regulatory-associated protein of mTOR	19.32	19.66	24	13	0.986
sp|P55145|MANF_HUMAN	MANF	Mesencephalic astrocyte-derived neurotrophic factor	23.21	23.26	64.8	21	0.986
sp|P04181|OAT_HUMAN	OAT	Ornithine aminotransferase, mitochondrial	52.1	55.53	86.8	67	0.986
sp|Q9ULC3|RAB23_HUMAN	RAB23	Ras-related protein Rab-23	15.26	17.46	52.7	10	0.986
sp|O15119|TBX3_HUMAN	TBX3	T-box transcription factor TBX3	4.12	4.2	16.3	2	0.986
sp|Q9NY27|PP4R2_HUMAN	PPP4R2	Serine/threonine-protein phosphatase 4 regulatory subunit 2	25.69	25.88	51.3	16	0.986
sp|O95573|ACSL3_HUMAN	ACSL3	Long-chain-fatty-acid--CoA ligase 3	58.43	67.23	70	56	0.986
sp|Q9GZS3|WDR61_HUMAN	WDR61	WD repeat-containing protein 61	28.87	28.96	70.2	20	0.986
sp|P23610|F8I2_HUMAN	F8A1	Factor VIII intron 22 protein	12.54	12.63	34	9	0.986
sp|Q8WWI5|CTL1_HUMAN	SLC44A1	Choline transporter-like protein 1	13.9	14.05	22.4	9	0.986
sp|Q9UDY8|MALT1_HUMAN	MALT1	Mucosa-associated lymphoid tissue lymphoma translocation protein 1	16.43	16.83	37.3	9	0.986
sp|P46060|RAGP1_HUMAN	RANGAP1	Ran GTPase-activating protein 1	74.97	75.17	78	66	0.986
sp|Q8N1N4|K2C78_HUMAN	KRT78	Keratin, type II cytoskeletal 78	2	7.62	23.7	8	0.986
sp|Q9H8K7|CJ088_HUMAN	C10orf88	Uncharacterized protein C10orf88	2.86	2.93	14.8	2	0.986
sp|O95248|MTMR5_HUMAN	SBF1	Myotubularin-related protein 5	38.59	40.93	27.8	22	0.986
sp|P08319|ADH4_HUMAN	ADH4	Alcohol dehydrogenase 4	13.18	15.77	58.4	11	0.986
sp|Q15334|L2GL1_HUMAN	LLGL1	Lethal(2) giant larvae protein homolog 1	26.21	26.38	27.8	14	0.986
sp|P42167|LAP2B_HUMAN	TMPO	Lamina-associated polypeptide 2, isoforms beta/gamma	23.37	48.38	69.2	53	0.987
sp|Q6ZMR3|LDH6A_HUMAN	LDHAL6A	L-lactate dehydrogenase A-like 6A	2.67	9.34	35.8	17	0.987
sp|Q8N3U4|STAG2_HUMAN	STAG2	Cohesin subunit SA-2	57.92	59.92	44	37	0.987
sp|P52655|TF2AA_HUMAN	GTF2A1	Transcription initiation factor IIA subunit 1	4.89	4.96	13.8	4	0.987
sp|O60783|RT14_HUMAN	MRPS14	28S ribosomal protein S14, mitochondrial	3.83	3.94	43	2	0.987
sp|Q9Y2Q9|RT28_HUMAN	MRPS28	28S ribosomal protein S28, mitochondrial	14.69	14.72	62.6	14	0.987
sp|P82930|RT34_HUMAN	MRPS34	28S ribosomal protein S34, mitochondrial	22.95	25.2	74.8	16	0.988
sp|A4D1E9|GTPBA_HUMAN	GTPBP10	GTP-binding protein 10	15.78	16.08	50.1	11	0.988
sp|Q7Z4Q2|HEAT3_HUMAN	HEATR3	HEAT repeat-containing protein 3	20.12	22.21	38.1	15	0.988
sp|Q96RF0|SNX18_HUMAN	SNX18	Sorting nexin-18	5.42	5.5	13.2	4	0.988
sp|O43670|ZN207_HUMAN	ZNF207	BUB3-interacting and GLEBS motif-containing protein ZNF207	12.73	12.86	20.7	9	0.988
sp|P43243|MATR3_HUMAN	MATR3	Matrin-3	75.05	75.23	66.4	71	0.988
sp|Q8NBI6|XXLT1_HUMAN	XXYLT1	Xyloside xylosyltransferase 1	13.25	13.39	51.7	7	0.988
sp|Q9H267|VP33B_HUMAN	VPS33B	Vacuolar protein sorting-associated protein 33B	23.14	23.31	43.8	12	0.988
sp|O95625|ZBT11_HUMAN	ZBTB11	Zinc finger and BTB domain-containing protein 11	5.43	5.57	18.7	3	0.988
sp|Q06546|GABPA_HUMAN	GABPA	GA-binding protein alpha chain	21.62	22.28	48	14	0.988
sp|Q9UJS0|CMC2_HUMAN	SLC25A13	Calcium-binding mitochondrial carrier protein Aralar2	54.49	55.09	67	48	0.988
sp|Q96AQ8|MCUR1_HUMAN	MCUR1	Mitochondrial calcium uniporter regulator 1	4.83	5.45	37.1	6	0.988
sp|P08670|VIME_HUMAN	VIM	Vimentin	75.99	83.93	88	127	0.988
sp|Q92600|RCD1_HUMAN	RQCD1	Cell differentiation protein RCD1 homolog	17.65	17.69	50.5	9	0.988
sp|P16220|CREB1_HUMAN	CREB1	Cyclic AMP-responsive element-binding protein 1	8.78	9.25	19.9	8	0.988
sp|P18846|ATF1_HUMAN	ATF1	Cyclic AMP-dependent transcription factor ATF-1	2	5.06	17	4	0.988
sp|P12268|IMDH2_HUMAN	IMPDH2	Inosine-5'-monophosphate dehydrogenase 2	61.76	64.42	71.6	54	0.988
sp|P61604|CH10_HUMAN	HSPE1	10 kDa heat shock protein, mitochondrial	22.07	22.33	90.2	36	0.988
sp|Q9HCE5|MET14_HUMAN	METTL14	N6-adenosine-methyltransferase subunit METTL14	5.87	6.17	30.3	3	0.989
sp|Q16625|OCLN_HUMAN	OCLN	Occludin	11.86	12	28.2	10	0.989
sp|P49790|NU153_HUMAN	NUP153	Nuclear pore complex protein Nup153	86.2	86.35	49	47	0.989
sp|O60927|PP1RB_HUMAN	PPP1R11	Protein phosphatase 1 regulatory subunit 11	4.67	4.87	45.2	3	0.989

sp|Q7Z406|MYH14_HUMAN	MYH14	Myosin-14	59.14	87.97	41.9	58	0.989
sp|Q9NZ09|UBAP1_HUMAN	UBAP1	Ubiquitin-associated protein 1	8.53	8.68	19.7	6	0.989
sp|Q9UHR5|S30BP_HUMAN	SAP30BP	SAP30-binding protein	13.88	13.93	38.6	8	0.989
sp|Q96FJ2|DYL2_HUMAN	DYNLL2	Dynein light chain 2, cytoplasmic	4.56	7.8	68.5	8	0.989
sp|O43929|ORC4_HUMAN	ORC4	Origin recognition complex subunit 4	8.62	9.81	39.9	6	0.989
sp|O60888|CUTA_HUMAN	CUTA	Protein CutA	8.02	8.24	70.4	9	0.989
sp|P42338|PK3CB_HUMAN	PIK3CB	Phosphatidylinositol 4,5-bisphosphate 3-kinase catalytic subunit beta isoform	2.74	2.94	21.8	3	0.989
sp|Q9ULT8|HECD1_HUMAN	HECTD1	E3 ubiquitin-protein ligase HECTD1	90.02	93.43	35.3	57	0.989
sp|Q01085|TIAR_HUMAN	TIAL1	Nucleolysin TIAR	16.25	22.97	51.5	15	0.989
sp|O14791|APOL1_HUMAN	APOL1	Apolipoprotein L1	2.21	2.28	21.9	2	0.989
sp|Q9UI26|IPO11_HUMAN	IPO11	Importin-11	11.11	11.92	25	7	0.989
sp|Q8NBF2|NHLC2_HUMAN	NHLRC2	NHL repeat-containing protein 2	28.99	31.31	52.3	21	0.989
sp|O95249|GOSR1_HUMAN	GOSR1	Golgi SNAP receptor complex member 1	6.45	6.61	36	10	0.989
sp|Q9UM13|APC10_HUMAN	ANAPC10	Anaphase-promoting complex subunit 10	1.91	2.11	20.5	3	0.990
sp|O00124|UBXN8_HUMAN	UBXN8	UBX domain-containing protein 8	2.84	2.91	27.4	3	0.990
sp|P31930|QCR1_HUMAN	UQCRC1	Cytochrome b-c1 complex subunit 1, mitochondrial	50.1	50.63	67.1	73	0.990
sp|Q9H0X4|ITFG3_HUMAN	ITFG3	Protein ITFG3	5.83	5.94	19.6	4	0.990
sp|O43252|PAPS1_HUMAN	PAPSS1	Bifunctional 3'-phosphoadenosine 5'-phosphosulfate synthase 1	48.56	50.05	76.6	32	0.990
sp|Q01780|EXOSX_HUMAN	EXOSC10	Exosome component 10	64.43	65.46	60.7	37	0.990
sp|O95816|BAG2_HUMAN	BAG2	BAG family molecular chaperone regulator 2	22.04	22.36	58.3	17	0.990
sp|Q9BSD7|NTPCR_HUMAN	NTPCR	Cancer-related nucleoside-triphosphatase	18.17	18.76	73.2	14	0.990
sp|Q5JWR5|DOP1_HUMAN	DOPEY1	Protein dopey-1	3.07	5	12.5	3	0.990
sp|Q9Y478|AAKB1_HUMAN	PRKAB1	5'-AMP-activated protein kinase subunit beta-1	5.05	7.48	31.1	5	0.990
sp|P49760|CLK2_HUMAN	CLK2	Dual specificity protein kinase CLK2	6	6.03	14.8	3	0.991
sp|Q01804|OTUD4_HUMAN	OTUD4	OTU domain-containing protein 4	2.2	2.31	12.3	2	0.991
sp|Q9UHB6|LIMA1_HUMAN	LIMA1	LIM domain and actin-binding protein 1	41.53	41.89	55.9	24	0.991
sp|P07237|PDIA1_HUMAN	P4HB	Protein disulfide-isomerase	97.4	101.96	91.5	161	0.991
sp|P16260|GDC_HUMAN	SLC25A16	Graves disease carrier protein	2.21	2.23	13.9	2	0.991
sp|P15151|PVR_HUMAN	PVR	Poliovirus receptor	9.07	9.35	22.5	7	0.991
sp|Q13948|CASP_HUMAN	CUX1	Protein CASP	5.38	31.26	51.2	18	0.991
sp|Q96BH1|RNF25_HUMAN	RNF25	E3 ubiquitin-protein ligase RNF25	8.07	8.31	22.9	5	0.991
sp|O94905|ERLN2_HUMAN	ERLIN2	Erlin-2	33.82	35.06	64	26	0.991
sp|Q6I9Y2|THOC7_HUMAN	THOC7	THO complex subunit 7 homolog	8.43	9.67	48.5	6	0.991
sp|Q13444|ADA15_HUMAN	ADAM15	Disintegrin and metalloproteinase domain-containing protein 15	2.68	2.82	8.6	2	0.991
sp|Q86YV9|HPS6_HUMAN	HPS6	Hermansky-Pudlak syndrome 6 protein	4.55	4.68	18.8	4	0.992
sp|P04424|ARLY_HUMAN	ASL	Argininosuccinate lyase	26.89	28.32	46.3	19	0.992
sp|Q15345|LRC41_HUMAN	LRRC41	Leucine-rich repeat-containing protein 41	5.88	6.11	27	5	0.992
sp|P41252|SYIC_HUMAN	IARS	Isoleucine--tRNA ligase, cytoplasmic	114.51	115.19	59.8	74	0.992
sp|Q92922|SMRC1_HUMAN	SMARCC1	SWI/SNF complex subunit SMARCC1	55.69	57.61	46	35	0.992
sp|Q969G6|RIFK_HUMAN	RFK	Riboflavin kinase	5.36	5.42	33.6	3	0.992
sp|P53990|IST1_HUMAN	IST1	IST1 homolog	23.34	23.72	55.8	16	0.992
sp|Q8N983|RM43_HUMAN	MRPL43	39S ribosomal protein L43, mitochondrial	11.24	11.31	48.8	7	0.992
sp|Q9BYN0|SRXN1_HUMAN	SRXN1	Sulfiredoxin-1	4.28	4.31	48.9	2	0.992
sp|O43149|ZZEF1_HUMAN	ZZEF1	Zinc finger ZZ-type and EF-hand domain-containing protein 1	18.82	24.09	17.3	15	0.992
sp|P61970|NTF2_HUMAN	NUTF2	Nuclear transport factor 2	9.68	9.8	61.4	20	0.992
sp|Q9Y490|TLN1_HUMAN	TLN1	Talin-1	231.49	232.16	68.4	179	0.992
sp|P21912|SDHB_HUMAN	SDHB	Succinate dehydrogenase [ubiquinone] iron-sulfur subunit, mitochondrial	30.61	31.03	62.9	22	0.992
sp|A6NDG6|PGP_HUMAN	PGP	Phosphoglycolate phosphatase	19.57	20.49	62.9	15	0.992
sp|Q9UBS0|KS6B2_HUMAN	RPS6KB2	Ribosomal protein S6 kinase beta-2	4.23	6.44	28.6	4	0.993

sp|P52756|RBM5_HUMAN	RBM5	RNA-binding protein 5	15.74	17.97	25.6	11	0.993
sp|Q53S33|BOLA3_HUMAN	BOLA3	BolA-like protein 3	3.83	4.44	55.1	4	0.993
sp|Q9HD15|SRA1_HUMAN	SRA1	Steroid receptor RNA activator 1	11.73	12.76	61.9	10	0.993
sp|Q2M389|WASH7_HUMAN	KIAA1033	WASH complex subunit 7	12.48	13.92	28.9	13	0.993
sp|Q96AA3|RFT1_HUMAN	RFT1	Protein RFT1 homolog	6.99	7.2	22.9	6	0.993
sp|O94776|MTA2_HUMAN	MTA2	Metastasis-associated protein MTA2	36.87	45.32	56.6	29	0.993
sp|O43865|SAHH2_HUMAN	AHCYL1	Adenosylhomocysteinase 2	35.1	40.86	57.2	28	0.993
sp|Q9NVV5|AIG1_HUMAN	AIG1	Androgen-induced gene 1 protein	4.46	4.5	13.5	3	0.993
sp|Q8N4Q1|MIA40_HUMAN	CHCHD4	Mitochondrial intermembrane space import and assembly protein 40	4.68	5.15	37.3	3	0.993
sp|P50995|ANX11_HUMAN	ANXA11	Annexin A11	33.51	35.79	44.6	22	0.993
sp|P40222|TXLNA_HUMAN	TXLNA	Alpha-taxilin	45.28	45.45	65.2	28	0.993
sp|Q6UW78|UQCC3_HUMAN	UQCC3	Ubiquinol-cytochrome-c reductase complex assembly factor 3	3.26	3.35	53.8	7	0.994
sp|Q9HBL8|NMRL1_HUMAN	NMRAL1	NmrA-like family domain-containing protein 1	4.02	4.17	35.1	3	0.994
sp|P11021|GRP78_HUMAN	HSPA5	78 kDa glucose-regulated protein	131.13	132.91	89.1	233	0.994
sp|Q9BUL8|PDC10_HUMAN	PDCD10	Programmed cell death protein 10	13	13.42	73.1	9	0.994
sp|Q01082|SPTB2_HUMAN	SPTBN1	Spectrin beta chain, non-erythrocytic 1	277.45	278.12	77.1	242	0.994
sp|O43181|NDUS4_HUMAN	NDUFS4	NADH dehydrogenase [ubiquinone] iron-sulfur protein 4, mitochondrial	13.97	14.01	64	11	0.994
sp|O75182|SIN3B_HUMAN	SIN3B	Paired amphipathic helix protein Sin3b	8.13	8.72	15.2	7	0.994
sp|Q53GS9|SNUT2_HUMAN	USP39	U4/U6.U5 tri-snRNP-associated protein 2	25.99	26.57	45.8	19	0.994
sp|Q7Z460|CLAP1_HUMAN	CLASP1	CLIP-associating protein 1	63.7	69.96	36.5	38	0.994
sp|O75319|DUS11_HUMAN	DUSP11	RNA/RNP complex-1-interacting phosphatase	8.99	9.08	25.8	6	0.994
sp|P26640|SYVC_HUMAN	VARS	Valine--tRNA ligase	99.25	99.98	60.5	75	0.994
sp|P42566|EPS15_HUMAN	EPS15	Epidermal growth factor receptor substrate 15	28.45	29.04	40.3	18	0.994
sp|Q9NYR9|KBRS2_HUMAN	NKIRAS2	NF-kappa-B inhibitor-interacting Ras-like protein 2	3.75	3.84	27.8	3	0.994
sp|P40926|MDHM_HUMAN	MDH2	Malate dehydrogenase, mitochondrial	51.7	52.18	88.2	104	0.994
sp|Q13085|ACACA_HUMAN	ACACA	Acetyl-CoA carboxylase 1	102.89	104.84	41.5	63	0.994
sp|Q8TCS8|PNPT1_HUMAN	PNPT1	Polyribonucleotide nucleotidyltransferase 1, mitochondrial	71.13	72.06	68.1	46	0.994
sp|Q8TBF4|ZCRB1_HUMAN	ZCRB1	Zinc finger CCHC-type and RNA-binding motif-containing protein 1	4.11	4.13	47.5	2	0.994
sp|Q9NTZ6|RBM12_HUMAN	RBM12	RNA-binding protein 12	36.92	37.17	28.7	24	0.994
sp|P46976|GLYG_HUMAN	GYG1	Glycogenin-1	16.43	16.5	35.1	10	0.995
sp|Q7Z4H8|KDEL2_HUMAN	KDELC2	KDEL motif-containing protein 2	29.71	29.76	51.5	17	0.995
sp|Q5VUA4|ZN318_HUMAN	ZNF318	Zinc finger protein 318	8.32	8.73	20.2	5	0.995
sp|P20248|CCNA2_HUMAN	CCNA2	Cyclin-A2	9.4	9.51	33.1	6	0.995
sp|Q7Z2Z2|ETUD1_HUMAN	EFTUD1	Elongation factor Tu GTP-binding domain-containing protein 1	17.77	18.4	32.9	11	0.995
sp|P61803|DAD1_HUMAN	DAD1	Dolichyl-diphosphooligosaccharide--protein glycosyltransferase subunit DAD1	6	6	36.3	5	0.995
sp|Q8N3R9|MPP5_HUMAN	MPP5	MAGUK p55 subfamily member 5	10.3	10.84	30.8	7	0.995
sp|Q9BRX9|WDR83_HUMAN	WDR83	WD repeat domain-containing protein 83	4.64	4.71	17.1	3	0.995
sp|P55201|BRPF1_HUMAN	BRPF1	Peregrin	4.55	9.95	21.4	9	0.995
sp|Q12824|SNF5_HUMAN	SMARCB1	SWI/SNF-related matrix-associated actin-dependent regulator of chromatin subfamily B member 1	19.64	19.75	53.3	14	0.995
sp|Q9UFF9|CNOT8_HUMAN	CNOT8	CCR4-NOT transcription complex subunit 8	2.5	2.54	29.5	3	0.995
sp|Q66PJ3|AR6P4_HUMAN	ARL6IP4	ADP-ribosylation factor-like protein 6-interacting protein 4	4.93	5.03	23.5	3	0.995
sp|Q8WYP5|ELYS_HUMAN	AHCTF1	Protein ELYS	88.66	89.81	40	52	0.995
sp|Q9NQP4|PFD4_HUMAN	PFDN4	Prefoldin subunit 4	8.38	9.63	67.9	6	0.995
sp|O94903|PROSC_HUMAN	PROSC	Proline synthase co-transcribed bacterial homolog protein	17.8	18.36	49.1	11	0.995
sp|Q9BUE6|ISCA1_HUMAN	ISCA1	Iron-sulfur cluster assembly 1 homolog, mitochondrial	7.78	7.94	59.7	5	0.996
sp|P22314|UBA1_HUMAN	UBA1	Ubiquitin-like modifier-activating enzyme 1	117.68	118.67	76.7	172	0.996
sp|P20674|COX5A_HUMAN	COX5A	Cytochrome c oxidase subunit 5A, mitochondrial	21.87	23.99	82.7	23	0.996
sp|P49914|MTHFS_HUMAN	MTHFS	5-formyltetrahydrofolate cyclo-ligase	10.19	10.91	60.6	8	0.996
sp|Q5VT66|MARC1_HUMAN	MARC1	Mitochondrial amidoxime-reducing component 1	13.08	13.31	45.4	10	0.996

sp|O76024|WFS1_HUMAN	WFS1	Wolframin	34.09	34.22	39.8	23	0.996
sp|O00743|PPP6_HUMAN	PPP6C	Serine/threonine-protein phosphatase 6 catalytic subunit	18.85	20.37	65.9	17	0.996
sp|Q5VWJ9|SNX30_HUMAN	SNX30	Sorting nexin-30	5.76	6.55	33.2	5	0.996
sp|Q8NDX6|ZN740_HUMAN	ZNF740	Zinc finger protein 740	10	10	39.4	5	0.996
sp|Q9Y3E7|CHMP3_HUMAN	CHMP3	Charged multivesicular body protein 3	6.15	6.48	30.2	4	0.996
sp|Q86UE8|TLK2_HUMAN	TLK2	Serine/threonine-protein kinase tousled-like 2	5.22	10.51	20.7	10	0.997
sp|Q14119|VEZF1_HUMAN	VEZF1	Vascular endothelial zinc finger 1	4.03	4.04	14	2	0.997
sp|Q9Y259|CHKB_HUMAN	CHKB	Choline/ethanolamine kinase	1.77	1.91	9.4	2	0.997
sp|P05787|K2C8_HUMAN	KRT8	Keratin, type II cytoskeletal 8	107.4	107.87	88	232	0.997
sp|Q9BSH4|TACO1_HUMAN	TACO1	Translational activator of cytochrome c oxidase 1	23.58	23.68	66.3	15	0.997
sp|Q9BZX2|UCK2_HUMAN	UCK2	Uridine-cytidine kinase 2	10.81	10.89	33.7	9	0.997
sp|Q9HAN9|NMNA1_HUMAN	NMNAT1	Nicotinamide/nicotinic acid mononucleotide adenylyltransferase 1	15.11	15.16	38	8	0.997
sp|Q6P9B6|TLDC1_HUMAN	TLDC1	TLD domain-containing protein 1	15.3	15.42	36	9	0.997
sp|Q969Q5|RAB24_HUMAN	RAB24	Ras-related protein Rab-24	16.1	16.19	59.1	9	0.997
sp|O43396|TXNL1_HUMAN	TXNL1	Thioredoxin-like protein 1	24.79	26.83	74.1	21	0.997
sp|P10809|CH60_HUMAN	HSPD1	60 kDa heat shock protein, mitochondrial	160.89	162.85	91.1	584	0.997
sp|Q9BRD0|BUD13_HUMAN	BUD13	BUD13 homolog	5.06	5.44	19.9	3	0.997
sp|Q9UMR5|PPT2_HUMAN	PPT2	Lysosomal thioesterase PPT2	4	4.04	16.9	2	0.997
sp|O14967|CLGN_HUMAN	CLGN	Calmegin	10.59	11.69	30.3	7	0.997
sp|P48681|NEST_HUMAN	NES	Nestin	4.28	4.48	15.2	6	0.997
sp|Q6NW29|RWDD4_HUMAN	RWDD4	RWD domain-containing protein 4	5.53	5.67	33.5	4	0.997
sp|Q96D71|REPS1_HUMAN	REPS1	RalBP1-associated Eps domain-containing protein 1	21.9	23.17	31.3	17	0.997
sp|Q9Y639|NPTN_HUMAN	NPTN	Neuroplastin	15.37	15.53	26.4	8	0.997
sp|Q9NXA8|SIR5_HUMAN	SIRT5	NAD-dependent protein deacylase sirtuin-5, mitochondrial	10.06	10.2	32.9	6	0.997
sp|P32321|DCTD_HUMAN	DCTD	Deoxycytidylate deaminase	11.07	11.18	53.9	10	0.997
sp|Q8ND56|LS14A_HUMAN	LSM14A	Protein LSM14 homolog A	10.9	11.82	32.2	8	0.997
sp|Q9NTG7|SIR3_HUMAN	SIRT3	NAD-dependent protein deacetylase sirtuin-3, mitochondrial	2.3	2.34	17	2	0.997
sp|Q9HC62|SENP2_HUMAN	SENP2	Sentrin-specific protease 2	4.06	4.1	26.2	3	0.998
sp|P07948|LYN_HUMAN	LYN	Tyrosine-protein kinase Lyn	24.89	29.77	56.8	17	0.998
sp|Q9NWS0|PIHD1_HUMAN	PIH1D1	PIH1 domain-containing protein 1	18.16	18.21	62.4	11	0.998
sp|P19652|A1AG2_HUMAN	ORM2	Alpha-1-acid glycoprotein 2	2.21	2.38	19.4	2	0.998
sp|P50502|F10A1_HUMAN	ST13	Hsc70-interacting protein	25.67	25.84	55.6	29	0.998
sp|O96011|PX11B_HUMAN	PEX11B	Peroxisomal membrane protein 11B	8.1	8.31	59.1	6	0.998
sp|P19256|LFA3_HUMAN	CD58	Lymphocyte function-associated antigen 3	5.59	5.64	16	3	0.998
sp|Q13416|ORC2_HUMAN	ORC2	Origin recognition complex subunit 2	15.32	15.78	36.7	9	0.998
sp|Q99733|NP1L4_HUMAN	NAP1L4	Nucleosome assembly protein 1-like 4	28.06	30.51	68.8	25	0.998
sp|Q13330|MTA1_HUMAN	MTA1	Metastasis-associated protein MTA1	51.93	52.06	62	34	0.998
sp|Q96EX3|WDR34_HUMAN	WDR34	WD repeat-containing protein 34	4.58	4.62	13.3	3	0.998
sp|Q5BKY9|F133B_HUMAN	FAM133B	Protein FAM133B	1.33	1.6	19.4	2	0.998
sp|Q75N03|HAKAI_HUMAN	CBLL1	E3 ubiquitin-protein ligase Hakai	5.15	5.24	17.3	3	0.998
sp|Q8TBZ3|WDR20_HUMAN	WDR20	WD repeat-containing protein 20	8.36	8.5	24.1	6	0.998
sp|Q9UPM8|AP4E1_HUMAN	AP4E1	AP-4 complex subunit epsilon-1	2.17	2.97	22	2	0.998
sp|Q9BYJ9|YTHD1_HUMAN	YTHDF1	YTH domain-containing family protein 1	14.31	20.31	26.8	13	0.999
sp|P30049|ATPD_HUMAN	ATP5D	ATP synthase subunit delta, mitochondrial	11.22	11.4	61.3	19	0.999
sp|P00492|HPRT_HUMAN	HPRT1	Hypoxanthine-guanine phosphoribosyltransferase	30.42	30.48	81.2	26	0.999
sp|Q9NP77|SSU72_HUMAN	SSU72	RNA polymerase II subunit A C-terminal domain phosphatase SSU72	8.34	8.44	54.1	7	0.999
sp|P31946|1433B_HUMAN	YWHAB	14-3-3 protein beta/alpha	19.75	38.48	88.6	56	0.999
sp|Q8N4P3|MESH1_HUMAN	HDDC3	Guanosine-3',5'-bis(diphosphate) 3'-pyrophosphohydrolase MESH1	3.31	3.47	41.3	4	0.999
sp|Q9UNM6|PSD13_HUMAN	PSMD13	26S proteasome non-ATPase regulatory subunit 13	43.93	44.35	66.5	33	0.999

sp|P02794|FRIH_HUMAN	FTH1	Ferritin heavy chain	9.02	9.18	53.6	5	0.999
sp|Q9H257|CARD9_HUMAN	CARD9	Caspase recruitment domain-containing protein 9	2.38	2.44	19.2	2	0.999
sp|P21675|TAF1_HUMAN	TAF1	Transcription initiation factor TFIID subunit 1	2.14	2.45	11.8	2	0.999
sp|P30047|GFRP_HUMAN	GCHFR	GTP cyclohydrolase 1 feedback regulatory protein	8	8.23	91.7	4	0.999
sp|Q13404|UB2V1_HUMAN	UBE2V1	Ubiquitin-conjugating enzyme E2 variant 1	18.8	19.42	89.1	20	0.999
sp|O76094|SRP72_HUMAN	SRP72	Signal recognition particle subunit SRP72	55.58	57.83	67.8	36	1.000
sp|Q96HA7|TONSL_HUMAN	TONSL	Tonsoku-like protein	3.67	4.16	12.3	6	1.000
sp|O60701|UGDH_HUMAN	UGDH	UDP-glucose 6-dehydrogenase	87.03	88.84	89.9	153	1.000
sp|P12277|KCRB_HUMAN	CKB	Creatine kinase B-type	58.29	58.69	94.2	138	1.000
sp|Q5VIR6|VPS53_HUMAN	VPS53	Vacuolar protein sorting-associated protein 53 homolog	14.33	15.16	33.3	12	1.000
sp|Q92572|AP3S1_HUMAN	AP3S1	AP-3 complex subunit sigma-1	13.62	13.66	52.9	8	1.000
sp|P05783|K1C18_HUMAN	KRT18	Keratin, type I cytoskeletal 18	95.83	96.13	91.9	178	1.000
sp|Q15286|RAB35_HUMAN	RAB35	Ras-related protein Rab-35	16.05	21.91	84.6	19	1.000
sp|Q8TBC3|SHKB1_HUMAN	SHKBP1	SH3KBP1-binding protein 1	3.26	4.59	19.2	4	1.000
sp|Q9NX46|ARHL2_HUMAN	ADPRHL2	Poly(ADP-ribose) glycohydrolase ARH3	19.33	19.48	52.1	11	1.000
sp|O15031|PLXB2_HUMAN	PLXNB2	Plexin-B2	61.48	62.47	33.4	33	1.000
sp|O60476|MA1A2_HUMAN	MAN1A2	Mannosyl-oligosaccharide 1,2-alpha-mannosidase IB	12.37	12.63	24.7	8	1.000
sp|O94964|SOGA1_HUMAN	SOGA1	Protein SOGA1	5.37	6.19	20	7	1.000
sp|Q14344|GNA13_HUMAN	GNA13	Guanine nucleotide-binding protein subunit alpha-13	16.6	19.96	53.9	14	1.001
sp|Q9UBP0|SPAST_HUMAN	SPAST	Spastin	16.24	16.62	40.4	9	1.001
sp|P08754|GNAI3_HUMAN	GNAI3	Guanine nucleotide-binding protein G(k) subunit alpha	12.06	26.58	70.1	21	1.001
sp|P27449|VATL_HUMAN	ATP6V0C	V-type proton ATPase 16 kDa proteolipid subunit	1.85	1.96	14.8	3	1.001
sp|Q9NW15|ANO10_HUMAN	ANO10	Anoctamin-10	7.31	7.75	23	6	1.001
sp|Q9UBL3|ASH2L_HUMAN	ASH2L	Set1/Ash2 histone methyltransferase complex subunit ASH2	14.39	14.57	30.1	11	1.001
sp|Q9H9T3|ELP3_HUMAN	ELP3	Elongator complex protein 3	14.36	15.05	33.6	9	1.001
sp|Q13505|MTX1_HUMAN	MTX1	Metaxin-1	13.02	13.24	37.6	10	1.001
sp|Q5SWX8|ODR4_HUMAN	ODR4	Protein odr-4 homolog	15.18	15.35	37.7	11	1.001
sp|Q15904|VAS1_HUMAN	ATP6AP1	V-type proton ATPase subunit S1	9.6	10.12	30.2	11	1.001
sp|Q9Y2G8|DJC16_HUMAN	DNAJC16	DnaJ homolog subfamily C member 16	2.44	2.48	23.5	2	1.002
sp|Q9Y450|HBS1L_HUMAN	HBS1L	HBS1-like protein	49.96	50.67	67.7	28	1.002
sp|O60885|BRD4_HUMAN	BRD4	Bromodomain-containing protein 4	27.48	27.85	17.9	18	1.002
sp|Q09328|MGT5A_HUMAN	MGAT5	Alpha-1,6-mannosylglycoprotein 6-beta-N-acetylglucosaminyltransferase A	3.11	3.36	17.4	3	1.002
sp|O95394|AGM1_HUMAN	PGM3	Phosphoacetylglucosamine mutase	41.53	41.68	64.4	37	1.002
sp|Q92830|KAT2A_HUMAN	KAT2A	Histone acetyltransferase KAT2A	2.54	4.13	22.3	6	1.002
sp|Q5VW32|BROX_HUMAN	BROX	BRO1 domain-containing protein BROX	18.71	21	55	14	1.002
sp|O15131|IMA6_HUMAN	KPNA5	Importin subunit alpha-6	2.01	15.87	38.4	12	1.002
sp|P11047|LAMC1_HUMAN	LAMC1	Laminin subunit gamma-1	76.68	78.51	47.1	49	1.002
sp|Q63ZY3|KANK2_HUMAN	KANK2	KN motif and ankyrin repeat domain-containing protein 2	4.04	6.46	20.5	5	1.002
sp|P05543|THBG_HUMAN	SERPINA7	Thyroxine-binding globulin	2.01	3.09	17.4	3	1.002
sp|Q9BZ67|FRMD8_HUMAN	FRMD8	FERM domain-containing protein 8	5.01	5.07	14.4	3	1.003
sp|Q9UNP9|PPIE_HUMAN	PPIE	Peptidyl-prolyl cis-trans isomerase E	5.33	8.17	57.8	8	1.003
sp|Q5ZPR3|CD276_HUMAN	CD276	CD276 antigen	7.97	8.88	37.3	12	1.003
sp|P62316|SMD2_HUMAN	SNRPD2	Small nuclear ribonucleoprotein Sm D2	17.82	17.97	71.2	19	1.003
sp|Q5TFE4|NT5D1_HUMAN	NT5DC1	5'-nucleotidase domain-containing protein 1	26.7	27.04	65.3	20	1.003
sp|Q9H061|T126A_HUMAN	TMEM126A	Transmembrane protein 126A	8.03	8.61	45.1	7	1.003
sp|P57078|RIPK4_HUMAN	RIPK4	Receptor-interacting serine/threonine-protein kinase 4	2.73	3.65	13.3	3	1.003
sp|Q8N7H5|PAF1_HUMAN	PAF1	RNA polymerase II-associated factor 1 homolog	34.82	34.93	55	24	1.003
sp|Q96JB2|COG3_HUMAN	COG3	Conserved oligomeric Golgi complex subunit 3	24.62	25.7	42.3	21	1.003
sp|O14497|ARI1A_HUMAN	ARID1A	AT-rich interactive domain-containing protein 1A	38.64	38.84	24.8	22	1.003

sp|Q8WW59|SPRY4_HUMAN	SPRYD4	SPRY domain-containing protein 4	20.28	20.48	76.8	13	1.003
sp|P15056|BRAF_HUMAN	BRAF	Serine/threonine-protein kinase B-raf	2.03	8.65	23.9	6	1.003
sp|Q14919|NC2A_HUMAN	DRAP1	Dr1-associated corepressor	9.59	9.66	33.2	9	1.003
sp|P47712|PA24A_HUMAN	PLA2G4A	Cytosolic phospholipase A2	12.58	13.15	21.6	8	1.004
sp|Q9NR28|DBLOH_HUMAN	DIABLO	Diablo homolog, mitochondrial	16.59	16.66	57.7	18	1.004
sp|Q9P2W1|HOP2_HUMAN	PSMC3IP	Homologous-pairing protein 2 homolog	8.01	8.02	38.3	4	1.004
sp|Q9NX58|LYAR_HUMAN	LYAR	Cell growth-regulating nucleolar protein	33.31	36.45	61.2	23	1.004
sp|Q66K74|MAP1S_HUMAN	MAP1S	Microtubule-associated protein 1S	26.82	26.97	26.5	16	1.004
sp|Q9UJX3|APC7_HUMAN	ANAPC7	Anaphase-promoting complex subunit 7	37.91	38.7	46.1	20	1.004
sp|P0CG12|CTF8A_HUMAN	CHTF8	Chromosome transmission fidelity protein 8 homolog isoform 2	2.08	2.1	17.8	3	1.004
sp|P22830|HEMH_HUMAN	FECH	Ferrochelatase, mitochondrial	20.62	20.94	61.2	14	1.004
sp|Q9NYG2|ZDHC3_HUMAN	ZDHHC3	Palmitoyltransferase ZDHHC3	1.45	1.66	13.4	2	1.004
sp|Q9UID3|VPS51_HUMAN	VPS51	Vacuolar protein sorting-associated protein 51 homolog	16.37	16.55	31.7	10	1.004
sp|P04003|C4BPA_HUMAN	C4BPA	C4b-binding protein alpha chain	2.49	2.82	10.2	3	1.004
sp|Q6P5R6|RL22L_HUMAN	RPL22L1	60S ribosomal protein L22-like 1	4.69	5.21	52.5	9	1.004
sp|Q8N5K1|CISD2_HUMAN	CISD2	CDGSH iron-sulfur domain-containing protein 2	13.38	14.35	62.2	12	1.004
sp|Q9UKY1|ZHX1_HUMAN	ZHX1	Zinc fingers and homeoboxes protein 1	2.05	2.07	14.9	2	1.004
sp|Q6RFH5|WDR74_HUMAN	WDR74	WD repeat-containing protein 74	15.66	15.83	57.4	9	1.005
sp|Q8NI60|ADCK3_HUMAN	ADCK3	Atypical kinase ADCK3, mitochondrial	35.19	35.23	45	28	1.005
sp|Q6PJ69|TRI65_HUMAN	TRIM65	Tripartite motif-containing protein 65	3.56	3.76	16.1	5	1.005
sp|O75521|ECI2_HUMAN	ECI2	Enoyl-CoA delta isomerase 2, mitochondrial	37.95	38	65	37	1.005
sp|P40939|ECHA_HUMAN	HADHA	Trifunctional enzyme subunit alpha, mitochondrial	93.35	93.92	74.1	104	1.005
sp|Q8IV48|ERI1_HUMAN	ERI1	3'-5' exoribonuclease 1	3.14	3.23	19.8	2	1.005
sp|O96017|CHK2_HUMAN	CHEK2	Serine/threonine-protein kinase Chk2	6.18	6.27	31.5	7	1.006
sp|O95319|CELF2_HUMAN	CELF2	CUGBP Elav-like family member 2	2.2	4.62	29.1	3	1.006
sp|Q9NUX5|POTE1_HUMAN	POT1	Protection of telomeres protein 1	5.03	5.3	17.5	3	1.006
sp|O00258|WRB_HUMAN	WRB	Tail-anchored protein insertion receptor WRB	2.08	2.12	31.6	3	1.006
sp|Q8N9N5|BANP_HUMAN	BANP	Protein BANP	7.2	7.3	21.2	4	1.006
sp|Q643R3|LPCT4_HUMAN	LPCAT4	Lysophospholipid acyltransferase LPCAT4	2.5	2.54	20.2	2	1.006
sp|Q9NVI1|FANCI_HUMAN	FANCI	Fanconi anemia group I protein	46.84	51.65	39.2	27	1.006
sp|Q9UFN0|NPS3A_HUMAN	NIPSNAP3A	Protein NipSnap homolog 3A	15.69	15.85	64	11	1.006
sp|Q14197|ICT1_HUMAN	ICT1	Peptidyl-tRNA hydrolase ICT1, mitochondrial	14.46	14.57	57.8	11	1.006
sp|P42575|CASP2_HUMAN	CASP2	Caspase-2	3.26	5.93	26.1	4	1.006
sp|Q92614|MY18A_HUMAN	MYO18A	Unconventional myosin-XVIIIa	57.46	59.75	32.1	32	1.006
sp|Q15642|CIP4_HUMAN	TRIP10	Cdc42-interacting protein 4	23.54	23.85	43.4	15	1.006
sp|Q9Y6A1|POMT1_HUMAN	POMT1	Protein O-mannosyl-transferase 1	2.49	2.54	8.3	3	1.006
sp|P42330|AK1C3_HUMAN	AKR1C3	Aldo-keto reductase family 1 member C3	20	55.69	88.5	73	1.007
sp|Q4U2R6|RM51_HUMAN	MRPL51	39S ribosomal protein L51, mitochondrial	1.83	2.03	22.7	2	1.007
sp|O43542|XRCC3_HUMAN	XRCC3	DNA repair protein XRCC3	5.26	5.39	38.7	4	1.007
sp|P51608|MECP2_HUMAN	MECP2	Methyl-CpG-binding protein 2	11.47	11.91	35.4	9	1.007
sp|Q7Z4G1|COMD6_HUMAN	COMMD6	COMM domain-containing protein 6	4.73	4.87	52.9	3	1.007
sp|Q6P1A2|MBOA5_HUMAN	LPCAT3	Lysophospholipid acyltransferase 5	8.76	8.82	25.1	5	1.007
sp|O60664|PLIN3_HUMAN	PLIN3	Perilipin-3	46.35	47.06	87.8	51	1.007
sp|P42898|MTHR_HUMAN	MTHFR	Methylenetetrahydrofolate reductase	3.75	4.24	18.9	4	1.007
sp|O95104|SFR15_HUMAN	SCAF4	Splicing factor, arginine/serine-rich 15	23.16	24.25	28.6	19	1.007
sp|P51970|NDUA8_HUMAN	NDUFA8	NADH dehydrogenase [ubiquinone] 1 alpha subcomplex subunit 8	21.87	24.05	85.5	14	1.007
sp|Q96MW5|COG8_HUMAN	COG8	Conserved oligomeric Golgi complex subunit 8	19.85	20.04	38.2	12	1.007
sp|O75376|NCOR1_HUMAN	NCOR1	Nuclear receptor corepressor 1	18.45	18.76	15.2	11	1.007
sp|Q8IWE4|DCNL3_HUMAN	DCUN1D3	DCN1-like protein 3	5.03	5.36	35.5	4	1.007

sp|P17152|TMM11_HUMAN	TMEM11	Transmembrane protein 11, mitochondrial	4.93	5.15	15.6	3	1.007
sp|Q96C23|GALM_HUMAN	GALM	Aldose 1-epimerase	19.55	19.6	59.7	11	1.007
sp|Q13129|RLF_HUMAN	RLF	Zinc finger protein Rlf	2.08	2.13	11	3	1.007
sp|Q9UIF9|BAZ2A_HUMAN	BAZ2A	Bromodomain adjacent to zinc finger domain protein 2A	42.6	43.06	28.8	30	1.007
sp|Q9P0I2|EMC3_HUMAN	EMC3	ER membrane protein complex subunit 3	11.81	11.94	40.6	6	1.007
sp|P67775|PP2AA_HUMAN	PPP2CA	Serine/threonine-protein phosphatase 2A catalytic subunit alpha isoform	27.26	28.93	74.1	28	1.007
sp|Q9UPT9|UBP22_HUMAN	USP22	Ubiquitin carboxyl-terminal hydrolase 22	4.75	4.85	19.4	5	1.008
sp|P63165|SUMO1_HUMAN	SUMO1	Small ubiquitin-related modifier 1	5.77	6.56	84.2	7	1.008
sp|Q9NR46|SHLB2_HUMAN	SH3GLB2	Endophilin-B2	17.27	17.39	40.3	10	1.008
sp|P62253|UB2G1_HUMAN	UBE2G1	Ubiquitin-conjugating enzyme E2 G1	8.71	8.81	41.8	6	1.008
sp|Q92797|SYMPK_HUMAN	SYMPK	Symplekin	50.25	53.04	46.2	36	1.008
sp|Q5T1V6|DDX59_HUMAN	DDX59	Probable ATP-dependent RNA helicase DDX59	4.09	6.66	25	4	1.008
sp|Q8IXI2|MIRO1_HUMAN	RHOT1	Mitochondrial Rho GTPase 1	17.49	18.44	39.6	10	1.008
sp|O14523|C2C2L_HUMAN	C2CD2L	C2 domain-containing protein 2-like	2.24	2.41	14.2	2	1.008
sp|Q8WXE0|CSKI2_HUMAN	CASKIN2	Caskin-2	2.16	4.25	13.6	3	1.008
sp|P30044|PRDX5_HUMAN	PRDX5	Peroxiredoxin-5, mitochondrial	22.15	22.4	63.1	29	1.009
sp|Q13617|CUL2_HUMAN	CUL2	Cullin-2	34.52	37.68	47.7	24	1.009
sp|Q9UM54|MYO6_HUMAN	MYO6	Unconventional myosin-VI	47.99	48.88	37.1	28	1.009
sp|Q9H4I3|TRABD_HUMAN	TRABD	TraB domain-containing protein	17.85	17.89	43.4	9	1.009
sp|Q13547|HDAC1_HUMAN	HDAC1	Histone deacetylase 1	17.11	25.65	47.3	17	1.009
sp|P55268|LAMB2_HUMAN	LAMB2	Laminin subunit beta-2	17.61	18.19	17	9	1.009
sp|O75449|KTNA1_HUMAN	KATNA1	Katanin p60 ATPase-containing subunit A1	24.32	27.09	46.2	15	1.009
sp|Q01167|FOXK2_HUMAN	FOXK2	Forkhead box protein K2	4.91	5.01	15.8	4	1.009
sp|Q9H7E9|CH033_HUMAN	C8orf33	UPF0488 protein C8orf33	8.26	8.45	53.7	5	1.009
sp|Q9NP73|ALG13_HUMAN	ALG13	Putative bifunctional UDP-N-acetylglucosamine transferase and deubiquitinase ALG13	5.08	7.6	12.7	6	1.009
sp|Q9HBH0|RHOF_HUMAN	RHOF	Rho-related GTP-binding protein RhoF	2.5	4.91	26.1	4	1.009
sp|Q9UHY1|NRBP_HUMAN	NRBP1	Nuclear receptor-binding protein	19.86	19.99	41.9	11	1.009
sp|Q7L590|MCM10_HUMAN	MCM10	Protein MCM10 homolog	6.07	6.12	21.4	3	1.010
sp|P60604|UB2G2_HUMAN	UBE2G2	Ubiquitin-conjugating enzyme E2 G2	5.85	5.92	32.7	5	1.010
sp|Q9NX47|MARH5_HUMAN	5-Mar	E3 ubiquitin-protein ligase MARCH5	10.39	10.55	48.9	6	1.010
sp|O60333|KIF1B_HUMAN	KIF1B	Kinesin-like protein KIF1B	18.86	24.25	23.5	13	1.010
sp|Q9NZD8|SPG21_HUMAN	SPG21	Maspardin	14.45	14.47	35.1	7	1.010
sp|P07919|QCR6_HUMAN	UQCRH	Cytochrome b-c1 complex subunit 6, mitochondrial	8.57	8.73	78	9	1.010
sp|O75676|KS6A4_HUMAN	RPS6KA4	Ribosomal protein S6 kinase alpha-4	6.07	9.6	19.2	7	1.010
sp|P13861|KAP2_HUMAN	PRKAR2A	cAMP-dependent protein kinase type II-alpha regulatory subunit	41.71	42.35	68.6	24	1.010
sp|Q9UG56|PISD_HUMAN	PISD	Phosphatidylserine decarboxylase proenzyme	7.73	9.82	22.5	6	1.010
sp|P27816|MAP4_HUMAN	MAP4	Microtubule-associated protein 4	116.01	117.01	73.8	74	1.010
sp|Q12834|CDC20_HUMAN	CDC20	Cell division cycle protein 20 homolog	3.16	3.25	21.8	2	1.010
sp|Q5MNZ6|WIPI3_HUMAN	WDR45B	WD repeat domain phosphoinositide-interacting protein 3	9.31	9.45	31.4	5	1.010
sp|Q9NS91|RAD18_HUMAN	RAD18	E3 ubiquitin-protein ligase RAD18	16.32	16.95	41.2	12	1.010
sp|Q9UQE7|SMC3_HUMAN	SMC3	Structural maintenance of chromosomes protein 3	99.86	103.65	63.4	72	1.011
sp|P37802|TAGL2_HUMAN	TAGLN2	Transgelin-2	22.19	22.24	70.4	33	1.011
sp|P11182|ODB2_HUMAN	DBT	Lipoamide acyltransferase component of branched-chain alpha-keto acid dehydrogenase complex	25.14	25.28	48.1	13	1.011
sp|Q86VU5|CMTD1_HUMAN	COMTD1	Catechol O-methyltransferase domain-containing protein 1	10.38	10.44	46.6	5	1.011
sp|Q86YP4|P66A_HUMAN	GATAD2A	Transcriptional repressor p66-alpha	22.44	28.81	53.4	18	1.011
sp|Q86Y91|KI18B_HUMAN	KIF18B	Kinesin-like protein KIF18B	6.05	6.14	15.1	3	1.011
sp|P29966|MARCS_HUMAN	MARCKS	Myristoylated alanine-rich C-kinase substrate	23.62	23.67	51.2	31	1.011
sp|P18510|IL1RA_HUMAN	IL1RN	Interleukin-1 receptor antagonist protein	6.16	6.2	49.2	3	1.011
sp|Q6P179|ERAP2_HUMAN	ERAP2	Endoplasmic reticulum aminopeptidase 2	4.58	4.75	18.4	4	1.012

sp|Q7L1V2|MON1B_HUMAN	MON1B	Vacuolar fusion protein MON1 homolog B	2.75	2.82	9	3	1.012
sp|O60566|BUB1B_HUMAN	BUB1B	Mitotic checkpoint serine/threonine-protein kinase BUB1 beta	22.21	24.77	33.9	18	1.012
sp|Q3V6T2|GRDN_HUMAN	CCDC88A	Girdin	13.54	15.26	30.2	11	1.012
sp|Q13232|NDK3_HUMAN	NME3	Nucleoside diphosphate kinase 3	11.63	13.19	69.2	8	1.012
sp|P53597|SUCA_HUMAN	SUCLG1	Succinyl-CoA ligase [ADP/GDP-forming] subunit alpha, mitochondrial	18.02	18.12	47.1	16	1.012
sp|Q9Y697|NFS1_HUMAN	NFS1	Cysteine desulfurase, mitochondrial	21.7	24.4	67.6	19	1.012
sp|Q7KZI7|MARK2_HUMAN	MARK2	Serine/threonine-protein kinase MARK2	31.31	31.86	50.1	14	1.012
sp|P35573|GDE_HUMAN	AGL	Glycogen debranching enzyme	71.93	72.29	47.4	35	1.012
sp|O00471|EXOC5_HUMAN	EXOC5	Exocyst complex component 5	19.09	21.44	39	13	1.012
sp|P78332|RBM6_HUMAN	RBM6	RNA-binding protein 6	9.26	12.18	23.7	9	1.012
sp|P62826|RAN_HUMAN	RAN	GTP-binding nuclear protein Ran	40.38	40.43	90.3	67	1.013
sp|P78310|CXAR_HUMAN	CXADR	Coxsackievirus and adenovirus receptor	20.96	21.09	55.6	14	1.013
sp|Q96JH7|VCIP1_HUMAN	VCPIP1	Deubiquitinating protein VCIP135	27	28.35	36.5	15	1.013
sp|Q9NQR4|NIT2_HUMAN	NIT2	Omega-amidase NIT2	23.88	27.45	71.7	19	1.013
sp|O60503|ADCY9_HUMAN	ADCY9	Adenylate cyclase type 9	8.26	8.91	17.2	7	1.013
sp|P16989|YBOX3_HUMAN	YBX3	Y-box-binding protein 3	12.67	24.6	71	26	1.013
sp|Q9BV79|MECR_HUMAN	MECR	Trans-2-enoyl-CoA reductase, mitochondrial	16	16.06	39.7	9	1.013
sp|Q96G74|OTUD5_HUMAN	OTUD5	OTU domain-containing protein 5	4	4.01	10.3	2	1.013
sp|P04066|FUCO_HUMAN	FUCA1	Tissue alpha-L-fucosidase	9.15	9.5	22.3	6	1.013
sp|P43003|EAA1_HUMAN	SLC1A3	Excitatory amino acid transporter 1	2	6.1	10.7	3	1.013
sp|Q5JRX3|PREP_HUMAN	PITRM1	Presequence protease, mitochondrial	64.54	65.82	51.1	42	1.013
sp|Q9Y6I3|EPN1_HUMAN	EPN1	Epsin-1	15.67	15.87	33.9	10	1.013
sp|Q16644|MAPK3_HUMAN	MAPKAPK3	MAP kinase-activated protein kinase 3	3.28	6.02	32.5	4	1.013
sp|Q8IX15|HOMEZ_HUMAN	HOMEZ	Homeobox and leucine zipper protein Homez	2.31	2.38	10.9	3	1.013
sp|Q9UL54|TAOK2_HUMAN	TAOK2	Serine/threonine-protein kinase TAO2	4.43	7.75	18.5	7	1.013
sp|Q96I51|WBS16_HUMAN	WBSCR16	Williams-Beuren syndrome chromosomal region 16 protein	16.59	16.93	42	9	1.013
sp|Q9UIV1|CNOT7_HUMAN	CNOT7	CCR4-NOT transcription complex subunit 7	2.41	2.51	31.6	3	1.013
sp|Q96ST3|SIN3A_HUMAN	SIN3A	Paired amphipathic helix protein Sin3a	50.71	51.42	36.5	31	1.014
sp|Q9Y3T9|NOC2L_HUMAN	NOC2L	Nucleolar complex protein 2 homolog	37.27	37.36	37.4	26	1.014
sp|Q2PZI1|D19L1_HUMAN	DPY19L1	Probable C-mannosyltransferase DPY19L1	5.34	5.56	19.7	4	1.014
sp|Q53EU6|GPAT3_HUMAN	AGPAT9	Glycerol-3-phosphate acyltransferase 3	10.14	10.5	33.2	7	1.014
sp|Q8NHG8|ZNRF2_HUMAN	ZNRF2	E3 ubiquitin-protein ligase ZNRF2	2.04	2.15	12.4	2	1.014
sp|P55854|SUMO3_HUMAN	SUMO3	Small ubiquitin-related modifier 3	2	4.49	61.2	7	1.014
sp|P48147|PPCE_HUMAN	PREP	Prolyl endopeptidase	59.92	60.83	72.4	37	1.014
sp|Q04724|TLE1_HUMAN	TLE1	Transducin-like enhancer protein 1	10.21	10.32	27.8	6	1.014
sp|Q9BVM2|DPCD_HUMAN	DPCD	Protein DPCD	5.48	5.58	34.5	3	1.014
sp|Q53H12|AGK_HUMAN	AGK	Acylglycerol kinase, mitochondrial	33.22	33.39	64.7	26	1.014
sp|Q8TBK2|SETD6_HUMAN	SETD6	N-lysine methyltransferase SETD6	1.63	1.82	16.3	2	1.014
sp|P29372|3MG_HUMAN	MPG	DNA-3-methyladenine glycosylase	14.56	14.59	50.7	9	1.015
sp|Q5T8P6|RBM26_HUMAN	RBM26	RNA-binding protein 26	37.66	38.64	32.3	20	1.015
sp|Q9Y2Z0|SGT1_HUMAN	SUGT1	Protein SGT1 homolog	32.54	32.71	74	24	1.015
sp|Q14160|SCRIB_HUMAN	SCRIB	Protein scribble homolog	62.24	64.32	45.9	42	1.015
sp|Q6IPR3|TYW3_HUMAN	TYW3	tRNA wybutosine-synthesizing protein 3 homolog	6.27	6.31	39.8	4	1.015
sp|P43005|EAA3_HUMAN	SLC1A1	Excitatory amino acid transporter 3	2	4.03	10.7	2	1.015
sp|Q96IZ6|MET2A_HUMAN	METTL2A	Methyltransferase-like protein 2A	4.08	4.11	23	2	1.016
sp|P62308|RUXG_HUMAN	SNRPG	Small nuclear ribonucleoprotein G	6.56	7.34	57.9	13	1.016
sp|O00499|BIN1_HUMAN	BIN1	Myc box-dependent-interacting protein 1	6.08	6.12	27.3	5	1.016
sp|O14727|APAF_HUMAN	APAF1	Apoptotic protease-activating factor 1	9.24	11.41	20.2	8	1.016
sp|Q13043|STK4_HUMAN	STK4	Serine/threonine-protein kinase 4	28.69	29.75	50.3	17	1.016

sp|Q9H1Z4|WDR13_HUMAN	WDR13	WD repeat-containing protein 13	2.64	2.87	13.4	3	1.016
sp|P21695|GPDA_HUMAN	GPD1	Glycerol-3-phosphate dehydrogenase [NAD(+)], cytoplasmic	16.78	21.81	61	14	1.016
sp|Q6P1N9|TATD1_HUMAN	TATDN1	Putative deoxyribonuclease TATDN1	13.21	13.44	53.2	7	1.016
sp|Q9H8H2|DDX31_HUMAN	DDX31	Probable ATP-dependent RNA helicase DDX31	19.59	19.73	23.6	11	1.016
sp|O95251|KAT7_HUMAN	KAT7	Histone acetyltransferase KAT7	23.53	23.76	41.9	13	1.016
sp|Q92692|PVRL2_HUMAN	PVRL2	Nectin-2	9.94	10.15	23.4	8	1.016
sp|P18887|XRCC1_HUMAN	XRCC1	DNA repair protein XRCC1	22.41	23.22	40.9	12	1.016
sp|Q8IZ69|TRM2A_HUMAN	TRMT2A	tRNA (uracil-5-)-methyltransferase homolog A	16.34	16.54	35.2	9	1.017
sp|Q96AX1|VP33A_HUMAN	VPS33A	Vacuolar protein sorting-associated protein 33A	14.37	14.63	40.9	9	1.017
sp|Q7L2J0|MEPCE_HUMAN	MEPCE	7SK snRNA methylphosphate capping enzyme	12.15	12.38	31.2	11	1.017
sp|O75843|AP1G2_HUMAN	AP1G2	AP-1 complex subunit gamma-like 2	14.64	17.51	25.1	10	1.017
sp|Q9H8Y8|GORS2_HUMAN	GORASP2	Golgi reassembly-stacking protein 2	25.82	26.15	56.4	33	1.017
sp|Q9NZQ3|SPN90_HUMAN	NCKIPSD	NCK-interacting protein with SH3 domain	15.09	15.21	18	9	1.017
sp|Q9UBU6|FA8A1_HUMAN	FAM8A1	Protein FAM8A1	12	12.02	33.2	6	1.017
sp|O15232|MATN3_HUMAN	MATN3	Matrilin-3	2.26	2.33	18.3	2	1.017
sp|Q9UJ14|GGT7_HUMAN	GGT7	Gamma-glutamyltransferase 7	8.01	8.06	18.4	5	1.017
sp|Q13332|PTPRS_HUMAN	PTPRS	Receptor-type tyrosine-protein phosphatase S	6.04	20.38	17.9	14	1.017
sp|Q86X29|LSR_HUMAN	LSR	Lipolysis-stimulated lipoprotein receptor	16.28	17.14	27.3	10	1.017
sp|Q9UL45|BL1S6_HUMAN	BLOC1S6	Biogenesis of lysosome-related organelles complex 1 subunit 6	2.01	2.01	32	2	1.017
sp|Q5T3I0|GPTC4_HUMAN	GPATCH4	G patch domain-containing protein 4	14.14	14.49	45.7	12	1.017
sp|O75131|CPNE3_HUMAN	CPNE3	Copine-3	49.19	50.25	63.5	38	1.017
sp|P42766|RL35_HUMAN	RPL35	60S ribosomal protein L35	11.14	18.02	68.3	17	1.017
sp|Q96AE4|FUBP1_HUMAN	FUBP1	Far upstream element-binding protein 1	40.93	54.05	68.9	53	1.018
sp|Q9NPH3|IL1AP_HUMAN	IL1RAP	Interleukin-1 receptor accessory protein	1.79	2.04	19	2	1.018
sp|Q08623|HDHD1_HUMAN	HDHD1	Pseudouridine-5'-phosphatase	6.22	6.35	40.8	5	1.018
sp|Q32P41|TRM5_HUMAN	TRMT5	tRNA (guanine(37)-N1)-methyltransferase	14.28	14.49	36.5	9	1.018
sp|Q9Y6X4|F169A_HUMAN	FAM169A	Soluble lamin-associated protein of 75 kDa	24.71	24.74	41.2	13	1.019
sp|Q9NYB0|TE2IP_HUMAN	TERF2IP	Telomeric repeat-binding factor 2-interacting protein 1	17.51	17.78	66.2	12	1.019
sp|P49736|MCM2_HUMAN	MCM2	DNA replication licensing factor MCM2	63.84	63.92	56.3	53	1.019
sp|P36405|ARL3_HUMAN	ARL3	ADP-ribosylation factor-like protein 3	19.37	19.77	71.4	11	1.019
sp|O00442|RTCA_HUMAN	RTCA	RNA 3'-terminal phosphate cyclase	19.37	19.46	49.2	14	1.019
sp|Q9NVT9|ARMC1_HUMAN	ARMC1	Armadillo repeat-containing protein 1	17.21	17.41	81.6	11	1.019
sp|Q9UI30|TR112_HUMAN	TRMT112	Multifunctional methyltransferase subunit TRM112-like protein	10.48	11.34	72.8	9	1.019
sp|P12270|TPR_HUMAN	TPR	Nucleoprotein TPR	199.32	202.59	60.3	142	1.019
sp|P18206|VINC_HUMAN	VCL	Vinculin	117.03	117.2	68.5	87	1.019
sp|P06746|DPOLB_HUMAN	POLB	DNA polymerase beta	7.47	8.74	43.6	8	1.019
sp|P09496|CLCA_HUMAN	CLTA	Clathrin light chain A	12.49	14.73	35.5	13	1.019
sp|O75909|CCNK_HUMAN	CCNK	Cyclin-K	10.15	10.55	28.6	8	1.019
sp|Q4G0J3|LARP7_HUMAN	LARP7	La-related protein 7	27.79	28.42	49.7	16	1.019
sp|Q9H6E4|CC134_HUMAN	CCDC134	Coiled-coil domain-containing protein 134	8.88	9.08	39.7	6	1.019
sp|Q9HBL7|PLRKT_HUMAN	PLGRKT	Plasminogen receptor (KT)	5.61	7.52	36.1	4	1.019
sp|Q9UJA5|TRM6_HUMAN	TRMT6	tRNA (adenine(58)-N(1))-methyltransferase non-catalytic subunit TRM6	21.78	22.17	43.9	13	1.019
sp|Q92793|CBP_HUMAN	CREBBP	CREB-binding protein	3.61	9.76	12.9	8	1.019
sp|P07148|FABPL_HUMAN	FABP1	Fatty acid-binding protein, liver	17.36	17.4	81.1	24	1.019
sp|Q9BRS2|RIOK1_HUMAN	RIOK1	Serine/threonine-protein kinase RIO1	15.25	15.61	36.8	9	1.019
sp|Q13425|SNTB2_HUMAN	SNTB2	Beta-2-syntrophin	33.52	33.71	47.4	18	1.019
sp|Q86XK2|FBX11_HUMAN	FBXO11	F-box only protein 11	4.23	4.28	7.4	2	1.019
sp|O75818|RPP40_HUMAN	RPP40	Ribonuclease P protein subunit p40	9.15	9.23	30	6	1.019
sp|Q9Y3S1|WNK2_HUMAN	WNK2	Serine/threonine-protein kinase WNK2	2.13	6.07	8.1	3	1.019

sp|A5D8V6|VP37C_HUMAN	VPS37C	Vacuolar protein sorting-associated protein 37C	4.67	4.72	17.8	3	1.019
sp|Q92542|NICA_HUMAN	NCSTN	Nicastrin	17.27	17.33	25.7	10	1.020
sp|Q9H3R5|CENPH_HUMAN	CENPH	Centromere protein H	6.79	9.23	54.7	11	1.020
sp|O15230|LAMA5_HUMAN	LAMA5	Laminin subunit alpha-5	65.93	67.41	22	36	1.020
sp|Q9Y4A5|TRRAP_HUMAN	TRRAP	Transformation/transcription domain-associated protein	71.95	74.1	27.1	38	1.020
sp|O43715|TRIA1_HUMAN	TRIAP1	TP53-regulated inhibitor of apoptosis 1	13.86	13.89	89.5	7	1.020
sp|P48634|PRC2A_HUMAN	PRRC2A	Protein PRRC2A	61.9	63.54	31.5	37	1.020
sp|Q13164|MK07_HUMAN	MAPK7	Mitogen-activated protein kinase 7	1.62	2.18	14.1	4	1.020
sp|Q99959|PKP2_HUMAN	PKP2	Plakophilin-2	34.36	38.59	38.3	20	1.020
sp|Q15398|DLGP5_HUMAN	DLGAP5	Disks large-associated protein 5	13.75	14.28	30.4	10	1.020
sp|Q9H999|PANK3_HUMAN	PANK3	Pantothenate kinase 3	2.01	2.74	25.1	2	1.021
sp|P84090|ERH_HUMAN	ERH	Enhancer of rudimentary homolog	11.97	12.08	64.4	13	1.021
sp|Q06265|EXOS9_HUMAN	EXOSC9	Exosome complex component RRP45	17.51	17.75	50.1	12	1.021
sp|Q86W92|LIPB1_HUMAN	PPFIBP1	Liprin-beta-1	16.98	17.64	33.2	10	1.021
sp|Q13049|TRI32_HUMAN	TRIM32	E3 ubiquitin-protein ligase TRIM32	9.1	9.87	21.8	6	1.021
sp|O00232|PSD12_HUMAN	PSMD12	26S proteasome non-ATPase regulatory subunit 12	52.13	56.31	66.5	42	1.021
sp|O94901|SUN1_HUMAN	SUN1	SUN domain-containing protein 1	24.8	25.11	37	14	1.021
sp|Q9NX07|TSAP1_HUMAN	TRNAU1AP	tRNA selenocysteine 1-associated protein 1	4.66	4.79	23.3	3	1.021
sp|Q8N9T8|KRI1_HUMAN	KRI1	Protein KRI1 homolog	20.91	23.92	38	12	1.021
sp|Q9H2U2|IPYR2_HUMAN	PPA2	Inorganic pyrophosphatase 2, mitochondrial	27.15	31.31	81.4	26	1.021
sp|O75717|WDHD1_HUMAN	WDHD1	WD repeat and HMG-box DNA-binding protein 1	41.78	42.16	36.9	23	1.021
sp|P18669|PGAM1_HUMAN	PGAM1	Phosphoglycerate mutase 1	40.95	40.97	81.1	53	1.021
sp|P27448|MARK3_HUMAN	MARK3	MAP/microtubule affinity-regulating kinase 3	2.12	12.19	27.4	6	1.022
sp|O14657|TOR1B_HUMAN	TOR1B	Torsin-1B	6.14	6.28	27.4	4	1.022
sp|Q5C9Z4|NOM1_HUMAN	NOM1	Nucleolar MIF4G domain-containing protein 1	16.24	18.82	31.4	12	1.022
sp|P04150|GCR_HUMAN	NR3C1	Glucocorticoid receptor	5.38	5.55	22.7	4	1.022
sp|O75586|MED6_HUMAN	MED6	Mediator of RNA polymerase II transcription subunit 6	6.13	6.21	37.8	5	1.022
sp|Q9H223|EHD4_HUMAN	EHD4	EH domain-containing protein 4	33.75	39.29	69.9	20	1.022
sp|P67812|SC11A_HUMAN	SEC11A	Signal peptidase complex catalytic subunit SEC11A	10.02	10.02	42.5	7	1.022
sp|P61244|MAX_HUMAN	MAX	Protein max	3.6	3.86	25	4	1.022
sp|O15379|HDAC3_HUMAN	HDAC3	Histone deacetylase 3	8.51	8.72	24.8	8	1.022
sp|Q08170|SRSF4_HUMAN	SRSF4	Serine/arginine-rich splicing factor 4	21.5	21.8	35.6	16	1.022
sp|P63096|GNAI1_HUMAN	GNAI1	Guanine nucleotide-binding protein G(i) subunit alpha-1	3.43	20.33	54.8	15	1.022
sp|Q8IYS1|P20D2_HUMAN	PM20D2	Peptidase M20 domain-containing protein 2	19.6	19.72	43.1	12	1.022
sp|Q92673|SORL_HUMAN	SORL1	Sortilin-related receptor	11.26	11.74	13.3	7	1.022
sp|Q9UFC0|LRWD1_HUMAN	LRWD1	Leucine-rich repeat and WD repeat-containing protein 1	18	18.11	31.4	11	1.022
sp|O14561|ACPM_HUMAN	NDUFAB1	Acyl carrier protein, mitochondrial	8.91	9.13	36.5	11	1.023
sp|O75177|CREST_HUMAN	SS18L1	Calcium-responsive transactivator	1.64	1.81	6.3	2	1.023
sp|Q5T1M5|FKB15_HUMAN	FKBP15	FK506-binding protein 15	21.12	22.25	38	13	1.023
sp|O43719|HTSF1_HUMAN	HTATSF1	HIV Tat-specific factor 1	23.45	24.11	35.9	18	1.023
sp|P62333|PRS10_HUMAN	PSMC6	26S protease regulatory subunit 10B	47.66	48.87	80.5	40	1.023
sp|P35998|PRS7_HUMAN	PSMC2	26S protease regulatory subunit 7	62.24	63.62	75.5	49	1.023
sp|Q15836|VAMP3_HUMAN	VAMP3	Vesicle-associated membrane protein 3	4	10	52	11	1.023
sp|Q9Y5A7|NUB1_HUMAN	NUB1	NEDD8 ultimate buster 1	10.51	12.8	33.7	8	1.023
sp|Q8IXU6|S35F2_HUMAN	SLC35F2	Solute carrier family 35 member F2	5.11	5.29	28.6	3	1.023
sp|P08133|ANXA6_HUMAN	ANXA6	Annexin A6	90.18	91.49	81.4	76	1.023
sp|P49593|PPM1F_HUMAN	PPM1F	Protein phosphatase 1F	26.28	26.78	61.5	17	1.023
sp|Q8TAD8|SNIP1_HUMAN	SNIP1	Smad nuclear-interacting protein 1	3.81	4.08	18.7	2	1.023
sp|Q16795|NDUA9_HUMAN	NDUFA9	NADH dehydrogenase [ubiquinone] 1 alpha subcomplex subunit 9, mitochondrial	21.93	22.34	51.7	15	1.023

sp|Q9UJ83|HACL1_HUMAN	HACL1	2-hydroxyacyl-CoA lyase 1	30.27	30.51	50.4	21	1.023
sp|Q96IF1|AJUBA_HUMAN	AJUBA	LIM domain-containing protein ajuba	14.57	15	43.3	13	1.023
sp|Q8WU90|ZC3HF_HUMAN	ZC3H15	Zinc finger CCCH domain-containing protein 15	36.6	37.19	63.2	20	1.024
sp|P13804|ETFA_HUMAN	ETFA	Electron transfer flavoprotein subunit alpha, mitochondrial	35.78	37.25	89.2	53	1.024
sp|Q92466|DDB2_HUMAN	DDB2	DNA damage-binding protein 2	9.98	10.66	36.8	6	1.024
sp|Q9NSK0|KLC4_HUMAN	KLC4	Kinesin light chain 4	31.3	40.09	47.7	25	1.024
sp|Q96BD8|SKA1_HUMAN	SKA1	Spindle and kinetochore-associated protein 1	4.03	4.06	29	3	1.024
sp|P07858|CATB_HUMAN	CTSB	Cathepsin B	18.98	19.11	46	16	1.024
sp|O75746|CMC1_HUMAN	SLC25A12	Calcium-binding mitochondrial carrier protein Aralar1	27.96	40.58	57.2	26	1.024
sp|Q5T200|ZC3HD_HUMAN	ZC3H13	Zinc finger CCCH domain-containing protein 13	10.81	11.22	14.1	10	1.025
sp|Q13131|AAPK1_HUMAN	PRKAA1	5'-AMP-activated protein kinase catalytic subunit alpha-1	23.68	23.96	39.7	12	1.025
sp|Q9BXW6|OSBL1_HUMAN	OSBPL1A	Oxysterol-binding protein-related protein 1	7.7	8.21	21.5	8	1.025
sp|P25311|ZA2G_HUMAN	AZGP1	Zinc-alpha-2-glycoprotein	2.5	2.58	28.5	2	1.025
sp|Q14966|ZN638_HUMAN	ZNF638	Zinc finger protein 638	48.09	48.46	35.2	28	1.025
sp|Q7Z6M1|RABEK_HUMAN	RABEPK	Rab9 effector protein with kelch motifs	16.07	16.23	37.1	10	1.025
sp|Q5T6F2|UBAP2_HUMAN	UBAP2	Ubiquitin-associated protein 2	28.37	28.43	24.7	18	1.025
sp|Q96B49|TOM6_HUMAN	TOMM6	Mitochondrial import receptor subunit TOM6 homolog	3.74	3.86	68.9	9	1.025
sp|Q96QE3|ATAD5_HUMAN	ATAD5	ATPase family AAA domain-containing protein 5	6.37	6.72	18.7	7	1.025
sp|P45983|MK08_HUMAN	MAPK8	Mitogen-activated protein kinase 8	2.8	7.31	28.1	4	1.025
sp|Q8IXM6|NRM_HUMAN	NRM	Nurim	5.38	5.51	26.3	4	1.025
sp|P40763|STAT3_HUMAN	STAT3	Signal transducer and activator of transcription 3	47.94	48.14	47	34	1.025
sp|Q14203|DCTN1_HUMAN	DCTN1	Dynactin subunit 1	86.49	89.57	61.6	58	1.026
sp|Q9P0S2|COX16_HUMAN	COX16	Cytochrome c oxidase assembly protein COX16 homolog, mitochondrial	2.63	2.67	31.1	2	1.026
sp|Q8NFD5|ARI1B_HUMAN	ARID1B	AT-rich interactive domain-containing protein 1B	9.13	12.39	13.1	9	1.026
sp|P17707|DCAM_HUMAN	AMD1	S-adenosylmethionine decarboxylase proenzyme	1.32	1.46	11.7	2	1.026
sp|P19367|HXK1_HUMAN	HK1	Hexokinase-1	56.01	74.6	54.2	46	1.026
sp|Q8IYI6|EXOC8_HUMAN	EXOC8	Exocyst complex component 8	23.02	23.32	45	15	1.026
sp|Q96ND0|F210A_HUMAN	FAM210A	Protein FAM210A	2.4	2.48	25	2	1.026
sp|Q13769|THOC5_HUMAN	THOC5	THO complex subunit 5 homolog	24.66	25.25	54.6	14	1.026
sp|Q6ZW49|PAXI1_HUMAN	PAXIP1	PAX-interacting protein 1	13.64	13.76	21.5	10	1.026
sp|Q99618|CDCA3_HUMAN	CDCA3	Cell division cycle-associated protein 3	6.05	6.07	47.4	4	1.026
sp|Q9Y4L1|HYOU1_HUMAN	HYOU1	Hypoxia up-regulated protein 1	94.68	94.82	67.9	101	1.027
sp|Q9BTY2|FUCO2_HUMAN	FUCA2	Plasma alpha-L-fucosidase	9.54	9.82	24.2	7	1.027
sp|Q00536|CDK16_HUMAN	CDK16	Cyclin-dependent kinase 16	13.62	19.24	43.6	10	1.027
sp|Q9NS93|TM7S3_HUMAN	TM7SF3	Transmembrane 7 superfamily member 3	5.48	5.55	16.7	3	1.027
sp|Q9UMZ2|SYNRG_HUMAN	SYNRG	Synergin gamma	7.75	8.06	15.5	4	1.027
sp|P56524|HDAC4_HUMAN	HDAC4	Histone deacetylase 4	5.29	5.46	14.1	5	1.027
sp|O43572|AKA10_HUMAN	AKAP10	A-kinase anchor protein 10, mitochondrial	4	4.38	12.8	3	1.027
sp|Q5VT52|RPRD2_HUMAN	RPRD2	Regulation of nuclear pre-mRNA domain-containing protein 2	46.13	46.85	33.5	28	1.027
sp|Q6PKG0|LARP1_HUMAN	LARP1	La-related protein 1	76.75	77.67	60.2	46	1.027
sp|Q9BVW5|TIPIN_HUMAN	TIPIN	TIMELESS-interacting protein	5.94	6.04	29.2	3	1.027
sp|Q15125|EBP_HUMAN	EBP	3-beta-hydroxysteroid-Delta(8),Delta(7)-isomerase	4.2	6.41	21.7	5	1.027
sp|Q5SGD2|PPM1L_HUMAN	PPM1L	Protein phosphatase 1L	2	3.18	13.9	3	1.027
sp|Q8N0Z8|PUSL1_HUMAN	PUSL1	tRNA pseudouridine synthase-like 1	3.23	3.35	9.6	3	1.027
sp|Q9NUQ9|FA49B_HUMAN	FAM49B	Protein FAM49B	28.14	28.16	54.9	25	1.027
sp|O75947|ATP5H_HUMAN	ATP5H	ATP synthase subunit d, mitochondrial	26.51	26.82	92.6	26	1.028
sp|P42696|RBM34_HUMAN	RBM34	RNA-binding protein 34	32.25	34.57	56.3	20	1.028
sp|P36404|ARL2_HUMAN	ARL2	ADP-ribosylation factor-like protein 2	13	14.01	62.5	14	1.028
sp|Q9GZP4|PITH1_HUMAN	PITHD1	PITH domain-containing protein 1	9.31	9.79	54	6	1.028

sp|Q05639|EF1A2_HUMAN	EEF1A2	Elongation factor 1-alpha 2	4.17	37.58	75.2	99	1.028
sp|Q709C8|VP13C_HUMAN	VPS13C	Vacuolar protein sorting-associated protein 13C	51.38	57.57	29.9	33	1.028
sp|Q99547|MPH6_HUMAN	MPHOSPH6	M-phase phosphoprotein 6	6.44	6.67	49.4	6	1.028
sp|O43432|IF4G3_HUMAN	EIF4G3	Eukaryotic translation initiation factor 4 gamma 3	15.39	33.41	30.1	18	1.028
sp|Q9BWM7|SFXN3_HUMAN	SFXN3	Sideroflexin-3	8.01	13.67	48.6	9	1.028
sp|Q9H6T0|ESRP2_HUMAN	ESRP2	Epithelial splicing regulatory protein 2	2.17	3.03	9.2	2	1.028
sp|Q9Y5B8|NDK7_HUMAN	NME7	Nucleoside diphosphate kinase 7	7.51	7.66	32.2	6	1.028
sp|Q14019|COTL1_HUMAN	COTL1	Coactosin-like protein	12.74	12.88	69.7	8	1.028
sp|P0C7V8|DC8L2_HUMAN	DCAF8L2	DDB1- and CUL4-associated factor 8-like protein 2	2	4.02	5.5	3	1.029
sp|Q6UB99|ANR11_HUMAN	ANKRD11	Ankyrin repeat domain-containing protein 11	5.72	6.18	12.8	4	1.029
sp|Q8WX93|PALLD_HUMAN	PALLD	Palladin	12.93	13.06	18.6	9	1.029
sp|Q96HR3|MED30_HUMAN	MED30	Mediator of RNA polymerase II transcription subunit 30	1.74	1.91	49.4	2	1.029
sp|Q6SZW1|SARM1_HUMAN	SARM1	Sterile alpha and TIR motif-containing protein 1	10.3	11.89	32.2	8	1.029
sp|Q71RC2|LARP4_HUMAN	LARP4	La-related protein 4	22.39	22.55	38.5	14	1.029
sp|P10515|ODP2_HUMAN	DLAT	Dihydrolipoyllysine-residue acetyltransferase component of pyruvate dehydrogenase complex	50.43	52.68	62.1	55	1.029
sp|P62072|TIM10_HUMAN	TIMM10	Mitochondrial import inner membrane translocase subunit Tim10	6	6.01	62.2	9	1.029
sp|Q9Y2L1|RRP44_HUMAN	DIS3	Exosome complex exonuclease RRP44	50.81	52.8	59.9	30	1.029
sp|P51116|FXR2_HUMAN	FXR2	Fragile X mental retardation syndrome-related protein 2	34.23	38.43	62	24	1.029
sp|Q8WVC0|LEO1_HUMAN	LEO1	RNA polymerase-associated protein LEO1	9.03	9.2	21.3	7	1.029
sp|P57088|TMM33_HUMAN	TMEM33	Transmembrane protein 33	15.77	16.65	51	17	1.029
sp|Q9BWL3|CA043_HUMAN	C1orf43	Uncharacterized protein C1orf43	2.01	2.09	22.9	2	1.029
sp|Q9NYY8|FAKD2_HUMAN	FASTKD2	FAST kinase domain-containing protein 2	31.62	32.1	44.9	18	1.029
sp|Q08257|QOR_HUMAN	CRYZ	Quinone oxidoreductase	27.87	28.62	72.3	24	1.029
sp|Q92558|WASF1_HUMAN	WASF1	Wiskott-Aldrich syndrome protein family member 1	10.79	10.86	27.4	6	1.030
sp|Q96CM8|ACSF2_HUMAN	ACSF2	Acyl-CoA synthetase family member 2, mitochondrial	29.09	29.74	42	17	1.030
sp|Q9BT09|CNPY3_HUMAN	CNPY3	Protein canopy homolog 3	12.29	12.41	43.2	8	1.030
sp|Q9UJJ9|GNPTG_HUMAN	GNPTG	N-acetylglucosamine-1-phosphotransferase subunit gamma	3.86	3.97	17.4	3	1.030
sp|Q9NVI7|ATD3A_HUMAN	ATAD3A	ATPase family AAA domain-containing protein 3A	21.85	57.2	60.4	41	1.030
sp|P55196|AFAD_HUMAN	MLLT4	Afadin	108.54	109.14	54.7	68	1.030
sp|Q9BTE1|DCTN5_HUMAN	DCTN5	Dynactin subunit 5	4.68	4.98	37.9	5	1.030
sp|P46939|UTRO_HUMAN	UTRN	Utrophin	168.99	172.67	49	102	1.030
sp|Q9NXR1|NDE1_HUMAN	NDE1	Nuclear distribution protein nudE homolog 1	13.08	13.19	48.3	8	1.030
sp|Q13625|ASPP2_HUMAN	TP53BP2	Apoptosis-stimulating of p53 protein 2	5.07	5.3	14.3	4	1.030
sp|P60174|TPIS_HUMAN	TPI1	Triosephosphate isomerase	62.6	62.71	87.4	137	1.031
sp|P17482|HXB9_HUMAN	HOXB9	Homeobox protein Hox-B9	8.06	8.06	23.6	4	1.031
sp|Q8NFA0|UBP32_HUMAN	USP32	Ubiquitin carboxyl-terminal hydrolase 32	6.99	7.5	15.8	5	1.031
sp|Q6L8Q7|PDE12_HUMAN	PDE12	2',5'-phosphodiesterase 12	35.8	36.46	51.7	23	1.031
sp|Q8IXK0|PHC2_HUMAN	PHC2	Polyhomeotic-like protein 2	12.21	12.37	15.3	7	1.031
sp|Q9H8V3|ECT2_HUMAN	ECT2	Protein ECT2	11.5	12.57	27.4	8	1.031
sp|O75822|EIF3J_HUMAN	EIF3J	Eukaryotic translation initiation factor 3 subunit J	26.38	27.8	72.5	24	1.031
sp|P63151|2ABA_HUMAN	PPP2R2A	Serine/threonine-protein phosphatase 2A 55 kDa regulatory subunit B alpha isoform	22.99	23.05	49	13	1.031
sp|Q9UJK0|TSR3_HUMAN	TSR3	Ribosome biogenesis protein TSR3 homolog	6.07	6.08	24	3	1.031
sp|Q6IN85|P4R3A_HUMAN	SMEK1	Serine/threonine-protein phosphatase 4 regulatory subunit 3A	23.95	25.37	32.7	15	1.031
sp|O75940|SPF30_HUMAN	SMNDC1	Survival of motor neuron-related-splicing factor 30	8.18	8.23	48.3	6	1.032
sp|Q96KA5|CLP1L_HUMAN	CLPTM1L	Cleft lip and palate transmembrane protein 1-like protein	8.52	8.57	21.8	5	1.032
sp|Q6ICB0|DESI1_HUMAN	DESI1	Desumoylating isopeptidase 1	3.11	3.29	15.5	3	1.032
sp|P16333|NCK1_HUMAN	NCK1	Cytoplasmic protein NCK1	11.61	14.33	42.4	10	1.032
sp|P13489|RINI_HUMAN	RNH1	Ribonuclease inhibitor	45.92	48.47	82.4	49	1.032
sp|P62899|RL31_HUMAN	RPL31	60S ribosomal protein L31	15.66	17.04	73.6	19	1.032

sp|Q96BS2|CHP3_HUMAN	TESC	Calcineurin B homologous protein 3	10.14	10.23	44.9	6	1.032
sp|Q8IUH4|ZDH13_HUMAN	ZDHHC13	Palmitoyltransferase ZDHHC13	3.89	4.75	14.6	6	1.032
sp|Q13190|STX5_HUMAN	STX5	Syntaxin-5	23.79	26.2	60	19	1.032
sp|Q53HC9|TSSC1_HUMAN	TSSC1	Protein TSSC1	5.91	6.2	18.1	4	1.032
sp|Q9NXW9|ALKB4_HUMAN	ALKBH4	Alpha-ketoglutarate-dependent dioxygenase alkB homolog 4	7.57	7.7	39.4	5	1.032
sp|Q9Y2Z4|SYYM_HUMAN	YARS2	Tyrosine--tRNA ligase, mitochondrial	39.36	39.47	63.3	27	1.032
sp|O95749|GGPPS_HUMAN	GGPS1	Geranylgeranyl pyrophosphate synthase	8.98	9.54	35.7	6	1.032
sp|O95926|SYF2_HUMAN	SYF2	Pre-mRNA-splicing factor SYF2	2.12	4.55	23.9	3	1.032
sp|O75368|SH3L1_HUMAN	SH3BGRL	SH3 domain-binding glutamic acid-rich-like protein	9.45	9.59	83.3	9	1.032
sp|Q7RTV0|PHF5A_HUMAN	PHF5A	PHD finger-like domain-containing protein 5A	12.46	12.59	82.7	7	1.032
sp|Q8NG68|TTL_HUMAN	TTL	Tubulin--tyrosine ligase	5.42	5.87	31	4	1.032
sp|P04920|B3A2_HUMAN	SLC4A2	Anion exchange protein 2	8.45	8.98	17.2	8	1.032
sp|Q96GA3|LTV1_HUMAN	LTV1	Protein LTV1 homolog	13.81	14.99	33.5	11	1.032
sp|P25398|RS12_HUMAN	RPS12	40S ribosomal protein S12	18.29	18.35	75.8	40	1.033
sp|P07225|PROS_HUMAN	PROS1	Vitamin K-dependent protein S	4.46	5.6	22.9	4	1.033
sp|Q9UBN7|HDAC6_HUMAN	HDAC6	Histone deacetylase 6	11.09	12	12.4	8	1.033
sp|Q5JS54|PSMG4_HUMAN	PSMG4	Proteasome assembly chaperone 4	4.01	4.01	43.9	2	1.033
sp|Q92575|UBXN4_HUMAN	UBXN4	UBX domain-containing protein 4	12.84	14.48	40.6	19	1.033
sp|Q9Y4Z0|LSM4_HUMAN	LSM4	U6 snRNA-associated Sm-like protein LSm4	12.45	12.52	61.9	9	1.033
sp|O43823|AKAP8_HUMAN	AKAP8	A-kinase anchor protein 8	17.92	18.21	33.1	11	1.033
sp|Q9HAC7|SUCHY_HUMAN	SUGCT	Succinate--hydroxymethylglutarate CoA-transferase	2	2.04	13.7	2	1.033
sp|Q9H1X3|DJC25_HUMAN	DNAJC25	DnaJ homolog subfamily C member 25	2.14	2.23	30.8	2	1.033
sp|Q15046|SYK_HUMAN	KARS	Lysine--tRNA ligase	63.94	65.02	70.9	48	1.033
sp|P49407|ARRB1_HUMAN	ARRB1	Beta-arrestin-1	18.53	18.73	36.6	10	1.034
sp|Q9Y2R0|COA3_HUMAN	COA3	Cytochrome c oxidase assembly factor 3 homolog, mitochondrial	8.57	8.68	58.5	6	1.034
sp|P68032|ACTC_HUMAN	ACTC1	Actin, alpha cardiac muscle 1	11.87	51.14	70.8	150	1.034
sp|Q9NWK9|BCD1_HUMAN	ZNHIT6	Box C/D snoRNA protein 1	2.44	2.55	37.9	3	1.034
sp|Q9Y5K8|VATD_HUMAN	ATP6V1D	V-type proton ATPase subunit D	13.61	15.21	49.8	11	1.034
sp|Q6NSJ2|PHLB3_HUMAN	PHLDB3	Pleckstrin homology-like domain family B member 3	3.59	4.3	24.2	4	1.034
sp|P32519|ELF1_HUMAN	ELF1	ETS-related transcription factor Elf-1	6.3	6.34	18.7	3	1.034
sp|Q5TKA1|LIN9_HUMAN	LIN9	Protein lin-9 homolog	4.01	4.06	22.7	3	1.034
sp|Q6P2H3|CEP85_HUMAN	CEP85	Centrosomal protein of 85 kDa	8.05	9.56	27.2	6	1.034
sp|Q8TBQ9|KISHA_HUMAN	TMEM167A	Protein kish-A	2.81	2.87	25	2	1.034
sp|P29692|EF1D_HUMAN	EEF1D	Elongation factor 1-delta	30.07	30.18	88.3	43	1.034
sp|Q969P0|IGSF8_HUMAN	IGSF8	Immunoglobulin superfamily member 8	13.11	13.27	27.4	8	1.034
sp|Q86Y39|NDUAB_HUMAN	NDUFA11	NADH dehydrogenase [ubiquinone] 1 alpha subcomplex subunit 11	12.79	12.89	61.7	10	1.035
sp|Q9BSH5|HDHD3_HUMAN	HDHD3	Haloacid dehalogenase-like hydrolase domain-containing protein 3	6.75	7.33	49.8	5	1.035
sp|Q9UH62|ARMX3_HUMAN	ARMCX3	Armadillo repeat-containing X-linked protein 3	12.29	12.62	35.6	10	1.035
sp|Q8TDW0|LRC8C_HUMAN	LRRC8C	Volume-regulated anion channel subunit LRRC8C	5.18	8.32	22.4	6	1.035
sp|O75886|STAM2_HUMAN	STAM2	Signal transducing adapter molecule 2	4.43	6.5	26.7	5	1.035
sp|P29597|TYK2_HUMAN	TYK2	Non-receptor tyrosine-protein kinase TYK2	4.66	6.86	16.2	5	1.035
sp|Q15057|ACAP2_HUMAN	ACAP2	Arf-GAP with coiled-coil, ANK repeat and PH domain-containing protein 2	9.32	9.74	30.9	6	1.035
sp|Q8N8N7|PTGR2_HUMAN	PTGR2	Prostaglandin reductase 2	6.28	6.39	30.2	4	1.035
sp|P07355|ANXA2_HUMAN	ANXA2	Annexin A2	47.98	48.21	83.2	30	1.035
sp|P07711|CATL1_HUMAN	CTSL	Cathepsin L1	6.92	7.14	34.5	7	1.035
sp|Q9UNQ2|DIM1_HUMAN	DIMT1	Probable dimethyladenosine transferase	20.31	22.21	58.5	13	1.035
sp|P56385|ATP5I_HUMAN	ATP5I	ATP synthase subunit e, mitochondrial	8.15	9.06	71	6	1.035
sp|Q96JI7|SPTCS_HUMAN	SPG11	Spatacsin	7.64	8	16.9	6	1.035
sp|Q9H8G2|CAAP1_HUMAN	CAAP1	Caspase activity and apoptosis inhibitor 1	15.16	16.88	46.5	10	1.035

sp|Q7Z5G4|GOGA7_HUMAN	GOLGA7	Golgin subfamily A member 7	3.88	3.97	38	2	1.035
sp|Q86UT6|NLRX1_HUMAN	NLRX1	NLR family member X1	16.12	16.87	23.6	9	1.035
sp|Q7Z7H8|RM10_HUMAN	MRPL10	39S ribosomal protein L10, mitochondrial	8.47	8.53	46.4	16	1.035
sp|Q9UGU0|TCF20_HUMAN	TCF20	Transcription factor 20	16.8	17.1	17.5	9	1.036
sp|Q9C0F1|CEP44_HUMAN	CEP44	Centrosomal protein of 44 kDa	4.12	4.13	17.7	2	1.036
sp|Q5JTD0|TJAP1_HUMAN	TJAP1	Tight junction-associated protein 1	4.51	4.74	16.7	3	1.036
sp|Q8TB72|PUM2_HUMAN	PUM2	Pumilio homolog 2	6.01	15.06	14.4	8	1.036
sp|Q9Y3D3|RT16_HUMAN	MRPS16	28S ribosomal protein S16, mitochondrial	4	4	21.9	2	1.036
sp|P82909|RT36_HUMAN	MRPS36	28S ribosomal protein S36, mitochondrial	10	10.01	64.1	5	1.036
sp|P38606|VATA_HUMAN	ATP6V1A	V-type proton ATPase catalytic subunit A	60.82	61.41	74.1	39	1.036
sp|P31751|AKT2_HUMAN	AKT2	RAC-beta serine/threonine-protein kinase	4.43	12.4	30.8	7	1.037
sp|Q92995|UBP13_HUMAN	USP13	Ubiquitin carboxyl-terminal hydrolase 13	10.33	12.69	24.6	8	1.037
sp|P17706|PTN2_HUMAN	PTPN2	Tyrosine-protein phosphatase non-receptor type 2	6.28	6.81	27.5	4	1.037
sp|Q9Y605|MOFA1_HUMAN	MRFAP1	MORF4 family-associated protein 1	2.03	2.04	42.5	2	1.037
sp|Q9UI43|MRM2_HUMAN	FTSJ2	rRNA methyltransferase 2, mitochondrial	4.99	5.28	26.8	4	1.037
sp|Q16718|NDUA5_HUMAN	NDUFA5	NADH dehydrogenase [ubiquinone] 1 alpha subcomplex subunit 5	12.1	12.65	85.3	9	1.037
sp|Q86W50|MET16_HUMAN	METTL16	Methyltransferase-like protein 16	9.95	10.42	30.4	8	1.037
sp|O95400|CD2B2_HUMAN	CD2BP2	CD2 antigen cytoplasmic tail-binding protein 2	18.7	18.86	59.2	13	1.037
sp|Q99571|P2RX4_HUMAN	P2RX4	P2X purinoceptor 4	1.96	2.1	19.3	3	1.037
sp|P53365|ARFP2_HUMAN	ARFIP2	Arfaptin-2	6.79	7.18	37.8	5	1.037
sp|Q8TCC3|RM30_HUMAN	MRPL30	39S ribosomal protein L30, mitochondrial	8.77	9.03	46	7	1.037
sp|P10619|PPGB_HUMAN	CTSA	Lysosomal protective protein	35.91	36.5	50.4	32	1.038
sp|Q2M2I8|AAK1_HUMAN	AAK1	AP2-associated protein kinase 1	24.4	24.65	34	16	1.038
sp|Q9UKZ1|CNO11_HUMAN	CNOT11	CCR4-NOT transcription complex subunit 11	2.64	2.68	19	2	1.038
sp|Q9H7D7|WDR26_HUMAN	WDR26	WD repeat-containing protein 26	26.36	26.59	38.4	15	1.038
sp|Q9Y5B0|CTDP1_HUMAN	CTDP1	RNA polymerase II subunit A C-terminal domain phosphatase	15.96	16.45	30.6	12	1.038
sp|Q96PY6|NEK1_HUMAN	NEK1	Serine/threonine-protein kinase Nek1	2.03	2.25	17.2	3	1.038
sp|Q9H0A8|COMD4_HUMAN	COMMD4	COMM domain-containing protein 4	6.23	6.29	70.9	4	1.038
sp|Q3MHD2|LSM12_HUMAN	LSM12	Protein LSM12 homolog	10.78	11.55	52.8	10	1.038
sp|Q99567|NUP88_HUMAN	NUP88	Nuclear pore complex protein Nup88	38.11	38.24	52.2	23	1.038
sp|O15162|PLS1_HUMAN	PLSCR1	Phospholipid scramblase 1	3.14	3.36	18.9	3	1.038
sp|Q9NV96|CC50A_HUMAN	TMEM30A	Cell cycle control protein 50A	10.75	11.06	31.9	8	1.038
sp|Q32MZ4|LRRF1_HUMAN	LRRFIP1	Leucine-rich repeat flightless-interacting protein 1	34.09	35.34	39.6	20	1.038
sp|P54764|EPHA4_HUMAN	EPHA4	Ephrin type-A receptor 4	4.06	6.15	14.7	3	1.038
sp|Q9BZ95|NSD3_HUMAN	WHSC1L1	Histone-lysine N-methyltransferase NSD3	7.64	7.85	18	4	1.038
sp|Q12996|CSTF3_HUMAN	CSTF3	Cleavage stimulation factor subunit 3	52.6	53.24	59.1	34	1.038
sp|Q96A35|RM24_HUMAN	MRPL24	39S ribosomal protein L24, mitochondrial	18.75	18.9	69	10	1.038
sp|Q96AB3|ISOC2_HUMAN	ISOC2	Isochorismatase domain-containing protein 2, mitochondrial	13.53	13.62	87.8	13	1.038
sp|Q96MF7|NSE2_HUMAN	NSMCE2	E3 SUMO-protein ligase NSE2	6.2	6.48	32.4	5	1.038
sp|Q7LBR1|CHM1B_HUMAN	CHMP1B	Charged multivesicular body protein 1b	4.37	4.68	26.1	4	1.039
sp|Q96L93|KI16B_HUMAN	KIF16B	Kinesin-like protein KIF16B	6.48	8.42	28.2	9	1.039
sp|O43491|E41L2_HUMAN	EPB41L2	Band 4.1-like protein 2	96.61	97.27	61	62	1.039
sp|Q6Y1H2|HACD2_HUMAN	HACD2	Very-long-chain (3R)-3-hydroxyacyl-CoA dehydratase 2	2	2.01	19.7	3	1.039
sp|Q8IYL3|CA174_HUMAN	C1orf174	UPF0688 protein C1orf174	3.41	3.5	31.7	2	1.039
sp|P13688|CEAM1_HUMAN	CEACAM1	Carcinoembryonic antigen-related cell adhesion molecule 1	4.91	4.98	17.7	9	1.039
sp|Q96SL1|DIRC2_HUMAN	DIRC2	Disrupted in renal carcinoma protein 2	2	2	9.4	2	1.039
sp|Q9NTM9|CUTC_HUMAN	CUTC	Copper homeostasis protein cutC homolog	7.66	7.83	45.4	6	1.039
sp|Q8TBB5|KLDC4_HUMAN	KLHDC4	Kelch domain-containing protein 4	6.98	7.07	26	4	1.039
sp|Q8IZ40|RCOR2_HUMAN	RCOR2	REST corepressor 2	6.01	9.38	22.2	6	1.040

sp|P29218|IMPA1_HUMAN	IMPA1	Inositol monophosphatase 1	22.34	22.46	45.1	12	1.040
sp|Q8N9B5|JMY_HUMAN	JMY	Junction-mediating and -regulatory protein	4.04	4.09	17.9	2	1.040
sp|Q6IEG0|SNR48_HUMAN	SNRNP48	U11/U12 small nuclear ribonucleoprotein 48 kDa protein	2.8	2.86	12.4	2	1.040
sp|Q9UBE0|SAE1_HUMAN	SAE1	SUMO-activating enzyme subunit 1	46.6	46.74	89.3	38	1.040
sp|Q9BY43|CHM4A_HUMAN	CHMP4A	Charged multivesicular body protein 4a	11.05	12.59	61.7	12	1.040
sp|Q9BWF3|RBM4_HUMAN	RBM4	RNA-binding protein 4	29.79	29.82	65.7	21	1.040
sp|O95825|QORL1_HUMAN	CRYZL1	Quinone oxidoreductase-like protein 1	10.35	10.62	38.4	7	1.040
sp|P46020|KPB1_HUMAN	PHKA1	Phosphorylase b kinase regulatory subunit alpha, skeletal muscle isoform	4.93	5.61	16.6	4	1.041
sp|Q12768|STRUM_HUMAN	KIAA0196	WASH complex subunit strumpellin	25.12	28.01	34.1	19	1.041
sp|Q8TBA6|GOGA5_HUMAN	GOLGA5	Golgin subfamily A member 5	36.26	36.63	57.1	22	1.041
sp|O94788|AL1A2_HUMAN	ALDH1A2	Retinal dehydrogenase 2	20.65	30.67	55.4	16	1.041
sp|Q9NQE9|HINT3_HUMAN	HINT3	Histidine triad nucleotide-binding protein 3	6.69	6.82	47.8	4	1.041
sp|O43920|NDUS5_HUMAN	NDUFS5	NADH dehydrogenase [ubiquinone] iron-sulfur protein 5	10.42	10.77	67.9	6	1.041
sp|P35232|PHB_HUMAN	PHB	Prohibitin	43.9	45.14	87.5	57	1.041
sp|Q8TAD4|ZNT5_HUMAN	SLC30A5	Zinc transporter 5	7.13	7.38	20	4	1.041
sp|Q96S52|PIGS_HUMAN	PIGS	GPI transamidase component PIG-S	19.74	20.08	29.4	12	1.041
sp|Q9UPP1|PHF8_HUMAN	PHF8	Histone lysine demethylase PHF8	19.87	22.28	25.9	13	1.041
sp|Q8IVD9|NUDC3_HUMAN	NUDCD3	NudC domain-containing protein 3	11.69	12.07	48.5	7	1.041
sp|O60547|GMDS_HUMAN	GMDS	GDP-mannose 4,6 dehydratase	33.28	35.12	59.7	25	1.041
sp|P25098|ARBK1_HUMAN	ADRBK1	Beta-adrenergic receptor kinase 1	18.88	19.24	43.1	11	1.041
sp|Q8IVW6|ARI3B_HUMAN	ARID3B	AT-rich interactive domain-containing protein 3B	6.52	8.67	29.4	5	1.041
sp|P45974|UBP5_HUMAN	USP5	Ubiquitin carboxyl-terminal hydrolase 5	50.75	51	60.5	38	1.042
sp|Q5JTW2|CEP78_HUMAN	CEP78	Centrosomal protein of 78 kDa	4.05	5.71	19.7	7	1.042
sp|Q709F0|ACD11_HUMAN	ACAD11	Acyl-CoA dehydrogenase family member 11	27.23	27.81	47.2	18	1.042
sp|P82970|HMGN5_HUMAN	HMGN5	High mobility group nucleosome-binding domain-containing protein 5	14.13	14.76	36.9	8	1.042
sp|Q6Y7W6|PERQ2_HUMAN	GIGYF2	PERQ amino acid-rich with GYF domain-containing protein 2	46.45	46.84	41.1	30	1.042
sp|Q96FK6|WDR89_HUMAN	WDR89	WD repeat-containing protein 89	8.14	8.25	29.2	4	1.042
sp|Q2KHR3|QSER1_HUMAN	QSER1	Glutamine and serine-rich protein 1	3.33	4.46	15	4	1.042
sp|Q9C0J8|WDR33_HUMAN	WDR33	pre-mRNA 3' end processing protein WDR33	40.9	43.37	31.8	24	1.042
sp|Q8IZD4|DCP1B_HUMAN	DCP1B	mRNA-decapping enzyme 1B	10.66	10.74	25.3	7	1.042
sp|O94855|SC24D_HUMAN	SEC24D	Protein transport protein Sec24D	34.15	36.9	40	22	1.042
sp|P11498|PYC_HUMAN	PC	Pyruvate carboxylase, mitochondrial	87.76	89.82	62.1	56	1.042
sp|Q14807|KIF22_HUMAN	KIF22	Kinesin-like protein KIF22	35.48	35.72	54.6	20	1.043
sp|Q8WVV9|HNRLL_HUMAN	HNRNPLL	Heterogeneous nuclear ribonucleoprotein L-like	34	37.29	60.7	23	1.043
sp|P26373|RL13_HUMAN	RPL13	60S ribosomal protein L13	20.37	22.28	59.7	27	1.043
sp|P56134|ATPK_HUMAN	ATP5J2	ATP synthase subunit f, mitochondrial	4.54	7.96	51.1	9	1.043
sp|P49411|EFTU_HUMAN	TUFM	Elongation factor Tu, mitochondrial	76.52	76.78	82.3	96	1.043
sp|Q8TAQ2|SMRC2_HUMAN	SMARCC2	SWI/SNF complex subunit SMARCC2	32.59	50.89	35	36	1.043
sp|Q9Y376|CAB39_HUMAN	CAB39	Calcium-binding protein 39	8.1	8.48	47.5	8	1.043
sp|Q08AM6|VAC14_HUMAN	VAC14	Protein VAC14 homolog	17.52	18.86	38.5	13	1.043
sp|Q14674|ESPL1_HUMAN	ESPL1	Separin	3.34	4.58	14.4	7	1.043
sp|Q9NUM4|T106B_HUMAN	TMEM106B	Transmembrane protein 106B	9.91	10.18	44.9	8	1.043
sp|Q9NYU1|UGGG2_HUMAN	UGGT2	UDP-glucose:glycoprotein glucosyltransferase 2	6.34	8.62	22.6	8	1.043
sp|Q8IWW6|RHG12_HUMAN	ARHGAP12	Rho GTPase-activating protein 12	5.79	6.03	19.6	3	1.043
sp|Q9BY49|PECR_HUMAN	PECR	Peroxisomal trans-2-enoyl-CoA reductase	18.7	18.84	56.8	11	1.044
sp|Q9UBT2|SAE2_HUMAN	UBA2	SUMO-activating enzyme subunit 2	60.58	60.92	71.6	48	1.044
sp|P56937|DHB7_HUMAN	HSD17B7	3-keto-steroid reductase	8.08	8.97	39.3	10	1.044
sp|P54198|HIRA_HUMAN	HIRA	Protein HIRA	9.84	12.41	23.8	11	1.044
sp|Q8WYH8|ING5_HUMAN	ING5	Inhibitor of growth protein 5	2.83	2.96	29.6	2	1.044

sp|O14910|LIN7A_HUMAN	LIN7A	Protein lin-7 homolog A	3.49	11.97	56.2	11	1.044
sp|Q9BSY4|CHCH5_HUMAN	CHCHD5	Coiled-coil-helix-coiled-coil-helix domain-containing protein 5	6	6	41.8	3	1.044
sp|Q92506|DHB8_HUMAN	HSD17B8	Estradiol 17-beta-dehydrogenase 8	9.65	9.76	46.4	5	1.044
sp|Q86UE4|LYRIC_HUMAN	MTDH	Protein LYRIC	33.22	33.52	59.3	17	1.044
sp|P60510|PP4C_HUMAN	PPP4C	Serine/threonine-protein phosphatase 4 catalytic subunit	14.43	19.08	69.4	17	1.045
sp|Q6DD87|ZN787_HUMAN	ZNF787	Zinc finger protein 787	5.82	5.88	24.3	3	1.045
sp|Q9BQD3|KXDL1_HUMAN	KXD1	KxDL motif-containing protein 1	3.17	3.24	33.5	2	1.045
sp|Q9NVA1|UQCC1_HUMAN	UQCC1	Ubiquinol-cytochrome-c reductase complex assembly factor 1	12.14	12.16	36.1	6	1.045
sp|Q15119|PDK2_HUMAN	PDK2	[Pyruvate dehydrogenase (acetyl-transferring)] kinase isozyme 2, mitochondrial	3.65	5.97	31.9	4	1.045
sp|Q5SW79|CE170_HUMAN	CEP170	Centrosomal protein of 170 kDa	50.93	52.59	37.1	29	1.046
sp|Q9UBI1|COMD3_HUMAN	COMMD3	COMM domain-containing protein 3	7.61	7.78	57.4	5	1.046
sp|Q9UHB9|SRP68_HUMAN	SRP68	Signal recognition particle subunit SRP68	60.65	60.74	67.9	43	1.046
sp|Q9Y535|RPC8_HUMAN	POLR3H	DNA-directed RNA polymerase III subunit RPC8	6	6.01	34.8	3	1.046
sp|O95563|MPC2_HUMAN	MPC2	Mitochondrial pyruvate carrier 2	8.42	8.46	38.6	5	1.046
sp|Q96MU7|YTDC1_HUMAN	YTHDC1	YTH domain-containing protein 1	9.8	12.36	26.4	9	1.046
sp|Q8TEU7|RPGF6_HUMAN	RAPGEF6	Rap guanine nucleotide exchange factor 6	2.12	5.88	17.2	6	1.046
sp|P41743|KPCI_HUMAN	PRKCI	Protein kinase C iota type	16.02	16.55	41.4	9	1.046
sp|O75486|SUPT3_HUMAN	SUPT3H	Transcription initiation protein SPT3 homolog	5.96	6.03	21.8	4	1.046
sp|P61978|HNRPK_HUMAN	HNRNPK	Heterogeneous nuclear ribonucleoprotein K	67.56	68.67	84.9	142	1.046
sp|Q8TB36|GDAP1_HUMAN	GDAP1	Ganglioside-induced differentiation-associated protein 1	4.7	5.24	41.9	5	1.046
sp|Q96GP6|SREC2_HUMAN	SCARF2	Scavenger receptor class F member 2	3.41	3.68	12.6	3	1.046
sp|P42858|HD_HUMAN	HTT	Huntingtin	56.57	59.3	24.8	32	1.046
sp|Q9NZE8|RM35_HUMAN	MRPL35	39S ribosomal protein L35, mitochondrial	3.31	3.43	37.2	2	1.046
sp|Q9UL63|MKLN1_HUMAN	MKLN1	Muskelin	24.14	24.38	33.6	15	1.046
sp|Q9BX40|LS14B_HUMAN	LSM14B	Protein LSM14 homolog B	15.18	16.31	45.2	12	1.046
sp|Q8WWK9|CKAP2_HUMAN	CKAP2	Cytoskeleton-associated protein 2	13.43	13.76	34.3	9	1.046
sp|Q9Y484|WIPI4_HUMAN	WDR45	WD repeat domain phosphoinositide-interacting protein 4	6.04	6.13	29.4	3	1.047
sp|Q96P47|AGAP3_HUMAN	AGAP3	Arf-GAP with GTPase, ANK repeat and PH domain-containing protein 3	6.98	11.85	19.3	8	1.047
sp|Q9BRQ6|MIC25_HUMAN	CHCHD6	MIC complex subunit MIC25	7.16	7.34	58.7	5	1.047
sp|P49821|NDUV1_HUMAN	NDUFV1	NADH dehydrogenase [ubiquinone] flavoprotein 1, mitochondrial	39.31	40.99	69.4	36	1.047
sp|Q12948|FOXC1_HUMAN	FOXC1	Forkhead box protein C1	11.82	11.99	29.1	6	1.048
sp|Q9H3F6|BACD3_HUMAN	KCTD10	BTB/POZ domain-containing adapter for CUL3-mediated RhoA degradation protein 3	2.41	2.55	18.2	5	1.048
sp|O43772|MCAT_HUMAN	SLC25A20	Mitochondrial carnitine/acylcarnitine carrier protein	19.76	19.86	71.4	11	1.048
sp|Q8NFH4|NUP37_HUMAN	NUP37	Nucleoporin Nup37	19.75	20.41	44.2	14	1.048
sp|Q04917|1433F_HUMAN	YWHAH	14-3-3 protein eta	22.86	33.79	75.2	38	1.048
sp|Q6P3W7|SCYL2_HUMAN	SCYL2	SCY1-like protein 2	13.86	14.43	27	8	1.048
sp|Q9BUG6|ZSA5A_HUMAN	ZSCAN5A	Zinc finger and SCAN domain-containing protein 5A	1.47	1.61	13.1	3	1.048
sp|Q9BSJ2|GCP2_HUMAN	TUBGCP2	Gamma-tubulin complex component 2	37.73	38.3	43.9	22	1.048
sp|P50895|BCAM_HUMAN	BCAM	Basal cell adhesion molecule	29.97	30.02	44	17	1.048
sp|Q9H773|DCTP1_HUMAN	DCTPP1	dCTP pyrophosphatase 1	9.37	9.42	36.5	5	1.048
sp|O43809|CPSF5_HUMAN	NUDT21	Cleavage and polyadenylation specificity factor subunit 5	27.7	27.78	77.5	18	1.048
sp|Q9HAF1|EAF6_HUMAN	MEAF6	Chromatin modification-related protein MEAF6	8.55	8.66	49.2	5	1.048
sp|P36955|PEDF_HUMAN	SERPINF1	Pigment epithelium-derived factor	12.06	13.69	28	7	1.048
sp|P55199|ELL_HUMAN	ELL	RNA polymerase II elongation factor ELL	2.4	2.5	16.1	2	1.048
sp|Q07617|SPAG1_HUMAN	SPAG1	Sperm-associated antigen 1	4.01	6.12	20.6	4	1.048
sp|P26038|MOES_HUMAN	MSN	Moesin	43.46	66.63	66.2	47	1.048
sp|P25786|PSA1_HUMAN	PSMA1	Proteasome subunit alpha type-1	35.85	36.6	84.4	30	1.048
sp|Q8NFF5|FAD1_HUMAN	FLAD1	FAD synthase	21	23	48.7	13	1.048
sp|Q9UHY8|FEZ2_HUMAN	FEZ2	Fasciculation and elongation protein zeta-2	4.88	4.96	24.4	3	1.049

sp|Q9Y265|RUVB1_HUMAN	RUVBL1	RuvB-like 1	44.65	45.85	74.3	40	1.049
sp|O14569|C56D2_HUMAN	CYB561D2	Cytochrome b561 domain-containing protein 2	1.66	1.77	11.3	2	1.049
sp|P29374|ARI4A_HUMAN	ARID4A	AT-rich interactive domain-containing protein 4A	3.59	4.86	16.3	5	1.049
sp|Q9BXS4|TMM59_HUMAN	TMEM59	Transmembrane protein 59	4	4	9.9	2	1.049
sp|O43166|SI1L1_HUMAN	SIPA1L1	Signal-induced proliferation-associated 1-like protein 1	2.2	4.45	10.3	3	1.049
sp|O43592|XPOT_HUMAN	XPOT	Exportin-T	43.23	45.36	50.6	28	1.049
sp|Q9ULZ3|ASC_HUMAN	PYCARD	Apoptosis-associated speck-like protein containing a CARD	3.51	3.6	20	2	1.049
sp|Q09161|NCBP1_HUMAN	NCBP1	Nuclear cap-binding protein subunit 1	24.95	27.38	41.8	17	1.049
sp|Q9H6U8|ALG9_HUMAN	ALG9	Alpha-1,2-mannosyltransferase ALG9	3.32	3.79	13.4	3	1.049
sp|Q9UBB4|ATX10_HUMAN	ATXN10	Ataxin-10	35.68	38.53	56	30	1.050
sp|Q9UJ41|RABX5_HUMAN	RABGEF1	Rab5 GDP/GTP exchange factor	12.29	12.64	35.5	8	1.050
sp|Q9Y3C6|PPIL1_HUMAN	PPIL1	Peptidyl-prolyl cis-trans isomerase-like 1	11.78	11.86	55.4	10	1.050
sp|Q9UIQ6|LCAP_HUMAN	LNPEP	Leucyl-cystinyl aminopeptidase	17.18	18.23	24.9	10	1.050
sp|P21127|CD11B_HUMAN	CDK11B	Cyclin-dependent kinase 11B	14.52	14.88	29.8	10	1.050
sp|O94906|PRP6_HUMAN	PRPF6	Pre-mRNA-processing factor 6	70.59	71.74	63.7	43	1.050
sp|Q99459|CDC5L_HUMAN	CDC5L	Cell division cycle 5-like protein	60.17	61.85	67	49	1.050
sp|Q9H1I8|ASCC2_HUMAN	ASCC2	Activating signal cointegrator 1 complex subunit 2	14.8	14.96	27.1	13	1.051
sp|Q9UG01|IF172_HUMAN	IFT172	Intraflagellar transport protein 172 homolog	2.03	2.1	16.9	3	1.051
sp|O14683|P5I11_HUMAN	TP53I11	Tumor protein p53-inducible protein 11	4.11	4.13	17.5	2	1.051
sp|Q9ULM6|CNOT6_HUMAN	CNOT6	CCR4-NOT transcription complex subunit 6	9.07	9.16	28	5	1.051
sp|Q8WUM0|NU133_HUMAN	NUP133	Nuclear pore complex protein Nup133	82.01	83.86	59.6	48	1.051
sp|Q96HC4|PDLI5_HUMAN	PDLIM5	PDZ and LIM domain protein 5	27.74	28.12	47.3	22	1.051
sp|Q7Z6K5|ARPIN_HUMAN	ARPIN	Arpin	5.19	5.33	31	4	1.051
sp|P01034|CYTC_HUMAN	CST3	Cystatin-C	10	10	45.9	5	1.052
sp|P58557|YBEY_HUMAN	YBEY	Putative ribonuclease	4.25	4.89	24	4	1.052
sp|Q9H5H4|ZN768_HUMAN	ZNF768	Zinc finger protein 768	12.13	13.01	19.6	7	1.052
sp|P51946|CCNH_HUMAN	CCNH	Cyclin-H	6.27	6.32	35.9	4	1.052
sp|P42684|ABL2_HUMAN	ABL2	Abelson tyrosine-protein kinase 2	7.11	7.36	21	5	1.052
sp|P80303|NUCB2_HUMAN	NUCB2	Nucleobindin-2	29.92	31.18	69.5	22	1.052
sp|P31150|GDIA_HUMAN	GDI1	Rab GDP dissociation inhibitor alpha	28.41	52.92	83.2	57	1.052
sp|Q9NYP9|MS18A_HUMAN	MIS18A	Protein Mis18-alpha	4.48	4.61	36.9	4	1.052
sp|Q96EA4|SPDLY_HUMAN	SPDL1	Protein Spindly	15.31	15.78	46.5	8	1.052
sp|P51398|RT29_HUMAN	DAP3	28S ribosomal protein S29, mitochondrial	40.17	40.38	62.3	27	1.052
sp|Q9HCN3|TMM8A_HUMAN	TMEM8A	Transmembrane protein 8A	2	2.05	6.5	3	1.052
sp|Q9H0U4|RAB1B_HUMAN	RAB1B	Ras-related protein Rab-1B	7.58	31.09	88.1	33	1.053
sp|Q02978|M2OM_HUMAN	SLC25A11	Mitochondrial 2-oxoglutarate/malate carrier protein	38.51	38.67	73.6	29	1.053
sp|Q7L014|DDX46_HUMAN	DDX46	Probable ATP-dependent RNA helicase DDX46	77.91	79.88	57.1	48	1.053
sp|O60610|DIAP1_HUMAN	DIAPH1	Protein diaphanous homolog 1	67.75	70.32	48.9	40	1.053
sp|O75462|CRLF1_HUMAN	CRLF1	Cytokine receptor-like factor 1	4.08	4.1	24.4	2	1.053
sp|Q9HD47|MOG1_HUMAN	RANGRF	Ran guanine nucleotide release factor	1.89	2.05	18.3	2	1.053
sp|Q9NRA8|4ET_HUMAN	EIF4ENIF1	Eukaryotic translation initiation factor 4E transporter	5.29	5.53	18.9	4	1.053
sp|Q96EK5|KBP_HUMAN	KIF1BP	KIF1-binding protein	27.37	27.81	52.5	15	1.053
sp|Q9NX63|MIC19_HUMAN	CHCHD3	MIC complex subunit MIC19	18.14	18.25	67	11	1.053
sp|P27708|PYR1_HUMAN	CAD	CAD protein	144.43	144.46	60.9	96	1.054
sp|O00186|STXB3_HUMAN	STXBP3	Syntaxin-binding protein 3	41.6	42.93	64.5	25	1.054
sp|Q8TD19|NEK9_HUMAN	NEK9	Serine/threonine-protein kinase Nek9	27.03	29.9	42	18	1.054
sp|P21397|AOFA_HUMAN	MAOA	Amine oxidase [flavin-containing] A	5.45	11.38	26.2	6	1.054
sp|Q07820|MCL1_HUMAN	MCL1	Induced myeloid leukemia cell differentiation protein Mcl-1	4.98	5.05	25.4	3	1.054
sp|Q96SK2|TM209_HUMAN	TMEM209	Transmembrane protein 209	12.46	12.75	28.3	8	1.054

sp|O96008|TOM40_HUMAN	TOMM40	Mitochondrial import receptor subunit TOM40 homolog	20.78	21.4	62.1	33	1.054
sp|Q8N6T7|SIR6_HUMAN	SIRT6	NAD-dependent protein deacetylase sirtuin-6	4.22	4.24	20.9	2	1.054
sp|Q8WUQ7|CATIN_HUMAN	CACTIN	Cactin	10.71	10.86	23.4	8	1.054
sp|P08579|RU2B_HUMAN	SNRPB2	U2 small nuclear ribonucleoprotein B''	16.66	21.94	66.2	15	1.054
sp|P56377|AP1S2_HUMAN	AP1S2	AP-1 complex subunit sigma-2	6.03	8.13	59.9	4	1.054
sp|Q96PU8|QKI_HUMAN	QKI	Protein quaking	31.02	32.04	61.3	19	1.054
sp|O75027|ABCB7_HUMAN	ABCB7	ATP-binding cassette sub-family B member 7, mitochondrial	28.76	31.18	39.4	19	1.054
sp|Q8TEP8|CE192_HUMAN	CEP192	Centrosomal protein of 192 kDa	1.73	2.66	13.8	2	1.054
sp|O75964|ATP5L_HUMAN	ATP5L	ATP synthase subunit g, mitochondrial	11.79	14.68	80.6	14	1.054
sp|Q8N668|COMD1_HUMAN	COMMD1	COMM domain-containing protein 1	6.98	7.32	56.8	7	1.054
sp|Q96KP1|EXOC2_HUMAN	EXOC2	Exocyst complex component 2	19.22	19.75	35.1	10	1.054
sp|P27695|APEX1_HUMAN	APEX1	DNA-(apurinic or apyrimidinic site) lyase	42.72	42.84	75.5	33	1.054
sp|Q5W0Z9|ZDH20_HUMAN	ZDHHC20	Probable palmitoyltransferase ZDHHC20	3.36	3.44	27.7	2	1.054
sp|Q9Y2V7|COG6_HUMAN	COG6	Conserved oligomeric Golgi complex subunit 6	14.44	15.86	33.6	12	1.054
sp|O95210|STBD1_HUMAN	STBD1	Starch-binding domain-containing protein 1	4.54	4.58	17.9	3	1.054
sp|P35611|ADDA_HUMAN	ADD1	Alpha-adducin	40.33	40.89	50.2	25	1.054
sp|Q99828|CIB1_HUMAN	CIB1	Calcium and integrin-binding protein 1	3.86	3.93	31.4	2	1.054
sp|P62191|PRS4_HUMAN	PSMC1	26S protease regulatory subunit 4	40.89	45.23	71.1	38	1.055
sp|Q86WU2|LDHD_HUMAN	LDHD	Probable D-lactate dehydrogenase, mitochondrial	8	8	15	4	1.055
sp|P14314|GLU2B_HUMAN	PRKCSH	Glucosidase 2 subunit beta	46.21	46.73	71	35	1.055
sp|O94817|ATG12_HUMAN	ATG12	Ubiquitin-like protein ATG12	2	2.04	20.7	2	1.055
sp|Q15386|UBE3C_HUMAN	UBE3C	Ubiquitin-protein ligase E3C	25.28	27.54	31.8	15	1.055
sp|Q8N1G0|ZN687_HUMAN	ZNF687	Zinc finger protein 687	15.16	17.22	23.9	12	1.055
sp|O15439|MRP4_HUMAN	ABCC4	Multidrug resistance-associated protein 4	29.94	30.11	28.2	17	1.055
sp|Q8TEM1|PO210_HUMAN	NUP210	Nuclear pore membrane glycoprotein 210	69.81	70.49	33.9	50	1.055
sp|Q9UKL6|PPCT_HUMAN	PCTP	Phosphatidylcholine transfer protein	1.78	1.94	34.1	2	1.055
sp|Q8WVQ1|CANT1_HUMAN	CANT1	Soluble calcium-activated nucleotidase 1	16.3	16.36	35.2	8	1.055
sp|Q9UJU6|DBNL_HUMAN	DBNL	Drebrin-like protein	22.58	22.72	49.1	20	1.055
sp|P0CAP2|GRL1A_HUMAN	POLR2M	DNA-directed RNA polymerase II subunit GRINL1A	2.54	2.6	31	2	1.056
sp|Q96B36|AKTS1_HUMAN	AKT1S1	Proline-rich AKT1 substrate 1	9.8	9.85	24.2	5	1.056
sp|O75771|RA51D_HUMAN	RAD51D	DNA repair protein RAD51 homolog 4	4	6	19.2	3	1.056
sp|Q5BKZ1|ZN326_HUMAN	ZNF326	DBIRD complex subunit ZNF326	28.39	29.86	38.5	24	1.056
sp|Q12931|TRAP1_HUMAN	TRAP1	Heat shock protein 75 kDa, mitochondrial	91.44	94.38	73.6	114	1.056
sp|Q5W0V3|F16B1_HUMAN	FAM160B1	Protein FAM160B1	3.49	4.21	17	3	1.056
sp|Q9Y2Z2|MTO1_HUMAN	MTO1	Protein MTO1 homolog, mitochondrial	9.96	10.34	28.9	8	1.056
sp|Q9NPL8|TIDC1_HUMAN	TIMMDC1	Complex I assembly factor TIMMDC1, mitochondrial	16.88	16.96	49.5	11	1.057
sp|Q969H8|MYDGF_HUMAN	MYDGF	Myeloid-derived growth factor	9.37	9.56	34.1	6	1.057
sp|Q9Y223|GLCNE_HUMAN	GNE	Bifunctional UDP-N-acetylglucosamine 2-epimerase/N-acetylmannosamine kinase	25.84	25.94	37.5	15	1.057
sp|Q9UPW6|SATB2_HUMAN	SATB2	DNA-binding protein SATB2	15.01	15.54	28.7	13	1.057
sp|P62760|VISL1_HUMAN	VSNL1	Visinin-like protein 1	14.35	14.37	67	7	1.057
sp|Q96II8|LRCH3_HUMAN	LRCH3	Leucine-rich repeat and calponin homology domain-containing protein 3	10.48	11.04	23.4	11	1.057
sp|Q86Y07|VRK2_HUMAN	VRK2	Serine/threonine-protein kinase VRK2	8.13	8.26	22.1	4	1.057
sp|P09211|GSTP1_HUMAN	GSTP1	Glutathione S-transferase P	24.93	25.09	73.8	55	1.057
sp|Q8N3P4|VPS8_HUMAN	VPS8	Vacuolar protein sorting-associated protein 8 homolog	4.91	5.1	12.8	5	1.057
sp|Q96TC7|RMD3_HUMAN	RMDN3	Regulator of microtubule dynamics protein 3	20.83	23.11	46.2	14	1.058
sp|Q96EY8|MMAB_HUMAN	MMAB	Cob(I)yrinic acid a,c-diamide adenosyltransferase, mitochondrial	12.15	12.18	56.8	8	1.058
sp|P09669|COX6C_HUMAN	COX6C	Cytochrome c oxidase subunit 6C	11.63	11.8	52	10	1.058
sp|Q9H008|LHPP_HUMAN	LHPP	Phospholysine phosphohistidine inorganic pyrophosphate phosphatase	6.61	6.64	36.7	4	1.058
sp|Q9UKV5|AMFR_HUMAN	AMFR	E3 ubiquitin-protein ligase AMFR	16.2	16.7	32.5	11	1.058

sp|P11586|C1TC_HUMAN	MTHFD1	C-1-tetrahydrofolate synthase, cytoplasmic	112.94	114.71	83.3	85	1.058
sp|Q15056|IF4H_HUMAN	EIF4H	Eukaryotic translation initiation factor 4H	24.7	25.17	61.7	26	1.058
sp|P05154|IPSP_HUMAN	SERPINA5	Plasma serine protease inhibitor	10.46	12.15	26.1	6	1.058
sp|P49427|UB2R1_HUMAN	CDC34	Ubiquitin-conjugating enzyme E2 R1	4.12	5.48	30.1	5	1.058
sp|Q5RKV6|EXOS6_HUMAN	EXOSC6	Exosome complex component MTR3	19.32	19.4	39	11	1.058
sp|P41235|HNF4A_HUMAN	HNF4A	Hepatocyte nuclear factor 4-alpha	11.83	11.9	33.8	7	1.058
sp|O15270|SPTC2_HUMAN	SPTLC2	Serine palmitoyltransferase 2	32.07	34.07	53.2	20	1.059
sp|P08559|ODPA_HUMAN	PDHA1	Pyruvate dehydrogenase E1 component subunit alpha, somatic form, mitochondrial	39.1	39.17	65.4	35	1.059
sp|Q8WYQ3|CHC10_HUMAN	CHCHD10	Coiled-coil-helix-coiled-coil-helix domain-containing protein 10, mitochondrial	1.96	2	16.2	2	1.059
sp|Q9UFW8|CGBP1_HUMAN	CGGBP1	CGG triplet repeat-binding protein 1	13.08	13.38	71.9	9	1.059
sp|O15438|MRP3_HUMAN	ABCC3	Canalicular multispecific organic anion transporter 2	2.03	2.17	9.8	2	1.059
sp|P22059|OSBP1_HUMAN	OSBP	Oxysterol-binding protein 1	38.35	42.18	45.1	27	1.059
sp|Q93100|KPBB_HUMAN	PHKB	Phosphorylase b kinase regulatory subunit beta	11.56	12.16	26.7	12	1.059
sp|P18858|DNLI1_HUMAN	LIG1	DNA ligase 1	44.84	45.47	47	22	1.059
sp|Q96K19|RN170_HUMAN	RNF170	E3 ubiquitin-protein ligase RNF170	2.75	2.82	18.6	2	1.060
sp|Q8WUA7|TB22A_HUMAN	TBC1D22A	TBC1 domain family member 22A	4.47	4.53	14.7	4	1.060
sp|Q96MG7|MAGG1_HUMAN	NDNL2	Melanoma-associated antigen G1	6.32	6.48	29.9	3	1.060
sp|O75391|SPAG7_HUMAN	SPAG7	Sperm-associated antigen 7	5.72	5.85	50.2	5	1.060
sp|Q6ZNA5|FRRS1_HUMAN	FRRS1	Ferric-chelate reductase 1	6.03	6.05	16.4	3	1.061
sp|P09132|SRP19_HUMAN	SRP19	Signal recognition particle 19 kDa protein	14.35	14.43	63.2	15	1.061
sp|Q16637|SMN_HUMAN	SMN1	Survival motor neuron protein	16.64	16.68	44.6	11	1.061
sp|Q92766|RREB1_HUMAN	RREB1	Ras-responsive element-binding protein 1	19.98	20.2	24.4	10	1.061
sp|P52815|RM12_HUMAN	MRPL12	39S ribosomal protein L12, mitochondrial	13.4	13.77	60.6	22	1.061
sp|Q96CT7|CC124_HUMAN	CCDC124	Coiled-coil domain-containing protein 124	19.26	19.9	70.4	11	1.061
sp|Q9NXW2|DJB12_HUMAN	DNAJB12	DnaJ homolog subfamily B member 12	7.83	7.99	34.1	5	1.061
sp|Q9Y6C9|MTCH2_HUMAN	MTCH2	Mitochondrial carrier homolog 2	17.37	17.5	51.8	19	1.061
sp|P23396|RS3_HUMAN	RPS3	40S ribosomal protein S3	37.03	37.1	86.8	43	1.061
sp|O14773|TPP1_HUMAN	TPP1	Tripeptidyl-peptidase 1	12.11	12.12	27	9	1.061
sp|P43246|MSH2_HUMAN	MSH2	DNA mismatch repair protein Msh2	69.46	71.33	57	47	1.061
sp|P57076|CU059_HUMAN	C21orf59	UPF0769 protein C21orf59	11.84	12.23	72.4	12	1.061
sp|Q8NI27|THOC2_HUMAN	THOC2	THO complex subunit 2	49.88	52.09	36.4	27	1.061
sp|O43399|TPD54_HUMAN	TPD52L2	Tumor protein D54	23.63	24.9	85.9	21	1.061
sp|Q96SZ5|AEDO_HUMAN	ADO	2-aminoethanethiol dioxygenase	7.24	7.36	38.9	5	1.061
sp|O00746|NDKM_HUMAN	NME4	Nucleoside diphosphate kinase, mitochondrial	8.29	9.16	48.7	5	1.062
sp|O15294|OGT1_HUMAN	OGT	UDP-N-acetylglucosamine--peptide N-acetylglucosaminyltransferase 110 kDa subunit	46.61	48.06	40.2	30	1.062
sp|Q5T2W1|NHRF3_HUMAN	PDZK1	Na(+)/H(+) exchange regulatory cofactor NHE-RF3	6.58	6.74	28.5	4	1.062
sp|Q7L5N7|PCAT2_HUMAN	LPCAT2	Lysophosphatidylcholine acyltransferase 2	14.09	15.4	33.6	10	1.062
sp|O00401|WASL_HUMAN	WASL	Neural Wiskott-Aldrich syndrome protein	13.98	14.2	48.1	10	1.062
sp|Q96JB3|HIC2_HUMAN	HIC2	Hypermethylated in cancer 2 protein	2.55	2.6	15.8	2	1.062
sp|Q9H2H9|S38A1_HUMAN	SLC38A1	Sodium-coupled neutral amino acid transporter 1	2.5	2.64	18.5	4	1.062
sp|P11310|ACADM_HUMAN	ACADM	Medium-chain specific acyl-CoA dehydrogenase, mitochondrial	38.92	39.08	61.1	37	1.062
sp|Q9HAU5|RENT2_HUMAN	UPF2	Regulator of nonsense transcripts 2	23.53	26.97	30	13	1.062
sp|Q9BYC5|FUT8_HUMAN	FUT8	Alpha-(1,6)-fucosyltransferase	5.51	5.69	17.7	3	1.062
sp|O60315|ZEB2_HUMAN	ZEB2	Zinc finger E-box-binding homeobox 2	1.91	2.31	10.3	3	1.062
sp|Q29RF7|PDS5A_HUMAN	PDS5A	Sister chromatid cohesion protein PDS5 homolog A	69.52	71.56	50.9	41	1.062
sp|P53367|ARFP1_HUMAN	ARFIP1	Arfaptin-1	15.91	20.32	47.7	13	1.062
sp|Q96L91|EP400_HUMAN	EP400	E1A-binding protein p400	31.93	32.98	17.4	20	1.062
sp|O14772|FPGT_HUMAN	FPGT	Fucose-1-phosphate guanylyltransferase	8.13	8.44	27.3	5	1.063
sp|O14828|SCAM3_HUMAN	SCAMP3	Secretory carrier-associated membrane protein 3	15.91	16.02	45	13	1.063

sp|Q86VN1|VPS36_HUMAN	VPS36	Vacuolar protein-sorting-associated protein 36	16.34	20.92	64	12	1.063
sp|Q9Y5L0|TNPO3_HUMAN	TNPO3	Transportin-3	39.09	39.19	37.7	22	1.063
sp|Q99871|HAUS7_HUMAN	HAUS7	HAUS augmin-like complex subunit 7	6.02	6.03	25.3	3	1.063
sp|Q14686|NCOA6_HUMAN	NCOA6	Nuclear receptor coactivator 6	2.41	3.77	8.4	4	1.063
sp|Q15738|NSDHL_HUMAN	NSDHL	Sterol-4-alpha-carboxylate 3-dehydrogenase, decarboxylating	40.15	40.4	76.7	30	1.063
sp|Q2TAA5|ALG11_HUMAN	ALG11	GDP-Man:Man(3)GlcNAc(2)-PP-Dol alpha-1,2-mannosyltransferase	7.45	7.56	22.2	4	1.063
sp|O14737|PDCD5_HUMAN	PDCD5	Programmed cell death protein 5	13.76	13.93	76	18	1.063
sp|Q10713|MPPA_HUMAN	PMPCA	Mitochondrial-processing peptidase subunit alpha	38.93	39.47	58.7	26	1.063
sp|Q96S44|PRPK_HUMAN	TP53RK	TP53-regulating kinase	5.87	7.94	44.7	5	1.063
sp|Q9BX68|HINT2_HUMAN	HINT2	Histidine triad nucleotide-binding protein 2, mitochondrial	6.56	6.68	62	6	1.064
sp|O75718|CRTAP_HUMAN	CRTAP	Cartilage-associated protein	15.21	15.42	43.4	10	1.064
sp|Q8NEM2|SHCBP_HUMAN	SHCBP1	SHC SH2 domain-binding protein 1	4.78	5.11	23.5	5	1.064
sp|O15014|ZN609_HUMAN	ZNF609	Zinc finger protein 609	2.7	3.04	12.8	3	1.064
sp|O94829|IPO13_HUMAN	IPO13	Importin-13	2.32	4.53	15.1	4	1.064
sp|Q4KMQ2|ANO6_HUMAN	ANO6	Anoctamin-6	15.3	17.87	23.5	11	1.064
sp|Q9Y4W6|AFG32_HUMAN	AFG3L2	AFG3-like protein 2	57.27	60.38	55.2	36	1.064
sp|Q96D46|NMD3_HUMAN	NMD3	60S ribosomal export protein NMD3	29.46	29.55	45.9	19	1.064
sp|P84022|SMAD3_HUMAN	SMAD3	Mothers against decapentaplegic homolog 3	8.56	13.54	35.5	7	1.064
sp|Q9H9B4|SFXN1_HUMAN	SFXN1	Sideroflexin-1	29.97	30.02	72.4	40	1.064
sp|Q13428|TCOF_HUMAN	TCOF1	Treacle protein	93.45	93.59	43.8	65	1.064
sp|Q12926|ELAV2_HUMAN	ELAVL2	ELAV-like protein 2	3.21	5.45	40.4	6	1.064
sp|Q8IX18|DHX40_HUMAN	DHX40	Probable ATP-dependent RNA helicase DHX40	12.93	13.19	30	7	1.065
sp|Q9ULW0|TPX2_HUMAN	TPX2	Targeting protein for Xklp2	40.77	40.9	49.8	25	1.065
sp|O00559|RCAS1_HUMAN	EBAG9	Receptor-binding cancer antigen expressed on SiSo cells	2.09	2.12	34.7	2	1.065
sp|P35613|BASI_HUMAN	BSG	Basigin	28.16	29.16	45.5	29	1.065
sp|Q5SRE7|PHYD1_HUMAN	PHYHD1	Phytanoyl-CoA dioxygenase domain-containing protein 1	2.86	2.97	23	4	1.065
sp|Q9NYK5|RM39_HUMAN	MRPL39	39S ribosomal protein L39, mitochondrial	26.1	26.58	57.4	13	1.065
sp|P00395|COX1_HUMAN	MT-CO1	Cytochrome c oxidase subunit 1	4	4	14.6	4	1.065
sp|O00151|PDLI1_HUMAN	PDLIM1	PDZ and LIM domain protein 1	49.84	51.47	90.3	53	1.065
sp|O60318|GANP_HUMAN	MCM3AP	Germinal-center associated nuclear protein	28.51	29.75	23.1	17	1.065
sp|Q9BXF6|RFIP5_HUMAN	RAB11FIP5	Rab11 family-interacting protein 5	10.51	10.58	26.2	5	1.065
sp|Q96T88|UHRF1_HUMAN	UHRF1	E3 ubiquitin-protein ligase UHRF1	37.21	37.38	41.7	24	1.065
sp|Q8IYB5|SMAP1_HUMAN	SMAP1	Stromal membrane-associated protein 1	7.82	7.92	19.5	4	1.066
sp|Q13724|MOGS_HUMAN	MOGS	Mannosyl-oligosaccharide glucosidase	50.27	52.41	50.8	38	1.066
sp|Q5T0F9|C2D1B_HUMAN	CC2D1B	Coiled-coil and C2 domain-containing protein 1B	13.25	13.56	26.9	8	1.066
sp|Q9BVL4|SELO_HUMAN	SELO	Selenoprotein O	2.3	2.4	21.2	2	1.066
sp|O43741|AAKB2_HUMAN	PRKAB2	5'-AMP-activated protein kinase subunit beta-2	4.78	6.94	38.6	4	1.066
sp|O43502|RA51C_HUMAN	RAD51C	DNA repair protein RAD51 homolog 3	4	4.01	14.9	2	1.066
sp|P16615|AT2A2_HUMAN	ATP2A2	Sarcoplasmic/endoplasmic reticulum calcium ATPase 2	85.35	88.26	50.3	69	1.066
sp|Q99613|EIF3C_HUMAN	EIF3C	Eukaryotic translation initiation factor 3 subunit C	52.65	56.67	42.8	47	1.066
sp|P07686|HEXB_HUMAN	HEXB	Beta-hexosaminidase subunit beta	33.69	34.67	46.8	20	1.066
sp|P09622|DLDH_HUMAN	DLD	Dihydrolipoyl dehydrogenase, mitochondrial	40.4	40.66	69	44	1.066
sp|Q15906|VPS72_HUMAN	VPS72	Vacuolar protein sorting-associated protein 72 homolog	4.06	4.32	18.4	4	1.066
sp|Q96HQ2|C2AIL_HUMAN	CDKN2AIPNL	CDKN2AIP N-terminal-like protein	8	8.01	53.5	7	1.066
sp|Q9NVM6|DJC17_HUMAN	DNAJC17	DnaJ homolog subfamily C member 17	7.52	7.67	39.8	5	1.066
sp|Q9P2D0|IBTK_HUMAN	IBTK	Inhibitor of Bruton tyrosine kinase	4.13	4.44	25.4	7	1.067
sp|O43913|ORC5_HUMAN	ORC5	Origin recognition complex subunit 5	11.39	11.66	40.2	7	1.067
sp|Q9BWE0|REPI1_HUMAN	REPIN1	Replication initiator 1	8.27	8.45	25.2	5	1.067
sp|Q9Y3C4|TPRKB_HUMAN	TPRKB	EKC/KEOPS complex subunit TPRKB	10.86	10.95	72	8	1.067

sp|Q71RG4|TMUB2_HUMAN	TMUB2	Transmembrane and ubiquitin-like domain-containing protein 2	2.4	2.44	17.5	2	1.067
sp|Q99081|HTF4_HUMAN	TCF12	Transcription factor 12	2.72	2.86	5.6	2	1.067
sp|P26599|PTBP1_HUMAN	PTBP1	Polypyrimidine tract-binding protein 1	63.97	64.83	88.9	176	1.067
sp|P29401|TKT_HUMAN	TKT	Transketolase	81.63	82.29	83.5	109	1.067
sp|O95197|RTN3_HUMAN	RTN3	Reticulon-3	3.1	3.53	20	8	1.068
sp|Q7Z2E3|APTX_HUMAN	APTX	Aprataxin	5.7	5.97	24.2	6	1.068
sp|Q04837|SSBP_HUMAN	SSBP1	Single-stranded DNA-binding protein, mitochondrial	18.04	18.46	68.9	30	1.068
sp|O14802|RPC1_HUMAN	POLR3A	DNA-directed RNA polymerase III subunit RPC1	64.17	68.53	49	36	1.068
sp|Q07666|KHDR1_HUMAN	KHDRBS1	KH domain-containing, RNA-binding, signal transduction-associated protein 1	31.83	32.17	50.1	38	1.068
sp|Q14671|PUM1_HUMAN	PUM1	Pumilio homolog 1	32.67	35.35	34.4	21	1.068
sp|Q9UKL0|RCOR1_HUMAN	RCOR1	REST corepressor 1	23.63	24.04	45.4	13	1.068
sp|O15550|KDM6A_HUMAN	KDM6A	Lysine-specific demethylase 6A	9.39	9.8	15.2	6	1.068
sp|Q13084|RM28_HUMAN	MRPL28	39S ribosomal protein L28, mitochondrial	18.8	19.5	66.4	12	1.068
sp|Q69YU5|CL073_HUMAN	C12orf73	Uncharacterized protein C12orf73	2.48	2.51	59.2	2	1.068
sp|Q86TI2|DPP9_HUMAN	DPP9	Dipeptidyl peptidase 9	31.72	33.58	32	17	1.069
sp|P62879|GBB2_HUMAN	GNB2	Guanine nucleotide-binding protein G(I)/G(S)/G(T) subunit beta-2	26.19	28.03	69.4	30	1.069
sp|Q8NEC7|GSTCD_HUMAN	GSTCD	Glutathione S-transferase C-terminal domain-containing protein	9.35	9.61	26.1	5	1.069
sp|P10646|TFPI1_HUMAN	TFPI	Tissue factor pathway inhibitor	7.1	7.18	43.4	4	1.069
sp|Q8N0U8|VKORL_HUMAN	VKORC1L1	Vitamin K epoxide reductase complex subunit 1-like protein 1	4.53	4.64	29	3	1.069
sp|P62258|1433E_HUMAN	YWHAE	14-3-3 protein epsilon	49.76	50.41	83.1	81	1.069
sp|O95299|NDUAA_HUMAN	NDUFA10	NADH dehydrogenase [ubiquinone] 1 alpha subcomplex subunit 10, mitochondrial	32.31	32.44	58.9	23	1.069
sp|P33993|MCM7_HUMAN	MCM7	DNA replication licensing factor MCM7	74.44	74.57	74.3	58	1.070
sp|O00217|NDUS8_HUMAN	NDUFS8	NADH dehydrogenase [ubiquinone] iron-sulfur protein 8, mitochondrial	8.52	8.57	45.7	8	1.070
sp|P07384|CAN1_HUMAN	CAPN1	Calpain-1 catalytic subunit	49.98	50.16	60.2	30	1.070
sp|Q03519|TAP2_HUMAN	TAP2	Antigen peptide transporter 2	14.15	14.33	26.1	8	1.070
sp|Q9BQG0|MBB1A_HUMAN	MYBBP1A	Myb-binding protein 1A	95.44	98.83	52.3	77	1.070
sp|Q86X83|COMD2_HUMAN	COMMD2	COMM domain-containing protein 2	2.04	2.16	17.6	2	1.070
sp|P32019|I5P2_HUMAN	INPP5B	Type II inositol 1,4,5-trisphosphate 5-phosphatase	2.1	4.38	15.8	3	1.070
sp|O75330|HMMR_HUMAN	HMMR	Hyaluronan mediated motility receptor	15.42	17.02	45.2	12	1.070
sp|O95229|ZWINT_HUMAN	ZWINT	ZW10 interactor	6.06	6.14	26.7	3	1.071
sp|Q9H6D7|HAUS4_HUMAN	HAUS4	HAUS augmin-like complex subunit 4	4.93	6	40.5	7	1.071
sp|Q9H6H4|REEP4_HUMAN	REEP4	Receptor expression-enhancing protein 4	9.35	9.42	30.7	5	1.071
sp|Q13011|ECH1_HUMAN	ECH1	Delta(3,5)-Delta(2,4)-dienoyl-CoA isomerase, mitochondrial	29.81	29.85	66.8	29	1.071
sp|A6NKD9|CC85C_HUMAN	CCDC85C	Coiled-coil domain-containing protein 85C	9.87	10.03	40.1	6	1.071
sp|Q9Y6D5|BIG2_HUMAN	ARFGEF2	Brefeldin A-inhibited guanine nucleotide-exchange protein 2	53.15	53.7	31.6	30	1.071
sp|Q7Z3J2|CP062_HUMAN	C16orf62	UPF0505 protein C16orf62	3.41	3.62	14.1	3	1.071
sp|P36542|ATPG_HUMAN	ATP5C1	ATP synthase subunit gamma, mitochondrial	26.65	28.4	63.8	28	1.071
sp|P21283|VATC1_HUMAN	ATP6V1C1	V-type proton ATPase subunit C 1	23.94	24.14	56	13	1.071
sp|O95363|SYFM_HUMAN	FARS2	Phenylalanine--tRNA ligase, mitochondrial	10.87	10.95	32.6	6	1.071
sp|Q92562|FIG4_HUMAN	FIG4	Polyphosphoinositide phosphatase	3.3	5.25	8.6	4	1.071
sp|Q6IBW4|CNDH2_HUMAN	NCAPH2	Condensin-2 complex subunit H2	2.02	2.03	13.2	2	1.071
sp|Q96BP3|PPWD1_HUMAN	PPWD1	Peptidylprolyl isomerase domain and WD repeat-containing protein 1	31.33	31.57	46.4	16	1.071
sp|P03897|NU3M_HUMAN	MT-ND3	NADH-ubiquinone oxidoreductase chain 3	2	2	24.4	2	1.072
sp|O15121|DEGS1_HUMAN	DEGS1	Sphingolipid delta(4)-desaturase DES1	6.01	6.47	21.4	4	1.072
sp|Q9Y485|DMXL1_HUMAN	DMXL1	DmX-like protein 1	2.69	5.32	9.6	4	1.072
sp|O15269|SPTC1_HUMAN	SPTLC1	Serine palmitoyltransferase 1	22.91	23.15	62.6	13	1.072
sp|P35226|BMI1_HUMAN	BMI1	Polycomb complex protein BMI-1	7.68	7.8	32.5	4	1.072
sp|Q8IV08|PLD3_HUMAN	PLD3	Phospholipase D3	9.92	10.02	24.1	8	1.072
sp|Q9GZT3|SLIRP_HUMAN	SLIRP	SRA stem-loop-interacting RNA-binding protein, mitochondrial	14.08	14.24	75.2	17	1.072

sp|O60934|NBN_HUMAN	NBN	Nibrin	15.08	15.45	35.2	9	1.072
sp|Q15005|SPCS2_HUMAN	SPCS2	Signal peptidase complex subunit 2	26.67	28.39	56.2	19	1.072
sp|Q9NVH2|INT7_HUMAN	INTS7	Integrator complex subunit 7	15.29	16.38	28.4	10	1.072
sp|A6NIH7|U119B_HUMAN	UNC119B	Protein unc-119 homolog B	19.43	19.5	68.9	10	1.072
sp|Q9UQ80|PA2G4_HUMAN	PA2G4	Proliferation-associated protein 2G4	56.5	60.3	85	65	1.072
sp|Q8N543|OGFD1_HUMAN	OGFOD1	Prolyl 3-hydroxylase OGFOD1	21.32	22.86	37.3	13	1.072
sp|Q6ZRS2|SRCAP_HUMAN	SRCAP	Helicase SRCAP	26.2	27.09	14.2	16	1.073
sp|P11216|PYGB_HUMAN	PYGB	Glycogen phosphorylase, brain form	67.89	85.74	71.3	64	1.073
sp|P98173|FAM3A_HUMAN	FAM3A	Protein FAM3A	2.75	2.92	14.8	2	1.073
sp|P78345|RPP38_HUMAN	RPP38	Ribonuclease P protein subunit p38	16.54	16.74	59	11	1.073
sp|Q9BUI4|RPC3_HUMAN	POLR3C	DNA-directed RNA polymerase III subunit RPC3	22.29	22.69	43.5	16	1.073
sp|P35219|CAH8_HUMAN	CA8	Carbonic anhydrase-related protein	10.55	10.67	37.6	6	1.073
sp|Q10472|GALT1_HUMAN	GALNT1	Polypeptide N-acetylgalactosaminyltransferase 1	13.6	14.37	33.1	9	1.073
sp|Q5BJH7|YIF1B_HUMAN	YIF1B	Protein YIF1B	6.59	6.64	29.3	4	1.073
sp|Q9H081|MIS12_HUMAN	MIS12	Protein MIS12 homolog	6.4	6.45	38.1	4	1.073
sp|P35670|ATP7B_HUMAN	ATP7B	Copper-transporting ATPase 2	3.59	4.02	16.1	3	1.074
sp|P31947|1433S_HUMAN	SFN	14-3-3 protein sigma	15.31	26.33	82.7	23	1.074
sp|Q8WXE1|ATRIP_HUMAN	ATRIP	ATR-interacting protein	2.55	2.72	15.8	4	1.074
sp|Q8IX12|CCAR1_HUMAN	CCAR1	Cell division cycle and apoptosis regulator protein 1	41.16	42.91	35.9	25	1.074
sp|O75592|MYCB2_HUMAN	MYCBP2	E3 ubiquitin-protein ligase MYCBP2	16.06	19.47	15.6	18	1.074
sp|Q96GM8|TOE1_HUMAN	TOE1	Target of EGR1 protein 1	22.45	22.84	42.6	15	1.074
sp|Q05513|KPCZ_HUMAN	PRKCZ	Protein kinase C zeta type	4.01	4.09	14	2	1.074
sp|Q9NYJ1|COA4_HUMAN	COA4	Cytochrome c oxidase assembly factor 4 homolog, mitochondrial	7.87	7.98	70.1	7	1.075
sp|Q8N4S7|PAQR4_HUMAN	PAQR4	Progestin and adipoQ receptor family member 4	3.96	4	13.6	2	1.075
sp|P27348|1433T_HUMAN	YWHAQ	14-3-3 protein theta	31.8	42.82	79.6	46	1.075
sp|P54760|EPHB4_HUMAN	EPHB4	Ephrin type-B receptor 4	24.73	29.39	35.6	19	1.075
sp|Q6PI78|TMM65_HUMAN	TMEM65	Transmembrane protein 65	7.12	7.19	33.8	4	1.075
sp|Q5HYI7|MTX3_HUMAN	MTX3	Metaxin-3	5.14	5.27	26.9	5	1.075
sp|Q9Y248|PSF2_HUMAN	GINS2	DNA replication complex GINS protein PSF2	8.9	9.07	63.8	6	1.075
sp|Q13162|PRDX4_HUMAN	PRDX4	Peroxiredoxin-4	23.86	28.92	79.7	39	1.075
sp|O75113|N4BP1_HUMAN	N4BP1	NEDD4-binding protein 1	8.22	8.64	24.6	7	1.075
sp|O95571|ETHE1_HUMAN	ETHE1	Persulfide dioxygenase ETHE1, mitochondrial	14.04	14.05	44.5	9	1.075
sp|Q99856|ARI3A_HUMAN	ARID3A	AT-rich interactive domain-containing protein 3A	20.7	20.83	33.4	16	1.075
sp|P43378|PTN9_HUMAN	PTPN9	Tyrosine-protein phosphatase non-receptor type 9	5.01	5.19	17.9	5	1.076
sp|Q17R31|TATD3_HUMAN	TATDN3	Putative deoxyribonuclease TATDN3	2	2.03	23.4	2	1.076
sp|Q9NXH9|TRM1_HUMAN	TRMT1	tRNA (guanine(26)-N(2))-dimethyltransferase	33.36	33.94	47.8	17	1.076
sp|O75390|CISY_HUMAN	CS	Citrate synthase, mitochondrial	45.3	45.3	64.8	62	1.076
sp|P54819|KAD2_HUMAN	AK2	Adenylate kinase 2, mitochondrial	44.17	44.23	86.6	51	1.076
sp|Q96G03|PGM2_HUMAN	PGM2	Phosphoglucomutase-2	35.25	37.19	50.8	22	1.076
sp|Q6QNY1|BL1S2_HUMAN	BLOC1S2	Biogenesis of lysosome-related organelles complex 1 subunit 2	4.26	4.85	61.3	3	1.077
sp|Q15036|SNX17_HUMAN	SNX17	Sorting nexin-17	9.71	9.85	31.9	5	1.077
sp|P60891|PRPS1_HUMAN	PRPS1	Ribose-phosphate pyrophosphokinase 1	22.67	22.78	55	21	1.077
sp|Q9NVG8|TBC13_HUMAN	TBC1D13	TBC1 domain family member 13	16.58	16.69	38.5	10	1.077
sp|Q9Y3D0|MIP18_HUMAN	FAM96B	Mitotic spindle-associated MMXD complex subunit MIP18	11.56	11.66	60.7	8	1.077
sp|P05156|CFAI_HUMAN	CFI	Complement factor I	4.76	4.87	17.2	3	1.077
sp|O95834|EMAL2_HUMAN	EML2	Echinoderm microtubule-associated protein-like 2	4.92	5	12.3	3	1.077
sp|Q9BRT2|UQCC2_HUMAN	UQCC2	Ubiquinol-cytochrome-c reductase complex assembly factor 2	12.74	12.84	66.7	7	1.077
sp|O14735|CDIPT_HUMAN	CDIPT	CDP-diacylglycerol--inositol 3-phosphatidyltransferase	6.72	6.78	27.7	6	1.077
sp|Q96AD5|PLPL2_HUMAN	PNPLA2	Patatin-like phospholipase domain-containing protein 2	4.37	5.07	23.2	4	1.077

sp|Q8N0Z6|TTC5_HUMAN	TTC5	Tetratricopeptide repeat protein 5	18.46	18.5	37.5	9	1.077
sp|Q7Z7E8|UB2Q1_HUMAN	UBE2Q1	Ubiquitin-conjugating enzyme E2 Q1	8.97	9.61	37	6	1.077
sp|Q86U44|MTA70_HUMAN	METTL3	N6-adenosine-methyltransferase 70 kDa subunit	26.17	26.25	55.9	13	1.078
sp|P15170|ERF3A_HUMAN	GSPT1	Eukaryotic peptide chain release factor GTP-binding subunit ERF3A	74.26	74.36	72.1	59	1.078
sp|Q8N108|MIER1_HUMAN	MIER1	Mesoderm induction early response protein 1	6.85	8.76	23.2	5	1.078
sp|Q9Y4E6|WDR7_HUMAN	WDR7	WD repeat-containing protein 7	4.64	5.37	13.5	6	1.078
sp|Q5HYI8|RABL3_HUMAN	RABL3	Rab-like protein 3	9.97	10.02	56.8	7	1.078
sp|Q9UMX5|NENF_HUMAN	NENF	Neudesin	12.09	12.1	70.4	10	1.079
sp|Q969V3|NCLN_HUMAN	NCLN	Nicalin	32.52	33.51	54.9	26	1.079
sp|Q9Y230|RUVB2_HUMAN	RUVBL2	RuvB-like 2	56.95	57.7	75.8	58	1.079
sp|P60002|ELOF1_HUMAN	ELOF1	Transcription elongation factor 1 homolog	2	2	21.7	2	1.079
sp|Q8NBP0|TTC13_HUMAN	TTC13	Tetratricopeptide repeat protein 13	18.58	18.83	30	13	1.079
sp|Q9BXJ9|NAA15_HUMAN	NAA15	N-alpha-acetyltransferase 15, NatA auxiliary subunit	84.05	86	68.9	54	1.079
sp|P12931|SRC_HUMAN	SRC	Proto-oncogene tyrosine-protein kinase Src	34.02	34.03	48.5	20	1.079
sp|Q5VWZ2|LYPL1_HUMAN	LYPLAL1	Lysophospholipase-like protein 1	6.14	6.16	39.7	4	1.079
sp|Q9H8H3|MET7A_HUMAN	METTL7A	Methyltransferase-like protein 7A	9.12	10.02	44.7	6	1.080
sp|O15371|EIF3D_HUMAN	EIF3D	Eukaryotic translation initiation factor 3 subunit D	39.51	40.38	65.5	33	1.080
sp|P11168|GTR2_HUMAN	SLC2A2	Solute carrier family 2, facilitated glucose transporter member 2	5.43	5.79	14.5	5	1.080
sp|Q9Y3B9|RRP15_HUMAN	RRP15	RRP15-like protein	15.02	15.29	42.9	10	1.080
sp|Q9UKJ3|GPTC8_HUMAN	GPATCH8	G patch domain-containing protein 8	9.81	11.21	13.8	6	1.080
sp|Q2TBE0|C19L2_HUMAN	CWF19L2	CWF19-like protein 2	12.93	13.19	30.7	7	1.080
sp|Q9BYW2|SETD2_HUMAN	SETD2	Histone-lysine N-methyltransferase SETD2	10	13.12	12.8	8	1.080
sp|P27544|CERS1_HUMAN	CERS1	Ceramide synthase 1	2.38	2.47	14	2	1.080
sp|Q9C0B0|UNK_HUMAN	UNK	RING finger protein unkempt homolog	4.3	4.45	16.4	4	1.080
sp|O60524|NEMF_HUMAN	NEMF	Nuclear export mediator factor NEMF	17.74	18.63	28.5	12	1.080
sp|Q7Z478|DHX29_HUMAN	DHX29	ATP-dependent RNA helicase DHX29	43.84	47.44	40.1	26	1.080
sp|Q9NWB7|IFT57_HUMAN	IFT57	Intraflagellar transport protein 57 homolog	4	4.01	23.5	2	1.080
sp|Q9BPY3|F118B_HUMAN	FAM118B	Protein FAM118B	6.64	6.8	33.3	5	1.081
sp|Q8N0Y2|ZN444_HUMAN	ZNF444	Zinc finger protein 444	2.05	2.31	16.2	2	1.081
sp|P29144|TPP2_HUMAN	TPP2	Tripeptidyl-peptidase 2	80.28	80.36	52.4	44	1.081
sp|Q9NZI7|UBIP1_HUMAN	UBP1	Upstream-binding protein 1	10.08	12.48	31.9	6	1.081
sp|Q92665|RT31_HUMAN	MRPS31	28S ribosomal protein S31, mitochondrial	25.92	28.72	59.8	21	1.081
sp|P24863|CCNC_HUMAN	CCNC	Cyclin-C	2.01	4.04	24	3	1.081
sp|Q01826|SATB1_HUMAN	SATB1	DNA-binding protein SATB1	4.02	10.45	20.8	9	1.081
sp|Q14145|KEAP1_HUMAN	KEAP1	Kelch-like ECH-associated protein 1	14.91	14.99	34.8	8	1.082
sp|Q6KC79|NIPBL_HUMAN	NIPBL	Nipped-B-like protein	53.46	56.55	29	36	1.083
sp|Q9NZ08|ERAP1_HUMAN	ERAP1	Endoplasmic reticulum aminopeptidase 1	19.07	20.1	27.3	13	1.083
sp|Q00169|PIPNA_HUMAN	PITPNA	Phosphatidylinositol transfer protein alpha isoform	12.27	18.84	69.6	10	1.083
sp|Q92597|NDRG1_HUMAN	NDRG1	Protein NDRG1	7.51	7.89	27.7	5	1.083
sp|Q9NX08|COMD8_HUMAN	COMMD8	COMM domain-containing protein 8	2.43	2.59	32.2	2	1.083
sp|Q99808|S29A1_HUMAN	SLC29A1	Equilibrative nucleoside transporter 1	7.78	7.89	21.5	4	1.083
sp|Q9NYB9|ABI2_HUMAN	ABI2	Abl interactor 2	12.29	12.54	20.7	7	1.083
sp|P20700|LMNB1_HUMAN	LMNB1	Lamin-B1	90.54	92.34	80.6	101	1.083
sp|Q13472|TOP3A_HUMAN	TOP3A	DNA topoisomerase 3-alpha	11.34	11.84	21.1	8	1.083
sp|Q6ZSJ8|CA122_HUMAN	C1orf122	Uncharacterized protein C1orf122	6.46	6.5	76.4	4	1.084
sp|Q9UQB8|BAIP2_HUMAN	BAIAP2	Brain-specific angiogenesis inhibitor 1-associated protein 2	36.1	36.32	59.2	19	1.084
sp|Q9H2G2|SLK_HUMAN	SLK	STE20-like serine/threonine-protein kinase	40.45	46.07	46.3	22	1.084
sp|Q96BP2|CHCH1_HUMAN	CHCHD1	Coiled-coil-helix-coiled-coil-helix domain-containing protein 1	3.15	3.23	32.2	2	1.084
sp|O43683|BUB1_HUMAN	BUB1	Mitotic checkpoint serine/threonine-protein kinase BUB1	1.33	3.63	8.8	3	1.084

sp|Q96IW7|SC22A_HUMAN	SEC22A	Vesicle-trafficking protein SEC22a	6.03	6.06	26.7	6	1.084
sp|Q9Y6M7|S4A7_HUMAN	SLC4A7	Sodium bicarbonate cotransporter 3	23.07	24.25	29.7	16	1.084
sp|Q9UL40|ZN346_HUMAN	ZNF346	Zinc finger protein 346	13.84	13.94	49	8	1.085
sp|P62854|RS26_HUMAN	RPS26	40S ribosomal protein S26	5.71	5.78	34.8	11	1.085
sp|O95831|AIFM1_HUMAN	AIFM1	Apoptosis-inducing factor 1, mitochondrial	44.81	45.13	68.4	38	1.085
sp|Q9Y5S2|MRCKB_HUMAN	CDC42BPB	Serine/threonine-protein kinase MRCK beta	35.44	38.25	35.8	23	1.085
sp|Q16537|2A5E_HUMAN	PPP2R5E	Serine/threonine-protein phosphatase 2A 56 kDa regulatory subunit epsilon isoform	6.95	8.37	38.3	6	1.085
sp|Q9UK61|TASOR_HUMAN	FAM208A	Protein TASOR	47.53	48.36	31.9	27	1.085
sp|Q8TEL6|TP4AP_HUMAN	TRPC4AP	Short transient receptor potential channel 4-associated protein	9.79	12.15	27.2	10	1.085
sp|Q14728|MFS10_HUMAN	MFSD10	Major facilitator superfamily domain-containing protein 10	2.14	2.21	21.3	2	1.085
sp|Q14694|UBP10_HUMAN	USP10	Ubiquitin carboxyl-terminal hydrolase 10	41.93	43.57	51.1	32	1.085
sp|P50151|GBG10_HUMAN	GNG10	Guanine nucleotide-binding protein G(I)/G(S)/G(O) subunit gamma-10	2.27	4.35	35.3	3	1.086
sp|O14578|CTRO_HUMAN	CIT	Citron Rho-interacting kinase	18.93	22.79	26.8	15	1.086
sp|P19440|GGT1_HUMAN	GGT1	Gamma-glutamyltranspeptidase 1	7.68	7.84	20.6	4	1.086
sp|Q52LJ0|FA98B_HUMAN	FAM98B	Protein FAM98B	33.39	33.58	68.5	19	1.086
sp|Q86U38|NOP9_HUMAN	NOP9	Nucleolar protein 9	30.87	33.49	48.4	24	1.086
sp|Q13439|GOGA4_HUMAN	GOLGA4	Golgin subfamily A member 4	57.42	65.47	43.3	40	1.086
sp|Q13952|NFYC_HUMAN	NFYC	Nuclear transcription factor Y subunit gamma	9.15	9.23	15.9	5	1.086
sp|Q5SQN1|SNP47_HUMAN	SNAP47	Synaptosomal-associated protein 47	6.5	6.59	28.7	4	1.086
sp|Q8NBJ7|SUMF2_HUMAN	SUMF2	Sulfatase-modifying factor 2	12.14	12.15	40.2	8	1.087
sp|Q9BQI0|AIF1L_HUMAN	AIF1L	Allograft inflammatory factor 1-like	9.31	9.53	60	5	1.087
sp|O15344|TRI18_HUMAN	MID1	E3 ubiquitin-protein ligase Midline-1	1.38	3.21	15.3	3	1.087
sp|Q9BVC4|LST8_HUMAN	MLST8	Target of rapamycin complex subunit LST8	7.52	7.87	40.5	5	1.087
sp|Q92643|GPI8_HUMAN	PIGK	GPI-anchor transamidase	19.17	19.32	49.6	10	1.087
sp|Q9H981|ARP8_HUMAN	ACTR8	Actin-related protein 8	6.67	6.89	28.7	6	1.087
sp|Q96EY5|MB12A_HUMAN	MVB12A	Multivesicular body subunit 12A	9.25	9.44	49.8	7	1.087
sp|P49792|RBP2_HUMAN	RANBP2	E3 SUMO-protein ligase RanBP2	221.72	221.73	57.9	134	1.087
sp|Q96ST2|IWS1_HUMAN	IWS1	Protein IWS1 homolog	5.35	6.35	19.1	6	1.087
sp|O75157|T22D2_HUMAN	TSC22D2	TSC22 domain family protein 2	4.06	4.07	9.2	2	1.087
sp|Q99933|BAG1_HUMAN	BAG1	BAG family molecular chaperone regulator 1	4.28	4.32	21.7	2	1.087
sp|P21399|ACOC_HUMAN	ACO1	Cytoplasmic aconitate hydratase	43.06	43.76	48	26	1.087
sp|Q9Y448|SKAP_HUMAN	KNSTRN	Small kinetochore-associated protein	4.18	4.22	30.7	2	1.087
sp|Q96T58|MINT_HUMAN	SPEN	Msx2-interacting protein	49.44	52.59	21.8	28	1.087
sp|P14635|CCNB1_HUMAN	CCNB1	G2/mitotic-specific cyclin-B1	11.44	11.54	28.9	9	1.088
sp|Q96L73|NSD1_HUMAN	NSD1	Histone-lysine N-methyltransferase, H3 lysine-36 and H4 lysine-20 specific	3.84	6.84	14.6	7	1.088
sp|Q9H2U1|DHX36_HUMAN	DHX36	ATP-dependent RNA helicase DHX36	27.74	30.44	38.8	18	1.088
sp|Q9NVX0|HAUS2_HUMAN	HAUS2	HAUS augmin-like complex subunit 2	6.41	6.49	40.9	4	1.088
sp|Q9UKG9|OCTC_HUMAN	CROT	Peroxisomal carnitine O-octanoyltransferase	4.49	4.58	23.2	3	1.088
sp|Q6P5Z2|PKN3_HUMAN	PKN3	Serine/threonine-protein kinase N3	2.13	8.3	21.4	6	1.088
sp|P04844|RPN2_HUMAN	RPN2	Dolichyl-diphosphooligosaccharide--protein glycosyltransferase subunit 2	59.19	59.21	73.2	63	1.088
sp|Q8N3F8|MILK1_HUMAN	MICALL1	MICAL-like protein 1	14.99	15.85	27	10	1.088
sp|Q6UWZ7|F175A_HUMAN	FAM175A	BRCA1-A complex subunit Abraxas	2.32	2.36	25.4	3	1.088
sp|Q9H7E2|TDRD3_HUMAN	TDRD3	Tudor domain-containing protein 3	2.07	2.22	14.9	3	1.088
sp|Q9BSR8|YIPF4_HUMAN	YIPF4	Protein YIPF4	3.7	3.78	13.1	3	1.088
sp|Q05209|PTN12_HUMAN	PTPN12	Tyrosine-protein phosphatase non-receptor type 12	22.02	22.37	30.8	13	1.088
sp|Q8IWT0|ARCH_HUMAN	ZBTB8OS	Protein archease	2.41	2.44	11.4	2	1.089
sp|Q9Y276|BCS1_HUMAN	BCS1L	Mitochondrial chaperone BCS1	17.72	18.1	52.5	14	1.089
sp|P49023|PAXI_HUMAN	PXN	Paxillin	10.18	10.39	34.7	10	1.089
sp|Q9NRP2|COXM2_HUMAN	CMC2	COX assembly mitochondrial protein 2 homolog	4.83	4.95	57	3	1.089

sp|Q86WA8|LONP2_HUMAN	LONP2	Lon protease homolog 2, peroxisomal	13.71	13.87	26.6	7	1.089
sp|P30501|1C02_HUMAN	HLA-C	HLA class I histocompatibility antigen, Cw-2 alpha chain	2	12.78	38	7	1.090
sp|O43172|PRP4_HUMAN	PRPF4	U4/U6 small nuclear ribonucleoprotein Prp4	29.69	30.03	51.2	19	1.090
sp|Q96E11|RRFM_HUMAN	MRRF	Ribosome-recycling factor, mitochondrial	15.35	15.92	58	9	1.090
sp|Q9BT30|ALKB7_HUMAN	ALKBH7	Alpha-ketoglutarate-dependent dioxygenase alkB homolog 7, mitochondrial	1.85	2.04	22.2	2	1.090
sp|Q13362|2A5G_HUMAN	PPP2R5C	Serine/threonine-protein phosphatase 2A 56 kDa regulatory subunit gamma isoform	5.48	8.53	36.6	6	1.090
sp|Q16513|PKN2_HUMAN	PKN2	Serine/threonine-protein kinase N2	55.16	58.11	53.8	32	1.090
sp|O95999|BCL10_HUMAN	BCL10	B-cell lymphoma/leukemia 10	5.16	5.46	36.1	3	1.090
sp|Q9UL26|RB22A_HUMAN	RAB22A	Ras-related protein Rab-22A	8.57	8.62	57.2	5	1.090
sp|O60343|TBCD4_HUMAN	TBC1D4	TBC1 domain family member 4	34.69	35.4	33.4	20	1.091
sp|Q86UV5|UBP48_HUMAN	USP48	Ubiquitin carboxyl-terminal hydrolase 48	10.06	10.44	22	7	1.091
sp|P46926|GNPI1_HUMAN	GNPDA1	Glucosamine-6-phosphate isomerase 1	25.31	25.94	72.7	22	1.091
sp|Q9Y5J7|TIM9_HUMAN	TIMM9	Mitochondrial import inner membrane translocase subunit Tim9	8.6	8.64	80.9	6	1.091
sp|Q13616|CUL1_HUMAN	CUL1	Cullin-1	55.2	56.09	59.4	39	1.091
sp|O75306|NDUS2_HUMAN	NDUFS2	NADH dehydrogenase [ubiquinone] iron-sulfur protein 2, mitochondrial	34.72	35.95	65.9	31	1.092
sp|Q96GY0|ZC21A_HUMAN	ZC2HC1A	Zinc finger C2HC domain-containing protein 1A	2.91	3.17	30.2	3	1.092
sp|Q8IWV8|UBR2_HUMAN	UBR2	E3 ubiquitin-protein ligase UBR2	11.33	12.14	20.7	9	1.092
sp|Q96JM7|LMBL3_HUMAN	L3MBTL3	Lethal(3)malignant brain tumor-like protein 3	5.74	5.91	21.5	4	1.092
sp|Q14527|HLTF_HUMAN	HLTF	Helicase-like transcription factor	44.96	46.05	45.5	25	1.092
sp|Q9P270|SLAI2_HUMAN	SLAIN2	SLAIN motif-containing protein 2	3.48	3.65	18.1	4	1.092
sp|P39748|FEN1_HUMAN	FEN1	Flap endonuclease 1	29.02	32.97	64	34	1.093
sp|Q15048|LRC14_HUMAN	LRRC14	Leucine-rich repeat-containing protein 14	6.01	8.05	19.7	5	1.093
sp|Q00266|METK1_HUMAN	MAT1A	S-adenosylmethionine synthase isoform type-1	13.43	16.11	47.9	10	1.093
sp|P46776|RL27A_HUMAN	RPL27A	60S ribosomal protein L27a	17.83	19.08	54.1	27	1.093
sp|O95202|LETM1_HUMAN	LETM1	LETM1 and EF-hand domain-containing protein 1, mitochondrial	52.6	54.1	65.4	39	1.093
sp|P59998|ARPC4_HUMAN	ARPC4	Actin-related protein 2/3 complex subunit 4	10.87	11.92	68.5	11	1.093
sp|O00458|IFRD1_HUMAN	IFRD1	Interferon-related developmental regulator 1	8.15	8.28	35.3	6	1.093
sp|Q5T5Y3|CAMP1_HUMAN	CAMSAP1	Calmodulin-regulated spectrin-associated protein 1	4.66	6.01	19.9	7	1.093
sp|Q96RY7|IF140_HUMAN	IFT140	Intraflagellar transport protein 140 homolog	2.62	2.75	15.9	3	1.094
sp|Q5XPI4|RN123_HUMAN	RNF123	E3 ubiquitin-protein ligase RNF123	5.54	5.84	11.5	4	1.094
sp|O95169|NDUB8_HUMAN	NDUFB8	NADH dehydrogenase [ubiquinone] 1 beta subcomplex subunit 8, mitochondrial	10.05	10.37	45.2	7	1.094
sp|Q9Y4D1|DAAM1_HUMAN	DAAM1	Disheveled-associated activator of morphogenesis 1	4.66	7.59	24.7	8	1.094
sp|O95602|RPA1_HUMAN	POLR1A	DNA-directed RNA polymerase I subunit RPA1	74.85	77.32	47.9	47	1.094
sp|Q96EQ0|SGTB_HUMAN	SGTB	Small glutamine-rich tetratricopeptide repeat-containing protein beta	4.12	4.22	20.7	3	1.094
sp|Q96EP5|DAZP1_HUMAN	DAZAP1	DAZ-associated protein 1	21.99	23.08	45.5	27	1.094
sp|Q15542|TAF5_HUMAN	TAF5	Transcription initiation factor TFIID subunit 5	10.46	10.59	19.6	9	1.094
sp|P0C0L4|CO4A_HUMAN	C4A	Complement C4-A	80.37	80.65	45.4	41	1.094
sp|P08581|MET_HUMAN	MET	Hepatocyte growth factor receptor	5.27	7.68	19	5	1.094
sp|Q6NYC1|JMJD6_HUMAN	JMJD6	Bifunctional arginine demethylase and lysyl-hydroxylase JMJD6	12.41	12.52	40.9	8	1.094
sp|Q14141|SEPT6_HUMAN	SEPT6	Septin-6	11.55	26.33	54.4	16	1.095
sp|Q6ULP2|AFTIN_HUMAN	AFTPH	Aftiphilin	5.06	5.16	18.7	3	1.095
sp|Q96EB6|SIR1_HUMAN	SIRT1	NAD-dependent protein deacetylase sirtuin-1	14.35	14.85	21.8	8	1.095
sp|Q7Z401|MYCPP_HUMAN	DENND4A	C-myc promoter-binding protein	5.34	5.7	15.1	4	1.095
sp|Q6IQ22|RAB12_HUMAN	RAB12	Ras-related protein Rab-12	6.47	10.87	50.8	8	1.096
sp|P31327|CPSM_HUMAN	CPS1	Carbamoyl-phosphate synthase [ammonia], mitochondrial	64.11	71.23	45.7	37	1.096
sp|P17252|KPCA_HUMAN	PRKCA	Protein kinase C alpha type	21.46	25.04	36	14	1.096
sp|O75554|WBP4_HUMAN	WBP4	WW domain-binding protein 4	3.16	3.35	14.4	2	1.096
sp|O96005|CLPT1_HUMAN	CLPTM1	Cleft lip and palate transmembrane protein 1	15.2	15.54	37.2	12	1.096
sp|P63098|CANB1_HUMAN	PPP3R1	Calcineurin subunit B type 1	11.64	11.7	57.7	8	1.097

sp|Q13596|SNX1_HUMAN	SNX1	Sorting nexin-1	40.52	41.09	49.6	29	1.097
sp|Q8TBC4|UBA3_HUMAN	UBA3	NEDD8-activating enzyme E1 catalytic subunit	35.61	36.01	71.1	22	1.097
sp|P04899|GNAI2_HUMAN	GNAI2	Guanine nucleotide-binding protein G(i) subunit alpha-2	30.66	33.01	72.4	23	1.097
sp|P35241|RADI_HUMAN	RDX	Radixin	39.24	70.32	71.7	50	1.097
sp|Q9NZD2|GLTP_HUMAN	GLTP	Glycolipid transfer protein	4.48	4.71	34.9	3	1.098
sp|Q6UN15|FIP1_HUMAN	FIP1L1	Pre-mRNA 3'-end-processing factor FIP1	21.8	21.88	34.5	13	1.098
sp|P58004|SESN2_HUMAN	SESN2	Sestrin-2	6.6	6.74	28.5	4	1.098
sp|Q9BV57|MTND_HUMAN	ADI1	1,2-dihydroxy-3-keto-5-methylthiopentene dioxygenase	22.67	24.67	91.1	17	1.098
sp|P07996|TSP1_HUMAN	THBS1	Thrombospondin-1	18.83	19.24	21.5	15	1.098
sp|P0CG08|GPHRB_HUMAN	GPR89B	Golgi pH regulator B	6.45	6.49	20.7	6	1.099
sp|O94763|RMP_HUMAN	URI1	Unconventional prefoldin RPB5 interactor 1	10.99	12.17	37	8	1.099
sp|Q8NB37|PDDC1_HUMAN	PDDC1	Parkinson disease 7 domain-containing protein 1	6.05	6.05	29.6	3	1.099
sp|P05166|PCCB_HUMAN	PCCB	Propionyl-CoA carboxylase beta chain, mitochondrial	33.1	33.65	63.5	20	1.099
sp|Q96B01|R51A1_HUMAN	RAD51AP1	RAD51-associated protein 1	1.94	2.12	21.3	2	1.100
sp|Q8NDZ4|DIA1_HUMAN	C3orf58	Deleted in autism protein 1	2.01	2.02	16.3	2	1.100
sp|P78356|PI42B_HUMAN	PIP4K2B	Phosphatidylinositol 5-phosphate 4-kinase type-2 beta	13.78	14	37	8	1.100
sp|Q9BYD2|RM09_HUMAN	MRPL9	39S ribosomal protein L9, mitochondrial	13.17	14.03	53.6	9	1.101
sp|Q9Y2G5|OFUT2_HUMAN	POFUT2	GDP-fucose protein O-fucosyltransferase 2	8.56	9.26	20.3	5	1.101
sp|Q9BR76|COR1B_HUMAN	CORO1B	Coronin-1B	17.99	24.09	44.4	15	1.101
sp|Q9HBI1|PARVB_HUMAN	PARVB	Beta-parvin	6.01	6.02	24.5	5	1.101
sp|Q8N2K0|ABD12_HUMAN	ABHD12	Monoacylglycerol lipase ABHD12	12.37	12.87	43.2	8	1.101
sp|Q9Y676|RT18B_HUMAN	MRPS18B	28S ribosomal protein S18b, mitochondrial	17.62	17.66	60.9	10	1.101
sp|P62304|RUXE_HUMAN	SNRPE	Small nuclear ribonucleoprotein E	8.04	8.06	69.6	15	1.101
sp|Q9UIA9|XPO7_HUMAN	XPO7	Exportin-7	63.53	65.89	49.7	40	1.101
sp|Q96G25|MED8_HUMAN	MED8	Mediator of RNA polymerase II transcription subunit 8	6.42	6.9	44	5	1.102
sp|Q8N5M4|TTC9C_HUMAN	TTC9C	Tetratricopeptide repeat protein 9C	4.34	4.88	59.1	4	1.102
sp|Q9H825|METL8_HUMAN	METTL8	Methyltransferase-like protein 8	1.36	1.52	19.2	2	1.102
sp|Q9H2M9|RBGPR_HUMAN	RAB3GAP2	Rab3 GTPase-activating protein non-catalytic subunit	41.99	45.29	36.7	26	1.102
sp|O15460|P4HA2_HUMAN	P4HA2	Prolyl 4-hydroxylase subunit alpha-2	29.81	32.49	52.5	21	1.103
sp|P19086|GNAZ_HUMAN	GNAZ	Guanine nucleotide-binding protein G(z) subunit alpha	3.9	4.79	22	4	1.103
sp|O75175|CNOT3_HUMAN	CNOT3	CCR4-NOT transcription complex subunit 3	13.27	14.32	17.3	12	1.103
sp|P61009|SPCS3_HUMAN	SPCS3	Signal peptidase complex subunit 3	4.2	4.24	27.8	8	1.103
sp|Q86VQ6|TRXR3_HUMAN	TXNRD3	Thioredoxin reductase 3	1.53	1.98	20.2	2	1.103
sp|Q9BYV8|CEP41_HUMAN	CEP41	Centrosomal protein of 41 kDa	5.03	5.87	20.4	5	1.103
sp|O15126|SCAM1_HUMAN	SCAMP1	Secretory carrier-associated membrane protein 1	10.1	10.21	41.1	7	1.103
sp|Q7Z5J4|RAI1_HUMAN	RAI1	Retinoic acid-induced protein 1	7.19	7.77	16.5	5	1.103
sp|Q9UBI6|GBG12_HUMAN	GNG12	Guanine nucleotide-binding protein G(I)/G(S)/G(O) subunit gamma-12	7.12	7.21	69.4	5	1.103
sp|Q9Y2X7|GIT1_HUMAN	GIT1	ARF GTPase-activating protein GIT1	14.27	25.44	36.7	13	1.103
sp|O14647|CHD2_HUMAN	CHD2	Chromodomain-helicase-DNA-binding protein 2	9.88	18.17	22.2	15	1.103
sp|Q96RR1|PEO1_HUMAN	PEO1	Twinkle protein, mitochondrial	11.24	12.3	31.1	6	1.103
sp|Q9UPT5|EXOC7_HUMAN	EXOC7	Exocyst complex component 7	21.23	21.86	40.4	13	1.103
sp|Q9H9S3|S61A2_HUMAN	SEC61A2	Protein transport protein Sec61 subunit alpha isoform 2	2.17	9.26	29.4	8	1.103
sp|Q8N129|CNPY4_HUMAN	CNPY4	Protein canopy homolog 4	4.51	4.62	29.4	3	1.103
sp|Q96QU8|XPO6_HUMAN	XPO6	Exportin-6	11.23	11.39	22.2	6	1.103
sp|O96007|MOC2B_HUMAN	MOCS2	Molybdopterin synthase catalytic subunit	5.71	5.78	34.6	3	1.104
sp|P48651|PTSS1_HUMAN	PTDSS1	Phosphatidylserine synthase 1	4.07	4.29	15.4	3	1.104
sp|O15091|MRRP3_HUMAN	KIAA0391	Mitochondrial ribonuclease P protein 3	17.14	19.52	38.4	11	1.104
sp|Q6UVJ0|SAS6_HUMAN	SASS6	Spindle assembly abnormal protein 6 homolog	2.42	2.58	27.9	3	1.104
sp|Q8WUH2|TGFA1_HUMAN	TGFBRAP1	Transforming growth factor-beta receptor-associated protein 1	4.69	4.8	18.8	4	1.104

sp|Q9Y3Z3|SAMH1_HUMAN	SAMHD1	Deoxynucleoside triphosphate triphosphohydrolase SAMHD1	47.79	47.87	54.3	28	1.104
sp|Q8TBR7|FA57A_HUMAN	FAM57A	Protein FAM57A	2.64	2.67	14.4	2	1.104
sp|Q9NZN8|CNOT2_HUMAN	CNOT2	CCR4-NOT transcription complex subunit 2	14.65	14.78	26.9	9	1.105
sp|O14531|DPYL4_HUMAN	DPYSL4	Dihydropyrimidinase-related protein 4	4.08	5.91	21.7	4	1.105
sp|Q9H0R1|AP5M1_HUMAN	AP5M1	AP-5 complex subunit mu-1	3.1	3.17	15.1	2	1.105
sp|Q9Y5L4|TIM13_HUMAN	TIMM13	Mitochondrial import inner membrane translocase subunit Tim13	11.73	11.88	79	14	1.105
sp|Q96EL3|RM53_HUMAN	MRPL53	39S ribosomal protein L53, mitochondrial	8.48	8.51	67	6	1.105
sp|Q5VTU8|AT5EL_HUMAN	ATP5EP2	ATP synthase subunit epsilon-like protein, mitochondrial	5.39	5.46	54.9	3	1.105
sp|Q96NT0|CC115_HUMAN	CCDC115	Coiled-coil domain-containing protein 115	6.14	6.29	40	4	1.105
sp|O43676|NDUB3_HUMAN	NDUFB3	NADH dehydrogenase [ubiquinone] 1 beta subcomplex subunit 3	4.06	4.18	30.6	3	1.105
sp|P01031|CO5_HUMAN	C5	Complement C5	2.74	2.93	16.7	7	1.105
sp|Q9UKK6|NXT1_HUMAN	NXT1	NTF2-related export protein 1	6.31	6.33	31.4	4	1.105
sp|Q86TN4|TRPT1_HUMAN	TRPT1	tRNA 2'-phosphotransferase 1	4.01	4.02	28.9	3	1.106
sp|Q8WUZ0|BCL7C_HUMAN	BCL7C	B-cell CLL/lymphoma 7 protein family member C	6.09	6.1	44.2	3	1.106
sp|P08069|IGF1R_HUMAN	IGF1R	Insulin-like growth factor 1 receptor	16.63	17.78	22	12	1.106
sp|Q92841|DDX17_HUMAN	DDX17	Probable ATP-dependent RNA helicase DDX17	87.57	88.97	71.1	74	1.106
sp|Q9P0T7|TMEM9_HUMAN	TMEM9	Transmembrane protein 9	3.96	4	30.1	2	1.106
sp|Q8N5V2|NGEF_HUMAN	NGEF	Ephexin-1	4.01	4.03	18.9	2	1.106
sp|P50479|PDLI4_HUMAN	PDLIM4	PDZ and LIM domain protein 4	2.98	3.05	14.6	2	1.106
sp|Q13123|RED_HUMAN	IK	Protein Red	26.82	29.06	48.3	16	1.106
sp|Q16540|RM23_HUMAN	MRPL23	39S ribosomal protein L23, mitochondrial	11.34	11.57	81.1	9	1.106
sp|P15336|ATF2_HUMAN	ATF2	Cyclic AMP-dependent transcription factor ATF-2	4.33	4.36	16	4	1.107
sp|Q9H0B6|KLC2_HUMAN	KLC2	Kinesin light chain 2	43.09	43.73	55.5	23	1.107
sp|Q9NPD3|EXOS4_HUMAN	EXOSC4	Exosome complex component RRP41	16.5	16.52	44.5	11	1.107
sp|Q9NZJ4|SACS_HUMAN	SACS	Sacsin	14.16	19.58	17.8	14	1.107
sp|Q9Y3X0|CCDC9_HUMAN	CCDC9	Coiled-coil domain-containing protein 9	4.18	4.39	29.4	5	1.107
sp|P82673|RT35_HUMAN	MRPS35	28S ribosomal protein S35, mitochondrial	21.42	21.81	59.4	14	1.107
sp|O14981|BTAF1_HUMAN	BTAF1	TATA-binding protein-associated factor 172	30.69	32.72	27.4	17	1.107
sp|O15431|COPT1_HUMAN	SLC31A1	High affinity copper uptake protein 1	2	2.01	14.2	3	1.107
sp|Q5VT06|CE350_HUMAN	CEP350	Centrosome-associated protein 350	4.23	7.28	16.5	9	1.107
sp|O43684|BUB3_HUMAN	BUB3	Mitotic checkpoint protein BUB3	23.5	23.72	57.3	21	1.108
sp|P25705|ATPA_HUMAN	ATP5A1	ATP synthase subunit alpha, mitochondrial	103.78	105.63	84.3	144	1.108
sp|Q9H6Y7|RN167_HUMAN	RNF167	E3 ubiquitin-protein ligase RNF167	4.85	4.94	24.3	4	1.108
sp|Q9Y678|COPG1_HUMAN	COPG1	Coatomer subunit gamma-1	76.49	76.63	70	57	1.108
sp|O95081|AGFG2_HUMAN	AGFG2	Arf-GAP domain and FG repeat-containing protein 2	6.91	8.13	19.8	6	1.109
sp|Q4G0N4|NAKD2_HUMAN	NADK2	NAD kinase 2, mitochondrial	27.61	29.28	56.8	18	1.109
sp|Q7Z4W1|DCXR_HUMAN	DCXR	L-xylulose reductase	22.18	22.21	76.6	16	1.109
sp|Q92925|SMRD2_HUMAN	SMARCD2	SWI/SNF-related matrix-associated actin-dependent regulator of chromatin subfamily D member 2	14.44	16.89	37.3	11	1.109
sp|Q8IWL3|HSC20_HUMAN	HSCB	Iron-sulfur cluster co-chaperone protein HscB, mitochondrial	6.23	6.39	34.9	7	1.109
sp|Q9BYD1|RM13_HUMAN	MRPL13	39S ribosomal protein L13, mitochondrial	21.23	21.3	80.9	13	1.110
sp|O95801|TTC4_HUMAN	TTC4	Tetratricopeptide repeat protein 4	21.16	21.3	60	11	1.110
sp|P61011|SRP54_HUMAN	SRP54	Signal recognition particle 54 kDa protein	53.08	53.28	63.9	37	1.110
sp|A4D1P6|WDR91_HUMAN	WDR91	WD repeat-containing protein 91	2.71	3.01	18.2	3	1.110
sp|Q8WTT2|NOC3L_HUMAN	NOC3L	Nucleolar complex protein 3 homolog	47.55	48.32	64.3	30	1.111
sp|Q96CQ1|S2536_HUMAN	SLC25A36	Solute carrier family 25 member 36	4	4.48	23.8	3	1.111
sp|P07307|ASGR2_HUMAN	ASGR2	Asialoglycoprotein receptor 2	11.22	11.94	51.1	7	1.111
sp|Q9H4A3|WNK1_HUMAN	WNK1	Serine/threonine-protein kinase WNK1	27.13	28.55	14.4	16	1.111
sp|P14406|CX7A2_HUMAN	COX7A2	Cytochrome c oxidase subunit 7A2, mitochondrial	4	4	47	3	1.111
sp|P49711|CTCF_HUMAN	CTCF	Transcriptional repressor CTCF	32.44	33.46	55.6	23	1.111

sp|Q96GK7|FAH2A_HUMAN	FAHD2A	Fumarylacetoacetate hydrolase domain-containing protein 2A	12.11	12.27	41.1	7	1.111
sp|P11274|BCR_HUMAN	BCR	Breakpoint cluster region protein	27.24	29.1	31.6	16	1.111
sp|Q99714|HCD2_HUMAN	HSD17B10	3-hydroxyacyl-CoA dehydrogenase type-2	34.75	34.85	96.9	44	1.111
sp|O00291|HIP1_HUMAN	HIP1	Huntingtin-interacting protein 1	10.31	12.93	31.9	8	1.111
sp|Q9HAP2|MLXIP_HUMAN	MLXIP	MLX-interacting protein	2.03	2.06	12.4	2	1.111
sp|P26374|RAE2_HUMAN	CHML	Rab proteins geranylgeranyltransferase component A 2	1.78	2.21	18.6	3	1.111
sp|Q96N66|MBOA7_HUMAN	MBOAT7	Lysophospholipid acyltransferase 7	11.38	11.52	24.6	6	1.112
sp|O15127|SCAM2_HUMAN	SCAMP2	Secretory carrier-associated membrane protein 2	6.39	6.43	21.6	7	1.112
sp|P30622|CLIP1_HUMAN	CLIP1	CAP-Gly domain-containing linker protein 1	37.31	38.69	44.9	21	1.112
sp|O94966|UBP19_HUMAN	USP19	Ubiquitin carboxyl-terminal hydrolase 19	38.86	38.98	32.6	19	1.112
sp|Q68CZ2|TENS3_HUMAN	TNS3	Tensin-3	17.63	17.92	22	11	1.112
sp|Q6PD62|CTR9_HUMAN	CTR9	RNA polymerase-associated protein CTR9 homolog	34.81	35.34	35.6	23	1.113
sp|Q92541|RTF1_HUMAN	RTF1	RNA polymerase-associated protein RTF1 homolog	28.3	28.78	41.1	15	1.113
sp|Q9NQ29|LUC7L_HUMAN	LUC7L	Putative RNA-binding protein Luc7-like 1	13.1	25.77	47.4	15	1.113
sp|P19387|RPB3_HUMAN	POLR2C	DNA-directed RNA polymerase II subunit RPB3	19.58	19.66	57.5	17	1.113
sp|P28331|NDUS1_HUMAN	NDUFS1	NADH-ubiquinone oxidoreductase 75 kDa subunit, mitochondrial	63.38	63.79	78.3	52	1.113
sp|Q9Y385|UB2J1_HUMAN	UBE2J1	Ubiquitin-conjugating enzyme E2 J1	3.28	3.35	15.1	2	1.113
sp|P49754|VPS41_HUMAN	VPS41	Vacuolar protein sorting-associated protein 41 homolog	4.02	4.05	16.2	2	1.113
sp|P55327|TPD52_HUMAN	TPD52	Tumor protein D52	15.29	15.67	64.7	13	1.113
sp|O00443|P3C2A_HUMAN	PIK3C2A	Phosphatidylinositol 4-phosphate 3-kinase C2 domain-containing subunit alpha	26.76	29.01	25.8	17	1.113
sp|Q9Y287|ITM2B_HUMAN	ITM2B	Integral membrane protein 2B	3.39	3.47	33.5	2	1.113
sp|Q9Y2X0|MED16_HUMAN	MED16	Mediator of RNA polymerase II transcription subunit 16	8.18	8.63	20.4	7	1.113
sp|P53778|MK12_HUMAN	MAPK12	Mitogen-activated protein kinase 12	4	6.06	30.3	3	1.114
sp|Q6UW63|KDEL1_HUMAN	KDELC1	KDEL motif-containing protein 1	11.58	12.37	34.9	8	1.114
sp|Q9BQ52|RNZ2_HUMAN	ELAC2	Zinc phosphodiesterase ELAC protein 2	51.05	51.9	64.9	34	1.114
sp|P99999|CYC_HUMAN	CYCS	Cytochrome c	18.59	18.88	68.6	26	1.114
sp|Q9P2B2|FPRP_HUMAN	PTGFRN	Prostaglandin F2 receptor negative regulator	24.98	25.28	33.9	14	1.114
sp|P19447|ERCC3_HUMAN	ERCC3	TFIIH basal transcription factor complex helicase XPB subunit	38.7	38.78	45.1	22	1.115
sp|Q96JP5|ZFP91_HUMAN	ZFP91	E3 ubiquitin-protein ligase ZFP91	2.84	3.26	23	4	1.115
sp|Q9BTE7|DCNL5_HUMAN	DCUN1D5	DCN1-like protein 5	17.27	17.56	71.3	9	1.115
sp|Q9H4H8|FA83D_HUMAN	FAM83D	Protein FAM83D	2.34	2.36	19.2	2	1.115
sp|P07947|YES_HUMAN	YES1	Tyrosine-protein kinase Yes	8.98	21.15	37.9	13	1.115
sp|O15321|TM9S1_HUMAN	TM9SF1	Transmembrane 9 superfamily member 1	11.77	11.89	24.8	7	1.115
sp|Q6DKJ4|NXN_HUMAN	NXN	Nucleoredoxin	24.37	24.5	49.7	13	1.115
sp|Q53FA7|QORX_HUMAN	TP53I3	Quinone oxidoreductase PIG3	12.19	12.44	39.5	8	1.115
sp|Q86YR5|GPSM1_HUMAN	GPSM1	G-protein-signaling modulator 1	8.03	8.04	15.4	4	1.115
sp|Q9H0K6|PUS7L_HUMAN	PUS7L	Pseudouridylate synthase 7 homolog-like protein	2.03	2.11	20.7	2	1.115
sp|Q08495|DEMA_HUMAN	DMTN	Dematin	3.3	3.48	16.1	2	1.116
sp|P17301|ITA2_HUMAN	ITGA2	Integrin alpha-2	43.98	44.56	37.7	27	1.116
sp|Q08334|I10R2_HUMAN	IL10RB	Interleukin-10 receptor subunit beta	1.62	2.51	12.6	3	1.116
sp|Q14746|COG2_HUMAN	COG2	Conserved oligomeric Golgi complex subunit 2	14.06	14.25	32.3	10	1.116
sp|Q7L5Y9|MAEA_HUMAN	MAEA	Macrophage erythroblast attacher	6.52	6.88	24.2	6	1.117
sp|Q8IVL6|P3H3_HUMAN	P3H3	Prolyl 3-hydroxylase 3	30.48	30.57	42.1	18	1.117
sp|P19174|PLCG1_HUMAN	PLCG1	1-phosphatidylinositol 4,5-bisphosphate phosphodiesterase gamma-1	54.32	54.82	47	32	1.117
sp|Q3B7T1|EDRF1_HUMAN	EDRF1	Erythroid differentiation-related factor 1	2.13	2.27	20.5	2	1.118
sp|O14929|HAT1_HUMAN	HAT1	Histone acetyltransferase type B catalytic subunit	27.47	29.26	60.9	24	1.118
sp|P30086|PEBP1_HUMAN	PEBP1	Phosphatidylethanolamine-binding protein 1	33.94	34.65	88.8	62	1.118
sp|Q9UJF2|NGAP_HUMAN	RASAL2	Ras GTPase-activating protein nGAP	2.16	5.99	16.1	5	1.118
sp|Q96JM3|CHAP1_HUMAN	CHAMP1	Chromosome alignment-maintaining phosphoprotein 1	31.01	31.15	43.8	17	1.118

sp|Q8N954|GPT11_HUMAN	GPATCH11	G patch domain-containing protein 11	5.92	6.22	25.5	3	1.118
sp|O95881|TXD12_HUMAN	TXNDC12	Thioredoxin domain-containing protein 12	14.47	16.33	73.8	14	1.119
sp|P12830|CADH1_HUMAN	CDH1	Cadherin-1	1.31	2.08	10.7	7	1.119
sp|Q9Y2U8|MAN1_HUMAN	LEMD3	Inner nuclear membrane protein Man1	25.83	26.61	36.3	14	1.119
sp|Q9Y624|JAM1_HUMAN	F11R	Junctional adhesion molecule A	12.87	13.05	44.2	8	1.119
sp|Q96RQ1|ERGI2_HUMAN	ERGIC2	Endoplasmic reticulum-Golgi intermediate compartment protein 2	10.05	10.06	24.7	6	1.119
sp|P54709|AT1B3_HUMAN	ATP1B3	Sodium/potassium-transporting ATPase subunit beta-3	32.49	32.78	69.2	27	1.119
sp|C9JLW8|F195B_HUMAN	FAM195B	Protein FAM195B	4.66	5.25	39.2	4	1.119
sp|P51530|DNA2_HUMAN	DNA2	DNA replication ATP-dependent helicase/nuclease DNA2	4.49	6.1	22.3	4	1.119
sp|O95183|VAMP5_HUMAN	VAMP5	Vesicle-associated membrane protein 5	1.87	2.25	37.9	2	1.119
sp|Q96RU2|UBP28_HUMAN	USP28	Ubiquitin carboxyl-terminal hydrolase 28	13.83	14.43	23.5	13	1.119
sp|P10589|COT1_HUMAN	NR2F1	COUP transcription factor 1	2.01	7.81	23.2	4	1.119
sp|Q9BYC8|RM32_HUMAN	MRPL32	39S ribosomal protein L32, mitochondrial	6.01	6.09	31.9	4	1.119
sp|P50148|GNAQ_HUMAN	GNAQ	Guanine nucleotide-binding protein G(q) subunit alpha	9.87	14.72	44	11	1.119
sp|Q2TAY7|SMU1_HUMAN	SMU1	WD40 repeat-containing protein SMU1	41.6	43.75	64.3	29	1.119
sp|O43148|MCES_HUMAN	RNMT	mRNA cap guanine-N7 methyltransferase	14.98	15.15	43.1	10	1.119
sp|Q5VW38|GP107_HUMAN	GPR107	Protein GPR107	5.01	7.54	23.5	5	1.120
sp|Q9UL18|AGO1_HUMAN	AGO1	Protein argonaute-1	10.37	18.65	33.5	11	1.120
sp|P17900|SAP3_HUMAN	GM2A	Ganglioside GM2 activator	6.64	6.73	54.9	5	1.120
sp|P55212|CASP6_HUMAN	CASP6	Caspase-6	6.49	8.89	42.3	6	1.120
sp|Q9NS69|TOM22_HUMAN	TOMM22	Mitochondrial import receptor subunit TOM22 homolog	14.03	14.03	64.8	19	1.120
sp|Q9H0C5|BTBD1_HUMAN	BTBD1	BTB/POZ domain-containing protein 1	4	4.12	16.2	2	1.121
sp|P59780|AP3S2_HUMAN	AP3S2	AP-3 complex subunit sigma-2	2.07	3.75	27.5	2	1.121
sp|Q9NSV4|DIAP3_HUMAN	DIAPH3	Protein diaphanous homolog 3	8.63	10.65	26.9	9	1.121
sp|Q13627|DYR1A_HUMAN	DYRK1A	Dual specificity tyrosine-phosphorylation-regulated kinase 1A	4.53	4.86	15.9	3	1.121
sp|P57740|NU107_HUMAN	NUP107	Nuclear pore complex protein Nup107	41.87	44.14	53.5	29	1.121
sp|A6ZKI3|F127A_HUMAN	FAM127A	Protein FAM127A	2.37	2.89	36.3	2	1.121
sp|P23368|MAOM_HUMAN	ME2	NAD-dependent malic enzyme, mitochondrial	43.41	43.75	62.7	33	1.121
sp|Q12981|SEC20_HUMAN	BNIP1	Vesicle transport protein SEC20	13.17	13.6	64	9	1.121
sp|Q05DH4|F16A1_HUMAN	FAM160A1	Protein FAM160A1	1.85	2.1	12.3	3	1.121
sp|P35218|CAH5A_HUMAN	CA5A	Carbonic anhydrase 5A, mitochondrial	5.49	5.61	20.7	4	1.122
sp|P46777|RL5_HUMAN	RPL5	60S ribosomal protein L5	39.89	40.11	72.1	55	1.122
sp|P32929|CGL_HUMAN	CTH	Cystathionine gamma-lyase	23.67	23.86	59.5	16	1.122
sp|Q9UNF0|PACN2_HUMAN	PACSIN2	Protein kinase C and casein kinase substrate in neurons protein 2	41.35	41.38	53.9	26	1.122
sp|Q9BUF5|TBB6_HUMAN	TUBB6	Tubulin beta-6 chain	26.57	68.69	78	157	1.122
sp|Q7KZN9|COX15_HUMAN	COX15	Cytochrome c oxidase assembly protein COX15 homolog	11.2	11.79	31.2	9	1.123
sp|Q96JA1|LRIG1_HUMAN	LRIG1	Leucine-rich repeats and immunoglobulin-like domains protein 1	2.01	2.12	9.8	3	1.123
sp|Q05932|FOLC_HUMAN	FPGS	Folylpolyglutamate synthase, mitochondrial	4.39	4.45	14.8	4	1.123
sp|O60831|PRAF2_HUMAN	PRAF2	PRA1 family protein 2	4	4	25.8	2	1.123
sp|Q9ULR3|PPM1H_HUMAN	PPM1H	Protein phosphatase 1H	4.81	4.9	25.1	3	1.124
sp|P62136|PP1A_HUMAN	PPP1CA	Serine/threonine-protein phosphatase PP1-alpha catalytic subunit	41.1	41.36	79.4	37	1.124
sp|P49454|CENPF_HUMAN	CENPF	Centromere protein F	75.11	79.32	38.9	49	1.124
sp|Q68EM7|RHG17_HUMAN	ARHGAP17	Rho GTPase-activating protein 17	26.81	27.56	42.3	14	1.125
sp|Q9HCE1|MOV10_HUMAN	MOV10	Putative helicase MOV-10	54.39	54.7	54.8	30	1.125
sp|P13995|MTDC_HUMAN	MTHFD2	Bifunctional methylenetetrahydrofolate dehydrogenase/cyclohydrolase, mitochondrial	31.72	32.55	75.7	27	1.126
sp|P54274|TERF1_HUMAN	TERF1	Telomeric repeat-binding factor 1	2.35	2.43	22.8	3	1.126
sp|Q969G3|SMCE1_HUMAN	SMARCE1	SWI/SNF-related matrix-associated actin-dependent regulator of chromatin subfamily E member 1	14.27	16.91	45.3	12	1.126
sp|Q8IXQ4|GPAM1_HUMAN	GPALPP1	GPALPP motifs-containing protein 1	4.09	4.11	16.5	3	1.126
sp|Q9UG63|ABCF2_HUMAN	ABCF2	ATP-binding cassette sub-family F member 2	37.47	39.58	51.5	21	1.127

sp|Q8NAN2|FA73A_HUMAN	FAM73A	Protein FAM73A	2.32	2.36	12.8	2	1.127
sp|O75064|DEN4B_HUMAN	DENND4B	DENN domain-containing protein 4B	3.21	3.3	8.8	4	1.127
sp|P49674|KC1E_HUMAN	CSNK1E	Casein kinase I isoform epsilon	19.89	20.46	42.1	13	1.127
sp|Q7Z6E9|RBBP6_HUMAN	RBBP6	E3 ubiquitin-protein ligase RBBP6	19.87	22.09	20.9	16	1.127
sp|P13796|PLSL_HUMAN	LCP1	Plastin-2	5.54	20.73	40.5	13	1.127
sp|P35221|CTNA1_HUMAN	CTNNA1	Catenin alpha-1	63.75	64.63	62.9	41	1.127
sp|Q9NUQ2|PLCE_HUMAN	AGPAT5	1-acyl-sn-glycerol-3-phosphate acyltransferase epsilon	9.65	9.85	40.7	5	1.127
sp|P18084|ITB5_HUMAN	ITGB5	Integrin beta-5	10.31	10.37	23.2	7	1.127
sp|Q9BWH6|RPAP1_HUMAN	RPAP1	RNA polymerase II-associated protein 1	23.42	23.77	25.8	14	1.127
sp|Q9NXC5|MIO_HUMAN	MIOS	WD repeat-containing protein mio	20.82	22.22	27.3	13	1.127
sp|Q9UH65|SWP70_HUMAN	SWAP70	Switch-associated protein 70	20.11	21.85	44.8	14	1.127
sp|Q9Y3D2|MSRB2_HUMAN	MSRB2	Methionine-R-sulfoxide reductase B2, mitochondrial	2.94	3.03	31.3	2	1.128
sp|Q9NRY5|F1142_HUMAN	FAM114A2	Protein FAM114A2	21.41	21.54	50.1	14	1.128
sp|Q15047|SETB1_HUMAN	SETDB1	Histone-lysine N-methyltransferase SETDB1	3.42	3.52	10.8	2	1.128
sp|Q8N6S5|AR6P6_HUMAN	ARL6IP6	ADP-ribosylation factor-like protein 6-interacting protein 6	5.11	5.29	37.6	4	1.128
sp|Q7Z4S6|KI21A_HUMAN	KIF21A	Kinesin-like protein KIF21A	30.98	32.26	33.2	19	1.128
sp|Q7Z736|PKHH3_HUMAN	PLEKHH3	Pleckstrin homology domain-containing family H member 3	4	4.02	15.9	2	1.129
sp|Q8WVB6|CTF18_HUMAN	CHTF18	Chromosome transmission fidelity protein 18 homolog	15.03	17.29	27.2	11	1.129
sp|Q8TCJ2|STT3B_HUMAN	STT3B	Dolichyl-diphosphooligosaccharide--protein glycosyltransferase subunit STT3B	17.16	21.25	24.5	17	1.129
sp|Q6NSI4|CX057_HUMAN	CXorf57	Uncharacterized protein CXorf57	19.76	19.96	30.1	11	1.129
sp|Q96A73|P33MX_HUMAN	KIAA1191	Putative monooxygenase p33MONOX	2.01	2.03	6.2	3	1.129
sp|Q9H089|LSG1_HUMAN	LSG1	Large subunit GTPase 1 homolog	18.82	19.4	37.1	11	1.129
sp|Q13823|NOG2_HUMAN	GNL2	Nucleolar GTP-binding protein 2	25.64	26.61	39.5	18	1.129
sp|Q09666|AHNK_HUMAN	AHNAK	Neuroblast differentiation-associated protein AHNAK	322.48	322.3	72.6	167	1.129
sp|Q12986|NFX1_HUMAN	NFX1	Transcriptional repressor NF-X1	3.09	3.19	11.2	3	1.129
sp|Q53HL2|BOREA_HUMAN	CDCA8	Borealin	15.37	15.76	50.7	11	1.129
sp|P50213|IDH3A_HUMAN	IDH3A	Isocitrate dehydrogenase [NAD] subunit alpha, mitochondrial	29.58	31.67	65	21	1.129
sp|O15381|NVL_HUMAN	NVL	Nuclear valosin-containing protein-like	53.18	57.97	52	34	1.129
sp|P15374|UCHL3_HUMAN	UCHL3	Ubiquitin carboxyl-terminal hydrolase isozyme L3	11.64	11.87	70.4	7	1.130
sp|Q15750|TAB1_HUMAN	TAB1	TGF-beta-activated kinase 1 and MAP3K7-binding protein 1	18.17	18.29	38.1	10	1.130
sp|Q9UPU5|UBP24_HUMAN	USP24	Ubiquitin carboxyl-terminal hydrolase 24	51.41	52.9	29.6	30	1.130
sp|O43663|PRC1_HUMAN	PRC1	Protein regulator of cytokinesis 1	29.25	32.52	57.6	19	1.130
sp|Q92945|FUBP2_HUMAN	KHSRP	Far upstream element-binding protein 2	74.81	78.03	82.8	69	1.130
sp|P51649|SSDH_HUMAN	ALDH5A1	Succinate-semialdehyde dehydrogenase, mitochondrial	42.72	43.06	74.6	32	1.130
sp|Q9Y5U2|TSSC4_HUMAN	TSSC4	Protein TSSC4	3.12	3.2	9.7	2	1.130
sp|P02749|APOH_HUMAN	APOH	Beta-2-glycoprotein 1	11.19	11.55	41.5	6	1.131
sp|Q5T280|CI114_HUMAN	C9orf114	Putative methyltransferase C9orf114	12.4	12.53	37.5	8	1.131
sp|P17812|PYRG1_HUMAN	CTPS1	CTP synthase 1	48.36	49.13	58.7	37	1.131
sp|Q14139|UBE4A_HUMAN	UBE4A	Ubiquitin conjugation factor E4 A	19.63	19.81	28.8	15	1.131
sp|P60900|PSA6_HUMAN	PSMA6	Proteasome subunit alpha type-6	35.77	35.85	74	36	1.131
sp|Q9NRW1|RAB6B_HUMAN	RAB6B	Ras-related protein Rab-6B	7.4	15.32	67.3	12	1.131
sp|Q9C0C2|TB182_HUMAN	TNKS1BP1	182 kDa tankyrase-1-binding protein	60.61	60.71	39.4	32	1.132
sp|Q9BZL1|UBL5_HUMAN	UBL5	Ubiquitin-like protein 5	4.16	4.18	57.5	3	1.132
sp|Q99700|ATX2_HUMAN	ATXN2	Ataxin-2	22.43	23.26	22.2	14	1.132
sp|Q9BYI3|HYCCI_HUMAN	FAM126A	Hyccin	4.01	4.01	13.8	2	1.132
sp|Q9UF12|PROD2_HUMAN	PRODH2	Probable proline dehydrogenase 2	6.06	6.12	17.2	4	1.132
sp|Q15233|NONO_HUMAN	NONO	Non-POU domain-containing octamer-binding protein	63.45	67.48	69.6	99	1.132
sp|Q9BXB5|OSB10_HUMAN	OSBPL10	Oxysterol-binding protein-related protein 10	1.89	4.82	14.3	3	1.132
sp|Q16555|DPYL2_HUMAN	DPYSL2	Dihydropyrimidinase-related protein 2	46.11	46.32	78.2	45	1.133

sp|Q9NVA2|SEP11_HUMAN	SEPT11	Septin-11	29.43	32.59	68.5	24	1.133
sp|Q9P275|UBP36_HUMAN	USP36	Ubiquitin carboxyl-terminal hydrolase 36	23.02	23.79	29.8	15	1.133
sp|Q68DK7|MSL1_HUMAN	MSL1	Male-specific lethal 1 homolog	6.75	9.05	23.8	4	1.133
sp|Q9NWZ5|UCKL1_HUMAN	UCKL1	Uridine-cytidine kinase-like 1	3.44	3.56	16.4	4	1.133
sp|P84157|MXRA7_HUMAN	MXRA7	Matrix-remodeling-associated protein 7	9.51	9.66	44.1	6	1.133
sp|O75461|E2F6_HUMAN	E2F6	Transcription factor E2F6	4.03	4.03	26.7	2	1.134
sp|Q92616|GCN1L_HUMAN	GCN1L1	Translational activator GCN1	203.06	204.06	56.8	131	1.134
sp|Q9Y6I9|TX264_HUMAN	TEX264	Testis-expressed sequence 264 protein	17.55	17.64	41.5	12	1.134
sp|Q9P219|DAPLE_HUMAN	CCDC88C	Protein Daple	2.26	2.6	19.8	3	1.134
sp|O95639|CPSF4_HUMAN	CPSF4	Cleavage and polyadenylation specificity factor subunit 4	10.7	10.76	50.6	8	1.134
sp|Q8NC96|NECP1_HUMAN	NECAP1	Adaptin ear-binding coat-associated protein 1	13.44	13.58	50.9	8	1.135
sp|Q8N201|INT1_HUMAN	INTS1	Integrator complex subunit 1	40.73	41.53	29.3	24	1.135
sp|Q9NSI2|F207A_HUMAN	FAM207A	Protein FAM207A	19.96	20.77	62.6	14	1.135
sp|Q8N3Y1|FBXW8_HUMAN	FBXW8	F-box/WD repeat-containing protein 8	5	5.06	13.6	4	1.135
sp|P51610|HCFC1_HUMAN	HCFC1	Host cell factor 1	82.35	82.44	40.2	55	1.135
sp|Q8IY17|PLPL6_HUMAN	PNPLA6	Neuropathy target esterase	13.98	14.17	18.9	8	1.135
sp|Q8N8L6|ARL10_HUMAN	ARL10	ADP-ribosylation factor-like protein 10	2.5	2.8	25	3	1.135
sp|Q96SY0|VWA9_HUMAN	VWA9	von Willebrand factor A domain-containing protein 9	9.16	9.25	24.7	8	1.136
sp|P30042|ES1_HUMAN	C21orf33	ES1 protein homolog, mitochondrial	22.05	22.18	70.2	16	1.136
sp|P52948|NUP98_HUMAN	NUP98	Nuclear pore complex protein Nup98-Nup96	77.79	78.26	37.7	46	1.136
sp|Q9GZQ3|COMD5_HUMAN	COMMD5	COMM domain-containing protein 5	5.4	5.72	34.8	3	1.136
sp|Q9HC35|EMAL4_HUMAN	EML4	Echinoderm microtubule-associated protein-like 4	98.81	98.87	62.4	73	1.136
sp|Q9BU61|NDUF3_HUMAN	NDUFAF3	NADH dehydrogenase [ubiquinone] 1 alpha subcomplex assembly factor 3	14.27	14.41	58.2	11	1.136
sp|P09543|CN37_HUMAN	CNP	2',3'-cyclic-nucleotide 3'-phosphodiesterase	51.15	54.12	68.7	36	1.136
sp|Q7Z6J0|SH3R1_HUMAN	SH3RF1	E3 ubiquitin-protein ligase SH3RF1	3.37	3.63	15.5	4	1.136
sp|Q96C19|EFHD2_HUMAN	EFHD2	EF-hand domain-containing protein D2	19.61	21.79	55	12	1.136
sp|P51788|CLCN2_HUMAN	CLCN2	Chloride channel protein 2	6.12	6.15	17.7	3	1.136
sp|Q9Y5M8|SRPRB_HUMAN	SRPRB	Signal recognition particle receptor subunit beta	29.5	30.37	67.2	22	1.137
sp|P22681|CBL_HUMAN	CBL	E3 ubiquitin-protein ligase CBL	11.34	11.5	20.9	7	1.137
sp|Q96GD0|PLPP_HUMAN	PDXP	Pyridoxal phosphate phosphatase	12.81	12.88	44.9	8	1.137
sp|P29375|KDM5A_HUMAN	KDM5A	Lysine-specific demethylase 5A	1.92	3.16	14.5	6	1.137
sp|Q6ZXV5|TMTC3_HUMAN	TMTC3	Transmembrane and TPR repeat-containing protein 3	18.28	21.86	33.9	19	1.137
sp|Q7Z3K3|POGZ_HUMAN	POGZ	Pogo transposable element with ZNF domain	45.35	46.08	35.1	31	1.137
sp|Q9Y613|FHOD1_HUMAN	FHOD1	FH1/FH2 domain-containing protein 1	42.63	42.85	45.3	22	1.137
sp|Q3SY69|AL1L2_HUMAN	ALDH1L2	Mitochondrial 10-formyltetrahydrofolate dehydrogenase	53.43	53.64	63.6	28	1.137
sp|Q8NET6|CHSTD_HUMAN	CHST13	Carbohydrate sulfotransferase 13	1.41	1.59	15.5	3	1.137
sp|P05556|ITB1_HUMAN	ITGB1	Integrin beta-1	44.54	44.93	43	29	1.137
sp|Q9H147|TDIF1_HUMAN	DNTTIP1	Deoxynucleotidyltransferase terminal-interacting protein 1	9.06	9.6	40.1	5	1.138
sp|Q9Y6M9|NDUB9_HUMAN	NDUFB9	NADH dehydrogenase [ubiquinone] 1 beta subcomplex subunit 9	15.84	16.28	76.5	13	1.138
sp|Q9H5N1|RABE2_HUMAN	RABEP2	Rab GTPase-binding effector protein 2	7.46	10.34	33	8	1.138
sp|Q5J8M3|EMC4_HUMAN	EMC4	ER membrane protein complex subunit 4	9.1	9.22	45.9	9	1.138
sp|P02679|FIBG_HUMAN	FGG	Fibrinogen gamma chain	22.08	23.37	57.4	20	1.138
sp|Q12765|SCRN1_HUMAN	SCRN1	Secernin-1	24.94	25.04	58	14	1.138
sp|Q13685|AAMP_HUMAN	AAMP	Angio-associated migratory cell protein	21.54	22.48	47.5	15	1.138
sp|P00966|ASSY_HUMAN	ASS1	Argininosuccinate synthase	22.64	22.79	66.3	15	1.138
sp|Q14008|CKAP5_HUMAN	CKAP5	Cytoskeleton-associated protein 5	124.86	127.88	52.3	81	1.138
sp|O75970|MPDZ_HUMAN	MPDZ	Multiple PDZ domain protein	12.33	13.98	18.8	8	1.138
sp|Q9Y666|S12A7_HUMAN	SLC12A7	Solute carrier family 12 member 7	8.88	9.29	21.4	7	1.138
sp|O00629|IMA3_HUMAN	KPNA4	Importin subunit alpha-3	26.27	26.87	53.9	22	1.139

sp|P78347|GTF2I_HUMAN	GTF2I	General transcription factor II-I	99.6	100.87	68.9	75	1.139
sp|O95396|MOCS3_HUMAN	MOCS3	Adenylyltransferase and sulfurtransferase MOCS3	19.82	19.99	51.1	10	1.139
sp|Q96BN8|OTUL_HUMAN	OTULIN	Ubiquitin thioesterase otulin	7.23	7.32	27	5	1.139
sp|Q96AJ9|VTI1A_HUMAN	VTI1A	Vesicle transport through interaction with t-SNAREs homolog 1A	2.79	2.87	33.6	3	1.139
sp|Q15024|EXOS7_HUMAN	EXOSC7	Exosome complex component RRP42	23.62	23.68	65	15	1.139
sp|P42773|CDN2C_HUMAN	CDKN2C	Cyclin-dependent kinase 4 inhibitor C	2.66	2.73	34.5	2	1.140
sp|Q4G148|GXLT1_HUMAN	GXYLT1	Glucoside xylosyltransferase 1	7.35	7.6	22.1	6	1.140
sp|P42356|PI4KA_HUMAN	PI4KA	Phosphatidylinositol 4-kinase alpha	27.21	27.76	25.3	17	1.140
sp|Q04323|UBXN1_HUMAN	UBXN1	UBX domain-containing protein 1	18.56	18.72	71	12	1.140
sp|Q16204|CCDC6_HUMAN	CCDC6	Coiled-coil domain-containing protein 6	21.63	21.82	42.8	11	1.140
sp|O60826|CCD22_HUMAN	CCDC22	Coiled-coil domain-containing protein 22	29.48	29.65	47.4	16	1.140
sp|Q13557|KCC2D_HUMAN	CAMK2D	Calcium/calmodulin-dependent protein kinase type II subunit delta	23.17	24.78	55.5	19	1.140
sp|Q9BZF9|UACA_HUMAN	UACA	Uveal autoantigen with coiled-coil domains and ankyrin repeats	11.02	15.59	33.8	12	1.140
sp|Q9Y263|PLAP_HUMAN	PLAA	Phospholipase A-2-activating protein	43.03	48.69	56.2	27	1.141
sp|P42765|THIM_HUMAN	ACAA2	3-ketoacyl-CoA thiolase, mitochondrial	46.77	47.28	80.4	45	1.141
sp|Q6NXE6|ARMC6_HUMAN	ARMC6	Armadillo repeat-containing protein 6	29.98	30.07	55.9	16	1.141
sp|Q9H444|CHM4B_HUMAN	CHMP4B	Charged multivesicular body protein 4b	9.78	10.15	50.5	12	1.141
sp|Q9UHR6|ZNHI2_HUMAN	ZNHIT2	Zinc finger HIT domain-containing protein 2	9.89	10	28.5	6	1.141
sp|Q12789|TF3C1_HUMAN	GTF3C1	General transcription factor 3C polypeptide 1	88.46	89.92	42.2	47	1.141
sp|P00742|FA10_HUMAN	F10	Coagulation factor X	5.77	5.83	13.5	3	1.141
sp|P46100|ATRX_HUMAN	ATRX	Transcriptional regulator ATRX	10.19	13.55	15.6	9	1.141
sp|Q69YQ0|CYTSA_HUMAN	SPECC1L	Cytospin-A	25.66	32.78	37.4	17	1.142
sp|P55789|ALR_HUMAN	GFER	FAD-linked sulfhydryl oxidase ALR	6.03	6.03	35.6	3	1.142
sp|Q99963|SH3G3_HUMAN	SH3GL3	Endophilin-A3	2.22	2.49	32	2	1.142
sp|Q92785|REQU_HUMAN	DPF2	Zinc finger protein ubi-d4	15.35	15.47	43.2	10	1.142
sp|O75394|RM33_HUMAN	MRPL33	39S ribosomal protein L33, mitochondrial	2.33	2.35	30.8	2	1.142
sp|Q4VC44|FWCH1_HUMAN	FLYWCH1	FLYWCH-type zinc finger-containing protein 1	4.26	4.29	25.8	2	1.142
sp|Q8IYB3|SRRM1_HUMAN	SRRM1	Serine/arginine repetitive matrix protein 1	9.17	11.12	25.1	8	1.142
sp|Q8WUR7|CO040_HUMAN	C15orf40	UPF0235 protein C15orf40	2.26	2.67	47.7	3	1.142
sp|Q9Y4P1|ATG4B_HUMAN	ATG4B	Cysteine protease ATG4B	19.84	19.99	63.9	12	1.142
sp|Q9BXV9|CN142_HUMAN	C14orf142	Uncharacterized protein C14orf142	4.42	4.46	57	3	1.143
sp|Q4VC05|BCL7A_HUMAN	BCL7A	B-cell CLL/lymphoma 7 protein family member A	4.02	6.03	12.9	3	1.143
sp|P29558|RBMS1_HUMAN	RBMS1	RNA-binding motif, single-stranded-interacting protein 1	8.79	9.02	33.7	6	1.143
sp|Q9NW82|WDR70_HUMAN	WDR70	WD repeat-containing protein 70	20.11	20.61	46	13	1.143
sp|P26358|DNMT1_HUMAN	DNMT1	DNA (cytosine-5)-methyltransferase 1	88.51	89.69	51.4	46	1.143
sp|Q9H3P7|GCP60_HUMAN	ACBD3	Golgi resident protein GCP60	24.45	25.91	62.3	19	1.144
sp|P06132|DCUP_HUMAN	UROD	Uroporphyrinogen decarboxylase	25.7	25.75	67.6	14	1.144
sp|Q9Y6D6|BIG1_HUMAN	ARFGEF1	Brefeldin A-inhibited guanine nucleotide-exchange protein 1	23.45	48	29.1	26	1.144
sp|P38435|VKGC_HUMAN	GGCX	Vitamin K-dependent gamma-carboxylase	19.91	20.3	26.5	12	1.144
sp|O95336|6PGL_HUMAN	PGLS	6-phosphogluconolactonase	18.43	18.93	70.2	19	1.144
sp|O60828|PQBP1_HUMAN	PQBP1	Polyglutamine-binding protein 1	11.76	11.87	61.5	12	1.145
sp|Q13619|CUL4A_HUMAN	CUL4A	Cullin-4A	13.72	37	44.8	24	1.145
sp|Q9NWU1|OXSM_HUMAN	OXSM	3-oxoacyl-[acyl-carrier-protein] synthase, mitochondrial	14.19	14.22	36.2	7	1.145
sp|O00178|GTPB1_HUMAN	GTPBP1	GTP-binding protein 1	29.26	29.58	41.7	22	1.145
sp|Q9UBS4|DJB11_HUMAN	DNAJB11	DnaJ homolog subfamily B member 11	21.68	22.25	52	17	1.145
sp|Q08AD1|CAMP2_HUMAN	CAMSAP2	Calmodulin-regulated spectrin-associated protein 2	3	5.87	14.5	9	1.145
sp|Q96BT7|ALKB8_HUMAN	ALKBH8	Alkylated DNA repair protein alkB homolog 8	2.56	2.61	20	2	1.145
sp|Q9ULL5|PRR12_HUMAN	PRR12	Proline-rich protein 12	4	4.37	11.6	3	1.145
sp|Q9P2D3|HTR5B_HUMAN	HEATR5B	HEAT repeat-containing protein 5B	25.96	27.53	24.6	16	1.145

sp|Q9GZM8|NDEL1_HUMAN	NDEL1	Nuclear distribution protein nudE-like 1	2.03	4.07	35.4	3	1.146
sp|Q6PJF5|RHDF2_HUMAN	RHBDF2	Inactive rhomboid protein 2	6.02	6.05	9.8	4	1.146
sp|P29353|SHC1_HUMAN	SHC1	SHC-transforming protein 1	19.37	19.53	35.7	12	1.146
sp|Q8WUY8|NAT14_HUMAN	NAT14	N-acetyltransferase 14	5.72	7.88	39.8	5	1.146
sp|Q96I24|FUBP3_HUMAN	FUBP3	Far upstream element-binding protein 3	50.74	51.07	81.3	35	1.147
sp|Q6PCB0|VWA1_HUMAN	VWA1	von Willebrand factor A domain-containing protein 1	4	4	12.4	2	1.147
sp|P37287|PIGA_HUMAN	PIGA	Phosphatidylinositol N-acetylglucosaminyltransferase subunit A	2.88	2.96	22.9	2	1.147
sp|Q15366|PCBP2_HUMAN	PCBP2	Poly(rC)-binding protein 2	16.09	30.64	86	37	1.147
sp|O15527|OGG1_HUMAN	OGG1	N-glycosylase/DNA lyase	4.54	4.58	13.6	3	1.147
sp|Q9NQC3|RTN4_HUMAN	RTN4	Reticulon-4	21.48	22.04	29.4	28	1.147
sp|P13284|GILT_HUMAN	IFI30	Gamma-interferon-inducible lysosomal thiol reductase	2.08	2.11	38.8	2	1.147
sp|Q07973|CP24A_HUMAN	CYP24A1	1,25-dihydroxyvitamin D(3) 24-hydroxylase, mitochondrial	2.77	2.95	21	2	1.147
sp|Q99653|CHP1_HUMAN	CHP1	Calcineurin B homologous protein 1	10.21	11.36	71.8	9	1.148
sp|Q9NVH1|DJC11_HUMAN	DNAJC11	DnaJ homolog subfamily C member 11	33.97	34.04	59.8	19	1.148
sp|Q8IYU8|MICU2_HUMAN	MICU2	Calcium uptake protein 2, mitochondrial	14.65	14.83	47.9	11	1.148
sp|Q9H6E5|STPAP_HUMAN	TUT1	Speckle targeted PIP5K1A-regulated poly(A) polymerase	8.07	8.09	18.4	5	1.149
sp|Q9BU89|DOHH_HUMAN	DOHH	Deoxyhypusine hydroxylase	16.08	16.15	61.6	9	1.149
sp|Q9UPR3|SMG5_HUMAN	SMG5	Protein SMG5	3.96	4.12	12.1	3	1.149
sp|O75419|CDC45_HUMAN	CDC45	Cell division control protein 45 homolog	11.22	12.29	26.2	10	1.149
sp|Q9Y320|TMX2_HUMAN	TMX2	Thioredoxin-related transmembrane protein 2	14.23	14.29	38.2	10	1.149
sp|Q14258|TRI25_HUMAN	TRIM25	E3 ubiquitin/ISG15 ligase TRIM25	44.15	44.4	69.4	31	1.150
sp|Q8IVH2|FOXP4_HUMAN	FOXP4	Forkhead box protein P4	2.42	2.46	16.5	2	1.150
sp|Q05086|UBE3A_HUMAN	UBE3A	Ubiquitin-protein ligase E3A	26	27.87	36.7	16	1.150
sp|Q08357|S20A2_HUMAN	SLC20A2	Sodium-dependent phosphate transporter 2	2.02	2.84	16.4	2	1.150
sp|Q7Z3D6|CN159_HUMAN	C14orf159	UPF0317 protein C14orf159, mitochondrial	5.2	6.56	24.7	5	1.150
sp|Q8WV22|NSE1_HUMAN	NSMCE1	Non-structural maintenance of chromosomes element 1 homolog	9.69	9.8	40.2	6	1.150
sp|Q5T3U5|MRP7_HUMAN	ABCC10	Multidrug resistance-associated protein 7	3.7	4.02	12.3	4	1.150
sp|Q8WWC4|CB047_HUMAN	C2orf47	Uncharacterized protein C2orf47, mitochondrial	4.69	4.75	36.8	3	1.150
sp|Q6QNY0|BL1S3_HUMAN	BLOC1S3	Biogenesis of lysosome-related organelles complex 1 subunit 3	4.64	4.7	25.7	3	1.150
sp|Q86XI2|CNDG2_HUMAN	NCAPG2	Condensin-2 complex subunit G2	28.02	30.77	33.9	17	1.150
sp|Q8TD30|ALAT2_HUMAN	GPT2	Alanine aminotransferase 2	44.38	44.48	70.4	33	1.151
sp|Q9UGR2|Z3H7B_HUMAN	ZC3H7B	Zinc finger CCCH domain-containing protein 7B	11.95	12.49	27.5	7	1.151
sp|Q9UJX6|ANC2_HUMAN	ANAPC2	Anaphase-promoting complex subunit 2	10.02	10.53	27.5	9	1.151
sp|Q9Y4K4|M4K5_HUMAN	MAP4K5	Mitogen-activated protein kinase kinase kinase kinase 5	16.03	16.42	28	11	1.151
sp|Q03111|ENL_HUMAN	MLLT1	Protein ENL	4.92	5.08	17.2	4	1.152
sp|Q96AY4|TTC28_HUMAN	TTC28	Tetratricopeptide repeat protein 28	7.68	8.06	15.1	5	1.152
sp|Q15052|ARHG6_HUMAN	ARHGEF6	Rho guanine nucleotide exchange factor 6	2	2.74	17.4	4	1.152
sp|Q9Y4J8|DTNA_HUMAN	DTNA	Dystrobrevin alpha	11.06	11.35	20.3	8	1.152
sp|Q9Y5R8|TPPC1_HUMAN	TRAPPC1	Trafficking protein particle complex subunit 1	4.21	4.45	53.8	3	1.152
sp|Q13137|CACO2_HUMAN	CALCOCO2	Calcium-binding and coiled-coil domain-containing protein 2	9.08	11.86	37.9	7	1.152
sp|Q96R06|SPAG5_HUMAN	SPAG5	Sperm-associated antigen 5	13.41	15.76	27.8	8	1.152
sp|P53701|CCHL_HUMAN	HCCS	Cytochrome c-type heme lyase	21.77	21.91	68.7	16	1.153
sp|Q3ZCW2|LEGL_HUMAN	LGALSL	Galectin-related protein	11.09	11.23	40.7	6	1.153
sp|O43815|STRN_HUMAN	STRN	Striatin	37.09	37.15	48.9	20	1.153
sp|Q9UIW2|PLXA1_HUMAN	PLXNA1	Plexin-A1	22.74	23.25	24.7	16	1.153
sp|O75529|TAF5L_HUMAN	TAF5L	TAF5-like RNA polymerase II p300/CBP-associated factor-associated factor 65 kDa subunit 5L	2.26	2.32	21.1	2	1.153
sp|Q8TAF3|WDR48_HUMAN	WDR48	WD repeat-containing protein 48	26.47	26.81	40.2	18	1.153
sp|O75356|ENTP5_HUMAN	ENTPD5	Ectonucleoside triphosphate diphosphohydrolase 5	13.28	13.34	33.2	8	1.153
sp|Q9UP83|COG5_HUMAN	COG5	Conserved oligomeric Golgi complex subunit 5	13.16	15.39	35.6	11	1.154

sp|Q15596|NCOA2_HUMAN	NCOA2	Nuclear receptor coactivator 2	1.82	4.42	8.2	5	1.154
sp|Q12792|TWF1_HUMAN	TWF1	Twinfilin-1	18.2	24.3	71.1	19	1.155
sp|P49902|5NTC_HUMAN	NT5C2	Cytosolic purine 5'-nucleotidase	17.62	17.72	39	9	1.155
sp|Q9Y2D2|S35A3_HUMAN	SLC35A3	UDP-N-acetylglucosamine transporter	1.54	1.63	18.2	2	1.155
sp|Q13188|STK3_HUMAN	STK3	Serine/threonine-protein kinase 3	6.04	19.47	42	11	1.155
sp|Q9Y2Q3|GSTK1_HUMAN	GSTK1	Glutathione S-transferase kappa 1	9.84	9.98	46.5	8	1.155
sp|P12273|PIP_HUMAN	PIP	Prolactin-inducible protein	2	5.88	30.1	3	1.155
sp|Q9NQ89|CL004_HUMAN	C12orf4	Protein C12orf4	2	2.02	9.1	2	1.155
sp|Q9H6S3|ES8L2_HUMAN	EPS8L2	Epidermal growth factor receptor kinase substrate 8-like protein 2	31.19	33.42	50.4	19	1.155
sp|P49789|FHIT_HUMAN	FHIT	Bis(5'-adenosyl)-triphosphatase	4	4	39.5	2	1.155
sp|Q9BXP5|SRRT_HUMAN	SRRT	Serrate RNA effector molecule homolog	58.8	61.5	54.6	38	1.156
sp|Q96SN8|CK5P2_HUMAN	CDK5RAP2	CDK5 regulatory subunit-associated protein 2	2.84	7.49	21.1	5	1.156
sp|P55795|HNRH2_HUMAN	HNRNPH2	Heterogeneous nuclear ribonucleoprotein H2	16.25	43.03	63	62	1.156
sp|P19823|ITIH2_HUMAN	ITIH2	Inter-alpha-trypsin inhibitor heavy chain H2	35.21	38.76	46	25	1.156
sp|Q86UL3|GPAT4_HUMAN	AGPAT6	Glycerol-3-phosphate acyltransferase 4	10.67	11.06	29	6	1.157
sp|Q5VT25|MRCKA_HUMAN	CDC42BPA	Serine/threonine-protein kinase MRCK alpha	19.19	30.65	30	18	1.157
sp|P61225|RAP2B_HUMAN	RAP2B	Ras-related protein Rap-2b	17.17	20.85	78.7	14	1.157
sp|Q7Z3T8|ZFY16_HUMAN	ZFYVE16	Zinc finger FYVE domain-containing protein 16	20.02	20.37	26.7	13	1.157
sp|O60716|CTND1_HUMAN	CTNND1	Catenin delta-1	58.03	58.9	56.1	44	1.157
sp|Q9NQT5|EXOS3_HUMAN	EXOSC3	Exosome complex component RRP40	17.81	18.33	77.5	16	1.157
sp|Q14789|GOGB1_HUMAN	GOLGB1	Golgin subfamily B member 1	139.66	144.62	49.7	81	1.158
sp|Q9Y4C1|KDM3A_HUMAN	KDM3A	Lysine-specific demethylase 3A	7.05	7.64	19.2	5	1.158
sp|Q8N4C8|MINK1_HUMAN	MINK1	Misshapen-like kinase 1	5.23	19.9	26.7	12	1.159
sp|P62745|RHOB_HUMAN	RHOB	Rho-related GTP-binding protein RhoB	8.46	21.37	75.5	21	1.159
sp|Q9UL33|TPC2L_HUMAN	TRAPPC2L	Trafficking protein particle complex subunit 2-like protein	4.72	4.8	47.1	3	1.159
sp|P61964|WDR5_HUMAN	WDR5	WD repeat-containing protein 5	22	22.13	66.8	13	1.159
sp|Q9H9A6|LRC40_HUMAN	LRRC40	Leucine-rich repeat-containing protein 40	47.86	48.47	73.1	27	1.159
sp|Q9Y2S2|CRYL1_HUMAN	CRYL1	Lambda-crystallin homolog	15.36	15.67	59.6	8	1.159
sp|P05067|A4_HUMAN	APP	Amyloid beta A4 protein	3.74	5.01	25.1	5	1.160
sp|Q9NPF5|DMAP1_HUMAN	DMAP1	DNA methyltransferase 1-associated protein 1	14.22	14.51	43.5	7	1.160
sp|P14324|FPPS_HUMAN	FDPS	Farnesyl pyrophosphate synthase	26.23	26.24	38	19	1.160
sp|P50613|CDK7_HUMAN	CDK7	Cyclin-dependent kinase 7	4.84	5.09	39.6	7	1.160
sp|Q9H6T3|RPAP3_HUMAN	RPAP3	RNA polymerase II-associated protein 3	31.54	32.83	60.6	22	1.160
sp|Q9Y3E5|PTH2_HUMAN	PTRH2	Peptidyl-tRNA hydrolase 2, mitochondrial	15.53	15.59	67	13	1.161
sp|P20618|PSB1_HUMAN	PSMB1	Proteasome subunit beta type-1	26.89	26.96	74.7	25	1.161
sp|O76054|S14L2_HUMAN	SEC14L2	SEC14-like protein 2	3.72	4.06	28.5	6	1.161
sp|Q86XL3|ANKL2_HUMAN	ANKLE2	Ankyrin repeat and LEM domain-containing protein 2	15.44	17.31	29.1	11	1.161
sp|P30626|SORCN_HUMAN	SRI	Sorcin	13.62	13.68	79.3	11	1.161
sp|Q9ULK4|MED23_HUMAN	MED23	Mediator of RNA polymerase II transcription subunit 23	15.22	15.56	17.3	8	1.162
sp|Q9NVS2|RT18A_HUMAN	MRPS18A	28S ribosomal protein S18a, mitochondrial	11.01	11.15	47.5	7	1.162
sp|Q8N1G4|LRC47_HUMAN	LRRC47	Leucine-rich repeat-containing protein 47	38.37	38.46	63.6	25	1.163
sp|O15027|SC16A_HUMAN	SEC16A	Protein transport protein Sec16A	52.29	52.4	24.1	30	1.163
sp|Q9UBB5|MBD2_HUMAN	MBD2	Methyl-CpG-binding domain protein 2	2.31	3.02	28.5	3	1.163
sp|P11441|UBL4A_HUMAN	UBL4A	Ubiquitin-like protein 4A	8.7	8.96	54.8	8	1.163
sp|Q8NFI3|ENASE_HUMAN	ENGASE	Cytosolic endo-beta-N-acetylglucosaminidase	6.22	6.27	13.9	4	1.163
sp|Q9BY77|PDIP3_HUMAN	POLDIP3	Polymerase delta-interacting protein 3	30.48	30.53	64.4	23	1.163
sp|Q8NCE0|SEN2_HUMAN	TSEN2	tRNA-splicing endonuclease subunit Sen2	4.15	4.2	22.4	3	1.163
sp|O95139|NDUB6_HUMAN	NDUFB6	NADH dehydrogenase [ubiquinone] 1 beta subcomplex subunit 6	5.37	5.49	67.2	4	1.163
sp|Q969H6|POP5_HUMAN	POP5	Ribonuclease P/MRP protein subunit POP5	2.62	2.66	27.6	3	1.163

sp|Q86WX3|AROS_HUMAN	RPS19BP1	Active regulator of SIRT1	6.2	7.1	62.5	5	1.164
sp|O15164|TIF1A_HUMAN	TRIM24	Transcription intermediary factor 1-alpha	26.6	31.12	37.2	20	1.164
sp|Q6PK04|CC137_HUMAN	CCDC137	Coiled-coil domain-containing protein 137	13.22	13.45	51.9	9	1.164
sp|P56962|STX17_HUMAN	STX17	Syntaxin-17	4.04	4.04	25.5	3	1.164
sp|P63146|UBE2B_HUMAN	UBE2B	Ubiquitin-conjugating enzyme E2 B	4	6	32.2	3	1.165
sp|O76062|ERG24_HUMAN	TM7SF2	Delta(14)-sterol reductase	2.56	2.78	17.9	3	1.165
sp|Q9Y608|LRRF2_HUMAN	LRRFIP2	Leucine-rich repeat flightless-interacting protein 2	12.27	15.72	43.7	16	1.165
sp|Q8N961|ABTB2_HUMAN	ABTB2	Ankyrin repeat and BTB/POZ domain-containing protein 2	4.04	4.08	13.5	2	1.165
sp|Q92734|TFG_HUMAN	TFG	Protein TFG	23.92	24.95	49	28	1.166
sp|Q9UHD8|SEPT9_HUMAN	SEPT9	Septin-9	61.35	62.28	78.2	48	1.166
sp|Q86V85|GP180_HUMAN	GPR180	Integral membrane protein GPR180	4	4.01	16.4	2	1.166
sp|Q5JPH6|SYEM_HUMAN	EARS2	Probable glutamate--tRNA ligase, mitochondrial	22.38	28.3	57.7	20	1.166
sp|O60271|JIP4_HUMAN	SPAG9	C-Jun-amino-terminal kinase-interacting protein 4	65.54	66.54	47.3	36	1.166
sp|Q07954|LRP1_HUMAN	LRP1	Prolow-density lipoprotein receptor-related protein 1	44.99	45.59	16.6	25	1.166
sp|P12081|SYHC_HUMAN	HARS	Histidine--tRNA ligase, cytoplasmic	53.1	56.08	74.5	33	1.166
sp|Q96AT1|K1143_HUMAN	KIAA1143	Uncharacterized protein KIAA1143	5.47	5.54	33.8	3	1.166
sp|Q86XX4|FRAS1_HUMAN	FRAS1	Extracellular matrix protein FRAS1	4.18	4.87	7.7	6	1.166
sp|Q92685|ALG3_HUMAN	ALG3	Dol-P-Man:Man(5)GlcNAc(2)-PP-Dol alpha-1,3-mannosyltransferase	2.13	2.15	11.9	3	1.167
sp|Q13111|CAF1A_HUMAN	CHAF1A	Chromatin assembly factor 1 subunit A	12.39	13.41	32.5	11	1.167
sp|Q9UQ90|SPG7_HUMAN	SPG7	Paraplegin	27.89	30.69	53.5	16	1.167
sp|Q9UJY5|GGA1_HUMAN	GGA1	ADP-ribosylation factor-binding protein GGA1	13.08	16.19	27.1	9	1.167
sp|O94808|GFPT2_HUMAN	GFPT2	Glutamine--fructose-6-phosphate aminotransferase [isomerizing] 2	6.55	16.38	34.5	10	1.167
sp|P54277|PMS1_HUMAN	PMS1	PMS1 protein homolog 1	7.92	8.08	18.6	5	1.167
sp|P53041|PPP5_HUMAN	PPP5C	Serine/threonine-protein phosphatase 5	30.48	30.72	57.5	24	1.168
sp|O75340|PDCD6_HUMAN	PDCD6	Programmed cell death protein 6	14.01	14.09	57.6	8	1.168
sp|Q9H0E3|SP130_HUMAN	SAP130	Histone deacetylase complex subunit SAP130	6.59	6.81	11.6	5	1.168
sp|Q96IG2|FXL20_HUMAN	FBXL20	F-box/LRR-repeat protein 20	3.47	3.74	28.4	4	1.168
sp|Q96EZ8|MCRS1_HUMAN	MCRS1	Microspherule protein 1	2.64	2.73	25.1	2	1.168
sp|Q9NNW5|WDR6_HUMAN	WDR6	WD repeat-containing protein 6	27.28	27.44	32.5	17	1.168
sp|O95544|NADK_HUMAN	NADK	NAD kinase	2.86	3.07	11.2	3	1.168
sp|Q09028|RBBP4_HUMAN	RBBP4	Histone-binding protein RBBP4	13.36	26.47	60.2	32	1.168
sp|Q8NBT2|SPC24_HUMAN	SPC24	Kinetochore protein Spc24	10.06	12.08	44.7	9	1.168
sp|Q8N3C0|ASCC3_HUMAN	ASCC3	Activating signal cointegrator 1 complex subunit 3	61.76	64.88	39.4	34	1.168
sp|Q9Y6H1|CHCH2_HUMAN	CHCHD2	Coiled-coil-helix-coiled-coil-helix domain-containing protein 2	8	8	42.4	8	1.169
sp|P63220|RS21_HUMAN	RPS21	40S ribosomal protein S21	11.33	12.43	85.5	24	1.169
sp|P61224|RAP1B_HUMAN	RAP1B	Ras-related protein Rap-1b	27.98	28.67	85.9	40	1.169
sp|Q92576|PHF3_HUMAN	PHF3	PHD finger protein 3	43.69	45	34.7	23	1.170
sp|Q9P031|TAP26_HUMAN	CCDC59	Thyroid transcription factor 1-associated protein 26	4.09	4.21	23.7	3	1.170
sp|Q8TEQ8|PIGO_HUMAN	PIGO	GPI ethanolamine phosphate transferase 3	2.29	2.43	18.1	7	1.170
sp|Q96DZ1|ERLEC_HUMAN	ERLEC1	Endoplasmic reticulum lectin 1	23.8	23.88	50.3	16	1.170
sp|Q5VW36|FOCAD_HUMAN	FOCAD	Focadhesin	10.02	13.94	18.7	8	1.170
sp|Q15785|TOM34_HUMAN	TOMM34	Mitochondrial import receptor subunit TOM34	34.43	34.49	67	23	1.170
sp|P00568|KAD1_HUMAN	AK1	Adenylate kinase isoenzyme 1	15.47	16.61	62.4	14	1.170
sp|Q16527|CSRP2_HUMAN	CSRP2	Cysteine and glycine-rich protein 2	10.57	10.81	66.8	7	1.170
sp|Q9H4K7|MTG2_HUMAN	MTG2	Mitochondrial ribosome-associated GTPase 2	2.02	2.1	16.5	2	1.171
sp|P78364|PHC1_HUMAN	PHC1	Polyhomeotic-like protein 1	2.4	2.53	11.3	2	1.171
sp|Q99798|ACON_HUMAN	ACO2	Aconitate hydratase, mitochondrial	60.85	63.28	65.6	57	1.171
sp|Q13637|RAB32_HUMAN	RAB32	Ras-related protein Rab-32	18.5	18.55	64.9	10	1.171
sp|Q99720|SGMR1_HUMAN	SIGMAR1	Sigma non-opioid intracellular receptor 1	8.06	9.06	36.8	11	1.172

sp|Q99611|SPS2_HUMAN	SEPHS2	Selenide, water dikinase 2	8.17	9.89	37.3	6	1.172
sp|Q5UIP0|RIF1_HUMAN	RIF1	Telomere-associated protein RIF1	104.55	105.88	44.5	61	1.172
sp|Q5T9L3|WLS_HUMAN	WLS	Protein wntless homolog	6.38	6.61	21.3	4	1.172
sp|P46977|STT3A_HUMAN	STT3A	Dolichyl-diphosphooligosaccharide--protein glycosyltransferase subunit STT3A	44.58	44.6	37.6	30	1.172
sp|P23378|GCSP_HUMAN	GLDC	Glycine dehydrogenase (decarboxylating), mitochondrial	63.52	63.71	52.8	40	1.173
sp|Q9Y5B6|PAXB1_HUMAN	PAXBP1	PAX3- and PAX7-binding protein 1	20.27	20.97	32.9	14	1.173
sp|P40123|CAP2_HUMAN	CAP2	Adenylyl cyclase-associated protein 2	16.87	20.67	48.2	13	1.173
sp|Q9BT25|HAUS8_HUMAN	HAUS8	HAUS augmin-like complex subunit 8	14.09	14.2	48.1	7	1.173
sp|Q10570|CPSF1_HUMAN	CPSF1	Cleavage and polyadenylation specificity factor subunit 1	80.85	82.33	50.1	54	1.174
sp|Q14677|EPN4_HUMAN	CLINT1	Clathrin interactor 1	34.77	34.98	37.8	22	1.174
sp|Q7LGA3|HS2ST_HUMAN	HS2ST1	Heparan sulfate 2-O-sulfotransferase 1	17	17.33	43.8	9	1.174
sp|Q9Y2U5|M3K2_HUMAN	MAP3K2	Mitogen-activated protein kinase kinase kinase 2	3.86	4.06	23.4	6	1.174
sp|O14936|CSKP_HUMAN	CASK	Peripheral plasma membrane protein CASK	39.59	39.83	40.8	22	1.174
sp|P26572|MGAT1_HUMAN	MGAT1	Alpha-1,3-mannosyl-glycoprotein 2-beta-N-acetylglucosaminyltransferase	12.28	12.37	29.9	7	1.174
sp|Q93050|VPP1_HUMAN	ATP6V0A1	V-type proton ATPase 116 kDa subunit a isoform 1	29.43	29.69	39.6	18	1.174
sp|Q9HBM6|TAF9B_HUMAN	TAF9B	Transcription initiation factor TFIID subunit 9B	9.07	9.37	36.7	7	1.174
sp|Q12955|ANK3_HUMAN	ANK3	Ankyrin-3	19.74	21.28	16.1	14	1.175
sp|Q5T0D9|TPRGL_HUMAN	TPRG1L	Tumor protein p63-regulated gene 1-like protein	2.09	2.25	39.3	2	1.175
sp|P04114|APOB_HUMAN	APOB	Apolipoprotein B-100	234.53	236.13	48.9	148	1.175
sp|Q9Y2H2|SAC2_HUMAN	INPP5F	Phosphatidylinositide phosphatase SAC2	2.08	2.28	15.6	3	1.175
sp|Q9BT22|ALG1_HUMAN	ALG1	Chitobiosyldiphosphodolichol beta-mannosyltransferase	10.08	10.09	30	5	1.175
sp|Q04864|REL_HUMAN	REL	Proto-oncogene c-Rel	7.29	7.57	20.4	7	1.175
sp|Q2VPK5|CTU2_HUMAN	CTU2	Cytoplasmic tRNA 2-thiolation protein 2	8.3	8.48	28.4	6	1.175
sp|Q9NVD7|PARVA_HUMAN	PARVA	Alpha-parvin	8.01	8.15	34.4	7	1.176
sp|Q9NRF9|DPOE3_HUMAN	POLE3	DNA polymerase epsilon subunit 3	11.62	11.74	65.3	11	1.176
sp|Q14693|LPIN1_HUMAN	LPIN1	Phosphatidate phosphatase LPIN1	3.61	4.07	11.4	3	1.176
sp|Q5VU97|CAHD1_HUMAN	CACHD1	VWFA and cache domain-containing protein 1	2.03	4.06	13.4	3	1.176
sp|Q8TCG2|P4K2B_HUMAN	PI4K2B	Phosphatidylinositol 4-kinase type 2-beta	2.07	2.47	20.2	2	1.176
sp|P05161|ISG15_HUMAN	ISG15	Ubiquitin-like protein ISG15	6.3	6.49	41.2	5	1.177
sp|Q9BY67|CADM1_HUMAN	CADM1	Cell adhesion molecule 1	16.91	17	49.6	11	1.177
sp|O00273|DFFA_HUMAN	DFFA	DNA fragmentation factor subunit alpha	24.93	26.54	69.5	18	1.177
sp|Q86YM7|HOME1_HUMAN	HOMER1	Homer protein homolog 1	3.97	4.16	27.1	4	1.177
sp|Q9UHI6|DDX20_HUMAN	DDX20	Probable ATP-dependent RNA helicase DDX20	36.19	36.74	55	20	1.177
sp|P08648|ITA5_HUMAN	ITGA5	Integrin alpha-5	11.01	11.19	16	8	1.177
sp|Q13098|CSN1_HUMAN	GPS1	COP9 signalosome complex subunit 1	28.24	30.78	55	18	1.177
sp|P62277|RS13_HUMAN	RPS13	40S ribosomal protein S13	21.42	23.29	71.5	23	1.178
sp|Q92733|PRCC_HUMAN	PRCC	Proline-rich protein PRCC	14.03	14.3	42.2	10	1.178
sp|Q6ZSZ5|ARHGI_HUMAN	ARHGEF18	Rho guanine nucleotide exchange factor 18	6.24	7.82	25.2	6	1.178
sp|P02786|TFR1_HUMAN	TFRC	Transferrin receptor protein 1	58.82	59.12	56.5	44	1.178
sp|P20810|ICAL_HUMAN	CAST	Calpastatin	37.66	38.59	57.1	24	1.178
sp|Q9BTD8|RBM42_HUMAN	RBM42	RNA-binding protein 42	4.82	4.94	36.3	8	1.179
sp|O75439|MPPB_HUMAN	PMPCB	Mitochondrial-processing peptidase subunit beta	37.78	37.84	63.2	37	1.179
sp|O15173|PGRC2_HUMAN	PGRMC2	Membrane-associated progesterone receptor component 2	17.8	22.01	57	21	1.179
sp|Q13586|STIM1_HUMAN	STIM1	Stromal interaction molecule 1	19.18	21.96	32.9	13	1.179
sp|Q8IWY9|CDAN1_HUMAN	CDAN1	Codanin-1	2.99	4.24	13.3	6	1.179
sp|Q8NCC3|PAG15_HUMAN	PLA2G15	Group XV phospholipase A2	4.82	4.9	19.7	4	1.179
sp|P09327|VILI_HUMAN	VIL1	Villin-1	78.23	78.89	68.8	65	1.179
sp|P35237|SPB6_HUMAN	SERPINB6	Serpin B6	31.24	31.85	73.4	18	1.179
sp|Q8WTS6|SETD7_HUMAN	SETD7	Histone-lysine N-methyltransferase SETD7	4.31	4.4	14.8	3	1.179

sp|Q8N183|MIMIT_HUMAN	NDUFAF2	Mimitin, mitochondrial	21.27	21.56	80.5	12	1.179
sp|Q96HW7|INT4_HUMAN	INTS4	Integrator complex subunit 4	17.4	17.61	29.7	9	1.180
sp|Q9H7N4|SFR19_HUMAN	SCAF1	Splicing factor, arginine/serine-rich 19	19.12	21.06	25.5	12	1.180
sp|Q9BVS5|TR61B_HUMAN	TRMT61B	tRNA (adenine(58)-N(1))-methyltransferase, mitochondrial	5.59	5.78	18.2	4	1.180
sp|P25325|THTM_HUMAN	MPST	3-mercaptopyruvate sulfurtransferase	20.42	20.59	67	12	1.180
sp|Q9UIS9|MBD1_HUMAN	MBD1	Methyl-CpG-binding domain protein 1	4.08	4.1	21.3	4	1.180
sp|Q9P0P0|RN181_HUMAN	RNF181	E3 ubiquitin-protein ligase RNF181	4	4.36	23.5	3	1.180
sp|Q5JRA6|MIA3_HUMAN	MIA3	Melanoma inhibitory activity protein 3	62.57	65.16	41.3	45	1.181
sp|O95562|SFT2B_HUMAN	SFT2D2	Vesicle transport protein SFT2B	3.03	3.1	20	2	1.181
sp|O75489|NDUS3_HUMAN	NDUFS3	NADH dehydrogenase [ubiquinone] iron-sulfur protein 3, mitochondrial	24.01	24.15	64.8	20	1.181
sp|Q8TF68|ZN384_HUMAN	ZNF384	Zinc finger protein 384	2.89	5.09	12.8	3	1.181
sp|Q9Y5X2|SNX8_HUMAN	SNX8	Sorting nexin-8	6	6.02	19.4	3	1.181
sp|Q96EK6|GNA1_HUMAN	GNPNAT1	Glucosamine 6-phosphate N-acetyltransferase	9.08	9.28	64.7	10	1.181
sp|Q9BWT3|PAPOG_HUMAN	PAPOLG	Poly(A) polymerase gamma	2.61	7.05	20	5	1.181
sp|Q99614|TTC1_HUMAN	TTC1	Tetratricopeptide repeat protein 1	17.89	18.13	49.7	12	1.182
sp|Q9Y680|FKBP7_HUMAN	FKBP7	Peptidyl-prolyl cis-trans isomerase FKBP7	6.2	6.22	39.8	3	1.182
sp|Q9Y3D6|FIS1_HUMAN	FIS1	Mitochondrial fission 1 protein	10	10.01	52.6	8	1.182
sp|Q9NQG5|RPR1B_HUMAN	RPRD1B	Regulation of nuclear pre-mRNA domain-containing protein 1B	26.94	27.18	71.2	21	1.182
sp|Q3YEC7|RABL6_HUMAN	RABL6	Rab-like protein 6	21.31	21.49	32.5	12	1.182
sp|P31153|METK2_HUMAN	MAT2A	S-adenosylmethionine synthase isoform type-2	28.67	29.49	73.7	17	1.182
sp|Q14691|PSF1_HUMAN	GINS1	DNA replication complex GINS protein PSF1	10.29	10.46	62.8	7	1.182
sp|Q641Q2|FA21A_HUMAN	FAM21A	WASH complex subunit FAM21A	36.17	36.43	38.6	20	1.182
sp|Q96C36|P5CR2_HUMAN	PYCR2	Pyrroline-5-carboxylate reductase 2	37.47	37.52	72.2	41	1.182
sp|Q5H9F3|BCORL_HUMAN	BCORL1	BCL-6 corepressor-like protein 1	4.02	4.09	7.8	3	1.183
sp|Q13045|FLII_HUMAN	FLII	Protein flightless-1 homolog	73.11	73.16	50	44	1.183
sp|Q5VU43|MYOME_HUMAN	PDE4DIP	Myomegalin	4.05	4.78	20.8	8	1.183
sp|Q04656|ATP7A_HUMAN	ATP7A	Copper-transporting ATPase 1	11.33	11.85	15.1	8	1.183
sp|O95359|TACC2_HUMAN	TACC2	Transforming acidic coiled-coil-containing protein 2	2.06	4.73	9.2	3	1.183
sp|O15067|PUR4_HUMAN	PFAS	Phosphoribosylformylglycinamidine synthase	69.18	69.35	58.5	52	1.183
sp|Q96PZ0|PUS7_HUMAN	PUS7	Pseudouridylate synthase 7 homolog	48.31	48.52	68.7	27	1.184
sp|P07942|LAMB1_HUMAN	LAMB1	Laminin subunit beta-1	68.31	72.22	38.5	39	1.184
sp|Q9NXR7|BRE_HUMAN	BRE	BRCA1-A complex subunit BRE	10.61	10.87	38.1	9	1.185
sp|Q9NY35|CLDN1_HUMAN	CLDND1	Claudin domain-containing protein 1	2.25	4.33	17	2	1.185
sp|O43150|ASAP2_HUMAN	ASAP2	Arf-GAP with SH3 domain, ANK repeat and PH domain-containing protein 2	6.01	6.23	14	3	1.185
sp|Q03252|LMNB2_HUMAN	LMNB2	Lamin-B2	61.87	69.88	70.3	51	1.185
sp|P00374|DYR_HUMAN	DHFR	Dihydrofolate reductase	23.82	23.9	79.7	17	1.185
sp|Q96LJ7|DHRS1_HUMAN	DHRS1	Dehydrogenase/reductase SDR family member 1	14.44	15.92	48.2	10	1.185
sp|P21953|ODBB_HUMAN	BCKDHB	2-oxoisovalerate dehydrogenase subunit beta, mitochondrial	19.14	19.26	55.9	13	1.185
sp|Q12888|TP53B_HUMAN	TP53BP1	Tumor suppressor p53-binding protein 1	57.3	58.51	34.5	35	1.186
sp|Q13825|AUHM_HUMAN	AUH	Methylglutaconyl-CoA hydratase, mitochondrial	9.69	10.19	40.7	9	1.186
sp|Q9H814|PHAX_HUMAN	PHAX	Phosphorylated adapter RNA export protein	4.07	4.21	31.5	5	1.186
sp|O94804|STK10_HUMAN	STK10	Serine/threonine-protein kinase 10	7	9.62	31.5	9	1.186
sp|P02763|A1AG1_HUMAN	ORM1	Alpha-1-acid glycoprotein 1	5.72	6.01	35.8	4	1.186
sp|P62195|PRS8_HUMAN	PSMC5	26S protease regulatory subunit 8	58.94	59.67	77.6	45	1.186
sp|P61077|UB2D3_HUMAN	UBE2D3	Ubiquitin-conjugating enzyme E2 D3	7.02	9.19	45.6	23	1.187
sp|Q9UJZ1|STML2_HUMAN	STOML2	Stomatin-like protein 2, mitochondrial	37.04	37.68	73.3	48	1.187
sp|P48637|GSHB_HUMAN	GSS	Glutathione synthetase	60.23	63.77	74.9	42	1.187
sp|P13671|CO6_HUMAN	C6	Complement component C6	2.01	2.05	16.5	2	1.187
sp|P07741|APT_HUMAN	APRT	Adenine phosphoribosyltransferase	28.61	31.23	91.1	41	1.188

sp|Q99584|S10AD_HUMAN	S100A13	Protein S100-A13	6.86	8.36	42.9	5	1.188
sp|P78330|SERB_HUMAN	PSPH	Phosphoserine phosphatase	21.89	22.64	76.4	17	1.188
sp|Q13496|MTM1_HUMAN	MTM1	Myotubularin	7.35	7.49	24.4	4	1.188
sp|Q8IWZ3|ANKH1_HUMAN	ANKHD1	Ankyrin repeat and KH domain-containing protein 1	59.09	64.14	27.9	36	1.188
sp|Q9Y6D9|MD1L1_HUMAN	MAD1L1	Mitotic spindle assembly checkpoint protein MAD1	40.3	41.4	61	23	1.188
sp|O95870|ABHGA_HUMAN	ABHD16A	Abhydrolase domain-containing protein 16A	16.95	17.79	36.9	10	1.188
sp|Q6P2C8|MED27_HUMAN	MED27	Mediator of RNA polymerase II transcription subunit 27	6.86	7.09	30.9	4	1.188
sp|Q9P287|BCCIP_HUMAN	BCCIP	BRCA2 and CDKN1A-interacting protein	26.29	26.38	60.2	18	1.188
sp|Q96T76|MMS19_HUMAN	MMS19	MMS19 nucleotide excision repair protein homolog	40.1	40.86	47.5	27	1.189
sp|P38935|SMBP2_HUMAN	IGHMBP2	DNA-binding protein SMUBP-2	5.7	5.89	16.7	5	1.189
sp|P51159|RB27A_HUMAN	RAB27A	Ras-related protein Rab-27A	11.04	13.18	40.7	11	1.189
sp|Q14457|BECN1_HUMAN	BECN1	Beclin-1	3.07	3.33	12	3	1.189
sp|Q99442|SEC62_HUMAN	SEC62	Translocation protein SEC62	9.87	9.98	26.6	6	1.189
sp|O15160|RPAC1_HUMAN	POLR1C	DNA-directed RNA polymerases I and III subunit RPAC1	23	23.3	59.3	15	1.189
sp|Q9H2K8|TAOK3_HUMAN	TAOK3	Serine/threonine-protein kinase TAO3	4.55	7.45	21.8	6	1.189
sp|Q8N4J0|CARME_HUMAN	C9orf41	Carnosine N-methyltransferase	4.81	4.95	24.7	3	1.189
sp|Q9H0G5|NSRP1_HUMAN	NSRP1	Nuclear speckle splicing regulatory protein 1	1.95	2.32	16.3	2	1.189
sp|Q6V1X1|DPP8_HUMAN	DPP8	Dipeptidyl peptidase 8	2.5	5.07	15.9	6	1.189
sp|Q99590|SCAFB_HUMAN	SCAF11	Protein SCAF11	17.63	19.55	24.1	12	1.189
sp|Q9NZ52|GGA3_HUMAN	GGA3	ADP-ribosylation factor-binding protein GGA3	5.37	8.43	24.5	5	1.189
sp|O15056|SYNJ2_HUMAN	SYNJ2	Synaptojanin-2	7.76	8.54	16.6	7	1.190
sp|P20020|AT2B1_HUMAN	ATP2B1	Plasma membrane calcium-transporting ATPase 1	58.14	59.42	44.7	38	1.190
sp|P26196|DDX6_HUMAN	DDX6	Probable ATP-dependent RNA helicase DDX6	41.15	44.21	80.5	36	1.190
sp|P49642|PRI1_HUMAN	PRIM1	DNA primase small subunit	13.77	14.65	45.7	11	1.190
sp|P05423|RPC4_HUMAN	POLR3D	DNA-directed RNA polymerase III subunit RPC4	9.65	11.96	39.5	9	1.190
sp|O95817|BAG3_HUMAN	BAG3	BAG family molecular chaperone regulator 3	16.91	17.21	50.6	10	1.191
sp|Q6NUK1|SCMC1_HUMAN	SLC25A24	Calcium-binding mitochondrial carrier protein SCaMC-1	38.36	39.39	67.1	26	1.191
sp|P25815|S100P_HUMAN	S100P	Protein S100-P	10.6	13.1	91.6	17	1.191
sp|Q1ED39|KNOP1_HUMAN	KNOP1	Lysine-rich nucleolar protein 1	25.98	26.56	52	14	1.191
sp|Q86Y56|DAAF5_HUMAN	DNAAF5	Dynein assembly factor 5, axonemal	48.7	50.04	64	25	1.191
sp|Q8NFW8|NEUA_HUMAN	CMAS	N-acylneuraminate cytidylyltransferase	26.02	27.62	61.5	22	1.191
sp|O95865|DDAH2_HUMAN	DDAH2	N(G),N(G)-dimethylarginine dimethylaminohydrolase 2	13.28	15.58	64.6	9	1.192
sp|Q9BRP4|PAAF1_HUMAN	PAAF1	Proteasomal ATPase-associated factor 1	18.95	19.01	54.3	9	1.192
sp|P53677|AP3M2_HUMAN	AP3M2	AP-3 complex subunit mu-2	7.85	10.06	30.4	5	1.192
sp|Q9Y6K1|DNM3A_HUMAN	DNMT3A	DNA (cytosine-5)-methyltransferase 3A	20.46	20.87	28.2	13	1.192
sp|O60341|KDM1A_HUMAN	KDM1A	Lysine-specific histone demethylase 1A	59.25	59.41	68	42	1.192
sp|P28482|MK01_HUMAN	MAPK1	Mitogen-activated protein kinase 1	37.03	37.26	72.5	26	1.192
sp|Q14168|MPP2_HUMAN	MPP2	MAGUK p55 subfamily member 2	11.41	16.79	43.4	13	1.192
sp|Q9H8Y5|ANKZ1_HUMAN	ANKZF1	Ankyrin repeat and zinc finger domain-containing protein 1	11.93	12.11	23.8	8	1.193
sp|Q96EH3|MASU1_HUMAN	MALSU1	Mitochondrial assembly of ribosomal large subunit protein 1	10.23	13.19	50	9	1.193
sp|Q9NUY8|TBC23_HUMAN	TBC1D23	TBC1 domain family member 23	21.07	21.67	33.8	14	1.193
sp|Q16719|KYNU_HUMAN	KYNU	Kynureninase	23.4	25.43	41.9	17	1.193
sp|Q7Z3B4|NUP54_HUMAN	NUP54	Nucleoporin p54	34.08	34.56	55.8	21	1.193
sp|Q96LA8|ANM6_HUMAN	PRMT6	Protein arginine N-methyltransferase 6	7.42	7.56	18.1	4	1.193
sp|Q9H4G0|E41L1_HUMAN	EPB41L1	Band 4.1-like protein 1	9.08	17.99	28.4	12	1.193
sp|Q99594|TEAD3_HUMAN	TEAD3	Transcriptional enhancer factor TEF-5	1.52	1.92	16.8	3	1.193
sp|Q96S97|MYADM_HUMAN	MYADM	Myeloid-associated differentiation marker	6	6	18.9	6	1.194
sp|Q8NHP8|PLBL2_HUMAN	PLBD2	Putative phospholipase B-like 2	10.39	10.8	27.3	7	1.194
sp|Q14152|EIF3A_HUMAN	EIF3A	Eukaryotic translation initiation factor 3 subunit A	106.09	109.89	54.2	77	1.195

sp|Q9BX95|SGPP1_HUMAN	SGPP1	Sphingosine-1-phosphate phosphatase 1	4.04	4.05	22.2	2	1.195
sp|O15085|ARHGB_HUMAN	ARHGEF11	Rho guanine nucleotide exchange factor 11	2.44	2.51	14.9	4	1.195
sp|Q658Y4|F91A1_HUMAN	FAM91A1	Protein FAM91A1	10.88	13.45	20.8	9	1.195
sp|Q8TB61|S35B2_HUMAN	SLC35B2	Adenosine 3'-phospho 5'-phosphosulfate transporter 1	12.04	12.1	18.8	7	1.195
sp|Q8IZL8|PELP1_HUMAN	PELP1	Proline-, glutamic acid- and leucine-rich protein 1	38.15	38.34	40.3	31	1.195
sp|Q9BV81|EMC6_HUMAN	EMC6	ER membrane protein complex subunit 6	4	4	23.6	2	1.195
sp|O15143|ARC1B_HUMAN	ARPC1B	Actin-related protein 2/3 complex subunit 1B	22.77	25.73	56.7	22	1.195
sp|P56282|DPOE2_HUMAN	POLE2	DNA polymerase epsilon subunit 2	12.21	12.33	40.6	7	1.195
sp|P50897|PPT1_HUMAN	PPT1	Palmitoyl-protein thioesterase 1	16.12	17.28	60.8	13	1.196
sp|Q9UHG3|PCYOX_HUMAN	PCYOX1	Prenylcysteine oxidase 1	31.94	32.9	56.4	31	1.196
sp|P61204|ARF3_HUMAN	ARF3	ADP-ribosylation factor 3	2	24.25	92.3	37	1.197
sp|Q86WB0|NIPA_HUMAN	ZC3HC1	Nuclear-interacting partner of ALK	25.39	25.45	45.2	17	1.197
sp|Q8N371|KDM8_HUMAN	KDM8	Lysine-specific demethylase 8	1.77	2.93	20.2	4	1.197
sp|Q02413|DSG1_HUMAN	DSG1	Desmoglein-1	2.15	2.24	11.4	2	1.197
sp|Q9UKN8|TF3C4_HUMAN	GTF3C4	General transcription factor 3C polypeptide 4	51.47	55.52	58.8	35	1.197
sp|Q6PH81|CP087_HUMAN	C16orf87	UPF0547 protein C16orf87	3.96	4.04	34.4	2	1.197
sp|Q9UHQ9|NB5R1_HUMAN	CYB5R1	NADH-cytochrome b5 reductase 1	12.3	12.88	46.6	6	1.198
sp|Q86WR0|CCD25_HUMAN	CCDC25	Coiled-coil domain-containing protein 25	4.92	6.51	46.2	5	1.198
sp|Q69YH5|CDCA2_HUMAN	CDCA2	Cell division cycle-associated protein 2	12.39	12.99	31.5	9	1.198
sp|Q15274|NADC_HUMAN	QPRT	Nicotinate-nucleotide pyrophosphorylase [carboxylating]	13.92	13.98	51.5	13	1.198
sp|Q8IZ73|RUSD2_HUMAN	RPUSD2	RNA pseudouridylate synthase domain-containing protein 2	18.21	18.45	47.9	12	1.198
sp|Q96RR4|KKCC2_HUMAN	CAMKK2	Calcium/calmodulin-dependent protein kinase kinase 2	4.04	5.13	21.6	4	1.199
sp|Q70CQ2|UBP34_HUMAN	USP34	Ubiquitin carboxyl-terminal hydrolase 34	28.81	29.93	18.6	16	1.199
sp|P19623|SPEE_HUMAN	SRM	Spermidine synthase	31.53	32.02	82.8	23	1.199
sp|P25208|NFYB_HUMAN	NFYB	Nuclear transcription factor Y subunit beta	4.11	4.23	26.1	3	1.200
sp|Q8NBZ0|IN80E_HUMAN	INO80E	INO80 complex subunit E	4.01	4.01	20.9	2	1.200
sp|P62857|RS28_HUMAN	RPS28	40S ribosomal protein S28	7	7.04	43.5	6	1.200
sp|Q9NUG6|PDRG1_HUMAN	PDRG1	p53 and DNA damage-regulated protein 1	4.03	4.04	53.4	2	1.200
sp|P34741|SDC2_HUMAN	SDC2	Syndecan-2	2.78	2.88	23.4	3	1.200
sp|O00255|MEN1_HUMAN	MEN1	Menin	17.16	17.28	33.3	10	1.201
sp|Q9H9B1|EHMT1_HUMAN	EHMT1	Histone-lysine N-methyltransferase EHMT1	29.96	30.84	29.7	15	1.201
sp|Q9P244|LRFN1_HUMAN	LRFN1	Leucine-rich repeat and fibronectin type III domain-containing protein 1	1.72	1.85	8	2	1.202
sp|P02768|ALBU_HUMAN	ALB	Serum albumin	80.29	81.12	82.9	67	1.202
sp|B1AK53|ESPN_HUMAN	ESPN	Espin	6.95	7.03	14.3	6	1.202
sp|P20073|ANXA7_HUMAN	ANXA7	Annexin A7	30.52	33.14	41.4	24	1.202
sp|P63272|SPT4H_HUMAN	SUPT4H1	Transcription elongation factor SPT4	4.02	4.25	61.5	3	1.202
sp|Q8IXH7|NELFD_HUMAN	NELFCD	Negative elongation factor C/D	16.65	18.06	30	10	1.202
sp|Q8IZA0|K319L_HUMAN	KIAA0319L	Dyslexia-associated protein KIAA0319-like protein	9.37	9.72	18.2	5	1.202
sp|P02788|TRFL_HUMAN	LTF	Lactotransferrin	13.97	21.49	31.1	16	1.203
sp|Q86UY8|NT5D3_HUMAN	NT5DC3	5'-nucleotidase domain-containing protein 3	12.03	14.27	41.1	9	1.203
sp|Q8WXA9|SREK1_HUMAN	SREK1	Splicing regulatory glutamine/lysine-rich protein 1	6.94	7.42	28.9	8	1.203
sp|Q8WV99|ZFN2B_HUMAN	ZFAND2B	AN1-type zinc finger protein 2B	2.73	2.78	18.7	2	1.203
sp|O94887|FARP2_HUMAN	FARP2	FERM, RhoGEF and pleckstrin domain-containing protein 2	9.8	10.76	18.2	7	1.203
sp|P35568|IRS1_HUMAN	IRS1	Insulin receptor substrate 1	1.74	1.86	6	2	1.203
sp|O15530|PDPK1_HUMAN	PDPK1	3-phosphoinositide-dependent protein kinase 1	9.76	11.51	33.5	9	1.203
sp|Q9BW27|NUP85_HUMAN	NUP85	Nuclear pore complex protein Nup85	42	42.74	48.5	24	1.203
sp|O43716|GATC_HUMAN	GATC	Glutamyl-tRNA(Gln) amidotransferase subunit C, mitochondrial	12.28	12.38	80.2	6	1.203
sp|O95376|ARI2_HUMAN	ARIH2	E3 ubiquitin-protein ligase ARIH2	23.74	23.83	45	13	1.203
sp|Q9H0V9|LMA2L_HUMAN	LMAN2L	VIP36-like protein	18.33	18.43	44.5	10	1.204

sp|Q9Y6N5|SQRD_HUMAN	SQRDL	Sulfide:quinone oxidoreductase, mitochondrial	31.67	31.84	62.7	18	1.204
sp|Q15843|NEDD8_HUMAN	NEDD8	NEDD8	8.66	9.07	59.3	4	1.204
sp|Q9Y4G8|RPGF2_HUMAN	RAPGEF2	Rap guanine nucleotide exchange factor 2	8.41	8.88	17	6	1.204
sp|Q14BN4|SLMAP_HUMAN	SLMAP	Sarcolemmal membrane-associated protein	21.66	22.16	42.9	12	1.204
sp|P04080|CYTB_HUMAN	CSTB	Cystatin-B	5.11	5.24	100	14	1.204
sp|Q14156|EFR3A_HUMAN	EFR3A	Protein EFR3 homolog A	6.7	10	23.8	7	1.205
sp|Q5VTQ0|TT39B_HUMAN	TTC39B	Tetratricopeptide repeat protein 39B	3.92	4.64	21.9	5	1.205
sp|P38117|ETFB_HUMAN	ETFB	Electron transfer flavoprotein subunit beta	39.62	40.02	87.5	27	1.205
sp|O43402|EMC8_HUMAN	EMC8	ER membrane protein complex subunit 8	14.91	16.92	61	11	1.205
sp|Q7Z3U7|MON2_HUMAN	MON2	Protein MON2 homolog	16.43	16.85	21.1	13	1.205
sp|Q14980|NUMA1_HUMAN	NUMA1	Nuclear mitotic apparatus protein 1	171.5	174.86	68	130	1.206
sp|Q15070|OXA1L_HUMAN	OXA1L	Mitochondrial inner membrane protein OXA1L	14.47	14.63	23.5	9	1.206
sp|O14776|TCRG1_HUMAN	TCERG1	Transcription elongation regulator 1	44.55	45.89	33.5	26	1.206
sp|P00738|HPT_HUMAN	HP	Haptoglobin	15.81	15.89	37.7	8	1.206
sp|Q9Y3I1|FBX7_HUMAN	FBXO7	F-box only protein 7	12.7	12.83	26.8	7	1.206
sp|Q9H6R7|CB044_HUMAN	C2orf44	WD repeat and coiled-coil-containing protein C2orf44	7.22	8.17	24.8	5	1.206
sp|Q5T160|SYRM_HUMAN	RARS2	Probable arginine--tRNA ligase, mitochondrial	17.98	18.36	39.3	9	1.207
sp|Q96NB3|ZN830_HUMAN	ZNF830	Zinc finger protein 830	10.35	11.39	51.3	8	1.207
sp|P29474|NOS3_HUMAN	NOS3	Nitric oxide synthase, endothelial	11.63	12.07	13.3	7	1.207
sp|A0FGR8|ESYT2_HUMAN	ESYT2	Extended synaptotagmin-2	43.57	43.72	43.9	24	1.207
sp|Q9HCJ3|RAVR2_HUMAN	RAVER2	Ribonucleoprotein PTB-binding 2	4.97	6.05	14.3	4	1.207
sp|Q8TEQ0|SNX29_HUMAN	SNX29	Sorting nexin-29	2.07	3.07	18.8	3	1.207
sp|P33121|ACSL1_HUMAN	ACSL1	Long-chain-fatty-acid--CoA ligase 1	38.48	38.71	46.7	23	1.207
sp|O96006|ZBED1_HUMAN	ZBED1	Zinc finger BED domain-containing protein 1	7.49	7.68	27.7	4	1.208
sp|P10451|OSTP_HUMAN	SPP1	Osteopontin	3.52	3.59	16.9	2	1.208
sp|O75179|ANR17_HUMAN	ANKRD17	Ankyrin repeat domain-containing protein 17	24.04	55.91	24.9	30	1.208
sp|Q9Y6M1|IF2B2_HUMAN	IGF2BP2	Insulin-like growth factor 2 mRNA-binding protein 2	23.97	34.15	46.1	21	1.209
sp|O00462|MANBA_HUMAN	MANBA	Beta-mannosidase	10.78	13.06	24	6	1.210
sp|P00813|ADA_HUMAN	ADA	Adenosine deaminase	6.05	6.08	32.8	3	1.210
sp|Q9BSV6|SEN34_HUMAN	TSEN34	tRNA-splicing endonuclease subunit Sen34	14.04	14.21	46.5	9	1.210
sp|Q96CU9|FXRD1_HUMAN	FOXRED1	FAD-dependent oxidoreductase domain-containing protein 1	18.94	19.22	38.7	11	1.211
sp|Q8WUX1|S38A5_HUMAN	SLC38A5	Sodium-coupled neutral amino acid transporter 5	4.66	4.87	20.8	3	1.211
sp|Q53LP3|SWAHC_HUMAN	SOWAHC	Ankyrin repeat domain-containing protein SOWAHC	11.58	11.7	31.6	7	1.211
sp|Q9UIC8|LCMT1_HUMAN	LCMT1	Leucine carboxyl methyltransferase 1	14.26	14.48	44	8	1.211
sp|Q9H9A5|CNO10_HUMAN	CNOT10	CCR4-NOT transcription complex subunit 10	14.84	15.14	25.1	8	1.211
sp|Q9Y4C8|RBM19_HUMAN	RBM19	Probable RNA-binding protein 19	28.01	29.25	33.7	17	1.212
sp|O14893|GEMI2_HUMAN	GEMIN2	Gem-associated protein 2	10.15	11.08	48.2	7	1.212
sp|O14734|ACOT8_HUMAN	ACOT8	Acyl-coenzyme A thioesterase 8	4.34	4.4	30.1	3	1.212
sp|O43488|ARK72_HUMAN	AKR7A2	Aflatoxin B1 aldehyde reductase member 2	27.33	27.61	63.5	18	1.212
sp|Q5TAX3|TUT4_HUMAN	ZCCHC11	Terminal uridylyltransferase 4	6.05	6.32	16.7	7	1.213
sp|O00233|PSMD9_HUMAN	PSMD9	26S proteasome non-ATPase regulatory subunit 9	10.33	10.44	44.4	9	1.213
sp|P53602|MVD1_HUMAN	MVD	Diphosphomevalonate decarboxylase	16.66	16.74	39	14	1.213
sp|Q13490|BIRC2_HUMAN	BIRC2	Baculoviral IAP repeat-containing protein 2	3.39	3.48	17.6	2	1.213
sp|Q9UJV9|DDX41_HUMAN	DDX41	Probable ATP-dependent RNA helicase DDX41	43.7	44.45	60.1	26	1.213
sp|P28161|GSTM2_HUMAN	GSTM2	Glutathione S-transferase Mu 2	5	7.54	54.1	5	1.214
sp|O00506|STK25_HUMAN	STK25	Serine/threonine-protein kinase 25	2.01	15.28	36.4	10	1.214
sp|Q9UNS1|TIM_HUMAN	TIMELESS	Protein timeless homolog	20.66	22.16	27	15	1.214
sp|Q13618|CUL3_HUMAN	CUL3	Cullin-3	51.13	51.53	56.4	34	1.214
sp|Q96A65|EXOC4_HUMAN	EXOC4	Exocyst complex component 4	39.17	41.71	46.5	22	1.214

sp|P24468|COT2_HUMAN	NR2F2	COUP transcription factor 2	10.73	10.9	24.2	6	1.214
sp|Q13901|C1D_HUMAN	C1D	Nuclear nucleic acid-binding protein C1D	2.57	2.79	40.4	3	1.215
sp|Q68E01|INT3_HUMAN	INTS3	Integrator complex subunit 3	27.67	29.25	34.1	16	1.215
sp|Q9ULJ3|ZBT21_HUMAN	ZBTB21	Zinc finger and BTB domain-containing protein 21	2.11	2.25	16.4	2	1.215
sp|Q9P0Z9|SOX_HUMAN	PIPOX	Peroxisomal sarcosine oxidase	15.72	15.9	38.5	13	1.215
sp|O95630|STABP_HUMAN	STAMBP	STAM-binding protein	12.61	12.99	38.9	9	1.215
sp|P48436|SOX9_HUMAN	SOX9	Transcription factor SOX-9	2.33	2.38	14	2	1.215
sp|Q9BTC0|DIDO1_HUMAN	DIDO1	Death-inducer obliterator 1	46.99	47.86	29.6	25	1.215
sp|P08574|CY1_HUMAN	CYC1	Cytochrome c1, heme protein, mitochondrial	18.11	18.19	58.8	25	1.215
sp|O15254|ACOX3_HUMAN	ACOX3	Peroxisomal acyl-coenzyme A oxidase 3	16.13	16.27	30.3	9	1.216
sp|P30038|AL4A1_HUMAN	ALDH4A1	Delta-1-pyrroline-5-carboxylate dehydrogenase, mitochondrial	37	37.37	59	25	1.216
sp|P11717|MPRI_HUMAN	IGF2R	Cation-independent mannose-6-phosphate receptor	152.87	154.85	55.8	82	1.217
sp|Q96PV6|LENG8_HUMAN	LENG8	Leukocyte receptor cluster member 8	6.68	6.76	17.8	4	1.217
sp|Q9UBV2|SE1L1_HUMAN	SEL1L	Protein sel-1 homolog 1	37.65	37.82	50.6	27	1.218
sp|P61962|DCAF7_HUMAN	DCAF7	DDB1- and CUL4-associated factor 7	20.47	20.59	49.7	13	1.218
sp|Q13418|ILK_HUMAN	ILK	Integrin-linked protein kinase	26.49	26.72	50.4	17	1.219
sp|P04818|TYSY_HUMAN	TYMS	Thymidylate synthase	18.46	18.59	58.5	12	1.219
sp|Q2TAA2|IAH1_HUMAN	IAH1	Isoamyl acetate-hydrolyzing esterase 1 homolog	11.56	11.84	42.7	8	1.219
sp|P61923|COPZ1_HUMAN	COPZ1	Coatomer subunit zeta-1	14.12	14.48	54.8	13	1.219
sp|Q96RS6|NUDC1_HUMAN	NUDCD1	NudC domain-containing protein 1	36.88	37.09	60.2	22	1.219
sp|Q15149|PLEC_HUMAN	PLEC	Plectin	364.57	364.01	61.8	202	1.220
sp|Q86V21|AACS_HUMAN	AACS	Acetoacetyl-CoA synthetase	23.55	24.39	39.6	14	1.220
sp|Q16342|PDCD2_HUMAN	PDCD2	Programmed cell death protein 2	4.37	4.42	28.8	3	1.220
sp|P60763|RAC3_HUMAN	RAC3	Ras-related C3 botulinum toxin substrate 3	2.18	13.59	89.1	10	1.221
sp|P23297|S10A1_HUMAN	S100A1	Protein S100-A1	5.37	5.85	53.2	4	1.221
sp|P07203|GPX1_HUMAN	GPX1	Glutathione peroxidase 1	20.36	20.47	72.4	12	1.221
sp|Q8N1S5|S39AB_HUMAN	SLC39A11	Zinc transporter ZIP11	4	4.2	12.3	3	1.221
sp|Q96DF8|DGC14_HUMAN	DGCR14	Protein DGCR14	8.52	8.76	46.4	5	1.222
sp|P09884|DPOLA_HUMAN	POLA1	DNA polymerase alpha catalytic subunit	30.26	31.28	32.6	23	1.222
sp|Q9Y217|MTMR6_HUMAN	MTMR6	Myotubularin-related protein 6	6.02	6.04	17.4	3	1.222
sp|Q8WTV0|SCRB1_HUMAN	SCARB1	Scavenger receptor class B member 1	8.73	8.8	21.2	5	1.222
sp|Q9Y296|TPPC4_HUMAN	TRAPPC4	Trafficking protein particle complex subunit 4	13.33	13.58	56.2	9	1.222
sp|Q5SRE5|NU188_HUMAN	NUP188	Nucleoporin NUP188 homolog	59.31	64.73	36.5	37	1.222
sp|Q14764|MVP_HUMAN	MVP	Major vault protein	61.63	61.88	61.6	39	1.222
sp|P35222|CTNB1_HUMAN	CTNNB1	Catenin beta-1	57.64	58.25	58.6	49	1.222
sp|Q9Y5N6|ORC6_HUMAN	ORC6	Origin recognition complex subunit 6	9.32	9.44	38.1	7	1.223
sp|P04179|SODM_HUMAN	SOD2	Superoxide dismutase [Mn], mitochondrial	17.52	17.64	86.9	13	1.223
sp|Q9ULH0|KDIS_HUMAN	KIDINS220	Kinase D-interacting substrate of 220 kDa	40.49	41.49	35.1	24	1.224
sp|Q8N5B7|CERS5_HUMAN	CERS5	Ceramide synthase 5	3.42	3.66	23.2	6	1.224
sp|Q02040|AK17A_HUMAN	AKAP17A	A-kinase anchor protein 17A	18.56	21.59	36	10	1.224
sp|Q9UQ49|NEUR3_HUMAN	NEU3	Sialidase-3	3.15	3.26	10.5	3	1.225
sp|Q9BRT9|SLD5_HUMAN	GINS4	DNA replication complex GINS protein SLD5	15.39	16.05	61.9	8	1.225
sp|Q9NZN5|ARHGC_HUMAN	ARHGEF12	Rho guanine nucleotide exchange factor 12	7.7	8.5	15.4	8	1.225
sp|Q8N4Q0|ZADH2_HUMAN	ZADH2	Zinc-binding alcohol dehydrogenase domain-containing protein 2	10.21	10.32	40.9	6	1.225
sp|Q6BDS2|URFB1_HUMAN	UHRF1BP1	UHRF1-binding protein 1	3.53	3.99	15.4	3	1.226
sp|Q13510|ASAH1_HUMAN	ASAH1	Acid ceramidase	16.14	16.25	49.9	10	1.226
sp|Q6YP21|KAT3_HUMAN	CCBL2	Kynurenine--oxoglutarate transaminase 3	28.35	28.89	52	19	1.226
sp|Q8N5M1|ATPF2_HUMAN	ATPAF2	ATP synthase mitochondrial F1 complex assembly factor 2	12	12.03	33.2	7	1.226
sp|Q9H6Z4|RANB3_HUMAN	RANBP3	Ran-binding protein 3	18.47	19.63	39.2	14	1.226

sp|Q9H4A4|AMPB_HUMAN	RNPEP	Aminopeptidase B	49.13	49.52	66.5	29	1.227
sp|O43674|NDUB5_HUMAN	NDUFB5	NADH dehydrogenase [ubiquinone] 1 beta subcomplex subunit 5, mitochondrial	6.08	6.21	24.3	3	1.227
sp|Q92878|RAD50_HUMAN	RAD50	DNA repair protein RAD50	93.41	95.9	58.2	55	1.228
sp|Q8TEQ6|GEMI5_HUMAN	GEMIN5	Gem-associated protein 5	66.65	68.85	37.7	39	1.228
sp|P19404|NDUV2_HUMAN	NDUFV2	NADH dehydrogenase [ubiquinone] flavoprotein 2, mitochondrial	25.27	25.37	62.3	20	1.229
sp|Q9BV86|NTM1A_HUMAN	NTMT1	N-terminal Xaa-Pro-Lys N-methyltransferase 1	9.43	9.55	47.5	8	1.229
sp|Q8TCF1|ZFAN1_HUMAN	ZFAND1	AN1-type zinc finger protein 1	6.71	6.9	33.6	4	1.229
sp|Q9H299|SH3L3_HUMAN	SH3BGRL3	SH3 domain-binding glutamic acid-rich-like protein 3	6.81	6.89	79.6	5	1.229
sp|Q9NR77|PXMP2_HUMAN	PXMP2	Peroxisomal membrane protein 2	2.08	2.51	23.1	3	1.230
sp|Q53H96|P5CR3_HUMAN	PYCRL	Pyrroline-5-carboxylate reductase 3	15.21	15.35	44.2	9	1.230
sp|Q9BXW7|CECR5_HUMAN	CECR5	Cat eye syndrome critical region protein 5	19.85	20.15	61.9	22	1.230
sp|Q9NS00|C1GLT_HUMAN	C1GALT1	Glycoprotein-N-acetylgalactosamine 3-beta-galactosyltransferase 1	4.36	4.47	28.7	3	1.230
sp|Q7L8W6|DPH6_HUMAN	DPH6	Diphthine--ammonia ligase	2.01	2.03	22.9	2	1.230
sp|Q9NV92|NFIP2_HUMAN	NDFIP2	NEDD4 family-interacting protein 2	2.2	2.97	22.3	2	1.231
sp|Q96G46|DUS3L_HUMAN	DUS3L	tRNA-dihydrouridine(47) synthase [NAD(P)(+)]-like	32.91	35.13	52.2	17	1.231
sp|Q8IWX8|CHERP_HUMAN	CHERP	Calcium homeostasis endoplasmic reticulum protein	39.81	40.08	47.4	30	1.231
sp|Q16698|DECR_HUMAN	DECR1	2,4-dienoyl-CoA reductase, mitochondrial	21.37	21.54	57.9	17	1.231
sp|Q9BSE5|SPEB_HUMAN	AGMAT	Agmatinase, mitochondrial	33.38	33.66	73.6	23	1.232
sp|O75665|OFD1_HUMAN	OFD1	Oral-facial-digital syndrome 1 protein	1.95	2.9	20.7	3	1.232
sp|Q96DV4|RM38_HUMAN	MRPL38	39S ribosomal protein L38, mitochondrial	26.51	26.63	47.1	17	1.232
sp|P06576|ATPB_HUMAN	ATP5B	ATP synthase subunit beta, mitochondrial	69.92	71.22	83	209	1.233
sp|Q6PJT7|ZC3HE_HUMAN	ZC3H14	Zinc finger CCCH domain-containing protein 14	31.97	32.74	55.4	22	1.233
sp|Q6PI98|IN80C_HUMAN	INO80C	INO80 complex subunit C	3.92	4.03	32.8	2	1.234
sp|A1L0T0|ILVBL_HUMAN	ILVBL	Acetolactate synthase-like protein	41.44	43.13	59.5	28	1.234
sp|Q6VY07|PACS1_HUMAN	PACS1	Phosphofurin acidic cluster sorting protein 1	8.49	8.82	17.1	6	1.234
sp|Q9H6R0|DHX33_HUMAN	DHX33	Putative ATP-dependent RNA helicase DHX33	32.69	33.87	45.5	17	1.234
sp|Q9NT62|ATG3_HUMAN	ATG3	Ubiquitin-like-conjugating enzyme ATG3	13.22	13.87	43	8	1.235
sp|Q9H469|FXL15_HUMAN	FBXL15	F-box/LRR-repeat protein 15	2.5	2.53	15	2	1.235
sp|Q96RU3|FNBP1_HUMAN	FNBP1	Formin-binding protein 1	6.97	7.51	28	4	1.235
sp|Q15102|PA1B3_HUMAN	PAFAH1B3	Platelet-activating factor acetylhydrolase IB subunit gamma	12.3	12.66	58.4	11	1.236
sp|Q06787|FMR1_HUMAN	FMR1	Fragile X mental retardation protein 1	29.6	36.42	57.4	26	1.236
sp|Q9Y496|KIF3A_HUMAN	KIF3A	Kinesin-like protein KIF3A	9.21	13.27	34.2	9	1.236
sp|Q9Y2K7|KDM2A_HUMAN	KDM2A	Lysine-specific demethylase 2A	22.81	23.11	25.2	14	1.237
sp|Q8WUD1|RAB2B_HUMAN	RAB2B	Ras-related protein Rab-2B	5.24	19.04	62.5	13	1.237
sp|Q9P2L0|WDR35_HUMAN	WDR35	WD repeat-containing protein 35	2.24	2.65	16.6	3	1.237
sp|P47985|UCRI_HUMAN	UQCRFS1	Cytochrome b-c1 complex subunit Rieske, mitochondrial	28.23	28.46	70.4	29	1.237
sp|Q8N6M3|FITM2_HUMAN	FITM2	Fat storage-inducing transmembrane protein 2	2.97	3.04	24.1	2	1.237
sp|P29590|PML_HUMAN	PML	Protein PML	11.21	11.84	21.5	7	1.238
sp|Q8NCE2|MTMRE_HUMAN	MTMR14	Myotubularin-related protein 14	8.44	8.6	20.9	5	1.238
sp|O75821|EIF3G_HUMAN	EIF3G	Eukaryotic translation initiation factor 3 subunit G	31.02	34.68	72.5	29	1.238
sp|Q9NYU2|UGGG1_HUMAN	UGGT1	UDP-glucose:glycoprotein glucosyltransferase 1	117.03	117.75	65	92	1.238
sp|P09455|RET1_HUMAN	RBP1	Retinol-binding protein 1	11.32	11.85	75.6	7	1.238
sp|P09382|LEG1_HUMAN	LGALS1	Galectin-1	16.23	16.3	91.1	21	1.239
sp|Q9P2N5|RBM27_HUMAN	RBM27	RNA-binding protein 27	18.77	22.01	29.8	14	1.239
sp|O15084|ANR28_HUMAN	ANKRD28	Serine/threonine-protein phosphatase 6 regulatory ankyrin repeat subunit A	21.52	21.92	23.5	15	1.239
sp|Q13409|DC1I2_HUMAN	DYNC1I2	Cytoplasmic dynein 1 intermediate chain 2	30.09	30.2	47.8	27	1.240
sp|A5YKK6|CNOT1_HUMAN	CNOT1	CCR4-NOT transcription complex subunit 1	98.87	103.22	39.9	60	1.241
sp|P34059|GALNS_HUMAN	GALNS	N-acetylgalactosamine-6-sulfatase	2.17	2.21	12.5	4	1.241
sp|Q9Y3P9|RBGP1_HUMAN	RABGAP1	Rab GTPase-activating protein 1	24.58	28.28	32.5	20	1.241

sp|O75326|SEM7A_HUMAN	SEMA7A	Semaphorin-7A	3.17	3.32	13.5	3	1.241
sp|Q8WXH0|SYNE2_HUMAN	SYNE2	Nesprin-2	82.27	93.68	29.1	56	1.241
sp|Q5TC12|ATPF1_HUMAN	ATPAF1	ATP synthase mitochondrial F1 complex assembly factor 1	18.33	18.51	53.4	13	1.241
sp|Q96B97|SH3K1_HUMAN	SH3KBP1	SH3 domain-containing kinase-binding protein 1	7.51	7.8	24.4	5	1.241
sp|O75695|XRP2_HUMAN	RP2	Protein XRP2	12.39	12.53	42.6	7	1.241
sp|P05141|ADT2_HUMAN	SLC25A5	ADP/ATP translocase 2	57.58	58.03	81.5	87	1.241
sp|Q99447|PCY2_HUMAN	PCYT2	Ethanolamine-phosphate cytidylyltransferase	30.56	32.13	63.5	18	1.242
sp|P06703|S10A6_HUMAN	S100A6	Protein S100-A6	5.34	5.46	58.9	4	1.242
sp|Q9NVH0|EXD2_HUMAN	EXD2	Exonuclease 3'-5' domain-containing protein 2	16.65	17.24	39	9	1.242
sp|P46952|3HAO_HUMAN	HAAO	3-hydroxyanthranilate 3,4-dioxygenase	12.58	12.67	58	7	1.242
sp|Q96A33|CCD47_HUMAN	CCDC47	Coiled-coil domain-containing protein 47	37.87	38.01	52.8	34	1.243
sp|Q96MY1|NOL4L_HUMAN	NOL4L	Nucleolar protein 4-like	2.52	2.68	14	2	1.243
sp|P28340|DPOD1_HUMAN	POLD1	DNA polymerase delta catalytic subunit	64.24	67.13	60.1	37	1.244
sp|Q9H000|MKRN2_HUMAN	MKRN2	Probable E3 ubiquitin-protein ligase makorin-2	11.85	11.93	27.6	6	1.244
sp|Q99996|AKAP9_HUMAN	AKAP9	A-kinase anchor protein 9	40.65	49.09	30.8	32	1.244
sp|Q9UBU9|NXF1_HUMAN	NXF1	Nuclear RNA export factor 1	40.74	40.82	52.8	27	1.244
sp|O75151|PHF2_HUMAN	PHF2	Lysine-specific demethylase PHF2	7.7	14.3	25.2	9	1.245
sp|Q99816|TS101_HUMAN	TSG101	Tumor susceptibility gene 101 protein	17.92	18.39	39	13	1.245
sp|Q9NRW7|VPS45_HUMAN	VPS45	Vacuolar protein sorting-associated protein 45	23.37	23.59	48.6	13	1.245
sp|P55735|SEC13_HUMAN	SEC13	Protein SEC13 homolog	18.84	21.35	60.3	18	1.245
sp|Q96KN1|FA84B_HUMAN	FAM84B	Protein FAM84B	10.49	10.77	49	7	1.245
sp|P16435|NCPR_HUMAN	POR	NADPH--cytochrome P450 reductase	66.23	67.89	75.5	58	1.245
sp|P08237|PFKAM_HUMAN	PFKM	ATP-dependent 6-phosphofructokinase, muscle type	25.37	30.97	44.1	22	1.245
sp|O95486|SC24A_HUMAN	SEC24A	Protein transport protein Sec24A	28.48	28.64	23.2	15	1.246
sp|Q16877|F264_HUMAN	PFKFB4	6-phosphofructo-2-kinase/fructose-2,6-bisphosphatase 4	1.5	6.09	37.5	4	1.246
sp|P78540|ARGI2_HUMAN	ARG2	Arginase-2, mitochondrial	17.76	18.09	57.9	13	1.246
sp|Q9P1Y6|PHRF1_HUMAN	PHRF1	PHD and RING finger domain-containing protein 1	4.1	4.96	12	3	1.247
sp|Q92887|MRP2_HUMAN	ABCC2	Canalicular multispecific organic anion transporter 1	28.8	32.84	27.7	22	1.247
sp|Q96D53|ADCK4_HUMAN	ADCK4	AarF domain-containing protein kinase 4	8.67	8.74	28.3	7	1.247
sp|Q92538|GBF1_HUMAN	GBF1	Golgi-specific brefeldin A-resistance guanine nucleotide exchange factor 1	43.55	46.96	31.7	28	1.247
sp|O43598|DNPH1_HUMAN	DNPH1	2'-deoxynucleoside 5'-phosphate N-hydrolase 1	9.46	11.61	61.5	9	1.248
sp|O94913|PCF11_HUMAN	PCF11	Pre-mRNA cleavage complex 2 protein Pcf11	4.27	4.47	13.7	3	1.248
sp|Q9BPX6|MICU1_HUMAN	MICU1	Calcium uptake protein 1, mitochondrial	15.17	15.33	46.2	10	1.248
sp|Q9NUQ8|ABCF3_HUMAN	ABCF3	ATP-binding cassette sub-family F member 3	31.64	33.99	53.5	23	1.248
sp|Q9P000|COMD9_HUMAN	COMMD9	COMM domain-containing protein 9	15.59	17.03	68.2	12	1.249
sp|Q53EP0|FND3B_HUMAN	FNDC3B	Fibronectin type III domain-containing protein 3B	36.49	36.57	35.8	19	1.249
sp|Q9BYK8|HELZ2_HUMAN	HELZ2	Helicase with zinc finger domain 2	8.69	8.87	13.1	6	1.249
sp|P16422|EPCAM_HUMAN	EPCAM	Epithelial cell adhesion molecule	16.03	16.13	43.6	13	1.250
sp|Q7Z2K8|GRIN1_HUMAN	GPRIN1	G protein-regulated inducer of neurite outgrowth 1	10.85	11.16	28.4	6	1.250
sp|Q9H0M0|WWP1_HUMAN	WWP1	NEDD4-like E3 ubiquitin-protein ligase WWP1	1.67	4.63	16.2	4	1.250
sp|P02750|A2GL_HUMAN	LRG1	Leucine-rich alpha-2-glycoprotein	2	3.33	11.8	2	1.251
sp|O95757|HS74L_HUMAN	HSPA4L	Heat shock 70 kDa protein 4L	57.51	75.48	71.2	53	1.251
sp|Q96PK6|RBM14_HUMAN	RBM14	RNA-binding protein 14	47.78	48.78	47.1	50	1.251
sp|O95235|KI20A_HUMAN	KIF20A	Kinesin-like protein KIF20A	19.72	21.5	40.8	17	1.251
sp|O00429|DNM1L_HUMAN	DNM1L	Dynamin-1-like protein	44.64	45.69	59.5	28	1.251
sp|Q96FC7|PHIPL_HUMAN	PHYHIPL	Phytanoyl-CoA hydroxylase-interacting protein-like	11.61	11.7	39.1	8	1.252
sp|Q9NQW7|XPP1_HUMAN	XPNPEP1	Xaa-Pro aminopeptidase 1	28.55	30.21	47.5	22	1.252
sp|Q9NP66|HM20A_HUMAN	HMG20A	High mobility group protein 20A	3.89	4.09	32	2	1.252
sp|Q08209|PP2BA_HUMAN	PPP3CA	Serine/threonine-protein phosphatase 2B catalytic subunit alpha isoform	16.46	20.91	44	14	1.253

sp|Q96GC5|RM48_HUMAN	MRPL48	39S ribosomal protein L48, mitochondrial	7.46	7.53	28.3	4	1.253
sp|O94762|RECQ5_HUMAN	RECQL5	ATP-dependent DNA helicase Q5	2.67	2.81	17.4	4	1.253
sp|Q9HA77|SYCM_HUMAN	CARS2	Probable cysteine--tRNA ligase, mitochondrial	22.76	23.09	36.5	15	1.254
sp|P54105|ICLN_HUMAN	CLNS1A	Methylosome subunit pICln	13.13	13.23	57.4	15	1.254
sp|Q8IWA5|CTL2_HUMAN	SLC44A2	Choline transporter-like protein 2	1.62	1.82	15.4	2	1.254
sp|Q08380|LG3BP_HUMAN	LGALS3BP	Galectin-3-binding protein	25.2	27.52	43.8	17	1.255
sp|Q14978|NOLC1_HUMAN	NOLC1	Nucleolar and coiled-body phosphoprotein 1	53.71	54.06	48.9	33	1.255
sp|P33981|TTK_HUMAN	TTK	Dual specificity protein kinase TTK	10.35	11.79	29.5	10	1.255
sp|Q16762|THTR_HUMAN	TST	Thiosulfate sulfurtransferase	25.15	25.2	60.3	17	1.255
sp|P43304|GPDM_HUMAN	GPD2	Glycerol-3-phosphate dehydrogenase, mitochondrial	28.51	28.64	39.8	17	1.255
sp|Q86UA1|PRP39_HUMAN	PRPF39	Pre-mRNA-processing factor 39	8.77	9.64	21.5	5	1.255
sp|Q15746|MYLK_HUMAN	MYLK	Myosin light chain kinase, smooth muscle	14.34	17.14	17.5	10	1.256
sp|Q9BW85|CCD94_HUMAN	CCDC94	Coiled-coil domain-containing protein 94	13.16	15.43	50.5	9	1.256
sp|P41208|CETN2_HUMAN	CETN2	Centrin-2	6.05	6.48	48.3	7	1.256
sp|O75191|XYLB_HUMAN	XYLB	Xylulose kinase	22.58	22.75	49.4	15	1.256
sp|Q9BQA1|MEP50_HUMAN	WDR77	Methylosome protein 50	26.48	27.61	66.1	23	1.256
sp|P82921|RT21_HUMAN	MRPS21	28S ribosomal protein S21, mitochondrial	6.48	6.55	55.2	5	1.257
sp|Q9ULT0|TTC7A_HUMAN	TTC7A	Tetratricopeptide repeat protein 7A	2.01	2.04	16.6	3	1.257
sp|O95861|BPNT1_HUMAN	BPNT1	3'(2'),5'-bisphosphate nucleotidase 1	28.31	28.49	77.6	20	1.257
sp|O75251|NDUS7_HUMAN	NDUFS7	NADH dehydrogenase [ubiquinone] iron-sulfur protein 7, mitochondrial	10.06	10.16	47	10	1.257
sp|Q765P7|MTSSL_HUMAN	MTSS1L	MTSS1-like protein	8.38	9.07	19.5	7	1.257
sp|Q8NI22|MCFD2_HUMAN	MCFD2	Multiple coagulation factor deficiency protein 2	8	8	80.1	5	1.258
sp|P78536|ADA17_HUMAN	ADAM17	Disintegrin and metalloproteinase domain-containing protein 17	13.71	13.97	30	8	1.258
sp|Q96SB3|NEB2_HUMAN	PPP1R9B	Neurabin-2	22.71	23.27	36.1	13	1.258
sp|Q8WV93|LACE1_HUMAN	LACE1	Lactation elevated protein 1	6.17	6.38	29.3	3	1.259
sp|O43678|NDUA2_HUMAN	NDUFA2	NADH dehydrogenase [ubiquinone] 1 alpha subcomplex subunit 2	7.62	8.32	64.7	8	1.259
sp|Q9NR12|PDLI7_HUMAN	PDLIM7	PDZ and LIM domain protein 7	14.64	14.89	43.3	12	1.259
sp|Q9Y3E2|BOLA1_HUMAN	BOLA1	BolA-like protein 1	6	6.01	33.6	4	1.259
sp|Q9UN37|VPS4A_HUMAN	VPS4A	Vacuolar protein sorting-associated protein 4A	27.03	27.38	60.6	17	1.259
sp|O75427|LRCH4_HUMAN	LRCH4	Leucine-rich repeat and calponin homology domain-containing protein 4	7.56	7.73	26.2	7	1.259
sp|P63027|VAMP2_HUMAN	VAMP2	Vesicle-associated membrane protein 2	11.25	11.39	71.6	12	1.260
sp|Q9NXB9|ELOV2_HUMAN	ELOVL2	Elongation of very long chain fatty acids protein 2	3.79	3.88	15.9	3	1.260
sp|Q9NYV4|CDK12_HUMAN	CDK12	Cyclin-dependent kinase 12	20.28	24.96	23.9	13	1.260
sp|O00764|PDXK_HUMAN	PDXK	Pyridoxal kinase	28.1	28.17	62.5	19	1.260
sp|Q9Y294|ASF1A_HUMAN	ASF1A	Histone chaperone ASF1A	8.13	8.22	48	6	1.261
sp|P43155|CACP_HUMAN	CRAT	Carnitine O-acetyltransferase	19.61	20.72	34	14	1.262
sp|Q7Z2T5|TRM1L_HUMAN	TRMT1L	TRMT1-like protein	24.33	25.56	38.1	15	1.262
sp|Q9BZV1|UBXN6_HUMAN	UBXN6	UBX domain-containing protein 6	14.14	14.35	43.3	8	1.262
sp|Q8TAA5|GRPE2_HUMAN	GRPEL2	GrpE protein homolog 2, mitochondrial	5.87	8.58	52	6	1.262
sp|Q9UBU8|MO4L1_HUMAN	MORF4L1	Mortality factor 4-like protein 1	19.24	19.27	40.6	10	1.263
sp|O14777|NDC80_HUMAN	NDC80	Kinetochore protein NDC80 homolog	20.48	21.68	43.2	14	1.263
sp|P01023|A2MG_HUMAN	A2M	Alpha-2-macroglobulin	70.94	72.56	48.9	45	1.263
sp|Q9BUN8|DERL1_HUMAN	DERL1	Derlin-1	5.85	5.92	19.9	4	1.263
sp|Q13356|PPIL2_HUMAN	PPIL2	Peptidyl-prolyl cis-trans isomerase-like 2	16.3	16.42	37.5	9	1.264
sp|Q9UH92|MLX_HUMAN	MLX	Max-like protein X	2.36	2.4	30.5	2	1.264
sp|Q9ULJ8|NEB1_HUMAN	PPP1R9A	Neurabin-1	4.84	5.6	14.8	4	1.265
sp|P49590|SYHM_HUMAN	HARS2	Probable histidine--tRNA ligase, mitochondrial	19.1	31.89	56.5	18	1.265
sp|Q96DM3|MIC1_HUMAN	C18orf8	Uncharacterized protein C18orf8	12.75	13.25	34.4	11	1.265
sp|O96028|NSD2_HUMAN	WHSC1	Histone-lysine N-methyltransferase NSD2	26.99	27.97	29.3	16	1.266

sp|Q6P4A7|SFXN4_HUMAN	SFXN4	Sideroflexin-4	8.23	9.64	33.2	6	1.267
sp|Q9Y446|PKP3_HUMAN	PKP3	Plakophilin-3	19.76	19.91	34.1	12	1.267
sp|O43264|ZW10_HUMAN	ZW10	Centromere/kinetochore protein zw10 homolog	25.51	26.7	48.8	18	1.267
sp|Q9H0T7|RAB17_HUMAN	RAB17	Ras-related protein Rab-17	3.2	6.66	47.2	6	1.267
sp|Q9ULV3|CIZ1_HUMAN	CIZ1	Cip1-interacting zinc finger protein	12.63	12.94	24.6	9	1.267
sp|Q14517|FAT1_HUMAN	FAT1	Protocadherin Fat 1	13.64	16.51	13.6	11	1.267
sp|Q9HCN8|SDF2L_HUMAN	SDF2L1	Stromal cell-derived factor 2-like protein 1	8.86	8.93	63.4	13	1.269
sp|Q9BYD3|RM04_HUMAN	MRPL4	39S ribosomal protein L4, mitochondrial	21.55	22.74	71.4	14	1.269
sp|Q92540|SMG7_HUMAN	SMG7	Protein SMG7	2.31	3.85	14.9	3	1.269
sp|Q13438|OS9_HUMAN	OS9	Protein -9	27.88	29.7	62.5	20	1.269
sp|Q15020|SART3_HUMAN	SART3	Squamous cell carcinoma antigen recognized by T-cells 3	59.36	60.62	54.2	37	1.270
sp|Q9NRK6|ABCBA_HUMAN	ABCB10	ATP-binding cassette sub-family B member 10, mitochondrial	31.88	32.38	37.7	19	1.270
sp|Q9Y4P3|TBL2_HUMAN	TBL2	Transducin beta-like protein 2	32.44	32.86	49.4	21	1.270
sp|Q9Y6A9|SPCS1_HUMAN	SPCS1	Signal peptidase complex subunit 1	2.96	3.11	31.4	2	1.270
sp|Q9H910|HN1L_HUMAN	HN1L	Hematological and neurological expressed 1-like protein	20.53	20.59	84.2	12	1.270
sp|Q9H4L5|OSBL3_HUMAN	OSBPL3	Oxysterol-binding protein-related protein 3	17.13	18.01	30.4	10	1.271
sp|P42785|PCP_HUMAN	PRCP	Lysosomal Pro-X carboxypeptidase	9.25	9.38	23.4	6	1.271
sp|P56211|ARP19_HUMAN	ARPP19	cAMP-regulated phosphoprotein 19	2.6	4.74	75	4	1.271
sp|Q8N9V3|WSDU1_HUMAN	WDSUB1	WD repeat, SAM and U-box domain-containing protein 1	8.02	8.04	27.1	5	1.271
sp|P23193|TCEA1_HUMAN	TCEA1	Transcription elongation factor A protein 1	28.3	28.75	72.4	23	1.271
sp|Q9Y295|DRG1_HUMAN	DRG1	Developmentally-regulated GTP-binding protein 1	30.6	30.85	61.9	20	1.271
sp|P83876|TXN4A_HUMAN	TXNL4A	Thioredoxin-like protein 4A	11.74	11.85	70.4	9	1.271
sp|Q8TDD1|DDX54_HUMAN	DDX54	ATP-dependent RNA helicase DDX54	61.57	62.16	57.8	43	1.272
sp|P24752|THIL_HUMAN	ACAT1	Acetyl-CoA acetyltransferase, mitochondrial	59.2	60.04	85.3	78	1.272
sp|Q92917|GPKOW_HUMAN	GPKOW	G patch domain and KOW motifs-containing protein	30.43	30.86	68.5	22	1.272
sp|Q8IWF2|FXRD2_HUMAN	FOXRED2	FAD-dependent oxidoreductase domain-containing protein 2	2.02	2.06	9.9	2	1.272
sp|P19022|CADH2_HUMAN	CDH2	Cadherin-2	23.5	23.67	34.6	18	1.272
sp|P05362|ICAM1_HUMAN	ICAM1	Intercellular adhesion molecule 1	22.04	22.06	37.6	13	1.273
sp|Q5JSL3|DOC11_HUMAN	DOCK11	Dedicator of cytokinesis protein 11	3.68	4.75	14.8	6	1.273
sp|Q9H967|WDR76_HUMAN	WDR76	WD repeat-containing protein 76	4.6	4.87	14.9	3	1.274
sp|Q9BV29|CO057_HUMAN	C15orf57	Uncharacterized protein C15orf57	2.74	2.9	32.4	2	1.275
sp|Q92989|CLP1_HUMAN	CLP1	Polyribonucleotide 5'-hydroxyl-kinase Clp1	4	4.07	20.2	2	1.275
sp|Q6PML9|ZNT9_HUMAN	SLC30A9	Zinc transporter 9	12.88	13.73	31.2	9	1.275
sp|P23229|ITA6_HUMAN	ITGA6	Integrin alpha-6	27.71	28.74	34	17	1.275
sp|Q8TAE8|G45IP_HUMAN	GADD45GIP1	Growth arrest and DNA damage-inducible proteins-interacting protein 1	17.12	17.29	65.8	11	1.276
sp|Q9Y289|SC5A6_HUMAN	SLC5A6	Sodium-dependent multivitamin transporter	8.67	8.84	16.5	6	1.276
sp|Q9NPA0|EMC7_HUMAN	EMC7	ER membrane protein complex subunit 7	12.19	12.5	65.3	9	1.276
sp|Q9NQ48|LZTL1_HUMAN	LZTFL1	Leucine zipper transcription factor-like protein 1	9.64	9.85	49.2	7	1.276
sp|Q99471|PFD5_HUMAN	PFDN5	Prefoldin subunit 5	18	18.02	81.8	15	1.277
sp|Q9Y2H0|DLGP4_HUMAN	DLGAP4	Disks large-associated protein 4	7.29	7.63	16.3	5	1.277
sp|Q96DA6|TIM14_HUMAN	DNAJC19	Mitochondrial import inner membrane translocase subunit TIM14	8.71	8.76	53.5	5	1.278
sp|Q562E7|WDR81_HUMAN	WDR81	WD repeat-containing protein 81	6.09	6.21	11.7	4	1.278
sp|P14373|TRI27_HUMAN	TRIM27	Zinc finger protein RFP	11.85	11.94	25.7	6	1.278
sp|O00515|LAD1_HUMAN	LAD1	Ladinin-1	26.47	26.91	50.1	14	1.278
sp|Q8NBQ5|DHB11_HUMAN	HSD17B11	Estradiol 17-beta-dehydrogenase 11	27.78	28.44	64.7	17	1.278
sp|Q8N5C6|SRBD1_HUMAN	SRBD1	S1 RNA-binding domain-containing protein 1	27.13	29.76	33.1	18	1.279
sp|Q13610|PWP1_HUMAN	PWP1	Periodic tryptophan protein 1 homolog	15.56	15.73	35.3	11	1.279
sp|Q9Y5Q8|TF3C5_HUMAN	GTF3C5	General transcription factor 3C polypeptide 5	28.79	29.54	51.6	17	1.279
sp|P49321|NASP_HUMAN	NASP	Nuclear autoantigenic sperm protein	89.05	91.25	83.6	90	1.279

sp|Q9UJT0|TBE_HUMAN	TUBE1	Tubulin epsilon chain	4.01	4.02	15.6	2	1.279
sp|P35914|HMGCL_HUMAN	HMGCL	Hydroxymethylglutaryl-CoA lyase, mitochondrial	15.54	16.81	41.2	10	1.280
sp|Q8NFX7|STXB6_HUMAN	STXBP6	Syntaxin-binding protein 6	1.6	1.76	28.6	2	1.280
sp|Q8TDH9|BL1S5_HUMAN	BLOC1S5	Biogenesis of lysosome-related organelles complex 1 subunit 5	3.17	3.53	29.4	3	1.280
sp|P55039|DRG2_HUMAN	DRG2	Developmentally-regulated GTP-binding protein 2	27.71	28.44	57.4	14	1.281
sp|Q9UJ70|NAGK_HUMAN	NAGK	N-acetyl-D-glucosamine kinase	13.52	13.66	41	10	1.281
sp|Q13907|IDI1_HUMAN	IDI1	Isopentenyl-diphosphate Delta-isomerase 1	18.2	19.62	75.8	19	1.281
sp|A0MZ66|SHOT1_HUMAN	SHTN1	Shootin-1	30.4	31.68	52.1	18	1.281
sp|Q15042|RB3GP_HUMAN	RAB3GAP1	Rab3 GTPase-activating protein catalytic subunit	33.01	33.78	45.5	18	1.281
sp|P49257|LMAN1_HUMAN	LMAN1	Protein ERGIC-53	31.62	34.05	60.4	42	1.282
sp|P01009|A1AT_HUMAN	SERPINA1	Alpha-1-antitrypsin	28.03	29.92	61.2	29	1.282
sp|Q0VDF9|HSP7E_HUMAN	HSPA14	Heat shock 70 kDa protein 14	21.76	22.57	57	13	1.283
sp|P01011|AACT_HUMAN	SERPINA3	Alpha-1-antichymotrypsin	13.89	14.29	40.4	15	1.284
sp|Q86VV8|RTTN_HUMAN	RTTN	Rotatin	4.3	7.99	13.4	9	1.284
sp|P51148|RAB5C_HUMAN	RAB5C	Ras-related protein Rab-5C	19.08	20.4	82.9	48	1.284
sp|Q05048|CSTF1_HUMAN	CSTF1	Cleavage stimulation factor subunit 1	19.57	19.72	47.1	14	1.284
sp|Q9Y4W2|LAS1L_HUMAN	LAS1L	Ribosomal biogenesis protein LAS1L	33.46	33.87	48.9	23	1.284
sp|Q12907|LMAN2_HUMAN	LMAN2	Vesicular integral-membrane protein VIP36	26.15	26.2	61	26	1.284
sp|Q9UNI6|DUS12_HUMAN	DUSP12	Dual specificity protein phosphatase 12	11.19	11.4	43.2	6	1.285
sp|Q9Y232|CDYL1_HUMAN	CDYL	Chromodomain Y-like protein	9.21	9.59	40.6	7	1.285
sp|Q9UKM7|MA1B1_HUMAN	MAN1B1	Endoplasmic reticulum mannosyl-oligosaccharide 1,2-alpha-mannosidase	16.22	16.26	28.5	8	1.285
sp|Q96JQ2|CLMN_HUMAN	CLMN	Calmin	2.65	2.73	13	3	1.286
sp|P48382|RFX5_HUMAN	RFX5	DNA-binding protein RFX5	4.92	5.02	27.1	4	1.286
sp|Q15291|RBBP5_HUMAN	RBBP5	Retinoblastoma-binding protein 5	27.66	29.64	48.5	17	1.286
sp|Q9H2P9|DPH5_HUMAN	DPH5	Diphthine methyl ester synthase	20.05	20.05	57.5	11	1.286
sp|P22090|RS4Y1_HUMAN	RPS4Y1	40S ribosomal protein S4, Y isoform 1	15.44	40.47	72.6	35	1.287
sp|Q96HP0|DOCK6_HUMAN	DOCK6	Dedicator of cytokinesis protein 6	8.49	13.24	14.3	9	1.287
sp|Q9BZD4|NUF2_HUMAN	NUF2	Kinetochore protein Nuf2	14.24	15.67	43.8	9	1.287
sp|P23141|EST1_HUMAN	CES1	Liver carboxylesterase 1	43.09	43.29	72.1	29	1.288
sp|Q8NI37|PPTC7_HUMAN	PPTC7	Protein phosphatase PTC7 homolog	4.07	4.28	21.1	3	1.289
sp|Q5VVQ6|OTU1_HUMAN	YOD1	Ubiquitin thioesterase OTU1	9.55	9.63	37.1	6	1.289
sp|Q9BV10|ALG12_HUMAN	ALG12	Dol-P-Man:Man(7)GlcNAc(2)-PP-Dol alpha-1,6-mannosyltransferase	4.82	4.91	18.2	3	1.290
sp|P16219|ACADS_HUMAN	ACADS	Short-chain specific acyl-CoA dehydrogenase, mitochondrial	24.73	24.81	54.9	15	1.290
sp|Q71SY5|MED25_HUMAN	MED25	Mediator of RNA polymerase II transcription subunit 25	10.53	10.71	20.1	6	1.290
sp|O15020|SPTN2_HUMAN	SPTBN2	Spectrin beta chain, non-erythrocytic 2	46.13	67.33	35.6	40	1.290
sp|Q9NVM4|ANM7_HUMAN	PRMT7	Protein arginine N-methyltransferase 7	7.64	7.73	16.8	6	1.290
sp|O75110|ATP9A_HUMAN	ATP9A	Probable phospholipid-transporting ATPase IIA	6.91	7.33	16.1	7	1.290
sp|O60488|ACSL4_HUMAN	ACSL4	Long-chain-fatty-acid--CoA ligase 4	76.73	79.46	77.4	68	1.291
sp|O94919|ENDD1_HUMAN	ENDOD1	Endonuclease domain-containing 1 protein	6.4	6.45	29.6	4	1.291
sp|Q9UBX3|DIC_HUMAN	SLC25A10	Mitochondrial dicarboxylate carrier	27.65	27.96	65.9	24	1.291
sp|O76031|CLPX_HUMAN	CLPX	ATP-dependent Clp protease ATP-binding subunit clpX-like, mitochondrial	39.45	40.73	61	28	1.291
sp|Q96SW2|CRBN_HUMAN	CRBN	Protein cereblon	2.69	3.19	28.7	4	1.291
sp|Q9UPN7|PP6R1_HUMAN	PPP6R1	Serine/threonine-protein phosphatase 6 regulatory subunit 1	7.29	7.41	24	4	1.292
sp|Q9UGP4|LIMD1_HUMAN	LIMD1	LIM domain-containing protein 1	12.13	12.62	25.2	9	1.292
sp|Q9NWB6|ARGL1_HUMAN	ARGLU1	Arginine and glutamate-rich protein 1	4.61	9.13	38.8	7	1.292
sp|Q96SU4|OSBL9_HUMAN	OSBPL9	Oxysterol-binding protein-related protein 9	19.88	20.09	24.2	11	1.292
sp|Q9HCC0|MCCB_HUMAN	MCCC2	Methylcrotonoyl-CoA carboxylase beta chain, mitochondrial	48.91	48.95	75.8	39	1.293
sp|Q9Y365|PCTL_HUMAN	STARD10	PCTP-like protein	16.61	16.79	48.8	13	1.293
sp|Q9H490|PIGU_HUMAN	PIGU	Phosphatidylinositol glycan anchor biosynthesis class U protein	6.84	6.91	20.2	5	1.293

sp|Q9NRG9|AAAS_HUMAN	AAAS	Aladin	26.36	26.58	49.6	16	1.293
sp|P82979|SARNP_HUMAN	SARNP	SAP domain-containing ribonucleoprotein	13.4	14.27	59.5	9	1.294
sp|Q8IYQ7|THNS1_HUMAN	THNSL1	Threonine synthase-like 1	19.17	19.95	30.7	13	1.294
sp|Q9NRF8|PYRG2_HUMAN	CTPS2	CTP synthase 2	26.47	37.15	61.6	21	1.294
sp|Q8TB22|SPT20_HUMAN	SPATA20	Spermatogenesis-associated protein 20	11.1	11.43	20.9	9	1.294
sp|O60907|TBL1X_HUMAN	TBL1X	F-box-like/WD repeat-containing protein TBL1X	3.71	19.34	29.8	11	1.294
sp|Q8NBN7|RDH13_HUMAN	RDH13	Retinol dehydrogenase 13	9.87	10.21	48.6	5	1.295
sp|P00533|EGFR_HUMAN	EGFR	Epidermal growth factor receptor	8.65	11.52	22.1	8	1.295
sp|Q7Z7K0|COXM1_HUMAN	CMC1	COX assembly mitochondrial protein homolog	4.98	5.33	42.5	5	1.296
sp|P10909|CLUS_HUMAN	CLU	Clusterin	6.41	6.58	23.4	5	1.296
sp|Q9BTK6|PAGR1_HUMAN	PAGR1	PAXIP1-associated glutamate-rich protein 1	2	2.03	13.8	2	1.296
sp|Q5SVS4|KMCP1_HUMAN	SLC25A30	Kidney mitochondrial carrier protein 1	4.86	5.71	32.7	4	1.296
sp|Q9UKV8|AGO2_HUMAN	AGO2	Protein argonaute-2	26.71	27.44	36.2	15	1.297
sp|Q8TF05|PP4R1_HUMAN	PPP4R1	Serine/threonine-protein phosphatase 4 regulatory subunit 1	30.55	32.18	34.8	16	1.297
sp|Q8N8R5|CB069_HUMAN	C2orf69	UPF0565 protein C2orf69	4.08	4.09	15.1	2	1.298
sp|Q9Y4B6|VPRBP_HUMAN	VPRBP	Protein VPRBP	26.26	27.48	25	16	1.298
sp|Q14571|ITPR2_HUMAN	ITPR2	Inositol 1,4,5-trisphosphate receptor type 2	69.83	75.17	31.5	43	1.298
sp|P32189|GLPK_HUMAN	GK	Glycerol kinase	39.39	40.33	57.4	24	1.299
sp|Q9UPN3|MACF1_HUMAN	MACF1	Microtubule-actin cross-linking factor 1, isoforms 1/2/3/5	211.17	213.14	37.1	113	1.299
sp|Q86YS7|C2CD5_HUMAN	C2CD5	C2 domain-containing protein 5	12.94	13.13	23	7	1.299
sp|Q8NF37|PCAT1_HUMAN	LPCAT1	Lysophosphatidylcholine acyltransferase 1	10.31	11.03	31.5	6	1.299
sp|Q14573|ITPR3_HUMAN	ITPR3	Inositol 1,4,5-trisphosphate receptor type 3	32.24	46.72	25.8	25	1.299
sp|Q9P2D1|CHD7_HUMAN	CHD7	Chromodomain-helicase-DNA-binding protein 7	46.7	49.31	22.1	26	1.299
sp|Q9NTJ4|MA2C1_HUMAN	MAN2C1	Alpha-mannosidase 2C1	6.05	6.13	12	4	1.300
sp|Q92889|XPF_HUMAN	ERCC4	DNA repair endonuclease XPF	7.63	7.89	24.8	6	1.300
sp|P16083|NQO2_HUMAN	NQO2	Ribosyldihydronicotinamide dehydrogenase [quinone]	5.51	5.73	53.7	4	1.300
sp|Q9NW81|AT5SL_HUMAN	ATP5SL	ATP synthase subunit s-like protein	2.91	2.98	23	4	1.300
sp|P62256|UBE2H_HUMAN	UBE2H	Ubiquitin-conjugating enzyme E2 H	4.41	4.47	31.7	3	1.300
sp|Q9HBE1|PATZ1_HUMAN	PATZ1	POZ-, AT hook-, and zinc finger-containing protein 1	11.94	12.07	18.5	6	1.301
sp|Q8IV63|VRK3_HUMAN	VRK3	Inactive serine/threonine-protein kinase VRK3	4.09	4.11	18.8	2	1.301
sp|Q5VV41|ARHGG_HUMAN	ARHGEF16	Rho guanine nucleotide exchange factor 16	4.13	4.28	19.5	2	1.301
sp|O95905|ECD_HUMAN	ECD	Protein ecdysoneless homolog	10.54	10.62	26.2	6	1.301
sp|P51617|IRAK1_HUMAN	IRAK1	Interleukin-1 receptor-associated kinase 1	10.71	10.83	20.9	6	1.302
sp|Q9BSJ8|ESYT1_HUMAN	ESYT1	Extended synaptotagmin-1	63.6	65.62	46.7	47	1.302
sp|Q99961|SH3G1_HUMAN	SH3GL1	Endophilin-A2	22.74	24.89	64.4	21	1.302
sp|Q8WVT3|TPC12_HUMAN	TRAPPC12	Trafficking protein particle complex subunit 12	3.59	3.88	15.9	3	1.302
sp|P42126|ECI1_HUMAN	ECI1	Enoyl-CoA delta isomerase 1, mitochondrial	15.9	18.87	49	23	1.303
sp|P14621|ACYP2_HUMAN	ACYP2	Acylphosphatase-2	4.62	4.66	43.4	3	1.303
sp|Q13464|ROCK1_HUMAN	ROCK1	Rho-associated protein kinase 1	15.2	28.75	40	17	1.303
sp|Q86VR2|F134C_HUMAN	FAM134C	Protein FAM134C	10.41	10.69	26.2	10	1.304
sp|P40692|MLH1_HUMAN	MLH1	DNA mismatch repair protein Mlh1	21.3	21.55	38	11	1.304
sp|Q92786|PROX1_HUMAN	PROX1	Prospero homeobox protein 1	6.08	7.56	31.1	6	1.304
sp|P11532|DMD_HUMAN	DMD	Dystrophin	15.24	27.45	22.3	19	1.305
sp|P41229|KDM5C_HUMAN	KDM5C	Lysine-specific demethylase 5C	14.33	15.55	23.7	10	1.305
sp|P18433|PTPRA_HUMAN	PTPRA	Receptor-type tyrosine-protein phosphatase alpha	13.71	15.97	18.2	8	1.305
sp|Q5JSH3|WDR44_HUMAN	WDR44	WD repeat-containing protein 44	40.53	41.48	46.4	23	1.305
sp|Q96I15|SCLY_HUMAN	SCLY	Selenocysteine lyase	18.49	18.58	54.2	11	1.306
sp|Q9UKS6|PACN3_HUMAN	PACSIN3	Protein kinase C and casein kinase substrate in neurons protein 3	19.8	20.36	46.9	12	1.306
sp|Q2M1P5|KIF7_HUMAN	KIF7	Kinesin-like protein KIF7	7.73	13.19	21.6	8	1.306

sp|O60678|ANM3_HUMAN	PRMT3	Protein arginine N-methyltransferase 3	23.79	24.92	42.9	16	1.306
sp|Q49A26|GLYR1_HUMAN	GLYR1	Putative oxidoreductase GLYR1	24.78	25.6	52.6	17	1.307
sp|P15848|ARSB_HUMAN	ARSB	Arylsulfatase B	11.83	11.92	25.9	8	1.307
sp|Q9Y4G6|TLN2_HUMAN	TLN2	Talin-2	16.26	51.02	28.7	37	1.307
sp|Q3KQU3|MA7D1_HUMAN	MAP7D1	MAP7 domain-containing protein 1	12.01	13.21	24.6	9	1.307
sp|Q9UBG0|MRC2_HUMAN	MRC2	C-type mannose receptor 2	4.29	4.35	7.2	2	1.307
sp|Q6AI12|ANR40_HUMAN	ANKRD40	Ankyrin repeat domain-containing protein 40	4.61	4.83	23.6	4	1.307
sp|Q9Y4F5|C170B_HUMAN	CEP170B	Centrosomal protein of 170 kDa protein B	2.16	3.59	12	2	1.308
sp|Q9H1K0|RBNS5_HUMAN	RBSN	Rabenosyn-5	2.73	3	21.6	4	1.308
sp|Q9Y3D9|RT23_HUMAN	MRPS23	28S ribosomal protein S23, mitochondrial	15.15	15.24	68.4	9	1.308
sp|Q99436|PSB7_HUMAN	PSMB7	Proteasome subunit beta type-7	19.45	19.8	59.9	21	1.308
sp|Q8TF71|MOT10_HUMAN	SLC16A10	Monocarboxylate transporter 10	1.87	2.05	13.6	2	1.308
sp|Q14315|FLNC_HUMAN	FLNC	Filamin-C	14.69	46.09	29.4	33	1.308
sp|Q9NYL9|TMOD3_HUMAN	TMOD3	Tropomodulin-3	20.93	21.17	59.4	15	1.309
sp|Q13308|PTK7_HUMAN	PTK7	Inactive tyrosine-protein kinase 7	57.73	57.82	53.6	34	1.309
sp|O95182|NDUA7_HUMAN	NDUFA7	NADH dehydrogenase [ubiquinone] 1 alpha subcomplex subunit 7	13.23	13.42	86.7	11	1.309
sp|Q9NRX4|PHP14_HUMAN	PHPT1	14 kDa phosphohistidine phosphatase	7.25	7.94	51.2	6	1.309
sp|O43822|CU002_HUMAN	C21orf2	Protein C21orf2	2	2.05	9	2	1.309
sp|Q14331|FRG1_HUMAN	FRG1	Protein FRG1	8.71	8.76	35.7	8	1.310
sp|O95456|PSMG1_HUMAN	PSMG1	Proteasome assembly chaperone 1	20.04	20.12	46.2	11	1.310
sp|P02751|FINC_HUMAN	FN1	Fibronectin	96.44	96.57	37.9	67	1.312
sp|O95758|PTBP3_HUMAN	PTBP3	Polypyrimidine tract-binding protein 3	10.67	20.09	44.9	21	1.312
sp|Q16611|BAK_HUMAN	BAK1	Bcl-2 homologous antagonist/killer	6	6	33.7	4	1.312
sp|Q9BW83|IFT27_HUMAN	IFT27	Intraflagellar transport protein 27 homolog	15.96	16.24	65.6	8	1.313
sp|P08236|BGLR_HUMAN	GUSB	Beta-glucuronidase	3.81	4.06	13.1	2	1.313
sp|P10301|RRAS_HUMAN	RRAS	Ras-related protein R-Ras	2.81	7.06	35.8	5	1.313
sp|Q9H2W6|RM46_HUMAN	MRPL46	39S ribosomal protein L46, mitochondrial	20.28	20.41	71	14	1.313
sp|Q9HCN4|GPN1_HUMAN	GPN1	GPN-loop GTPase 1	7.96	8.07	28.3	7	1.313
sp|Q96FN9|DTD2_HUMAN	DTD2	Probable D-tyrosyl-tRNA(Tyr) deacylase 2	2.8	3.02	41.7	2	1.314
sp|Q96RT7|GCP6_HUMAN	TUBGCP6	Gamma-tubulin complex component 6	6.94	7.35	14.3	6	1.314
sp|Q15582|BGH3_HUMAN	TGFBI	Transforming growth factor-beta-induced protein ig-h3	2.34	2.57	15.4	2	1.314
sp|Q13214|SEM3B_HUMAN	SEMA3B	Semaphorin-3B	2.88	3.28	16.8	3	1.315
sp|Q6UX07|DHR13_HUMAN	DHRS13	Dehydrogenase/reductase SDR family member 13	2.02	2.03	19.6	2	1.315
sp|P06756|ITAV_HUMAN	ITGAV	Integrin alpha-V	37.43	37.47	37.1	22	1.315
sp|Q63HQ0|AP1AR_HUMAN	AP1AR	AP-1 complex-associated regulatory protein	3	3.1	13.9	3	1.315
sp|Q02809|PLOD1_HUMAN	PLOD1	Procollagen-lysine,2-oxoglutarate 5-dioxygenase 1	50.52	52.3	59.3	31	1.315
sp|Q969S3|ZN622_HUMAN	ZNF622	Zinc finger protein 622	20.24	20.37	43.2	13	1.316
sp|Q9Y3C0|CCD53_HUMAN	CCDC53	WASH complex subunit CCDC53	6.26	8.43	40.2	5	1.317
sp|Q6UXN9|WDR82_HUMAN	WDR82	WD repeat-containing protein 82	17.3	18.43	56.6	13	1.317
sp|Q3B726|RPA43_HUMAN	TWISTNB	DNA-directed RNA polymerase I subunit RPA43	8.68	8.73	15.7	7	1.317
sp|Q9UBW7|ZMYM2_HUMAN	ZMYM2	Zinc finger MYM-type protein 2	14.02	14.7	15	9	1.317
sp|Q9Y3L5|RAP2C_HUMAN	RAP2C	Ras-related protein Rap-2c	8.4	20.02	78.7	14	1.317
sp|P82094|TMF1_HUMAN	TMF1	TATA element modulatory factor	42.53	43.13	46.1	24	1.318
sp|Q06203|PUR1_HUMAN	PPAT	Amidophosphoribosyltransferase	38	38.06	57.6	21	1.319
sp|Q8IWS0|PHF6_HUMAN	PHF6	PHD finger protein 6	19.92	20.08	47.1	10	1.319
sp|Q9H5Z1|DHX35_HUMAN	DHX35	Probable ATP-dependent RNA helicase DHX35	11.37	13.53	33.3	9	1.319
sp|P40189|IL6RB_HUMAN	IL6ST	Interleukin-6 receptor subunit beta	8.03	8.08	24.1	5	1.319
sp|Q92696|PGTA_HUMAN	RABGGTA	Geranylgeranyl transferase type-2 subunit alpha	31.1	33.47	50.8	23	1.320
sp|Q5VWQ8|DAB2P_HUMAN	DAB2IP	Disabled homolog 2-interacting protein	12.13	14.05	19.9	8	1.320

sp|Q9H8M9|EVA1A_HUMAN	EVA1A	Protein eva-1 homolog A	4	4	19.1	2	1.320
sp|Q96EP0|RNF31_HUMAN	RNF31	E3 ubiquitin-protein ligase RNF31	20.34	22.56	30.3	13	1.321
sp|Q9BUT1|BDH2_HUMAN	BDH2	3-hydroxybutyrate dehydrogenase type 2	10.05	12.15	37.1	7	1.321
sp|P02671|FIBA_HUMAN	FGA	Fibrinogen alpha chain	17.22	18.09	28.4	12	1.322
sp|O95819|M4K4_HUMAN	MAP4K4	Mitogen-activated protein kinase kinase kinase kinase 4	48.7	49.68	38.7	28	1.322
sp|Q13371|PHLP_HUMAN	PDCL	Phosducin-like protein	8.01	8.02	26.6	4	1.323
sp|Q14697|GANAB_HUMAN	GANAB	Neutral alpha-glucosidase AB	82.8	82.93	67.3	75	1.323
sp|Q14061|COX17_HUMAN	COX17	Cytochrome c oxidase copper chaperone	3.98	4.14	85.7	4	1.323
sp|Q92888|ARHG1_HUMAN	ARHGEF1	Rho guanine nucleotide exchange factor 1	51.53	53.13	51.6	31	1.323
sp|Q9H2D6|TARA_HUMAN	TRIOBP	TRIO and F-actin-binding protein	8.88	9.62	13.5	8	1.324
sp|Q9Y285|SYFA_HUMAN	FARSA	Phenylalanine--tRNA ligase alpha subunit	35.33	35.89	53.4	28	1.324
sp|Q9Y2B0|CNPY2_HUMAN	CNPY2	Protein canopy homolog 2	16.41	16.48	57.7	14	1.324
sp|P35612|ADDB_HUMAN	ADD2	Beta-adducin	15.71	18.72	35.4	11	1.324
sp|O14976|GAK_HUMAN	GAK	Cyclin-G-associated kinase	38.02	40.35	34.3	22	1.325
sp|Q9Y5U4|INSI2_HUMAN	INSIG2	Insulin-induced gene 2 protein	3.88	4	11.6	2	1.325
sp|Q96P16|RPR1A_HUMAN	RPRD1A	Regulation of nuclear pre-mRNA domain-containing protein 1A	10.72	21.17	55.1	12	1.326
sp|P98160|PGBM_HUMAN	HSPG2	Basement membrane-specific heparan sulfate proteoglycan core protein	4.89	5.02	5.9	4	1.326
sp|Q9NRC1|ST7_HUMAN	ST7	Suppressor of tumorigenicity 7 protein	2.01	2.36	22.4	3	1.326
sp|P37173|TGFR2_HUMAN	TGFBR2	TGF-beta receptor type-2	12.75	12.93	25.4	6	1.327
sp|P10586|PTPRF_HUMAN	PTPRF	Receptor-type tyrosine-protein phosphatase F	79.01	82.06	42.8	44	1.327
sp|Q8NEN9|PDZD8_HUMAN	PDZD8	PDZ domain-containing protein 8	4.29	4.71	18.9	4	1.327
sp|P52735|VAV2_HUMAN	VAV2	Guanine nucleotide exchange factor VAV2	20.8	21.55	33.1	15	1.328
sp|Q5H8A4|PIGG_HUMAN	PIGG	GPI ethanolamine phosphate transferase 2	10.2	10.35	15.9	7	1.328
sp|Q16222|UAP1_HUMAN	UAP1	UDP-N-acetylhexosamine pyrophosphorylase	26.59	28.03	47.9	16	1.329
sp|Q9Y3D8|KAD6_HUMAN	AK6	Adenylate kinase isoenzyme 6	10.62	10.67	55.2	6	1.329
sp|P13716|HEM2_HUMAN	ALAD	Delta-aminolevulinic acid dehydratase	10.5	10.67	42.7	7	1.329
sp|Q96KP4|CNDP2_HUMAN	CNDP2	Cytosolic non-specific dipeptidase	47.95	48.08	68.8	33	1.330
sp|Q9BRA0|LSMD1_HUMAN	NAA38	N-alpha-acetyltransferase 38, NatC auxiliary subunit	3.74	3.9	66.4	5	1.330
sp|Q9NRZ5|PLCD_HUMAN	AGPAT4	1-acyl-sn-glycerol-3-phosphate acyltransferase delta	4.24	4.82	19.8	5	1.330
sp|Q5UCC4|EMC10_HUMAN	EMC10	ER membrane protein complex subunit 10	5.78	5.9	33.6	5	1.330
sp|Q9UBQ5|EIF3K_HUMAN	EIF3K	Eukaryotic translation initiation factor 3 subunit K	15.35	15.42	54.1	14	1.331
sp|Q9ULP9|TBC24_HUMAN	TBC1D24	TBC1 domain family member 24	10.6	11.87	29.3	9	1.331
sp|Q9Y2S0|RPAC2_HUMAN	POLR1D	DNA-directed RNA polymerases I and III subunit RPAC2	9.45	9.71	69.9	7	1.331
sp|P15144|AMPN_HUMAN	ANPEP	Aminopeptidase N	47.02	47.54	45.2	37	1.331
sp|Q9NX74|DUS2L_HUMAN	DUS2	tRNA-dihydrouridine(20) synthase [NAD(P)+]-like	7.65	9.17	36.7	6	1.332
sp|Q9P0U1|TOM7_HUMAN	TOMM7	Mitochondrial import receptor subunit TOM7 homolog	4.01	5.34	47.3	4	1.332
sp|P60520|GBRL2_HUMAN	GABARAPL2	Gamma-aminobutyric acid receptor-associated protein-like 2	14.66	14.76	66.7	8	1.333
sp|Q01658|NC2B_HUMAN	DR1	Protein Dr1	8.53	8.64	49.4	7	1.333
sp|O75150|BRE1B_HUMAN	RNF40	E3 ubiquitin-protein ligase BRE1B	27.27	31.04	47.3	16	1.334
sp|Q9BZI7|REN3B_HUMAN	UPF3B	Regulator of nonsense transcripts 3B	11.42	12.43	39.1	9	1.334
sp|Q92620|PRP16_HUMAN	DHX38	Pre-mRNA-splicing factor ATP-dependent RNA helicase PRP16	43.29	44.28	31	24	1.335
sp|Q5T447|HECD3_HUMAN	HECTD3	E3 ubiquitin-protein ligase HECTD3	10.62	11.65	26.4	9	1.335
sp|Q14012|KCC1A_HUMAN	CAMK1	Calcium/calmodulin-dependent protein kinase type 1	7.13	7.31	39.5	5	1.335
sp|P50453|SPB9_HUMAN	SERPINB9	Serpin B9	15.54	18.32	46.3	10	1.336
sp|P56545|CTBP2_HUMAN	CTBP2	C-terminal-binding protein 2	8.87	17.44	50.6	13	1.337
sp|Q01970|PLCB3_HUMAN	PLCB3	1-phosphatidylinositol 4,5-bisphosphate phosphodiesterase beta-3	27.44	29.92	36.3	21	1.337
sp|P15529|MCP_HUMAN	CD46	Membrane cofactor protein	3.65	3.76	19.6	2	1.338
sp|Q9NXU5|ARL15_HUMAN	ARL15	ADP-ribosylation factor-like protein 15	3.23	3.32	22.1	3	1.338
sp|Q96CS3|FAF2_HUMAN	FAF2	FAS-associated factor 2	31.74	31.82	75.3	29	1.339

sp|Q9HCD5|NCOA5_HUMAN	NCOA5	Nuclear receptor coactivator 5	22.7	22.88	44.6	15	1.340
sp|Q5SYE7|NHSL1_HUMAN	NHSL1	NHS-like protein 1	4.02	4.11	8.4	2	1.341
sp|Q8WUA4|TF3C2_HUMAN	GTF3C2	General transcription factor 3C polypeptide 2	27.56	27.88	33.6	16	1.341
sp|P49759|CLK1_HUMAN	CLK1	Dual specificity protein kinase CLK1	2	4.01	12	3	1.342
sp|O94832|MYO1D_HUMAN	MYO1D	Unconventional myosin-Id	48.85	51.12	51.5	32	1.342
sp|O95168|NDUB4_HUMAN	NDUFB4	NADH dehydrogenase [ubiquinone] 1 beta subcomplex subunit 4	9.93	9.97	50.4	6	1.343
sp|Q9BZG1|RAB34_HUMAN	RAB34	Ras-related protein Rab-34	2.11	4.57	30.5	6	1.344
sp|Q86T82|UBP37_HUMAN	USP37	Ubiquitin carboxyl-terminal hydrolase 37	2	2.88	11.5	4	1.344
sp|O95777|LSM8_HUMAN	LSM8	U6 snRNA-associated Sm-like protein LSm8	11.89	12.01	100	17	1.345
sp|Q7Z4H7|HAUS6_HUMAN	HAUS6	HAUS augmin-like complex subunit 6	10.19	11.61	31.5	7	1.345
sp|Q9BV40|VAMP8_HUMAN	VAMP8	Vesicle-associated membrane protein 8	3.28	3.37	45	2	1.346
sp|Q8IUC8|GLT13_HUMAN	GALNT13	Polypeptide N-acetylgalactosaminyltransferase 13	2	2.53	13.1	2	1.347
sp|Q8IWC1|MA7D3_HUMAN	MAP7D3	MAP7 domain-containing protein 3	3.49	5.53	23.5	6	1.347
sp|Q86VS8|HOOK3_HUMAN	HOOK3	Protein Hook homolog 3	26.85	28.14	45.1	18	1.348
sp|Q7Z2W4|ZCCHV_HUMAN	ZC3HAV1	Zinc finger CCCH-type antiviral protein 1	61.01	61.47	52.7	37	1.348
sp|O14841|OPLA_HUMAN	OPLAH	5-oxoprolinase	25.72	25.9	28	13	1.349
sp|Q86YS6|RAB43_HUMAN	RAB43	Ras-related protein Rab-43	5.93	8.57	61.8	6	1.349
sp|O75616|ERAL1_HUMAN	ERAL1	GTPase Era, mitochondrial	25.61	28.22	54.5	15	1.349
sp|P02748|CO9_HUMAN	C9	Complement component C9	5.03	5.88	22.4	7	1.349
sp|Q9Y256|FACE2_HUMAN	RCE1	CAAX prenyl protease 2	4	4	15.5	2	1.349
sp|Q8IY67|RAVR1_HUMAN	RAVER1	Ribonucleoprotein PTB-binding 1	27.92	28	47.7	19	1.351
sp|P01024|CO3_HUMAN	C3	Complement C3	100.64	102.61	54.4	61	1.351
sp|Q9NS86|LANC2_HUMAN	LANCL2	LanC-like protein 2	10.22	11.78	31.1	7	1.351
sp|O60925|PFD1_HUMAN	PFDN1	Prefoldin subunit 1	12.06	12.36	76.2	10	1.351
sp|Q14CZ7|FAKD3_HUMAN	FASTKD3	FAST kinase domain-containing protein 3	5.43	5.53	24	3	1.352
sp|Q9NUV7|SPTC3_HUMAN	SPTLC3	Serine palmitoyltransferase 3	3.55	3.68	18.5	3	1.352
sp|Q14CX7|NAA25_HUMAN	NAA25	N-alpha-acetyltransferase 25, NatB auxiliary subunit	38.89	45.11	44.6	28	1.353
sp|P52758|UK114_HUMAN	HRSP12	Ribonuclease UK114	15.78	15.9	61.3	10	1.353
sp|P29317|EPHA2_HUMAN	EPHA2	Ephrin type-A receptor 2	42.46	42.56	41.9	24	1.353
sp|Q9NVU0|RPC5_HUMAN	POLR3E	DNA-directed RNA polymerase III subunit RPC5	23.49	23.77	38.4	13	1.353
sp|Q9H0E2|TOLIP_HUMAN	TOLLIP	Toll-interacting protein	13.12	13.23	47.1	13	1.354
sp|Q9H7P6|MB12B_HUMAN	MVB12B	Multivesicular body subunit 12B	6.4	6.51	29.5	4	1.354
sp|Q9NXE8|CWC25_HUMAN	CWC25	Pre-mRNA-splicing factor CWC25 homolog	1.66	1.88	25.9	3	1.354
sp|Q6P1K2|PMF1_HUMAN	PMF1	Polyamine-modulated factor 1	4.01	4.09	24.4	2	1.355
sp|Q96RD7|PANX1_HUMAN	PANX1	Pannexin-1	3.31	3.63	26.3	2	1.356
sp|Q13576|IQGA2_HUMAN	IQGAP2	Ras GTPase-activating-like protein IQGAP2	98.16	112.37	59.2	77	1.356
sp|Q9UNS2|CSN3_HUMAN	COPS3	COP9 signalosome complex subunit 3	27.42	27.99	68.1	21	1.356
sp|Q9H0E9|BRD8_HUMAN	BRD8	Bromodomain-containing protein 8	12.58	12.9	20.9	7	1.356
sp|Q9NQX3|GEPH_HUMAN	GPHN	Gephyrin	39.56	43.34	52.6	23	1.357
sp|Q6NZI2|PTRF_HUMAN	PTRF	Polymerase I and transcript release factor	4	4.02	30.3	4	1.357
sp|Q9HAD4|WDR41_HUMAN	WDR41	WD repeat-containing protein 41	14.25	14.55	38.6	12	1.357
sp|Q93099|HGD_HUMAN	HGD	Homogentisate 1,2-dioxygenase	26.65	27.08	59.3	17	1.358
sp|P00441|SODC_HUMAN	SOD1	Superoxide dismutase [Cu-Zn]	24.97	25.09	90.9	58	1.359
sp|P78362|SRPK2_HUMAN	SRPK2	SRSF protein kinase 2	5.78	11.38	28.5	8	1.359
sp|Q6PJI9|WDR59_HUMAN	WDR59	WD repeat-containing protein 59	7.71	7.84	16.8	4	1.359
sp|P50579|MAP2_HUMAN	METAP2	Methionine aminopeptidase 2	32.59	34.75	61.9	23	1.359
sp|Q9C0D9|EPT1_HUMAN	EPT1	Ethanolaminephosphotransferase 1	3.48	3.58	10.3	4	1.361
sp|Q9NQX7|ITM2C_HUMAN	ITM2C	Integral membrane protein 2C	8.02	8.07	33.3	4	1.361
sp|Q9BW92|SYTM_HUMAN	TARS2	Threonine--tRNA ligase, mitochondrial	40.39	40.72	47.2	23	1.361

sp|P04040|CATA_HUMAN	CAT	Catalase	45.25	45.28	57.9	33	1.361
sp|Q14558|KPRA_HUMAN	PRPSAP1	Phosphoribosyl pyrophosphate synthase-associated protein 1	21.72	27.34	63.8	16	1.362
sp|P78318|IGBP1_HUMAN	IGBP1	Immunoglobulin-binding protein 1	14.58	14.73	48.1	10	1.362
sp|Q9NZB2|F120A_HUMAN	FAM120A	Constitutive coactivator of PPAR-gamma-like protein 1	46.22	46.35	39.3	26	1.363
sp|P17568|NDUB7_HUMAN	NDUFB7	NADH dehydrogenase [ubiquinone] 1 beta subcomplex subunit 7	8.02	8.19	49.6	5	1.363
sp|Q4ZIN3|MBRL_HUMAN	TMEM259	Membralin	5.81	5.9	21.5	7	1.364
sp|Q12962|TAF10_HUMAN	TAF10	Transcription initiation factor TFIID subunit 10	5.65	5.77	34.4	4	1.364
sp|O75674|TM1L1_HUMAN	TOM1L1	TOM1-like protein 1	3.13	5.36	19.1	5	1.364
sp|Q9NWX6|THG1_HUMAN	THG1L	Probable tRNA(His) guanylyltransferase	5.62	6.34	23.8	4	1.364
sp|Q6ZMZ3|SYNE3_HUMAN	SYNE3	Nesprin-3	5.7	5.99	19.8	4	1.364
sp|P02545|LMNA_HUMAN	LMNA	Prelamin-A/C	74.04	78.67	72.3	73	1.365
sp|Q15650|TRIP4_HUMAN	TRIP4	Activating signal cointegrator 1	14.82	15.22	37.9	10	1.365
sp|Q9Y5K5|UCHL5_HUMAN	UCHL5	Ubiquitin carboxyl-terminal hydrolase isozyme L5	29.63	29.76	62.6	20	1.365
sp|Q16706|MA2A1_HUMAN	MAN2A1	Alpha-mannosidase 2	46.12	49.96	44.3	29	1.366
sp|Q15910|EZH2_HUMAN	EZH2	Histone-lysine N-methyltransferase EZH2	15.2	15.56	31.1	12	1.366
sp|P40938|RFC3_HUMAN	RFC3	Replication factor C subunit 3	23.41	27.21	72.2	18	1.366
sp|O60231|DHX16_HUMAN	DHX16	Putative pre-mRNA-splicing factor ATP-dependent RNA helicase DHX16	43.84	46.95	41.5	27	1.366
sp|Q6P2D0|ZFP1_HUMAN	ZFP1	Zinc finger protein 1 homolog	2.01	4.11	17.9	3	1.367
sp|Q7Z434|MAVS_HUMAN	MAVS	Mitochondrial antiviral-signaling protein	18.76	18.83	42.2	13	1.367
sp|Q9P2K3|RCOR3_HUMAN	RCOR3	REST corepressor 3	7.49	15.89	36.8	11	1.367
sp|P48668|K2C6C_HUMAN	KRT6C	Keratin, type II cytoskeletal 6C	4.18	33.73	47	28	1.367
sp|Q7Z7F0|K0907_HUMAN	KIAA0907	UPF0469 protein KIAA0907	3.59	3.78	16.5	3	1.367
sp|O75147|OBSL1_HUMAN	OBSL1	Obscurin-like protein 1	20.98	21.42	19	12	1.367
sp|Q8ND76|CCNY_HUMAN	CCNY	Cyclin-Y	2.05	2.06	22.6	2	1.367
sp|Q9P0S3|ORML1_HUMAN	ORMDL1	ORM1-like protein 1	6.82	6.87	36.6	4	1.368
sp|Q9H7F0|AT133_HUMAN	ATP13A3	Probable cation-transporting ATPase 13A3	4.06	4.11	14.8	2	1.368
sp|Q8N6R0|MET13_HUMAN	METTL13	Methyltransferase-like protein 13	25.05	25.64	46.6	14	1.369
sp|O75436|VP26A_HUMAN	VPS26A	Vacuolar protein sorting-associated protein 26A	25.03	25.2	60.2	12	1.369
sp|Q9UL25|RAB21_HUMAN	RAB21	Ras-related protein Rab-21	14.49	17.83	67.1	12	1.369
sp|O15037|KHNYN_HUMAN	KHNYN	Protein KHNYN	2.01	4.02	14.9	2	1.370
sp|Q9UM00|TMCO1_HUMAN	TMCO1	Transmembrane and coiled-coil domain-containing protein 1	6.46	6.62	44.2	6	1.370
sp|O75817|POP7_HUMAN	POP7	Ribonuclease P protein subunit p20	2.68	2.73	31.4	2	1.371
sp|Q5TA45|INT11_HUMAN	CPSF3L	Integrator complex subunit 11	12.56	12.91	35.5	8	1.372
sp|Q9H2Y7|ZN106_HUMAN	ZNF106	Zinc finger protein 106	6.76	7.14	11.4	5	1.372
sp|Q9P0J0|NDUAD_HUMAN	NDUFA13	NADH dehydrogenase [ubiquinone] 1 alpha subcomplex subunit 13	19.58	19.68	71.5	11	1.373
sp|Q9NYM9|BET1L_HUMAN	BET1L	BET1-like protein	4	4.01	32.4	2	1.374
sp|P09104|ENOG_HUMAN	ENO2	Gamma-enolase	24.03	36.78	74.4	84	1.375
sp|Q9NRY4|RHG35_HUMAN	ARHGAP35	Rho GTPase-activating protein 35	33.88	35.33	30	20	1.375
sp|Q9P0V3|SH3B4_HUMAN	SH3BP4	SH3 domain-binding protein 4	2.2	2.48	19.9	2	1.376
sp|P14543|NID1_HUMAN	NID1	Nidogen-1	7.48	7.62	13.4	5	1.376
sp|Q9Y4X5|ARI1_HUMAN	ARIH1	E3 ubiquitin-protein ligase ARIH1	18.63	18.9	47.8	11	1.377
sp|Q9Y547|IFT25_HUMAN	HSPB11	Intraflagellar transport protein 25 homolog	7.04	7.1	38.2	4	1.378
sp|Q6PHR2|ULK3_HUMAN	ULK3	Serine/threonine-protein kinase ULK3	2.33	2.67	31.8	3	1.379
sp|Q15390|MTFR1_HUMAN	MTFR1	Mitochondrial fission regulator 1	2.79	2.9	28.2	5	1.380
sp|Q9UK76|HN1_HUMAN	HN1	Hematological and neurological expressed 1 protein	9.76	9.92	79.2	9	1.380
sp|Q7L5Y1|ENOF1_HUMAN	ENOSF1	Mitochondrial enolase superfamily member 1	5.25	5.38	19.4	3	1.380
sp|O15511|ARPC5_HUMAN	ARPC5	Actin-related protein 2/3 complex subunit 5	14.67	16.56	85.4	14	1.380
sp|O95372|LYPA2_HUMAN	LYPLA2	Acyl-protein thioesterase 2	13.33	13.9	67.5	12	1.381
sp|P48163|MAOX_HUMAN	ME1	NADP-dependent malic enzyme	8.04	8.17	28.5	5	1.381

sp|Q15276|RABE1_HUMAN	RABEP1	Rab GTPase-binding effector protein 1	24.65	29.44	46.9	18	1.381
sp|Q8IWB1|IPRI_HUMAN	ITPRIP	Inositol 1,4,5-trisphosphate receptor-interacting protein	1.66	2.13	20.1	3	1.383
sp|Q96J02|ITCH_HUMAN	ITCH	E3 ubiquitin-protein ligase Itchy homolog	39.6	39.69	38	33	1.383
sp|Q969M7|UBE2F_HUMAN	UBE2F	NEDD8-conjugating enzyme UBE2F	3.38	3.53	28.7	3	1.383
sp|Q8NBU5|ATAD1_HUMAN	ATAD1	ATPase family AAA domain-containing protein 1	8.72	11.56	46	7	1.383
sp|Q9P035|HACD3_HUMAN	HACD3	Very-long-chain (3R)-3-hydroxyacyl-CoA dehydratase 3	22.94	23.06	38.4	28	1.384
sp|Q9H900|ZWILC_HUMAN	ZWILCH	Protein zwilch homolog	11.72	11.92	25.9	6	1.384
sp|Q8N6T3|ARFG1_HUMAN	ARFGAP1	ADP-ribosylation factor GTPase-activating protein 1	32.58	33.67	63.6	20	1.384
sp|Q5VTR2|BRE1A_HUMAN	RNF20	E3 ubiquitin-protein ligase BRE1A	37.19	39.87	51	24	1.385
sp|Q03001|DYST_HUMAN	DST	Dystonin	21.49	48.75	21.6	31	1.386
sp|Q5T5X7|BEND3_HUMAN	BEND3	BEN domain-containing protein 3	15.49	16	31	14	1.386
sp|Q13136|LIPA1_HUMAN	PPFIA1	Liprin-alpha-1	35.81	40.89	38.8	22	1.386
sp|P10109|ADX_HUMAN	FDX1	Adrenodoxin, mitochondrial	6.87	7.1	23.4	4	1.386
sp|Q9NWL6|ASND1_HUMAN	ASNSD1	Asparagine synthetase domain-containing protein 1	2.59	2.64	19.1	2	1.386
sp|O60437|PEPL_HUMAN	PPL	Periplakin	2.45	4.87	31.4	5	1.387
sp|P50750|CDK9_HUMAN	CDK9	Cyclin-dependent kinase 9	8.27	11.61	40.3	9	1.387
sp|Q9Y315|DEOC_HUMAN	DERA	Deoxyribose-phosphate aldolase	20.61	20.86	62	11	1.387
sp|Q96L92|SNX27_HUMAN	SNX27	Sorting nexin-27	29.62	29.65	51.6	18	1.387
sp|P21291|CSRP1_HUMAN	CSRP1	Cysteine and glycine-rich protein 1	5.98	6.11	38.3	5	1.387
sp|Q3T8J9|GON4L_HUMAN	GON4L	GON-4-like protein	2.32	4.09	9.2	3	1.387
sp|Q9Y6N7|ROBO1_HUMAN	ROBO1	Roundabout homolog 1	16.79	16.89	17.2	8	1.387
sp|P23443|KS6B1_HUMAN	RPS6KB1	Ribosomal protein S6 kinase beta-1	7.07	9.3	25.3	6	1.388
sp|Q06278|AOXA_HUMAN	AOX1	Aldehyde oxidase	2.6	2.79	11.1	2	1.388
sp|O60763|USO1_HUMAN	USO1	General vesicular transport factor p115	57.54	61.55	58.8	44	1.389
sp|P50748|KNTC1_HUMAN	KNTC1	Kinetochore-associated protein 1	41.81	45.37	32.6	24	1.390
sp|Q9H6S0|YTDC2_HUMAN	YTHDC2	Probable ATP-dependent RNA helicase YTHDC2	51.19	52.49	47.5	34	1.390
sp|P15260|INGR1_HUMAN	IFNGR1	Interferon gamma receptor 1	5.02	5.18	16.4	3	1.390
sp|Q9H5X1|FA96A_HUMAN	FAM96A	MIP18 family protein FAM96A	5.83	5.9	34.4	4	1.391
sp|Q15126|PMVK_HUMAN	PMVK	Phosphomevalonate kinase	21.37	21.46	85.9	11	1.391
sp|O95714|HERC2_HUMAN	HERC2	E3 ubiquitin-protein ligase HERC2	17.91	21.4	15.4	15	1.391
sp|Q969Y2|GTPB3_HUMAN	GTPBP3	tRNA modification GTPase GTPBP3, mitochondrial	12.66	13.26	39.8	6	1.391
sp|P07902|GALT_HUMAN	GALT	Galactose-1-phosphate uridylyltransferase	2.02	2.03	26.7	2	1.393
sp|Q9UPT8|ZC3H4_HUMAN	ZC3H4	Zinc finger CCCH domain-containing protein 4	23.81	24.76	26.5	13	1.394
sp|O14530|TXND9_HUMAN	TXNDC9	Thioredoxin domain-containing protein 9	14.87	15.18	69	9	1.394
sp|Q12893|TM115_HUMAN	TMEM115	Transmembrane protein 115	8.55	8.85	34.2	6	1.395
sp|O94916|NFAT5_HUMAN	NFAT5	Nuclear factor of activated T-cells 5	5.69	5.79	7.1	4	1.395
sp|P15735|PHKG2_HUMAN	PHKG2	Phosphorylase b kinase gamma catalytic chain, liver/testis isoform	10.43	10.79	39.2	6	1.395
sp|Q8NHG7|SVIP_HUMAN	SVIP	Small VCP/p97-interacting protein	3.02	3.27	55.8	2	1.395
sp|Q9UKU7|ACAD8_HUMAN	ACAD8	Isobutyryl-CoA dehydrogenase, mitochondrial	11.85	12.96	39.8	9	1.395
sp|Q6P1M0|S27A4_HUMAN	SLC27A4	Long-chain fatty acid transport protein 4	35.34	36.33	51.2	20	1.396
sp|Q9NZT2|OGFR_HUMAN	OGFR	Opioid growth factor receptor	24.09	24.19	30.9	16	1.396
sp|Q4KMP7|TB10B_HUMAN	TBC1D10B	TBC1 domain family member 10B	23.12	23.21	37.3	17	1.397
sp|Q8N8A6|DDX51_HUMAN	DDX51	ATP-dependent RNA helicase DDX51	32.69	32.9	55.1	21	1.397
sp|Q8N766|EMC1_HUMAN	EMC1	ER membrane protein complex subunit 1	65.24	65.52	56.7	43	1.398
sp|Q99569|PKP4_HUMAN	PKP4	Plakophilin-4	18.09	22.01	27.2	13	1.398
sp|Q9Y2L5|TPPC8_HUMAN	TRAPPC8	Trafficking protein particle complex subunit 8	10.01	10.28	15.6	6	1.398
sp|P04792|HSPB1_HUMAN	HSPB1	Heat shock protein beta-1	18.34	18.36	76.1	13	1.398
sp|Q96HA1|P121A_HUMAN	POM121	Nuclear envelope pore membrane protein POM 121	15.85	16.16	18.3	10	1.399
sp|Q9HBR0|S38AA_HUMAN	SLC38A10	Putative sodium-coupled neutral amino acid transporter 10	8.94	9.16	20.6	8	1.399

sp|P13073|COX41_HUMAN	COX4I1	Cytochrome c oxidase subunit 4 isoform 1, mitochondrial	18.07	18.44	60.4	13	1.401
sp|Q9UBW8|CSN7A_HUMAN	COPS7A	COP9 signalosome complex subunit 7a	16.83	16.93	60	10	1.401
sp|Q86TM6|SYVN1_HUMAN	SYVN1	E3 ubiquitin-protein ligase synoviolin	6.62	10.1	18.6	6	1.401
sp|O95340|PAPS2_HUMAN	PAPSS2	Bifunctional 3'-phosphoadenosine 5'-phosphosulfate synthase 2	2.14	5.08	31.1	6	1.402
sp|Q86U28|ISCA2_HUMAN	ISCA2	Iron-sulfur cluster assembly 2 homolog, mitochondrial	12.04	12.23	61.7	7	1.402
sp|O94927|HAUS5_HUMAN	HAUS5	HAUS augmin-like complex subunit 5	15.11	15.94	30.2	9	1.403
sp|Q9NPJ6|MED4_HUMAN	MED4	Mediator of RNA polymerase II transcription subunit 4	10.02	10.15	57.8	7	1.403
sp|P34949|MPI_HUMAN	MPI	Mannose-6-phosphate isomerase	18.4	18.63	44.4	15	1.403
sp|Q8IYH5|ZZZ3_HUMAN	ZZZ3	ZZ-type zinc finger-containing protein 3	3.98	4.05	14.2	2	1.404
sp|Q96BD5|PF21A_HUMAN	PHF21A	PHD finger protein 21A	2.04	2.07	21.5	2	1.404
sp|O75223|GGCT_HUMAN	GGCT	Gamma-glutamylcyclotransferase	17.83	18.59	71.3	13	1.405
sp|P02533|K1C14_HUMAN	KRT14	Keratin, type I cytoskeletal 14	11.56	36.37	58.5	34	1.405
sp|O15258|RER1_HUMAN	RER1	Protein RER1	4	4	16.8	2	1.405
sp|Q5ST30|SYVM_HUMAN	VARS2	Valine--tRNA ligase, mitochondrial	38.04	38.9	40.8	20	1.406
sp|Q92908|GATA6_HUMAN	GATA6	Transcription factor GATA-6	4	4.01	8.7	2	1.406
sp|P42694|HELZ_HUMAN	HELZ	Probable helicase with zinc finger domain	14.94	16.15	19.6	9	1.407
sp|Q9BY12|SCAPE_HUMAN	SCAPER	S phase cyclin A-associated protein in the endoplasmic reticulum	2.09	2.25	12.9	3	1.407
sp|Q5T653|RM02_HUMAN	MRPL2	39S ribosomal protein L2, mitochondrial	15.85	16.01	45.9	14	1.407
sp|Q8NEW0|ZNT7_HUMAN	SLC30A7	Zinc transporter 7	4	4.04	16.5	5	1.408
sp|P50416|CPT1A_HUMAN	CPT1A	Carnitine O-palmitoyltransferase 1, liver isoform	29.25	29.59	31.8	15	1.408
sp|Q15572|TAF1C_HUMAN	TAF1C	TATA box-binding protein-associated factor RNA polymerase I subunit C	3.02	4.49	10.9	4	1.409
sp|Q8WVK2|SNR27_HUMAN	SNRNP27	U4/U6.U5 small nuclear ribonucleoprotein 27 kDa protein	4	4	21.9	3	1.410
sp|P07311|ACYP1_HUMAN	ACYP1	Acylphosphatase-1	5.75	5.87	61.6	4	1.411
sp|Q9Y3A5|SBDS_HUMAN	SBDS	Ribosome maturation protein SBDS	31.09	32.72	78.4	29	1.411
sp|Q9ULX6|AKP8L_HUMAN	AKAP8L	A-kinase anchor protein 8-like	24	24.12	37.6	15	1.411
sp|O95707|RPP29_HUMAN	POP4	Ribonuclease P protein subunit p29	5.56	5.73	40.5	3	1.412
sp|P49137|MAPK2_HUMAN	MAPKAPK2	MAP kinase-activated protein kinase 2	9.2	9.32	33.5	6	1.412
sp|Q6WKZ4|RFIP1_HUMAN	RAB11FIP1	Rab11 family-interacting protein 1	20.53	20.78	21.8	12	1.414
sp|Q9BRT3|MIEN1_HUMAN	MIEN1	Migration and invasion enhancer 1	10.64	10.74	72.2	6	1.415
sp|Q93062|RBPMS_HUMAN	RBPMS	RNA-binding protein with multiple splicing	7.12	7.52	45.4	5	1.416
sp|Q6P6C2|ALKB5_HUMAN	ALKBH5	RNA demethylase ALKBH5	11.59	12.26	45.7	7	1.416
sp|Q9BW19|KIFC1_HUMAN	KIFC1	Kinesin-like protein KIFC1	29.3	29.7	50.7	17	1.417
sp|O43824|GTPB6_HUMAN	GTPBP6	Putative GTP-binding protein 6	14.23	14.65	40.9	8	1.418
sp|P39880|CUX1_HUMAN	CUX1	Homeobox protein cut-like 1	38.05	39.71	33	22	1.419
sp|Q9ULD0|OGDHL_HUMAN	OGDHL	2-oxoglutarate dehydrogenase-like, mitochondrial	2.5	14.3	18.8	9	1.421
sp|P35250|RFC2_HUMAN	RFC2	Replication factor C subunit 2	27.43	28.35	67.8	19	1.421
sp|Q8WU79|SMAP2_HUMAN	SMAP2	Stromal membrane-associated protein 2	10.34	10.38	26.3	6	1.421
sp|P58107|EPIPL_HUMAN	EPPK1	Epiplakin	122.87	135.03	62.3	76	1.422
sp|Q96S94|CCNL2_HUMAN	CCNL2	Cyclin-L2	4.13	4.15	29.4	2	1.422
sp|Q00577|PURA_HUMAN	PURA	Transcriptional activator protein Pur-alpha	20.76	22.08	55.9	14	1.423
sp|P13591|NCAM1_HUMAN	NCAM1	Neural cell adhesion molecule 1	14.71	15.87	28.8	10	1.423
sp|P28074|PSB5_HUMAN	PSMB5	Proteasome subunit beta type-5	32.53	32.8	71.1	24	1.423
sp|Q9NRL3|STRN4_HUMAN	STRN4	Striatin-4	16.14	18.72	26.4	10	1.424
sp|P04062|GLCM_HUMAN	GBA	Glucosylceramidase	18.88	18.96	30.8	10	1.424
sp|O43567|RNF13_HUMAN	RNF13	E3 ubiquitin-protein ligase RNF13	2	2	22.6	3	1.425
sp|O43169|CYB5B_HUMAN	CYB5B	Cytochrome b5 type B	9.27	9.54	70.6	10	1.427
sp|Q96CB9|NSUN4_HUMAN	NSUN4	5-methylcytosine rRNA methyltransferase NSUN4	6.99	7.32	22.7	4	1.428
sp|P17676|CEBPB_HUMAN	CEBPB	CCAAT/enhancer-binding protein beta	3.89	3.96	12.8	2	1.430
sp|Q99829|CPNE1_HUMAN	CPNE1	Copine-1	29.84	32.23	48.8	21	1.430

sp|Q68CQ7|GL8D1_HUMAN	GLT8D1	Glycosyltransferase 8 domain-containing protein 1	9.98	10.2	32.9	5	1.431
sp|O00762|UBE2C_HUMAN	UBE2C	Ubiquitin-conjugating enzyme E2 C	8.1	8.48	53.6	6	1.432
sp|Q9NXV6|CARF_HUMAN	CDKN2AIP	CDKN2A-interacting protein	26.92	28.67	50.9	17	1.432
sp|O43156|TTI1_HUMAN	TTI1	TELO2-interacting protein 1 homolog	17.37	17.98	27.5	12	1.434
sp|Q969S9|RRF2M_HUMAN	GFM2	Ribosome-releasing factor 2, mitochondrial	28.12	28.28	43.5	18	1.434
sp|Q96CW5|GCP3_HUMAN	TUBGCP3	Gamma-tubulin complex component 3	22.7	22.9	32.4	12	1.434
sp|P55899|FCGRN_HUMAN	FCGRT	IgG receptor FcRn large subunit p51	7.97	8.09	32.3	6	1.434
sp|Q9HD20|AT131_HUMAN	ATP13A1	Manganese-transporting ATPase 13A1	60.98	61.37	44.4	42	1.435
sp|Q9P021|CRIPT_HUMAN	CRIPT	Cysteine-rich PDZ-binding protein	3.28	3.35	18.8	2	1.436
sp|P62875|RPAB5_HUMAN	POLR2L	DNA-directed RNA polymerases I, II, and III subunit RPABC5	10.17	11.1	83.6	7	1.438
sp|P27707|DCK_HUMAN	DCK	Deoxycytidine kinase	13.5	13.62	38.5	8	1.438
sp|Q75QN2|INT8_HUMAN	INTS8	Integrator complex subunit 8	4.5	5.17	24.2	7	1.439
sp|Q8NCD3|HJURP_HUMAN	HJURP	Holliday junction recognition protein	5.37	5.46	17.1	3	1.440
sp|Q9Y5Y2|NUBP2_HUMAN	NUBP2	Cytosolic Fe-S cluster assembly factor NUBP2	12.57	13.94	51.3	16	1.441
sp|P51812|KS6A3_HUMAN	RPS6KA3	Ribosomal protein S6 kinase alpha-3	66.2	66.95	71	51	1.442
sp|O43826|G6PT1_HUMAN	SLC37A4	Glucose-6-phosphate translocase	8.68	8.72	21.7	5	1.443
sp|Q5TCZ1|SPD2A_HUMAN	SH3PXD2A	SH3 and PX domain-containing protein 2A	4.06	5.9	20.5	3	1.444
sp|Q8IZV5|RDH10_HUMAN	RDH10	Retinol dehydrogenase 10	13.26	13.86	40.5	8	1.444
sp|Q9UMX1|SUFU_HUMAN	SUFU	Suppressor of fused homolog	3.96	6.04	22.5	4	1.445
sp|O43766|LIAS_HUMAN	LIAS	Lipoyl synthase, mitochondrial	2.31	2.43	17.5	2	1.445
sp|P45954|ACDSB_HUMAN	ACADSB	Short/branched chain specific acyl-CoA dehydrogenase, mitochondrial	38.08	38.55	71.5	35	1.445
sp|O75648|MTU1_HUMAN	TRMU	Mitochondrial tRNA-specific 2-thiouridylase 1	17.83	17.92	42.3	10	1.446
sp|P67936|TPM4_HUMAN	TPM4	Tropomyosin alpha-4 chain	42.6	47.04	77.8	41	1.446
sp|Q5PRF9|SMAG2_HUMAN	SAMD4B	Protein Smaug homolog 2	8.71	8.96	27.7	5	1.447
sp|Q9ULE6|PALD_HUMAN	PALD1	Paladin	3.98	4.1	15.2	3	1.448
sp|Q9NR50|EI2BG_HUMAN	EIF2B3	Translation initiation factor eIF-2B subunit gamma	20.65	21.53	56.6	16	1.448
sp|O43759|SNG1_HUMAN	SYNGR1	Synaptogyrin-1	2.81	2.87	11.6	2	1.449
sp|Q96GM5|SMRD1_HUMAN	SMARCD1	SWI/SNF-related matrix-associated actin-dependent regulator of chromatin subfamily D member 1	29.62	31.07	57.5	19	1.449
sp|Q58WW2|DCAF6_HUMAN	DCAF6	DDB1- and CUL4-associated factor 6	2.23	2.34	12.6	2	1.450
sp|Q9NS18|GLRX2_HUMAN	GLRX2	Glutaredoxin-2, mitochondrial	3.51	3.59	33.5	2	1.450
sp|O75116|ROCK2_HUMAN	ROCK2	Rho-associated protein kinase 2	107.3	109.28	59.3	58	1.451
sp|Q14011|CIRBP_HUMAN	CIRBP	Cold-inducible RNA-binding protein	10.08	10.93	54.1	12	1.451
sp|Q8IWI9|MGAP_HUMAN	MGA	MAX gene-associated protein	25.74	26.38	23.4	15	1.452
sp|Q9C004|SPY4_HUMAN	SPRY4	Protein sprouty homolog 4	7.51	7.67	36.8	4	1.452
sp|Q9HCE6|ARGAL_HUMAN	ARHGEF10L	Rho guanine nucleotide exchange factor 10-like protein	14.25	14.43	16.6	9	1.453
sp|Q9HD26|GOPC_HUMAN	GOPC	Golgi-associated PDZ and coiled-coil motif-containing protein	21.64	21.99	53.9	13	1.454
sp|P22670|RFX1_HUMAN	RFX1	MHC class II regulatory factor RFX1	9.92	10.08	17	6	1.454
sp|Q6UWE0|LRSM1_HUMAN	LRSAM1	E3 ubiquitin-protein ligase LRSAM1	13	13.16	30.7	7	1.454
sp|O43583|DENR_HUMAN	DENR	Density-regulated protein	12.9	13.6	75.8	6	1.454
sp|P46821|MAP1B_HUMAN	MAP1B	Microtubule-associated protein 1B	78.89	82.05	31.7	42	1.454
sp|Q9NQ94|A1CF_HUMAN	A1CF	APOBEC1 complementation factor	31.86	33.98	52.7	20	1.455
sp|P98170|XIAP_HUMAN	XIAP	E3 ubiquitin-protein ligase XIAP	13.51	13.65	32.2	7	1.455
sp|P98194|AT2C1_HUMAN	ATP2C1	Calcium-transporting ATPase type 2C member 1	26.57	27.11	41.5	26	1.455
sp|P15559|NQO1_HUMAN	NQO1	NAD(P)H dehydrogenase [quinone] 1	30.62	30.71	53.7	29	1.455
sp|Q9BST9|RTKN_HUMAN	RTKN	Rhotekin	12.12	12.98	31.8	10	1.456
sp|O75063|XYLK_HUMAN	FAM20B	Glycosaminoglycan xylosylkinase	7.41	7.65	28.6	5	1.458
sp|P53384|NUBP1_HUMAN	NUBP1	Cytosolic Fe-S cluster assembly factor NUBP1	16.12	16.38	49.1	8	1.458
sp|Q9NU22|MDN1_HUMAN	MDN1	Midasin	144	147.09	31.2	84	1.459
sp|Q8TE67|ES8L3_HUMAN	EPS8L3	Epidermal growth factor receptor kinase substrate 8-like protein 3	8.35	8.56	22.6	4	1.459

sp|Q96T60|PNKP_HUMAN	PNKP	Bifunctional polynucleotide phosphatase/kinase	20.85	21.13	45.3	13	1.459
sp|Q99519|NEUR1_HUMAN	NEU1	Sialidase-1	29.15	31.29	53	30	1.460
sp|A6QL63|BTBDB_HUMAN	BTBD11	Ankyrin repeat and BTB/POZ domain-containing protein BTBD11	2	2.22	11.6	4	1.461
sp|Q15014|MO4L2_HUMAN	MORF4L2	Mortality factor 4-like protein 2	16.05	16.08	44.4	8	1.461
sp|Q9BYM8|HOIL1_HUMAN	RBCK1	RanBP-type and C3HC4-type zinc finger-containing protein 1	9.62	9.92	35.9	6	1.461
sp|Q9NX14|NDUBB_HUMAN	NDUFB11	NADH dehydrogenase [ubiquinone] 1 beta subcomplex subunit 11, mitochondrial	8.52	8.58	41.8	6	1.461
sp|P05165|PCCA_HUMAN	PCCA	Propionyl-CoA carboxylase alpha chain, mitochondrial	37.92	40	45.2	22	1.461
sp|O95295|SNAPN_HUMAN	SNAPIN	SNARE-associated protein Snapin	12	12	72.1	9	1.462
sp|Q9H098|F107B_HUMAN	FAM107B	Protein FAM107B	6.1	6.35	60.3	6	1.463
sp|Q9H9E3|COG4_HUMAN	COG4	Conserved oligomeric Golgi complex subunit 4	22.51	22.79	38.3	12	1.463
sp|Q01433|AMPD2_HUMAN	AMPD2	AMP deaminase 2	28.19	29.96	36.5	16	1.463
sp|Q8WVM0|TFB1M_HUMAN	TFB1M	Dimethyladenosine transferase 1, mitochondrial	29.93	30.81	47.1	19	1.463
sp|Q9H3L0|MMAD_HUMAN	MMADHC	Methylmalonic aciduria and homocystinuria type D protein, mitochondrial	4	4	14.5	2	1.464
sp|Q9NR19|ACSA_HUMAN	ACSS2	Acetyl-coenzyme A synthetase, cytoplasmic	25.34	26.54	45.5	17	1.464
sp|Q9NZL9|MAT2B_HUMAN	MAT2B	Methionine adenosyltransferase 2 subunit beta	25.79	26.46	57.8	17	1.465
sp|P01892|1A02_HUMAN	HLA-A	HLA class I histocompatibility antigen, A-2 alpha chain	22.13	22.17	61.1	13	1.466
sp|Q96C57|CL043_HUMAN	C12orf43	Uncharacterized protein C12orf43	13.14	13.2	55.3	9	1.468
sp|Q9H6R3|ACSS3_HUMAN	ACSS3	Acyl-CoA synthetase short-chain family member 3, mitochondrial	44.41	44.47	55.5	26	1.468
sp|P28072|PSB6_HUMAN	PSMB6	Proteasome subunit beta type-6	20.73	20.78	68.6	22	1.469
sp|Q96E39|RMXL1_HUMAN	RBMXL1	RNA binding motif protein, X-linked-like-1	3.5	25.11	45.6	28	1.469
sp|Q9HA47|UCK1_HUMAN	UCK1	Uridine-cytidine kinase 1	2.01	3.78	17	3	1.469
sp|O15264|MK13_HUMAN	MAPK13	Mitogen-activated protein kinase 13	4.33	6.48	32.6	4	1.470
sp|Q9H270|VPS11_HUMAN	VPS11	Vacuolar protein sorting-associated protein 11 homolog	16.23	17.64	38.4	11	1.473
sp|Q14738|2A5D_HUMAN	PPP2R5D	Serine/threonine-protein phosphatase 2A 56 kDa regulatory subunit delta isoform	26.28	27.05	52	17	1.473
sp|O75581|LRP6_HUMAN	LRP6	Low-density lipoprotein receptor-related protein 6	9.84	10.34	14.8	7	1.473
sp|O95479|G6PE_HUMAN	H6PD	GDH/6PGL endoplasmic bifunctional protein	10.83	11.12	26.3	8	1.473
sp|Q9NZJ0|DTL_HUMAN	DTL	Denticleless protein homolog	7.64	7.8	13.2	4	1.474
sp|A6NCS6|CB072_HUMAN	C2orf72	Uncharacterized protein C2orf72	7.83	7.96	31.2	4	1.476
sp|O00268|TAF4_HUMAN	TAF4	Transcription initiation factor TFIID subunit 4	17.51	19.01	22.7	11	1.476
sp|Q8IZ83|A16A1_HUMAN	ALDH16A1	Aldehyde dehydrogenase family 16 member A1	26.46	26.53	42.8	20	1.476
sp|P16662|UD2B7_HUMAN	UGT2B7	UDP-glucuronosyltransferase 2B7	1.61	1.82	15.3	2	1.477
sp|Q96FF9|CDCA5_HUMAN	CDCA5	Sororin	6.42	6.57	52.4	4	1.478
sp|P61218|RPAB2_HUMAN	POLR2F	DNA-directed RNA polymerases I, II, and III subunit RPABC2	3.5	3.83	32.3	5	1.479
sp|Q9Y6X9|MORC2_HUMAN	MORC2	MORC family CW-type zinc finger protein 2	16.81	18.5	35.2	11	1.480
sp|Q7L266|ASGL1_HUMAN	ASRGL1	Isoaspartyl peptidase/L-asparaginase	13.8	13.94	53.9	9	1.481
sp|Q16656|NRF1_HUMAN	NRF1	Nuclear respiratory factor 1	5.01	5.07	11.1	3	1.482
sp|P52630|STAT2_HUMAN	STAT2	Signal transducer and activator of transcription 2	10.93	12.13	25.6	9	1.482
sp|Q13393|PLD1_HUMAN	PLD1	Phospholipase D1	27.5	28.47	34.3	14	1.482
sp|Q9H4I9|EMRE_HUMAN	SMDT1	Essential MCU regulator, mitochondrial	2.32	2.35	40.2	2	1.484
sp|Q4LE39|ARI4B_HUMAN	ARID4B	AT-rich interactive domain-containing protein 4B	17.27	17.57	22.6	11	1.484
sp|Q14696|MESD_HUMAN	MESDC2	LDLR chaperone MESD	22.82	22.93	69.2	18	1.485
sp|Q13153|PAK1_HUMAN	PAK1	Serine/threonine-protein kinase PAK 1	11.44	23.29	46.4	18	1.487
sp|Q9NZ45|CISD1_HUMAN	CISD1	CDGSH iron-sulfur domain-containing protein 1	2.77	2.83	39.8	2	1.487
sp|Q9Y3L3|3BP1_HUMAN	SH3BP1	SH3 domain-binding protein 1	15.59	16.62	31.7	10	1.488
sp|P13807|GYS1_HUMAN	GYS1	Glycogen [starch] synthase, muscle	25.58	25.76	35.1	15	1.488
sp|P05198|IF2A_HUMAN	EIF2S1	Eukaryotic translation initiation factor 2 subunit 1	40.49	40.72	69.8	33	1.489
sp|Q15437|SC23B_HUMAN	SEC23B	Protein transport protein Sec23B	30.22	40.28	51.6	24	1.490
sp|O75787|RENR_HUMAN	ATP6AP2	Renin receptor	14.04	14.26	51.1	11	1.490
sp|Q9H2H8|PPIL3_HUMAN	PPIL3	Peptidyl-prolyl cis-trans isomerase-like 3	13.54	15.39	80.1	13	1.490

sp|Q6P1N0|C2D1A_HUMAN	CC2D1A	Coiled-coil and C2 domain-containing protein 1A	39.26	39.42	43.1	21	1.490
sp|Q9Y2D4|EXC6B_HUMAN	EXOC6B	Exocyst complex component 6B	17.04	19.43	27.9	12	1.492
sp|Q5T440|CAF17_HUMAN	IBA57	Putative transferase CAF17, mitochondrial	23.22	23.28	56.5	14	1.493
sp|Q9NYS0|KBRS1_HUMAN	NKIRAS1	NF-kappa-B inhibitor-interacting Ras-like protein 1	2	2.01	12	2	1.493
sp|Q9BQE3|TBA1C_HUMAN	TUBA1C	Tubulin alpha-1C chain	10.1	86.39	92.7	303	1.494
sp|Q99439|CNN2_HUMAN	CNN2	Calponin-2	22.39	23.21	73.5	14	1.494
sp|Q5TDH0|DDI2_HUMAN	DDI2	Protein DDI1 homolog 2	26.14	26.53	54.9	13	1.494
sp|Q9UBB6|NCDN_HUMAN	NCDN	Neurochondrin	18.99	21.93	32.4	13	1.495
sp|Q12899|TRI26_HUMAN	TRIM26	Tripartite motif-containing protein 26	14.7	15.02	34.3	9	1.496
sp|Q9P2K5|MYEF2_HUMAN	MYEF2	Myelin expression factor 2	11.63	11.76	27.5	7	1.496
sp|Q92805|GOGA1_HUMAN	GOLGA1	Golgin subfamily A member 1	4.87	7.49	31.7	6	1.497
sp|Q9BR61|ACBD6_HUMAN	ACBD6	Acyl-CoA-binding domain-containing protein 6	5.93	6.15	18.8	3	1.497
sp|Q06210|GFPT1_HUMAN	GFPT1	Glutamine--fructose-6-phosphate aminotransferase [isomerizing] 1	59.3	59.47	67	44	1.499
sp|Q9UN86|G3BP2_HUMAN	G3BP2	Ras GTPase-activating protein-binding protein 2	23.9	27.75	45.6	21	1.499
sp|Q9NYJ8|TAB2_HUMAN	TAB2	TGF-beta-activated kinase 1 and MAP3K7-binding protein 2	3.19	3.53	14.6	4	1.500
sp|Q96MX6|WDR92_HUMAN	WDR92	WD repeat-containing protein 92	9.58	10.58	47.1	9	1.500
sp|P48723|HSP13_HUMAN	HSPA13	Heat shock 70 kDa protein 13	12.03	12.06	33.8	9	1.501
sp|Q13268|DHRS2_HUMAN	DHRS2	Dehydrogenase/reductase SDR family member 2, mitochondrial	32.07	32.5	86.1	58	1.503
sp|Q6P1R3|MSD2_HUMAN	MSANTD2	Myb/SANT-like DNA-binding domain-containing protein 2	3.64	3.75	16.8	3	1.503
sp|O75935|DCTN3_HUMAN	DCTN3	Dynactin subunit 3	9.14	9.55	54.3	9	1.504
sp|Q5T7V8|GORAB_HUMAN	GORAB	RAB6-interacting golgin	2.79	2.93	22.8	2	1.504
sp|O95989|NUDT3_HUMAN	NUDT3	Diphosphoinositol polyphosphate phosphohydrolase 1	9.66	10.41	55.2	7	1.505
sp|Q9UBP6|TRMB_HUMAN	METTL1	tRNA (guanine-N(7)-)-methyltransferase	14.71	14.88	50.4	8	1.505
sp|P08833|IBP1_HUMAN	IGFBP1	Insulin-like growth factor-binding protein 1	8.11	8.13	33.2	5	1.506
sp|P0CG39|POTEJ_HUMAN	POTEJ	POTE ankyrin domain family member J	1.72	21.11	30.6	50	1.506
sp|Q96KM6|Z512B_HUMAN	ZNF512B	Zinc finger protein 512B	8.19	8.28	26.2	5	1.507
sp|P32780|TF2H1_HUMAN	GTF2H1	General transcription factor IIH subunit 1	11.25	11.96	35.2	7	1.509
sp|Q96ER3|SAAL1_HUMAN	SAAL1	Protein SAAL1	14.03	14.1	29.8	7	1.509
sp|Q9H4W6|COE3_HUMAN	EBF3	Transcription factor COE3	5.23	5.41	17.5	3	1.512
sp|Q14997|PSME4_HUMAN	PSME4	Proteasome activator complex subunit 4	41.7	44.48	31	27	1.514
sp|Q96DU7|IP3KC_HUMAN	ITPKC	Inositol-trisphosphate 3-kinase C	2	2.03	12.2	2	1.514
sp|O95297|MPZL1_HUMAN	MPZL1	Myelin protein zero-like protein 1	5.87	5.99	23.1	3	1.515
sp|Q6IAN0|DRS7B_HUMAN	DHRS7B	Dehydrogenase/reductase SDR family member 7B	9.98	10.2	40.6	6	1.517
sp|A3KMH1|VWA8_HUMAN	VWA8	von Willebrand factor A domain-containing protein 8	74.7	77.91	43.6	41	1.518
sp|A8TX70|CO6A5_HUMAN	COL6A5	Collagen alpha-5(VI) chain	1.38	1.59	11.7	4	1.518
sp|P17858|PFKAL_HUMAN	PFKL	ATP-dependent 6-phosphofructokinase, liver type	50.07	50.14	62.2	46	1.519
sp|Q96TA1|NIBL1_HUMAN	FAM129B	Niban-like protein 1	28.66	28.9	45.6	16	1.520
sp|Q9BZK7|TBL1R_HUMAN	TBL1XR1	F-box-like/WD repeat-containing protein TBL1XR1	27.98	30.16	52.3	17	1.520
sp|Q9Y619|ORNT1_HUMAN	SLC25A15	Mitochondrial ornithine transporter 1	13.41	13.94	58.8	6	1.520
sp|Q99717|SMAD5_HUMAN	SMAD5	Mothers against decapentaplegic homolog 5	15.68	15.97	20.9	8	1.524
sp|P33908|MA1A1_HUMAN	MAN1A1	Mannosyl-oligosaccharide 1,2-alpha-mannosidase IA	27.56	28.12	36.5	18	1.525
sp|P52747|ZN143_HUMAN	ZNF143	Zinc finger protein 143	3.41	3.57	10.8	3	1.526
sp|Q96NC0|ZMAT2_HUMAN	ZMAT2	Zinc finger matrin-type protein 2	4.82	5.11	48.2	4	1.527
sp|P50336|PPOX_HUMAN	PPOX	Protoporphyrinogen oxidase	11.95	12.76	21.8	7	1.527
sp|P11169|GTR3_HUMAN	SLC2A3	Solute carrier family 2, facilitated glucose transporter member 3	12	14.02	16.9	11	1.527
sp|Q9H7H0|MET17_HUMAN	METTL17	Methyltransferase-like protein 17, mitochondrial	12.27	12.63	34.4	7	1.527
sp|P51687|SUOX_HUMAN	SUOX	Sulfite oxidase, mitochondrial	12.16	12.36	37.1	7	1.528
sp|Q5VTE6|ANGE2_HUMAN	ANGEL2	Protein angel homolog 2	6.49	6.56	22.8	5	1.529
sp|Q9P0V9|SEP10_HUMAN	SEPT10	Septin-10	15.12	17.7	43.2	15	1.529

sp|P31040|SDHA_HUMAN	SDHA	Succinate dehydrogenase [ubiquinone] flavoprotein subunit, mitochondrial	46.17	47.9	69.7	55	1.531
sp|Q8TAG9|EXOC6_HUMAN	EXOC6	Exocyst complex component 6	4.06	4.59	22.6	6	1.533
sp|P46063|RECQ1_HUMAN	RECQL	ATP-dependent DNA helicase Q1	43.63	43.92	55.8	26	1.534
sp|Q9BYN8|RT26_HUMAN	MRPS26	28S ribosomal protein S26, mitochondrial	16.18	16.3	52.7	9	1.534
sp|O75127|PTCD1_HUMAN	PTCD1	Pentatricopeptide repeat-containing protein 1, mitochondrial	23.19	23.27	35.9	14	1.534
sp|Q6YN16|HSDL2_HUMAN	HSDL2	Hydroxysteroid dehydrogenase-like protein 2	57.19	58.72	81.6	35	1.534
sp|Q9Y6B6|SAR1B_HUMAN	SAR1B	GTP-binding protein SAR1b	16	20.19	82.3	15	1.535
sp|Q8IY37|DHX37_HUMAN	DHX37	Probable ATP-dependent RNA helicase DHX37	49.82	50.17	51.9	30	1.535
sp|P13987|CD59_HUMAN	CD59	CD59 glycoprotein	7.81	7.91	39.1	6	1.535
sp|P09668|CATH_HUMAN	CTSH	Pro-cathepsin H	11.39	11.59	48.4	6	1.535
sp|O95140|MFN2_HUMAN	MFN2	Mitofusin-2	31.12	31.23	35.9	17	1.537
sp|Q8NC56|LEMD2_HUMAN	LEMD2	LEM domain-containing protein 2	20.69	22.8	46.9	11	1.537
sp|Q9H974|QTRD1_HUMAN	QTRTD1	Queuine tRNA-ribosyltransferase subunit QTRTD1	22.26	23.25	54.2	14	1.538
sp|Q6GMV2|SMYD5_HUMAN	SMYD5	SET and MYND domain-containing protein 5	5.74	7.96	37.1	8	1.540
sp|Q8NEU8|DP13B_HUMAN	APPL2	DCC-interacting protein 13-beta	5.62	6.32	24.1	4	1.541
sp|Q96LB3|IFT74_HUMAN	IFT74	Intraflagellar transport protein 74 homolog	5.69	6.29	31.2	5	1.541
sp|Q9UHN1|DPOG2_HUMAN	POLG2	DNA polymerase subunit gamma-2, mitochondrial	7.15	7.26	25.4	5	1.542
sp|Q9UPN4|CP131_HUMAN	CEP131	Centrosomal protein of 131 kDa	20.62	21	35.2	11	1.543
sp|Q9NVN3|RIC8B_HUMAN	RIC8B	Synembryn-B	2.05	4.25	24	6	1.546
sp|Q8NEB9|PK3C3_HUMAN	PIK3C3	Phosphatidylinositol 3-kinase catalytic subunit type 3	23.71	24.01	38.8	12	1.547
sp|Q5R372|RBG1L_HUMAN	RABGAP1L	Rab GTPase-activating protein 1-like	4.12	4.33	23.2	5	1.548
sp|Q86WR7|PRSR2_HUMAN	PROSER2	Proline and serine-rich protein 2	4.72	4.8	21.4	4	1.548
sp|Q8WUP2|FBLI1_HUMAN	FBLIM1	Filamin-binding LIM protein 1	5.38	5.5	27.6	3	1.549
sp|Q8IUR7|ARMC8_HUMAN	ARMC8	Armadillo repeat-containing protein 8	10.3	10.45	30.6	7	1.549
sp|P62310|LSM3_HUMAN	LSM3	U6 snRNA-associated Sm-like protein LSm3	5.17	8	75.5	7	1.550
sp|Q86Y82|STX12_HUMAN	STX12	Syntaxin-12	10.4	10.52	50.7	12	1.551
sp|Q96Q89|KI20B_HUMAN	KIF20B	Kinesin-like protein KIF20B	3.13	3.77	29	7	1.551
sp|Q8WTW3|COG1_HUMAN	COG1	Conserved oligomeric Golgi complex subunit 1	25.91	26.16	32.9	18	1.551
sp|P21359|NF1_HUMAN	NF1	Neurofibromin	12.65	13.25	19.2	11	1.552
sp|Q8ND24|RN214_HUMAN	RNF214	RING finger protein 214	18.1	22.33	42.4	14	1.553
sp|Q04828|AK1C1_HUMAN	AKR1C1	Aldo-keto reductase family 1 member C1	72.63	73.17	93.2	170	1.553
sp|Q9ULG1|INO80_HUMAN	INO80	DNA helicase INO80	5.32	6.54	16.9	5	1.553
sp|Q8N138|ORML3_HUMAN	ORMDL3	ORM1-like protein 3	2	4.79	26.8	3	1.555
sp|Q2NKX8|ERC6L_HUMAN	ERCC6L	DNA excision repair protein ERCC-6-like	25.32	25.79	31.8	17	1.557
sp|Q96CN9|GCC1_HUMAN	GCC1	GRIP and coiled-coil domain-containing protein 1	1.72	1.87	21.6	2	1.558
sp|Q8WUN7|UBTD2_HUMAN	UBTD2	Ubiquitin domain-containing protein 2	3.09	3.27	27.4	3	1.558
sp|Q9Y303|NAGA_HUMAN	AMDHD2	Putative N-acetylglucosamine-6-phosphate deacetylase	8.63	8.84	24.9	5	1.558
sp|Q8WV24|PHLA1_HUMAN	PHLDA1	Pleckstrin homology-like domain family A member 1	1.81	1.95	16.2	2	1.558
sp|Q92794|KAT6A_HUMAN	KAT6A	Histone acetyltransferase KAT6A	7.96	10.53	14.5	10	1.558
sp|P23025|XPA_HUMAN	XPA	DNA repair protein complementing XP-A cells	4.91	5.1	38.8	3	1.559
sp|O95983|MBD3_HUMAN	MBD3	Methyl-CpG-binding domain protein 3	21.53	21.77	62.2	17	1.561
sp|Q96B26|EXOS8_HUMAN	EXOSC8	Exosome complex component RRP43	16.32	16.34	53.3	9	1.562
sp|Q6PID6|TTC33_HUMAN	TTC33	Tetratricopeptide repeat protein 33	2.69	2.78	27.1	2	1.564
sp|Q8WY54|PPM1E_HUMAN	PPM1E	Protein phosphatase 1E	2	2.05	8.2	2	1.565
sp|Q8N3X1|FNBP4_HUMAN	FNBP4	Formin-binding protein 4	6.92	7.02	12.2	4	1.565
sp|Q9UL03|INT6_HUMAN	INTS6	Integrator complex subunit 6	8.29	8.36	25.5	4	1.566
sp|Q02318|CP27A_HUMAN	CYP27A1	Sterol 26-hydroxylase, mitochondrial	15.81	17.6	31.3	9	1.566
sp|Q9Y6R4|M3K4_HUMAN	MAP3K4	Mitogen-activated protein kinase kinase kinase 4	3.12	3.74	18.5	5	1.567
sp|Q86VW0|SESD1_HUMAN	SESTD1	SEC14 domain and spectrin repeat-containing protein 1	8.23	9.59	28	7	1.568

sp|O75891|AL1L1_HUMAN	ALDH1L1	Cytosolic 10-formyltetrahydrofolate dehydrogenase	27.33	35.94	49	19	1.569
sp|Q9P013|CWC15_HUMAN	CWC15	Spliceosome-associated protein CWC15 homolog	5.96	8.17	46.3	7	1.570
sp|Q9BU02|THTPA_HUMAN	THTPA	Thiamine-triphosphatase	7.53	7.61	53	4	1.571
sp|Q7Z2W9|RM21_HUMAN	MRPL21	39S ribosomal protein L21, mitochondrial	17.24	17.35	61	11	1.571
sp|Q14534|ERG1_HUMAN	SQLE	Squalene monooxygenase	25.6	26.12	50	17	1.572
sp|Q8TD22|SFXN5_HUMAN	SFXN5	Sideroflexin-5	3.11	3.25	25.6	3	1.574
sp|Q99755|PI51A_HUMAN	PIP5K1A	Phosphatidylinositol 4-phosphate 5-kinase type-1 alpha	7.09	7.31	19.4	5	1.576
sp|Q86TG7|PEG10_HUMAN	PEG10	Retrotransposon-derived protein PEG10	39.16	39.37	58.6	36	1.576
sp|Q96DT7|ZBT10_HUMAN	ZBTB10	Zinc finger and BTB domain-containing protein 10	8.14	8.28	21.6	4	1.577
sp|Q86XA9|HTR5A_HUMAN	HEATR5A	HEAT repeat-containing protein 5A	22.56	25.29	17.2	13	1.579
sp|P51956|NEK3_HUMAN	NEK3	Serine/threonine-protein kinase Nek3	8.73	9.01	34	6	1.579
sp|Q0VDG4|SCRN3_HUMAN	SCRN3	Secernin-3	8.75	8.99	34.4	6	1.579
sp|O75879|GATB_HUMAN	GATB	Glutamyl-tRNA(Gln) amidotransferase subunit B, mitochondrial	27.44	30.05	57.1	19	1.579
sp|Q9NUL7|DDX28_HUMAN	DDX28	Probable ATP-dependent RNA helicase DDX28	16.96	17.08	35.4	11	1.580
sp|Q5T3J3|LRIF1_HUMAN	LRIF1	Ligand-dependent nuclear receptor-interacting factor 1	2	4.06	15.3	4	1.581
sp|P22570|ADRO_HUMAN	FDXR	NADPH:adrenodoxin oxidoreductase, mitochondrial	57.93	58.03	79.4	39	1.582
sp|P51690|ARSE_HUMAN	ARSE	Arylsulfatase E	6.25	6.62	17.8	3	1.583
sp|Q9BW91|NUDT9_HUMAN	NUDT9	ADP-ribose pyrophosphatase, mitochondrial	8.69	8.9	41.7	5	1.584
sp|Q9NP58|ABCB6_HUMAN	ABCB6	ATP-binding cassette sub-family B member 6, mitochondrial	18.43	19.67	35.9	15	1.585
sp|P10253|LYAG_HUMAN	GAA	Lysosomal alpha-glucosidase	40.07	40.22	40.2	34	1.585
sp|P42345|MTOR_HUMAN	MTOR	Serine/threonine-protein kinase mTOR	56.89	60.24	30.6	31	1.586
sp|O00257|CBX4_HUMAN	CBX4	E3 SUMO-protein ligase CBX4	7.14	8.21	33.2	5	1.586
sp|Q9UI12|VATH_HUMAN	ATP6V1H	V-type proton ATPase subunit H	25.42	25.94	50.5	17	1.586
sp|P23677|IP3KA_HUMAN	ITPKA	Inositol-trisphosphate 3-kinase A	12.48	12.91	33.4	8	1.587
sp|Q969U7|PSMG2_HUMAN	PSMG2	Proteasome assembly chaperone 2	11.21	12.5	37.5	8	1.587
sp|Q8NFH3|NUP43_HUMAN	NUP43	Nucleoporin Nup43	13.51	15.69	37.6	14	1.588
sp|P27487|DPP4_HUMAN	DPP4	Dipeptidyl peptidase 4	29.4	29.5	33	15	1.589
sp|P53680|AP2S1_HUMAN	AP2S1	AP-2 complex subunit sigma	9.23	9.29	47.2	5	1.592
sp|Q15811|ITSN1_HUMAN	ITSN1	Intersectin-1	11.58	15.43	23.5	11	1.593
sp|Q9NVQ4|FAIM1_HUMAN	FAIM	Fas apoptotic inhibitory molecule 1	5.74	5.99	44.1	4	1.594
sp|Q6IN84|MRM1_HUMAN	MRM1	rRNA methyltransferase 1, mitochondrial	4.05	4.08	18.7	2	1.594
sp|P78537|BL1S1_HUMAN	BLOC1S1	Biogenesis of lysosome-related organelles complex 1 subunit 1	6.01	6.03	55.6	3	1.594
sp|Q8WUF5|IASPP_HUMAN	PPP1R13L	RelA-associated inhibitor	3.83	3.96	11.2	6	1.594
sp|Q8TF65|GIPC2_HUMAN	GIPC2	PDZ domain-containing protein GIPC2	3.92	6.67	41.9	5	1.596
sp|Q9H4L4|SENP3_HUMAN	SENP3	Sentrin-specific protease 3	12.56	12.61	31.2	6	1.596
sp|Q9NZL4|HPBP1_HUMAN	HSPBP1	Hsp70-binding protein 1	21.95	21.99	52.8	14	1.596
sp|Q9NX05|F120C_HUMAN	FAM120C	Constitutive coactivator of PPAR-gamma-like protein 2	2.02	3.68	13.1	3	1.597
sp|Q7Z417|NUFP2_HUMAN	NUFIP2	Nuclear fragile X mental retardation-interacting protein 2	25.61	26.39	41.7	14	1.601
sp|Q9Y6E0|STK24_HUMAN	STK24	Serine/threonine-protein kinase 24	12	27.07	47.2	14	1.601
sp|Q01850|CDR2_HUMAN	CDR2	Cerebellar degeneration-related protein 2	13.78	13.91	43.2	13	1.602
sp|Q8NFC6|BD1L1_HUMAN	BOD1L1	Biorientation of chromosomes in cell division protein 1-like 1	17.43	19.53	17.4	11	1.603
sp|Q9P2K8|E2AK4_HUMAN	EIF2AK4	Eukaryotic translation initiation factor 2-alpha kinase 4	10.52	12.54	20.5	8	1.603
sp|Q5JVS0|HABP4_HUMAN	HABP4	Intracellular hyaluronan-binding protein 4	6.02	8.03	30	7	1.603
sp|P12259|FA5_HUMAN	F5	Coagulation factor V	6.06	6.18	11.1	4	1.603
sp|Q9Y679|AUP1_HUMAN	AUP1	Ancient ubiquitous protein 1	15.07	15.15	37.8	14	1.604
sp|O60218|AK1BA_HUMAN	AKR1B10	Aldo-keto reductase family 1 member B10	42.01	45.63	92.1	38	1.605
sp|Q567U6|CCD93_HUMAN	CCDC93	Coiled-coil domain-containing protein 93	11.88	13.69	35.5	8	1.605
sp|Q9UMN6|KMT2B_HUMAN	KMT2B	Histone-lysine N-methyltransferase 2B	5.57	5.97	10.7	4	1.605
sp|Q9BW62|KATL1_HUMAN	KATNAL1	Katanin p60 ATPase-containing subunit A-like 1	2.39	8.1	32.7	7	1.606

sp|P31483|TIA1_HUMAN	TIA1	Nucleolysin TIA-1 isoform p40	23.68	23.82	63.7	17	1.609
sp|Q99541|PLIN2_HUMAN	PLIN2	Perilipin-2	29.22	29.27	66.6	21	1.610
sp|P48506|GSH1_HUMAN	GCLC	Glutamate--cysteine ligase catalytic subunit	50.21	50.59	55.1	28	1.611
sp|Q9NYL4|FKB11_HUMAN	FKBP11	Peptidyl-prolyl cis-trans isomerase FKBP11	4.56	4.61	34.3	6	1.613
sp|Q6P1R4|DUS1L_HUMAN	DUS1L	tRNA-dihydrouridine(16/17) synthase [NAD(P)(+)]-like	14.46	14.53	35.5	7	1.614
sp|P46199|IF2M_HUMAN	MTIF2	Translation initiation factor IF-2, mitochondrial	19.56	20.6	44.6	12	1.615
sp|Q14651|PLSI_HUMAN	PLS1	Plastin-1	18.88	31.5	50.1	22	1.616
sp|Q2TB10|ZN800_HUMAN	ZNF800	Zinc finger protein 800	4.94	5.07	19.1	3	1.616
sp|Q9BTT4|MED10_HUMAN	MED10	Mediator of RNA polymerase II transcription subunit 10	6.01	6.01	36.3	4	1.617
sp|P35790|CHKA_HUMAN	CHKA	Choline kinase alpha	7.13	7.4	39.4	6	1.617
sp|Q00013|EM55_HUMAN	MPP1	55 kDa erythrocyte membrane protein	15.77	15.96	49.6	11	1.618
sp|Q15067|ACOX1_HUMAN	ACOX1	Peroxisomal acyl-coenzyme A oxidase 1	46.71	47.14	55.3	35	1.620
sp|Q6UWM9|UD2A3_HUMAN	UGT2A3	UDP-glucuronosyltransferase 2A3	4.39	4.64	23.2	6	1.620
sp|Q9H1E5|TMX4_HUMAN	TMX4	Thioredoxin-related transmembrane protein 4	10	10	21.5	5	1.621
sp|Q3MIT2|PUS10_HUMAN	PUS10	Putative tRNA pseudouridine synthase Pus10	12.45	12.71	30.4	8	1.624
sp|Q8N4V1|MMGT1_HUMAN	MMGT1	Membrane magnesium transporter 1	4	4.01	29.8	3	1.625
sp|Q9GZM5|YIPF3_HUMAN	YIPF3	Protein YIPF3	8.4	10.49	28.6	6	1.625
sp|Q53SF7|COBL1_HUMAN	COBLL1	Cordon-bleu protein-like 1	19.38	19.63	30	12	1.626
sp|O95070|YIF1A_HUMAN	YIF1A	Protein YIF1A	5.43	5.5	16.4	3	1.627
sp|P35527|K1C9_HUMAN	KRT9	Keratin, type I cytoskeletal 9	50.75	53.04	54.7	49	1.628
sp|P54278|PMS2_HUMAN	PMS2	Mismatch repair endonuclease PMS2	4.12	4.19	17.5	2	1.629
sp|Q8NCH0|CHSTE_HUMAN	CHST14	Carbohydrate sulfotransferase 14	5.34	5.52	21	4	1.631
sp|O00418|EF2K_HUMAN	EEF2K	Eukaryotic elongation factor 2 kinase	19.86	20.53	30.5	12	1.633
sp|O14880|MGST3_HUMAN	MGST3	Microsomal glutathione S-transferase 3	4.95	5.03	29	6	1.635
sp|Q9Y2I7|FYV1_HUMAN	PIKFYVE	1-phosphatidylinositol 3-phosphate 5-kinase	6.33	6.83	17.4	6	1.636
sp|O14908|GIPC1_HUMAN	GIPC1	PDZ domain-containing protein GIPC1	21.23	22.48	59.8	12	1.638
sp|Q06323|PSME1_HUMAN	PSME1	Proteasome activator complex subunit 1	35.16	35.56	82.7	27	1.638
sp|P49354|FNTA_HUMAN	FNTA	Protein farnesyltransferase/geranylgeranyltransferase type-1 subunit alpha	14.19	14.21	35.4	8	1.638
sp|P30825|CTR1_HUMAN	SLC7A1	High affinity cationic amino acid transporter 1	9.8	9.93	26.7	7	1.639
sp|Q8NC42|RN149_HUMAN	RNF149	E3 ubiquitin-protein ligase RNF149	15.55	17.06	31.8	9	1.639
sp|Q92530|PSMF1_HUMAN	PSMF1	Proteasome inhibitor PI31 subunit	18.62	20	52.4	14	1.640
sp|Q9BPU6|DPYL5_HUMAN	DPYSL5	Dihydropyrimidinase-related protein 5	27.81	28.24	40.6	14	1.640
sp|Q9UI14|PRAF1_HUMAN	RABAC1	Prenylated Rab acceptor protein 1	2.36	2.39	35.7	2	1.643
sp|Q8WU76|SCFD2_HUMAN	SCFD2	Sec1 family domain-containing protein 2	6.54	7.12	26.5	6	1.643
sp|Q8TEW0|PARD3_HUMAN	PARD3	Partitioning defective 3 homolog	17.17	17.36	26.3	12	1.646
sp|Q9P2K2|TXD16_HUMAN	TXNDC16	Thioredoxin domain-containing protein 16	2.01	2.06	10.4	3	1.646
sp|Q9HBH5|RDH14_HUMAN	RDH14	Retinol dehydrogenase 14	9.83	13.24	46.1	8	1.650
sp|P55809|SCOT1_HUMAN	OXCT1	Succinyl-CoA:3-ketoacid coenzyme A transferase 1, mitochondrial	14.6	15.41	37.5	10	1.653
sp|Q9H0W9|CK054_HUMAN	C11orf54	Ester hydrolase C11orf54	6.7	6.78	36.8	6	1.653
sp|Q9H0R6|GATA_HUMAN	QRSL1	Glutamyl-tRNA(Gln) amidotransferase subunit A, mitochondrial	19.24	19.53	51.9	12	1.653
sp|Q96AE7|TTC17_HUMAN	TTC17	Tetratricopeptide repeat protein 17	2.88	5.16	14.9	5	1.655
sp|Q9UBS8|RNF14_HUMAN	RNF14	E3 ubiquitin-protein ligase RNF14	3.5	3.63	16.2	2	1.655
sp|Q9P265|DIP2B_HUMAN	DIP2B	Disco-interacting protein 2 homolog B	56.53	56.73	33.6	32	1.658
sp|P09234|RU1C_HUMAN	SNRPC	U1 small nuclear ribonucleoprotein C	6.54	6.58	32.1	7	1.658
sp|Q13370|PDE3B_HUMAN	PDE3B	cGMP-inhibited 3',5'-cyclic phosphodiesterase B	5.16	5.36	10.7	4	1.662
sp|O15440|MRP5_HUMAN	ABCC5	Multidrug resistance-associated protein 5	6.15	6.34	14.1	4	1.665
sp|Q96SQ9|CP2S1_HUMAN	CYP2S1	Cytochrome P450 2S1	2.01	2.02	12.5	3	1.666
sp|Q96CP6|GRM1A_HUMAN	GRAMD1A	GRAM domain-containing protein 1A	5.55	5.85	20.7	6	1.666
sp|Q9Y625|GPC6_HUMAN	GPC6	Glypican-6	14.88	15.28	36.6	13	1.668

sp|P31350|RIR2_HUMAN	RRM2	Ribonucleoside-diphosphate reductase subunit M2	25.39	26.17	66.3	16	1.668
sp|Q15654|TRIP6_HUMAN	TRIP6	Thyroid receptor-interacting protein 6	16.1	16.14	42.7	8	1.669
sp|O75781|PALM_HUMAN	PALM	Paralemmin-1	5.69	5.94	24.8	3	1.670
sp|P04156|PRIO_HUMAN	PRNP	Major prion protein	2.03	2.04	15	2	1.670
sp|P08582|TRFM_HUMAN	MFI2	Melanotransferrin	1.54	4.25	19.9	4	1.671
sp|O15514|RPB4_HUMAN	POLR2D	DNA-directed RNA polymerase II subunit RPB4	10.96	12.58	72.5	6	1.673
sp|Q9BW60|ELOV1_HUMAN	ELOVL1	Elongation of very long chain fatty acids protein 1	5.34	5.47	17.2	4	1.673
sp|Q96K76|UBP47_HUMAN	USP47	Ubiquitin carboxyl-terminal hydrolase 47	41.47	42.17	32.9	20	1.675
sp|Q12979|ABR_HUMAN	ABR	Active breakpoint cluster region-related protein	4.28	7.76	25.5	5	1.676
sp|Q13315|ATM_HUMAN	ATM	Serine-protein kinase ATM	18.78	20.38	21.3	13	1.676
sp|P05121|PAI1_HUMAN	SERPINE1	Plasminogen activator inhibitor 1	4.63	6.16	24.6	4	1.676
sp|Q04446|GLGB_HUMAN	GBE1	1,4-alpha-glucan-branching enzyme	16.76	18.4	35.3	10	1.676
sp|Q8NHM5|KDM2B_HUMAN	KDM2B	Lysine-specific demethylase 2B	2.26	4.33	16.2	3	1.678
sp|Q03518|TAP1_HUMAN	TAP1	Antigen peptide transporter 1	10.14	13.12	31.9	9	1.679
sp|Q53FV1|ORML2_HUMAN	ORMDL2	ORM1-like protein 2	2	6	32	3	1.683
sp|Q9Y371|SHLB1_HUMAN	SH3GLB1	Endophilin-B1	13.78	16.2	50.1	10	1.686
sp|Q16626|MEA1_HUMAN	MEA1	Male-enhanced antigen 1	4	4	18.9	2	1.687
sp|Q15819|UB2V2_HUMAN	UBE2V2	Ubiquitin-conjugating enzyme E2 variant 2	5.56	17.38	80	18	1.687
sp|Q9NX61|T161A_HUMAN	TMEM161A	Transmembrane protein 161A	8.01	8.04	30.1	4	1.689
sp|P78560|CRADD_HUMAN	CRADD	Death domain-containing protein CRADD	4	4	17.6	2	1.694
sp|Q7Z2K6|ERMP1_HUMAN	ERMP1	Endoplasmic reticulum metallopeptidase 1	26.59	27.07	32.6	17	1.695
sp|P53992|SC24C_HUMAN	SEC24C	Protein transport protein Sec24C	61.21	62.28	45.3	38	1.695
sp|Q9BX63|FANCJ_HUMAN	BRIP1	Fanconi anemia group J protein	1.91	2.15	17.4	3	1.696
sp|Q9UHJ6|SHPK_HUMAN	SHPK	Sedoheptulokinase	18.4	18.47	45.8	12	1.698
sp|P62699|YPEL5_HUMAN	YPEL5	Protein yippee-like 5	8.16	8.88	52.1	6	1.699
sp|Q7RTP6|MICA3_HUMAN	MICAL3	Protein-methionine sulfoxide oxidase MICAL3	10.68	11.1	15.3	7	1.703
sp|Q8IYR2|SMYD4_HUMAN	SMYD4	SET and MYND domain-containing protein 4	2	2.17	10	2	1.704
sp|P78509|RELN_HUMAN	RELN	Reelin	22.16	23.26	10.6	12	1.704
sp|Q9P215|POGK_HUMAN	POGK	Pogo transposable element with KRAB domain	4	4	8.9	4	1.705
sp|Q96PE7|MCEE_HUMAN	MCEE	Methylmalonyl-CoA epimerase, mitochondrial	8.91	9.05	69.9	6	1.705
sp|Q9BV23|ABHD6_HUMAN	ABHD6	Monoacylglycerol lipase ABHD6	5.05	5.19	36.2	3	1.705
sp|Q14914|PTGR1_HUMAN	PTGR1	Prostaglandin reductase 1	18.95	19.36	65.4	13	1.707
sp|Q2M296|MTHSD_HUMAN	MTHFSD	Methenyltetrahydrofolate synthase domain-containing protein	2.2	2.34	27.9	2	1.710
sp|P98172|EFNB1_HUMAN	EFNB1	Ephrin-B1	3.86	4.16	21.1	2	1.711
sp|Q16401|PSMD5_HUMAN	PSMD5	26S proteasome non-ATPase regulatory subunit 5	21.81	23.15	54.2	14	1.713
sp|Q96RK0|CIC_HUMAN	CIC	Protein capicua homolog	6.06	6.14	8.8	3	1.714
sp|Q96DX4|RSPRY_HUMAN	RSPRY1	RING finger and SPRY domain-containing protein 1	8.23	12.27	23.8	8	1.715
sp|Q9Y673|ALG5_HUMAN	ALG5	Dolichyl-phosphate beta-glucosyltransferase	14.64	14.7	44.1	9	1.716
sp|Q92759|TF2H4_HUMAN	GTF2H4	General transcription factor IIH subunit 4	10.01	10.02	21	6	1.717
sp|P48449|ERG7_HUMAN	LSS	Lanosterol synthase	40.36	40.64	47.4	26	1.719
sp|Q15154|PCM1_HUMAN	PCM1	Pericentriolar material 1 protein	32.93	33.49	21.2	22	1.719
sp|O75884|RBBP9_HUMAN	RBBP9	Putative hydrolase RBBP9	11.58	12.11	81.2	8	1.720
sp|Q13325|IFIT5_HUMAN	IFIT5	Interferon-induced protein with tetratricopeptide repeats 5	6.17	6.31	34.7	7	1.722
sp|Q9BRX2|PELO_HUMAN	PELO	Protein pelota homolog	25.49	25.69	64.4	19	1.722
sp|P08779|K1C16_HUMAN	KRT16	Keratin, type I cytoskeletal 16	1.74	24.97	43.1	25	1.723
sp|Q92828|COR2A_HUMAN	CORO2A	Coronin-2A	7.57	8.24	22.7	6	1.724
sp|Q9BTZ2|DHRS4_HUMAN	DHRS4	Dehydrogenase/reductase SDR family member 4	17.48	21.96	71.9	14	1.725
sp|O95059|RPP14_HUMAN	RPP14	Ribonuclease P protein subunit p14	4.65	4.69	55.7	3	1.725
sp|Q8N465|D2HDH_HUMAN	D2HGDH	D-2-hydroxyglutarate dehydrogenase, mitochondrial	14.05	14.3	38.6	13	1.725

sp|P55210|CASP7_HUMAN	CASP7	Caspase-7	6.15	7.18	46.5	8	1.728
sp|P51570|GALK1_HUMAN	GALK1	Galactokinase	25.53	25.6	52.6	17	1.728
sp|Q9BYG5|PAR6B_HUMAN	PARD6B	Partitioning defective 6 homolog beta	4.19	4.62	29	5	1.728
sp|O60504|VINEX_HUMAN	SORBS3	Vinexin	6.97	7.19	19.1	5	1.729
sp|Q96GX9|MTNB_HUMAN	APIP	Methylthioribulose-1-phosphate dehydratase	10.9	11.06	46.7	7	1.729
sp|Q8TCD5|NT5C_HUMAN	NT5C	5'(3')-deoxyribonucleotidase, cytosolic type	9.25	9.67	45.8	7	1.731
sp|Q8NHH9|ATLA2_HUMAN	ATL2	Atlastin-2	24.32	24.48	44.3	14	1.731
sp|P41214|EIF2D_HUMAN	EIF2D	Eukaryotic translation initiation factor 2D	28.74	29.05	63.4	15	1.731
sp|Q6P1M3|L2GL2_HUMAN	LLGL2	Lethal(2) giant larvae protein homolog 2	15.38	16.03	18.6	10	1.731
sp|Q9H4A6|GOLP3_HUMAN	GOLPH3	Golgi phosphoprotein 3	18.14	18.2	44	10	1.733
sp|O15357|SHIP2_HUMAN	INPPL1	Phosphatidylinositol 3,4,5-trisphosphate 5-phosphatase 2	7.95	12.59	21.9	11	1.734
sp|Q03188|CENPC_HUMAN	CENPC	Centromere protein C	7.73	8.06	24	7	1.734
sp|O60427|FADS1_HUMAN	FADS1	Fatty acid desaturase 1	4.19	4.27	21.2	3	1.735
sp|Q96SZ6|CK5P1_HUMAN	CDK5RAP1	CDK5 regulatory subunit-associated protein 1	23.69	23.77	41.9	14	1.737
sp|Q96PY5|FMNL2_HUMAN	FMNL2	Formin-like protein 2	13.41	13.73	27.3	8	1.740
sp|P51511|MMP15_HUMAN	MMP15	Matrix metalloproteinase-15	3.03	5.22	12.1	3	1.740
sp|P43250|GRK6_HUMAN	GRK6	G protein-coupled receptor kinase 6	13.82	13.96	27.6	8	1.744
sp|P01019|ANGT_HUMAN	AGT	Angiotensinogen	10	10.02	18.8	5	1.744
sp|Q8NBF6|AVL9_HUMAN	AVL9	Late secretory pathway protein AVL9 homolog	2.45	2.9	20.1	3	1.746
sp|O75792|RNH2A_HUMAN	RNASEH2A	Ribonuclease H2 subunit A	8.83	11.05	46.2	7	1.746
sp|Q9Y4B4|ARIP4_HUMAN	RAD54L2	Helicase ARIP4	4.28	4.47	11	3	1.746
sp|Q68CZ6|HAUS3_HUMAN	HAUS3	HAUS augmin-like complex subunit 3	15.26	15.47	30.2	12	1.748
sp|Q8N9M1|CS047_HUMAN	C19orf47	Uncharacterized protein C19orf47	6.74	6.89	23.9	4	1.749
sp|Q9NPF0|CD320_HUMAN	CD320	CD320 antigen	3.8	3.89	15.3	2	1.751
sp|Q9BXP2|S12A9_HUMAN	SLC12A9	Solute carrier family 12 member 9	8.79	8.98	22.2	7	1.751
sp|Q8N3E9|PLCD3_HUMAN	PLCD3	1-phosphatidylinositol 4,5-bisphosphate phosphodiesterase delta-3	8.62	8.74	21	5	1.754
sp|P55157|MTP_HUMAN	MTTP	Microsomal triglyceride transfer protein large subunit	40.81	42.29	46.8	28	1.754
sp|O95674|CDS2_HUMAN	CDS2	Phosphatidate cytidylyltransferase 2	10.46	10.64	25.4	8	1.756
sp|Q14451|GRB7_HUMAN	GRB7	Growth factor receptor-bound protein 7	9.24	9.35	26.3	6	1.757
sp|Q9HCG7|GBA2_HUMAN	GBA2	Non-lysosomal glucosylceramidase	1.85	2.11	12.9	3	1.759
sp|Q8WWI1|LMO7_HUMAN	LMO7	LIM domain only protein 7	58.22	60.4	40.2	32	1.760
sp|Q9Y6G5|COMDA_HUMAN	COMMD10	COMM domain-containing protein 10	4.58	4.62	29.7	5	1.760
sp|Q9Y618|NCOR2_HUMAN	NCOR2	Nuclear receptor corepressor 2	7.43	10.32	13.2	8	1.760
sp|P20585|MSH3_HUMAN	MSH3	DNA mismatch repair protein Msh3	20.23	20.78	31.2	18	1.761
sp|Q7Z7A3|CTU1_HUMAN	CTU1	Cytoplasmic tRNA 2-thiolation protein 1	7.49	7.63	37.1	5	1.762
sp|P57764|GSDMD_HUMAN	GSDMD	Gasdermin-D	8	8	22.9	4	1.763
sp|P12429|ANXA3_HUMAN	ANXA3	Annexin A3	13.86	13.99	42.1	10	1.766
sp|O95613|PCNT_HUMAN	PCNT	Pericentrin	4.99	6.32	18.9	13	1.767
sp|Q9BXS6|NUSAP_HUMAN	NUSAP1	Nucleolar and spindle-associated protein 1	10.97	12.46	37	7	1.767
sp|Q8IW35|CEP97_HUMAN	CEP97	Centrosomal protein of 97 kDa	5.55	6.39	20.5	5	1.768
sp|Q02487|DSC2_HUMAN	DSC2	Desmocollin-2	4.67	5	18.3	4	1.769
sp|E9PRG8|CK098_HUMAN	C11orf98	Uncharacterized protein C11orf98	2.07	2.27	39.3	2	1.769
sp|P08047|SP1_HUMAN	SP1	Transcription factor Sp1	9.7	9.81	17.5	7	1.769
sp|Q8TB52|FBX30_HUMAN	FBXO30	F-box only protein 30	9.04	9.35	13.8	6	1.770
sp|P49281|NRAM2_HUMAN	SLC11A2	Natural resistance-associated macrophage protein 2	3.04	3.11	15.1	2	1.772
sp|Q9H6V9|LDAH_HUMAN	LDAH	Lipid droplet-associated hydrolase	7.44	9.73	37.5	6	1.773
sp|Q8WXI9|P66B_HUMAN	GATAD2B	Transcriptional repressor p66-beta	26.83	27.93	46.9	16	1.773
sp|Q96H79|ZCCHL_HUMAN	ZC3HAV1L	Zinc finger CCCH-type antiviral protein 1-like	11.99	12.03	52.3	8	1.776
sp|Q71F23|CENPU_HUMAN	CENPU	Centromere protein U	3.58	3.91	19.9	4	1.776

sp|Q8TF01|PNISR_HUMAN	PNISR	Arginine/serine-rich protein PNISR	2.1	2.26	11.2	4	1.778
sp|Q86X76|NIT1_HUMAN	NIT1	Nitrilase homolog 1	9.23	9.52	42.2	9	1.778
sp|O43292|GPAA1_HUMAN	GPAA1	Glycosylphosphatidylinositol anchor attachment 1 protein	8.41	8.47	22.1	5	1.778
sp|O75387|LAT3_HUMAN	SLC43A1	Large neutral amino acids transporter small subunit 3	5.15	5.23	18.3	3	1.781
sp|P23921|RIR1_HUMAN	RRM1	Ribonucleoside-diphosphate reductase large subunit	68.53	69.56	69.4	55	1.783
sp|Q68DQ2|CRBG3_HUMAN	CRYBG3	Very large A-kinase anchor protein	4.56	5.27	12	7	1.789
sp|Q96G28|CFA36_HUMAN	CFAP36	Cilia- and flagella-associated protein 36	4.04	4.51	26	3	1.797
sp|Q96IK1|BOD1_HUMAN	BOD1	Biorientation of chromosomes in cell division protein 1	1.73	3.22	28.7	2	1.799
sp|Q13126|MTAP_HUMAN	MTAP	S-methyl-5'-thioadenosine phosphorylase	28.09	29.92	82.3	18	1.801
sp|Q3LXA3|TKFC_HUMAN	TKFC	Triokinase/FMN cyclase	43.78	44.1	55.5	35	1.803
sp|Q9GZY8|MFF_HUMAN	MFF	Mitochondrial fission factor	11.22	11.57	45.9	7	1.809
sp|Q9H269|VPS16_HUMAN	VPS16	Vacuolar protein sorting-associated protein 16 homolog	13.18	16.48	23.7	9	1.809
sp|Q13217|DNJC3_HUMAN	DNAJC3	DnaJ homolog subfamily C member 3	41.98	45.9	72.8	26	1.810
sp|O15047|SET1A_HUMAN	SETD1A	Histone-lysine N-methyltransferase SETD1A	8.99	9.22	12.3	6	1.811
sp|Q9HCK8|CHD8_HUMAN	CHD8	Chromodomain-helicase-DNA-binding protein 8	29.9	45.05	26.1	24	1.811
sp|Q9NR09|BIRC6_HUMAN	BIRC6	Baculoviral IAP repeat-containing protein 6	43.5	53.38	18	28	1.817
sp|Q96C12|ARMC5_HUMAN	ARMC5	Armadillo repeat-containing protein 5	3.48	3.82	9	2	1.819
sp|Q8TB37|NUBPL_HUMAN	NUBPL	Iron-sulfur protein NUBPL	15.7	17.48	52.4	11	1.819
sp|Q9BQE4|SELS_HUMAN	VIMP	Selenoprotein S	2.53	2.8	35.5	2	1.821
sp|Q9HB90|RRAGC_HUMAN	RRAGC	Ras-related GTP-binding protein C	19.7	19.75	53.6	10	1.826
sp|Q9NRG1|PRDC1_HUMAN	PRTFDC1	Phosphoribosyltransferase domain-containing protein 1	3.87	6.94	47.1	7	1.827
sp|Q8N5A5|ZGPAT_HUMAN	ZGPAT	Zinc finger CCCH-type with G patch domain-containing protein	18.97	19.07	36.4	11	1.828
sp|Q15058|KIF14_HUMAN	KIF14	Kinesin-like protein KIF14	6.1	6.41	18.9	5	1.828
sp|Q8TEA8|DTD1_HUMAN	DTD1	D-tyrosyl-tRNA(Tyr) deacylase 1	7.3	7.54	42.1	5	1.831
sp|Q8N392|RHG18_HUMAN	ARHGAP18	Rho GTPase-activating protein 18	47.05	47.37	59.6	26	1.832
sp|Q14653|IRF3_HUMAN	IRF3	Interferon regulatory factor 3	12.23	12.45	36.5	8	1.833
sp|Q9UI10|EI2BD_HUMAN	EIF2B4	Translation initiation factor eIF-2B subunit delta	31.03	31.11	52.6	19	1.837
sp|Q9BY50|SC11C_HUMAN	SEC11C	Signal peptidase complex catalytic subunit SEC11C	6	6	28.1	3	1.837
sp|O00767|ACOD_HUMAN	SCD	Acyl-CoA desaturase	7.99	8.05	25.9	5	1.839
sp|P22455|FGFR4_HUMAN	FGFR4	Fibroblast growth factor receptor 4	13.42	15.55	22.3	8	1.839
sp|Q9H1H9|KI13A_HUMAN	KIF13A	Kinesin-like protein KIF13A	7.74	8.03	18.2	4	1.839
sp|Q08426|ECHP_HUMAN	EHHADH	Peroxisomal bifunctional enzyme	49.74	50.24	59.5	25	1.839
sp|Q9UBK9|UXT_HUMAN	UXT	Protein UXT	2	2.06	20.4	2	1.843
sp|Q8NDI1|EHBP1_HUMAN	EHBP1	EH domain-binding protein 1	30.68	31.53	38.3	21	1.846
sp|P51114|FXR1_HUMAN	FXR1	Fragile X mental retardation syndrome-related protein 1	40.78	42.73	59.9	27	1.850
sp|P06454|PTMA_HUMAN	PTMA	Prothymosin alpha	2.87	3.74	39.6	8	1.854
sp|Q96H55|MYO19_HUMAN	MYO19	Unconventional myosin-XIX	12.53	12.97	20.2	8	1.855
sp|Q6IQ23|PKHA7_HUMAN	PLEKHA7	Pleckstrin homology domain-containing family A member 7	9.9	10.19	17	7	1.857
sp|Q9UEU0|VTI1B_HUMAN	VTI1B	Vesicle transport through interaction with t-SNAREs homolog 1B	7.43	7.67	33.6	5	1.858
sp|Q9H0U6|RM18_HUMAN	MRPL18	39S ribosomal protein L18, mitochondrial	6.52	6.77	46.1	6	1.860
sp|Q12866|MERTK_HUMAN	MERTK	Tyrosine-protein kinase Mer	2.01	3.3	13.2	2	1.861
sp|O15533|TPSN_HUMAN	TAPBP	Tapasin	2.7	2.85	14.1	3	1.861
sp|P00519|ABL1_HUMAN	ABL1	Tyrosine-protein kinase ABL1	2.69	4.88	16.4	4	1.863
sp|Q68CP9|ARID2_HUMAN	ARID2	AT-rich interactive domain-containing protein 2	21.62	21.91	18.8	11	1.863
sp|Q15528|MED22_HUMAN	MED22	Mediator of RNA polymerase II transcription subunit 22	3.57	3.83	28	4	1.865
sp|P34913|HYES_HUMAN	EPHX2	Bifunctional epoxide hydrolase 2	15.92	16.68	36.2	11	1.866
sp|Q9HBK9|AS3MT_HUMAN	AS3MT	Arsenite methyltransferase	6.72	7.19	39.2	6	1.866
sp|Q12770|SCAP_HUMAN	SCAP	Sterol regulatory element-binding protein cleavage-activating protein	6.49	6.67	13.5	6	1.868
sp|Q8WUX9|CHMP7_HUMAN	CHMP7	Charged multivesicular body protein 7	7.39	7.68	37.5	6	1.876

sp|Q8IYS2|K2013_HUMAN	KIAA2013	Uncharacterized protein KIAA2013	15.41	15.69	31.2	9	1.878
sp|Q9H1C4|UN93B_HUMAN	UNC93B1	Protein unc-93 homolog B1	1.34	1.58	9.2	2	1.884
sp|Q2T9J0|TYSD1_HUMAN	TYSND1	Peroxisomal leader peptide-processing protease	4.03	4.03	12.5	3	1.890
sp|O95551|TYDP2_HUMAN	TDP2	Tyrosyl-DNA phosphodiesterase 2	11.62	11.8	35.1	7	1.893
sp|Q2KHT3|CL16A_HUMAN	CLEC16A	Protein CLEC16A	4.06	4.18	12.8	3	1.896
sp|O60293|ZC3H1_HUMAN	ZFC3H1	Zinc finger C3H1 domain-containing protein	8.18	9.43	19.6	11	1.902
sp|Q9UK23|NAGPA_HUMAN	NAGPA	N-acetylglucosamine-1-phosphodiester alpha-N-acetylglucosaminidase	11	11.13	32.6	8	1.904
sp|Q14999|CUL7_HUMAN	CUL7	Cullin-7	9.65	9.78	14.7	7	1.906
sp|Q9Y6M5|ZNT1_HUMAN	SLC30A1	Zinc transporter 1	11.3	11.59	27.4	10	1.911
sp|Q9NUN5|LMBD1_HUMAN	LMBRD1	Probable lysosomal cobalamin transporter	2.84	2.91	12.2	4	1.920
sp|Q8NG11|TSN14_HUMAN	TSPAN14	Tetraspanin-14	4.7	4.76	23.3	3	1.923
sp|Q92547|TOPB1_HUMAN	TOPBP1	DNA topoisomerase 2-binding protein 1	21.33	22.8	27.7	15	1.926
sp|P51790|CLCN3_HUMAN	CLCN3	H(+)/Cl(-) exchange transporter 3	4.77	5.45	18.5	4	1.931
sp|Q15018|F175B_HUMAN	FAM175B	BRISC complex subunit Abro1	8.13	8.36	23.4	6	1.939
sp|Q86XZ4|SPAS2_HUMAN	SPATS2	Spermatogenesis-associated serine-rich protein 2	12.09	12.56	26.1	7	1.940
sp|Q9BRQ8|AIFM2_HUMAN	AIFM2	Apoptosis-inducing factor 2	16.01	16.05	45.3	10	1.943
sp|Q9HCM3|K1549_HUMAN	KIAA1549	UPF0606 protein KIAA1549	2	2.24	5.2	3	1.947
sp|Q8IVH8|M4K3_HUMAN	MAP4K3	Mitogen-activated protein kinase kinase kinase kinase 3	5.22	7.26	15	8	1.948
sp|Q969K3|RNF34_HUMAN	RNF34	E3 ubiquitin-protein ligase RNF34	3.44	3.57	16.4	3	1.948
sp|P07197|NFM_HUMAN	NEFM	Neurofilament medium polypeptide	2.01	4.47	22.3	9	1.948
sp|O75896|TUSC2_HUMAN	TUSC2	Tumor suppressor candidate 2	3.53	3.69	48.2	2	1.951
sp|Q4L180|FIL1L_HUMAN	FILIP1L	Filamin A-interacting protein 1-like	3.29	3.69	27.1	6	1.951
sp|P13929|ENOB_HUMAN	ENO3	Beta-enolase	8.35	32.82	77.9	105	1.953
sp|Q96F07|CYFP2_HUMAN	CYFIP2	Cytoplasmic FMR1-interacting protein 2	6.13	31.02	32.6	19	1.954
sp|O75208|COQ9_HUMAN	COQ9	Ubiquinone biosynthesis protein COQ9, mitochondrial	8.72	9.32	25.5	7	1.955
sp|O75764|TCEA3_HUMAN	TCEA3	Transcription elongation factor A protein 3	8.53	8.82	37.4	7	1.956
sp|Q92947|GCDH_HUMAN	GCDH	Glutaryl-CoA dehydrogenase, mitochondrial	25.78	26.84	70.1	23	1.957
sp|P01130|LDLR_HUMAN	LDLR	Low-density lipoprotein receptor	17.67	17.81	25.9	10	1.959
sp|Q14353|GAMT_HUMAN	GAMT	Guanidinoacetate N-methyltransferase	13.65	13.8	68.6	10	1.963
sp|Q9NVM9|ASUN_HUMAN	ASUN	Protein asunder homolog	11.37	11.82	31.4	9	1.966
sp|Q7LG56|RIR2B_HUMAN	RRM2B	Ribonucleoside-diphosphate reductase subunit M2 B	6.01	9.12	28.5	6	1.968
sp|Q86TU7|SETD3_HUMAN	SETD3	Histone-lysine N-methyltransferase setd3	14.64	21.11	49.3	11	1.969
sp|Q9NPD8|UBE2T_HUMAN	UBE2T	Ubiquitin-conjugating enzyme E2 T	17.74	17.91	57.9	12	1.969
sp|Q9H792|PEAK1_HUMAN	PEAK1	Pseudopodium-enriched atypical kinase 1	2.6	2.66	12	3	1.970
sp|P02787|TRFE_HUMAN	TF	Serotransferrin	61.87	66.09	75.6	44	1.970
sp|P53801|PTTG_HUMAN	PTTG1IP	Pituitary tumor-transforming gene 1 protein-interacting protein	4	4	13.9	2	1.971
sp|P06396|GELS_HUMAN	GSN	Gelsolin	21.47	21.92	33.6	13	1.976
sp|Q86WQ0|NR2CA_HUMAN	NR2C2AP	Nuclear receptor 2C2-associated protein	4.15	4.18	49.6	6	1.983
sp|Q9NSC2|SALL1_HUMAN	SALL1	Sal-like protein 1	2.48	2.51	7.5	2	1.987
sp|P47224|MSS4_HUMAN	RABIF	Guanine nucleotide exchange factor MSS4	4.61	4.78	36.6	4	1.988
sp|P10398|ARAF_HUMAN	ARAF	Serine/threonine-protein kinase A-Raf	16.88	17.18	31.2	11	1.990
sp|O60292|SI1L3_HUMAN	SIPA1L3	Signal-induced proliferation-associated 1-like protein 3	3.94	6.18	12.1	5	1.993
sp|O75410|TACC1_HUMAN	TACC1	Transforming acidic coiled-coil-containing protein 1	19.33	20.17	35.3	13	1.998
sp|Q9UK22|FBX2_HUMAN	FBXO2	F-box only protein 2	25.85	25.92	79.1	19	1.998
sp|Q5R3I4|TTC38_HUMAN	TTC38	Tetratricopeptide repeat protein 38	29.98	30.45	68.2	27	1.998
sp|Q13884|SNTB1_HUMAN	SNTB1	Beta-1-syntrophin	27.88	30.76	55.2	18	1.998
sp|P61599|NAA20_HUMAN	NAA20	N-alpha-acetyltransferase 20	11.59	11.73	62.9	7	2.000
sp|Q96JJ3|ELMO2_HUMAN	ELMO2	Engulfment and cell motility protein 2	20.77	21.15	39.3	14	2.002
sp|Q7Z3C6|ATG9A_HUMAN	ATG9A	Autophagy-related protein 9A	18.02	19.99	20.4	12	2.003

sp|P13647|K2C5_HUMAN	KRT5	Keratin, type II cytoskeletal 5	15.78	37.38	37.8	26	2.004
sp|Q13630|FCL_HUMAN	TSTA3	GDP-L-fucose synthase	15.55	15.63	57.3	9	2.005
sp|O95302|FKBP9_HUMAN	FKBP9	Peptidyl-prolyl cis-trans isomerase FKBP9	22.38	22.55	41.9	14	2.008
sp|Q9UBD5|ORC3_HUMAN	ORC3	Origin recognition complex subunit 3	20.19	20.59	28.3	11	2.008
sp|Q92610|ZN592_HUMAN	ZNF592	Zinc finger protein 592	7.38	8.03	18.7	8	2.008
sp|Q5T6V5|CI064_HUMAN	C9orf64	UPF0553 protein C9orf64	15.27	16.64	48.7	11	2.012
sp|Q14161|GIT2_HUMAN	GIT2	ARF GTPase-activating protein GIT2	22.66	23.85	30.3	14	2.015
sp|Q9UP95|S12A4_HUMAN	SLC12A4	Solute carrier family 12 member 4	8.55	8.97	19.9	8	2.022
sp|Q92796|DLG3_HUMAN	DLG3	Disks large homolog 3	7.92	10.28	27.5	7	2.023
sp|Q7Z589|EMSY_HUMAN	EMSY	Protein EMSY	6.47	6.66	16.9	5	2.024
sp|Q9NXS2|QPCTL_HUMAN	QPCTL	Glutaminyl-peptide cyclotransferase-like protein	15.41	15.5	31.7	8	2.025
sp|P42771|CDN2A_HUMAN	CDKN2A	Cyclin-dependent kinase inhibitor 2A	7.62	7.71	70.5	6	2.026
sp|Q86W56|PARG_HUMAN	PARG	Poly(ADP-ribose) glycohydrolase	9.06	9.29	24.1	5	2.030
sp|Q96RL1|UIMC1_HUMAN	UIMC1	BRCA1-A complex subunit RAP80	4.08	4.23	20.2	3	2.030
sp|O95772|MENTO_HUMAN	STARD3NL	MLN64 N-terminal domain homolog	4.49	4.6	32.9	3	2.035
sp|P62942|FKB1A_HUMAN	FKBP1A	Peptidyl-prolyl cis-trans isomerase FKBP1A	12.34	12.83	82.4	15	2.039
sp|Q8TB03|CX038_HUMAN	CXorf38	Uncharacterized protein CXorf38	2.23	2.27	27.6	3	2.045
sp|O43739|CYH3_HUMAN	CYTH3	Cytohesin-3	2	2.03	13.3	2	2.048
sp|Q96MN5|TEAN2_HUMAN	TCEANC2	Transcription elongation factor A N-terminal and central domain-containing protein 2	2	2.05	25	2	2.055
sp|P46108|CRK_HUMAN	CRK	Adapter molecule crk	16	18	45.7	10	2.057
sp|Q5T3F8|CSCL2_HUMAN	TMEM63B	CSC1-like protein 2	2.39	2.48	12.6	2	2.061
sp|Q8N142|PURA1_HUMAN	ADSSL1	Adenylosuccinate synthetase isozyme 1	2.01	6.05	22.1	3	2.066
sp|Q8IWT6|LRC8A_HUMAN	LRRC8A	Volume-regulated anion channel subunit LRRC8A	17.95	20.62	30.7	16	2.070
sp|P16455|MGMT_HUMAN	MGMT	Methylated-DNA--protein-cysteine methyltransferase	4.36	4.45	36.2	5	2.077
sp|P40818|UBP8_HUMAN	USP8	Ubiquitin carboxyl-terminal hydrolase 8	19.7	21.69	33	13	2.083
sp|Q9UGT4|SUSD2_HUMAN	SUSD2	Sushi domain-containing protein 2	2	2.04	7.8	2	2.084
sp|Q9P2T1|GMPR2_HUMAN	GMPR2	GMP reductase 2	26.76	27.11	73	16	2.089
sp|P48728|GCST_HUMAN	AMT	Aminomethyltransferase, mitochondrial	2.24	2.36	21.8	3	2.093
sp|Q9NWM8|FKB14_HUMAN	FKBP14	Peptidyl-prolyl cis-trans isomerase FKBP14	11.09	11.21	41.7	8	2.097
sp|Q8NEZ5|FBX22_HUMAN	FBXO22	F-box only protein 22	27.08	27.16	54.1	22	2.099
sp|Q9UJM3|ERRFI_HUMAN	ERRFI1	ERBB receptor feedback inhibitor 1	2.19	2.32	15.6	3	2.119
sp|Q9NZM3|ITSN2_HUMAN	ITSN2	Intersectin-2	16.41	17.75	24.6	11	2.123
sp|P10588|NR2F6_HUMAN	NR2F6	Nuclear receptor subfamily 2 group F member 6	4.79	4.85	19.3	3	2.123
sp|P60709|ACTB_HUMAN	ACTB	Actin, cytoplasmic 1	2	105.23	92.3	466	2.127
sp|P63261|ACTG_HUMAN	ACTG1	Actin, cytoplasmic 2	103.46	106.24	92.3	473	2.127
sp|Q9P2C4|TM181_HUMAN	TMEM181	Transmembrane protein 181	5.24	5.44	17.7	6	2.128
sp|Q6ZSR9|YJ005_HUMAN	2	Uncharacterized protein FLJ45252	10	10.85	28.2	7	2.129
sp|P11166|GTR1_HUMAN	SLC2A1	Solute carrier family 2, facilitated glucose transporter member 1	13.55	13.85	18.1	12	2.132
sp|Q8WXG6|MADD_HUMAN	MADD	MAP kinase-activating death domain protein	6.27	6.45	15	4	2.137
sp|P08727|K1C19_HUMAN	KRT19	Keratin, type I cytoskeletal 19	69.67	76.54	88.3	80	2.141
sp|O60613|SEP15_HUMAN	15-Sep	15 kDa selenoprotein	8.55	8.66	54.9	6	2.142
sp|P36915|GNL1_HUMAN	GNL1	Guanine nucleotide-binding protein-like 1	24.03	24.08	29.7	13	2.144
sp|P80297|MT1X_HUMAN	MT1X	Metallothionein-1X	5.22	5.29	34.4	3	2.146
sp|Q8IZ52|CHSS2_HUMAN	CHPF	Chondroitin sulfate synthase 2	3.93	4.03	16	2	2.151
sp|Q7Z739|YTHD3_HUMAN	YTHDF3	YTH domain-containing family protein 3	11.68	18.38	28.9	13	2.153
sp|Q8TEB1|DCA11_HUMAN	DCAF11	DDB1- and CUL4-associated factor 11	12.23	12.39	36.5	9	2.155
sp|P50135|HNMT_HUMAN	HNMT	Histamine N-methyltransferase	8.93	9.2	44.2	11	2.161
sp|Q02252|MMSA_HUMAN	ALDH6A1	Methylmalonate-semialdehyde dehydrogenase [acylating], mitochondrial	41.9	42.57	57.8	26	2.166
sp|Q9ULC5|ACSL5_HUMAN	ACSL5	Long-chain-fatty-acid--CoA ligase 5	30.09	32.74	49.2	20	2.167

sp|O95786|DDX58_HUMAN	DDX58	Probable ATP-dependent RNA helicase DDX58	3.41	3.99	19.8	4	2.170
sp|P43007|SATT_HUMAN	SLC1A4	Neutral amino acid transporter A	9.68	9.88	28.8	7	2.177
sp|P35908|K22E_HUMAN	KRT2	Keratin, type II cytoskeletal 2 epidermal	57.79	69.24	72.1	62	2.177
sp|Q9GZU2|PEG3_HUMAN	PEG3	Paternally-expressed gene 3 protein	44.04	44.15	29.7	28	2.186
sp|Q32P44|EMAL3_HUMAN	EML3	Echinoderm microtubule-associated protein-like 3	8.51	9.12	22.3	6	2.188
sp|Q96GX2|A7L3B_HUMAN	ATXN7L3B	Putative ataxin-7-like protein 3B	3.64	3.72	30.9	2	2.196
sp|P05534|1A24_HUMAN	HLA-A	HLA class I histocompatibility antigen, A-24 alpha chain	8.04	18.04	48.5	11	2.203
sp|Q6XZF7|DNMBP_HUMAN	DNMBP	Dynamin-binding protein	4.56	4.78	15.3	4	2.203
sp|Q8TD43|TRPM4_HUMAN	TRPM4	Transient receptor potential cation channel subfamily M member 4	2.18	4.3	11.5	3	2.215
sp|Q9Y312|AAR2_HUMAN	AAR2	Protein AAR2 homolog	11.56	11.78	45.1	9	2.219
sp|P02652|APOA2_HUMAN	APOA2	Apolipoprotein A-II	7.14	7.28	70	4	2.220
sp|P21980|TGM2_HUMAN	TGM2	Protein-glutamine gamma-glutamyltransferase 2	27.59	28.62	47.9	22	2.221
sp|Q63HN8|RN213_HUMAN	RNF213	E3 ubiquitin-protein ligase RNF213	76.27	81	25	45	2.229
sp|O95954|FTCD_HUMAN	FTCD	Formimidoyltransferase-cyclodeaminase	31.72	32.17	57.5	20	2.232
sp|P02649|APOE_HUMAN	APOE	Apolipoprotein E	28.11	28.16	58.7	18	2.242
sp|Q6P4R8|NFRKB_HUMAN	NFRKB	Nuclear factor related to kappa-B-binding protein	3.87	4.24	16.3	4	2.242
sp|Q29963|1C06_HUMAN	HLA-C	HLA class I histocompatibility antigen, Cw-6 alpha chain	8.56	14.85	42.4	8	2.263
sp|Q5RI15|COX20_HUMAN	COX20	Cytochrome c oxidase protein 20 homolog	4.34	4.39	37.3	4	2.270
sp|Q12802|AKP13_HUMAN	AKAP13	A-kinase anchor protein 13	12.72	13.29	15.7	9	2.271
sp|Q9BRZ2|TRI56_HUMAN	TRIM56	E3 ubiquitin-protein ligase TRIM56	5.41	5.62	19.5	6	2.273
sp|Q96CX2|KCD12_HUMAN	KCTD12	BTB/POZ domain-containing protein KCTD12	11.27	11.39	47.1	7	2.274
sp|Q86W74|ANR46_HUMAN	ANKRD46	Ankyrin repeat domain-containing protein 46	4.01	4.02	25.9	3	2.279
sp|Q8N0V3|RBFA_HUMAN	RBFA	Putative ribosome-binding factor A, mitochondrial	13.97	14.06	44	8	2.281
sp|Q9NVP2|ASF1B_HUMAN	ASF1B	Histone chaperone ASF1B	6.13	8.06	52	5	2.284
sp|Q9H6A9|PCX3_HUMAN	PCNXL3	Pecanex-like protein 3	3.11	3.34	6.9	4	2.290
sp|P59768|GBG2_HUMAN	GNG2	Guanine nucleotide-binding protein G(I)/G(S)/G(O) subunit gamma-2	1.54	3.75	36.6	2	2.295
sp|Q14155|ARHG7_HUMAN	ARHGEF7	Rho guanine nucleotide exchange factor 7	9.18	9.3	22.9	7	2.298
sp|P13056|NR2C1_HUMAN	NR2C1	Nuclear receptor subfamily 2 group C member 1	4.35	4.47	22.2	3	2.299
sp|Q9P2J3|KLHL9_HUMAN	KLHL9	Kelch-like protein 9	4.43	4.58	17.2	3	2.310
sp|Q9Y5W7|SNX14_HUMAN	SNX14	Sorting nexin-14	4.02	4.29	19.1	6	2.315
sp|Q8NF91|SYNE1_HUMAN	SYNE1	Nesprin-1	5.31	10.57	18.7	17	2.335
sp|Q96HN2|SAHH3_HUMAN	AHCYL2	Adenosylhomocysteinase 3	3.49	29.25	37.5	21	2.344
sp|Q9NS87|KIF15_HUMAN	KIF15	Kinesin-like protein KIF15	27.07	29.92	36.7	16	2.350
sp|P37059|DHB2_HUMAN	HSD17B2	Estradiol 17-beta-dehydrogenase 2	10.82	10.94	30.8	6	2.355
sp|Q9C0B7|TNG6_HUMAN	TANGO6	Transport and Golgi organization protein 6 homolog	15.29	17.41	26.4	10	2.356
sp|P28332|ADH6_HUMAN	ADH6	Alcohol dehydrogenase 6	13.47	13.61	29.6	7	2.378
sp|Q5T0N5|FBP1L_HUMAN	FNBP1L	Formin-binding protein 1-like	12.58	12.98	39.8	7	2.379
sp|P27338|AOFB_HUMAN	MAOB	Amine oxidase [flavin-containing] B	23.64	24.95	36.9	14	2.383
sp|Q2TB90|HKDC1_HUMAN	HKDC1	Putative hexokinase HKDC1	26.76	37.13	36.6	19	2.392
sp|P46934|NEDD4_HUMAN	NEDD4	E3 ubiquitin-protein ligase NEDD4	10.04	10.14	12.6	5	2.404
sp|P61626|LYSC_HUMAN	LYZ	Lysozyme C	13.23	13.36	59.5	12	2.406
sp|Q96EK9|KTI12_HUMAN	KTI12	Protein KTI12 homolog	10.82	10.89	42.9	6	2.415
sp|Q9UL12|SARDH_HUMAN	SARDH	Sarcosine dehydrogenase, mitochondrial	25.97	26.14	39.7	13	2.418
sp|Q6NUQ4|TM214_HUMAN	TMEM214	Transmembrane protein 214	39.88	40.48	42.1	23	2.420
sp|Q8NHP6|MSPD2_HUMAN	MOSPD2	Motile sperm domain-containing protein 2	8.36	8.65	32.1	6	2.422
sp|O14730|RIOK3_HUMAN	RIOK3	Serine/threonine-protein kinase RIO3	3.02	3.14	18.7	2	2.427
sp|Q9BXK1|KLF16_HUMAN	KLF16	Krueppel-like factor 16	5.51	5.57	44.4	3	2.437
sp|Q8NB46|ANR52_HUMAN	ANKRD52	Serine/threonine-protein phosphatase 6 regulatory ankyrin repeat subunit C	6.02	6.22	14.3	4	2.442
sp|Q99988|GDF15_HUMAN	GDF15	Growth/differentiation factor 15	4.45	4.57	38	3	2.442

sp|Q6Y288|B3GLT_HUMAN	B3GALTL	Beta-1,3-glucosyltransferase	6.82	7.81	27.9	5	2.450
sp|Q9H479|FN3K_HUMAN	FN3K	Fructosamine-3-kinase	16.01	16.06	43	8	2.462
sp|Q2VPB7|AP5B1_HUMAN	AP5B1	AP-5 complex subunit beta-1	8.83	8.92	18.9	7	2.484
sp|Q13424|SNTA1_HUMAN	SNTA1	Alpha-1-syntrophin	6.17	7.23	29.1	4	2.489
sp|P07306|ASGR1_HUMAN	ASGR1	Asialoglycoprotein receptor 1	26.05	26.09	70.1	20	2.511
sp|Q86YH6|DLP1_HUMAN	PDSS2	Decaprenyl-diphosphate synthase subunit 2	9.36	9.54	32.8	5	2.518
sp|Q15493|RGN_HUMAN	RGN	Regucalcin	4.44	4.5	29.8	3	2.524
sp|Q9NQB0|TF7L2_HUMAN	TCF7L2	Transcription factor 7-like 2	1.37	1.5	15.4	2	2.528
sp|P37235|HPCL1_HUMAN	HPCAL1	Hippocalcin-like protein 1	9.18	13.35	56	7	2.533
sp|Q7Z6J9|SEN54_HUMAN	TSEN54	tRNA-splicing endonuclease subunit Sen54	3.07	3.29	13.3	3	2.536
sp|O15427|MOT4_HUMAN	SLC16A3	Monocarboxylate transporter 4	12.75	12.84	21.9	7	2.537
sp|Q9Y508|RN114_HUMAN	RNF114	E3 ubiquitin-protein ligase RNF114	11.25	11.32	54	6	2.549
sp|P16591|FER_HUMAN	FER	Tyrosine-protein kinase Fer	8.71	11.41	34.1	9	2.575
sp|Q96QR8|PURB_HUMAN	PURB	Transcriptional activator protein Pur-beta	10.75	13.28	53.9	9	2.580
sp|Q9BQC3|DPH2_HUMAN	DPH2	Diphthamide biosynthesis protein 2	10.24	10.39	33.1	7	2.605
sp|P29279|CTGF_HUMAN	CTGF	Connective tissue growth factor	6.8	7.01	22.9	5	2.615
sp|P31025|LCN1_HUMAN	LCN1	Lipocalin-1	3.33	3.45	29.6	2	2.620
sp|Q01650|LAT1_HUMAN	SLC7A5	Large neutral amino acids transporter small subunit 1	8.31	8.4	19.5	6	2.656
sp|Q6ZRI6|CO039_HUMAN	C15orf39	Uncharacterized protein C15orf39	4.01	4.02	8.7	2	2.684
sp|O60784|TOM1_HUMAN	TOM1	Target of Myb protein 1	29.03	29.23	57.9	21	2.689
sp|Q9HD23|MRS2_HUMAN	MRS2	Magnesium transporter MRS2 homolog, mitochondrial	12.1	12.26	42.7	7	2.700
sp|O60281|ZN292_HUMAN	ZNF292	Zinc finger protein 292	3.05	4.76	11.1	9	2.711
sp|Q6SPF0|SAMD1_HUMAN	SAMD1	Atherin	11.65	11.81	29.7	8	2.715
sp|Q9ULV0|MYO5B_HUMAN	MYO5B	Unconventional myosin-Vb	2.91	11.1	21.7	8	2.760
sp|Q9H082|RB33B_HUMAN	RAB33B	Ras-related protein Rab-33B	4	7.85	25.8	6	2.773
sp|Q96QZ7|MAGI1_HUMAN	MAGI1	Membrane-associated guanylate kinase, WW and PDZ domain-containing protein 1	13.77	15.22	17.6	9	2.773
sp|Q9H9Q2|CSN7B_HUMAN	COPS7B	COP9 signalosome complex subunit 7b	16.45	17.36	51.9	11	2.793
sp|P05412|JUN_HUMAN	JUN	Transcription factor AP-1	4	4.06	30.5	2	2.808
sp|Q8IWV7|UBR1_HUMAN	UBR1	E3 ubiquitin-protein ligase UBR1	9.75	9.94	13.8	7	2.820
sp|Q13488|VPP3_HUMAN	TCIRG1	V-type proton ATPase 116 kDa subunit a isoform 3	5.17	5.41	13.7	5	2.823
sp|Q8N128|F177A_HUMAN	FAM177A1	Protein FAM177A1	5.84	5.93	39.4	3	2.826
sp|P02792|FRIL_HUMAN	FTL	Ferritin light chain	7.11	7.79	42.3	7	2.828
sp|Q8NFG4|FLCN_HUMAN	FLCN	Folliculin	4.66	4.92	19.3	4	2.849
sp|Q13443|ADAM9_HUMAN	ADAM9	Disintegrin and metalloproteinase domain-containing protein 9	12.11	12.14	24.1	9	2.886
sp|Q96LD4|TRI47_HUMAN	TRIM47	Tripartite motif-containing protein 47	14.12	14.4	34	9	2.888
sp|Q9BPZ3|PAIP2_HUMAN	PAIP2	Polyadenylate-binding protein-interacting protein 2	4.89	4.95	46.5	3	2.911
sp|Q92618|ZN516_HUMAN	ZNF516	Zinc finger protein 516	2.36	2.42	9.6	2	2.981
sp|Q15170|TCAL1_HUMAN	TCEAL1	Transcription elongation factor A protein-like 1	4	4.01	29.3	3	2.984
sp|P40121|CAPG_HUMAN	CAPG	Macrophage-capping protein	5.8	6.57	32.8	5	2.997
sp|Q96RG2|PASK_HUMAN	PASK	PAS domain-containing serine/threonine-protein kinase	2.28	2.33	11.7	2	3.016
sp|Q9UQL6|HDAC5_HUMAN	HDAC5	Histone deacetylase 5	2.14	2.25	13.3	3	3.024
sp|Q8IW45|NNRD_HUMAN	CARKD	ATP-dependent (S)-NAD(P)H-hydrate dehydratase	8.45	8.57	45.8	5	3.121
sp|O60825|F262_HUMAN	PFKFB2	6-phosphofructo-2-kinase/fructose-2,6-bisphosphatase 2	17.16	17.47	45.7	10	3.134
sp|P02760|AMBP_HUMAN	AMBP	Protein AMBP	15.38	15.5	35.2	9	3.158
sp|Q96EN8|MOCOS_HUMAN	MOCOS	Molybdenum cofactor sulfurase	9.82	10.21	22.1	6	3.169
sp|Q92851|CASPA_HUMAN	CASP10	Caspase-10	7.4	7.58	25.9	6	3.194
sp|Q7Z4L5|TT21B_HUMAN	TTC21B	Tetratricopeptide repeat protein 21B	4.53	6.77	17	6	3.194
sp|Q96AB6|NTAN1_HUMAN	NTAN1	Protein N-terminal asparagine amidohydrolase	4.03	4.54	25.8	3	3.195
sp|P21549|SPYA_HUMAN	AGXT	Serine--pyruvate aminotransferase	4.35	4.38	24.2	3	3.236

sp|Q01459|DIAC_HUMAN	CTBS	Di-N-acetylchitobiase	1.77	1.99	18.7	2	3.275
sp|Q9H582|ZN644_HUMAN	ZNF644	Zinc finger protein 644	2.56	3.08	15.2	5	3.295
sp|Q96FX7|TRM61_HUMAN	TRMT61A	tRNA (adenine(58)-N(1))-methyltransferase catalytic subunit TRMT61A	9	9.07	35	7	3.301
sp|Q03426|KIME_HUMAN	MVK	Mevalonate kinase	16.11	16.22	54.3	13	3.308
sp|Q9HBU6|EKI1_HUMAN	ETNK1	Ethanolamine kinase 1	4.01	4.02	23.5	2	3.343
sp|O60294|TYW4_HUMAN	LCMT2	tRNA wybutosine-synthesizing protein 4	4.03	4.13	12.5	3	3.357
sp|P09972|ALDOC_HUMAN	ALDOC	Fructose-bisphosphate aldolase C	19.47	33.97	62.6	58	3.466
sp|P47736|RPGP1_HUMAN	RAP1GAP	Rap1 GTPase-activating protein 1	2.38	3.32	13.3	3	3.479
sp|Q92503|S14L1_HUMAN	SEC14L1	SEC14-like protein 1	14.64	17.26	27.7	10	3.490
sp|P41440|S19A1_HUMAN	SLC19A1	Folate transporter 1	3.15	3.4	21.7	4	3.528
sp|Q6PUV4|CPLX2_HUMAN	CPLX2	Complexin-2	11.82	11.89	61.9	6	3.545
sp|P30711|GSTT1_HUMAN	GSTT1	Glutathione S-transferase theta-1	13.92	13.98	52.9	8	3.609
sp|Q53GG5|PDLI3_HUMAN	PDLIM3	PDZ and LIM domain protein 3	4.01	4.01	23.9	2	3.626
sp|Q9BQL6|FERM1_HUMAN	FERMT1	Fermitin family homolog 1	12.12	15.37	30.4	9	3.648
sp|Q6NUM9|RETST_HUMAN	RETSAT	All-trans-retinol 13,14-reductase	7.43	7.8	24.6	5	3.703
sp|Q9UKK3|PARP4_HUMAN	PARP4	Poly [ADP-ribose] polymerase 4	4.7	5.26	18.1	4	3.821
sp|P13645|K1C10_HUMAN	KRT10	Keratin, type I cytoskeletal 10	64.27	70.75	67	73	3.825
sp|Q9Y5N5|HEMK2_HUMAN	N6AMT1	HemK methyltransferase family member 2	2.4	2.44	28.5	5	3.919
sp|Q4AC94|C2CD3_HUMAN	C2CD3	C2 domain-containing protein 3	2.01	2.45	7.8	3	3.971
sp|Q99836|MYD88_HUMAN	MYD88	Myeloid differentiation primary response protein MyD88	8.07	8.2	43.2	7	4.033
sp|Q7Z6K3|PTAR1_HUMAN	PTAR1	Protein prenyltransferase alpha subunit repeat-containing protein 1	2.76	3	18.7	3	4.099
sp|Q8IWE2|NXP20_HUMAN	FAM114A1	Protein NOXP20	12.6	13.53	36.8	10	4.115
sp|Q13303|KCAB2_HUMAN	KCNAB2	Voltage-gated potassium channel subunit beta-2	7.7	7.91	30.8	6	4.163
sp|Q9H2F3|3BHS7_HUMAN	HSD3B7	3 beta-hydroxysteroid dehydrogenase type 7	12.08	12.2	26.8	7	4.184
sp|Q9UK39|NOCT_HUMAN	CCRN4L	Nocturnin	4.88	5.08	17.9	4	4.231
sp|P04264|K2C1_HUMAN	KRT1	Keratin, type II cytoskeletal 1	78.21	91.34	61.3	81	4.301
sp|Q9Y4B5|MTCL1_HUMAN	MTCL1	Microtubule cross-linking factor 1	2.65	2.94	12.8	3	4.389
sp|P01137|TGFB1_HUMAN	TGFB1	Transforming growth factor beta-1	4.01	4.03	18	2	4.405
sp|Q96FQ6|S10AG_HUMAN	S100A16	Protein S100-A16	5.91	6.08	61.2	3	4.425
sp|Q96HR9|REEP6_HUMAN	REEP6	Receptor expression-enhancing protein 6	5.38	5.48	32.6	5	4.725
sp|P11150|LIPC_HUMAN	LIPC	Hepatic triacylglycerol lipase	3.01	3.08	16.2	3	4.796
sp|Q15560|TCEA2_HUMAN	TCEA2	Transcription elongation factor A protein 2	1.85	5.04	39.5	3	4.868
sp|P52943|CRIP2_HUMAN	CRIP2	Cysteine-rich protein 2	6.77	7.55	49.5	7	5.023
sp|O95810|SDPR_HUMAN	SDPR	Serum deprivation-response protein	7.66	8.07	29.9	8	5.078
sp|P08572|CO4A2_HUMAN	COL4A2	Collagen alpha-2(IV) chain	2	2.11	6.5	2	5.224
sp|Q93097|WNT2B_HUMAN	WNT2B	Protein Wnt-2b	2	2	6.1	2	5.405
sp|P04233|HG2A_HUMAN	CD74	HLA class II histocompatibility antigen gamma chain	3.67	3.75	17.6	2	5.486
sp|Q14642|I5P1_HUMAN	INPP5A	Type I inositol 1,4,5-trisphosphate 5-phosphatase	2.54	2.59	20.2	2	5.932
sp|P17516|AK1C4_HUMAN	AKR1C4	Aldo-keto reductase family 1 member C4	2	30.36	72.1	34	5.950
sp|P31431|SDC4_HUMAN	SDC4	Syndecan-4	2.66	2.74	30.3	2	6.050
sp|Q99576|T22D3_HUMAN	TSC22D3	TSC22 domain family protein 3	2	4.4	55.2	3	6.463
sp|P53671|LIMK2_HUMAN	LIMK2	LIM domain kinase 2	5.44	5.67	28.4	3	6.636
sp|Q9C0D3|ZY11B_HUMAN	ZYG11B	Protein zyg-11 homolog B	6	6.05	15.7	5	7.292
sp|O15055|PER2_HUMAN	PER2	Period circadian protein homolog 2	1.38	1.55	7	4	8.655
sp|Q13322|GRB10_HUMAN	GRB10	Growth factor receptor-bound protein 10	3.85	3.93	9.9	3	10.956
sp|Q9HA65|TBC17_HUMAN	TBC1D17	TBC1 domain family member 17	2.23	2.59	11.6	3	30.138
