# Supplementary material for: The circular RNA CDR1as regulate cell proliferation via TMED2 and TMED10
Source: BMC Cancer. 2020 Apr 15;20:312. doi: 10.1186/s12885-020-06794-5 (PMC7160961; doi:10.1186/s12885-020-06794-5)
Supplement: Supplementary file 4 — Additional file 4. [file 12885_2020_6794_MOESM4_ESM.pdf]

Table S3 Complete list of the CRPs

Accession	Gene Name	Unused	Total	% Cov	Peptides(95%)	Ratio (siCDR1as/NC)
Q6ZS30	NBEAL1	2.05	4.97	11.3	5	0.16
Q96BR1	SGK3	4.03	4.32	21.2	3	0.22
P52926	HMGA2	10.02	10.31	60.6	6	0.25
P60059	SEC61G	4.01	4.01	48.5	4	0.26
P68431	HIST1H3A	35.62	37.49	80.9	139	0.27
Q08945	SSRP1	53.38	53.59	50.6	35	0.27
P69905	HBA1	4.96	5.1	43.7	6	0.30
Q53R41	FASTKD1	10.91	13.75	24.1	10	0.30
Q9Y5B9	SUPT16H	114.46	114.77	68.4	86	0.32
O75367	H2AFY	37.36	37.74	58.6	34	0.32
O43903	GAS2	8.08	9.71	37.7	6	0.33
Q71DI3	HIST2H3A	2.22	32.94	80.9	146	0.34
P51991	HNRNPA3	49.27	60.17	63	78	0.34
P54727	RAD23B	36.01	36.11	68.2	34	0.35
Q15043	SLC39A14	6.14	6.23	21.3	6	0.35
O95994	AGR2	3.8	3.89	24.6	2	0.35
O95747	OXSR1	26.34	28.12	52	16	0.36
P62805	HIST1H4A	33.01	33.08	87.4	140	0.36
Q96M27	PRRC1	15.17	15.33	33.5	10	0.36
P51149	RAB7A	33.21	33.47	92.3	34	0.36
O43493	TGOLN2	7.46	7.54	22.9	5	0.36
P11388	TOP2A	133.68	136.24	60.3	102	0.37
Q16695	HIST3H3	1.89	23.26	78.7	26	0.37
O43306	ADCY6	2.04	2.09	12.7	3	0.37
Q9UNW9	NOVA2	3.51	3.76	13.8	4	0.38
P17096	HMGA1	10.55	11.1	49.5	6	0.40
P84243	H3F3A	7.6	31.53	78.7	78	0.40
Q8TE76	MORC4	2.02	4.71	16.1	3	0.40
Q9BXS9	SLC26A6	4.03	4.09	16.3	3	0.40
P43307	SSR1	8.01	8.01	29.4	14	0.41
Q8IVP5	FUNDC1	2	2	18.7	2	0.41
Q6NXT1	ANKRD54	1.9	2.09	13	4	0.42
P62995	TRA2B	12.07	14.62	41.3	11	0.42
P09210	GSTA2	6.81	7.95	44.1	7	0.42
Q15166	PON3	5.53	5.7	47.5	5	0.42
P00450	CP	4.01	4.12	11	2	0.43
Q53GQ0	HSD17B12	24.86	24.98	69.6	19	0.43
P16949	STMN1	15.35	15.53	76.5	16	0.44
P19971	TYMP	6.55	6.6	20.1	3	0.44
Q9H832	UBE2Z	10.12	10.3	31.1	7	0.44
O76021	RSL1D1	64.21	65.26	72.6	52	0.44
Q9BZE2	PUS3	7.61	7.74	23.3	5	0.45
O00422	SAP18	21.18	21.41	73.9	12	0.45
Q96EB1	ELP4	4	4.01	17	2	0.46

Q15375	EPHA7	1.83	6.09	13.4	3	0.46
Q92982	NINJ1	2.02	2.02	17.1	2	0.46
Q2PPJ7	RALGAPA2	2.2	2.37	14.6	2	0.46
Q9H2V7	SPNS1	10	10	19.3	5	0.46
Q13185	CBX3	22.95	23.08	68.9	27	0.46
Q9H501	ESF1	41.33	42.55	42.9	25	0.47
P61026	RAB10	17.24	28.34	77	20	0.47
Q99805	TM9SF2	28.43	29.19	36.5	27	0.47
P05114	HMGN1	12.87	13	69	10	0.47
P30040	ERP29	27.12	29.79	82	33	0.47
P12956	XRCC6	107.37	108.98	75	137	0.48
P53999	SUB1	17.98	18.53	55.1	15	0.48
Q9UKM9	RALY	34.52	36.45	77.8	30	0.48
Q9H019	MTFR1L	6.04	6.44	27.1	5	0.48
P13010	XRCC5	102.39	102.61	85.8	127	0.48
P05204	HMGN2	11.18	11.44	64.4	9	0.48
Q9UGJ1	TUBGCP4	7.66	7.78	24.4	5	0.48
Q9P2E3	ZNFX1	2.95	3.49	12	4	0.49
Q8IZ21	PHACTR4	7.3	8.07	22.7	6	0.49
Q6P4F2	FDX1L	4.08	4.1	40.4	3	0.49
P11387	TOP1	78.81	79	63.5	51	0.49
Q9BPZ7	MAPKAP1	2.01	2.45	19.5	3	0.49
Q9BVQ7	SPATA5L1	13.69	13.96	33.6	10	0.49
Q8WUA2	PPIL4	26.85	26.99	44.5	15	0.49
P45973	CBX5	13.84	15.96	51.8	11	0.49
Q15629	TRAM1	7.8	8.01	22.5	6	0.49
P06753	TPM3	13.12	32.62	67	23	0.49
P04004	VTN	2.08	2.09	21.8	5	0.49
Q15363	TMED2	12.11	12.32	50.3	13	0.50
Q9BRJ2	MRPL45	23.63	23.77	58.2	14	0.50
Q9H9H4	VPS37B	8.01	8.03	38.3	4	0.50
P58876	HIST1H2BD	31.58	32.94	88.1	154	0.51
Q96GG9	DCUN1D1	8.29	8.39	41.3	5	0.51
P50552	VASP	28.49	28.57	55	20	0.51
Q5JU69	TOR2A	4	4.88	25.6	3	0.51
P16104	H2AFX	28.86	28.98	53.9	91	0.51
O60493	SNX3	7.84	11.7	67.3	7	0.51
P02655	APOC2	5.17	6.57	47.5	4	0.51
O00566	MPHOSPH10	28.88	30.39	40.8	20	0.51
Q9UMY1	NOL7	11.47	12.91	40.5	10	0.51
Q9H7B2	RPF2	15.27	19.95	62.4	13	0.51
Q9BQE5	APOL2	10.85	14.02	44.2	12	0.51
Q9UN36	NDRG2	7.4	7.55	23.5	5	0.52
Q9H583	HEATR1	100.56	104.79	44.5	65	0.52
Q96T23	RSF1	26.05	28.94	27	16	0.52
Q9Y2X3	NOP58	45.5	47.7	62.8	40	0.52
Q15061	WDR43	35.03	35.21	54.8	33	0.52
P62910	RPL32	13.21	13.97	63	16	0.52

Q9P0M6	H2AFY2	23.66	30.65	62.6	25	0.52
Q9BTM1	H2AFJ	4.15	22.97	52.7	83	0.52
Q92833	JARID2	2.02	2.26	13.2	3	0.52
Q15417	CNN3	19.61	24.07	61.4	22	0.52
Q8N257	HIST3H2BB	5.61	29.83	84.9	144	0.52
P81605	DCD	7.58	8.99	38.2	6	0.52
O95425	SVIL	7.03	8.29	13.6	7	0.52
O00560	SDCBP	21.61	21.74	78.5	19	0.52
Q9NQZ2	UTP3	14.18	14.52	34.5	9	0.52
Q9Y241	HIGD1A	3.76	4	53.8	4	0.52
Q8TED0	UTP15	35	35.16	54.8	22	0.53
O75414	NME6	3.5	3.65	30.7	5	0.53
Q92598	HSPH1	91.53	100.67	72.7	78	0.53
O43504	LAMTOR5	10.01	10.01	96.7	11	0.53
Q9Y2Q5	LAMTOR2	12.67	12.79	68	8	0.53
P18583	SON	67.9	68.89	32.1	44	0.53
Q8N584	TTC39C	1.63	2.04	17.3	2	0.53
P11908	PRPS2	7.26	20.79	49.1	16	0.53
P49755	TMED10	19.04	19.18	54.8	23	0.53
Q13671	RIN1	2.01	4.03	9.2	2	0.53
Q9NXJ5	PGPEP1	4	4	28.2	2	0.53
Q13206	DDX10	47.16	48.86	49.1	28	0.53
Q9BQ13	KCTD14	2.48	2.52	20.8	2	0.54
Q9NYV6	RRN3	6	6.07	26.1	3	0.54
Q969Z3	MARC2	6.44	9.03	34.9	7	0.54
P10768	ESD	33.54	33.61	85.5	27	0.54
Q9UK23	NAGPA	11	11.13	32.6	8	1.90
Q14999	CUL7	9.65	9.78	14.7	7	1.91
Q9Y6M5	SLC30A1	11.3	11.59	27.4	10	1.91
Q9NUN5	LMBRD1	2.84	2.91	12.2	4	1.92
Q8NG11	TSPAN14	4.7	4.76	23.3	3	1.92
Q92547	TOPBP1	21.33	22.8	27.7	15	1.93
P51790	CLCN3	4.77	5.45	18.5	4	1.93
Q15018	FAM175B	8.13	8.36	23.4	6	1.94
Q86XZ4	SPATS2	12.09	12.56	26.1	7	1.94
Q9BRQ8	AIFM2	16.01	16.05	45.3	10	1.94
Q9HCM3	KIAA1549	2	2.24	5.2	3	1.95
Q8IVH8	MAP4K3	5.22	7.26	15	8	1.95
Q969K3	RNF34	3.44	3.57	16.4	3	1.95
P07197	NEFM	2.01	4.47	22.3	9	1.95
O75896	TUSC2	3.53	3.69	48.2	2	1.95
Q4L180	FILIP1L	3.29	3.69	27.1	6	1.95
P13929	ENO3	8.35	32.82	77.9	105	1.95
Q96F07	CYFIP2	6.13	31.02	32.6	19	1.95
O75208	COQ9	8.72	9.32	25.5	7	1.95
O75764	TCEA3	8.53	8.82	37.4	7	1.96
Q92947	GCDH	25.78	26.84	70.1	23	1.96
P01130	LDLR	17.67	17.81	25.9	10	1.96

Q14353	GAMT	13.65	13.8	68.6	10	1.96
Q9NVM9	ASUN	11.37	11.82	31.4	9	1.97
Q7LG56	RRM2B	6.01	9.12	28.5	6	1.97
Q86TU7	SETD3	14.64	21.11	49.3	11	1.97
Q9NPD8	UBE2T	17.74	17.91	57.9	12	1.97
Q9H792	PEAK1	2.6	2.66	12	3	1.97
P02787	TF	61.87	66.09	75.6	44	1.97
P53801	PTTG1IP	4	4	13.9	2	1.97
P06396	GSN	21.47	21.92	33.6	13	1.98
Q86WQ0	NR2C2AP	4.15	4.18	49.6	6	1.98
Q9NSC2	SALL1	2.48	2.51	7.5	2	1.99
P47224	RABIF	4.61	4.78	36.6	4	1.99
P10398	ARAF	16.88	17.18	31.2	11	1.99
O60292	SIPA1L3	3.94	6.18	12.1	5	1.99
O75410	TACC1	19.33	20.17	35.3	13	2.00
Q9UK22	FBXO2	25.85	25.92	79.1	19	2.00
Q5R3I4	TTC38	29.98	30.45	68.2	27	2.00
Q13884	SNTB1	27.88	30.76	55.2	18	2.00
P61599	NAA20	11.59	11.73	62.9	7	2.00
Q96JJ3	ELMO2	20.77	21.15	39.3	14	2.00
Q7Z3C6	ATG9A	18.02	19.99	20.4	12	2.00
P13647	KRT5	15.78	37.38	37.8	26	2.00
Q13630	TSTA3	15.55	15.63	57.3	9	2.00
O95302	FKBP9	22.38	22.55	41.9	14	2.01
Q9UBD5	ORC3	20.19	20.59	28.3	11	2.01
Q92610	ZNF592	7.38	8.03	18.7	8	2.01
Q5T6V5	C9orf64	15.27	16.64	48.7	11	2.01
Q14161	GIT2	22.66	23.85	30.3	14	2.01
Q9UP95	SLC12A4	8.55	8.97	19.9	8	2.02
Q92796	DLG3	7.92	10.28	27.5	7	2.02
Q7Z589	EMSY	6.47	6.66	16.9	5	2.02
Q9NXS2	QPCTL	15.41	15.5	31.7	8	2.03
P42771	CDKN2A	7.62	7.71	70.5	6	2.03
Q86W56	PARG	9.06	9.29	24.1	5	2.03
Q96RL1	UIMC1	4.08	4.23	20.2	3	2.03
O95772	STARD3NL	4.49	4.6	32.9	3	2.03
P62942	FKBP1A	12.34	12.83	82.4	15	2.04
Q8TB03	CXorf38	2.23	2.27	27.6	3	2.05
O43739	CYTH3	2	2.03	13.3	2	2.05
Q96MN5	TCEANC2	2	2.05	25	2	2.05
P46108	CRK	16	18	45.7	10	2.06
Q5T3F8	TMEM63B	2.39	2.48	12.6	2	2.06
Q8N142	ADSSL1	2.01	6.05	22.1	3	2.07
Q8IWT6	LRRC8A	17.95	20.62	30.7	16	2.07
P16455	MGMT	4.36	4.45	36.2	5	2.08
P40818	USP8	19.7	21.69	33	13	2.08
Q9UGT4	SUSD2	2	2.04	7.8	2	2.08
Q9P2T1	GMPR2	26.76	27.11	73	16	2.09

P48728	AMT	2.24	2.36	21.8	3	2.09
Q9NWM8	FKBP14	11.09	11.21	41.7	8	2.10
Q8NEZ5	FBXO22	27.08	27.16	54.1	22	2.10
Q9UJM3	ERRFI1	2.19	2.32	15.6	3	2.12
Q9NZM3	ITSN2	16.41	17.75	24.6	11	2.12
P10588	NR2F6	4.79	4.85	19.3	3	2.12
P60709	ACTB	2	105.23	92.3	466	2.13
P63261	ACTG1	103.46	106.24	92.3	473	2.13
Q9P2C4	TMEM181	5.24	5.44	17.7	6	2.13
P11166	SLC2A1	13.55	13.85	18.1	12	2.13
Q8WXG6	MADD	6.27	6.45	15	4	2.14
P08727	KRT19	69.67	76.54	88.3	80	2.14
O60613	SEP15	8.55	8.66	54.9	6	2.14
P36915	GNL1	24.03	24.08	29.7	13	2.14
P80297	MT1X	5.22	5.29	34.4	3	2.15
Q8IZ52	CHPF	3.93	4.03	16	2	2.15
Q7Z739	YTHDF3	11.68	18.38	28.9	13	2.15
Q8TEB1	DCAF11	12.23	12.39	36.5	9	2.15
P50135	HNMT	8.93	9.2	44.2	11	2.16
Q02252	ALDH6A1	41.9	42.57	57.8	26	2.17
Q9ULC5	ACSL5	30.09	32.74	49.2	20	2.17
O95786	DDX58	3.41	3.99	19.8	4	2.17
P43007	SLC1A4	9.68	9.88	28.8	7	2.18
P35908	KRT2	57.79	69.24	72.1	62	2.18
Q9GZU2	PEG3	44.04	44.15	29.7	28	2.19
Q32P44	EML3	8.51	9.12	22.3	6	2.19
Q96GX2	ATXN7L3B	3.64	3.72	30.9	2	2.20
P05534	HLA-A	8.04	18.04	48.5	11	2.20
Q6XZF7	DNMBP	4.56	4.78	15.3	4	2.20
Q8TD43	TRPM4	2.18	4.3	11.5	3	2.21
Q9Y312	AAR2	11.56	11.78	45.1	9	2.22
P02652	APOA2	7.14	7.28	70	4	2.22
P21980	TGM2	27.59	28.62	47.9	22	2.22
Q63HN8	RNF213	76.27	81	25	45	2.23
O95954	FTCD	31.72	32.17	57.5	20	2.23
P02649	APOE	28.11	28.16	58.7	18	2.24
Q6P4R8	NFRKB	3.87	4.24	16.3	4	2.24
Q29963	HLA-C	8.56	14.85	42.4	8	2.26
Q5RI15	COX20	4.34	4.39	37.3	4	2.27
Q12802	AKAP13	12.72	13.29	15.7	9	2.27
Q9BRZ2	TRIM56	5.41	5.62	19.5	6	2.27
Q96CX2	KCTD12	11.27	11.39	47.1	7	2.27
Q86W74	ANKRD46	4.01	4.02	25.9	3	2.28
Q8N0V3	RBFA	13.97	14.06	44	8	2.28
Q9NVP2	ASF1B	6.13	8.06	52	5	2.28
Q9H6A9	PCNXL3	3.11	3.34	6.9	4	2.29
P59768	GNG2	1.54	3.75	36.6	2	2.29
Q14155	ARHGEF7	9.18	9.3	22.9	7	2.30

P13056	NR2C1	4.35	4.47	22.2	3	2.30
Q9P2J3	KLHL9	4.43	4.58	17.2	3	2.31
Q9Y5W7	SNX14	4.02	4.29	19.1	6	2.32
Q8NF91	SYNE1	5.31	10.57	18.7	17	2.33
Q96HN2	AHCYL2	3.49	29.25	37.5	21	2.34
Q9NS87	KIF15	27.07	29.92	36.7	16	2.35
P37059	HSD17B2	10.82	10.94	30.8	6	2.36
Q9C0B7	TANGO6	15.29	17.41	26.4	10	2.36
P28332	ADH6	13.47	13.61	29.6	7	2.38
Q5T0N5	FNBP1L	12.58	12.98	39.8	7	2.38
P27338	MAOB	23.64	24.95	36.9	14	2.38
Q2TB90	HKDC1	26.76	37.13	36.6	19	2.39
P46934	NEDD4	10.04	10.14	12.6	5	2.40
P61626	LYZ	13.23	13.36	59.5	12	2.41
Q96EK9	KTI12	10.82	10.89	42.9	6	2.42
Q9UL12	SARDH	25.97	26.14	39.7	13	2.42
Q6NUQ4	TMEM214	39.88	40.48	42.1	23	2.42
Q8NHP6	MOSPD2	8.36	8.65	32.1	6	2.42
O14730	RIOK3	3.02	3.14	18.7	2	2.43
Q9BXK1	KLF16	5.51	5.57	44.4	3	2.44
Q8NB46	ANKRD52	6.02	6.22	14.3	4	2.44
Q99988	GDF15	4.45	4.57	38	3	2.44
Q6Y288	B3GALTL	6.82	7.81	27.9	5	2.45
Q9H479	FN3K	16.01	16.06	43	8	2.46
Q2VPB7	AP5B1	8.83	8.92	18.9	7	2.48
Q13424	SNTA1	6.17	7.23	29.1	4	2.49
P07306	ASGR1	26.05	26.09	70.1	20	2.51
Q86YH6	PDSS2	9.36	9.54	32.8	5	2.52
Q15493	RGN	4.44	4.5	29.8	3	2.52
Q9NQB0	TCF7L2	1.37	1.5	15.4	2	2.53
P37235	HPCAL1	9.18	13.35	56	7	2.53
Q7Z6J9	TSEN54	3.07	3.29	13.3	3	2.54
O15427	SLC16A3	12.75	12.84	21.9	7	2.54
Q9Y508	RNF114	11.25	11.32	54	6	2.55
P16591	FER	8.71	11.41	34.1	9	2.57
Q96QR8	PURB	10.75	13.28	53.9	9	2.58
Q9BQC3	DPH2	10.24	10.39	33.1	7	2.60
P29279	CTGF	6.8	7.01	22.9	5	2.62
P31025	LCN1	3.33	3.45	29.6	2	2.62
Q01650	SLC7A5	8.31	8.4	19.5	6	2.66
Q6ZRI6	C15orf39	4.01	4.02	8.7	2	2.68
O60784	TOM1	29.03	29.23	57.9	21	2.69
Q9HD23	MRS2	12.1	12.26	42.7	7	2.70
O60281	ZNF292	3.05	4.76	11.1	9	2.71
Q6SPF0	SAMD1	11.65	11.81	29.7	8	2.72
Q9ULV0	MYO5B	2.91	11.1	21.7	8	2.76
Q9H082	RAB33B	4	7.85	25.8	6	2.77
Q96QZ7	MAGI1	13.77	15.22	17.6	9	2.77

Q9H9Q2	COPS7B	16.45	17.36	51.9	11	2.79
P05412	JUN	4	4.06	30.5	2	2.81
Q8IWV7	UBR1	9.75	9.94	13.8	7	2.82
Q13488	TCIRG1	5.17	5.41	13.7	5	2.82
Q8N128	FAM177A1	5.84	5.93	39.4	3	2.83
P02792	FTL	7.11	7.79	42.3	7	2.83
Q8NFG4	FLCN	4.66	4.92	19.3	4	2.85
Q13443	ADAM9	12.11	12.14	24.1	9	2.89
Q96LD4	TRIM47	14.12	14.4	34	9	2.89
Q9BPZ3	PAIP2	4.89	4.95	46.5	3	2.91
Q92618	ZNF516	2.36	2.42	9.6	2	2.98
Q15170	TCEAL1	4	4.01	29.3	3	2.98
P40121	CAPG	5.8	6.57	32.8	5	3.00
Q96RG2	PASK	2.28	2.33	11.7	2	3.02
Q9UQL6	HDAC5	2.14	2.25	13.3	3	3.02
Q8IW45	CARKD	8.45	8.57	45.8	5	3.12
O60825	PFKFB2	17.16	17.47	45.7	10	3.13
P02760	AMBP	15.38	15.5	35.2	9	3.16
Q96EN8	MOCOS	9.82	10.21	22.1	6	3.17
Q92851	CASP10	7.4	7.58	25.9	6	3.19
Q7Z4L5	TTC21B	4.53	6.77	17	6	3.19
Q96AB6	NTAN1	4.03	4.54	25.8	3	3.20
P21549	AGXT	4.35	4.38	24.2	3	3.24
Q01459	CTBS	1.77	1.99	18.7	2	3.27
Q9H582	ZNF644	2.56	3.08	15.2	5	3.29
Q96FX7	TRMT61A	9	9.07	35	7	3.30
Q03426	MVK	16.11	16.22	54.3	13	3.31
Q9HBU6	ETNK1	4.01	4.02	23.5	2	3.34
O60294	LCMT2	4.03	4.13	12.5	3	3.36
P09972	ALDOC	19.47	33.97	62.6	58	3.47
P47736	RAP1GAP	2.38	3.32	13.3	3	3.48
Q92503	SEC14L1	14.64	17.26	27.7	10	3.49
P41440	SLC19A1	3.15	3.4	21.7	4	3.53
Q6PUV4	CPLX2	11.82	11.89	61.9	6	3.55
P30711	GSTT1	13.92	13.98	52.9	8	3.61
Q53GG5	PDLIM3	4.01	4.01	23.9	2	3.63
Q9BQL6	FERMT1	12.12	15.37	30.4	9	3.65
Q6NUM9	RETSAT	7.43	7.8	24.6	5	3.70
Q9UKK3	PARP4	4.7	5.26	18.1	4	3.82
P13645	KRT10	64.27	70.75	67	73	3.83
Q9Y5N5	N6AMT1	2.4	2.44	28.5	5	3.92
Q4AC94	C2CD3	2.01	2.45	7.8	3	3.97
Q99836	MYD88	8.07	8.2	43.2	7	4.03
Q7Z6K3	PTAR1	2.76	3	18.7	3	4.10
Q8IWE2	FAM114A1	12.6	13.53	36.8	10	4.11
Q13303	KCNAB2	7.7	7.91	30.8	6	4.16
Q9H2F3	HSD3B7	12.08	12.2	26.8	7	4.18
Q9UK39	CCRN4L	4.88	5.08	17.9	4	4.23

P04264	KRT1	78.21	91.34	61.3	81	4.30
Q9Y4B5	MTCL1	2.65	2.94	12.8	3	4.39
P01137	TGFB1	4.01	4.03	18	2	4.41
Q96FQ6	S100A16	5.91	6.08	61.2	3	4.42
Q96HR9	REEP6	5.38	5.48	32.6	5	4.73
P11150	LIPC	3.01	3.08	16.2	3	4.80
Q15560	TCEA2	1.85	5.04	39.5	3	4.87
P52943	CRIP2	6.77	7.55	49.5	7	5.02
O95810	SDPR	7.66	8.07	29.9	8	5.08
P08572	COL4A2	2	2.11	6.5	2	5.22
Q93097	WNT2B	2	2	6.1	2	5.41
P04233	CD74	3.67	3.75	17.6	2	5.49
Q14642	INPP5A	2.54	2.59	20.2	2	5.93
P17516	AKR1C4	2	30.36	72.1	34	5.95
P31431	SDC4	2.66	2.74	30.3	2	6.05
Q99576	TSC22D3	2	4.4	55.2	3	6.46
P53671	LIMK2	5.44	5.67	28.4	3	6.64
Q9C0D3	ZYG11B	6	6.05	15.7	5	7.29
O15055	PER2	1.38	1.55	7	4	8.66
Q13322	GRB10	3.85	3.93	9.9	3	10.96
Q9HA65	TBC1D17	2.23	2.59	11.6	3	30.14
