# Supplementary material for: The circular RNA CDR1as regulate cell proliferation via TMED2 and TMED10
Source: BMC Cancer. 2020 Apr 15;20:312. doi: 10.1186/s12885-020-06794-5 (PMC7160961; doi:10.1186/s12885-020-06794-5)
Supplement: Supplementary file 5 — Additional file 5. [file 12885_2020_6794_MOESM5_ESM.pdf]

Table S4 Complete list of GO terms enriched in the CRPs

| GO-ID | Description | p-val | cluster freq |
|---|---|---|---|
| **GO_Biological Process** | | | |
| 71103 | DNA conformation change | 3.86E-10 | 18/289 6.2% |
| 34728 | nucleosome organization | 5.08E-10 | 15/289 5.1% |
| 6333 | chromatin assembly | 7.11E-10 | 17/289 5.8% |
| 6323 | DNA packaging | 1.88E-09 | 16/289 5.5% |
| 6334 | nucleosome assembly | 1.07E-08 | 13/289 4.4% |
| 31497 | chromatin assembly | 1.93E-08 | 13/289 4.4% |
| **Molecular Function** | | | |
| 5515 | protein binding | 1.42E-05 | 195/302 64.5% |
| 5200 | structural constituent of cytoskeleton | 9.63E-05 | 8/302 2.6% |
| 19899 | enzyme binding | 2.90E-04 | 26/302 8.6% |
| **Cellular Component** | | | |
| 785 | chromatin | 2.3738E-11 | 22/302 7.2% |
| 43228 | non-membrane-bounded organelle | 1.0988E-10 | 88/302 29.1% |
| 43232 | intracellular non-membrane-bounded organelle | 1.0988E-10 | 88/302 29.1% |
