# Supplementary material for: The circular RNA CDR1as regulate cell proliferation via TMED2 and TMED10
Source: BMC Cancer. 2020 Apr 15;20:312. doi: 10.1186/s12885-020-06794-5 (PMC7160961; doi:10.1186/s12885-020-06794-5)
Supplement: Supplementary file 6 — Additional file 6. [file 12885_2020_6794_MOESM6_ESM.pdf]

Table S5 PPI analysis of the CRPs

Node1	Node2	Node1 Accession	Node2 Accession	Score
SEP15	TMED10	9606.ENSP00000328729	9606.ENSP00000303145	0.421
ACSL5	LIPC	9606.ENSP00000348429	9606.ENSP00000299022	0.824
ACSL5	GCDH	9606.ENSP00000348429	9606.ENSP00000222214	0.738
ACSL5	COQ9	9606.ENSP00000348429	9606.ENSP00000262507	0.421
ACTB	ACTG1	9606.ENSP00000349960	9606.ENSP00000331514	0.997
ACTB	CYFIP2	9606.ENSP00000349960	9606.ENSP00000325817	0.911
ACTB	COPS7B	9606.ENSP00000349960	9606.ENSP00000272995	0.9
ACTB	VASP	9606.ENSP00000349960	9606.ENSP00000245932	0.854
ACTB	MYO5B	9606.ENSP00000349960	9606.ENSP00000285039	0.81
ACTB	SPATS2	9606.ENSP00000349960	9606.ENSP00000326841	0.599
ACTB	RAB7A	9606.ENSP00000349960	9606.ENSP00000265062	0.525
ACTB	TGFB1	9606.ENSP00000349960	9606.ENSP00000221930	0.508
ACTB	ENO3	9606.ENSP00000349960	9606.ENSP00000324105	0.49
ACTB	RAB10	9606.ENSP00000349960	9606.ENSP00000264710	0.48
ACTB	CAPG	9606.ENSP00000349960	9606.ENSP00000263867	0.459
ACTB	HIST1H2BD	9606.ENSP00000349960	9606.ENSP00000289316	0.445
ACTB	HKDC1	9606.ENSP00000349960	9606.ENSP00000346643	0.426
ACTG1	VASP	9606.ENSP00000331514	9606.ENSP00000245932	0.926
ACTG1	CYFIP2	9606.ENSP00000331514	9606.ENSP00000325817	0.908
ACTG1	MYO5B	9606.ENSP00000331514	9606.ENSP00000285039	0.81
ACTG1	ENO3	9606.ENSP00000331514	9606.ENSP00000324105	0.48
ACTG1	RAB10	9606.ENSP00000331514	9606.ENSP00000264710	0.46
ACTG1	RAB7A	9606.ENSP00000331514	9606.ENSP00000265062	0.443
ACTG1	CAPG	9606.ENSP00000331514	9606.ENSP00000263867	0.436
ACTG1	HIST1H2BD	9606.ENSP00000331514	9606.ENSP00000289316	0.423
ADH6	GSTT1	9606.ENSP00000378359	9606.ENSP00000248935	0.909
ADH6	MAOB	9606.ENSP00000378359	9606.ENSP00000367309	0.908
ADH6	RETSAT	9606.ENSP00000378359	9606.ENSP00000295802	0.905
ADH6	ESD	9606.ENSP00000378359	9606.ENSP00000367992	0.893
ADH6	ALDOC	9606.ENSP00000378359	9606.ENSP00000226253	0.452
ADH6	HDAC5	9606.ENSP00000378359	9606.ENSP00000225983	0.447
ADSSL1	FTCD	9606.ENSP00000333019	9606.ENSP00000291670	0.81
AGXT	AMT	9606.ENSP00000302620	9606.ENSP00000273588	0.937
AGXT	AMBP	9606.ENSP00000302620	9606.ENSP00000265132	0.454
AHCYL2	AMT	9606.ENSP00000315931	9606.ENSP00000273588	0.579
AKR1C4	RGN	9606.ENSP00000263126	9606.ENSP00000253303	0.493
ALDH6A1	ADH6	9606.ENSP00000450436	9606.ENSP00000378359	0.469
AMBP	ALDOC	9606.ENSP00000265132	9606.ENSP00000226253	0.575
AMBP	SLC1A4	9606.ENSP00000265132	9606.ENSP00000234256	0.446

AMBP	CP	9606.ENSP00000265132	9606.ENSP00000264613	0.421
AMBP	VTN	9606.ENSP00000265132	9606.ENSP00000226218	0.414
APOA2	APOE	9606.ENSP00000356969	9606.ENSP00000252486	0.98
APOA2	AMBP	9606.ENSP00000356969	9606.ENSP00000265132	0.86
APOA2	LIPC	9606.ENSP00000356969	9606.ENSP00000299022	0.505
APOA2	ASGR1	9606.ENSP00000356969	9606.ENSP00000269299	0.463
APOA2	CP	9606.ENSP00000356969	9606.ENSP00000264613	0.432
APOC2	APOE	9606.ENSP00000466775	9606.ENSP00000252486	0.928
APOC2	APOA2	9606.ENSP00000466775	9606.ENSP00000356969	0.883
APOC2	LDLR	9606.ENSP00000466775	9606.ENSP00000454071	0.577
APOC2	LIPC	9606.ENSP00000466775	9606.ENSP00000299022	0.449
APOE	TGFB1	9606.ENSP00000252486	9606.ENSP00000221930	0.691
APOE	NEFM	9606.ENSP00000252486	9606.ENSP00000221166	0.464
APOE	VTN	9606.ENSP00000252486	9606.ENSP00000226218	0.457
ARAF	LAMTOR2	9606.ENSP00000366244	9606.ENSP00000357288	0.9
ARAF	JUN	9606.ENSP00000366244	9606.ENSP00000360266	0.73
ARHGEF7	GIT2	9606.ENSP00000364893	9606.ENSP00000347464	0.993
ARHGEF7	CRK	9606.ENSP00000364893	9606.ENSP00000300574	0.447
ARHGEF7	JUN	9606.ENSP00000364893	9606.ENSP00000360266	0.44
ARHGEF7	OXSR1	9606.ENSP00000364893	9606.ENSP00000311713	0.427
ARHGEF7	DLG3	9606.ENSP00000364893	9606.ENSP00000363480	0.411
ASF1B	RRM2B	9606.ENSP00000263382	9606.ENSP00000251810	0.773
ASF1B	SUPT16H	9606.ENSP00000263382	9606.ENSP00000216297	0.661
ASGR1	AMBP	9606.ENSP00000269299	9606.ENSP00000265132	0.486
ATG9A	RAB7A	9606.ENSP00000355173	9606.ENSP00000265062	0.551
C9orf64	CARKD	9606.ENSP00000365522	9606.ENSP00000311984	0.417
CAPG	SNX3	9606.ENSP00000263867	9606.ENSP00000230085	0.776
CBX3	HIST1H2BD	9606.ENSP00000336687	9606.ENSP00000289316	0.9
CBX3	APOE	9606.ENSP00000336687	9606.ENSP00000252486	0.452
CBX3	HDAC5	9606.ENSP00000336687	9606.ENSP00000225983	0.408
CCRN4L	AMBP	9606.ENSP00000280614	9606.ENSP00000265132	0.424
CDKN2A	TOP1	9606.ENSP00000394932	9606.ENSP00000354522	0.798
CDKN2A	JUN	9606.ENSP00000394932	9606.ENSP00000360266	0.753
CDKN2A	MGMT	9606.ENSP00000394932	9606.ENSP00000302111	0.742
CDKN2A	HMGA2	9606.ENSP00000394932	9606.ENSP00000384026	0.586
CDKN2A	TGFB1	9606.ENSP00000394932	9606.ENSP00000221930	0.557
CDKN2A	H2AFX	9606.ENSP00000394932	9606.ENSP00000364310	0.534
CDKN2A	GDF15	9606.ENSP00000394932	9606.ENSP00000252809	0.533
CDKN2A	HIST1H4A	9606.ENSP00000394932	9606.ENSP00000352980	0.51
CDKN2A	ACTB	9606.ENSP00000394932	9606.ENSP00000349960	0.476
CDKN2A	ACTG1	9606.ENSP00000394932	9606.ENSP00000331514	0.469
CDKN2A	APOE	9606.ENSP00000394932	9606.ENSP00000252486	0.429

CDKN2A	KRT19	9606.ENSP00000394932	9606.ENSP00000355124	0.417
CDKN2A	TF	9606.ENSP00000394932	9606.ENSP00000385834	0.408
CNN3	TMEM181	9606.ENSP00000359225	9606.ENSP00000356057	0.8
COX20	COQ9	9606.ENSP00000406327	9606.ENSP00000262507	0.622
CRK	ELMO2	9606.ENSP00000300574	9606.ENSP00000290246	0.981
CRK	FER	9606.ENSP00000300574	9606.ENSP00000281092	0.523
CTGF	TGFB1	9606.ENSP00000356954	9606.ENSP00000221930	0.944
CTGF	ACTB	9606.ENSP00000356954	9606.ENSP00000349960	0.542
CUL7	FBXO2	9606.ENSP00000438788	9606.ENSP00000346240	0.931
CUL7	UBE2Z	9606.ENSP00000438788	9606.ENSP00000354201	0.919
CUL7	KLHL9	9606.ENSP00000438788	9606.ENSP00000351933	0.917
CUL7	FBXO22	9606.ENSP00000438788	9606.ENSP00000307833	0.911
CUL7	NEDD4	9606.ENSP00000438788	9606.ENSP00000345530	0.908
CUL7	UBR1	9606.ENSP00000438788	9606.ENSP00000290650	0.905
CUL7	RNF34	9606.ENSP00000438788	9606.ENSP00000376258	0.903
CUL7	RNF213	9606.ENSP00000438788	9606.ENSP00000324392	0.9
CUL7	RNF114	9606.ENSP00000438788	9606.ENSP00000244061	0.9
CYFIP2	ELMO2	9606.ENSP00000325817	9606.ENSP00000290246	0.933
CYFIP2	CRK	9606.ENSP00000325817	9606.ENSP00000300574	0.901
DDX10	NOP58	9606.ENSP00000314348	9606.ENSP00000264279	0.958
DDX10	ESF1	9606.ENSP00000314348	9606.ENSP00000202816	0.883
DDX10	UTP3	9606.ENSP00000314348	9606.ENSP00000254803	0.747
DDX10	UTP15	9606.ENSP00000314348	9606.ENSP00000296792	0.728
DDX10	MPHOSPH10	9606.ENSP00000314348	9606.ENSP00000244230	0.691
DDX10	RALY	9606.ENSP00000314348	9606.ENSP00000246194	0.424
DDX58	CASP10	9606.ENSP00000369213	9606.ENSP00000286186	0.912
DDX58	SDC4	9606.ENSP00000369213	9606.ENSP00000361818	0.471
DLG3	RRM2B	9606.ENSP00000363480	9606.ENSP00000251810	0.504
DLG3	LIMK2	9606.ENSP00000363480	9606.ENSP00000339916	0.429
DLG3	SARDH	9606.ENSP00000363480	9606.ENSP00000360938	0.42
DLG3	EPHA7	9606.ENSP00000363480	9606.ENSP00000358309	0.42
DLG3	PASK	9606.ENSP00000363480	9606.ENSP00000234040	0.42
DNMBP	VASP	9606.ENSP00000315659	9606.ENSP00000245932	0.404
ENO3	ALDOC	9606.ENSP00000324105	9606.ENSP00000226253	0.949
ENO3	DDX10	9606.ENSP00000324105	9606.ENSP00000314348	0.531
ENO3	AHCYL2	9606.ENSP00000324105	9606.ENSP00000315931	0.477
ENO3	NTAN1	9606.ENSP00000324105	9606.ENSP00000287706	0.437
ENO3	MVK	9606.ENSP00000324105	9606.ENSP00000228510	0.403
ERRFI1	LYZ	9606.ENSP00000366702	9606.ENSP00000261267	0.48
FBXO2	NEDD4	9606.ENSP00000346240	9606.ENSP00000345530	0.913
FBXO2	FBXO22	9606.ENSP00000346240	9606.ENSP00000307833	0.909
FBXO2	UBR1	9606.ENSP00000346240	9606.ENSP00000290650	0.906

FBXO2	RNF114	9606.ENSP00000346240	9606.ENSP00000244061	0.9
FBXO2	RNF213	9606.ENSP00000346240	9606.ENSP00000324392	0.9
FBXO22	RNF114	9606.ENSP00000307833	9606.ENSP00000244061	0.901
FBXO22	UBR1	9606.ENSP00000307833	9606.ENSP00000290650	0.9
FDX1L	HSPH1	9606.ENSP00000377311	9606.ENSP00000318687	0.562
FER	AMBP	9606.ENSP00000281092	9606.ENSP00000265132	0.447
FKBP1A	TGFB1	9606.ENSP00000371138	9606.ENSP00000221930	0.949
FKBP1A	ENO3	9606.ENSP00000371138	9606.ENSP00000324105	0.802
FKBP1A	GSTT1	9606.ENSP00000371138	9606.ENSP00000248935	0.439
FN3K	LYZ	9606.ENSP00000300784	9606.ENSP00000261267	0.756
FTCD	AMT	9606.ENSP00000291670	9606.ENSP00000273588	0.913
FTL	CP	9606.ENSP00000366525	9606.ENSP00000264613	0.528
GAMT	RPF2	9606.ENSP00000403536	9606.ENSP00000402338	0.725
GAMT	WDR43	9606.ENSP00000403536	9606.ENSP00000384302	0.595
GAMT	SETD3	9606.ENSP00000403536	9606.ENSP00000327436	0.427
GMPR2	ADSSL1	9606.ENSP00000392859	9606.ENSP00000333019	0.956
GNG2	HDAC5	9606.ENSP00000334448	9606.ENSP00000225983	0.9
GNG2	ADCY6	9606.ENSP00000334448	9606.ENSP00000311405	0.9
GNL1	UTP15	9606.ENSP00000365806	9606.ENSP00000296792	0.783
GNL1	NOP58	9606.ENSP00000365806	9606.ENSP00000264279	0.705
GNL1	HEATR1	9606.ENSP00000365806	9606.ENSP00000355541	0.684
GNL1	UTP3	9606.ENSP00000365806	9606.ENSP00000254803	0.684
GNL1	ESF1	9606.ENSP00000365806	9606.ENSP00000202816	0.521
GNL1	MPHOSPH10	9606.ENSP00000365806	9606.ENSP00000244230	0.463
GNL1	DDX10	9606.ENSP00000365806	9606.ENSP00000314348	0.4
GRB10	NEDD4	9606.ENSP00000381793	9606.ENSP00000345530	0.955
GRB10	VASP	9606.ENSP00000381793	9606.ENSP00000245932	0.445
GRB10	PEG3	9606.ENSP00000381793	9606.ENSP00000326581	0.442
GSN	ACTG1	9606.ENSP00000362924	9606.ENSP00000331514	0.751
GSN	ACTB	9606.ENSP00000362924	9606.ENSP00000349960	0.713
GSN	VASP	9606.ENSP00000362924	9606.ENSP00000245932	0.614
GSN	SVIL	9606.ENSP00000362924	9606.ENSP00000348128	0.613
GSN	LIMK2	9606.ENSP00000362924	9606.ENSP00000339916	0.41
GSTA2	GSTT1	9606.ENSP00000420168	9606.ENSP00000248935	0.935
GSTA2	JUN	9606.ENSP00000420168	9606.ENSP00000360266	0.92
GSTA2	ADH6	9606.ENSP00000420168	9606.ENSP00000378359	0.911
H2AFJ	HIST1H2BD	9606.ENSP00000373730	9606.ENSP00000289316	0.996
H2AFJ	HIST3H3	9606.ENSP00000373730	9606.ENSP00000355657	0.984
H2AFJ	H2AFX	9606.ENSP00000373730	9606.ENSP00000364310	0.95
H2AFJ	CBX3	9606.ENSP00000373730	9606.ENSP00000336687	0.904
H2AFJ	SYNE1	9606.ENSP00000373730	9606.ENSP00000356224	0.902
H2AFJ	SAP18	9606.ENSP00000373730	9606.ENSP00000371973	0.902

H2AFJ	JARID2	9606.ENSP00000373730	9606.ENSP00000341280	0.902
H2AFJ	JUN	9606.ENSP00000373730	9606.ENSP00000360266	0.902
H2AFJ	RSF1	9606.ENSP00000373730	9606.ENSP00000311513	0.901
H2AFJ	H3F3A	9606.ENSP00000373730	9606.ENSP00000355778	0.852
H2AFJ	HIST1H3A	9606.ENSP00000373730	9606.ENSP00000350275	0.852
H2AFJ	HDAC5	9606.ENSP00000373730	9606.ENSP00000225983	0.847
H2AFJ	HIST1H4A	9606.ENSP00000373730	9606.ENSP00000352980	0.738
H2AFJ	SUPT16H	9606.ENSP00000373730	9606.ENSP00000216297	0.464
H2AFJ	ASF1B	9606.ENSP00000373730	9606.ENSP00000263382	0.425
H2AFX	HIST1H2BD	9606.ENSP00000364310	9606.ENSP00000289316	0.996
H2AFX	HIST3H3	9606.ENSP00000364310	9606.ENSP00000355657	0.99
H2AFX	XRCC6	9606.ENSP00000364310	9606.ENSP00000352257	0.983
H2AFX	JUN	9606.ENSP00000364310	9606.ENSP00000360266	0.933
H2AFX	CBX3	9606.ENSP00000364310	9606.ENSP00000336687	0.919
H2AFX	HIST1H3A	9606.ENSP00000364310	9606.ENSP00000350275	0.913
H2AFX	HIST1H4A	9606.ENSP00000364310	9606.ENSP00000352980	0.909
H2AFX	RSF1	9606.ENSP00000364310	9606.ENSP00000311513	0.905
H2AFX	JARID2	9606.ENSP00000364310	9606.ENSP00000341280	0.902
H2AFX	SYNE1	9606.ENSP00000364310	9606.ENSP00000356224	0.902
H2AFX	H3F3A	9606.ENSP00000364310	9606.ENSP00000355778	0.879
H2AFX	HDAC5	9606.ENSP00000364310	9606.ENSP00000225983	0.848
H2AFX	SUPT16H	9606.ENSP00000364310	9606.ENSP00000216297	0.703
H2AFX	TOP1	9606.ENSP00000364310	9606.ENSP00000354522	0.678
H2AFX	TOPBP1	9606.ENSP00000364310	9606.ENSP00000260810	0.669
H2AFX	SSRP1	9606.ENSP00000364310	9606.ENSP00000278412	0.614
H2AFX	ASF1B	9606.ENSP00000364310	9606.ENSP00000263382	0.532
H2AFX	CBX5	9606.ENSP00000364310	9606.ENSP00000209875	0.43
H2AFY	HIST1H2BD	9606.ENSP00000423563	9606.ENSP00000289316	0.995
H2AFY	HIST3H2BB	9606.ENSP00000423563	9606.ENSP00000375736	0.995
H2AFY	HIST1H3A	9606.ENSP00000423563	9606.ENSP00000350275	0.921
H2AFY	H3F3A	9606.ENSP00000423563	9606.ENSP00000355778	0.92
H2AFY	HIST3H3	9606.ENSP00000423563	9606.ENSP00000355657	0.907
H2AFY	HIST1H4A	9606.ENSP00000423563	9606.ENSP00000352980	0.88
H2AFY	HDAC5	9606.ENSP00000423563	9606.ENSP00000225983	0.873
H2AFY	HIST2H3A	9606.ENSP00000423563	9606.ENSP00000385479	0.861
H2AFY	CBX3	9606.ENSP00000423563	9606.ENSP00000336687	0.57
H2AFY	CBX5	9606.ENSP00000423563	9606.ENSP00000209875	0.54
H2AFY	TOP2A	9606.ENSP00000423563	9606.ENSP00000411532	0.516
H2AFY	H2AFY2	9606.ENSP00000423563	9606.ENSP00000362352	0.512
H2AFY	XRCC5	9606.ENSP00000423563	9606.ENSP00000375977	0.476
H2AFY	SUPT16H	9606.ENSP00000423563	9606.ENSP00000216297	0.463
H2AFY	ASF1B	9606.ENSP00000423563	9606.ENSP00000263382	0.453

H2AFY	TOP1	9606.ENSP00000423563	9606.ENSP00000354522	0.449
H2AFY2	HIST1H2BD	9606.ENSP00000362352	9606.ENSP00000289316	0.995
H2AFY2	HIST1H3A	9606.ENSP00000362352	9606.ENSP00000350275	0.875
H2AFY2	H3F3A	9606.ENSP00000362352	9606.ENSP00000355778	0.875
H2AFY2	HDAC5	9606.ENSP00000362352	9606.ENSP00000225983	0.873
H2AFY2	HIST3H3	9606.ENSP00000362352	9606.ENSP00000355657	0.865
H2AFY2	HIST1H4A	9606.ENSP00000362352	9606.ENSP00000352980	0.772
H2AFY2	CBX5	9606.ENSP00000362352	9606.ENSP00000209875	0.52
H2AFY2	ASF1B	9606.ENSP00000362352	9606.ENSP00000263382	0.441
H2AFY2	SUPT16H	9606.ENSP00000362352	9606.ENSP00000216297	0.401
H3F3A	ASF1B	9606.ENSP00000355778	9606.ENSP00000263382	0.977
H3F3A	HIST1H2BD	9606.ENSP00000355778	9606.ENSP00000289316	0.945
H3F3A	HIST1H4A	9606.ENSP00000355778	9606.ENSP00000352980	0.902
H3F3A	HIST3H3	9606.ENSP00000355778	9606.ENSP00000355657	0.9
H3F3A	HIST1H3A	9606.ENSP00000355778	9606.ENSP00000350275	0.9
H3F3A	CBX3	9606.ENSP00000355778	9606.ENSP00000336687	0.763
H3F3A	HDAC5	9606.ENSP00000355778	9606.ENSP00000225983	0.734
H3F3A	SSRP1	9606.ENSP00000355778	9606.ENSP00000278412	0.669
H3F3A	SUPT16H	9606.ENSP00000355778	9606.ENSP00000216297	0.639
H3F3A	CBX5	9606.ENSP00000355778	9606.ENSP00000209875	0.572
H3F3A	ACTB	9606.ENSP00000355778	9606.ENSP00000349960	0.544
H3F3A	XRCC6	9606.ENSP00000355778	9606.ENSP00000352257	0.528
H3F3A	ACTG1	9606.ENSP00000355778	9606.ENSP00000331514	0.479
HBA1	TGFB1	9606.ENSP00000322421	9606.ENSP00000221930	0.501
HBA1	TYMP	9606.ENSP00000322421	9606.ENSP00000252029	0.426
HDAC5	CBX5	9606.ENSP00000225983	9606.ENSP00000209875	0.698
HEATR1	UTP15	9606.ENSP00000355541	9606.ENSP00000296792	0.999
HEATR1	NOP58	9606.ENSP00000355541	9606.ENSP00000264279	0.998
HEATR1	MPHOSPH10	9606.ENSP00000355541	9606.ENSP00000244230	0.996
HEATR1	UTP3	9606.ENSP00000355541	9606.ENSP00000254803	0.962
HEATR1	RIOK3	9606.ENSP00000355541	9606.ENSP00000341874	0.905
HEATR1	DDX10	9606.ENSP00000355541	9606.ENSP00000314348	0.616
HEATR1	ESF1	9606.ENSP00000355541	9606.ENSP00000202816	0.594
HIGD1A	SUB1	9606.ENSP00000398064	9606.ENSP00000265073	0.425
HIST1H2BD	HDAC5	9606.ENSP00000289316	9606.ENSP00000225983	0.871
HIST1H3A	HIST1H2BD	9606.ENSP00000350275	9606.ENSP00000289316	0.903
HIST1H3A	ASF1B	9606.ENSP00000350275	9606.ENSP00000263382	0.89
HIST1H3A	HDAC5	9606.ENSP00000350275	9606.ENSP00000225983	0.789
HIST1H3A	SSRP1	9606.ENSP00000350275	9606.ENSP00000278412	0.698
HIST1H3A	CBX3	9606.ENSP00000350275	9606.ENSP00000336687	0.687
HIST1H3A	SUPT16H	9606.ENSP00000350275	9606.ENSP00000216297	0.532
HIST1H3A	ACTB	9606.ENSP00000350275	9606.ENSP00000349960	0.479

HIST1H3A	ACTG1	9606.ENSP00000350275	9606.ENSP00000331514	0.471
HIST1H3A	CBX5	9606.ENSP00000350275	9606.ENSP00000209875	0.401
HIST1H4A	HIST1H3A	9606.ENSP00000352980	9606.ENSP00000350275	0.908
HIST1H4A	ASF1B	9606.ENSP00000352980	9606.ENSP00000263382	0.837
HIST1H4A	HDAC5	9606.ENSP00000352980	9606.ENSP00000225983	0.813
HIST1H4A	HIST1H2BD	9606.ENSP00000352980	9606.ENSP00000289316	0.763
HIST1H4A	CBX3	9606.ENSP00000352980	9606.ENSP00000336687	0.671
HIST1H4A	SSRP1	9606.ENSP00000352980	9606.ENSP00000278412	0.658
HIST1H4A	ACTB	9606.ENSP00000352980	9606.ENSP00000349960	0.589
HIST1H4A	SUPT16H	9606.ENSP00000352980	9606.ENSP00000216297	0.589
HIST1H4A	ACTG1	9606.ENSP00000352980	9606.ENSP00000331514	0.476
HIST1H4A	XRCC6	9606.ENSP00000352980	9606.ENSP00000352257	0.471
HIST1H4A	HNMT	9606.ENSP00000352980	9606.ENSP00000280097	0.449
HIST2H3A	H2AFX	9606.ENSP00000385479	9606.ENSP00000364310	0.989
HIST2H3A	H2AFJ	9606.ENSP00000385479	9606.ENSP00000373730	0.984
HIST2H3A	HIST3H2BB	9606.ENSP00000385479	9606.ENSP00000375736	0.98
HIST2H3A	HIST1H2BD	9606.ENSP00000385479	9606.ENSP00000289316	0.977
HIST2H3A	CBX3	9606.ENSP00000385479	9606.ENSP00000336687	0.966
HIST2H3A	CBX5	9606.ENSP00000385479	9606.ENSP00000209875	0.952
HIST2H3A	SAP18	9606.ENSP00000385479	9606.ENSP00000371973	0.922
HIST2H3A	SETD3	9606.ENSP00000385479	9606.ENSP00000327436	0.913
HIST2H3A	JUN	9606.ENSP00000385479	9606.ENSP00000360266	0.911
HIST2H3A	ELP4	9606.ENSP00000385479	9606.ENSP00000298937	0.908
HIST2H3A	JARID2	9606.ENSP00000385479	9606.ENSP00000341280	0.902
HIST2H3A	HIST3H3	9606.ENSP00000385479	9606.ENSP00000355657	0.9
HIST2H3A	H2AFY2	9606.ENSP00000385479	9606.ENSP00000362352	0.864
HIST2H3A	HIST1H4A	9606.ENSP00000385479	9606.ENSP00000352980	0.851
HIST2H3A	ASF1B	9606.ENSP00000385479	9606.ENSP00000263382	0.802
HIST2H3A	HDAC5	9606.ENSP00000385479	9606.ENSP00000225983	0.684
HIST2H3A	SSRP1	9606.ENSP00000385479	9606.ENSP00000278412	0.525
HIST2H3A	ACTB	9606.ENSP00000385479	9606.ENSP00000349960	0.474
HIST2H3A	ACTG1	9606.ENSP00000385479	9606.ENSP00000331514	0.471
HIST2H3A	SUPT16H	9606.ENSP00000385479	9606.ENSP00000216297	0.45
HIST2H3A	HMGA1	9606.ENSP00000385479	9606.ENSP00000308227	0.414
HIST3H2BB	H2AFX	9606.ENSP00000375736	9606.ENSP00000364310	0.997
HIST3H2BB	H2AFJ	9606.ENSP00000375736	9606.ENSP00000373730	0.997
HIST3H2BB	H2AFY2	9606.ENSP00000375736	9606.ENSP00000362352	0.995
HIST3H2BB	HIST3H3	9606.ENSP00000375736	9606.ENSP00000355657	0.977
HIST3H2BB	HIST1H2BD	9606.ENSP00000375736	9606.ENSP00000289316	0.969
HIST3H2BB	HIST1H3A	9606.ENSP00000375736	9606.ENSP00000350275	0.914
HIST3H2BB	XRCC6	9606.ENSP00000375736	9606.ENSP00000352257	0.907
HIST3H2BB	JUN	9606.ENSP00000375736	9606.ENSP00000360266	0.902

HIST3H2BB	UIMC1	9606.ENSP00000375736	9606.ENSP00000366434	0.901
HIST3H2BB	SYNE1	9606.ENSP00000375736	9606.ENSP00000356224	0.901
HIST3H2BB	RSF1	9606.ENSP00000375736	9606.ENSP00000311513	0.9
HIST3H2BB	CBX3	9606.ENSP00000375736	9606.ENSP00000336687	0.9
HIST3H2BB	JARID2	9606.ENSP00000375736	9606.ENSP00000341280	0.9
HIST3H2BB	SAP18	9606.ENSP00000375736	9606.ENSP00000371973	0.9
HIST3H2BB	H3F3A	9606.ENSP00000375736	9606.ENSP00000355778	0.898
HIST3H2BB	HDAC5	9606.ENSP00000375736	9606.ENSP00000225983	0.871
HIST3H2BB	HIST1H4A	9606.ENSP00000375736	9606.ENSP00000352980	0.753
HIST3H2BB	ACTB	9606.ENSP00000375736	9606.ENSP00000349960	0.423
HIST3H2BB	ACTG1	9606.ENSP00000375736	9606.ENSP00000331514	0.423
HIST3H3	HIST1H2BD	9606.ENSP00000355657	9606.ENSP00000289316	0.977
HIST3H3	XRCC6	9606.ENSP00000355657	9606.ENSP00000352257	0.935
HIST3H3	HIST1H3A	9606.ENSP00000355657	9606.ENSP00000350275	0.9
HIST3H3	ASF1B	9606.ENSP00000355657	9606.ENSP00000263382	0.868
HIST3H3	HIST1H4A	9606.ENSP00000355657	9606.ENSP00000352980	0.816
HIST3H3	HDAC5	9606.ENSP00000355657	9606.ENSP00000225983	0.716
HIST3H3	CBX3	9606.ENSP00000355657	9606.ENSP00000336687	0.674
HIST3H3	SSRP1	9606.ENSP00000355657	9606.ENSP00000278412	0.516
HIST3H3	ACTB	9606.ENSP00000355657	9606.ENSP00000349960	0.471
HIST3H3	ACTG1	9606.ENSP00000355657	9606.ENSP00000331514	0.471
HIST3H3	SUPT16H	9606.ENSP00000355657	9606.ENSP00000216297	0.469
HKDC1	ALDOC	9606.ENSP00000346643	9606.ENSP00000226253	0.882
HKDC1	ENO3	9606.ENSP00000346643	9606.ENSP00000324105	0.53
HKDC1	AHCYL2	9606.ENSP00000346643	9606.ENSP00000315931	0.429
HKDC1	ACTG1	9606.ENSP00000346643	9606.ENSP00000331514	0.426
HLA-A	HLA-C	9606.ENSP00000366005	9606.ENSP00000365402	0.94
HLA-A	JUN	9606.ENSP00000366005	9606.ENSP00000360266	0.919
HLA-A	CD74	9606.ENSP00000366005	9606.ENSP00000009530	0.447
HLA-A	HBA1	9606.ENSP00000366005	9606.ENSP00000322421	0.407
HLA-C	RNF114	9606.ENSP00000365402	9606.ENSP00000244061	0.424
HLA-C	CD74	9606.ENSP00000365402	9606.ENSP00000009530	0.424
HMGA1	MVK	9606.ENSP00000308227	9606.ENSP00000228510	0.504
HMGA2	HMGA1	9606.ENSP00000384026	9606.ENSP00000308227	0.952
HMGA2	H2AFX	9606.ENSP00000384026	9606.ENSP00000364310	0.475
HMGA2	AMBP	9606.ENSP00000384026	9606.ENSP00000265132	0.419
HMGN1	HMGN2	9606.ENSP00000370125	9606.ENSP00000355228	0.933
HMGN1	H2AFX	9606.ENSP00000370125	9606.ENSP00000364310	0.502
HNRNPA3	RAB7A	9606.ENSP00000376309	9606.ENSP00000265062	0.441
HNRNPA3	DDX10	9606.ENSP00000376309	9606.ENSP00000314348	0.424
HNRNPA3	RAB10	9606.ENSP00000376309	9606.ENSP00000264710	0.418
HNRNPA3	XRCC6	9606.ENSP00000376309	9606.ENSP00000352257	0.417

HSD17B12	HSD17B2	9606.ENSP00000278353	9606.ENSP00000199936	0.833
HSD17B12	TMED2	9606.ENSP00000278353	9606.ENSP00000262225	0.425
HSD3B7	GCDH	9606.ENSP00000297679	9606.ENSP00000222214	0.478
HSD3B7	AKR1C4	9606.ENSP00000297679	9606.ENSP00000263126	0.417
ITSN2	FNBP1L	9606.ENSP00000347244	9606.ENSP00000271234	0.9
ITSN2	UBR1	9606.ENSP00000347244	9606.ENSP00000290650	0.469
JARID2	HIST1H2BD	9606.ENSP00000341280	9606.ENSP00000289316	0.9
JUN	TGFB1	9606.ENSP00000360266	9606.ENSP00000221930	0.965
JUN	ACTB	9606.ENSP00000360266	9606.ENSP00000349960	0.953
JUN	KRT5	9606.ENSP00000360266	9606.ENSP00000252242	0.925
JUN	APOA2	9606.ENSP00000360266	9606.ENSP00000356969	0.908
JUN	HIST1H2BD	9606.ENSP00000360266	9606.ENSP00000289316	0.905
JUN	TSC22D3	9606.ENSP00000360266	9606.ENSP00000314655	0.712
JUN	CTGF	9606.ENSP00000360266	9606.ENSP00000356954	0.704
JUN	TOP1	9606.ENSP00000360266	9606.ENSP00000354522	0.61
JUN	KRT10	9606.ENSP00000360266	9606.ENSP00000269576	0.557
JUN	APOE	9606.ENSP00000360266	9606.ENSP00000252486	0.528
JUN	NEDD4	9606.ENSP00000360266	9606.ENSP00000345530	0.519
JUN	HIST1H4A	9606.ENSP00000360266	9606.ENSP00000352980	0.477
JUN	HMGA1	9606.ENSP00000360266	9606.ENSP00000308227	0.474
JUN	LYZ	9606.ENSP00000360266	9606.ENSP00000261267	0.456
JUN	VTN	9606.ENSP00000360266	9606.ENSP00000226218	0.415
JUN	AIFM2	9606.ENSP00000360266	9606.ENSP00000312370	0.413
JUN	HBA1	9606.ENSP00000360266	9606.ENSP00000322421	0.405
KCNAB2	AKR1C4	9606.ENSP00000367323	9606.ENSP00000263126	0.441
KIF15	RAB7A	9606.ENSP00000324020	9606.ENSP00000265062	0.904
KIF15	TMED2	9606.ENSP00000324020	9606.ENSP00000262225	0.9
KIF15	TMED10	9606.ENSP00000324020	9606.ENSP00000303145	0.9
KIF15	ASF1B	9606.ENSP00000324020	9606.ENSP00000263382	0.484
KLHL9	UBR1	9606.ENSP00000351933	9606.ENSP00000290650	0.902
KLHL9	NEDD4	9606.ENSP00000351933	9606.ENSP00000345530	0.901
KLHL9	RNF114	9606.ENSP00000351933	9606.ENSP00000244061	0.9
KLHL9	FBXO22	9606.ENSP00000351933	9606.ENSP00000307833	0.9
KLHL9	RNF213	9606.ENSP00000351933	9606.ENSP00000324392	0.9
KLHL9	FBXO2	9606.ENSP00000351933	9606.ENSP00000346240	0.9
KRT1	KRT5	9606.ENSP00000252244	9606.ENSP00000252242	0.79
KRT10	KRT1	9606.ENSP00000269576	9606.ENSP00000252244	0.996
KRT10	KRT5	9606.ENSP00000269576	9606.ENSP00000252242	0.83
KRT19	HBA1	9606.ENSP00000355124	9606.ENSP00000322421	0.487
KRT19	KRT5	9606.ENSP00000355124	9606.ENSP00000252242	0.429
KRT2	KRT10	9606.ENSP00000310861	9606.ENSP00000269576	0.865
KRT2	KRT1	9606.ENSP00000310861	9606.ENSP00000252244	0.823

KRT2	KRT5	9606.ENSP00000310861	9606.ENSP00000252242	0.79
KTI12	ELP4	9606.ENSP00000360676	9606.ENSP00000298937	0.696
LAMTOR2	LAMTOR5	9606.ENSP00000357288	9606.ENSP00000256644	0.941
LCN1	LYZ	9606.ENSP00000263598	9606.ENSP00000261267	0.579
LDLR	APOE	9606.ENSP00000454071	9606.ENSP00000252486	0.997
LDLR	TF	9606.ENSP00000454071	9606.ENSP00000385834	0.948
LDLR	LIPC	9606.ENSP00000454071	9606.ENSP00000299022	0.945
LDLR	APOA2	9606.ENSP00000454071	9606.ENSP00000356969	0.941
LDLR	TGOLN2	9606.ENSP00000454071	9606.ENSP00000386443	0.924
LDLR	ITSN2	9606.ENSP00000454071	9606.ENSP00000347244	0.901
LDLR	FNBP1L	9606.ENSP00000454071	9606.ENSP00000271234	0.9
LDLR	JUN	9606.ENSP00000454071	9606.ENSP00000360266	0.659
LDLR	PON3	9606.ENSP00000454071	9606.ENSP00000265627	0.522
LDLR	HMGA1	9606.ENSP00000454071	9606.ENSP00000308227	0.439
LDLR	VTN	9606.ENSP00000454071	9606.ENSP00000226218	0.408
LDLR	TGFB1	9606.ENSP00000454071	9606.ENSP00000221930	0.4
LIPC	APOE	9606.ENSP00000299022	9606.ENSP00000252486	0.956
LYZ	APOE	9606.ENSP00000261267	9606.ENSP00000252486	0.446
MAOB	HNMT	9606.ENSP00000367309	9606.ENSP00000280097	0.915
MAPKAP1	SGK3	9606.ENSP00000265960	9606.ENSP00000262211	0.474
MGMT	ERP29	9606.ENSP00000302111	9606.ENSP00000261735	0.434
MOSPD2	CXorf38	9606.ENSP00000369860	9606.ENSP00000330488	0.435
MPHOSPH1(	ESF1	9606.ENSP00000244230	9606.ENSP00000202816	0.99
MT1X	SLC30A1	9606.ENSP00000377995	9606.ENSP00000355968	0.405
MYD88	DDX58	9606.ENSP00000401399	9606.ENSP00000369213	0.747
MYD88	JUN	9606.ENSP00000401399	9606.ENSP00000360266	0.597
MYD88	TGFB1	9606.ENSP00000401399	9606.ENSP00000221930	0.473
MYO5B	RAB10	9606.ENSP00000285039	9606.ENSP00000264710	0.577
NAGPA	AMBP	9606.ENSP00000310998	9606.ENSP00000265132	0.658
NAGPA	CCRN4L	9606.ENSP00000310998	9606.ENSP00000280614	0.481
NAGPA	FER	9606.ENSP00000310998	9606.ENSP00000281092	0.442
NEDD4	UBR1	9606.ENSP00000345530	9606.ENSP00000290650	0.931
NEDD4	RNF213	9606.ENSP00000345530	9606.ENSP00000324392	0.906
NEDD4	RNF114	9606.ENSP00000345530	9606.ENSP00000244061	0.902
NEDD4	FBXO22	9606.ENSP00000345530	9606.ENSP00000307833	0.9
NEDD4	USP8	9606.ENSP00000345530	9606.ENSP00000302239	0.583
NEDD4	SGK3	9606.ENSP00000345530	9606.ENSP00000262211	0.512
NEDD4	DCUN1D1	9606.ENSP00000345530	9606.ENSP00000292782	0.413
NFRKB	COPS7B	9606.ENSP00000436926	9606.ENSP00000272995	0.9
NFRKB	ACTB	9606.ENSP00000436926	9606.ENSP00000349960	0.9
NFRKB	RAD23B	9606.ENSP00000436926	9606.ENSP00000350708	0.9
NME6	RRM2B	9606.ENSP00000416658	9606.ENSP00000251810	0.948

NME6	ADCY6	9606.ENSP00000416658	9606.ENSP00000311405	0.905
NME6	DLG3	9606.ENSP00000416658	9606.ENSP00000363480	0.507
NOL7	HBA1	9606.ENSP00000405674	9606.ENSP00000322421	0.672
NOP58	MPHOSPH10	9606.ENSP00000264279	9606.ENSP00000244230	0.999
NOP58	UTP3	9606.ENSP00000264279	9606.ENSP00000254803	0.996
NOP58	ESF1	9606.ENSP00000264279	9606.ENSP00000202816	0.979
PARG	RPL32	9606.ENSP00000384408	9606.ENSP00000380156	0.64
PARG	PARP4	9606.ENSP00000384408	9606.ENSP00000371419	0.615
PCNXL3	CP	9606.ENSP00000347931	9606.ENSP00000264613	0.418
PDSS2	COQ9	9606.ENSP00000358033	9606.ENSP00000262507	0.889
PDSS2	RETSAT	9606.ENSP00000358033	9606.ENSP00000295802	0.488
PDSS2	MVK	9606.ENSP00000358033	9606.ENSP00000228510	0.404
PEAK1	CRK	9606.ENSP00000309230	9606.ENSP00000300574	0.646
PFKFB2	HKDC1	9606.ENSP00000356047	9606.ENSP00000346643	0.928
PFKFB2	ENO3	9606.ENSP00000356047	9606.ENSP00000324105	0.585
RAB10	TBC1D17	9606.ENSP00000264710	9606.ENSP00000221543	0.416
RAB33B	RAB10	9606.ENSP00000306496	9606.ENSP00000264710	0.637
RAB7A	TBC1D17	9606.ENSP00000265062	9606.ENSP00000221543	0.708
RAB7A	RAB10	9606.ENSP00000265062	9606.ENSP00000264710	0.519
RAB7A	SNX3	9606.ENSP00000265062	9606.ENSP00000230085	0.509
RABIF	RAB10	9606.ENSP00000356231	9606.ENSP00000264710	0.843
RABIF	RAB33B	9606.ENSP00000356231	9606.ENSP00000306496	0.721
RAD23B	ACTB	9606.ENSP00000350708	9606.ENSP00000349960	0.917
RAD23B	COPS7B	9606.ENSP00000350708	9606.ENSP00000272995	0.905
RAD23B	SPATA5L1	9606.ENSP00000350708	9606.ENSP00000305494	0.662
RAD23B	TCEA2	9606.ENSP00000350708	9606.ENSP00000343515	0.446
RAD23B	NEDD4	9606.ENSP00000350708	9606.ENSP00000345530	0.41
RIOK3	MPHOSPH10	9606.ENSP00000341874	9606.ENSP00000244230	0.973
RIOK3	UTP15	9606.ENSP00000341874	9606.ENSP00000296792	0.922
RIOK3	UTP3	9606.ENSP00000341874	9606.ENSP00000254803	0.919
RIOK3	NOP58	9606.ENSP00000341874	9606.ENSP00000264279	0.914
RIOK3	CASP10	9606.ENSP00000341874	9606.ENSP00000286186	0.663
RIOK3	ESF1	9606.ENSP00000341874	9606.ENSP00000202816	0.552
RNF213	UBR1	9606.ENSP00000324392	9606.ENSP00000290650	0.909
RNF213	FBXO22	9606.ENSP00000324392	9606.ENSP00000307833	0.9
RNF213	RNF114	9606.ENSP00000324392	9606.ENSP00000244061	0.9
RNF34	UBR1	9606.ENSP00000376258	9606.ENSP00000290650	0.904
RNF34	RNF213	9606.ENSP00000376258	9606.ENSP00000324392	0.903
RNF34	FBXO22	9606.ENSP00000376258	9606.ENSP00000307833	0.9
RNF34	KLHL9	9606.ENSP00000376258	9606.ENSP00000351933	0.9
RNF34	NEDD4	9606.ENSP00000376258	9606.ENSP00000345530	0.9
RNF34	RNF114	9606.ENSP00000376258	9606.ENSP00000244061	0.9

RNF34	UBE2Z	9606.ENSP00000376258	9606.ENSP00000354201	0.9
RNF34	FBXO2	9606.ENSP00000376258	9606.ENSP00000346240	0.9
RPF2	NOP58	9606.ENSP00000402338	9606.ENSP00000264279	0.967
RPF2	UTP15	9606.ENSP00000402338	9606.ENSP00000296792	0.965
RPF2	WDR43	9606.ENSP00000402338	9606.ENSP00000384302	0.962
RPF2	UTP3	9606.ENSP00000402338	9606.ENSP00000254803	0.928
RPF2	MPHOSPH10	9606.ENSP00000402338	9606.ENSP00000244230	0.902
RPF2	DDX10	9606.ENSP00000402338	9606.ENSP00000314348	0.891
RPF2	GNL1	9606.ENSP00000402338	9606.ENSP00000365806	0.822
RPF2	HEATR1	9606.ENSP00000402338	9606.ENSP00000355541	0.818
RPF2	ESF1	9606.ENSP00000402338	9606.ENSP00000202816	0.723
RPF2	TRMT61A	9606.ENSP00000402338	9606.ENSP00000374399	0.579
RPF2	FKBP14	9606.ENSP00000402338	9606.ENSP00000222803	0.493
RPF2	FKBP1A	9606.ENSP00000402338	9606.ENSP00000371138	0.491
RPF2	FKBP9	9606.ENSP00000402338	9606.ENSP00000242209	0.491
RPL32	SEC61G	9606.ENSP00000380156	9606.ENSP00000341538	0.914
RPL32	SSR1	9606.ENSP00000380156	9606.ENSP00000244763	0.901
RPL32	TRAM1	9606.ENSP00000380156	9606.ENSP00000262213	0.9
RSF1	HIST1H2BD	9606.ENSP00000311513	9606.ENSP00000289316	0.9
RSF1	SSRP1	9606.ENSP00000311513	9606.ENSP00000278412	0.427
RSF1	SUPT16H	9606.ENSP00000311513	9606.ENSP00000216297	0.424
RSL1D1	NOP58	9606.ENSP00000460871	9606.ENSP00000264279	0.993
RSL1D1	UTP15	9606.ENSP00000460871	9606.ENSP00000296792	0.983
RSL1D1	RPF2	9606.ENSP00000460871	9606.ENSP00000402338	0.982
RSL1D1	WDR43	9606.ENSP00000460871	9606.ENSP00000384302	0.968
RSL1D1	HEATR1	9606.ENSP00000460871	9606.ENSP00000355541	0.939
RSL1D1	MPHOSPH10	9606.ENSP00000460871	9606.ENSP00000244230	0.9
RSL1D1	GNL1	9606.ENSP00000460871	9606.ENSP00000365806	0.88
RSL1D1	DDX10	9606.ENSP00000460871	9606.ENSP00000314348	0.873
RSL1D1	ESF1	9606.ENSP00000460871	9606.ENSP00000202816	0.872
RSL1D1	UTP3	9606.ENSP00000460871	9606.ENSP00000254803	0.825
RSL1D1	FKBP14	9606.ENSP00000460871	9606.ENSP00000222803	0.451
RSL1D1	FKBP9	9606.ENSP00000460871	9606.ENSP00000242209	0.451
RSL1D1	FKBP1A	9606.ENSP00000460871	9606.ENSP00000371138	0.451
S100A16	TMED10	9606.ENSP00000357692	9606.ENSP00000303145	0.774
SAP18	H2AFX	9606.ENSP00000371973	9606.ENSP00000364310	0.903
SAP18	HIST1H2BD	9606.ENSP00000371973	9606.ENSP00000289316	0.9
SAP18	HDAC5	9606.ENSP00000371973	9606.ENSP00000225983	0.821
SAP18	SSRP1	9606.ENSP00000371973	9606.ENSP00000278412	0.788
SAP18	CBX5	9606.ENSP00000371973	9606.ENSP00000209875	0.551
SAP18	SUPT16H	9606.ENSP00000371973	9606.ENSP00000216297	0.454
SAP18	VTN	9606.ENSP00000371973	9606.ENSP00000226218	0.451

SARDH	AGXT	9606.ENSP00000360938	9606.ENSP00000302620	0.938
SARDH	AHCYL2	9606.ENSP00000360938	9606.ENSP00000315931	0.495
SDC4	SDCBP	9606.ENSP00000361818	9606.ENSP00000260130	0.945
SDC4	VTN	9606.ENSP00000361818	9606.ENSP0000226218	0.931
SDC4	TGFB1	9606.ENSP00000361818	9606.ENSP00000221930	0.926
SDC4	APOE	9606.ENSP00000361818	9606.ENSP00000252486	0.916
SDC4	APOA2	9606.ENSP00000361818	9606.ENSP00000356969	0.9
SDC4	COL4A2	9606.ENSP00000361818	9606.ENSP00000353654	0.805
SDC4	TGM2	9606.ENSP00000361818	9606.ENSP00000355330	0.543
SDCBP	SNTA1	9606.ENSP00000260130	9606.ENSP00000217381	0.7
SDPR	ANKRD52	9606.ENSP00000305675	9606.ENSP00000267116	0.52
SEC14L1	MOSPD2	9606.ENSP00000376268	9606.ENSP00000369860	0.581
SEC14L1	DDX58	9606.ENSP00000376268	9606.ENSP00000369213	0.416
SEC61G	SSR1	9606.ENSP00000341538	9606.ENSP00000244763	0.942
SEC61G	TRAM1	9606.ENSP00000341538	9606.ENSP00000262213	0.908
SEC61G	TMEM214	9606.ENSP00000341538	9606.ENSP00000238788	0.73
SEC61G	TMED2	9606.ENSP00000341538	9606.ENSP00000262225	0.437
SLC2A1	TOP2A	9606.ENSP00000416293	9606.ENSP00000411532	0.553
SLC2A1	HBA1	9606.ENSP00000416293	9606.ENSP00000322421	0.46
SLC2A1	SLC7A5	9606.ENSP00000416293	9606.ENSP00000261622	0.431
SLC30A1	SLC39A14	9606.ENSP00000355968	9606.ENSP00000289952	0.752
SNTB1	SNTA1	9606.ENSP00000378965	9606.ENSP00000217381	0.727
SNTB1	SDCBP	9606.ENSP00000378965	9606.ENSP00000260130	0.524
SNX14	SNX3	9606.ENSP00000313121	9606.ENSP00000230085	0.441
SON	NAGPA	9606.ENSP00000348984	9606.ENSP00000310998	0.629
SON	AMBP	9606.ENSP00000348984	9606.ENSP00000265132	0.605
SON	FER	9606.ENSP00000348984	9606.ENSP00000281092	0.475
SPATA5L1	SSRP1	9606.ENSP00000305494	9606.ENSP00000278412	0.432
SSRP1	SUPT16H	9606.ENSP00000278412	9606.ENSP00000216297	0.999
SSRP1	ASF1B	9606.ENSP00000278412	9606.ENSP00000263382	0.725
STMN1	S100A16	9606.ENSP00000410452	9606.ENSP00000357692	0.503
STMN1	TPM3	9606.ENSP00000410452	9606.ENSP00000357516	0.466
SYNE1	HIST1H2BD	9606.ENSP00000356224	9606.ENSP00000289316	0.902
SYNE1	HIST3H3	9606.ENSP00000356224	9606.ENSP00000355657	0.9
TCEA2	ELP4	9606.ENSP00000343515	9606.ENSP00000298937	0.901
TCEA2	SUPT16H	9606.ENSP00000343515	9606.ENSP00000216297	0.619
TCEA2	SSRP1	9606.ENSP00000343515	9606.ENSP00000278412	0.495
TCEA3	SUPT16H	9606.ENSP00000406293	9606.ENSP00000216297	0.592
TCEA3	SSRP1	9606.ENSP00000406293	9606.ENSP00000278412	0.492
TCEA3	RAD23B	9606.ENSP00000406293	9606.ENSP00000350708	0.439
TCF7L2	JUN	9606.ENSP00000444972	9606.ENSP00000360266	0.922
TCF7L2	H2AFX	9606.ENSP00000444972	9606.ENSP00000364310	0.906

TCF7L2	H2AFJ	9606.ENSP00000444972	9606.ENSP00000373730	0.906
TCF7L2	HIST3H3	9606.ENSP00000444972	9606.ENSP00000355657	0.904
TCF7L2	HIST2H3A	9606.ENSP00000444972	9606.ENSP00000385479	0.904
TCF7L2	HIST1H2BD	9606.ENSP00000444972	9606.ENSP00000289316	0.901
TCF7L2	HIST3H2BB	9606.ENSP00000444972	9606.ENSP00000375736	0.9
TCF7L2	WNT2B	9606.ENSP00000444972	9606.ENSP00000358698	0.696
TCF7L2	CDKN2A	9606.ENSP00000444972	9606.ENSP00000394932	0.429
TF	ITSN2	9606.ENSP00000385834	9606.ENSP00000347244	0.95
TF	FNBP1L	9606.ENSP00000385834	9606.ENSP00000271234	0.901
TF	TCIRG1	9606.ENSP00000385834	9606.ENSP00000265686	0.901
TF	CP	9606.ENSP00000385834	9606.ENSP00000264613	0.859
TF	APOA2	9606.ENSP00000385834	9606.ENSP00000356969	0.772
TF	AMBP	9606.ENSP00000385834	9606.ENSP00000265132	0.734
TF	APOE	9606.ENSP00000385834	9606.ENSP00000252486	0.711
TF	LYZ	9606.ENSP00000385834	9606.ENSP00000261267	0.575
TF	JUN	9606.ENSP00000385834	9606.ENSP00000360266	0.548
TF	TM9SF2	9606.ENSP00000385834	9606.ENSP00000365567	0.532
TF	RAB7A	9606.ENSP00000385834	9606.ENSP00000265062	0.532
TF	FTL	9606.ENSP00000385834	9606.ENSP00000366525	0.526
TF	VTN	9606.ENSP00000385834	9606.ENSP00000226218	0.524
TF	TGFB1	9606.ENSP00000385834	9606.ENSP00000221930	0.524
TF	ASGR1	9606.ENSP00000385834	9606.ENSP00000269299	0.424
TF	HBA1	9606.ENSP00000385834	9606.ENSP00000322421	0.414
TF	SLC39A14	9606.ENSP00000385834	9606.ENSP00000289952	0.41
TF	ACTB	9606.ENSP00000385834	9606.ENSP00000349960	0.408
TGM2	GNG2	9606.ENSP00000355330	9606.ENSP00000334448	0.9
TGM2	ACTB	9606.ENSP00000355330	9606.ENSP00000349960	0.469
TGM2	KRT19	9606.ENSP00000355330	9606.ENSP00000355124	0.439
TGOLN2	TF	9606.ENSP00000386443	9606.ENSP00000385834	0.95
TGOLN2	ITSN2	9606.ENSP00000386443	9606.ENSP00000347244	0.912
TGOLN2	FTL	9606.ENSP00000386443	9606.ENSP00000366525	0.9
TGOLN2	FNBP1L	9606.ENSP00000386443	9606.ENSP00000271234	0.9
TGOLN2	RAB7A	9606.ENSP00000386443	9606.ENSP00000265062	0.514
TM9SF2	15-Sep	9606.ENSP00000365567	9606.ENSP00000328729	0.444
TMED10	TMED2	9606.ENSP00000303145	9606.ENSP00000262225	0.999
TMED10	USP8	9606.ENSP00000303145	9606.ENSP00000302239	0.431
TOP1	SUPT16H	9606.ENSP00000354522	9606.ENSP00000216297	0.842
TOP1	TOPBP1	9606.ENSP00000354522	9606.ENSP00000260810	0.564
TOP1	XRCC6	9606.ENSP00000354522	9606.ENSP00000352257	0.506
TOP1	SON	9606.ENSP00000354522	9606.ENSP00000348984	0.47
TOP1	ASF1B	9606.ENSP00000354522	9606.ENSP00000263382	0.459
TOP1	HIST1H4A	9606.ENSP00000354522	9606.ENSP00000352980	0.404

TOP2A	TOP1	9606.ENSP00000411532	9606.ENSP00000354522	0.998
TOP2A	SSRP1	9606.ENSP00000411532	9606.ENSP00000278412	0.892
TOP2A	KIF15	9606.ENSP00000411532	9606.ENSP00000324020	0.863
TOP2A	H2AFX	9606.ENSP00000411532	9606.ENSP00000364310	0.744
TOP2A	TOPBP1	9606.ENSP00000411532	9606.ENSP00000260810	0.744
TOP2A	ASF1B	9606.ENSP00000411532	9606.ENSP00000263382	0.702
TOP2A	RRM2B	9606.ENSP00000411532	9606.ENSP00000251810	0.648
TOP2A	XRCC5	9606.ENSP00000411532	9606.ENSP00000375977	0.643
TOP2A	PPIL4	9606.ENSP00000411532	9606.ENSP00000253329	0.617
TOP2A	MGMT	9606.ENSP00000411532	9606.ENSP00000302111	0.578
TOP2A	DDX10	9606.ENSP00000411532	9606.ENSP00000314348	0.559
TOP2A	HSPH1	9606.ENSP00000411532	9606.ENSP00000318687	0.557
TOP2A	PRPS2	9606.ENSP00000411532	9606.ENSP00000381504	0.556
TOP2A	ADSSL1	9606.ENSP00000411532	9606.ENSP00000333019	0.552
TOP2A	UBE2T	9606.ENSP00000411532	9606.ENSP00000356243	0.552
TOP2A	JUN	9606.ENSP00000411532	9606.ENSP00000360266	0.548
TOP2A	MOCOS	9606.ENSP00000411532	9606.ENSP00000261326	0.545
TOP2A	DLG3	9606.ENSP00000411532	9606.ENSP00000363480	0.536
TOP2A	H3F3A	9606.ENSP00000411532	9606.ENSP00000355778	0.536
TOP2A	SUPT16H	9606.ENSP00000411532	9606.ENSP00000216297	0.529
TOP2A	XRCC6	9606.ENSP00000411532	9606.ENSP00000352257	0.525
TOP2A	PUS3	9606.ENSP00000411532	9606.ENSP00000227474	0.523
TOP2A	SPNS1	9606.ENSP00000411532	9606.ENSP00000309945	0.52
TOP2A	ENO3	9606.ENSP00000411532	9606.ENSP00000324105	0.515
TOP2A	H2AFY2	9606.ENSP00000411532	9606.ENSP00000362352	0.514
TOP2A	GSTT1	9606.ENSP00000411532	9606.ENSP00000248935	0.511
TOP2A	ETNK1	9606.ENSP00000411532	9606.ENSP00000266517	0.51
TOP2A	HBA1	9606.ENSP00000411532	9606.ENSP00000322421	0.498
TOP2A	H2AFJ	9606.ENSP00000411532	9606.ENSP00000373730	0.49
TOP2A	HIST1H4A	9606.ENSP00000411532	9606.ENSP00000352980	0.479
TOP2A	SLC7A5	9606.ENSP00000411532	9606.ENSP00000261622	0.467
TOP2A	GMPR2	9606.ENSP00000411532	9606.ENSP00000392859	0.465
TOP2A	PDSS2	9606.ENSP00000411532	9606.ENSP00000358033	0.461
TOP2A	CLCN3	9606.ENSP00000411532	9606.ENSP00000261514	0.458
TOP2A	RETSAT	9606.ENSP00000411532	9606.ENSP00000295802	0.447
TOP2A	ACTB	9606.ENSP00000411532	9606.ENSP00000349960	0.446
TOP2A	HDAC5	9606.ENSP00000411532	9606.ENSP00000225983	0.444
TOP2A	KCNAB2	9606.ENSP00000411532	9606.ENSP00000367323	0.44
TOP2A	SLC12A4	9606.ENSP00000411532	9606.ENSP00000395983	0.439
TOP2A	AIFM2	9606.ENSP00000411532	9606.ENSP00000312370	0.438
TOP2A	CDKN2A	9606.ENSP00000411532	9606.ENSP00000394932	0.431
TPM3	ACTB	9606.ENSP00000357516	9606.ENSP00000349960	0.887

TPM3	ACTG1	9606.ENSP00000357516	9606.ENSP00000331514	0.826
TPM3	MYO5B	9606.ENSP00000357516	9606.ENSP00000285039	0.604
TPM3	SYNE1	9606.ENSP00000357516	9606.ENSP00000356224	0.464
TRA2B	HNRNPA3	9606.ENSP00000416959	9606.ENSP00000376309	0.906
TRA2B	TPM3	9606.ENSP00000416959	9606.ENSP00000357516	0.526
TRAM1	SSR1	9606.ENSP00000262213	9606.ENSP00000244763	0.951
TRMT61A	MPHOSPH10	9606.ENSP00000374399	9606.ENSP00000244230	0.708
TRMT61A	ESF1	9606.ENSP00000374399	9606.ENSP00000202816	0.605
TRMT61A	UTP15	9606.ENSP00000374399	9606.ENSP00000296792	0.524
TRMT61A	PUS3	9606.ENSP00000374399	9606.ENSP00000227474	0.454
TSC22D3	SGK3	9606.ENSP00000314655	9606.ENSP00000262211	0.913
TSTA3	GCDH	9606.ENSP00000398803	9606.ENSP00000222214	0.478
TSTA3	RABIF	9606.ENSP00000398803	9606.ENSP00000356231	0.424
TTC39C	CRK	9606.ENSP00000323645	9606.ENSP00000300574	0.478
TYMP	RRM2B	9606.ENSP00000252029	9606.ENSP00000251810	0.703
UBE2T	TOPBP1	9606.ENSP00000356243	9606.ENSP00000260810	0.915
UBE2T	UBR1	9606.ENSP00000356243	9606.ENSP00000290650	0.461
UBE2T	RAD23B	9606.ENSP00000356243	9606.ENSP00000350708	0.445
UBE2T	NEDD4	9606.ENSP00000356243	9606.ENSP00000345530	0.432
UBE2T	ASF1B	9606.ENSP00000356243	9606.ENSP00000263382	0.418
UBE2Z	UBR1	9606.ENSP00000354201	9606.ENSP00000290650	0.959
UBE2Z	NEDD4	9606.ENSP00000354201	9606.ENSP00000345530	0.938
UBE2Z	KLHL9	9606.ENSP00000354201	9606.ENSP00000351933	0.909
UBE2Z	RNF114	9606.ENSP00000354201	9606.ENSP00000244061	0.9
UBE2Z	FBXO22	9606.ENSP00000354201	9606.ENSP00000307833	0.9
UBE2Z	FBXO2	9606.ENSP00000354201	9606.ENSP00000346240	0.9
UBE2Z	RNF213	9606.ENSP00000354201	9606.ENSP00000324392	0.9
UBE2Z	RAD23B	9606.ENSP00000354201	9606.ENSP00000350708	0.423
UBR1	RNF114	9606.ENSP00000290650	9606.ENSP00000244061	0.9
UIMC1	H2AFX	9606.ENSP00000366434	9606.ENSP00000364310	0.964
UIMC1	XRCC6	9606.ENSP00000366434	9606.ENSP00000352257	0.921
UIMC1	HIST1H2BD	9606.ENSP00000366434	9606.ENSP00000289316	0.902
UIMC1	HIST3H3	9606.ENSP00000366434	9606.ENSP00000355657	0.9
UIMC1	FAM175B	9606.ENSP00000366434	9606.ENSP00000298492	0.866
UTP15	NOP58	9606.ENSP00000296792	9606.ENSP00000264279	0.999
UTP15	MPHOSPH10	9606.ENSP00000296792	9606.ENSP00000244230	0.999
UTP15	UTP3	9606.ENSP00000296792	9606.ENSP00000254803	0.964
UTP15	ESF1	9606.ENSP00000296792	9606.ENSP00000202816	0.667
UTP15	DPH2	9606.ENSP00000296792	9606.ENSP00000255108	0.463
UTP3	MPHOSPH10	9606.ENSP00000254803	9606.ENSP00000244230	0.999
UTP3	ESF1	9606.ENSP00000254803	9606.ENSP00000202816	0.988
VTN	TGFB1	9606.ENSP00000226218	9606.ENSP00000221930	0.634

WDR43	HEATR1	9606.ENSP00000384302	9606.ENSP00000355541	0.999
WDR43	UTP15	9606.ENSP00000384302	9606.ENSP00000296792	0.999
WDR43	NOP58	9606.ENSP00000384302	9606.ENSP00000264279	0.998
WDR43	MPHOSPH10	9606.ENSP00000384302	9606.ENSP00000244230	0.998
WDR43	UTP3	9606.ENSP00000384302	9606.ENSP00000254803	0.992
WDR43	RIOK3	9606.ENSP00000384302	9606.ENSP00000341874	0.92
WDR43	GNL1	9606.ENSP00000384302	9606.ENSP00000365806	0.857
WDR43	ESF1	9606.ENSP00000384302	9606.ENSP00000202816	0.851
WDR43	DDX10	9606.ENSP00000384302	9606.ENSP00000314348	0.817
WDR43	TRMT61A	9606.ENSP00000384302	9606.ENSP00000374399	0.447
XRCC5	XRCC6	9606.ENSP00000375977	9606.ENSP00000352257	0.999
XRCC5	H2AFX	9606.ENSP00000375977	9606.ENSP00000364310	0.982
XRCC5	RAD23B	9606.ENSP00000375977	9606.ENSP00000350708	0.926
XRCC5	UIMC1	9606.ENSP00000375977	9606.ENSP00000366434	0.923
XRCC5	HIST3H3	9606.ENSP00000375977	9606.ENSP00000355657	0.907
XRCC5	HIST1H2BD	9606.ENSP00000375977	9606.ENSP00000289316	0.901
XRCC5	HIST3H2BB	9606.ENSP00000375977	9606.ENSP00000375736	0.901
XRCC5	TOP1	9606.ENSP00000375977	9606.ENSP00000354522	0.639
XRCC5	JUN	9606.ENSP00000375977	9606.ENSP00000360266	0.554
XRCC5	SSRP1	9606.ENSP00000375977	9606.ENSP00000278412	0.553
XRCC5	SUPT16H	9606.ENSP00000375977	9606.ENSP00000216297	0.527
XRCC6	RAD23B	9606.ENSP00000352257	9606.ENSP00000350708	0.919
XRCC6	HIST1H2BD	9606.ENSP00000352257	9606.ENSP00000289316	0.907
XRCC6	CBX5	9606.ENSP00000352257	9606.ENSP00000209875	0.653
XRCC6	HIST1H3A	9606.ENSP00000352257	9606.ENSP00000350275	0.585
ZNF516	HIST3H3	9606.ENSP00000394757	9606.ENSP00000355657	0.479
